# Axially Chiral Cyclic Diphosphine Ligand-Enabled Palladium-Catalyzed Intramolecular Asymmetric Hydroarylation

**DOI:** 10.1016/j.isci.2018.11.018

**Published:** 2018-11-14

**Authors:** Can Liu, Xianjin Zhu, Pengxiang Zhang, Haijun Yang, Changjin Zhu, Hua Fu

**Affiliations:** 1 School of Chemistry and Chemical Engineering, Beijing Institute of Technology, Beijing, China; 2 Key Laboratory of Bioorganic Phosphorus Chemistry and Chemical Biology (Ministry of Education), Department of Chemistry, Tsinghua University, Beijing, China

**Keywords:** Chemistry, Catalysis, Organic Chemistry, Stereochemistry

## Abstract

In transition metal-catalyzed asymmetric synthesis, enantioselectivity strongly depends on the structures of chiral ligands, so the development of new chiral ligands is crucial. Here, an efficient and highly enantioselective palladium-catalyzed intramolecular hydroarylation has been developed, and a new kind of N-heterocycles, 1H-pyrazolo[5,1-a]isoindol-2(8H)-ones containing a quaternary stereocenter, was prepared in high yields and excellent enantiomeric excess values. The reaction was effectively catalyzed by palladium-diphosphine complexes with numerous functional group tolerance, in which the newly developed axially chiral cyclic diphosphine ligands played key roles in the reactivity and enantioselectivity of the substrates. We believe that the cyclic diphosphine ligands with adjustable dihedral angles will find wide application in asymmetric synthesis.

## Highlights

• Novel axially chiral cyclic diphosphine ligands
• Excellent enantioselectivity
• Wide substrate scope
• Synthesis of a new kind of chiral N-heterocycles

## Graphical Abstract

**Figure:**
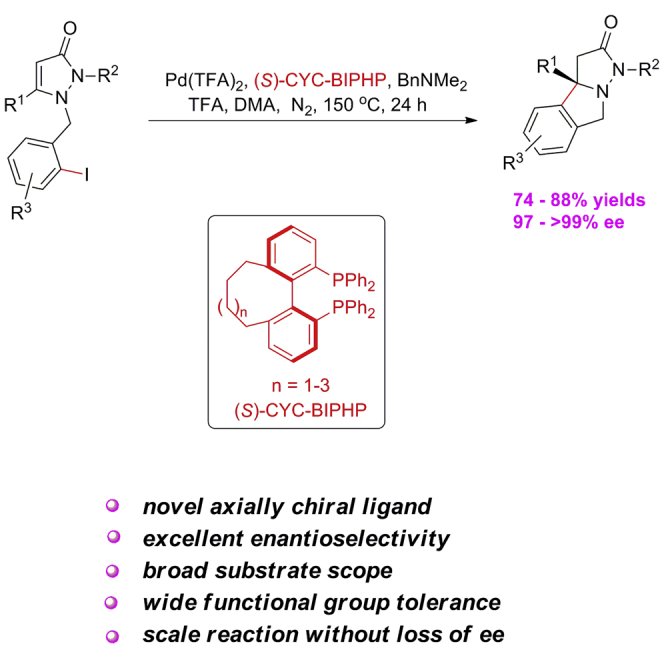

## Introduction

**Figure 1:**
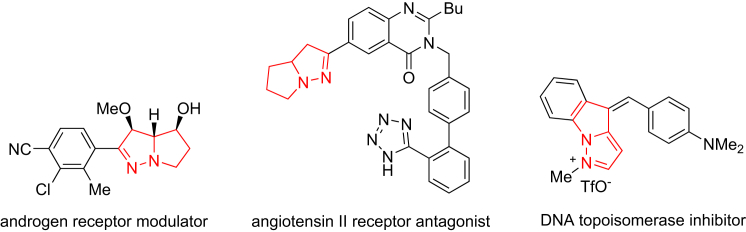
Selected Bioactive Compounds with a Diazabicyclo[3.3.0]octane Skeleton

Nitrogen-containing compounds widely occur in biologically active molecules including natural products (Ruiz-Sanchis et al., 2011), agrochemicals, and pharmaceuticals (Leeson and Springthorpe, 2007). In particular, over 90% of pharmaceuticals contain at least one nitrogen atom in their structures, so the development of efficient approaches to *N*-heterocycles is of paramount importance (Carey et al., 2006, Duggers et al., 2005). Compounds containing a l,8-diazabicyclo[3.3.0]octane skeleton exhibit diverse biological activities. For example, they are used as the androgen receptor modulator (Ullrich et al., 2014), angiotensin II receptor antagonist (Levin et al., 1994), and DNA topoisomerase inhibitor (Figure 1) (Katayama et al., 1999). However, 1*H*-pyrazolo[5,1-*a*]isoindol-2(8*H*)-ones as their derivatives have been ignored (Ivanovich et al., 2016). To the best of our knowledge, enantioselective synthesis of this kind of compounds containing a quaternary stereocenter has not been reported thus far.

Since the pioneering work by Cacchi and co-workers (Cacchi and Arcadi, 1983, Amorese et al., 1989; Cacchi, 1990, Arcadi et al., 1996), the palladium-catalyzed hydroarylation or reductive Heck reaction of aryl halides (pseudohalides) with alkenes has attracted much attention (Trost and Toste, 1999, Lee and Cha, 2001, Ichikawa et al., 2004, Dounay et al., 2008, Diethelm and Carreira, 2013, Schmidt and Hoffmann, 1991, Gottumukkala et al., 2011, Chen et al., 2012, Gao and Cook, 2012, Raoufmoghaddam et al., 2015). However, the development of highly enantioselective hydroarylation is still a great challenge, and only some examples of the enantioselective protocols have been reported till now (Minatti et al., 2007, Mannathan et al., 2017, Liu and Zhou, 2013, Yue et al., 2015, Shen et al., 2015, Kong et al., 2017). It is well known that the enantioselectivity highly depends on structures of chiral ligands in the transition-metal-catalyzed asymmetric synthesis, so the development of new chiral ligands is crucial (Tang and Zhang, 2003, Noyori and Ohkuma, 2001). In this regard, the axially chiral diphosphine ligands have been proved to be highly efficient in various enantioselective transformations (Qiu et al., 2006, Zhang et al., 2000, Sun et al., 2008, Wu et al., 2005, Pai et al., 2000, Jeulin et al., 2004a, Jeulin et al., 2004b, Genêt, 2003, Benincori et al., 2000, Tietze et al., 2000, Hatano et al., 2001, Graff et al., 2015). Recently, we have developed a kind of novel axially chiral cyclo-[1,1′-biphenyl]-2,2′-diols (CYCNOL) with adjustable dihedral angles (Zhang et al., 2016), and the chiral cyclic phosphoramidite ligands derived from CYCNOL have been successfully applied in iridium-catalyzed enantioselective arylation of unactivated racemic secondary allylic alcohols (Tian et al., 2017) and synthesis of dihydroimidazoquinazolinones (Peng et al., 2017). Inspired by the ligands we developed (Zhang et al., 2016, Tian et al., 2017, Peng et al., 2017), we herein report a palladium-catalyzed intramolecular enantioselective hydroarylation by elaborate tuning of newly developed axially chiral cyclic diphosphine ligands derived from CYCNOL.

## Results and Discussion

### Synthesis of Ligands

**Figure 2:**
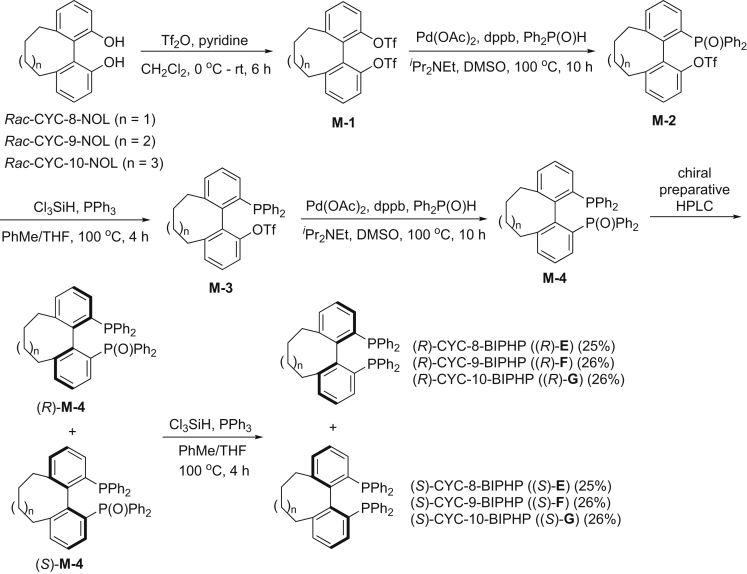
Synthesis of Axially Chiral Cyclic Diphosphine Ligands

Racemic CYCNOL, *Rac*-CYC-8-NOL, *Rac*-CYC-9-NOL, and *Rac*-CYC-10-NOL, were prepared according to our previous procedures (Zhang et al., 2016). Subsequently, synthesis (following Zhou's protocol [Xie et al., 2003]) and resolution of our axially chiral cyclic diphosphine ligands were performed (Figure 2) (see Supplemental Information for details).

### Crystal Structures of Ligands

**Figure 3:**
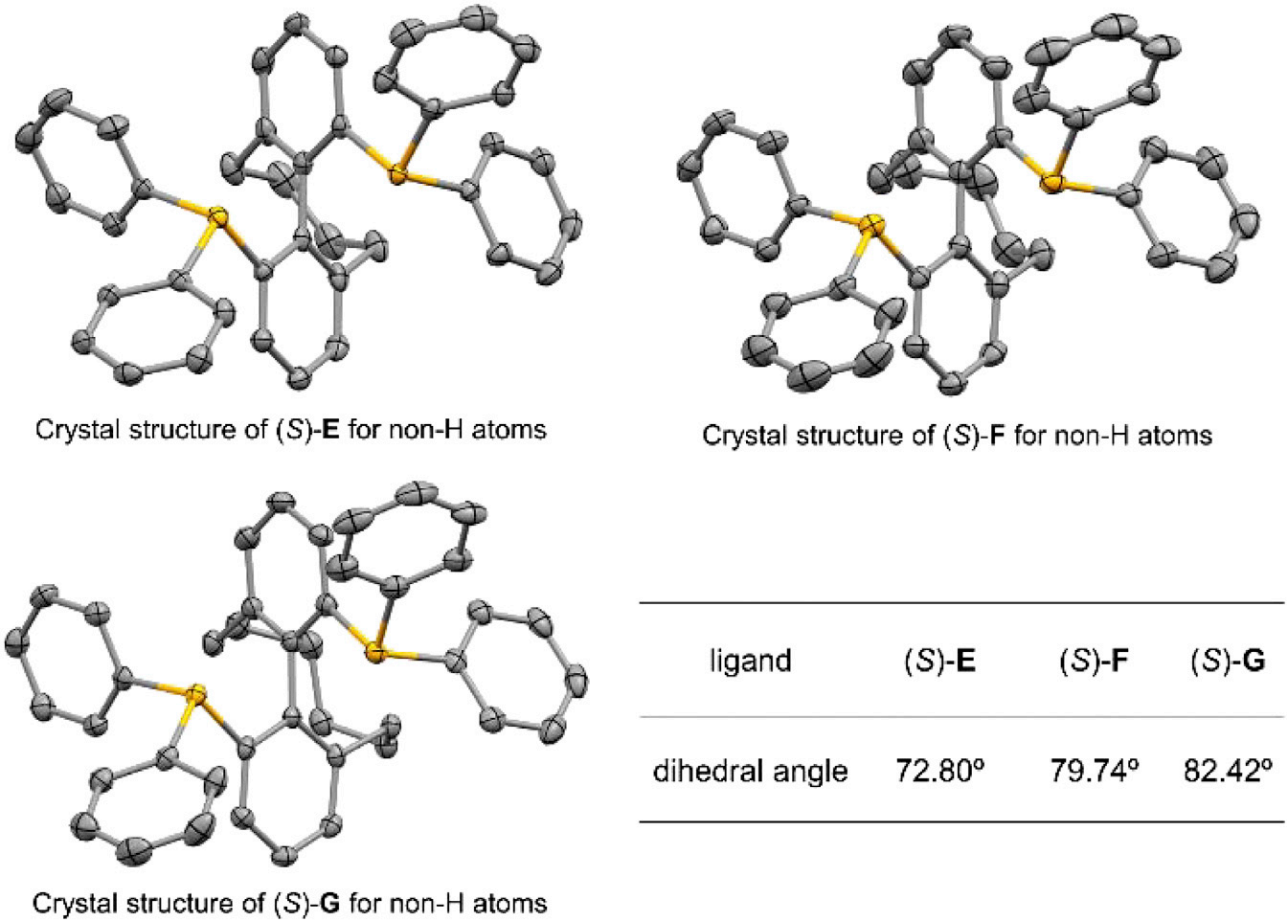
Crystal Structures and Dihedral Angles of Axially Chiral Cyclic Diphosphine Ligands (*S*)-**E**, (*S*)-**F**, and (*S*)-**G**

Single crystals of the axially chiral cyclic diphosphine ligands (*S*)-CYC-8-BIPHP ((*S*)-**E**), (*S*)-CYC-9-BIPHP ((*S*)-**F**), and (*S*)-CYC-10-BIPHP ((*S*)-**G**) from mixed hexane and dichloromethane solvent were prepared, and their structures were unambiguously confirmed by X-ray diffraction analysis (see Supplemental Information, Data S1, S2, and S3 for details). According to the data from X-ray diffraction analysis, dihedral angles of the diphosphine ligands showed remarkable difference with a variety of ring sizes (Figure 3). It is known to all that the reactivity and enantioselectivity of substrates in the transition metal asymmetric synthesis are closely related to the structures of the ligands, such as the dihedral angles of axially chiral ligands.

### Optimization Study

At first, palladium-catalyzed enantioselective hydroarylation of 1-(2-iodobenzyl)-5-methyl-2-phenyl-1*H*-pyrazol-3(2*H*)-one (**1a**) leading to (*S*)-3*a*-methyl-1-phenyl-3,3*a*-dihydro-1*H*-pyrazolo[5,1-*a*]isoindol-2(8*H*)-one (**2a**) was used as the model to optimize conditions including catalysts, ligands, tertiary amines, acids, solvents, and temperature. As shown in Table 1, seven ligands including four common diphosphine ligands, (*S*)- 2,2′- bis(diphenylphosphino)-1,1′-binaphthyl (BINAP), (*R*)- 5,5′-bis[di(3,5-di-t-butyl-4-methoxyphenyl)phosphino]-4,4′-bi-1,3-benzodioxole (DTBM-SEGPHOS), (*S*)-MeO-BIPNEP, and (*S*)- 7,7′-bis(diphenylphosphino)-2,2′,3,3′-tetrahydro-1,1′-spirobiindane (SDP), and our three cyclic diphosphine ligands, (*S*)-**E**, (*S*)-**F,** and (*S*)-**G**, were screened using Pd(trifluoroacetic acid [TFA])_2_ as the catalysts and *N*,*N*-dimethylbenzylamine/TFA as the hydride donors in *N*,*N*-dimethylacetamide (DMA) under a nitrogen atmosphere at 150°C for 24 hr (entries 1–7). We were pleased to find that the three cyclic diphosphine ligands, (*S*)-**E**, (*S*)-**F**, and (*S*)-**G**, all provided high yields with excellent enantiomeric excess (ee) values (entries 5–7), in which (*S*)-**F** was optimal (entry 6). Compared with the four common ligands, the advantage of our cyclo-[1,1′-biphenyl]diphosphine ligands, (*S*)-**E**, (*S*)-**F,** and (*S*)-**G**, is attributed to their combination of conformational rigidity and flexibility because they own the rigid biphenyl and the flexible full-carbon 6,6′-tethers. Meanwhile, the three cyclo-diphosphine ligands had little influence on the yields and ee values because of this factor. Single crystal of product **2a** in entry 6 from mixed hexane and dichloromethane solvent was prepared, and its absolute configuration was determined to be *S*-form based on its single-crystal X-ray analysis (Table 1) (see Supplemental Information and Data S4 for details). Racemic **2a** was obtained in 37% yield in the absence of ligand (entry 8). When other three tertiary amines, triethylamine, diisopropylethylamine, and proton sponge, were used instead of *N*,*N*-dimethylbenzylamine, lower ee values were observed (entries 9–11). Only a small amount of product **2a** was found in the absence of amine (entry 12). Use of HOAc or HCOOH or absence of acid led to lower yields (entries 13–15). Two more palladium catalysts, Pd(dba)_2_ and Pd(OAc)_2_, were tested (entries 16 and 17), and they were inferior to Pd(TFA)_2_ (compare entries 6, 16, and 17). The effect of solvents was surveyed, and DMA proved to be a suitable solvent (compare entries 6, 18, and 19). When ligand (*S*)-**F** was increased from 7.5 mol % to 10 mol % (entry 20), the same yield and ee value were observed (compare entries 6 and 20). We attempted variation of temperature (entries 21 and 22), and the results showed that 150°C was a suitable temperature (compare entries 6, 21, and 22). According to the aforementioned results, we think that Pd(TFA)_2_ as the catalyst; (*S*)-**E**, (*S*)-**F,** and (*S*)-**G** as the ligands; *N*,*N*-dimethylbenzylamine/TFA as the hydride donor; and DMA as the solvent are suitable in the present palladium-catalyzed intramolecular enantioselective hydroarylation.

**Table 1:** Optimization of Conditions


Entry	Ligand	Amine	Acid	Yield of 2a (%)^a^	ee of 2a (%)^b^
1	(*S*)-**A**	BnNMe_2_	TFA	68	23
2	(*R*)-**B**	BnNMe_2_	TFA	31	−59
3	(*S*)-**C**	BnNMe_2_	TFA	63	28
4	**(*S*)-D**	BnNMe_2_	TFA	73	−2
5	(*S*)-**E**	BnNMe_2_	TFA	70	96
**6**	**(*S*)-F**	**BnNMe**_2_	**TFA**	**76**	**97**
7	(*S*)-**G**	BnNMe_2_	TFA	73	96
8	–	BnNMe_2_	TFA	37	0
9	(*S*)-**F**	NEt_3_	TFA	76	93
10	(*S*)-**F**	DIPEA	TFA	75	92
11	(*S*)-**F**	PS	TFA	57	88
12	(*S*)-**F**	–	TFA	8	94
13	(*S*)-**F**	BnNMe_2_	HOAc	48	95
14	(*S*)-**F**	BnNMe_2_	HCOOH	37	96
15	(*S*)-**F**	BnNMe_2_	–	35	96
16^c^	(*S*)-**F**	BnNMe_2_	TFA	51	95
17^d^	(*S*)-**F**	BnNMe_2_	TFA	63	96
18^e^	(*S*)-**F**	BnNMe_2_	TFA	62	96
19^f^	(*S*)-**F**	BnNMe_2_	TFA	56	95
20^g^	(*S*)-**F**	BnNMe_2_	TFA	76	97
21^h^	(*S*)-**F**	BnNMe_2_	TFA	38	97
22^i^	(*S*)-**F**	BnNMe_2_	TFA	76	96

Reaction conditions: under nitrogen atmosphere, 1-(2-iodobenzyl)-5-methyl-2-phenyl-1*H*-pyrazol-3(2*H*)-one (**1a**) (0.2 mmol, 1.0 equiv), Pd(TFA)_2_ (10 μmol, 5 mol%), ligand (15 μmol, 7.5 mol%), amine (1.0 mmol, 5 equiv), acid (0.4 mmol, 2 equiv), *N*,*N*-dimethylacetamide (DMA) (4.0 mL), temperature (150^°^C), time (24 hr) in a sealed Schlenk tube. Absolute configuration of (*S*)-**2a** was assigned by X-ray diffraction analysis.

PS, proton sponge; DMF, *N,N*-dimethylformamide; DMSO, dimethylsulfoxide.

a Isolated yield.

b The ee values were determined by high-performance liquid chromatography analysis.

c Using Pd(dba)_2_ (10 μmol, 5 mol%) as the catalyst.

d Using Pd(OAc)_2_ (10 μmol, 5 mol%) as the catalyst.

e Using DMF (4.0 mL) as the solvent.

f Using DMSO (4.0 mL) as the solvent.

g Using (*S*)-**F** (20 μmol, 10 mol%) as the ligand.

h The reaction was carried out at 130°C.

i The reaction was carried out at 160°C.

### Scope of the Investigation

**Figure 4:**
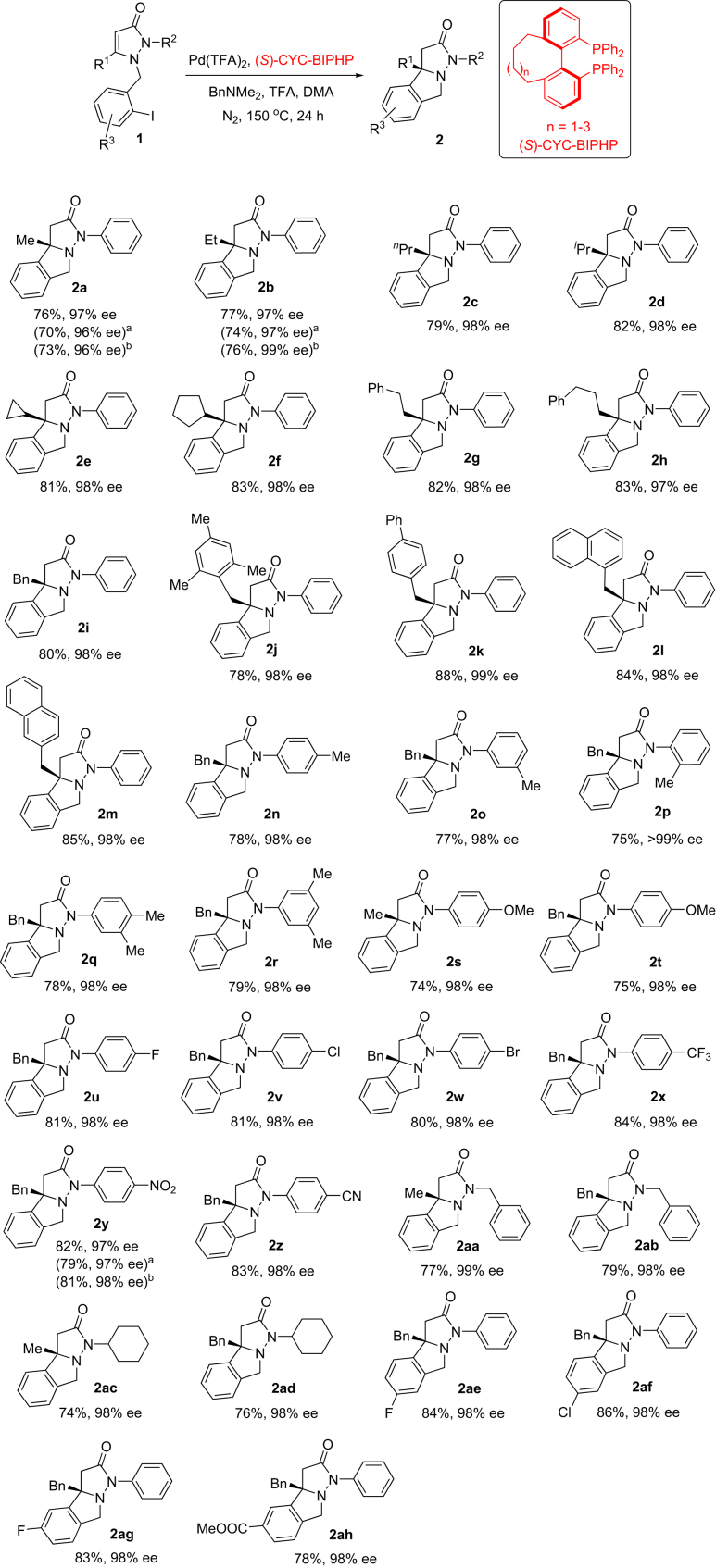
Substrate Scope for Palladium-Catalyzed Asymmetric Cyclization of 1 Reaction conditions: under nitrogen atmosphere, 1-(2-iodobenzyl)-5-alkyl-2-alkyl-1*H*-pyrazol-3(2*H*)-one (**1**) (0.2 mmol, 1.0 equiv), Pd(TFA)_2_ (10 μmol, 5 mol%), (*S*)-**F** (15 μmol, 7.5 mol%), BnNMe_2_ (1.0 mmol, 5 equiv), TFA (0.4 mmol, 2 equiv), DMA (4.0 mL), temperature (150°C), time (24 hr) in a sealed tube. Isolated yield was obtained, and the ee values were determined by high-performance liquid chromatography analysis. Absolute configurations of products **2** were determined by comparing structure of (*S*)-**2a** (absolute configuration of (*S*)-**2a** was assigned by X-ray diffraction analysis). Bn, benzyl. See Transparent Methods for experimental details.

After obtaining the optimized conditions, the substrate scope for the palladium-catalyzed intramolecular enantioselective hydroarylation of **1** was surveyed using (*S*)-**F** as the ligand. As shown in Figure 4, we first attempted variation of substituents R^1^ in **1**; various alkyl groups including methyl, ethyl, propyl, isopropyl, cyclopropyl, cyclopentyl, phenethyl, and phenpropyl were feasible, and the reaction provided high reactivity (76%–83% yields) and excellent enantioselectivity (97%–99% ee) (see **2a-h**). When substituents R^1^ in **1** were different substituted benzyls, their enantioselectivity was also excellent (98%–99% ee) (see **2i-m**). Subsequently, variation of substituents R^2^ in **1** was investigated (see **2n-ad**). For substituents R^2^ with different substituted phenyls, the influence of electronic effect including electron-donating (see **2n-t**), slight electron-withdrawing (see **2u-w**), and strong electron-withdrawing groups (see **2x-z**) on the phenyl rings was slight, and high reactivity (74%–84% yields) and excellent enantioselectivity (97%–99% ee) of the substrates were observed. When substituents R^2^ were benzyl (see **2aa** and **2ab**) and cyclohexyl (see **2ac** and **2ad**), the reaction also afforded high yields and excellent ee values. Variation of substituents R^3^ on the phenyl rings was investigated, and excellent results were obtained (see **2ae-ah**).

Next, influence of the cyclic diphosphine ligands, (*S*)-**E**, (*S*)-**F,** and (*S*)-**G**, with different dihedral angles was investigated (Figure 4), and we found that the different substrates exhibited slight difference in reactivity and enantioselectivity with variation of the ligands. For all the tested substrates, (*S*)-**F** containing nine-membered ring was a suitable ligand. For synthesis of **2b** and **2y**, (*S*)-**G** containing ten-membered ring showed slightly higher enantioselectivity than (*S*)-**E**, which contained an eight-membered ring and (*S*)-**F**. The present reaction showed tolerance of various functional groups including C-F, C-Cl, and C-Br bonds and ether, CF_3_, nitro, cyano, ester, and amide groups. It is worthwhile to note that substrates **1** have unactivated 2-iodobenzy unit. In fact, it was usually difficult for the reaction of the substrates with this unit in previous report, and an effective solution was the use of substituted 2-halobenzoyls with high reactivity as the alternatives of 2-iodobenzy unit (Shen et al., 2015). In addition, no erosion of ee values was observed at such high temperature (150°C). The results showed that our catalyst system was highly efficient in the present reaction.

### Applications of the Method

**Figure 5:**
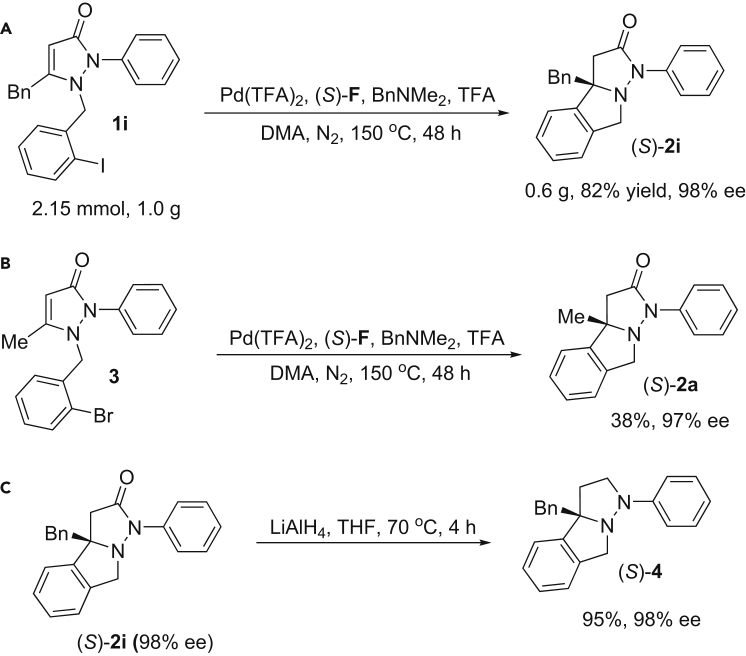
Applications of the Method (A) Scale synthesis of (*S*)-**2i**. (B) Palladium-catalyzed asymmetric cyclization of 1-(2-bromobenzyl)-5-methyl-2-phenyl-1*H*-pyrazol-3(2*H*)-one (**3**). (C) Reduction of (*S*)-**2i**.

A scale synthesis of (*S*)-**2i** was performed as example. As shown in Figure 5A, reaction of **1i** (2.15 mmol, 1.0 g) under standard conditions provided (*S*)-**2i** in 82% yield with 98% ee without loss of yield and enantioselectivity. We attempted the reaction of aryl bromide **3** under the conditions (Figure 5B), and (*S*)-**2a** was obtained in 38% yield with 97% ee. Furthermore, reduction of (*S*)-**2i** with LiAlH_4_ provided (*S*)-**4** in 95% yield with 98% ee without loss of ee (Figure 5C).

### Mechanism of the Reaction

**Figure 6:**
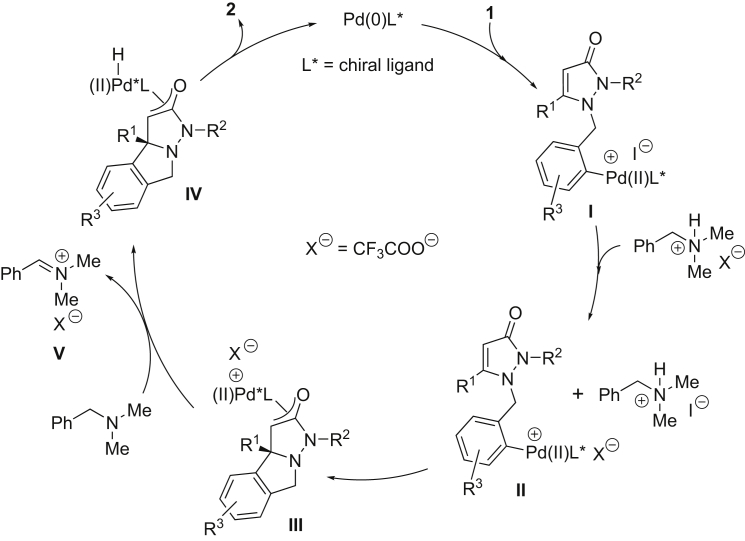
Possible Mechanism for the Palladium-Catalyzed Intramolecular Asymmetric Hydroarylation

According to the experiments mentioned above and previous references (Raoufmoghaddam et al., 2015, Minatti et al., 2007), a reaction pathway of this palladium-catalyzed intramolecular enantioselective hydroarylation is proposed in Figure 6. Oxidative addition of the aryl iodide **1** to the *in situ*-formed Pd(0) diphosphine complex leads to the Pd(II) intermediate **I**, and then anion exchange of **I** with the salt (BnNHMe_2_^+-^O_2_CCF_3_) provides **II**. Carbopalladation of the double bond in **II** yields the *π*-oxa-allyl palladium species **III**. A hydride transfer from the CH_2_ of benzyl in BnNMe_2_ to palladium gives the Pd(II) hydride complex **IV** leaving the iminium ion **V**. Reductive elimination of the Pd(II) hydride complex **IV** finally affords the target product (**2**) with regeneration of Pd(0)L*.

### Extension of the Method

**Figure 7:**
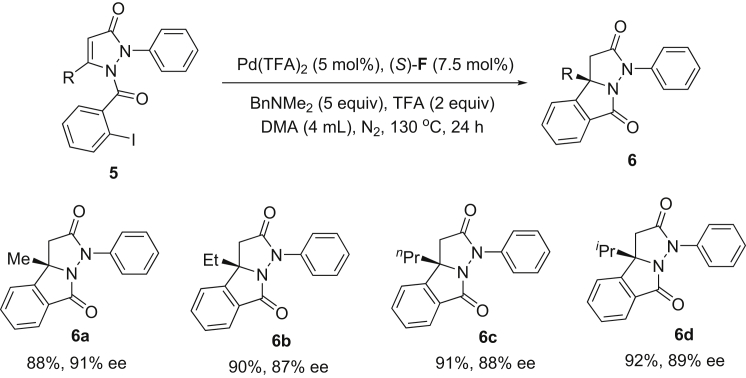
Palladium-Catalyzed Intramolecular Asymmetric Hydroarylation of *o*-Iodobenzoyl Derivatives (5)

Furthermore, the palladium-catalyzed intramolecular asymmetric hydroarylation of *o*-iodobenzoyl derivatives (**5**) was attempted under conditions similar to those in (Figures 4 and 7), and we found that *o*-iodobenzoyl derivatives (**5**) exhibited higher reactivity and lower enantioselectivity than *o*-iodobenzy derivatives (**1**). Unfortunately, the factors that lead to lower enantioselectivity of **6** than **2** are unknown for us.

### Limitations of Study

It should be pointed out that there are limitations to the present method including requirement of higher temperature and maladjustment of other common ligands.

### Conclusions

In summary, we have developed an efficient and highly enantioselective palladium-catalyzed intramolecular hydroarylation, in which the reactivity and enantioselectivity of the substrates were tuned by our newly developed axially chiral cyclic diphosphine ligands and the new kind of *N*-heterocycles, 1*H*-pyrazolo[5,1-*a*]isoindol-2(8*H*)-ones containing a quaternary stereocenter, were prepared in high yields and excellent ee values with numerous functional group tolerance. We believe that our axially chiral cyclic diphosphine ligands with the adjustable dihedral angles will find wide application in asymmetric synthesis.

## Methods

All methods can be found in the accompanying Transparent Methods supplemental file.

## Data and Software Availability

Crystallographic data have been deposited in the Cambridge Crystallographic Data Center under accession numbers CCDC: 1842685, 1822026, 1842686, and 1822025.

## Supplemental Information

Document S1. Transparent Methods, Figures S1–S312, and Tables S1–S4

Data S1. Crystal Data and Structure Refinement for (*S*)-**E**, Related to Figure 3

Data S2. Crystal Data and Structure Refinement for (*S*)-**F**, Related to Figure 3

Data S3. Crystal Data and Structure Refinement for (*S*)-**G**, Related to Figure 3

Data S4. Crystal Data and Structure Refinement for (*S*)-**2a**, Related to Table 1
