# Supplementary material for: Axially Chiral Cyclic Diphosphine Ligand-Enabled Palladium-Catalyzed Intramolecular Asymmetric Hydroarylation
Source: iScience. 2018 Nov 14;10:11–22. doi: 10.1016/j.isci.2018.11.018 (PMC6260458; doi:10.1016/j.isci.2018.11.018)
Supplement: Document S1. Transparent Methods, Figures S1–S312, and Tables S1–S4 [file mmc1.pdf]

ISCI, Volume *10*

Supplemental Information

Axially Chiral Cyclic Diphosphine Ligand-Enabled Palladium-Catalyzed Intramolecular Asymmetric Hydroarylation

Can Liu, Xianjin Zhu, Pengxiang Zhang, Haijun Yang, Changjin Zhu, and Hua Fu

Supplemental Information

Axially Chiral Cyclic Diphosphine Ligand-Enabled Palladium-Catalyzed Intramolecular Asymmetric Hydroarylation

Can Liu,[1,2] Xianjin Zhu,[2] Pengxiang Zhang,[2] Haijun Yang,[2] Changjin Zhu,[1] and Hua Fu[1,2*]

[1]School of Chemistry and Chemical Engineering, Beijing Institute of Technology, Beijing 100081, China

[2]Key Laboratory of Bioorganic Phosphorus Chemistry and Chemical Biology (Ministry of Education), Department of Chemistry, Tsinghua University, Beijing 100084, China

*To whom correspondence should be addressed. E-mail: fuhua@mail.tsinghua.edu.cn

Transparent Methods

1. General Procedures

All reactions were carried out under a nitrogen atmosphere in dry solvents. The reactions were monitored by thin layer chromatography (TLC), and the products were isolated by silica gel column chromatography. Melting points were recorded on a Beijing Tech X-4 melting point apparatus. High-resolution mass spectra (HRMS) were recorded on LCMS-IT/TOF (SHIMADZU, Japan) with an electrospray ionization source. ^{1}H, ^{13}C, ^{19}F and ^{31}P NMR spectra were recorded on JNM-ECA 300, JEOL ECS-400 or JNM-ECA 600 spectrometers. Chemical shifts were reported in ppm down field from internal Me_4Si, external $CFCl_3$ and external H_3PO_4, respectively. The following abbreviations (or combinations thereof) were used to explain the multiplicities: s = singlet, d = doublet, t = triplet, q = quartet, quint = quintet, sext = sextet, h = heptet, m = multiplet, dd = doublet of doublets, dt = doublet of triplets, td = triplet of doublets, br = broad. Chiral HPLC analysis was achieved using an Agilent 1100 Infinity series normal phase HPLC unit and Agilent Chemstation software. Daicel Chiralpak columns (250 × 4.6 mm) were used as specified in the text. Solvents were used of HPLC grade (Sigma Aldrich); all eluent systems were isocratic. Optical rotations were recorded using a WZZ-2S Polarimeter. Single crystal X-ray data were collected on a Bruker APEXII X-ray diffractometer equipped with a CMOS PHOTON 100 detector with a Cu Kα X-ray source (Kα = 1.54178 Å). Data were indexed, integrated and scaled using DENZO and SCALEPACK from the HKL program suite (Otwinowski and Minor, 1997). Structures of (*S*)-**2a**, (*S*)-**C**, (*S*)-**D** and (*S*)-**E** were solved through direct method (SHELXS-97) and refined by full-matrix least-squares (SHELXL-2014) on F^2. Anisotropic thermal parameters were used for the non-hydrogen atoms and isotropic parameters for the hydrogen atoms. The data obtained were deposited at the Cambridge Crystallographic Data Centre.

2. Synthesis and Characterizaion Data of Ligands (*R*)-E, (*S*)-E, (*R*)-F, (*S*)-F, (*R*)-G and (*S*)-G

Synthesis of diphosphine ligands were performed according to the previous procedures (Xie et al., 2003).

(1) Synthesis of compounds *Rac*-M-1

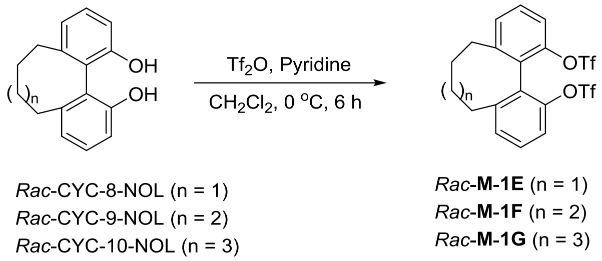

(a) Synthesis of *Rac*-5,6,7,8-tetrahydrodibenzo[*a,c*][8]annulene-1,12-diyl bis(trifluoromethanesulfonate) (*Rac*-M-1E)

Typical procedure: To a solution of *Rac*-CYC-8-NOL (*see the reference for their synthesis*) (Zhang et al., 2016) (3.0 g, 12.5 mmol) in 60 mL of CH_2Cl_2 was added pyridine (4.0 mL, 50 mmol), and followed by dropwise addition of triflic anhydride (5.2 mL, 27.7 mmol) at 0 ℃. The mixture was stirred at room temperature for 6 h. After removal of the solvent, the residue was diluted with EtOAc (60 mL) and then washed with 5% aqueous HCl, saturated $NaHCO_3$, and brine (once for each). The organic layer was dried over anhydrous sodium sulfate, concentrated under reduced pressure, and passed through a silica gel plug (eluted with CH_2Cl_2) to give *Rac*-**M-1E** (6.0 g, 95%) as a white solid, mp = 72-73 ℃. ^{1}H NMR (400 MHz, $CDCl_3$) δ 7.48 (t, *J* = 8.0 Hz, 2H), 7.37 (d, *J* = 7.1 Hz, 2H), 7.23 (d, *J* = 7.6 Hz, 2H), 2.83 (dd, *J* = 13.7, 8.4 Hz, 2H), 2.25-2.15 (m, 2H), 2.15-2.02 (m, 2H), 1.53-1.39 (m, 2H); ^{13}C NMR (100 MHz, $CDCl_3$) δ 147.83, 146.99, 130.92, 129.78, 127.06, 118.97, 118.45 (q, *J* = 320.2 Hz), 32.55, 29.30; ^{19}F NMR (565 MHz, $CDCl_3$) δ −74.99; MS (EI): Calcd for $C_{18}H_{14}F_6O_6S_2$, M$^+$ *m/z* 504. Found M$^+$ *m/z* 504.

(b) Synthesis of *Rac*-6,7,8,9-tetrahydro-5*H*-dibenzo[*a,c*][9]annulene-1,13-diyl bis(trifluoromethanesulfonate) (*Rac*-M-1F)

Rac-**M-1F** was synthesized by the same procedure as that for *Rac*-**M-1E** as white solid. Yield 94%. mp = 73-74 ℃. ^{1}H NMR (400 MHz, $CDCl_3$) δ 7.48 (t, *J* = 8.0 Hz, 2H), 7.37 (d, *J* = 7.7 Hz, 2H), 7.25 (d, *J* = 8.1 Hz, 2H), 2.81 (ddd, *J* = 14.4, 7.0, 3.5 Hz, 2H), 2.08 (ddd, *J* = 14.2, 10.9, 3.1 Hz, 2H), 1.93-1.79 (m, 2H), 1.61-1.50 (m, 2H), 1.47-1.35 (m, 2H); ^{13}C NMR (75 MHz, $CDCl_3$) δ

147.10, 146.89, 130.50, 129.55, 128.23, 118.86, 118.41 (q, J = 319.5 Hz), 33.50, 28.81, 28.63; ^{19}F NMR (565 MHz, $CDCl_3$) δ −75.17; MS (EI): Calcd for $C_{19}H_{16}F_6O_6S_2$, M^+ m/z 518. Found M^+ m/z 518.

(c) Synthesis of *Rac*-5,6,7,8,9,10-hexahydrodibenzo[*a,c*][10]annulene-1,14-diyl bis(trifluoromethanesulfonate) (*Rac*-M-1G)

Rac-**M-1G** was synthesized by the same procedure as that for *Rac*-**M-1E** as a white solid. Yield 94%. mp = 74-75 ℃. ^{1}H NMR (400 MHz, $CDCl_3$) δ 7.47 (t, J = 8.0 Hz, 2H), 7.39 (d, J = 7.7 Hz, 2H), 7.26 (d, J = 8.1 Hz, 2H), 2.67 (dt, J = 14.1, 3.9 Hz, 2H), 2.41 (td, J = 13.6, 4.2 Hz, 2H), 1.85-1.70 (m, 2H), 1.56-1.46 (m, 2H), 1.40-1.27 (m, 3H), 0.93-0.66 (m, 3H); ^{13}C NMR (100 MHz, $CDCl_3$) δ 147.54, 145.49, 130.45, 128.79, 128.46, 118.42, 118.33 (q, J = 319.5 Hz), 28.96 , 28.80 , 21.02; ^{19}F NMR (565 MHz, $CDCl_3$) δ −75.54; MS (EI): Calcd for $C_{19}H_{16}F_6O_6S_2$, M^+ m/z 518. Found M^+ m/z 518.

(2) Synthesis of compounds *Rac*-M-2

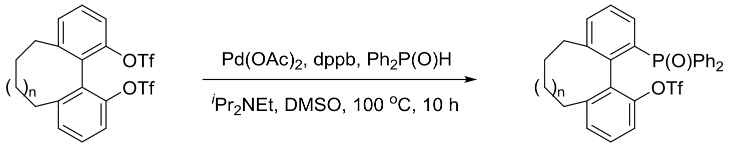

Rac-**M-1E** (n = 1)
Rac-**M-1F** (n = 2)
Rac-**M-1G** (n = 3)

Rac-**M-2E** (n = 1)
Rac-**M-2F** (n = 2)
Rac-**M-2G** (n = 3)

(a) Synthesis of *Rac*-12-(diphenylphosphoryl)-5,6,7,8-tetrahydrodibenzo[*a,c*][8]-annulen-1-yl trifluoromethanesulfonate (*Rac*-M-2E)

Typical procedure: To a mixture of *Rac*-**M-1E** (5.0 g, 9.92 mmol), diphenylphosphine oxide (4.0 g, 19.84 mmol), palladium acetate (112 mg, 0.5 mmol) and 1,4-bis(diphenylphospino)butane (dppb, 213 mg, 0.5 mmol) was added 30 mL of degassed DMSO and diisopropylethylamine (6.56 mL, 5.13 g, 39.7 mmol), and the mixture was heated with stirring at 100 ℃ for 10 h. After cooling to room temperature, the reaction mixture was diluted with EtOAc, washed twice with water, dried over anhydrous $MgSO_4$ and concentrated under reduced pressure. The residue was chromatographed on a silica gel column eluted with petroleum ether/EtOAc (4:1 in volume) to give (*S*)-12-(diphenylphosphoryl)-5,6,7,8-tetrahydrodibenzo[*a,c*][8] annulen-1-yl trifluoromethanesulfonate *Rac*-**M-2E** (4.9 g, 89%) as a white solid, mp = 203-205 ℃. ^{1}H NMR (400 MHz, $CDCl_3$) δ 7.72 (dd, J_1 = 11.7, J_2 = 7.4 Hz, 2H), 7.53-7.33 (m, 8H), 7.32-7.17 (m, 4H), 7.03 (d, *J* = 8.0 Hz, 2H), 2.69 (dd, J_1 = 13.6, J_2 = 7.9 Hz, 1H), 2.37 (dd, J_1 = 13.2, J_2 =7.5 Hz, 1H), 2.27-2.15 (m, 1H), 2.09-1.96 (m, 1H), 1.96-1.78 (m, 2H), 1.38-1.26 (m, 2H); ^{13}C NMR (100 MHz, $CDCl_3$) δ 147.77, 146.69, 146.60, 146.21, 136.85, 136.78, 134.22, 133.14, 132.24, 132.15, 132.11, 131.77, 131.69, 131.59, 131.56, 131.49, 131.26, 131.24, 131.19, 131.15, 130.77, 130.01, 128.58, 128.44, 128.36, 128.24, 128.17, 128.05, 118.31 (q, *J* = 320.2 Hz), 118.23, , 32.36, 32.05, 29.73, 29.67; ^{19}F NMR (283 MHz, $CDCl_3$) δ −74.49; ^{31}P NMR (122 MHz, $CDCl_3$) δ 28.09; HRMS (ESI$^+$): Calcd for $C_{29}H_{25}F_3O_4PS$, $[M+H]^+$ *m/z* 557.1163. Found 557.1169.

(b) Synthesis of *Rac*-13-(diphenylphosphoryl)-6,7,8,9-tetrahydro-5*H*-dibenzo[*a,c*][9]-annulen-1-yl trifluoromethanesulfonate (*Rac*-M-2F)

Rac-**M-2F** was synthesized by the same procedure as that for *Rac*-**M-2E** as white solid, mp = 206-207 ℃, yield 88%. ^{1}H NMR (400 MHz, $CDCl_3$) δ 7.63-7.52 (m, 4H), 7.43 (t, *J* = 7.7 Hz, 3H), 7.39-7.30 (m, 5H), 7.28-7.20 (m, 2H), 7.09 (d, *J* = 7.7 Hz, 1H), 6.90 (d, *J* = 8.2 Hz, 1H), 2.68 (dt, J_1 = 13.8, J_2 = 4.5 Hz, 1H), 2.50-2.38 (m, 1H), 2.12-1.98 (m, 1H), 1.97-1.85 (m, 1H), 1.79-1.59 (m, 2H), 1.56-1.40 (m, 1H), 1.39-1.23 (m, 2H), 1.19-1.04 (m, 1H); ^{13}C NMR (100 MHz, $CDCl_3$) δ 147.29, 146.17, 144.77, 144.68, 138.03, 137.95, 133.51, 132.94, 132.86, 132.85, 132.48, 131.96, 131.90, 131.87, 131.80, 131.70, 131.42, 131.37, 131.34, 131.31, 130.84, 129.36, 128.54, 128.20, 128.08, 128.03, 127.97, 127.90, 118.10 (q, *J* = 319.8 Hz), 117.64, 34.15, 32.30, 29.65, 28.92, 27.77; ^{19}F NMR (565 MHz, $CDCl_3$) δ −75.64; ^{31}P NMR (243 MHz, $CDCl_3$) δ 27.27; HRMS (ESI$^+$): Calcd for $C_{30}H_{27}F_3O_4PS$, $[M+H]^+$ *m/z* 571.1320. Found 571.1317.

(c) Synthesis of *Rac*-14-(diphenylphosphoryl)-5,6,7,8,9,10-hexahydrodibenzo[*a*,*c*][10]-annulen-1-yl trifluoromethanesulfonate (*Rac*-M-2G)

Rac-**M-2G** was synthesized by the same procedure as that for *Rac*-**M-2E** as a white solid. Yield 90%. mp = 204-205 ℃. ^{1}H NMR (400 MHz, $CDCl_3$) δ 7.79-7.70 (m, 2H), 7.54-7.33 (m, 8H), 7.29-7.17 (m, 5H), 6.61 (d, *J* = 7.8 Hz, 1H), 2.60 (t, *J* = 13.6 Hz, 2H), 2.46-2.32 (m, 2H), 1.70 (q, *J* = 12.7 Hz, 2H), 1.49-1.38 (m, 2H), 1.31-1.20 (m, 2H), 0.73-0.55 (m, 2H); ^{13}C NMR (75 MHz, $CDCl_3$) δ 147.50, 146.44, 143.68, 143.56, 138.82, 138.72, 133.50, 132.93, 132.90, 132.24, 132.12, 131.84, 131.67, 131.58, 131.55, 131.52, 131.15, 131.10, 130.97, 129.56, 128.42, 128.27, 128.06, 127.90, 127.71, 118.07 (q, *J* = 319.5 Hz), 116.55, 29.95, 29.08, 28.62, 28.54, 21.15, 20.55; ^{19}F NMR (283 MHz, $CDCl_3$) δ −75.05; ^{31}P NMR (122 MHz, $CDCl_3$) δ 27.11; HRMS (ESI$^+$): Calcd for $C_{31}H_{29}F_3O_4PS$, $[M+H]^+$ *m/z* 585.1476. Found 585.1516.

(3) Synthesis of compounds *Rac*-M-3 (Wu et al., 2004)

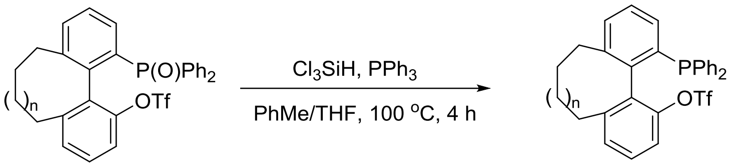

Rac-**M-2E** (n = 1)
Rac-**M-2F** (n = 2)
Rac-**M-2G** (n = 3)

Rac-**M-3E** (n = 1)
Rac-**M-3D** (n = 2)
Rac-**M-3G** (n = 3)

(a) Synthesis of *Rac*-12-(diphenylphosphanyl)-5,6,7,8-tetrahydrodibenzo[*a*,*c*][8]-annulen-1-yl trifluoromethanesulfonate (*Rac*-M-3E)

Typical procedure: In a 250 mL pressure tube *Rac*-**M-2E** **(**2.78 g, 5.0 mmol) and triphenylphosphine (2.62 g, 10.0 mmol) were dissolved in 100 mL of mixed solvent of degassed THF and toluene (1:1) under nitrogen atmosphere. To the solution was added trichlorosilane (10.1 mL, 100.0 mmol) at room temperature, and the mixture was stirred at 100 ℃ for 4 h. After cooling to ambient temperature, the mixture was diluted with diethyl ether. To the solution was added ice (250 g) and 20% NaOH solution (250 mL). The mixture was transferred to a separating funnel and shaked for 10 min. The organic layer was separated and washed successively with saturated $NaHCO_3$, brine and water. The solution was then dried over anhydrous Na_2SO_4 and the solvent removed under reduced pressure. The residue was purified by flash chromatography on silica gel (petroleum ether/EtOAc 50:1) to afford *Rac*-**M-3E** (2.2 g, 82%) as a white solid, mp = 76-78 ℃. ^{1}H NMR (400 MHz, $CDCl_3$) δ 7.37-7.23 (m, 8H), 7.23-7.18 (m, 2H), 7.18-7.12 (m, 2H), 7.05 (d, *J* = 7.7 Hz, 1H), 7.02-6.94 (m, 3H), 2.68 (dd, *J* = 13.5, 7.8 Hz, 1H), 2.23-2.06 (m, 2H), 2.02-1.93 (m, 1H), 1.92-1.81 (m, 1H), 1.59 (t, *J* = 12.3 Hz, 1H), 1.41-1.19 (m, 2H); ^{13}C NMR (100 MHz, $CDCl_3$) δ 147.38, 146.48, 145.13, 145.08, 138.08, 137.95, 137.79, 137.49, 137.19, 137.07, 136.10, 135.99, 134.62, 134.40, 133.33, 133.16, 132.48, 132.40, 130.84, 130.27, 129.79, 129.77, 129.35, 129.01, 128.85, 128.64, 128.52, 128.49, 128.25, 128.16, 120.10, 118.81, 116.91, 32.42, 31.92, 29.87, 29.72; ^{19}F NMR (565 MHz, $CDCl_3$) δ −75.18; ^{31}P NMR (243 MHz, $CDCl_3$) δ −9.79; HRMS (ESI$^+$): Calcd for $C_{29}H_{25}F_3O_3PS$, $[M+H]^+$ *m*/*z* 541.1214. Found 544.1207.

(b) Synthesis of *Rac*-13-(diphenylphosphanyl)-6,7,8,9-tetrahydro-5*H*-dibenzo[*a*,*c*][9]-annulen-1-yl trifluoromethanesulfonate (*Rac*-M-3F)

Rac-**M-3F** was synthesized by the same procedure as that for *Rac*-**M-3E** as a white solid. Yield 84%. mp = 96-97 ℃. ^{1}H NMR (400 MHz, $CDCl_3$) δ 7.37 (t, *J* = 8.0 Hz, 1H), 7.33-7.18 (m, 11H), 7.15-7.08 (m, 3H), 7.08-7.02 (m, 1H), 2.80-2.66 (m, 1H), 2.09-1.90 (m, 2H), 1.81-1.66 (m, 1H), 1.64-1.17 (m, 6H); ^{13}C NMR (100 MHz, $CDCl_3$) δ 146.78, 146.49, 143.52, 143.46, 139.67, 139.34, 137.88, 137.77, 137.31, 137.19, 135.95, 135.84, 134.84, 134.63, 133.84, 133.76, 133.27, 133.08, 131.41, 130.27, 129.38, 128.96, 128.91, 128.45, 128.39, 128.32, 128.21, 123.18, 119.92, 118.61, 116.81, 113.63, 33.48, 32.85, 29.47, 29.10, 28.24; ^{19}F NMR (565 MHz, $CDCl_3$) δ −75.44; ^{31}P NMR (243 MHz, $CDCl_3$) δ −12.45; HRMS (ESI$^+$): Calcd for $C_{30}H_{27}F_3O_3PS$, $[M+H]^+$ *m*/*z* 555.1371. Found 555.1357.

(c) Synthesis of *Rac*-14-(diphenylphosphanyl)-5,6,7,8,9,10-hexahydrodibenzo[*a*,*c*][10]-annulen-1-yl trifluoromethanesulfonate (*Rac*-M-3G)

Rac-**M-3G** was synthesized by the same procedure as that for *Rac*-**M-3E** as a white solid. Yield 86%. mp = 115-116 ℃. ^{1}H NMR (400 MHz, $CDCl_3$) δ 7.39-7.18 (m, 12H), 7.12-7.03 (m, 3H), 7.01 (ddd, *J* = 7.2, 3.4, 1.5 Hz, 1H), 2.61 (dt, *J* = 13.9, 3.4 Hz, 1H), 2.36 (td, *J* = 13.6, 4.0 Hz, 1H), 2.19 (td, *J* = 13.7, 3.9 Hz, 1H), 2.04-1.91 (m, 1H), 1.81-1.57 (m, 2H), 1.53-1.40 (m, 1H), 1.37-1.20 (m, 3H), 0.81-0.58 (m, 2H); ^{13}C NMR (100 MHz, $CDCl_3$) δ 147.64, 145.30, 141.85, 141.80, 140.59, 140.28, 138.31, 138.21, 136.82, 136.71, 136.55, 136.43, 134.50, 134.36, 134.28, 133.75, 133.56, 131.82, 129.73, 129.38, 128.80, 128.43, 128.21, 119.80, 117.87, 116.62, 29.00, 28.83, 28.48, 21.08, 20.96; ^{19}F NMR (565 MHz, $CDCl_3$) δ −75.69; ^{31}P NMR (243 MHz, $CDCl_3$) δ −13.29; HRMS (ESI$^+$): Calcd for $C_{31}H_{29}F_3O_3PS$, $[M+H]^+$ *m*/*z* 569.1527. Found 569.1522.

(4) Synthesis of compounds *Rac*-M-4

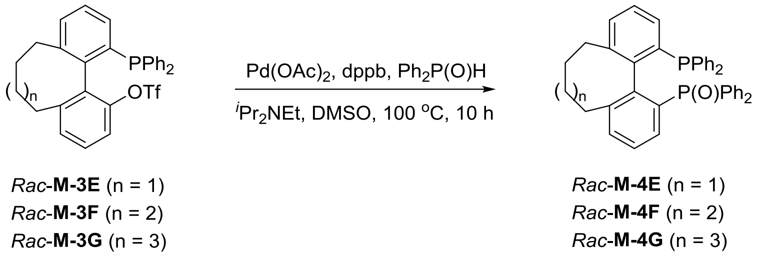

(a) Synthesis of *Rac*-(12-(diphenylphosphanyl)-5,6,7,8-tetrahydrodibenzo[*a*,*c*][8]-annulen-1-yl) diphenylphosphine oxide (*Rac*-M-4E)

Typical procedure: To a mixture of *Rac*-**M-3E** (1.35 g, 2.5 mmol), diphenylphosphine oxide (1.0 g, 4.95 mmol), palladium acetate (28 mg, 0.125 mmol) and 1,4-bis(diphenylphospino)butane (dppb, 53 mg, 0.125 mmol) was added 15 mL of degassed DMSO and diisopropylethylamine (1.64 mL, 1.28 g, 9.93 mmol), and the mixture was heated with stirring at 100 ℃ for 10 h. After cooling to room temperature, the reaction mixture was diluted with EtOAc, washed twice with water, dried over anhydrous $MgSO_4$ and concentrated under reduced pressure. The residue was chromatographed on a silica gel column eluted with petroleum ether/EtOAc (4:1 in volume) to give *Rac*-**M-4E** (2.15 g, 86%) as a white solid, mp = 248-249 ℃. ^{1}H NMR (600 MHz, $CDCl_3$) δ 8.01-7.87 (m, 2H), 7.54 (t, J = 6.9 Hz, 2H), 7.49 (t, J = 7.3 Hz, 1H), 7.47-7.41 (m, 2H), 7.37-7.25 (m, 8H), 7.22-7.15 (m, 3H), 7.16-7.11 (m, 3H), 7.05 (t, J = 7.5 Hz, 1H), 7.02-6.96 (m, 3H), 6.59 (d, J = 7.5 Hz, 1H), 1.91 (dd, J = 13.2, 7.5 Hz, 1H), 1.73-1.62 (m, 4H), 1.17 (t, J = 11.6 Hz, 1H), 1.12-1.01 (m, 2H); ^{13}C NMR (150 MHz, $CDCl_3$) δ 146.06, 145.98, 145.92, 143.22, 143.19, 142.86, 142.80, 142.75, 141.20, 141.12, 141.03, 141.00, 140.82, 139.38, 139.27, 136.92, 136.82, 135.99, 135.83, 134.40, 133.83, 133.71, 133.42, 133.31, 133.15, 132.61, 132.56, 131.74, 131.65, 131.30, 130.95, 130.89, 130.52, 129.48, 128.64, 128.59, 128.53, 128.29, 128.21, 128.15, 128.12, 127.96, 127.91, 127.87, 127.79, 127.70, 127.61, 127.30, 33.04, 30.88, 30.08, 29.93; ^{31}P NMR (243 MHz, $CDCl_3$) δ 25.30, −11.16; HRMS (ESI$^+$): Calcd for $C_{40}H_{35}OP_2$, $[M+H]^+$ *m/z* 593.2163. Found 593.2169.

(b) Synthesis of *Rac*-(13-(diphenylphosphanyl)-6,7,8,9-tetrahydro-5*H*-dibenzo[*a*,*c*][9]-annulen-1-yl) diphenylphosphine oxide (*Rac*-M-4F)

Rac-**M-4F** was synthesized by the same procedure as that for *Rac*-**M-4E** as a white solid. Yield 85%. mp = 227-229 ℃. ^{1}H NMR (400 MHz, $CDCl_3$) δ 7.83 (dd, *J* = 11.3, 7.4 Hz, 2H), 7.49 (dd, *J* = 11.4, 7.6 Hz, 2H), 7.44-7.29 (m, 6H), 7.27-7.06 (m, 15H), 6.79 (d, *J* = 7.0 Hz, 1H), 1.95-1.76 (m, 1H), 1.55-1.26 (m, 4H), 1.24-0.98 (m, 4H), 0.97-0.80 (m, 1H); ^{13}C NMR (100 MHz, $CDCl_3$) δ 144.50, 144.41, 144.31, 144.23, 144.15, 142.91, 142.87, 142.59, 142.55, 141.62, 141.57, 139.96, 139.86, 139.72, 139.57, 136.29, 136.14, 135.78, 135.55, 134.62, 133.59, 133.43, 132.94, 132.77, 132.42, 132.24, 132.15, 132.12, 131.67, 131.58, 131.32, 131.20, 131.08, 130.74, 130.36, 130.06, 129.08, 128.65, 128.10, 127.97, 127.95, 127.87, 127.83, 127.71, 126.96, 126.79, 33.91, 30.99, 29.50, 29.26, 27.85; ^{31}P NMR (243 MHz, $CDCl_3$) δ 26.51, −12.45; HRMS (ESI^+): Calcd for $C_{41}H_{37}OP_2$, $[M+H]^+$ *m*/*z* 607.2320. Found 607.2309.

(c) Synthesis of *Rac*-(14-(diphenylphosphanyl)-5,6,7,8,9,10-hexahydrodibenzo[*a*,*c*][10]-annulen-1-yl)diphenylphosphine oxide (*Rac*-M-4G)

Rac-**M-4G** was synthesized by the same procedure as that for *Rac*-**M-4E** as a white solid. Yield 87%. mp = 259-260 ℃. ^{1}H NMR (400 MHz, $CDCl_3$) δ 7.60 (q, *J* = 10.1 Hz, 4H), 7.45-7.14 (m, 21H), 7.10-7.03 (m, 2H), 1.96-1.71 (m, 2H), 1.59-1.39 (m, 3H), 1.21-1.01 (m, 5H), 0.71-0.36 (m, 2H); ^{13}C NMR (100 MHz, $CDCl_3$) δ 145.93, 145.85, 145.77, 144.97, 144.94, 144.65, 144.62, 143.96, 143.93, 143.86, 143.83, 140.10, 140.05, 139.92, 139.37, 139.28, 136.67, 136.54, 135.61, 135.38, 135.30, 134.26, 133.77, 133.01, 132.84, 132.76, 132.25, 132.16, 131.92, 131.82, 131.68, 131.62, 131.21, 131.20, 130.26, 128.81, 128.24, 128.21, 128.13, 128.04, 127.96, 127.91, 127.10, 126.79, 126.65, 28.96, 28.66, 28.41, 27.52, 21.07, 20.80; ^{31}P NMR (122 MHz, $CDCl_3$) δ 29.53, −14.53; HRMS (ESI^+): Calcd for $C_{42}H_{39}OP_2$, $[M+H]^+$ *m*/*z* 621.2676. Found 621.2470.

(5) Seperation of *Rac*-M-4 to provide (*R*)-M-4 and (*S*)-M-4

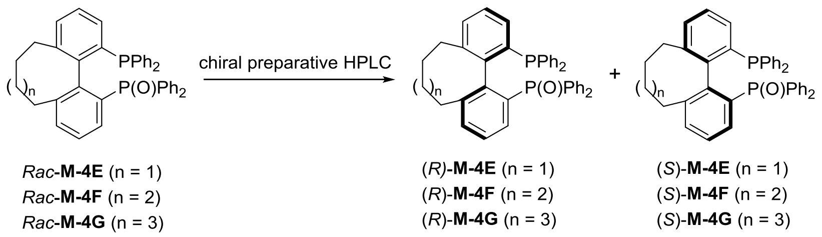

Rac-**M-4** were separated to afford the (*R*)-**M-4** and (*S*)-**M-4** with the help of Daicel Chiral Technologies (China) Co., Ltd..

HPLC analysis for (*R*)-**M-4E**: Daicel Chiralpak IF; hexane/EtOH: 90:10; flow: 1.0 mL/min; λ = 220 nm. 25 °C. 99% ee (t_R (major) = 9.7 min, t_R (minor) = 12.4 min).

HPLC analysis for (*S*)-**M-4E**: Daicel Chiralpak IF; hexane/EtOH: 90:10; flow: 1.0 mL/min; λ = 220 nm. 25 °C. 99% ee (t_R (minor) = 9.8 min, t_R (major) = 12.4 min).

HPLC analysis for (*R*)-**M-4F**: Daicel Chiralpak IF; hexane/EtOH: 90:10; flow: 1.0 mL/min; λ = 220 nm. 25 °C. 99% ee (t_R (major) = 9.4 min, t_R (minor) = 12.2 min).

HPLC analysis for (*S*)-**M-4F**: Daicel Chiralpak IF; hexane/EtOH: 90:10; flow: 1.0 mL/min; λ = 220 nm. 25 °C. 99% ee (t_R (minor) = 9.3 min, t_R (major) = 12.2 min).

HPLC analysis for (*R*)-**M-4G**: Daicel Chiralpak IF; hexane/EtOH: 90:10; flow: 1.0 mL/min; λ = 220 nm. 25 °C. 98% ee (t_R (major) = 8.2 min, t_R (minor) = 11.2 min).

HPLC analysis for (*S*)-**M-4G**: Daicel Chiralpak IF; hexane/EtOH: 90:10; flow: 1.0 mL/min; λ = 220 nm. 25 °C. 98% ee (t_R (minor) = 8.3 min, t_R (major) = 11.3 min).

(6) Synthesis of ligands (*R*)-E, (*S*)-E, (*R*)-F, (*S*)-F, (*R*)-G and (*S*)-G

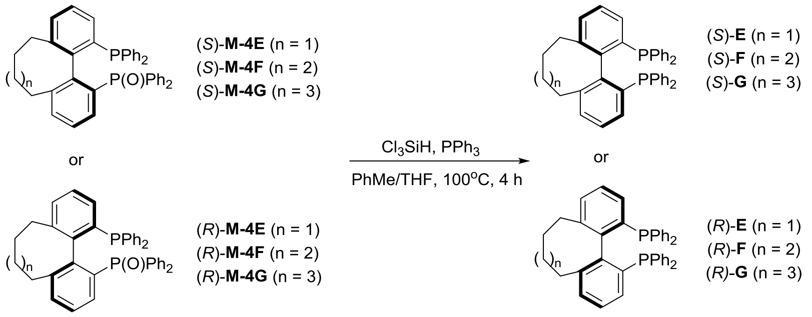

(a) Synthesis of (*S*)-1,12-bis(diphenylphosphanyl)-5,6,7,8-tetrahydrodibenzo[*a*,*c*][8]-annulene ((*S*)-E)

Typical procedure: In a 250 mL pressure tube, (*S*)-**M-4E** (0.592 g, 1.0 mmol) and triphenylphosphine (0.52 g, 2.0 mmol) were dissolved in the 20 mL of mixed solvent of degassed THF and toluene (1:1) under nitrogen atmosphere. To the solution was added trichlorosilane (2.02 mL, 20.0mmol) at room temperature, and the mixture was stirred at 100 ℃ for 4 h. After cooling to ambient temperature, the mixture was diluted with diethyl ether. To the solution was added ice (50 g) and 20% NaOH solution (50 mL). The mixture was transferred to a separating funnel and shaked for 10 min. The organic layer was separated and washed successively with saturated $NaHCO_3$, brine and water. The solution was then dried over anhydrous Na_2SO_4 and the solvent removed under reduced pressure. The residue was purified by flash chromatography on silica gel (petroleum ether/EtOAc 50:1) to afford (*S*)-**E** (0.484 g, 84%) as a white solid, mp = 275-277 ℃. ^{1}H NMR (600 MHz, $CDCl_3$) δ 7.50-7.43 (m, 4H), 7.34-7.27 (m, 6H), 7.24-7.20 (m, 2H), 7.19-7.14 (m, 6H), 7.11-7.05 (m, 4H), 6.97 (d, *J* = 7.5 Hz, 2H), 6.92 (d, *J* = 7.6 Hz, 2H), 1.87 (dd, *J* = 13.3, 7.8 Hz, 2H), 1.77-1.66 (m, 2H), 1.49-1.39 (m, 2H), 1.20-1.08 (m, 2H); ^{13}C NMR (100 MHz, $CDCl_3$) δ 144.68, 144.65, 144.62, 142.47, 142.31, 142.15, 139.03, 138.95, 138.87, 138.46, 136.52, 136.47, 136.42, 135.40, 135.28, 135.17, 133.32, 133.22, 133.12, 130.38, 129.42, 128.65, 128.36, 128.01, 127.98, 127.94, 127.86, 31.82, 30.16; ^{31}P NMR (243 MHz, $CDCl_3$) δ −9.37; HRMS (ESI$^+$): Calcd for $C_{40}H_{35}P_2$, [M+H]$^+$ *m*/*z* 577.2214. Found 577.2215. (*R*)-**E** was prepared by using the similar procedures.

(b) Synthesis of (*S*)-1,13-bis(diphenylphosphanyl)-6,7,8,9-tetrahydro-5*H*-dibenzo-[*a*,*c*][9]annulene ((*S*)-F)

(*S*)-**F** was synthesized by the same procedure as that for (*S*)-**E** as a white solid. Yield 87%. mp = 225-226 ℃. ^{1}H NMR (400 MHz, $CDCl_3$) δ 7.35-7.18 (m, 23H), 7.04 (d, *J* = 7.5 Hz, 2H), 6.97 (d, *J* = 7.6 Hz, 2H), 1.75-1.63 (m, 2H), 1.51-1.38 (m, 2H), 1.34-1.23 (m, 2H), 1.23-1.11 (m, 2H), 1.11-0.99 (m, 2H); ^{13}C NMR (100 MHz, $CDCl_3$) δ 144.83, 144.66, 144.48, 143.35, 143.31, 143.28, 139.26, 139.19, 139.12, 138.14, 136.12, 136.06, 136.01, 135.63, 135.51, 135.40, 133.00, 132.90, 132.80, 131.21, 129.54, 128.84, 128.26, 128.18, 128.15, 128.11, 127.92, 127.67, 32.72, 29.56, 28.11; ^{31}P NMR (121 MHz, $CDCl_3$) δ −12.45; HRMS (ESI$^+$): Calcd for $C_{41}H_{37}P_2$, $[M+H]^+$ *m*/*z* 591.2370. Found 591.2361. (*R*)-**F** was prepared by using the similar procedures.

(c) Synthesis of (*S*)-1,14-bis(diphenylphosphanyl)-5,6,7,8,9,10-hexahydrodibenzo-[*a*,*c*][10]annulene ((*S*)-G)

(*S*)-**G** was synthesized by the same procedure as that for (*S*)-**E** as a white solid. Yield 80%. mp = 247-249 ℃. ^{1}H NMR (400 MHz, $CDCl_3$) δ 7.35-7.26 (m, 12H), 7.23-7.16 (m, 6H), 7.14-7.06 (m, 6H), 7.06-7.00 (m, 2H), 1.84 (td, *J* = 13.6, 3.6 Hz, 2H), 1.56-1.49 (m, 2H), 1.43-1.32 (m, 2H), 1.20-1.08 (m, 4H), 0.71-0.49 (m, 2H); ^{13}C NMR (100 MHz, $CDCl_3$) δ 147.04, 146.99, 146.80, 146.60, 146.55, 142.06, 142.02, 141.98, 139.36, 139.28, 139.22, 138.28, 136.65, 136.58, 136.52, 135.55, 135.42, 135.30, 135.19, 135.06, 133.24, 133.10, 133.00, 132.90, 132.77, 132.30, 129.16, 128.85, 128.28, 128.19, 127.88, 127.62, 28.96, 28.43, 21.09; ^{31}P NMR (121 MHz, $CDCl_3$) δ −14.16; HRMS (ESI$^+$): Calcd for $C_{42}H_{39}P_2$, $[M+H]^+$ *m*/*z* 605.2527. Found 605.2524. (*R*)-**G** was prepared by using the similar procedures.

3. Synthesis and Characterizaion Data of Substrates

As shown in Scheme S1, compounds **S4** were synthesized from the corresponding carboxylic acid as starting materials according to the literature procedure (Svenstrup et al., 1999). Compounds **S5** were synthesized by the corresponding substituted toluene (Roberts et al., 2015). Compounds **S6** were synthesized from the corresponding substituted hydrazine hydrochloride and the corresponding **S4** (Sheng et al., 2015). Compounds **S6** were performed from the corresponding **S5** and the corresponding **S4** according to the previous procedure (Yang et al., 2013).

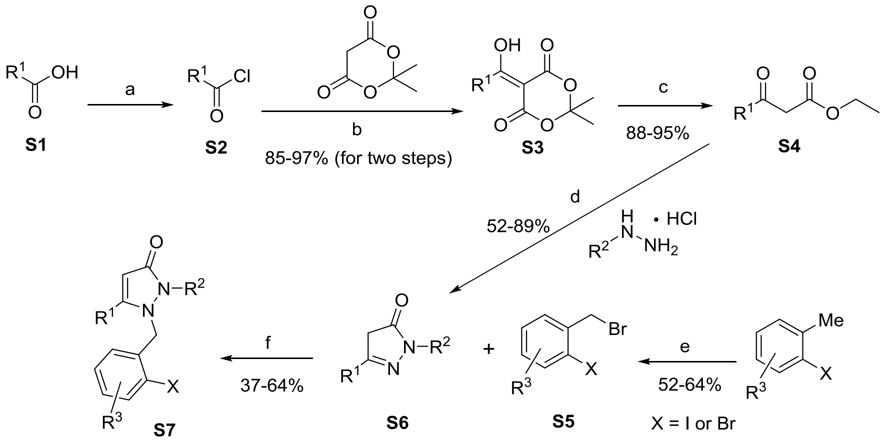

Scheme S1. Synthetic routes of compounds **1a-ah** and **3**. Reagents and conditions: (a) $SOCl_2$, overnight; (b) Pyridine, CH_2Cl_2, 0 ℃ to rt, 2 h; (c) Abs. EtOH, reflux, 2.5 h; (d) AcONa, AcOH, reflux, 5–10 h; (e) NBS, CCl_4, $(PhCO_2)_2$, reflux; (f) CH_3CN, 120 ℃, 24 h.

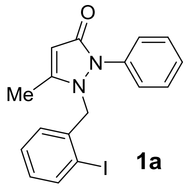

1a

1-(2-Iodobenzyl)-5-methyl-2-phenyl-1,2-dihydro-3*H*-pyrazol-3-one (1a): Pale yellow solid, mp = 173-174 ℃; Eluent: EtOAc; ^{1}H NMR (400 MHz, $CDCl_3$) δ 7.74 (d, *J* = 7.9 Hz, 1H), 7.41 (t, *J* = 7.7 Hz, 2H), 7.33-7.25 (m, 4H), 6.95 (t, *J* = 7.5 Hz, 1H), 6.82 (d, *J* = 7.8 Hz, 1H), 5.47 (s, 1H), 4.73 (s, 2H), 2.19 (s, 3H); ^{13}C NMR (100 MHz, $CDCl_3$) δ 166.53, 154.01, 139.57, 137.58, 134.49, 129.54, 129.41, 128.96, 127.76, 126.43, 125.86, 97.92, 96.60, 54.46, 12.87; HRMS (ESI$^+$): Calcd for $C_{17}H_{16}IN_2O$, $[M+H]^+$ *m*/*z* 391.0307. Found 391.0305.

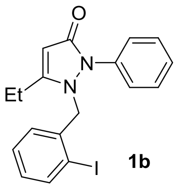

1b

5-Ethyl-1-(2-iodobenzyl)-2-phenyl-1,2-dihydro-3*H*-pyrazol-3-one (2a): Pale yellow solid, mp = 120-121 ℃; Eluent: EtOAc; ^{1}H NMR (400 MHz, $CDCl_3$) δ 7.72 (d, *J* = 8.0 Hz, 1H), 7.41 (t, *J* = 7.7 Hz, 2H), 7.32-7.23 (m, 4H), 6.93 (t, *J* = 7.6 Hz, 1H), 6.80 (d, *J* = 7.8 Hz, 1H), 5.49 (s, 1H), 4.73 (s, 2H), 2.46 (q, *J* = 7.5 Hz, 2H), 1.26 (t, *J* = 7.5 Hz, 3H); ^{13}C NMR (100 MHz, $CDCl_3$) δ 166.66, 159.89, 139.55, 137.76, 134.46, 129.50, 129.40, 128.93, 127.73, 126.47, 125.86, 96.62, 96.24, 54.43, 20.06, 11.61; HRMS (ESI$^+$): Calcd for $C_{18}H_{18}IN_2O$, $[M+H]^+$ *m/z* 405.0464. Found 405.0454.

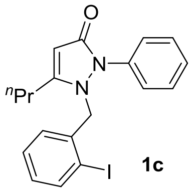

1c

1-(2-Iodobenzyl)-2-phenyl-5-propyl-1,2-dihydro-3*H*-pyrazol-3-one (1c): Pale yellow solid, mp = 108-109 ℃; Eluent: EtOAc; ^{1}H NMR (400 MHz, $CDCl_3$) δ 7.72 (d, *J* = 7.9 Hz, 1H), 7.41 (t, *J* = 7.8 Hz, 2H), 7.32-7.23 (m, 4H), 6.93 (t, *J* = 7.6 Hz, 1H), 6.81 (d, *J* = 7.1 Hz, 1H), 5.48 (s, 1H), 4.73 (s, 2H), 2.41 (t, *J* = 7.6 Hz, 2H), 1.70 (sext, *J* = 7.4 Hz, 2H), 1.01 (t, *J* = 7.4 Hz, 3H); ^{13}C NMR (100 MHz, $CDCl_3$) δ 166.65, 158.43, 139.54, 137.77, 134.47, 129.50, 129.39, 128.91, 127.72, 126.51, 125.87, 96.91, 96.64, 54.43, 28.59, 20.86, 13.76; HRMS (ESI$^+$): Calcd for $C_{19}H_{20}IN_2O$, $[M+H]^+$ *m/z* 419.0620. Found 419.0612.

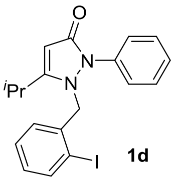

1d

1-(2-Iodobenzyl)-5-isopropyl-2-phenyl-1,2-dihydro-3*H*-pyrazol-3-one (1d): Pale yellow solid, mp = 164-165 ℃; Eluent: EtOAc; ^{1}H NMR (400 MHz, $CDCl_3$) δ 7.71 (d, *J* = 7.9 Hz, 1H), 7.40 (t, *J* = 7.7 Hz, 2H), 7.32-7.22 (m, 4H), 6.91 (t, *J* = 7.7 Hz, 1H), 6.77 (d, *J* = 7.8 Hz, 1H), 5.48 (s, 1H), 4.76 (s, 2H), 2.68 (hept, *J* = 6.8 Hz, 1H), 1.26 (d, *J* = 6.8 Hz, 6H); ^{13}C NMR (100 MHz, $CDCl_3$) δ 166.55, 164.65, 139.47, 137.93, 134.34, 129.44, 129.33, 128.83, 127.67, 126.38, 125.86, 96.57,

94.97, 54.38, 26.22, 22.03; HRMS (ESI$^+$): Calcd for $C_{19}H_{20}IN_2O$, $[M+H]^+$ *m/z* 419.0620. Found 419.0613.

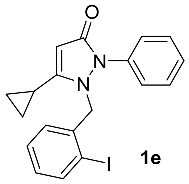

1e

5-Cyclopropyl-1-(2-iodobenzyl)-2-phenyl-1,2-dihydro-3*H*-pyrazol-3-one (1e): Pale yellow solid, mp = 135-136 ℃; Eluent: EtOAc; ^{1}H NMR (400 MHz, $CDCl_3$) δ 7.73 (d, *J* = 7.9 Hz, 1H), 7.43-7.37 (m, 2H), 7.32-7.23 (m, 4H), 6.96-6.87 (m, 2H), 5.17 (s, 1H), 4.88 (s, 2H), 1.66-1.53 (m, 1H), 0.99-0.93 (m, 2H), 0.71-0.66 (m, 2H); ^{13}C NMR (100 MHz, $CDCl_3$) δ 166.70, 161.17, 139.48, 137.96, 134.60, 129.42, 129.34, 128.79, 127.52, 126.88, 125.55, 97.03, 93.30, 55.15, 8.27, 7.70; HRMS (ESI$^+$): Calcd for $C_{19}H_{18}IN_2O$, $[M+H]^+$ *m/z* 417.0664. Found 417.0462.

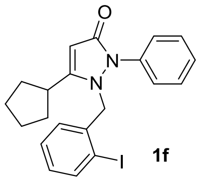

1f

5-Cyclopentyl-1-(2-iodobenzyl)-2-phenyl-1,2-dihydro-3*H*-pyrazol-3-one (1f): Pale yellow solid, mp = 166-167 ℃; Eluent: EtOAc; ^{1}H NMR (400 MHz, $CDCl_3$) δ 7.72 (d, *J* = 7.9 Hz, 1H), 7.41 (t, *J* = 7.6 Hz, 2H), 7.33-7.21 (m, 4H), 6.93 (t, *J* = 7.5 Hz, 1H), 6.80 (d, *J* = 7.7 Hz, 1H), 5.48 (s, 1H), 4.78 (s, 2H), 2.76 (quint, *J* = 7.8 Hz, 1H), 2.05-1.91 (m, 2H), 1.83-1.59 (m, 6H); ^{13}C NMR (150 MHz, $CDCl_3$) δ 166.74, 163.03, 139.48, 138.06, 134.39, 129.44, 129.37, 128.87, 127.72, 126.59, 125.93, 96.57, 95.01, 54.55, 36.98, 32.67, 25.26; HRMS (ESI$^+$): Calcd for $C_{21}H_{22}IN_2O$, $[M+H]^+$ *m/z* 445.0777. Found 445.0769.

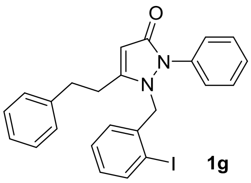

1g

1-(2-Iodobenzyl)-5-phenethyl-2-phenyl-1,2-dihydro-3*H*-pyrazol-3-one (1g): Pale yellow solid, mp = 200-201 ℃; Eluent: EtOAc; ^{1}H NMR (400 MHz, $CDCl_3$) δ 7.71 (d, *J* = 7.9 Hz, 1H), 7.41 (t, *J* = 7.5 Hz, 2H), 7.36-7.15 (m, 9H), 6.93 (t, *J* = 7.6 Hz, 1H), 6.67 (d, *J* = 7.6 Hz, 1H), 5.69 (s, 1H), 4.66 (s, 2H), 2.98 (t, *J* = 7.4 Hz, 2H), 2.80 (t, *J* = 7.5 Hz, 2H); ^{13}C NMR (100 MHz, $CDCl_3$) δ 165.34, 156.61, 139.67, 139.50, 137.28, 133.50, 129.73, 129.61, 129.05, 128.77, 128.59, 128.52,

126.81, 126.44, 96.46, 96.37, 54.41, 33.90, 28.47; HRMS (ESI^+): Calcd for $C_{24}H_{22}IN_2O$, $[M+H]^+$ *m*/*z* 481.0777. Found 481.0771.

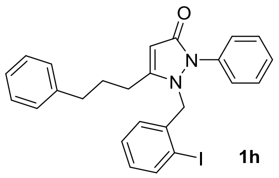

1h

1-(2-Iodobenzyl)-2-phenyl-5-(3-phenylpropyl)-1,2-dihydro-3*H*-pyrazol-3-one (1h): Pale yellow solid, mp = 172-173 ℃; Eluent: EtOAc; ^{1}H NMR (400 MHz, $CDCl_3$) δ 7.73 (d, *J* = 7.9 Hz, 1H), 7.49 (t, *J* = 7.4 Hz, 1H), 7.40 (t, *J* = 7.7 Hz, 2H), 7.33-7.16 (m, 6H), 7.08 (d, *J* = 7.2 Hz, 2H), 7.00 (t, *J* = 7.6 Hz, 1H), 6.68 (s, 1H), 6.41 (d, *J* = 7.7 Hz, 1H), 5.06 (s, 2H), 2.71-2.60 (m, 4H), 1.99 (quint, *J* = 7.6 Hz, 2H); ^{13}C NMR (100 MHz, $CDCl_3$) δ 158.85, 154.01, 140.16, 140.14, 135.17, 131.97, 130.47, 130.27, 129.47, 129.22, 128.71, 128.45, 128.41, 126.46, 126.20, 96.19, 92.39, 54.68, 34.95, 28.41, 25.95; HRMS (ESI^+): Calcd for $C_{25}H_{24}IN_2O$, $[M+H]^+$ *m*/*z* 495.0933. Found 495.0931.

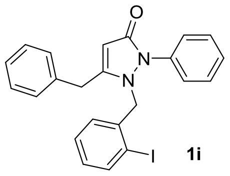

1i

5-Benzyl-1-(2-iodobenzyl)-2-phenyl-1,2-dihydro-3*H*-pyrazol-3-one (1i): Pale yellow solid, mp = 133-134 ℃; Eluent: EtOAc; ^{1}H NMR (400 MHz, $CDCl_3$) δ 7.72 (d, *J* = 8.7 Hz, 1H), 7.39 (t, *J* = 7.7 Hz, 2H), 7.34-7.23 (m, 7H), 7.19 (d, *J* = 6.9 Hz, 2H), 6.93 (t, *J* = 7.1 Hz, 1H), 6.84-6.80 (m, 1H), 5.41 (s, 1H), 4.69 (s, 2H), 3.76 (s, 2H); ^{13}C NMR (100 MHz, $CDCl_3$) δ 166.27, 156.81, 139.58, 137.75, 135.30, 134.29, 129.53, 129.43, 128.94, 128.91, 128.78, 127.86, 127.37, 126.45, 125.90, 98.91, 96.51, 54.51, 33.30; HRMS (ESI^+): Calcd for $C_{22}H_{20}IN_2O$, $[M+H]^+$ *m*/*z* 467.0620. Found 467.0615.

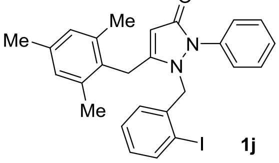

1j

1-(2-Iodobenzyl)-2-phenyl-5-(2,4,6-trimethylbenzyl)-1,2-dihydro-3*H*-pyrazol-3-one (1j): Pale yellow solid, mp = 194-195 ℃; Eluent: EtOAc; ^{1}H NMR (400 MHz, $CDCl_3$) δ 7.83-7.75 (m, 1H), 7.44 (t, *J* = 7.8 Hz, 2H), 7.38-7.28 (m, 4H), 7.03-6.94 (m, 2H), 6.84 (s, 2H), 4.89 (s, 1H), 4.85 (s,

2H), 3.63 (s, 2H), 2.26 (s, 3H), 2.05 (s, 6H); ^{13}C NMR (100 MHz, $CDCl_3$) δ 166.59, 157.66, 139.78, 137.89, 136.93, 136.75, 134.53, 129.76, 129.49, 129.20, 129.17, 128.90, 127.61, 126.81, 125.45, 98.53, 97.19, 54.84, 27.36, 20.99, 19.67; HRMS (ESI$^+$): Calcd for $C_{26}H_{26}IN_2O$, $[M+H]^+$ *m*/*z* 509.1090. Found 509.1078.

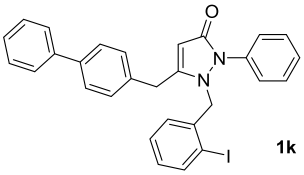

1k

5-([1,1'-Biphenyl]-4-ylmethyl)-1-(2-iodobenzyl)-2-phenyl-1,2-dihydro-3*H*-pyrazol-3-one (1k): Pale yellow solid, mp = 184-185 ℃; Eluent: EtOAc; ^{1}H NMR (400 MHz, $CDCl_3$) δ 7.71 (d, *J* = 7.9 Hz, 1H), 7.56 (d, *J* = 7.6 Hz, 2H), 7.52 (d, *J* = 8.1 Hz, 2H), 7.46-7.22 (m, 12H), 6.92 (t, *J* = 7.5 Hz, 1H), 6.83 (d, *J* = 7.7 Hz, 1H), 5.49 (s, 1H), 4.70 (s, 2H), 3.80 (s, 2H); ^{13}C NMR (100 MHz, $CDCl_3$) δ 166.26, 156.60, 140.51, 140.28, 139.55, 137.78, 134.28, 129.51, 129.47, 129.20, 128.94, 128.87, 127.89, 127.59, 127.47, 127.06, 126.48, 125.91, 98.99, 96.53, 54.54, 32.99; HRMS (ESI$^+$): Calcd for $C_{29}H_{24}IN_2O$, $[M+H]^+$ *m*/*z* 543.0933. Found 543.0928.

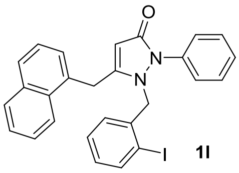

1l

1-(2-Iodobenzyl)-5-(naphthalen-1-ylmethyl)-2-phenyl-1,2-dihydro-3*H*-pyrazol-3-one (1l): Pale yellow solid, mp = 205-206 ℃; Eluent: EtOAc; ^{1}H NMR (400 MHz, $CDCl_3$) δ 7.83 (d, *J* = 8.0 Hz, 1H), 7.77 (d, *J* = 8.2 Hz, 1H), 7.73 (d, *J* = 7.8 Hz, 1H), 7.63 (d, *J* = 8.3 Hz, 1H), 7.46 (t, *J* = 7.3 Hz, 1H), 7.43-7.36 (m, 4H), 7.33-7.23 (m, 5H), 6.99-6.91 (m, 2H), 5.16 (s, 1H), 4.79 (s, 2H), 4.15 (s, 2H); ^{13}C NMR (100 MHz, $CDCl_3$) δ 166.23, 157.00, 139.66, 137.80, 134.31, 133.86, 131.64, 131.11, 129.60, 129.42, 129.00, 128.85, 128.39, 127.73, 127.41, 126.60, 126.47, 125.96, 125.71, 125.48, 123.43, 99.58, 96.82, 54.73, 30.95; HRMS (ESI$^+$): Calcd for $C_{27}H_{22}IN_2O$, $[M+H]^+$ *m*/*z* 517.0777. Found 517.0774.

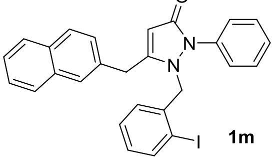

1m

1-(2-Iodobenzyl)-5-(naphthalen-2-ylmethyl)-2-phenyl-1,2-dihydro-3*H*-pyrazol-3-one (1m): Pale yellow solid, mp = 196-197 ℃; Eluent: EtOAc; ^{1}H NMR (400 MHz, $CDCl_3$) δ 7.86-7.74 (m, 3H), 7.69 (d, *J* = 7.9 Hz, 1H), 7.64 (s, 1H), 7.53-7.44 (m, 2H), 7.39 (t, *J* = 7.6 Hz, 2H), 7.33-7.20 (m, 5H), 6.89 (t, *J* = 7.6 Hz, 1H), 6.83 (d, *J* = 7.7 Hz, 1H), 5.52 (s, 1H), 4.69 (s, 2H), 3.92 (s, 2H); ^{13}C NMR (100 MHz, $CDCl_3$) δ 166.33, 156.49, 139.56, 137.87, 134.30, 133.45, 132.82, 132.58, 129.50, 128.98, 128.78, 127.92, 127.76, 127.72, 127.61, 126.67, 126.50, 126.44, 126.14, 125.93, 99.13, 96.48, 54.50, 33.59; HRMS (ESI$^+$): Calcd for $C_{27}H_{22}IN_2O$, $[M+H]^+$ *m/z* 517.0777. Found 517.0769.

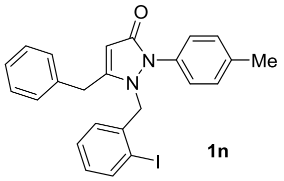

1n

5-Benzyl-1-(2-iodobenzyl)-2-(*p*-tolyl)-1,2-dihydro-3*H*-pyrazol-3-one (1n): Pale yellow solid, mp = 148-149 ℃; Eluent: EtOAc; ^{1}H NMR (600 MHz, $CDCl_3$) δ 7.73 (d, *J* = 7.9 Hz, 1H), 7.31 (t, *J* = 7.3 Hz, 2H), 7.28-7.24 (m, 2H), 7.21-7.17 (m, 4H), 7.12 (d, *J* = 8.3 Hz, 2H), 6.94 (t, *J* = 8.2 Hz, 1H), 6.80 (d, *J* = 7.7 Hz, 1H), 5.41 (s, 1H), 4.66 (s, 2H), 3.74 (s, 2H), 2.33 (s, 3H); ^{13}C NMR (150 MHz, $CDCl_3$) δ 166.31, 155.99, 139.58, 138.28, 137.98, 135.39, 131.56, 130.14, 129.52, 129.00, 128.93, 128.83, 127.39, 126.46, 126.31, 98.61, 96.36, 54.31, 33.31, 21.22; HRMS (ESI$^+$): Calcd for $C_{24}H_{22}IN_2O$, $[M+H]^+$ *m/z* 481.0777. Found 481.0774.

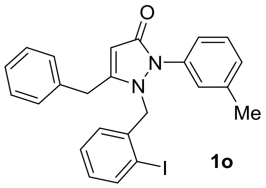

1o

5-Benzyl-1-(2-iodobenzyl)-2-(*m*-tolyl)-1,2-dihydro-3*H*-pyrazol-3-one (1o): Pale yellow solid, mp = 159-160 ℃; Eluent: EtOAc; ^{1}H NMR (400 MHz, $CDCl_3$) δ 7.72 (d, *J* = 7.9 Hz, 1H), 7.34-7.23 (m, 5H), 7.19 (d, *J* = 7.1 Hz, 2H), 7.12-7.06 (m, 2H), 7.02 (d, *J* = 7.9 Hz, 1H), 6.93 (t, *J* = 7.6 Hz, 1H), 6.82 (d, *J* = 7.7 Hz, 1H), 5.40 (s, 1H), 4.67 (s, 2H), 3.75 (s, 2H), 2.32 (s, 3H); ^{13}C NMR (100 MHz, $CDCl_3$) δ 166.24, 156.40, 139.51, 139.45, 137.87, 135.32, 134.12, 129.48, 129.17, 128.91, 128.87, 128.78, 127.33, 126.84, 126.48, 123.08, 98.77, 96.50, 54.43, 33.27, 21.38; HRMS (ESI$^+$): Calcd for $C_{24}H_{22}IN_2O$, $[M+H]^+$ *m/z* 481.0777. Found 481.0774.

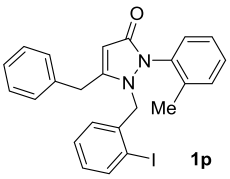

1p

5-Benzyl-1-(2-iodobenzyl)-2-(*o*-tolyl)-1,2-dihydro-3*H*-pyrazol-3-one (1p): Pale yellow solid, mp = 197-198 ℃; Eluent: EtOAc; ^{1}H NMR (400 MHz, $CDCl_3$) δ 7.73 (d, *J* = 7.8 Hz, 1H), 7.35-7.20 (m, 8H), 7.19-7.11 (m, 1H), 7.01-6.91 (m, 2H), 6.76 (d, *J* = 7.6 Hz, 1H), 5.46 (s, 1H), 4.61 (d, *J* = 17.9 Hz, 1H), 4.45 (d, *J* = 17.9 Hz, 1H), 3.89-3.71 (m, 2H), 2.04 (s, 3H); ^{13}C NMR (100 MHz, $CDCl_3$) δ 166.32, 154.89, 139.63, 137.93, 137.80, 135.50, 133.07, 131.56, 129.57, 128.96, 128.86, 128.68, 127.40, 126.90, 126.39, 97.84, 96.24, 53.88, 33.22, 17.57; HRMS (ESI^{+}): Calcd for $C_{24}H_{22}IN_2O$, $[M+H]^{+}$ *m/z* 481.0777. Found 481.0773.

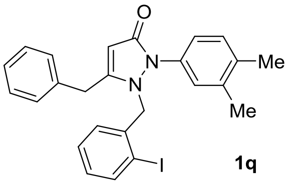

1q

5-Benzyl-2-(3,4-dimethylphenyl)-1-(2-iodobenzyl)-1,2-dihydro-3*H*-pyrazol-3-one (1q): Pale yellow solid, mp = 166-167 ℃; Eluent: EtOAc; ^{1}H NMR (400 MHz, $CDCl_3$) δ 7.72 (d, *J* = 7.8 Hz, 1H), 7.33-7.22 (m, 4H), 7.18 (d, *J* = 7.1 Hz, 2H), 7.13 (d, *J* = 8.0 Hz, 1H), 7.02 (s, 1H), 6.96-6.91 (m, 2H), 6.81 (d, *J* = 7.7 Hz, 1H), 5.40 (s, 1H), 4.65 (s, 2H), 3.73 (s, 2H), 2.22 (s, 3H), 2.21 (s, 3H); ^{13}C NMR (100 MHz, $CDCl_3$) δ 166.25, 155.60, 139.46, 138.05, 137.95, 137.08, 135.37, 131.65, 130.47, 129.42, 128.92, 128.85, 128.77, 127.71, 127.29, 126.44, 123.84, 98.44, 96.32, 54.18, 33.22, 19.87, 19.50; HRMS (ESI^{+}): Calcd for $C_{25}H_{24}IN_2O$, $[M+H]^{+}$ *m/z* 495.0933. Found 495.0925.

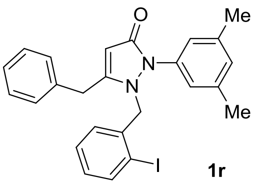

1r

5-Benzyl-2-(3,5-dimethylphenyl)-1-(2-iodobenzyl)-1,2-dihydro-3*H*-pyrazol-3-one (1r): Pale yellow solid, mp = 200-201 ℃; Eluent: EtOAc; ^{1}H NMR (400 MHz, $CDCl_3$) δ 7.73 (d, *J* = 7.9 Hz, 1H), 7.34-7.24 (m, 4H), 7.19 (d, *J* = 7.2 Hz, 2H), 6.97-6.91 (m, 2H), 6.85-6.80 (m, 3H), 5.39 (s, 1H), 4.67 (s, 2H), 3.74 (s, 2H), 2.27 (s, 6H); ^{13}C NMR (100 MHz, $CDCl_3$) δ 166.31, 156.01, 139.53, 139.20, 138.04, 135.42, 133.98, 130.08, 129.50, 128.96, 128.91, 128.83, 127.35, 126.58,

124.17, 98.71, 96.52, 54.40, 33.30, 21.31; HRMS (ESI$^+$): Calcd for $C_{25}H_{24}IN_2O$, $[M+H]^+$ *m/z* 495.0933. Found 495.0927.

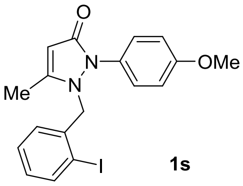

1s

1-(2-Iodobenzyl)-2-(4-methoxyphenyl)-5-methyl-1,2-dihydro-3*H*-pyrazol-3-one (1s): Pale yellow solid, mp = 105-106 ℃; Eluent: EtOAc; ^{1}H NMR (600 MHz, $CDCl_3$) δ 7.75 (d, *J* = 7.9 Hz, 1H), 7.32-7.26 (m, 1H), 7.13 (d, *J* = 8.9 Hz, 2H), 6.96 (t, *J* = 7.1 Hz, 1H), 6.91 (d, *J* = 8.9 Hz, 2H), 6.77 (d, *J* = 7.5 Hz, 1H), 5.45 (s, 1H), 4.68 (s, 2H), 3.80 (s, 3H), 2.18 (s, 3H); ^{13}C NMR (150 MHz, $CDCl_3$) δ 166.75, 159.63, 152.56, 139.64, 137.91, 129.60, 129.07, 128.48, 127.05, 126.46, 114.93, 97.30, 96.40, 55.65, 54.13, 12.79; HRMS (ESI$^+$): Calcd for $C_{18}H_{18}IN_2O$, $[M+H]^+$ *m/z* 421.0413. Found 421.0406.

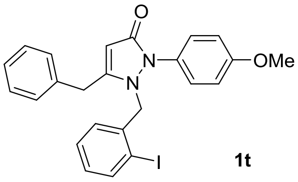

1t

5-Benzyl-1-(2-iodobenzyl)-2-(4-methoxyphenyl)-1,2-dihydro-3*H*-pyrazol-3-one (1t): Pale yellow solid, mp = 120-121 ℃; Eluent: EtOAc; ^{1}H NMR (600 MHz, $CDCl_3$) δ 7.74 (d, *J* = 7.9 Hz, 1H), 7.31 (t, *J* = 6.8 Hz, 2H), 7.29-7.24 (m, 2H), 7.19 (d, *J* = 7.5 Hz, 2H), 7.13 (d, *J* = 8.8 Hz, 2H), 6.95 (t, *J* = 7.6 Hz, 1H), 6.90 (d, *J* = 8.8 Hz, 2H), 6.77 (d, *J* = 7.7 Hz, 1H), 5.41 (s, 1H), 4.63 (s, 2H), 3.78 (s, 3H), 3.76 (s, 2H); ^{13}C NMR (150 MHz, $CDCl_3$) δ 166.42, 159.68, 155.28, 139.61, 138.02, 135.44, 129.57, 129.02, 128.97, 128.84, 128.50, 127.42, 126.76, 126.48, 114.93, 98.24, 96.30, 55.64, 54.18, 33.32; HRMS (ESI$^+$): Calcd for $C_{24}H_{22}IN_2O$, $[M+H]^+$ *m/z* 497.0726. Found 497.0719.

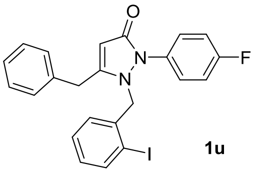

1u

5-Benzyl-2-(4-fluorophenyl)-1-(2-iodobenzyl)-1,2-dihydro-3*H*-pyrazol-3-one (1u): Pale yellow solid, mp = 123-124 ℃; Eluent: EtOAc; ^{1}H NMR (600 MHz, $CDCl_3$) δ 7.74 (d, *J* = 7.9 Hz, 1H), 7.33 (t, *J* = 7.3 Hz, 2H), 7.29-7.25 (m, 2H), 7.22-7.18 (m, 4H), 7.08 (t, *J* = 8.6 Hz, 2H), 6.96 (t, *J* =

8.2 Hz, 1H), 6.78 (d, J = 7.6 Hz, 1H), 5.41 (s, 1H), 4.66 (s, 2H), 3.79 (s, 2H); ^{13}C NMR (150 MHz, $CDCl_3$) δ 166.53, 162.06 (d, $^{1}J_{C\text{-}F}$ = 248.5 Hz), 156.98, 139.76, 137.60, 135.29, 130.40, 129.72, 129.04, 128.86, 128.22 (d, $^{3}J_{C\text{-}F}$ = 8.7 Hz), 127.53, 126.54, 116.54 (d, $^{2}J_{C\text{-}F}$ = 23.0 Hz), 98.81, 96.62, 54.65, 33.43; ^{19}F NMR (283 MHz, $CDCl_3$) δ -112.76; HRMS (ESI$^+$): Calcd for $C_{23}H_{19}FIN_2O$, $[M+H]^+$ m/z 485.0526. Found 485.0520.

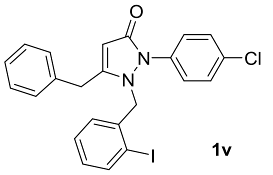

1v

5-Benzyl-2-(4-chlorophenyl)-1-(2-iodobenzyl)-1,2-dihydro-3*H*-pyrazol-3-one (1v): Pale yellow solid, mp = 181-182 ℃; Eluent: EtOAc; ^{1}H NMR (400 MHz, $CDCl_3$) δ 7.72 (d, J = 7.8 Hz, 1H), 7.35 (d, J = 7.6 Hz, 2H), 7.31-7.12 (m, 8H), 6.97 (t, J = 7.5 Hz, 1H), 6.39 (d, J = 7.1 Hz, 1H), 6.23 (s, 1H), 5.16 (s, 2H), 4.00 (s, 2H); ^{13}C NMR (100 MHz, $CDCl_3$) δ 158.80, 153.74, 140.13, 138.03, 135.10, 133.25, 130.47, 130.38, 130.05, 129.25, 129.21, 129.08, 128.15, 127.89, 126.69, 96.32, 93.54, 55.33, 33.54; HRMS (ESI$^+$): Calcd for $C_{23}H_{19}ClIN_2O$, $[M+H]^+$ m/z 501.0231. Found 501.0229.

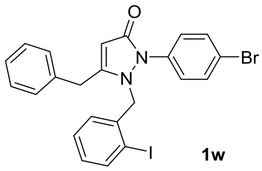

1w

5-Benzyl-2-(4-bromophenyl)-1-(2-iodobenzyl)-1,2-dihydro-3*H*-pyrazol-3-one (1w): Pale yellow solid, mp = 152-153 ℃; Eluent: EtOAc; ^{1}H NMR (400 MHz, $CDCl_3$) δ 7.75 (d, J = 7.9 Hz, 1H), 7.52 (d, J = 8.6 Hz, 2H), 7.36-7.24 (m, 5H), 7.19 (d, J = 7.0 Hz, 2H), 7.14 (d, J = 8.6 Hz, 2H), 6.96 (t, J = 7.1 Hz, 1H), 6.80 (d, J = 7.7 Hz, 1H), 5.40 (s, 1H), 4.68 (s, 2H), 3.78 (s, 2H); ^{13}C NMR (100 MHz, $CDCl_3$) δ 166.40, 158.00, 139.81, 137.50, 135.21, 133.58, 132.64, 129.77, 129.07, 128.89, 127.57, 127.13, 126.56, 121.48, 99.37, 96.80, 54.92, 33.51; HRMS (ESI$^+$): Calcd for $C_{23}H_{19}BrIN_2O$, $[M+H]^+$ m/z 544.9725. Found 544.9705.

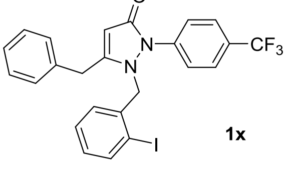

1x

5-Benzyl-1-(2-iodobenzyl)-2-(4-(trifluoromethyl)phenyl)-1,2-dihydro-3*H*-pyrazol-3-one (1x): Pale yellow solid, mp = 146-147 ℃; Eluent: EtOAc; ^{1}H NMR (600 MHz, $CDCl_3$) δ 7.75 (d, *J* = 7.7 Hz, 1H), 7.66 (d, *J* = 8.4 Hz, 2H), 7.42 (d, *J* = 8.3 Hz, 2H), 7.34 (t, *J* = 7.3 Hz, 2H), 7.31-7.24 (m, 2H), 7.20 (d, *J* = 7.1 Hz, 2H), 6.95 (t, *J* = 7.6 Hz, 1H), 6.85 (d, *J* = 7.6 Hz, 1H), 5.42 (s, 1H), 4.73 (s, 2H), 3.81 (s, 2H); ^{13}C NMR (100 MHz, $CDCl_3$) δ 166.40, 159.57, 139.81, 137.82, 137.15, 135.02, 129.78, 129.01, 128.97, 128.84, 128.86 (q, $^2J_{C-F}$ = 32.7 Hz), 127.53, 126.67, 126.47(q, $^3J_{C-F}$ = 3.5 Hz), 124.66, 123.84 (q, $^1J_{C-F}$ = 270.7 Hz), 99.86, 97.07, 55.40, 33.53; ^{19}F NMR (283 MHz, $CDCl_3$) δ -62.29; HRMS (ESI$^+$): Calcd for $C_{24}H_{19}F_3IN_2O$, $[M+H]^+$ *m/z* 535.0494. Found 535.0486.

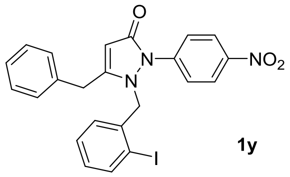

1y

5-Benzyl-1-(2-iodobenzyl)-2-(4-nitrophenyl)-1,2-dihydro-3*H*-pyrazol-3-one (1y): Pale yellow solid, mp = 131-132 ℃; Eluent: EtOAc; ^{1}H NMR (400 MHz, $CDCl_3$) δ 8.25 (d, *J* = 9.0 Hz, 2H), 7.76 (d, *J* = 8.8 Hz, 1H), 7.48 (d, *J* = 9.0 Hz, 2H), 7.38-7.20 (m, 6H), 6.96 (t, *J* = 7.6 Hz, 1H), 6.88 (d, *J* = 8.6 Hz, 1H), 5.43 (s, 1H), 4.75 (s, 2H), 3.84 (s, 2H); ^{13}C NMR (100 MHz, $CDCl_3$) δ 166.43, 161.43, 145.47, 140.59, 140.02, 136.78, 134.87, 130.03, 129.15, 129.05, 128.92, 127.72, 126.94, 124.84, 123.86, 100.64, 97.52, 56.19, 33.79; HRMS (ESI$^+$): Calcd for $C_{23}H_{19}IN_3O_3$, $[M+H]^+$ *m/z* 512.0471. Found 512.0465.

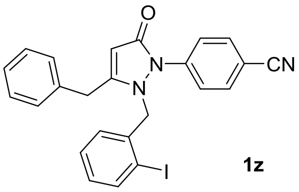

1z

4-(3-Benzyl-2-(2-iodobenzyl)-5-oxo-2,5-dihydro-1*H*-pyrazol-1-yl)benzonitrile (1z): Pale yellow solid, mp = 157-158 ℃; Eluent: EtOAc; ^{1}H NMR (600 MHz, $CDCl_3$) δ 7.90 (d, *J* = 8.9 Hz, 2H), 7.62 (d, *J* = 8.9 Hz, 2H), 7.41-7.35 (m, 1H), 7.35-7.30 (m, 2H), 7.18 (d, *J* = 8.1 Hz, 1H), 7.16-7.12 (m, 2H), 7.11-7.05 (m, 3H), 4.68 (d, *J* = 13.9 Hz, 1H), 3.98 (d, *J* = 13.8 Hz, 1H), 3.21 (s, 2H), 3.12 (d, *J* = 17.2 Hz, 1H), 2.95 (d, *J* = 17.2 Hz, 1H); ^{13}C NMR (150 MHz, $CDCl_3$) δ 170.72, 142.41, 141.68, 136.12, 135.09, 133.00, 130.36, 128.68, 128.35, 127.94, 127.03, 123.35, 122.50,

119.07, 118.57, 106.93, 73.59, 60.61, 45.40, 43.25; HRMS (ESI$^+$): Calcd for $C_{24}H_{19}IN_3O$, $[M+H]^+$ *m*/*z* 492.0573. Found 492.0570.

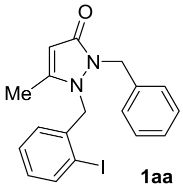

1aa

2-Benzyl-1-(2-iodobenzyl)-5-methyl-1,2-dihydro-3*H*-pyrazol-3-one (1aa): Pale yellow solid, mp = 147-148 ℃; Eluent: EtOAc; ^{1}H NMR (400 MHz, $CDCl_3$) δ 7.80 (d, *J* = 7.9 Hz, 1H), 7.30-7.20 (m, 4H), 7.16 (d, *J* = 7.7 Hz, 2H), 6.97 (t, *J* = 7.6 Hz, 1H), 6.46 (d, *J* = 7.7 Hz, 1H), 5.45 (s, 1H), 4.87 (s, 2H), 4.70 (s, 2H), 2.12 (s, 3H); ^{13}C NMR (100 MHz, $CDCl_3$) δ 166.06, 150.48, 139.63, 137.41, 136.03, 129.72, 129.09, 128.79, 127.87, 127.25, 126.13, 95.97, 95.80, 53.72, 45.60, 12.20; HRMS (ESI$^+$): Calcd for $C_{18}H_{18}IN_2O$, $[M+H]^+$ *m*/*z* 405.0464. Found 405.0460.

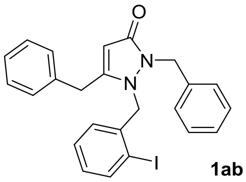

1ab

2,5-Dibenzyl-1-(2-iodobenzyl)-1,2-dihydro-3*H*-pyrazol-3-one (1ab): Pale yellow solid, mp = 156-157 ℃; Eluent: EtOAc; ^{1}H NMR (400 MHz, $CDCl_3$) δ 7.76 (d, *J* = 7.8 Hz, 1H), 7.27-7.07 (m, 11H), 6.94 (t, *J* = 7.6 Hz, 1H), 6.44 (d, *J* = 7.7 Hz, 1H), 5.40 (s, 1H), 4.88 (s, 2H), 4.64 (s, 2H), 3.72 (s, 2H); ^{13}C NMR (100 MHz, $CDCl_3$) δ 165.98, 153.30, 139.51, 137.41, 135.91, 135.20, 129.61, 128.93, 128.82, 128.75, 128.61, 127.85, 127.28, 127.14, 126.20, 96.81, 95.71, 53.85, 45.63, 32.64; HRMS (ESI$^+$): Calcd for $C_{24}H_{22}IN_2O$, $[M+H]^+$ *m*/*z* 481.0777. Found 481.0772.

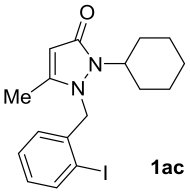

1ac

2-Cyclohexyl-1-(2-iodobenzyl)-5-methyl-1,2-dihydro-3*H*-pyrazol-3-one (1ac): Pale yellow solid, mp = 177-178 ℃; Eluent: EtOAc; ^{1}H NMR (400 MHz, $CDCl_3$) δ 7.84 (d, *J* = 7.9 Hz, 1H), 7.28-7.22 (m, 1H), 6.99 (t, *J* = 8.3 Hz, 1H), 6.59 (d, *J* = 7.8 Hz, 1H), 5.35 (s, 1H), 4.86 (s, 2H), 4.03 (tt, *J* = 12.3, 3.5 Hz, 1H), 2.11 (s, 3H), 1.88-1.74 (m, 4H), 1.66-1.61 (m, 3H), 1.31-1.15 (m, 3H); ^{13}C NMR (100 MHz, $CDCl_3$) δ 167.49, 152.18, 139.57, 137.99, 129.62, 129.05, 126.47, 97.83, 96.10, 56.02, 54.87, 30.77, 26.28, 25.35, 12.56; HRMS (ESI$^+$): Calcd for $C_{17}H_{22}IN_2O$, $[M+H]^+$ *m*/*z* 397.0777. Found 397.0775.

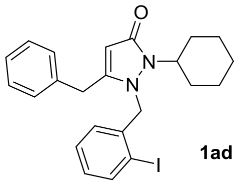

1ad

5-Benzyl-2-cyclohexyl-1-(2-iodobenzyl)-1,2-dihydro-3*H*-pyrazol-3-one (1ad): Pale yellow solid, mp = 112-113 ℃; Eluent: EtOAc; ^{1}H NMR (400 MHz, $CDCl_3$) δ 7.82 (d, *J* = 7.8 Hz, 1H), 7.31-7.20 (m, 4H), 7.15 (d, *J* = 6.9 Hz, 2H), 6.97 (t, *J* = 7.6 Hz, 1H), 6.59 (d, *J* = 7.7 Hz, 1H), 5.30 (s, 1H), 4.81 (s, 2H), 4.00 (tt, *J* = 12.3, 3.3 Hz, 1H), 3.70 (s, 2H), 1.87 (td, *J* = 12.4, 2.9 Hz, 2H), 1.81-1.72 (m, 2H), 1.68-1.58 (m, 3H), 1.31-1.13 (m, 3H); ^{13}C NMR (100 MHz, $CDCl_3$) δ 167.26, 154.80, 139.53, 138.06, 135.51, 129.59, 128.98, 128.87, 128.71, 127.27, 126.55, 98.90, 96.05, 56.16, 54.87, 33.06, 30.71, 26.27, 25.30; HRMS (ESI$^+$): Calcd for $C_{23}H_{26}IN_2O$, $[M+H]^+$ *m*/*z* 473.1090. Found 473.1079.

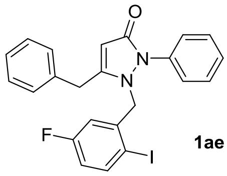

1ae

5-Benzyl-1-(5-fluoro-2-iodobenzyl)-2-phenyl-1,2-dihydro-3*H*-pyrazol-3-one (1ae): Pale yellow solid, mp = 191-192 ℃; Eluent: EtOAc; ^{1}H NMR (400 MHz, $CDCl_3$) δ 7.65 (dd, *J* = 8.6, 5.5 Hz, 1H), 7.40 (t, *J* = 7.7 Hz, 2H), 7.34-7.24 (m, 6H), 7.20 (d, *J* = 7.3 Hz, 2H), 6.70 (td, *J* = 8.3, 2.8 Hz, 1H), 6.59-6.53 (m, 1H), 5.46 (s, 1H), 4.64 (s, 2H), 3.78 (s, 2H); ^{13}C NMR (100 MHz, $CDCl_3$) δ 166.32, 163.33 (d, $^{1}J_{C\text{-}F}$ = 249.4 Hz), 156.72, 140.72 (d, $^{3}J_{C\text{-}F}$ = 7.6 Hz), 139.97 (d, $^{3}J_{C\text{-}F}$ = 6.9 Hz), 135.15, 134.26, 129.53, 128.99, 128.75, 127.99, 127.48, 125.89, 117.05 (d, $^{2}J_{C\text{-}F}$ = 21.9 Hz), 114.23 (d, $^{2}J_{C\text{-}F}$ = 24.1 Hz), 99.36, 89.15 (d, $^{4}J_{C\text{-}F}$ = 2.7 Hz), 54.32, 33.38; ^{19}F NMR (283 MHz, $CDCl_3$) δ -111.16; HRMS (ESI$^+$): Calcd for $C_{23}H_{19}FIN_2O$, $[M+H]^+$ *m*/*z* 485.0526. Found 485.0520.

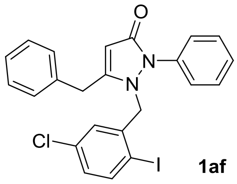

1af

5-Benzyl-1-(5-chloro-2-iodobenzyl)-2-phenyl-1,2-dihydro-3*H*-pyrazol-3-one (1af): Pale yellow solid, mp = 212-213 ℃; Eluent: EtOAc; ^{1}H NMR (400 MHz, $CDCl_3$) δ 7.60 (d, *J* = 8.4 Hz, 1H), 7.43-7.38 (m, 2H), 7.33-7.29 (m, 3H), 7.27-7.19 (m, 6H), 6.91 (dd, *J* = 8.3, 2.0 Hz, 1H), 6.74 (s,

1H), 5.48 (s, 1H), 4.63 (s, 2H), 3.80 (s, 2H); ^{13}C NMR (100 MHz, $CDCl_3$) δ 166.29, 156.49, 140.46, 139.36, 135.35, 135.04, 134.23, 133.88, 129.50, 128.97, 128.68, 128.04, 127.45, 126.82, 126.02, 99.19, 93.49, 54.28, 33.39; HRMS (ESI$^+$): Calcd for $C_{23}H_{19}ClIN_2O$, $[M+H]^+$ *m/z* 501.0231. Found 501.0224.

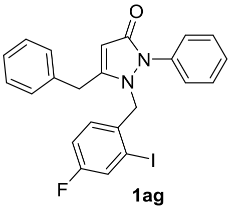

1ag

5-Benzyl-1-(4-fluoro-2-iodobenzyl)-2-phenyl-1,2-dihydro-3*H*-pyrazol-3-one (1ag): Pale yellow solid, mp = 152-153 ℃; Eluent: EtOAc; ^{1}H NMR (600 MHz, $CDCl_3$) δ 7.42 (dd, *J* = 7.7, 2.4 Hz, 1H), 7.38 (t, *J* = 7.7 Hz, 2H), 7.33-7.22 (m, 6H), 7.19 (d, *J* = 7.3 Hz, 2H), 7.01-6.91 (m, 1H), 6.75 (dd, *J* = 8.5, 5.7 Hz, 1H), 5.42 (s, 1H), 4.65 (s, 2H), 3.77 (s, 2H); ^{13}C NMR (150 MHz, $CDCl_3$) δ 166.13, 161.09 (d, $^1J_{C\text{-}F}$ = 252.7 Hz), 156.83, 135.04, 134.17, 133.47 (d, $^4J_{C\text{-}F}$ = 2.5 Hz), 129.28, 128.77, 128.59, 127.66, 127.24, 127.18, 126.31 (d, $^2J_{C\text{-}F}$ = 24.0 Hz), 125.60, 115.76 (d, $^2J_{C\text{-}F}$ = 21.3 Hz), 99.07, 95.79 (d, $^3J_{C\text{-}F}$ = 7.6 Hz), 53.70, 33.17; ^{19}F NMR (283 MHz, $CDCl_3$) δ -112.13; HRMS (ESI$^+$): Calcd for $C_{23}H_{19}FIN_2O$, $[M+H]^+$ *m/z* 485.0526. Found 485.0520.

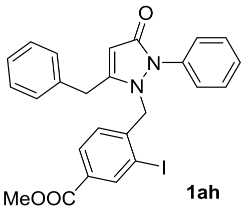

1ah

Methyl 4-((5-benzyl-3-oxo-2-phenyl-2,3-dihydro-1*H*-pyrazol-1-yl)methyl)-3-iodo-benzoate (1ah): Pale yellow solid, Mp = 189-190 ℃; Eluent: EtOAc; ^{1}H NMR (400 MHz, $CDCl_3$) δ 8.38 (d, *J* = 1.6 Hz, 1H), 7.90 (dd, *J* = 8.1, 1.6 Hz, 1H), 7.40 (t, *J* = 7.7 Hz, 2H), 7.33-7.29 (m, 3H), 7.28-7.23 (m, 3H), 7.19 (d, *J* = 6.9 Hz, 2H), 6.85 (d, *J* = 8.1 Hz, 1H), 5.47 (s, 1H), 4.70 (s, 2H), 3.91 (s, 3H), 3.77 (s, 2H); ^{13}C NMR (100 MHz, $CDCl_3$) δ 166.30, 165.09, 156.66, 142.73, 140.58, 135.07, 134.13, 131.20, 129.90, 129.61, 129.04, 128.80, 128.13, 127.54, 126.26, 125.97, 99.28, 95.92, 54.49, 52.62, 33.40; HRMS (ESI$^+$): Calcd for $C_{25}H_{22}IN_2O_3$, $[M+H]^+$ *m/z* 525.0675. Found 525.0665.

4. Synthesis and Characterization of 2a-ah

(1) General Procedures

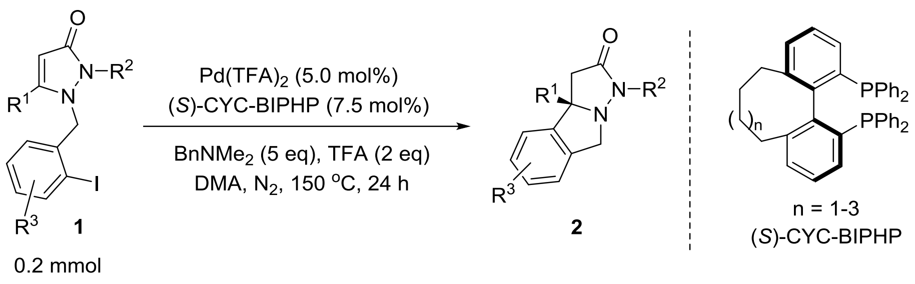

To a dried Schlenk tube were added $Pd(TFA)_2$ (3.32 mg, 0.01 mmol) and ligand (*S*)-**E**, (*S*)-**F** or (*S*)-**G** (0.015 mmol) under N_2, 3.0 mL of anhydrous *N*,*N*-dimethylacetamide (DMA) was then introduced via syringe. After stirring for 1 h, **1** (0.2 mmol, dissolved in 1 mL of DMA), $BnNMe_2$ (1.0 mmol, 5 equiv) and TFA (0.4 mmol, 2 equiv) were added via syringe. The mixture was vigorously stirred in a pre-warmed oil bath at 150 ℃ for 24 h. The solvent was then removed under vacuum, and the residue was purified by column chromatography on silica to give the desired product **2**. The enantiomeric excess was determined by chiral HPLC analysis.

(2) Preparation of Racemic Products *Rac*-2a-ah

Racemic products *Rac*-**2a-ah** were prepared according to the above procedures in the absence of ligand.

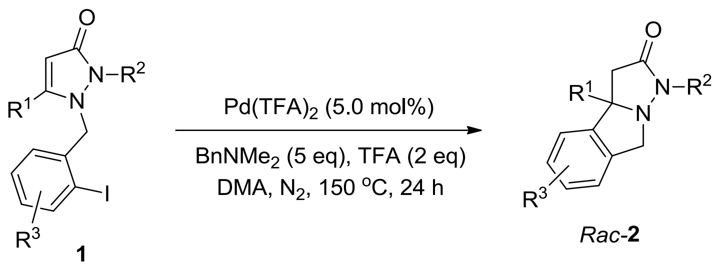

(3) Characterization of 2a-ah

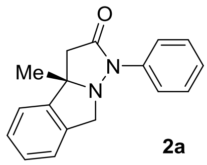

2a

(*S*)-3*a*-Methyl-1-phenyl-3*a*,8-dihydro-1*H*-pyrazolo[5,1-*a*]isoindol-2(3*H*)-one (2a): Pale yellow solid, mp = 127-128 ℃; Eluent: petroleum ether/EtOAc 4:1; Yield: 76%;

HPLC analysis: Daicel Chiralpak IB; hexane/*i*PrOH: 95:5; flow: 1.0 mL/min; λ = 220 nm. 25 ^{o}C. 97% ee (t_R (minor) = 9.5 min, t_R (major) = 28.9 min).

HRMS (ESI$^+$): Calcd for $C_{17}H_{17}N_2O$, $[M+H]^+$ *m/z* 265.1341. Found 265.1332.

^{1}H NMR (400 MHz, $CDCl_3$) δ 7.88 (d, *J* = 8.4 Hz, 2H), 7.42-7.35 (m, 2H), 7.35-7.27 (m, 2H), 7.26-7.18 (m, 2H), 7.15 (t, *J* = 7.4 Hz, 1H), 4.72 (d, *J* = 13.7 Hz, 1H), 4.07 (d, *J* = 13.7 Hz, 1H), 3.10 (d, *J* = 16.7 Hz, 1H), 2.66 (d, *J* = 16.8 Hz, 1H), 1.65 (s, 3H); ^{13}C NMR (100 MHz, $CDCl_3$) δ 170.35, 143.76, 138.53, 136.33, 128.97, 128.34, 128.20, 124.66, 123.37, 122.07, 119.48, 70.21, 60.11, 44.38, 25.99. IR (cm^{-1}): 3032 (w), 2966 (m), 2926 (m), 2845 (w), 1588 (vs), 1547 (m), 1482 (s), 1419 (m), 1353 (s), 1326 (m), 1306 (m), 1094 (m), 753 (vs), 816 (m), 733 (s), 694 (s).

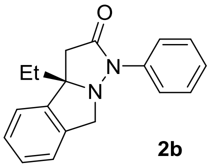

2b

(*S*)-3*a*-Ethyl-1-phenyl-3*a*,8-dihydro-1*H*-pyrazolo[5,1-*a*]isoindol-2(3*H*)-one (2b): Pale yellow solid, mp = 116-117 ℃; Eluent: petroleum ether/EtOAc 4:1; Yield: 74%;

HPLC analysis: Daicel Chiralpak IB; hexane/*i*PrOH: 95:5; flow: 1.0 mL/min; λ = 220 nm. 25 ^{o}C. 99% ee (t_R (minor) = 9.0 min, t_R (major) = 23.4 min).

HRMS (ESI$^+$): Calcd for $C_{18}H_{19}N_2O$, $[M+H]^+$ *m/z* 279.1497. Found 279.1492.

^{1}H NMR (400 MHz, $CDCl_3$) δ 7.89 (d, *J* = 8.2 Hz, 2H), 7.38 (t, *J* = 7.9 Hz, 2H), 7.34-7.26 (m, 2H), 7.22-7.11 (m, 3H), 4.72 (d, *J* = 13.9 Hz, 1H), 4.07 (d, *J* = 13.9 Hz, 1H), 3.11 (d, 1H), 2.67 (d, *J* = 18.2 Hz, 1H), 2.02-1.89 (m, 2H), 0.91 (t, *J* = 6.8 Hz, 3H); ^{13}C NMR (100 MHz, $CDCl_3$) δ 170.40, 142.39, 138.43, 136.98, 128.97, 128.23, 128.11, 124.59, 123.28, 122.22, 119.47, 73.33, 60.60, 43.86, 31.73, 8.33. IR (cm^{-1}): 3062 (w), 2969 (m), 2926 (m), 2851 (w), 1691 (vs), 1593 (m), 1485 (s), 1460 (m), 1414 (m), 1359 (s), 1327 (m), 1308 (m), 1097 (m), 758 (vs), 832 (m), 731 (s), 695 (s).

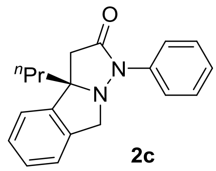

2c

(*S*)-1-Phenyl-3*a*-propyl-3*a*,8-dihydro-1*H*-pyrazolo[5,1-*a*]isoindol-2(3*H*)-one (2c): Light yellow oil; Eluent: petroleum ether/EtOAc 4:1; Yield: 79%;

HPLC analysis: Daicel Chiralpak IB; hexane/*i*PrOH: 90:10; flow: 1.0 mL/min; λ = 220 nm. 25 °C. 98% ee (t_R (minor) = 6.3 min, t_R (major) = 16.9 min).

HRMS (ESI$^+$): Calcd for $C_{19}H_{21}N_2O$, $[M+H]^+$ *m/z* 293.1654. Found 293.1651.

^{1}H NMR (400 MHz, $CDCl_3$) δ 7.88 (d, *J* = 8.5 Hz, 2H), 7.39 (t, *J* = 7.9 Hz, 2H), 7.35-7.27 (m, 2H), 7.23-7.12 (m, 3H), 4.72 (d, *J* = 14.0 Hz, 1H), 4.07 (d, *J* = 14.0 Hz, 1H), 3.11 (d, *J* = 16.9 Hz, 1H), 2.69 (d, *J* = 16.9 Hz, 1H), 1.99-1.82 (m, 2H), 1.60-1.50 (m, 1H), 1.26-1.15 (m, 1H), 0.90 (t, *J* = 7.4 Hz, 3H); ^{13}C NMR (100 MHz, $CDCl_3$) δ 170.36, 142.87, 138.41, 136.84, 129.00, 128.23, 128.12, 124.61, 123.29, 122.22, 119.51, 73.05, 60.58, 44.06, 41.52, 17.29, 14.49. IR (cm^{-1}): 3030 (w), 2957 (m), 2931 (w), 2871 (w), 1697 (vs), 1594 (s), 1542 (s), 1493 (m), 1459 (m), 1419 (w), 1353 (m), 1308 (m), 1028 (w), 751 (vs), 732 (m), 708 (s), 691 (w).

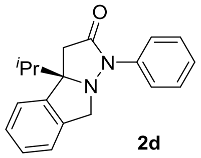

2d

(*R*)-3*a*-Isopropyl-1-phenyl-3*a*,8-dihydro-1*H*-pyrazolo[5,1-*a*]isoindol-2(3*H*)-one (2d): Pale yellow solid, mp = 103-104 ℃; Eluent: petroleum ether/EtOAc 4:1; Yield: 82%;

HPLC analysis: Daicel Chiralpak IB; hexane/*i*PrOH: 95:5; flow: 1.0 mL/min; λ = 220 nm. 25 °C. 98% ee (t_R (minor) = 8.8 min, t_R (major) = 20.0 min).

HRMS (ESI$^+$): Calcd for $C_{19}H_{21}N_2O$, $[M+H]^+$ *m/z* 293.1654. Found 293.1648.

^{1}H NMR (400 MHz, $CDCl_3$) δ 7.87 (d, *J* = 7.9 Hz, 2H), 7.40 (t, *J* = 7.9 Hz, 2H), 7.34-7.25 (m, 2H), 7.21-7.12 (m, 3H), 4.70 (d, *J* = 14.6 Hz, 1H), 4.06 (d, *J* = 14.6 Hz, 1H), 3.10 (d, *J* = 17.2 Hz, 1H), 2.90 (d, *J* = 17.3 Hz, 1H), 2.19 (hept, *J* = 6.7 Hz, 1H), 1.03 (d, *J* = 6.8 Hz, 3H), 0.89 (d, *J* = 6.7 Hz, 3H); ^{13}C NMR (100 MHz, $CDCl_3$) δ 169.75, 143.38, 137.99, 137.04, 128.96, 128.07, 124.66, 123.12, 122.46, 119.73, 75.77, 61.47, 42.92, 36.58, 17.73, 16.56. IR (cm^{-1}): 3057 (w), 2964 (m), 2923 (w), 2871 (w), 1690 (vs), 1591 (m), 1487 (m), 1353 (s), 1307(w), 1070 (m), 768 (vs), 730 (m), 693 (s).

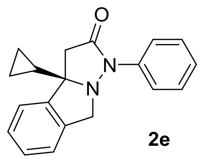

2e

(*R*)-3*a*-Cyclopropyl-1-phenyl-3*a*,8-dihydro-1*H*-pyrazolo[5,1-*a*]isoindol-2(3*H*)-one (2e): Pale yellow solid, mp = 163-164 ℃; Eluent: petroleum ether/EtOAc 4:1; Yield: 81%;

HPLC analysis: Daicel Chiralpak IB; hexane/*i*PrOH: 95:5; flow: 1.0 mL/min; λ = 220 nm. 25 °C. 98% ee (t_R (minor) = 9.7 min, t_R (major) = 27.7 min).

HRMS (ESI$^+$): Calcd for $C_{19}H_{19}N_2O$, $[M+H]^+$ *m*/*z* 291.1497. Found 291.1391.

^1H NMR (400 MHz, $CDCl_3$) δ 7.82 (d, *J* = 7.8 Hz, 2H), 7.38 (t, *J* = 8.0 Hz, 2H), 7.35-7.23 (m, 3H), 7.20-7.10 (m, 2H), 4.67 (d, *J* = 14.0 Hz, 1H), 4.04 (d, *J* = 14.0 Hz, 1H), 3.14 (d, *J* = 16.9 Hz, 1H), 2.80 (d, *J* = 16.9 Hz, 1H), 1.40-1.32 (m, 1H), 0.56-0.35 (m, 4H); ^{13}C NMR (100 MHz, $CDCl_3$) δ 170.17, 143.92, 137.97, 136.32, 129.04, 128.28, 128.22, 124.63, 123.10, 122.48, 119.38, 71.85, 60.75, 43.43, 19.31, 0.85, 0.35. IR (cm^{-1}): 3013 (w), 2923 (w), 2853 (w), 1691 (vs), 1485 (m), 1458 (s), 1413 (w), 1351 (m), 1048 (w), 1025 (m), 1308 (m), 758 (vs), 732 (m), 731 (s), 693 (s).

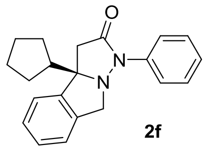

2f

(*R*)-3*a*-Cyclopentyl-1-phenyl-3*a*,8-dihydro-1*H*-pyrazolo[5,1-*a*]isoindol-2(3*H*)-one (2f): Pale yellow solid, mp = 97-98 ℃; Eluent: petroleum ether/EtOAc 4:1; Yield: 83%;

HPLC analysis of **2f**: Daicel Chiralpak IB; hexane/*i*PrOH: 85:15; flow: 1.0 mL/min; λ = 220 nm. 25 °C. 98% ee (t_R (minor) = 5.8 min, t_R (major) = 11.4 min)

HRMS (ESI$^+$): Calcd for $C_{21}H_{23}N_2O$, $[M+H]^+$ *m*/*z* 319.1810. Found 319.1804.

^1H NMR (400 MHz, $CDCl_3$) δ 7.87 (d, *J* = 7.9 Hz, 2H), 7.39 (t, *J* = 7.9 Hz, 2H), 7.32-7.20 (m, 3H), 7.18-7.11 (m, 2H), 4.71 (d, *J* = 14.5 Hz, 1H), 4.06 (d, *J* = 14.5 Hz, 1H), 3.13 (d, *J* = 17.2 Hz, 1H), 2.89 (d, *J* = 17.2 Hz, 1H), 2.46 (quint, *J* = 8.8, 8.2 Hz, 1H), 1.86-1.77 (m, 1H), 1.71-1.49 (m, 4H), 1.47-1.33 (m, 3H); ^{13}C NMR (100 MHz, $CDCl_3$) δ 169.73, 143.98, 138.01, 136.78, 128.97, 128.11, 128.05, 124.62, 123.07, 122.45, 119.68, 74.27, 61.47, 49.52, 43.63, 27.54, 26.79, 26.05, 25.22. IR (cm^{-1}): 3029 (w), 2949 (m), 2855 (w), 1694 (vs), 1592 (m), 1486 (s), 1457 (w), 1418 (m), 1306 (s), 1306 (m), 1075 (m), 1028 (m), 757 (vs), 731 (m), 692 (s).

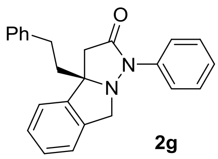

2g

(*S*)-3*a*-Phenethyl-1-phenyl-3*a*,8-dihydro-1*H*-pyrazolo[5,1-*a*]isoindol-2(3*H*)-one (2g): Light yellow oil; Eluent: petroleum ether/EtOAc 4:1; Yield: 82%;

HPLC analysis: Daicel Chiralpak IB; hexane/*i*PrOH: 95:5; flow: 1.0 mL/min; λ = 220 nm. 25 °C. 98% ee (t_R (minor) = 12.0 min, t_R (major) = 29.0 min).

HRMS (ESI$^+$): Calcd for $C_{24}H_{23}N_2O$, $[M+H]^+$ *m/z* 355.1810. Found 355.1805.

^1H NMR (600 MHz, $CDCl_3$) δ 7.89 (d, *J* = 8.7 Hz, 2H), 7.39 (t, *J* = 8.0 Hz, 2H), 7.36-7.28 (m, 2H), 7.26-7.19 (m, 4H), 7.18-7.12 (m, 2H), 7.10 (d, *J* = 8.0 Hz, 2H), 4.77 (d, *J* = 13.8 Hz, 1H), 4.09 (d, *J* = 13.7 Hz, 1H), 3.15 (d, *J* = 17.0 Hz, 1H), 2.90-2.80 (m, 1H), 2.70 (d, *J* = 16.9 Hz, 1H), 2.49-2.39 (m, 1H), 2.32-2.24 (m, 1H), 2.24-2.13 (m, 1H); ^{13}C NMR (100 MHz, $CDCl_3$) δ 170.20, 142.07, 141.94, 138.41, 136.95, 129.07, 128.49, 128.45, 128.38, 128.33, 125.92, 124.67, 123.44, 122.17, 119.39, 72.89, 60.66, 44.22, 40.74, 30.55. IR (cm^{-1}): 3026 (w), 2927 (w), 2854 (w), 1696 (vs), 1594 (m), 1491 (s), 1457 (m), 1352 (s), 1309 (s), 1028 (w), 754 (vs), 729 (m), 695 (s) , 620 (w).

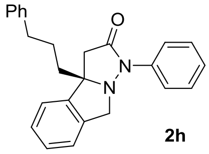

2h

(*S*)-1-Phenyl-3*a*-(3-phenylpropyl)-3*a*,8-dihydro-1*H*-pyrazolo[5,1-*a*]isoindol-2(3*H*)-one (2h): Light yellow oil; Eluent: petroleum ether/EtOAc 4:1; Yield: 83%;

HPLC analysis of **2h**: Daicel Chiralpak IB; hexane/*i*PrOH: 90:10; flow: 1.0 mL/min; λ = 220 nm. 25 °C. 97% ee (t_R (minor) = 7.9 min, t_R (major) = 15.4 min).

HRMS (ESI$^+$): Calcd for $C_{25}H_{25}N_2O$, $[M+H]^+$ *m/z* 369.1967. Found 369.1962.

^1H NMR (400 MHz, $CDCl_3$) δ 7.86 (d, *J* = 8.1 Hz, 2H), 7.39 (t, *J* = 7.9 Hz, 2H), 7.32-7.12 (m, 7H), 7.09 (d, *J* = 6.9 Hz, 3H), 4.71 (d, *J* = 13.9 Hz, 1H), 4.06 (d, *J* = 13.9 Hz, 1H), 3.09 (d, *J* = 16.9 Hz, 1H), 2.71-2.52 (m, 3H), 2.03-1.84 (m, 3H), 1.56-1.41 (m, 1H); ^{13}C NMR (100 MHz, $CDCl_3$) δ 170.35, 142.38, 142.03, 138.43, 136.91, 129.05, 128.49, 128.41, 128.32, 128.19, 125.92, 124.67, 123.34, 122.22, 119.51, 73.04, 60.59, 44.24, 38.22, 35.90, 25.61. IR (cm^{-1}): 3026 (w),

2933 (w), 2852 (w), 1696 (vs), 1594 (m), 1491 (s), 1457 (m), 1353 (s), 1309 (s), 1028 (m), 750 (vs), 729 (m), 694 (s) , 620 (w).

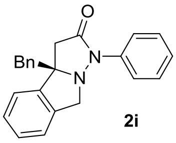

2i

(*S*)-3*a*-Benzyl-1-phenyl-3*a*,8-dihydro-1*H*-pyrazolo[5,1-*a*]isoindol-2(3*H*)-one (2i): Pale yellow solid, mp = 140-141 ℃; Eluent: petroleum ether/EtOAc 4:1; Yield: 80%;

HPLC analysis: Daicel Chiralpak IB; hexane/*i*PrOH: 95:5; flow: 1.0 mL/min; λ = 220 nm. 25 ^{o}C. 98% ee (t_R (minor) = 14.3 min, t_R (major) = 26.9 min).

HRMS (ESI$^+$): Calcd for $C_{23}H_{21}N_2O$, $[M+H]^+$ *m*/*z* 341.1654. Found 341.1648.

^{1}H NMR (400 MHz, $CDCl_3$) δ 7.79 (d, *J* = 8.7 Hz, 2H), 7.40-7.22 (m, 5H), 7.20-7.08 (m, 7H), 4.50 (d, *J* = 14.5 Hz, 1H), 4.00 (d, *J* = 14.4 Hz, 1H), 3.25-3.16 (m, 2H), 3.12 (d, *J* = 17.0 Hz, 1H), 2.92 (d, *J* = 17.0 Hz, 1H); ^{13}C NMR (100 MHz, $CDCl_3$) δ 169.15, 142.98, 138.03, 137.07, 135.76, 130.70, 128.89, 128.42, 128.01, 127.87, 126.81, 124.63, 123.30, 122.71, 119.65, 73.64, 60.36, 45.40, 43.65. IR (cm^{-1}): 3071 (w), 3026 (w),2927 (w), 2853 (w), 1682 (vs), 1591 (m), 1486 (s), 1456 (m), 1363 (s), 1312 (m), 1025 (m), 992 (w), 770 (m), 749 (s), 731 (m), 695 (vs) , 660 (m).

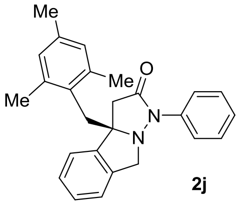

2j

(*S*)-1-Phenyl-3*a*-(2,4,6-trimethylbenzyl)-3*a*,8-dihydro-1*H*-pyrazolo[5,1-*a*]isoindol-2(3*H*)-one (2j): Light yellow oil; Eluent: petroleum ether/EtOAc 4:1; Yield: 78%;

HPLC analysis: Daicel Chiralpak IB; hexane/*i*PrOH: 95:5; flow: 1.0 mL/min; λ = 220 nm. 25 ^{o}C. 98% ee (t_R (minor) = 11.7 min, t_R (major) = 13.2 min).

HRMS (ESI$^+$): Calcd for $C_{26}H_{27}N_2O$, $[M+H]^+$ *m*/*z* 383.2123. Found 383.2118.

^{1}H NMR (600 MHz, $CDCl_3$) δ 7.50 (d, *J* = 8.4 Hz, 2H), 7.37-7.29 (m, 5H), 7.18-7.11 (m, 2H), 6.83 (s, 2H), 4.31 (d, *J* = 16.1 Hz, 1H), 4.08 (d, *J* = 16.1 Hz, 1H), 3.29 (d, *J* = 14.9 Hz, 1H), 3.11 (d, *J* = 4.7 Hz, 1H), 3.09 (d, *J* = 2.8 Hz, 1H), 2.98 (d, *J* = 16.7 Hz, 1H), 2.31 (s, 6H), 2.23 (s, 3H); ^{13}C NMR (100 MHz, $CDCl_3$) δ 167.93, 146.30, 138.02, 137.67, 137.13, 136.36, 130.50, 129.38, 128.87, 128.65, 128.24, 125.42, 123.33, 123.00, 121.67, 75.80, 58.80, 42.17, 37.80, 21.15, 20.92.

IR (cm^{-1}): 2951 (w), 2918 (w), 2858 (w), 1698 (vs), 1593 (m), 1490 (s), 1457 (m), 1351 (s), 1307 (s), 1026 (w), 989 (w), 851 (s), 754 (vs), 692 (s) , 626 (w).

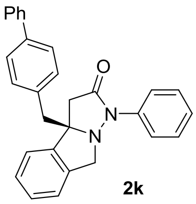

2k

(*S*)-3*a*-([1,1'-Biphenyl]-4-ylmethyl)-1-phenyl-3*a*,8-dihydro-1*H*-pyrazolo[5,1-*a*]isoindol-2(3*H*)-one (2k): Light yellow oil; Eluent: petroleum ether/EtOAc 4:1; Yield: 88%;

HPLC analysis: Daicel Chiralpak ID; hexane/*i*PrOH: 97:3; flow: 1.0 mL/min; λ = 220 nm. 25 °C. 99% ee (t_R (major) = 46.4 min, t_R (minor) = 52.7 min).

HRMS (ESI^+): Calcd for $C_{29}H_{25}N_2O$, $[M+H]^+$ *m/z* 417.1967. Found 417.1958.

^1H NMR (400 MHz, $CDCl_3$) δ 7.80 (d, *J* = 8.5 Hz, 2H), 7.47 (d, *J* = 8.1 Hz, 2H), 7.40-7.19 (m, 12H), 7.17-7.06 (m, 2H), 4.52 (d, *J* = 14.4 Hz, 1H), 4.00 (d, *J* = 14.4 Hz, 1H), 3.29-3.17 (m, 2H), 3.12 (d, *J* = 17.0 Hz, 1H), 2.93 (d, *J* = 17.1 Hz, 1H); ^{13}C NMR (100 MHz, $CDCl_3$) δ 169.25, 142.88, 140.84, 139.47, 138.11, 137.09, 134.91, 131.10, 128.89, 128.72, 128.43, 128.01, 127.16, 127.01, 126.51, 124.62, 123.33, 122.69, 119.57, 73.72, 60.38, 44.88, 43.76. IR (cm^{-1}): 3028 (w), 2916 (w), 1696 (vs), 1594 (m), 1489 (s), 1458 (m), 1355 (s), 1309 (s), 1074 (w), 847 (w), 821 (vs), 757 (s) , 693 (w).

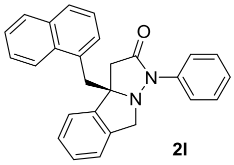

2l

(*S*)-3*a*-(Naphthalen-1-ylmethyl)-1-phenyl-3*a*,8-dihydro-1*H*-pyrazolo[5,1-*a*]isoindol-2(3*H*)-one (2l): Light yellow oil; Eluent: petroleum ether/EtOAc 4:1; Yield: 84%;

HPLC analysis: Daicel Chiralpak IB; hexane/*i*PrOH: 95:5; flow: 1.0 mL/min; λ = 220 nm. 25 °C. 98% ee (t_R (minor) = 24.2 min, t_R (major) = 28.7 min).

HRMS (ESI^+): Calcd for $C_{27}H_{23}N_2O$, $[M+H]^+$ *m/z* 391.1810. Found 391.1809.

^1H NMR (600 MHz, $CDCl_3$) δ 8.15 (d, *J* = 8.2 Hz, 1H), 7.74 (d, *J* = 7.7 Hz, 1H), 7.64 (d, *J* = 8.1 Hz, 1H), 7.50 (d, *J* = 8.0 Hz, 2H), 7.41-7.31 (m, 4H), 7.28-7.19 (m, 5H), 7.09-7.03 (m, 2H), 4.31 (d, *J* = 14.8 Hz, 1H), 3.92 (d, *J* = 14.9 Hz, 1H), 3.81 (d, *J* = 14.4 Hz, 1H), 3.60 (d, *J* = 14.4 Hz, 1H), 3.15 (d, *J* = 17.0 Hz, 1H), 3.07 (d, *J* = 17.0 Hz, 1H); ^{13}C NMR (100 MHz, $CDCl_3$) δ 168.64,

143.79, 137.53, 137.15, 133.80, 133.16, 132.21, 128.75, 128.64, 128.51, 128.09, 127.71, 125.73, 125.43, 124.96, 124.90, 124.63, 123.28, 122.88, 120.05, 77.48, 77.16, 76.84, 74.30, 60.25, 43.66, 41.35. IR (cm^{-1}): 3036 (w), 2914 (w), 2830 (w), 1689 (vs), 1594 (m), 1490 (s), 1457 (m), 1395 (s), 1326 (s), 1062 (w), 802 (w), 776 (s), 745 (vs), 689 (s) , 625 (w).

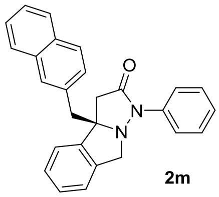

2m

(*S*)-3*a*-(Naphthalen-2-ylmethyl)-1-phenyl-3*a*,8-dihydro-1*H*-pyrazolo[5,1-*a*]isoindol-2(3*H*)-one (2m): Pale yellow solid, mp = 116-118 ℃; Eluent: petroleum ether/EtOAc 4:1; Yield: 85%; HPLC analysis: Daicel Chiralpak IB; hexane/*i*PrOH: 95:5; flow: 1.0 mL/min; λ = 220 nm. 25 °C. 98% ee (t_R (minor) = 19.2 min, t_R (major) = 32.0 min).

HRMS (ESI$^+$): Calcd for $C_{27}H_{23}N_2O$, $[M+H]^+$ *m/z* 391.1810. Found 391.1807.

^{1}H NMR (600 MHz, $CDCl_3$) δ 7.80 (d, *J* = 7.7 Hz, 2H), 7.73-7.70 (m, 1H), 7.63-7.58 (m, 3H), 7.39-7.32 (m, 6H), 7.30-7.23 (m, 2H), 7.14 (t, *J* = 7.4 Hz, 1H), 7.07 (d, *J* = 7.5 Hz, 1H), 4.48 (d, *J* = 14.4 Hz, 1H), 4.00 (d, *J* = 14.4 Hz, 1H), 3.42-3.32 (m, 2H), 3.15 (d, *J* = 16.9 Hz, 1H), 2.98 (d, *J* = 16.9 Hz, 1H); ^{13}C NMR (100 MHz, $CDCl_3$) δ 169.32, 142.72, 138.12, 137.11, 133.48, 133.14, 132.33, 129.66, 129.05, 128.93, 128.47, 128.01, 127.66, 127.58, 127.24, 125.84, 125.54, 124.67, 123.35, 122.76, 119.57, 73.87, 60.36, 45.27, 43.94. IR (cm^{-1}): 3049 (w), 2918 (w), 2849 (w), 1689 (vs), 1593 (m), 1490 (s), 1458 (m), 1356 (s), 1306 (s), 1063 (w), 818 (w), 747 (vs), 689 (m) , 633 (w).

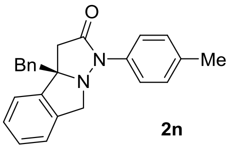

2n

(*S*)-3*a*-Benzyl-1-(*p*-tolyl)-3*a*,8-dihydro-1*H*-pyrazolo[5,1-*a*]isoindol-2(3*H*)-one (2n): Pale yellow solid, mp = 128-129 ℃; Eluent: petroleum ether/EtOAc 4:1; Yield: 78%;

HPLC analysis: Daicel Chiralpak IB; hexane/*i*PrOH: 95:5; flow: 1.0 mL/min; λ = 220 nm. 25 °C. 98% ee (t_R (minor) = 13.9 min, t_R (major) = 22.3 min).

HRMS (ESI$^+$): Calcd for $C_{24}H_{23}N_2O$, $[M+H]^+$ *m/z* 355.1810. Found 355.1802.

^{1}H NMR (400 MHz, $CDCl_3$) δ 7.65 (d, *J* = 8.4 Hz, 2H), 7.35-7.24 (m, 3H), 7.22-7.07 (m, 8H), 4.47 (d, *J* = 14.6 Hz, 1H), 4.00 (d, *J* = 14.5 Hz, 1H), 3.25-3.16 (m, 2H), 3.11 (d, *J* = 16.9 Hz, 1H), 2.92 (d, *J* = 16.9 Hz, 1H), 2.35 (s, 3H); ^{13}C NMR (100 MHz, $CDCl_3$) δ 168.78, 143.13, 137.22, 135.90, 135.56, 134.46, 130.77, 129.48, 128.43, 128.00, 127.90, 126.82, 123.32, 122.79, 119.95, 73.75, 60.27, 45.43, 43.67, 21.06. IR (cm^{-1}): 3051 (w), 2913 (w), 2846 (w), 1686 (vs), 1593 (m), 1491 (s), 1455 (m), 1354 (s), 1306 (s), 1064 (w), 819 (w), 744 (vs), 686 (m).

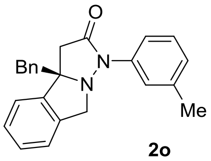

2o

(*S*)-3*a*-Benzyl-1-(*m*-tolyl)-3*a*,8-dihydro-1*H*-pyrazolo[5,1-*a*]isoindol-2(3*H*)-one (2o): Light yellow oil; Eluent: petroleum ether/EtOAc 4:1; Yield: 77%;

HPLC analysis: Daicel Chiralpak IB; hexane/*i*PrOH: 95:5; flow: 1.0 mL/min; λ = 220 nm. 25 °C. 98% ee (t_R (minor) = 12.5 min, t_R (major) = 22.7 min).

HRMS (ESI$^+$): Calcd for $C_{24}H_{23}N_2O$, $[M+H]^+$ *m/z* 355.1810. Found 355.1806.

^{1}H NMR (400 MHz, $CDCl_3$) δ 7.62 (s, 1H), 7.57 (d, *J* = 8.2 Hz, 1H), 7.35-7.30 (m, 1H), 7.29-7.23 (m, 3H), 7.19-7.09 (m, 6H), 6.97 (d, *J* = 7.6 Hz, 1H), 4.50 (d, *J* = 14.5 Hz, 1H), 4.00 (d, *J* = 14.5 Hz, 1H), 3.25-3.16 (m, 2H), 3.11 (d, *J* = 16.9 Hz, 1H), 2.92 (d, *J* = 17.0 Hz, 1H), 2.37 (s, 3H); ^{13}C NMR (100 MHz, $CDCl_3$) δ 169.10, 143.01, 138.76, 137.97, 137.17, 135.86, 130.78, 128.75, 128.42, 127.99, 127.87, 126.81, 125.57, 123.32, 122.77, 120.51, 116.99, 73.67, 60.32, 45.34, 43.73, 21.79. IR (cm^{-1}): 3062 (w), 3030 (w), 2915 (m), 2856 (w), 1684 (vs), 1604 (m), 1584 (s), 1489 (m), 1364 (s), 1204 (s), 779 (w), 753 (s), 729 (s), 695 (vs), 664 (s).

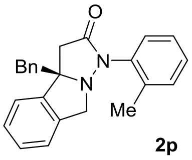

2p

(*S*)-3*a*-Benzyl-1-(*o*-tolyl)-3*a*,8-dihydro-1*H*-pyrazolo[5,1-*a*]isoindol-2(3*H*)-one (2p): Pale yellow solid, mp = 133-134 ℃; Eluent: petroleum ether/EtOAc 4:1; Yield: 75%;

HPLC analysis: Daicel Chiralpak IB; hexane/*i*PrOH: 98:2; flow: 1.0 mL/min; λ = 220 nm. 25 °C. >99% ee (t_R (major) = 36.0 min, t_R (minor) = 42.8 min).

HRMS (ESI$^+$): Calcd for $C_{24}H_{23}N_2O$, $[M+H]^+$ *m/z* 355.1810. Found 355.1802.

^{1}H NMR (600 MHz, $CDCl_3$) δ 7.35 (d, *J* = 7.0 Hz, 1H), 7.33-7.20 (m, 8H), 7.18-7.12 (m, 3H), 7.10 (d, *J* = 7.1 Hz, 1H), 3.92 (s, 2H), 3.20 (d, *J* = 13.8 Hz, 1H), 3.14-3.00 (m, 3H), 2.06 (s, 3H); ^{13}C NMR (100 MHz, $CDCl_3$) δ 167.22, 144.88, 138.26, 136.89, 136.16, 135.50, 131.13, 130.85, 128.65, 128.38, 128.09, 127.80, 127.34, 126.94, 126.71, 123.36, 123.20, 76.04, 58.01, 45.05, 42.13, 18.28. IR (cm^{-1}): 3028 (w), 2969 (m), 2920 (w), 1685 (vs), 1601 (w), 1582 (w), 1488 (m), 1447 (s), 1369 (s), 779 (w), 1072 (w), 765 (s), 721 (s), 699 (vs), 660 (s), 638 (s).

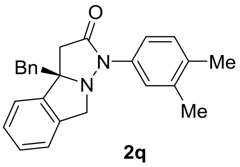

2q

(*S*)-3*a*-Benzyl-1-(3,4-dimethylphenyl)-3*a*,8-dihydro-1*H*-pyrazolo[5,1-*a*]isoindol-2(3*H*)-one (2q): Pale yellow solid, mp = 110-111 ℃; Eluent: petroleum ether/EtOAc 4:1; Yield: 78%; HPLC analysis: Daicel Chiralpak IB; hexane/*i*PrOH: 95:5; flow: 1.0 mL/min; λ = 220 nm. 25 °C. 98% ee (t_R (minor) = 13.4 min, t_R (major) = 20.5 min).

HRMS (ESI^+): Calcd for $C_{25}H_{25}N_2O$, $[M+H]^+$ *m/z* 369.1967. Found 369.1961.

^{1}H NMR (600 MHz, $CDCl_3$) δ 7.56 (s, 1H), 7.47 (d, *J* = 8.2 Hz, 1H), 7.32 (t, *J* = 7.4 Hz, 1H), 7.29-7.24 (m, 2H), 7.19-7.12 (m, 6H), 7.09 (d, *J* = 7.9 Hz, 1H), 4.45 (d, *J* = 14.5 Hz, 1H), 4.00 (d, *J* = 14.6 Hz, 14H), 3.26-3.15 (m, 2H), 3.10 (d, *J* = 16.9 Hz, 1H), 2.92 (d, *J* = 16.9 Hz, 1H), 2.28 (s, 3H), 2.25 (s, 3H); ^{13}C NMR (100 MHz, $CDCl_3$) δ 168.70, 143.11, 137.29, 137.17, 135.96, 135.76, 133.31, 130.82, 129.97, 128.38, 127.93, 127.86, 126.79, 123.31, 122.82, 121.44, 117.67, 73.74, 60.18, 45.33, 43.69, 20.19, 19.39. IR (cm^{-1}): 3030 (w), 2967 (m), 2917 (w), 2861 (w), 1694 (vs), 1609 (w), 1576 (w), 1498 (m), 1358 (s), 895 (w), 876 (w), 761 (s), 727 (s), 693 (m), 675 (s), 615 (w).

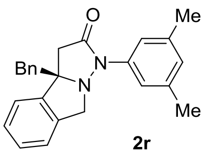

2r

(*S*)-3*a*-Benzyl-1-(3,5-dimethylphenyl)-3*a*,8-dihydro-1*H*-pyrazolo[5,1-*a*]isoindol-2(3*H*)-one (2r): Pale yellow solid, Mp = 113-114 ℃; Eluent: petroleum ether/EtOAc 4:1; Yield: 79%; HPLC analysis: Daicel Chiralpak IB; hexane/*i*PrOH: 95:5; flow: 1.0 mL/min; λ = 220 nm. 25 °C. 98% ee (t_R (minor) = 11.9 min, t_R (major) = 18.5 min).

HRMS (ESI$^+$): Calcd for $C_{25}H_{25}N_2O$, $[M+H]^+$ *m/z* 369.1967. Found 369.1960.

^{1}H NMR (400 MHz, $CDCl_3$) δ 7.40 (s, 2H), 7.34-7.29 (m, 1H), 7.28-7.23 (m, 2H), 7.18-7.12 (m, 5H), 7.10 (d, *J* = 7.9 Hz, 1H), 6.80 (s, 1H), 4.48 (d, *J* = 14.5 Hz, 1H), 3.99 (d, *J* = 14.5 Hz, 1H), 3.26-3.14 (m, 2H), 3.10 (d, *J* = 16.9 Hz, 1H), 2.91 (d, *J* = 16.9 Hz, 1H), 2.33 (s, 6H); ^{13}C NMR (100 MHz, $CDCl_3$) δ 169.04, 142.96, 138.55, 137.86, 137.22, 135.91, 130.83, 128.37, 127.92, 127.82, 126.78, 126.60, 123.31, 122.79, 117.81, 73.66, 60.21, 45.22, 43.75, 21.67. IR (cm^{-1}): 3059 (w), 3031 (w), 2932 (m), 2860 (w), 1683 (vs), 1591 (w), 1459 (w), 1363 (s), 1289 (m), 1066 (w), 848 (m), 753 (w), 730 (s), 697 (vs), 665 (s).

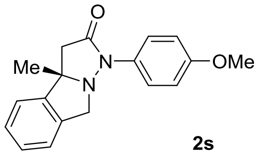

2s

(*S*)-1-(4-Methoxyphenyl)-3*a*-methyl-3*a*,8-dihydro-1*H*-pyrazolo[5,1-*a*]isoindol-2(3*H*)-one (2s): Light yellow oil; Eluent: petroleum ether/EtOAc 4:1; Yield: 74%;

HPLC analysis: Daicel Chiralpak IB; hexane/*i*PrOH: 95:5; flow: 1.0 mL/min; λ = 220 nm. 25 °C. 98% ee (t_R (minor) = 16.9 min, t_R (major) = 29.9 min).

HRMS (ESI$^+$): Calcd for $C_{18}H_{19}N_2O_2$, $[M+H]^+$ *m/z* 295.1447. Found 295.1441.

^{1}H NMR (400 MHz, $CDCl_3$) δ 7.74 (d, *J* = 9.1 Hz, 2H), 7.35-7.28 (m, 2H), 7.24 (t, *J* = 5.8 Hz, 1H), 7.20 (d, *J* = 7.0 Hz, 1H), 6.93 (d, *J* = 9.0 Hz, 2H), 4.66 (d, *J* = 13.8 Hz, 1H), 4.08 (d, *J* = 13.8 Hz, 1H), 3.81 (s, 3H), 3.10 (d, *J* = 16.8 Hz, 1H), 2.66 (d, *J* = 16.6 Hz, 1H), 1.65 (s, 3H); ^{13}C NMR (100 MHz, $CDCl_3$) δ 169.63, 156.92, 144.01, 136.48, 131.70, 128.35, 128.23, 123.40, 122.15, 121.72, 114.22, 70.38, 60.10, 55.64, 44.29, 26.14. IR (cm^{-1}): 2957 (w), 2924 (m), 2836 (w), 1689 (vs), 1608 (w), 1585 (w), 1505 (s), 1357 (s), 1296 (m), 1243 (vs), 1065 (s), 795 (s), 762 (s), 729 (m), 660 (w).

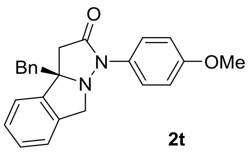

2t

(*S*)-3*a*-Benzyl-1-(4-methoxyphenyl)-3*a*,8-dihydro-1*H*-pyrazolo[5,1-*a*]isoindol-2(3*H*)-one (2t): Pale yellow solid, mp = 191-193 ℃; Eluent: petroleum ether/EtOAc 4:1; Yield: 75%;

HPLC analysis: Daicel Chiralpak IB; hexane/*i*PrOH: 95:5; flow: 1.0 mL/min; λ = 220 nm. 25 °C. 98% ee (t_R (minor) = 27.5 min, t_R (major) = 32.2 min).

HRMS (ESI^+): Calcd for $C_{24}H_{23}N_2O_2$, $[M+H]^+$ *m/z* 371.1760. Found 371.1750.

^1H NMR (600 MHz, $CDCl_3$) δ 7.65 (d, *J* = 9.1 Hz, 2H), 7.33 (t, *J* = 7.5 Hz, 1H), 7.28-7.24 (m, 2H), 7.19-7.12 (m, 5H), 7.10 (d, *J* = 7.4 Hz, 1H), 6.91 (d, *J* = 9.1 Hz, 2H), 4.41 (d, *J* = 14.6 Hz, 1H), 4.00 (d, *J* = 14.6 Hz, 1H), 3.81 (s, 3H), 3.25-3.15 (m, 2H), 3.10 (d, *J* = 16.9 Hz, 1H), 2.93 (d, *J* = 16.9 Hz, 1H); ^{13}C NMR (100 MHz, $CDCl_3$) δ 168.33, 156.85, 143.28, 137.25, 135.89, 131.19, 130.72, 128.42, 127.99, 127.90, 126.82, 123.30, 122.80, 121.86, 114.13, 73.84, 60.11, 55.59, 45.47, 43.47. IR (cm^{-1}): 3022 (w), 2955 (m), 2923 (w), 2852 (w), 1675 (vs), 1602 (w), 1544 (w), 1502 (s), 1373 (s), 1243 (s), 837 (s), 755 (m), 729 (m), 697 (vs), 665 (w).

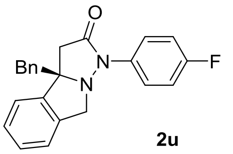

2u

(*S*)-3*a*-Benzyl-1-(4-fluorophenyl)-3a,8-dihydro-1*H*-pyrazolo[5,1-*a*]isoindol-2(3*H*)-one (2u): Pale yellow solid, mp = 119-120 ℃; Eluent: petroleum ether/EtOAc 4:1; Yield: 81%;

HPLC analysis: Daicel Chiralpak IB; hexane/*i*PrOH: 95:5; flow: 1.0 mL/min; λ = 220 nm. 25 °C. 98% ee (t_R (minor) = 14.8 min, t_R (major) = 29.1 min).

HRMS (ESI^+): Calcd for $C_{23}H_{20}FN_2O$, $[M+H]^+$ *m/z* 359.1560. Found 359.1553.

^1H NMR (600 MHz, $CDCl_3$) δ 7.80-7.68 (m, 2H), 7.34 (t, *J* = 7.4 Hz, 1H), 7.28 (t, *J* = 6.7 Hz, 2H), 7.17-7.10 (m, 6H), 7.08-7.03 (m, 2H), 4.49 (d, *J* = 14.4 Hz, 1H), 3.98 (d, *J* = 14.4 Hz, 1H), 3.26-3.15 (m, 2H), 3.11 (d, *J* = 17.0 Hz, 1H), 2.93 (d, *J* = 17.0 Hz, 1H); ^{13}C NMR (100 MHz, $CDCl_3$) δ 168.97, 159.56 (d, $^1J_{C\text{-}F}$ = 244.1 Hz), 142.97, 136.88, 135.63, 134.14 (d, $^4J_{C\text{-}F}$ = 2.5 Hz), 130.58, 128.49, 128.10, 127.90, 126.88, 123.31, 122.69, 121.30 (d, $^3J_{C\text{-}F}$ = 7.8 Hz), 115.53 (d, $^2J_{C\text{-}F}$ = 22.6 Hz), 73.72, 60.29, 45.45, 43.38; ^{19}F NMR (283 MHz, $CDCl_3$) δ -117.47. IR (cm^{-1}): 3084 (w), 3027 (m), 3006 (w), 2924 (w), 2855 (w), 1681 (vs), 1602 (w), 1503 (s), 1456 (m), 1421 (s), 1236 (m), 907 (m), 755 (w), 729 (s), 697 (s), 663 (w).

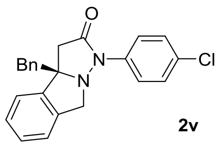

2v

(*S*)-3*a*-Benzyl-1-(4-chlorophenyl)-3*a*,8-dihydro-1*H*-pyrazolo[5,1-*a*]isoindol-2(3*H*)-one (2v): Pale yellow solid, mp = 147-148 ℃; Eluent: petroleum ether/EtOAc 4:1; Yield: 81%;

HPLC analysis: Daicel Chiralpak IB; hexane/*i*PrOH: 95:5; flow: 1.0 mL/min; λ = 220 nm. 25 °C. 98% ee (t_R (minor) = 14.7 min, t_R (major) = 31.4 min).

HRMS (ESI$^+$): Calcd for $C_{23}H_{20}ClN_2O$, $[M+H]^+$ *m/z* 375.1264. Found 375.1261.

^1H NMR (600 MHz, $CDCl_3$) δ 7.73 (d, *J* = 8.9 Hz, 2H), 7.38-7.27 (m, 5H), 7.18-7.09 (m, 6H), 4.55 (d, *J* = 14.2 Hz, 1H), 3.98 (d, *J* = 14.2 Hz, 1H), 3.28-3.15 (m, 2H), 3.11 (d, *J* = 17.0 Hz, 1H), 2.92 (d, *J* = 17.1 Hz, 1H); ^{13}C NMR (150 MHz, $CDCl_3$) δ 169.42, 142.80, 136.72, 136.68, 135.53, 130.56, 129.50, 128.89, 128.54, 128.16, 127.93, 126.93, 123.34, 122.65, 120.52, 73.67, 60.42, 45.43, 43.45. IR (cm^{-1}): 3085 (w), 3055 (m), 3030 (w), 2930 (w), 2906 (w), 2846 (w), 1695 (vs), 1590 (w), 1486 (s), 1414 (m), 1362 (s), 829 (m), 758 (m), 695 (vs), 672 (w).

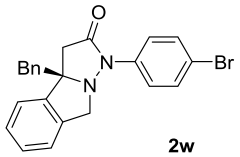

2w

(*S*)-3*a*-Benzyl-1-(4-bromophenyl)-3*a*,8-dihydro-1*H*-pyrazolo[5,1-*a*]isoindol-2(3*H*)-one (2w): Pale yellow solid, mp = 144-145 ℃; Eluent: petroleum ether/EtOAc 4:1; Yield: 80%;

HPLC analysis: Daicel Chiralpak IB; hexane/*i*PrOH: 95:5; flow: 1.0 mL/min; λ = 220 nm. 25 °C. 98% ee (t_R (minor) = 13.7 min, t_R (major) = 39.6 min).

HRMS (ESI$^+$): Calcd for $C_{23}H_{20}BrN_2O$, $[M+H]^+$ *m/z* 419.0759. Found 419.0757.

^1H NMR (600 MHz, $CDCl_3$) δ 7.68 (d, *J* = 8.9 Hz, 2H), 7.47 (d, *J* = 9.0 Hz, 2H), 7.39-7.32 (m, 1H), 7.32-7.24 (m, 3H), 7.17-7.09 (m, 10H), 4.55 (d, *J* = 14.2 Hz, 2H), 3.98 (d, *J* = 14.2 Hz, 1H), 3.25-3.16 (m, 2H), 3.10 (d, *J* = 17.0 Hz, 1H), 2.92 (d, *J* = 17.0 Hz, 1H); ^{13}C NMR (100 MHz, $CDCl_3$) δ 169.52, 142.78, 137.20, 136.73, 135.53, 131.87, 130.59, 128.58, 128.20, 127.97, 126.97, 123.38, 122.67, 120.85, 117.26, 73.68, 60.45, 45.44, 43.50. IR (cm^{-1}): 3030 (w), 2907 (w), 2848 (w), 1693 (vs), 1588 (w), 1483 (s), 1461 (m), 1343 (s), 1068 (m), 828 (m), 757 (s), 729 (w), 697 (vs), 669 (s).

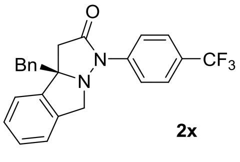

2x

(*S*)-3*a*-Benzyl-1-(4-(trifluoromethyl)phenyl)-3*a*,8-dihydro-1*H*-pyrazolo[5,1-*a*]isoindol-2(3*H*)-one (2x): Pale yellow solid, Mp = 171-173 ℃; Eluent: petroleum ether/EtOAc 4:1; Yield: 84%;

HPLC analysis: Daicel Chiralpak IB; hexane/*i*PrOH: 95:5; flow: 1.0 mL/min; λ = 220 nm. 25 °C. 98% ee (t_R (minor) = 14.0 min, t_R (major) = 41.2 min).

HRMS (ESI$^+$): Calcd for $C_{24}H_{20}F_3N_2O$, [M+H]$^+$ *m/z* 409.1528. Found 409.1520.

^{1}H NMR (400 MHz, $CDCl_3$) δ 7.91 (d, *J* = 8.7 Hz, 2H), 7.61 (d, *J* = 8.7 Hz, 2H), 7.39-7.28 (m, 3H), 7.19-7.06 (m, 6H), 4.63 (d, *J* = 14.1 Hz, 1H), 3.99 (d, *J* = 14.1 Hz, 1H), 3.26-3.17 (m, 2H), 3.13 (d, *J* = 17.1 Hz, 1H), 2.94 (d, *J* = 17.1 Hz, 1H); ^{13}C NMR (100 MHz, $CDCl_3$) δ 170.27, 142.63, 140.98, 136.53, 135.38, 130.55, 128.65, 128.29, 127.98, 127.02, 126.11 (q, $^3J_{C-F}$ = 3.7 Hz), 126.05, 125.83 (q, $^2J_{C-F}$ = 32.6 Hz), 124.30 (q, $^1J_{C-F}$ = 270.0 Hz), 123.40, 122.63, 118.69, 73.70, 60.59, 45.44, 43.48. ^{19}F NMR (283 MHz, $CDCl_3$) δ -61.89. IR (cm^{-1}): 3085 (w), 3027 (w), 2918 (w), 2853 (w), 1686 (vs), 1608 (m), 1512 (w), 1361 (m), 1327 (s), 1170 (m), 1117 (s), 1065 (m), 844 (s), 730 (w), 698 (vs), 666 (w).

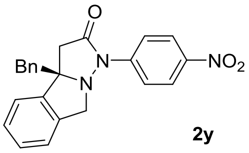

2y

(*S*)-3*a*-Benzyl-1-(4-nitrophenyl)-3*a*,8-dihydro-1*H*-pyrazolo[5,1-*a*]isoindol-2(3*H*)-one (2y): Pale yellow solid, mp = 158-159 °C; Eluent: petroleum ether/EtOAc 4:1; Yield: 82%;

HPLC analysis: Daicel Chiralpak IB; hexane/*i*PrOH: 70:30; flow: 1.0 mL/min; λ = 220 nm. 25 °C. 98% ee (t_R (minor) = 11.6 min, t_R (major) = 24.9 min).

HRMS (ESI$^+$): Calcd for $C_{23}H_{20}N_3O_3$, [M+H]$^+$ *m/z* 386.1505. Found 386.1504.

^{1}H NMR (600 MHz, $CDCl_3$) δ 8.22 (d, *J* = 9.2 Hz, 2H), 7.94 (d, *J* = 9.2 Hz, 2H), 7.42-7.37 (m, 1H), 7.36-7.32 (m, 2H), 7.20 (d, *J* = 7.8 Hz, 1H), 7.17-7.13 (m, 2H), 7.11-7.04 (m, 3H), 4.73 (d, *J* = 13.8 Hz, 1H), 4.00 (d, *J* = 13.8 Hz, 1H), 3.23 (s, 2H), 3.14 (d, *J* = 17.2 Hz, 1H), 2.97 (d, *J* = 17.2 Hz, 1H); ^{13}C NMR (100 MHz, $CDCl_3$) δ 171.02, 143.35, 143.33, 142.35, 136.01, 135.01, 130.35, 128.77, 128.44, 128.01, 127.12, 124.82, 123.42, 122.50, 118.11, 73.62, 60.68, 45.45, 43.19. IR (cm^{-1}): 3028 (w), 2920 (w), 1711 (s), 1589 (m), 1491 (s), 1458 (m), 1314 (vs), 1109 (m), 1117 (s), 899 (s), 753 (m), 700 (s), 637 (w).

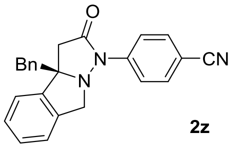

2z

(*S*)-4-(3*a*-Benzyl-2-oxo-2,3,3*a*,8-tetrahydro-1*H*-pyrazolo[5,1-*a*]isoindol-1-yl)benzonitrile (2z): Pale yellow solid, mp = 168-169 ℃; Eluent: petroleum ether/EtOAc 4:1; Yield: 83%;

HPLC analysis: Daicel Chiralpak IB; hexane/*i*PrOH: 80:20; flow: 1.0 mL/min; λ = 220 nm. 25 ^{o}C. 98% ee (t_R (minor) = 14.3 min, t_R (major) = 25.6 min).

HRMS (ESI$^+$): Calcd for $C_{24}H_{20}N_3O$, [M+H]$^+$ *m/z* 366.1606. Found 366.1604.

^{1}H NMR (600 MHz, $CDCl_3$) δ 7.90 (d, *J* = 8.9 Hz, 2H), 7.62 (d, *J* = 8.9 Hz, 2H), 7.41-7.35 (m, 1H), 7.35-7.30 (m, 2H), 7.18 (d, *J* = 8.1 Hz, 1H), 7.16-7.12 (m, 2H), 7.11-7.05 (m, 3H), 4.68 (d, *J* = 13.9 Hz, 1H), 3.98 (d, *J* = 13.8 Hz, 1H), 3.21 (s, 2H), 3.12 (d, *J* = 17.2 Hz, 1H), 2.95 (d, *J* = 17.2 Hz, 1H); ^{13}C NMR (150 MHz, $CDCl_3$) δ 170.72, 142.41, 141.68, 136.12, 135.09, 133.00, 130.36, 128.68, 128.35, 127.94, 127.03, 123.35, 122.50, 119.07, 118.57, 106.93, 73.59, 60.61, 45.40, 43.25. IR (cm^{-1}): 3124 (w), 3088 (w), 3029 (w), 2928 (w), 2838 (w), 2218 (s), 1703 (vs), 1600 (s), 1499 (s), 1417 (s), 1367 (m), 775 (m), 750 (m), 701 (s), 673 (vs), 651 (w).

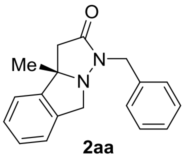

2aa

(*S*)-1-Benzyl-3*a*-methyl-3*a*,8-dihydro-1*H*-pyrazolo[5,1-*a*]isoindol-2(3*H*)-one (2aa): Light yellow oil; Eluent: petroleum ether/EtOAc 4:1; Yield: 77%;

HPLC analysis: Daicel Chiralpak IB; hexane/*i*PrOH: 95:5; flow: 1.0 mL/min; λ = 220 nm. 25 ^{o}C. 99% ee (t_R (minor) = 12.2 min, t_R (major) = 18.5 min).

HRMS (ESI$^+$): Calcd for $C_{18}H_{19}N_2O$, [M+H]$^+$ *m/z* 279.1497. Found 279.1488.

^{1}H NMR (400 MHz, $CDCl_3$) δ 7.40-7.28 (m, 5H), 7.27-7.21 (m, 2H), 7.17-7.12 (m, 1H), 7.09 (d, *J* = 6.7 Hz, 1H), 4.74-4.62 (m, 2H), 4.32 (d, *J* = 13.6 Hz, 1H), 3.89 (d, *J* = 13.6 Hz, 1H), 2.91 (d, *J* = 16.5 Hz, 1H), 2.46 (d, *J* = 16.5 Hz, 1H), 1.43 (s, 3H); ^{13}C NMR (100 MHz, $CDCl_3$) δ 171.49, 143.60, 136.73, 128.89, 128.65, 128.18, 128.07, 127.84, 123.20, 122.01, 71.62, 61.03, 48.52, 42.62, 25.91. IR (cm^{-1}): 3031 (w), 2963 (w), 2922 (w), 2838 (w), 1683 (vs), 1455 (m), 1402 (s), 1265 (m), 1116 (m), 1075 (m), 759 (m), 731 (m), 700 (vs), 653 (w).

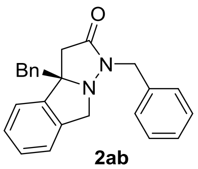

2ab

(*S*)-1,3*a*-Dibenzyl-3*a*,8-dihydro-1*H*-pyrazolo[5,1-*a*]isoindol-2(3*H*)-one (2ab): Pale yellow solid, mp = 137-138 ℃; Eluent: petroleum ether/EtOAc 4:1; Yield: 79%;

HPLC analysis: Daicel Chiralpak IB; hexane/*i*PrOH: 95:5; flow: 1.0 mL/min; λ = 220 nm. 25 °C. 99% ee (t_R (minor) = 18.6 min, t_R (major) = 21.9 min).

HRMS (ESI$^+$): Calcd for $C_{24}H_{23}N_2O$, $[M+H]^+$ *m/z* 355.1810. Found 355.1806.

^{1}H NMR (600 MHz, $CDCl_3$) δ 7.39-7.29 (m, 5H), 7.27-7.23 (m, 1H), 7.21-7.15 (m, 2H), 7.14-7.07 (m, 3H), 7.00 (d, *J* = 7.5 Hz, 1H), 6.94-6.87 (m, 2H), 4.57-4.41 (m, 2H), 4.12 (d, *J* = 14.3 Hz, 1H), 3.86 (d, *J* = 14.3 Hz, 1H), 3.00 (s, 2H), 2.93 (d, *J* = 16.8 Hz, 1H), 2.73 (d, *J* = 16.8 Hz, 1H); ^{13}C NMR (100 MHz, $CDCl_3$) δ 170.35, 142.80, 137.36, 136.81, 135.94, 130.73, 129.03, 128.67, 128.16, 127.82, 127.76, 127.72, 126.56, 123.10, 122.62, 75.01, 60.67, 48.25, 45.05, 42.05. IR (cm^{-1}): 3031 (w), 2916 (w), 2847 (w), 1673 (vs), 1604 (m), 1409 (s), 1353 (m), 1293 (m), 1069 (m), 769 (s), 756 (m), 700 (vs), 665 (w).

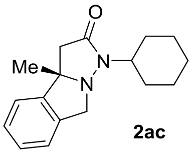

2ac

(*S*)-1-Cyclohexyl-3*a*-methyl-3*a*,8-dihydro-1*H*-pyrazolo[5,1-*a*]isoindol-2(3*H*)-one (2ac): Light yellow oil; Eluent: petroleum ether/EtOAc 4:1; Yield: 74%;

HPLC analysis: Daicel Chiralpak IB; hexane/*i*PrOH: 80:20; flow: 1.0 mL/min; λ = 220 nm. 25 °C. 98% ee (t_R (minor) = 4.6 min, t_R (major) = 5.7 min).

HRMS (ESI$^+$): Calcd for $C_{17}H_{23}N_2O$, $[M+H]^+$ *m/z* 271.1810. Found 271.1799.

^{1}H NMR (400 MHz, $CDCl_3$) δ 7.31-7.25 (m, 2H), 7.22-7.12 (m, 2H), 4.60 (d, *J* = 13.3 Hz, 1H), 4.16-4.02 (m, 2H), 2.88 (d, *J* = 16.3 Hz, 1H), 2.32 (d, *J* = 16.4 Hz, 1H), 1.91-1.78 (m, 3H), 1.75-1.54 (m, 4H), 1.51 (s, 3H) 1.47-1.31 (m, 2H), 1.23-1.07 (m, 1H); ^{13}C NMR (100 MHz, $CDCl_3$) δ 172.32, 143.28, 137.06, 128.13, 127.99, 123.07, 121.95, 72.09, 63.68, 53.37, 42.23, 31.77, 30.04, 25.87, 25.46. IR (cm^{-1}): 3043 (w), 2926 (w), 2854 (w), 1681 (vs), 1589 (m), 1482 (m), 1449 (s), 1250 (s), 1073 (m), 890 (m), 765 (s), 737 (s), 655 (w).

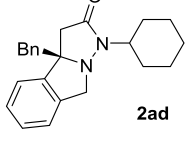

2ad

(*S*)-3*a*-Benzyl-1-cyclohexyl-3*a*,8-dihydro-1*H*-pyrazolo[5,1-*a*]isoindol-2(3*H*)-one (2ad): Pale yellow solid, mp = 134-135 °C; Eluent: petroleum ether/EtOAc 4:1; Yield: 76%;

HPLC analysis: Daicel Chiralpak IB; hexane/*i*PrOH: 98:2; flow: 1.0 mL/min; λ = 220 nm. 25 °C. 98% ee (t_R (minor) = 22.3 min, t_R (major) = 30.2 min).

HRMS (ESI$^+$): Calcd for $C_{23}H_{27}N_2O$, $[M+H]^+$ *m/z* 347.2123. Found 347.2121.

^{1}H NMR (400 MHz, $CDCl_3$) δ 7.23-7.10 (m, 7H), 7.10-7.04 (m, 2H), 4.53 (d, *J* = 13.6 Hz, 1H), 4.16-3.99 (m, 2H), 3.20 (d, *J* = 14.3 Hz, 1H), 3.06 (d, *J* = 14.3 Hz, 1H), 2.93 (d, *J* = 16.7 Hz, 1H), 2.62 (d, *J* = 16.8 Hz, 1H), 1.95-1.74 (m, 5H), 1.73-1.65 (m, 1H), 1.59 (qd, *J* = 12.3, 3.2 Hz, 1H), 1.48-1.32 (m, 2H), 1.18 (qt, *J* = 13.0, 3.3 Hz, 1H); ^{13}C NMR (100 MHz, $CDCl_3$) δ 171.37, 141.62, 137.61, 136.78, 130.80, 127.92, 127.77, 127.60, 126.42, 122.93, 122.89, 75.46, 63.72, 54.17, 44.19, 43.38, 31.92, 30.87, 25.92, 25.74, 25.56. IR (cm^{-1}): 3025 (w), 2931 (w), 2856 (w), 1674 (vs), 1602 (m), 1431 (m), 1405 (s), 1255 (m), 1206 (m), 731 (m), 697 (s), 673 (s), 636 (w).

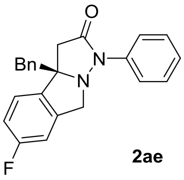

2ae

(*S*)-3*a*-Benzyl-6-fluoro-1-phenyl-3*a*,8-dihydro-1*H*-pyrazolo[5,1-*a*]isoindol-2(3*H*)-one (2ae): Light yellow oil; Eluent: petroleum ether/EtOAc 4:1; Yield: 84%;

HPLC analysis: Daicel Chiralpak IB; hexane/*i*PrOH: 95:5; flow: 1.0 mL/min; λ = 220 nm. 25 °C. 98% ee (t_R (minor) = 16.3 min, t_R (major) = 26.5 min).

HRMS (ESI$^+$): Calcd for $C_{23}H_{20}FN_2O$, $[M+H]^+$ *m/z* 359.1560. Found 359.1557.

^{1}H NMR (400 MHz, $CDCl_3$) δ 7.77 (d, *J* = 8.1 Hz, 2H), 7.37 (t, *J* = 8.0 Hz, 2H), 7.23-7.12 (m, 7H), 7.01 (td, *J* = 8.7, 2.0 Hz, 1H), 6.78 (d, *J* = 8.3 Hz, 1H), 4.44 (d, *J* = 14.9 Hz, 1H), 3.97 (d, *J* = 14.8 Hz, 1H), 3.18 (s, 2H), 3.09 (d, *J* = 17.0 Hz, 1H), 2.93 (d, *J* = 17.0 Hz, 1H); ^{13}C NMR (100 MHz, $CDCl_3$) δ 168.88, 163.03 (d, $^1J_{C\text{-}F}$ = 246.4 Hz), 139.40 (d, $^3J_{C\text{-}F}$ = 8.5 Hz), 138.59 (d, $^4J_{C\text{-}F}$ = 1.8 Hz), 137.87, 135.58, 130.69, 128.97, 127.96, 126.93, 124.82, 124.14 (d, $^3J_{C\text{-}F}$ = 9.1 Hz), 119.74, 115.26 (d, $^2J_{C\text{-}F}$ = 23.0 Hz), 110.5 (d, $^2J_{C\text{-}F}$ = 23.1 Hz), 73.26, 60.04, 45.45, 43.75; ^{19}F NMR (283 MHz, $CDCl_3$) δ -113.51. IR (cm^{-1}): 3062 (w), 3030 (w), 2917 (w), 2848 (w), 1696 (vs), 1595 (m), 1489 (s), 1405 (s), 1355 (m), 1311 (m), 1260 (m), 861 (m), 819 (m), 751 (vs), 695 (s).

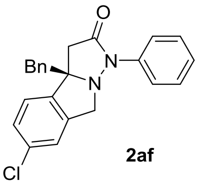

2af

(*S*)-3*a*-Benzyl-6-chloro-1-phenyl-3*a*,8-dihydro-1*H*-pyrazolo[5,1-*a*]isoindol-2(3*H*)-one (2af):

Light yellow oil; Eluent: petroleum ether/EtOAc 4:1; Yield: 86%;

HPLC analysis: Daicel Chiralpak IB; hexane/*i*PrOH: 95:5; flow: 1.0 mL/min; λ = 220 nm. 25 ^oC.

98% ee (t_R (minor) = 18.3 min, t_R (major) = 33.1 min).

HRMS (ESI^+): Calcd for $C_{23}H_{20}ClN_2O$, $[M+H]^+$ *m/z* 375.1264. Found 375. 1257.

^1H NMR (400 MHz, $CDCl_3$) δ 7.76 (d, *J* = 7.8 Hz, 2H), 7.41-7.35 (m, 2H), 7.30 (dd, *J* = 8.0, 1.7 Hz, 1H), 7.20-7.12 (m, 7H), 7.08 (s, 1H), 4.43 (d, *J* = 14.9 Hz, 1H), 3.97 (d, *J* = 14.9 Hz, 1H), 3.18 (s, 2H), 3.08 (d, *J* = 17.0 Hz, 1H), 2.93 (d, *J* = 17.0 Hz, 1H); ^{13}C NMR (100 MHz, $CDCl_3$) δ 168.74, 141.49, 139.15, 137.77, 135.42, 134.23, 130.67, 128.98, 128.30, 127.98, 126.96, 124.86, 124.00, 123.61, 119.73, 73.42, 59.86, 45.20, 43.59. IR (cm^{-1}): 3062 (w), 3030 (w), 2921 (w), 2850 (w), 1696 (vs), 1594 (m), 1491 (s), 1455 (s), 1356 (m), 1310 (m), 1076 (m), 752 (m), 728 (m), 694 (s).

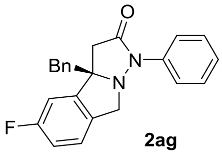

2ag

(*S*)-3*a*-Benzyl-5-fluoro-1-phenyl-3*a*,8-dihydro-1*H*-pyrazolo[5,1-*a*]isoindol-2(3*H*)-one (2ag):

Light yellow oil; Eluent: petroleum ether/EtOAc 4:1; Yield: 83%;

HPLC analysis: Daicel Chiralpak IB; hexane/*i*PrOH: 95:5; flow: 1.0 mL/min; λ = 220 nm. 25 ^oC.

98% ee (t_R (minor) = 16.7 min, t_R (major) = 21.7 min).

HRMS (ESI^+): Calcd for $C_{23}H_{20}FN_2O$, $[M+H]^+$ *m/z* 359.1560. Found 359.1550.

^1H NMR (400 MHz, $CDCl_3$) δ 7.78 (d, *J* = 7.9 Hz, 2H), 7.42-7.34 (m, 2H), 7.21-7.12 (m, 6H), 7.09-7.02 (m, 1H), 7.01-6.93 (m, 2H), 4.47 (d, *J* = 14.2 Hz, 1H), 3.95 (d, *J* = 14.2 Hz, 1H), 3.19 (s, 2H), 3.11 (d, *J* = 17.0 Hz, 1H), 2.92 (d, *J* = 17.0 Hz, 1H); ^{13}C NMR (100 MHz, $CDCl_3$) δ 168.79, 167.63 (d, $^3J_{C-F}$ = 4.6 Hz), 162.88 (d, $^1J_{C-F}$ = 245.7 Hz), 145.06 (d, $^3J_{C-F}$ = 7.8 Hz), 137.87, 135.36, 132.44, 130.69, 128.96, 128.00, 126.99, 124.77, 124.66, 119.62, 115.75 (d, $^2J_{C-F}$ = 22.8 Hz), 110.06 (d, $^2J_{C-F}$ = 23.5 Hz), 73.64, 59.86, 45.21, 43.46; ^{19}F NMR (283 MHz, $CDCl_3$) δ -113.74. IR

(cm^{-1}): 3064 (w), 3030 (w), 2923 (w), 2847 (w), 1693 (vs), 1594 (m), 1492 (s), 1458 (s), 1352 (m), 1311 (m), 1075 (m), 758 (m), 727 (m), 691 (s).

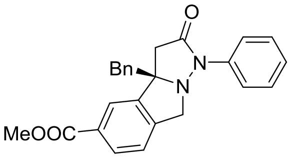

2ah

Methyl (*S*)-3*a*-benzyl-2-oxo-1-phenyl-2,3,3*a*,8-tetrahydro-1*H*-pyrazolo[5,1-*a*]isoindole-5-carboxylate (2ah): Light yellow oil; Eluent: petroleum ether/EtOAc 4:1; Yield: 78%;

HPLC analysis: Daicel Chiralpak IB; hexane/*i*PrOH: 85:15; flow: 1.0 mL/min; λ = 220 nm. 25 ^oC. 98% ee (t_R (minor) = 12.5 min, t_R (major) = 26.1 min).

HRMS (ESI^+): Calcd for $C_{25}H_{23}N_2O_3$, $[M+H]^+$ *m/z* 399.1709. Found 399.1699.

^1H NMR (400 MHz, $CDCl_3$) δ 8.03-7.93 (m, 2H), 7.82-7.73 (m, 2H), 7.40-7.35 (m, 2H), 7.20-7.10 (m, 7H), 4.51 (d, *J* = 15.4 Hz, 1H), 4.03 (d, *J* = 15.4 Hz, 1H), 3.96 (s, 3H), 3.25 (s, 2H), 3.13 (d, *J* = 17.0 Hz, 1H), 2.96 (d, *J* = 17.0 Hz, 1H); ^{13}C NMR (100 MHz, $CDCl_3$) δ 168.79, 166.74, 143.64, 142.55, 137.88, 135.42, 130.73, 130.40, 130.13, 129.00, 128.00, 126.98, 124.84, 124.14, 123.43, 119.70, 73.59, 60.38, 52.46, 45.39, 43.64. IR (cm^{-1}): 3062 (w), 3030 (w), 2958 (w), 2919 (w), 2849 (w), 1695 (vs), 1595 (m), 1489 (s), 1456 (s), 1354 (m), 1310 (s), 794 (m), 753 (vs), 695 (s), 630 (w).

5. Scale Synthesis of 2i

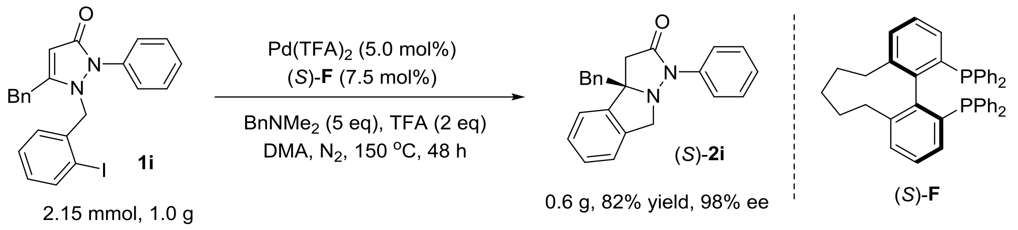

To a dried Schlenk tube were added Pd(TFA)$_2$ (35.9 mg, 0.108 mmol) and ligand (*S*)-**F** (95.1 mg, 0.161 mmol) under N$_2$, 25.0 mL of anhydrous *N*,*N*-dimethylacetamide (DMA) was then introduced via syringe. After stirring for 1 h, substrate **1i** (1.0 g, dissolved in 8 mL of DMA), BnNMe$_2$ (10.75 mmol, 5 equiv) and TFA (4.3 mmol, 2 equiv) were added via syringe. The mixture was vigorously stirred in a pre-warmed oil bath at 150 ℃ for 48 h. The solvent was then removed under vacuum, and the residue was purified by chromatography on silica to give the desired product **2i** (0.6 g, 82% yield, 98% ee).

6. Procedure for Hydroarylation Using Aryl Bromide 3

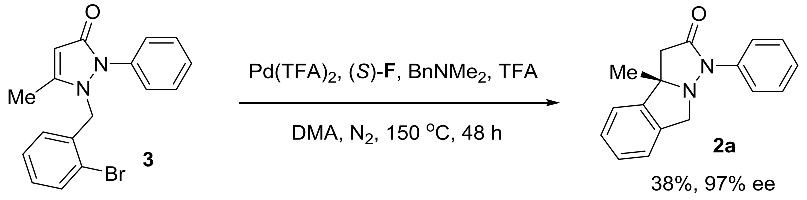

To a dried Schlenk tube were added Pd(TFA)$_2$ (3.32 mg, 0.01 mmol) and ligand (*S*)-**F** (8.85 mg, 0.015 mmol) under N$_2$, 3.0 mL of anhydrous *N*,*N*-dimethylacetamide (DMA) was then introduced via syringe. After stirring for 1 h, **3** (0.2 mmol, dissolved in 1mL of DMA), BnNMe$_2$ (1.0 mmol, 5 equiv) and TFA (0.4 mmol, 2 equiv) were added via syringe. The mixture was vigorously stirred in a pre-warmed oil bath at 150 ℃ for 24 h. The solvent was then removed under vacuum, and the residue was purified by chromatography on silica to give the desired product **2a** (38% yield, 97% ee).

1-(2-Bromobenzyl)-5-methyl-2-phenyl-1,2-dihydro-3*H*-pyrazol-3-one (3): Pale yellow solid, Mp = 158-159 ℃; Eluent: EtOAc; HRMS (ESI$^+$): Calcd for $C_{17}H_{16}BrN_2O$, [M+H]$^+$ *m/z* 343.0446. Found 343.0441. ^{1}H NMR (400 MHz, CDCl$_3$) δ 7.55-7.44 (m, 2H), 7.41 (t, *J* = 7.8 Hz, 2H), 7.28-7.22 (m, 3H), 7.18 (td, *J* = 7.7, 1.5 Hz, 1H), 6.62 (s, 1H), 6.54 (dd, *J* = 7.5, 1.1 Hz, 1H), 5.27

(s, 2H), 2.47 (s, 3H); ^{13}C NMR (100 MHz, $CDCl_3$) δ 158.26, 150.17, 133.33, 131.97, 131.76, 130.27, 130.06, 129.55, 128.32, 128.22, 127.16, 121.71, 93.21, 50.19, 13.15.

7. Application of the Synthesized Compound (*S*)-2i

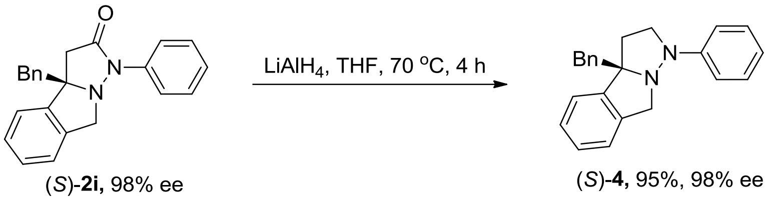

To a solution of (*S*)-3*a*-benzyl-1-phenyl-3a,8-dihydro-1*H*-pyrazolo[5,1-*a*]isoindol-2(3*H*)-one ((*S*)-**2i**, 68 mg, 0.2 mmol, 98% ee) in THF (4 mL) was added $LiAlH_4$ (38 mg, 1.0 mmol) at room temperature and the mixture was stirred at 70 ℃ for 4 h. After which, the mixture was quenched with water at room temperature and extracted with ethyl acetate. The organic layers were washed with brine and dried over Na_2SO_4. After filtered and concentrated under vacuum, the residue was purified with flash chromatography on silica gel, eluting with ethyl acetate/petroleum ether 1:25 (v/v), to afford (*S*)-**4** (62 mg, 95%, 98% ee). White solid, mp = 97-98 ℃; HPLC analysis: Daicel Chiralpak IB; hexane/*i*PrOH: 99:1; flow: 0.5 mL/min; λ = 220 nm. 25 °C. 98% ee (t_R (minor) = 31.1 min, t_R (major) = 32.4 min). HRMS (ESI$^+$): Calcd for $C_{23}H_{23}N_2$, $[M+H]^+$ *m*/*z* 327.1861. Found 327.1864. ^{1}H NMR (600 MHz, $CDCl_3$) δ 7.29-7.20 (m, 3H), 7.16 (t, *J* = 7.4 Hz, 1H), 7.13-7.05 (m, 8H), 7.03 (d, *J* = 7.4 Hz, 1H), 6.80 (t, *J* = 7.3 Hz, 1H), 4.32 (d, *J* = 15.3 Hz, 1H), 3.96 (d, *J* = 15.2 Hz, 1H), 3.52-3.44 (m, 1H), 3.43-3.34 (m, 1H), 3.17 (d, *J* = 13.6 Hz, 1H), 3.03 (d, *J* = 13.6 Hz, 1H), 2.38-2.23 (m, 2H); ^{13}C NMR (150 MHz, $CDCl_3$) δ 151.69, 143.97, 140.38, 137.92, 130.74, 128.91, 127.55, 127.50, 127.04, 126.14, 122.66, 122.45, 118.45, 114.09, 80.08, 62.07, 50.74, 45.97, 39.35.

8. Procedure for Hydroarylation Using *o*-Iodobenzoyl Derivatives

(1) Synthesis of Substrates 5a-d

Compounds **5** were synthesized from the corresponding pyrazol-3-one and 2-iodobenzoyl chloride according to the literature procedure (Maruoka et al., 2013).

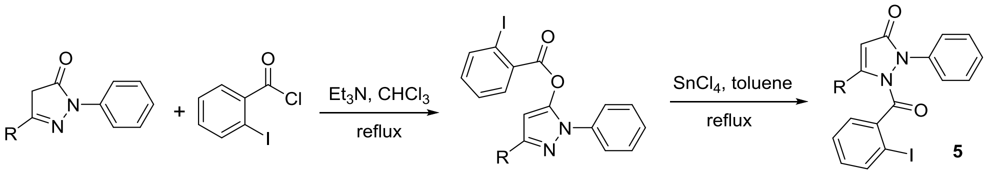

(2) General Procedures

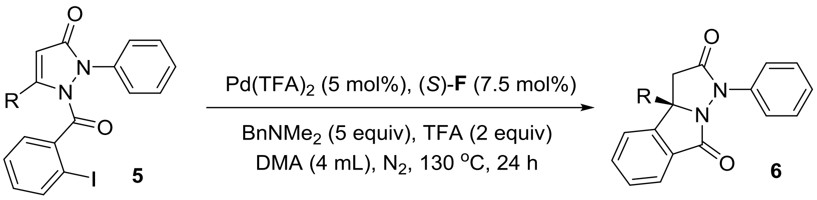

To a dried Schlenk tube were added Pd(TFA)$_2$ (3.32 mg, 0.01 mmol) and ligand (*S*)-**F** (8.85 mg, 0.015 mmol) under N_2, 3.0 mL of anhydrous *N*,*N*-dimethylacetamide (DMA) was then introduced via syringe. After stirring for 1 h, **5** (0.2 mmol, dissolved in 1mL of DMA), BnNMe$_2$ (1.0 mmol, 5 equiv) and TFA (0.4 mmol, 2 equiv) were added via syringe. The mixture was vigorously stirred in a pre-warmed oil bath at 130 ℃ for 24 h. The solvent was then removed under vacuum, and the residue was purified by chromatography on silica to give the desired product **6**. The enantiomeric excess was determined by chiral HPLC analysis.

Racemic products *Rac*-**6a-d** were prepared according to the above procedures in the absence of ligand

(3) Characterizaion Data of Substrates 5a-d and 6a-d

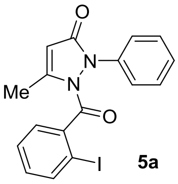

5a

1-(2-Iodobenzoyl)-5-methyl-2-phenyl-1,2-dihydro-3*H*-pyrazol-3-one (5a): Pale yellow solid, mp = 218-219 ℃; Eluent: petroleum ether/EtOAc 2:1; ^{1}H NMR (400 MHz, CDCl$_3$) δ 7.68 (d, *J* = 7.8 Hz, 1H), 7.16 (t, *J* = 7.4 Hz, 2H), 7.12-7.06 (m, 1H), 7.02 (t, *J* = 7.1 Hz, 1H), 6.97-6.91 (m, 3H), 6.86 (dd, *J* = 7.5, 1.5 Hz, 1H), 5.63 (s, 1H), 2.60 (s, 3H); ^{13}C NMR (100 MHz, CDCl$_3$) δ 167.74, 166.78, 155.74, 139.87, 139.01, 138.02, 131.78, 129.86, 128.95, 127.53, 127.24, 124.27, 102.70, 92.04, 15.98.

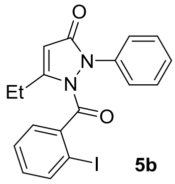

5b

5-Ethyl-1-(2-iodobenzoyl)-2-phenyl-1,2-dihydro-3*H*-pyrazol-3-one (5b): Pale yellow solid, mp = 204-205 ℃; Eluent: petroleum ether/EtOAc 2:1; ^{1}H NMR (400 MHz, CDCl$_3$) δ 7.65 (d, *J* = 7.8

Hz, 1H), 7.20-7.04 (m, 3H), 6.99 (t, *J* = 7.4 Hz, 1H), 6.95-6.85 (m, 3H), 6.82 (d, *J* = 7.2 Hz, 1H), 5.67 (s, 1H), 3.02 (q, *J* = 7.2 Hz, 2H), 1.40 (t, *J* = 7.3 Hz, 3H); ^{13}C NMR (100 MHz, $CDCl_3$) δ 167.97, 166.85, 161.96, 140.01, 138.86, 138.06, 131.59, 129.76, 128.87, 127.40, 127.12, 124.11, 100.75, 91.92, 22.78, 11.81.

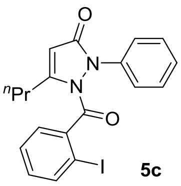

5c

1-(2-Iodobenzoyl)-2-phenyl-5-propyl-1,2-dihydro-3*H*-pyrazol-3-one (5c): Pale yellow solid, mp = 197-198 ℃; Eluent: petroleum ether/EtOAc 2:1; ^{1}H NMR (400 MHz, $CDCl_3$) δ 7.65 (d, *J* = 7.8 Hz, 1H), 7.14 (t, *J* = 7.4 Hz, 2H), 7.08 (t, *J* = 7.3 Hz, 1H), 6.99 (t, *J* = 7.5 Hz, 1H), 6.94-6.86 (m, 3H), 6.82 (d, *J* = 7.6 Hz, 1H), 5.66 (s, 1H), 2.98 (t, *J* = 7.6 Hz, 2H), 1.93-1.81 (m, 2H), 1.11 (t, *J* = 7.4 Hz, 3H); ^{13}C NMR (100 MHz, $CDCl_3$) δ 168.05, 166.86, 160.59, 140.08, 138.91, 138.16, 131.60, 129.80, 128.92, 127.41, 127.15, 124.13, 101.47, 91.95, 31.10, 21.04, 13.89.

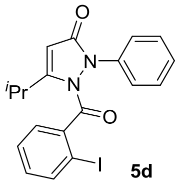

5d

1-(2-Iodobenzoyl)-5-isopropyl-2-phenyl-1,2-dihydro-3*H*-pyrazol-3-one (5d): Pale yellow solid, mp = 208-209 ℃; Eluent: petroleum ether/EtOAc 2:1; ^{1}H NMR (400 MHz, $CDCl_3$) δ 7.64 (d, *J* = 7.8 Hz, 1H), 7.14 (t, *J* = 7.4 Hz, 2H), 7.07 (t, *J* = 7.2 Hz, 1H), 6.98 (t, *J* = 7.5 Hz, 1H), 6.93- 6.83 (m, 3H), 6.79 (d, *J* = 7.5 Hz, 1H), 5.67 (s, 1H), 3.59 (hept, *J* = 6.6 Hz, 1H), 1.45 (d, *J* = 6.8 Hz, 6H); ^{13}C NMR (100 MHz, $CDCl_3$) δ 168.24, 167.41, 166.94, 140.39, 138.88, 138.29, 131.50, 129.79, 128.93, 127.37, 127.09, 123.98, 99.50, 91.85, 28.23, 22.15.

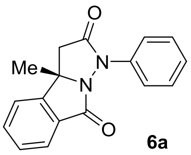

6a

(*S*)-3*a*-Methyl-1-phenyl-3,3*a*-dihydro-1*H*-pyrazolo[5,1-*a*]isoindole-2,8-dione (6a): Pale yellow solid, mp = 157-158 ℃; Eluent: petroleum ether/EtOAc 3:1.

HPLC analysis: Daicel Chiralpak IB; hexane/*i*PrOH: 80:20; flow: 1.0 mL/min; λ = 220 nm. 25 °C. 91% ee (t_R (minor) = 10.9 min, t_R (major) = 13.3 min);

^{1}H NMR (600 MHz, $CDCl_3$) δ 7.93 (d, *J* = 7.6 Hz, 1H), 7.78 (d, *J* = 9.7 Hz, 2H), 7.72 (t, *J* = 8.1 Hz, 1H), 7.58 (t, *J* = 7.9 Hz, 1H), 7.54 (d, *J* = 7.6 Hz, 1H), 7.44 -7.38 (m, 2H), 7.20 (t, *J* = 7.5 Hz, 1H), 2.86-2.77 (m, 2H), 1.76 (s, 3H); ^{13}C NMR (100 MHz, $CDCl_3$) δ 171.92, 171.20, 149.30, 139.14, 134.26, 129.69, 128.98, 128.83, 125.58, 125.23, 122.33, 118.55, 66.92, 45.69, 23.09.

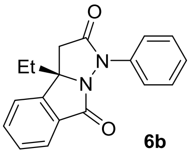

6b

(*S*)-3*a*-Ethyl-1-phenyl-3,3*a*-dihydro-1*H*-pyrazolo[5,1-*a*]isoindole-2,8-dione (6b): Pale yellow solid, mp = 149-150 ℃; Eluent: petroleum ether/EtOAc 3:1;

HPLC analysis: Daicel Chiralpak IB; hexane/iPrOH: 80:20; flow: 1.0 mL/min; λ = 220 nm. 25 oC. 87% ee (tR (minor) = 10.1 min, tR (major) = 12.4 min)

^{1}H NMR (400 MHz, $CDCl_3$) δ 7.92 (d, *J* = 7.7 Hz, 1H), 7.79 (d, *J* = 8.5 Hz, 2H), 7.71 (t, *J* = 7.6 Hz, 1H), 7.57 (t, *J* = 7.5 Hz, 1H), 7.48 (d, *J* = 7.7 Hz, 1H), 7.41 (t, *J* = 7.6 Hz, 2H), 7.19 (t, *J* = 7.2 Hz, 1H), 2.83 (s, 2H), 2.19 – 2.02 (m, 2H), 0.84 (t, *J* = 7.2 Hz, 3H); ^{13}C NMR (100 MHz, $CDCl_3$) δ 172.61, 171.39, 147.97, 139.08, 134.22, 129.62, 129.53, 128.96, 125.55, 125.03, 122.36, 118.35, 70.20, 44.84, 29.04, 8.04.

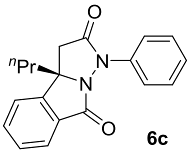

6c

(*S*)-1-Phenyl-3*a*-propyl-3,3*a*-dihydro-1*H*-pyrazolo[5,1-*a*]isoindole-2,8-dione (6c): Pale yellow solid, mp = 138-139 ℃; Eluent: petroleum ether/EtOAc 3:1;

HPLC analysis: Daicel Chiralpak IB; hexane/*i*PrOH: 80:20; flow: 1.0 mL/min; λ = 220 nm. 25 °C. 88% ee (t_R (minor) = 8.9 min, t_R (major) = 11.0 min)

^{1}H NMR (400 MHz, $CDCl_3$) δ 7.90 (d, *J* = 7.7 Hz, 1H), 7.78 (d, *J* = 8.6 Hz, 2H), 7.70 (t, *J* = 7.6 Hz, 1H), 7.55 (t, *J* = 7.5 Hz, 1H), 7.49 (d, *J* = 7.8 Hz, 1H), 7.41 (t, *J* = 7.8 Hz, 2H), 7.19 (t, *J* = 7.0 Hz, 1H), 2.82 (s, 2H), 2.11-1.95 (m, 2H), 1.52-1.37 (m, 1H), 1.16-1.00 (m, 1H), 0.86 (t, *J* = 7.3 Hz,

3H); ^{13}C NMR (100 MHz, $CDCl_3$) δ 172.51, 171.32, 148.36, 139.04, 134.18, 129.56, 129.31, 128.95, 125.50, 124.98, 122.35, 69.80, 44.99, 38.37, 17.05, 14.14.

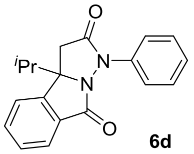

6d

3*a*-Isopropyl-1-phenyl-3,3*a*-dihydro-1*H*-pyrazolo[5,1-*a*]isoindole-2,8-dione (6d): Pale yellow solid, mp = 142-143 ℃; Eluent: petroleum ether/EtOAc 3:1;

HPLC analysis: Daicel Chiralpak IB; hexane/*i*PrOH: 80:20; flow: 1.0 mL/min; λ = 220 nm. 25 °C. 89% ee (t_R (minor) = 10.0 min, t_R (major) = 12.3 min)

^{1}H NMR (400 MHz, $CDCl_3$) δ 7.91 (d, *J* = 7.6 Hz, 1H), 7.81 (d, *J* = 8.4 Hz, 2H), 7.69 (t, *J* = 7.6 Hz, 1H), 7.55 (t, *J* = 7.5 Hz, 1H), 7.48 (d, *J* = 7.6 Hz, 1H), 7.41 (t, *J* = 7.9 Hz, 2H), 7.18 (t, *J* = 7.3 Hz, 1H), 3.03 (d, *J* = 16.7 Hz, 1H), 2.85 (d, *J* = 16.6 Hz, 1H), 2.36 (hept, *J* = 6.9 Hz, 1H), 1.03 (d, *J* = 6.9 Hz, 3H), 0.91 (d, *J* = 6.7 Hz, 3H); ^{13}C NMR (100 MHz, $CDCl_3$) δ 173.22, 171.48, 149.17, 138.91, 134.24, 129.48, 129.37, 128.98, 125.63, 124.90, 122.50, 118.13, 72.36, 43.05, 34.52, 16.92.

9. X-Ray Crystallographic Data for (*S*)-2a, (*S*)-E, (*S*)-F and (*S*)-G

(1) Crystal report of compound (*S*)-2a

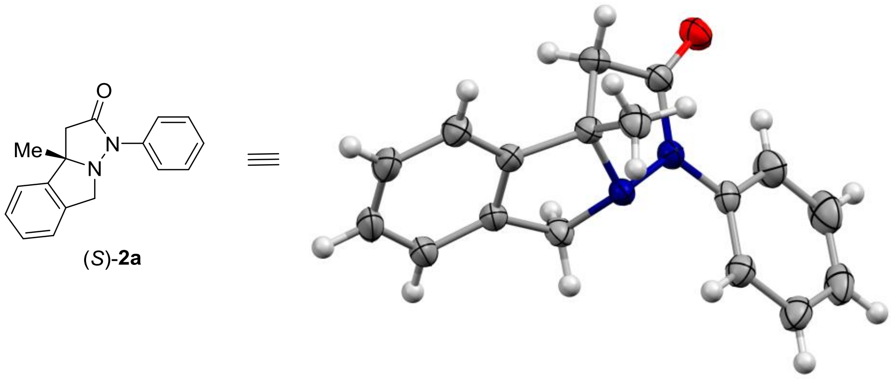

Figure S1. Crystal structure of (*S*)-2a showing 25% probability displacement ellipsoids, related to Table 1.

Table S1. Crystal Data and Structure Refinement for (*S*)-2a, related to Table 1.

Identification code	CCDC 1822025

Empirical formula	$C_{17}H_{16}N_2O$
Formula weight	264.32
Temperature/K	293(2)
Crystal system	orthorhombic
Space group	$P2_12_12_1$
a/Å	8.6230(3)
b/Å	9.0471(4)
c/Å	18.2985(5)
α/°	90
β/°	90
γ/°	90
Volume/Å^3	1427.52(9)
Z	4
ρ_{calc}g/cm^3	1.230
μ/mm^{-1}	0.613
F(000)	560.0
Crystal size/mm^3	0.55 × 0.40 × 0.30
Radiation	CuK\α (λ = 1.54184)
2Θ range for data collection/°	9.666 to 148.988
Index ranges	-5 <= h <= 10, -10 <= k <= 11, -22 <= l <= 22
Reflections collected	3465
Independent reflections	2443[R_{int} = 0.0147, R_{sigma} = 0.0242]
Data/restraints/parameters	2443/0/183
Goodness-of-fit on F^2	1.050
Final R indexes [I>=2σ (I)]	R_1 = 0.0328, wR_2 = 0.0874
Final R indexes [all data]	R_1 = 0.0354, wR_2 = 0.0904
Largest diff. peak/hole / e Å^{-3}	0.109/-0.107
Flack parameter	-0.1(3)

(2) Crystal report of compound (*S*)-E

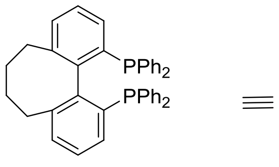

(*S*)-**E**

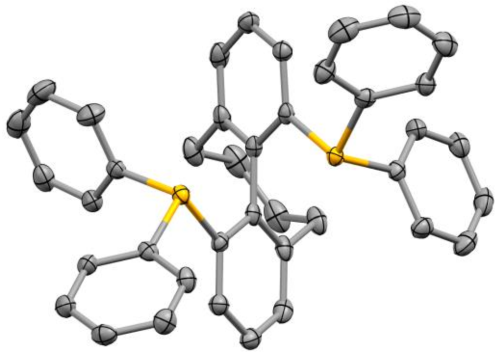

Figure S2. Crystal structure of (*S*)-E showing 60% probability displacement ellipsoids for non-H atoms, related to Figure 3.

Table S2. Crystal Data and Structure Refinement for (*S*)-E, related to Figure 3.

Identification code	CCDC 1842685
Empirical formula	$C_{40}H_{34}P_2$
Formula weight	576.61
Temperature/K	99.9(5)
Crystal system	orthorhombic
Space group	$P2_12_12_1$
a/Å	10.07990(10)
b/Å	15.64660(10)
c/Å	19.5369(2)
α/°	90
β/°	90
γ/°	90
Volume/Å^3	3081.28(5)
Z	4
ρ_{calc}g/cm^3	1.243
μ/mm^{-1}	1.477
F(000)	1216
Crystal size/mm^3	0.8 × 0.2 × 0.1

Radiation	CuK\a (λ = 1.54184)
2Θ range for data collection/°	7.238 to 193.384
Index ranges	-12 <= h <= 9, -19 <= k <= 19, -24 <= l <= 24
Reflections collected	25919
Independent reflections	6419 [R_{int} = 0.0437, R_{sigma} = 0.0331]
Data/restraints/parameters	6419 /0/ 379
Goodness-of-fit on F^2	1.039
Final R indexes [I>=2σ (I)]	R_1 = 0.0295, wR_2 = 0.0769
Final R indexes [all data]	R_1 = 0.0308, wR_2 = 0.0781
Largest diff. peak/hole / e $Å^{-3}$	0.345/ -0.244
Flack parameter	-0.011(8)

(3) Crystal report of compound (*S*)-F

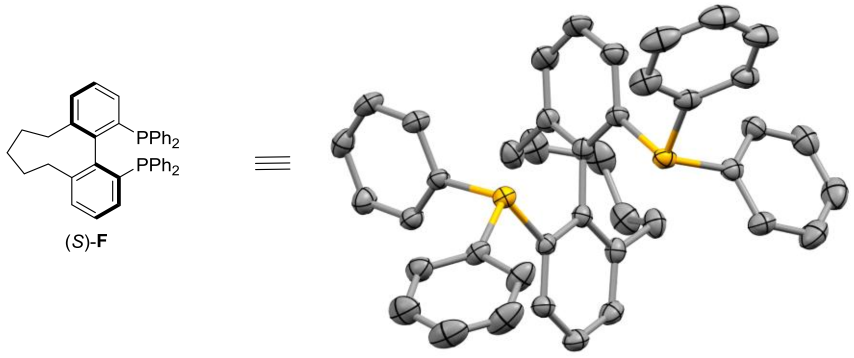

Figure S3. Crystal structure of (*S*)-F showing 25% probability displacement ellipsoids for non-H atoms, related to Figure 3.

Table S3. Crystal Data and Structure Refinement for (*S*)-F, related to Figure 3.

Identification code	CCDC 1822026
Empirical formula	$C_{41}H_{36}P_2$
Formula weight	590.64
Temperature/K	293(2)
Crystal system	monoclinic

Space group	C2
a/Å	20.4128(4)
b/Å	10.4888(2)
c/Å	15.8277(3)
α/°	90
β/°	99.378(2)
γ/°	90
Volume/Å^3	3343.51(11)
Z	4
ρ_{calc}g/cm^3	1.173
μ/mm^{-1}	1.372
F(000)	1248
Crystal size/mm^3	0.2 × 0.2 × 0.1
Radiation	CuK\a (λ = 1.54184)
2Θ range for data collection/ °	8.782 to 148.846
Index ranges	-23 <= h <= 25, -12 <= k <= 11, -15 <= l <= 19
Reflections collected	7520
Independent reflections	4917 [R_{int} = 0.0170, R_{sigma} = 0.0261]
Data/restraints/parameters	4917/1/388
Goodness-of-fit on F^2	1.022
Final R indexes [I>=2σ (I)]	R_1 = 0.0354, wR_2 = 0.1032
Final R indexes [all data]	R_1 = 0.0366, wR_2 = 0.1055
Largest diff. peak/hole / e Å^{-3}	0.38/-0.15
Flack parameter	0.022(11)

(4) Crystal report of compound (*S*)-G

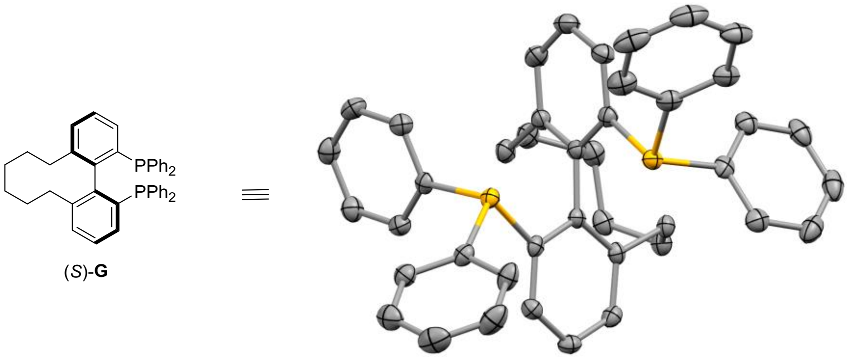

Figure S4. Crystal structure of (*S*)-G showing 60% probability displacement ellipsoids for non-H atoms, related to Figure 3.

Table S4. Crystal Data and Structure Refinement for (*S*)-G, related to Figure 3.

Identification code	CCDC 1842686
Empirical formula	$C_{42}H_{38}P_2$
Formula weight	604.66
Temperature/K	99.9(5)
Crystal system	monoclinic
Space group	C2
a/Å	20.3682(2)
b/Å	10.54400(10)
c/Å	15.6750(2)
α/°	90
β/°	99.9320(10)
γ/°	90
Volume/Å^3	3315.95(6)
Z	4
ρ_{calc}g/cm^3	1.211
μ/mm^{-1}	1.395
F(000)	1280
Crystal size/mm^3	0.45 ×0.4×0.32
Radiation	CuK\a (λ = 1.54184)

2Θ range for data collection/°	8.816 to 134.986
Index ranges	-23 <= h <= 24, -12 <= k <= 12, -18 <= l <= 18
Reflections collected	11595
Independent reflections	5431 [R_{int} = 0.0244, R_{sigma} = 0.0295]
Data/restraints/parameters	5431 /1/ 397
Goodness-of-fit on F^2	1.054
Final R indexes [I>=2σ (I)]	R_1 = 0.0263, wR_2 = 0.0687
Final R indexes [all data]	R_1 = 0.0264, wR_2 = 0.0688
Largest diff. peak/hole / e $Å^{-3}$	0.202/ -0.251
Flack parameter	0.012(7)

10. References

Maruoka, H., Yamagata, K., Okabe, F., and Tomioka, Y. (2013). *J.* Heterocyclic Chem. *50*, 164.

Otwinowski, Z., and Minor, W. (1997). Method Enzymol *276*, 307.

Roberts, L. R., Corbett, M. S., Fussell, S. J., Hitzel, L., Jessiman, A. S., Mason, H. J., Osborne, R., Ralph, M. J., Stennett, A. S. D., Wheeler, S., and Ian Storer, R. (2015). Tetrahedron Lett. *56*, 6246.

Sheng, X., Hua, K., Yang, C., Wang, X., Ji, H., Xu, J., Huang, Z., and Zhang, Y. (2015). Bioorg. Med. Chem. Lett. *25*, 3535.

Svenstrup, N.; Simonsen, K. B.; Thorup, N.; Brodersen, J.; Dehaen, W.; Becher, J. (1999). J. Org. Chem. *64*, 2814.

Wu, H.-C., Yu, J.-Q., and Spencer, J. B. (2004). Org. Lett. *6*, 4675.

Xie, J.-H., Wang, L.-X., Fu, Y., Zhu, S.-F., Fan, B.-M., Duan, H.-F., and Zhou, Q.-L. (2003). J. Am. Chem. Soc. *125*, 4404.

Yang, Y., Gong, H., and Kuang, C. (2013). Eur. J. Org. Chem. 5276.

Zhang, P., Yu, J., Peng, F., Wu, X., Jie, J., Liu, C., Tian, H., Yang, H., and Fu, H. (2016). Chem. Eur. J. *22*, 17477.

11. HPLC Analysis of Products M-4, 2a-ah, 4 and 6

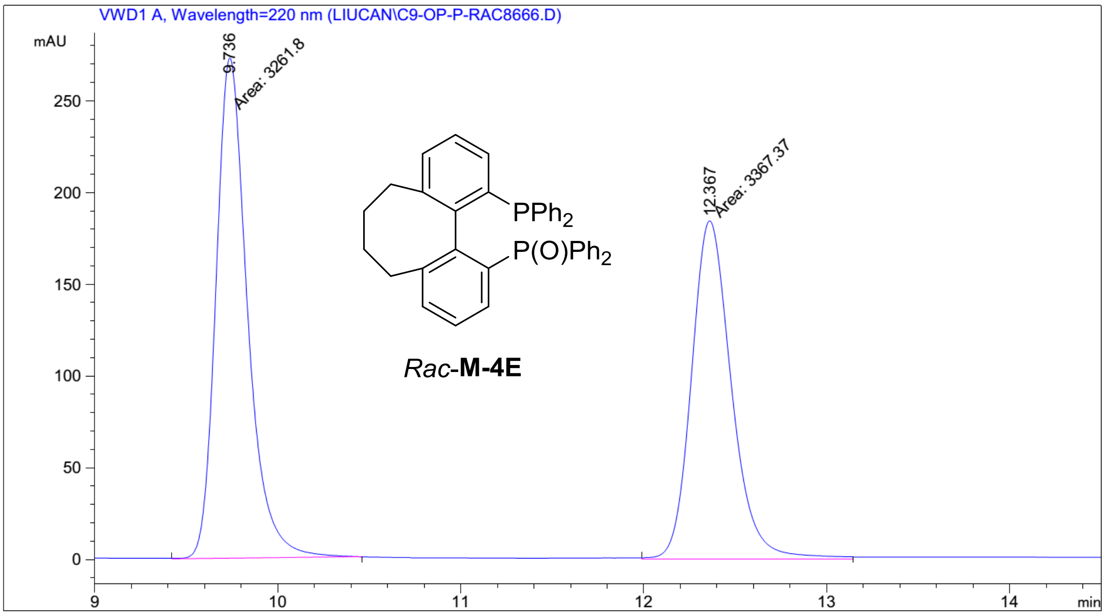

Peak #	RetTime [min]	Type	Width [min]	Area mAU *s	Height [mAU]	Area %
1	9.736	MM	0.1993	3261.80029	272.71555	49.2038
2	12.367	MM	0.3044	3367.36890	184.36462	50.7962

Figure S5. HPLC of *Rac*-M-4E, related to Figure 2.

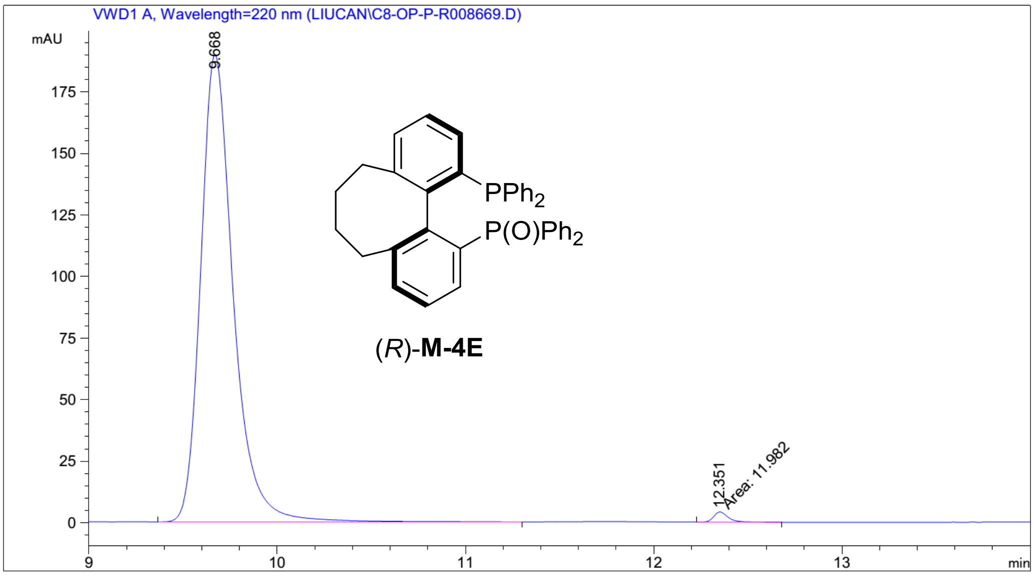

Peak #	RetTime [min]	Type	Width [min]	Area mAU *s	Height [mAU]	Area %
1	9.668	BB	0.1823	2280.00488	189.85515	99.4772
2	12.351	MM	0.0411	11.98201	4.85442	0.5228

Figure S6. HPLC of (*R*)-M-4E, related to Figure 2.

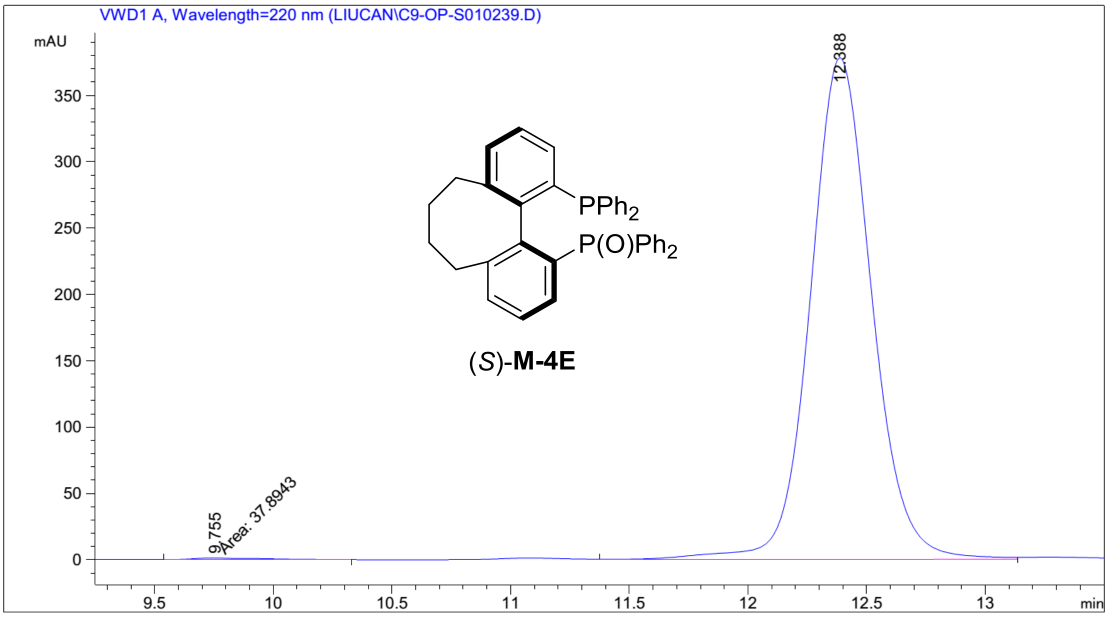

Peak #	RetTime [min]	Type	Width [min]	Area mAU *s	Height [mAU]	Area %
1	9.755	MM	0.1060	37.89430	5.95832	0.5526
2	12.388	VV	0.2746	6819.36377	377.95212	99.4474

Figure S7. HPLC of (*S*)-M-4E, related to Figure 2.

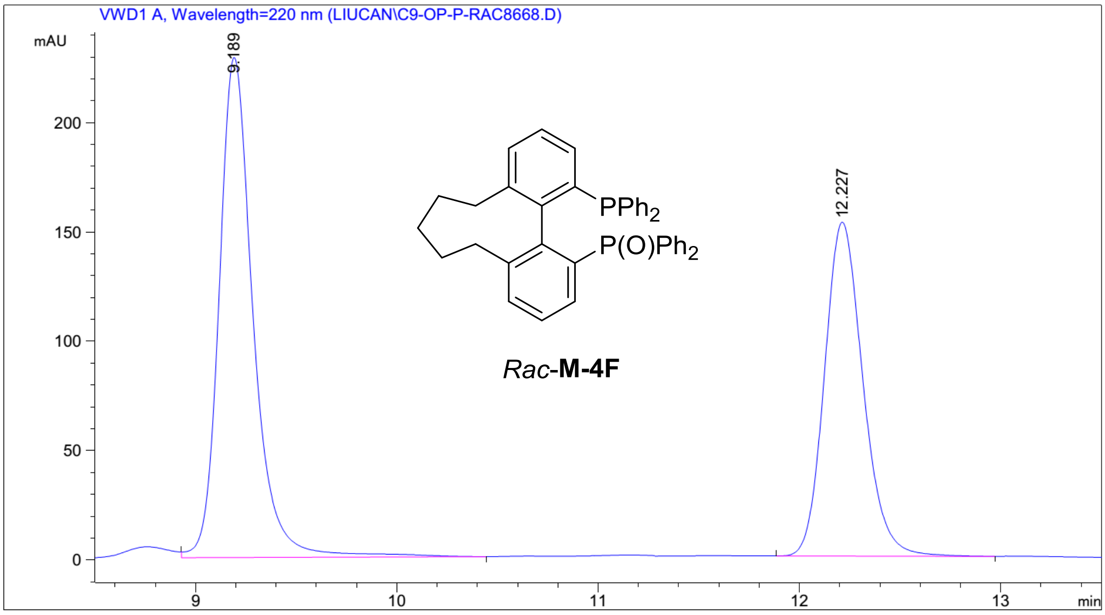

Peak #	RetTime [min]	Type	Width [min]	Area mAU *s	Height [mAU]	Area %
1	9.189	VB	0.1781	2693.27368	228.80057	50.9144
2	12.227	BB	0.2622	2596.53003	152.89336	49.0856

Figure S8. HPLC of *Rac*-M-4F, related to Figure 2.

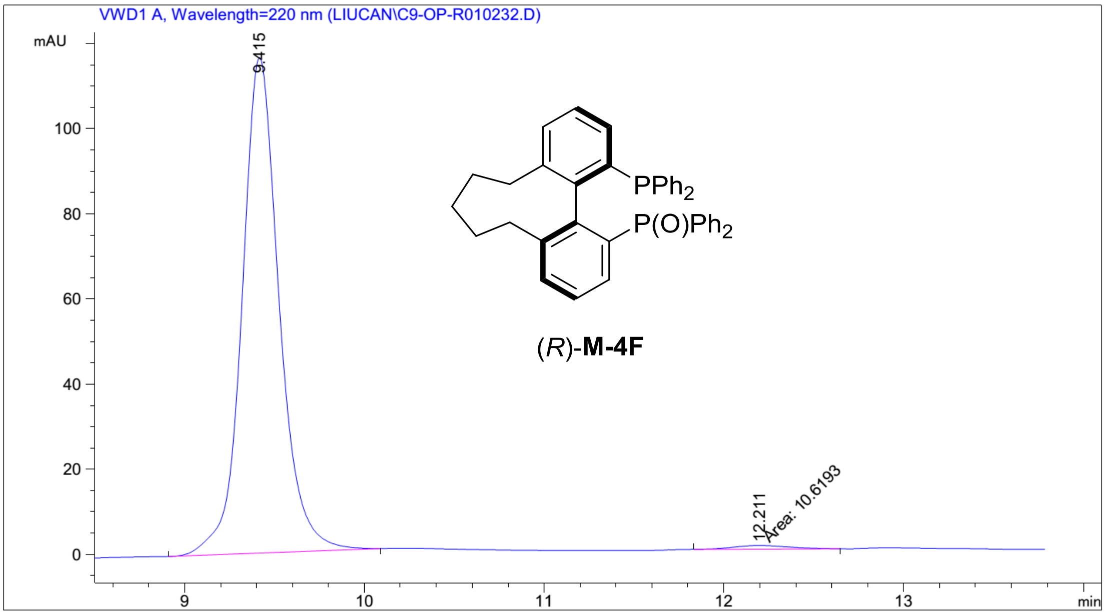

Peak #	RetTime [min]	Type	Width [min]	Area mAU *s	Height [mAU]	Area %
1	9.415	BB	0.2172	1672.99622	116.40432	99.3693
2	12.211	MM	0.1446	10.61933	1.31450	0.6307

Figure S9. HPLC of (*R*)-M-4F, related to Figure 2.

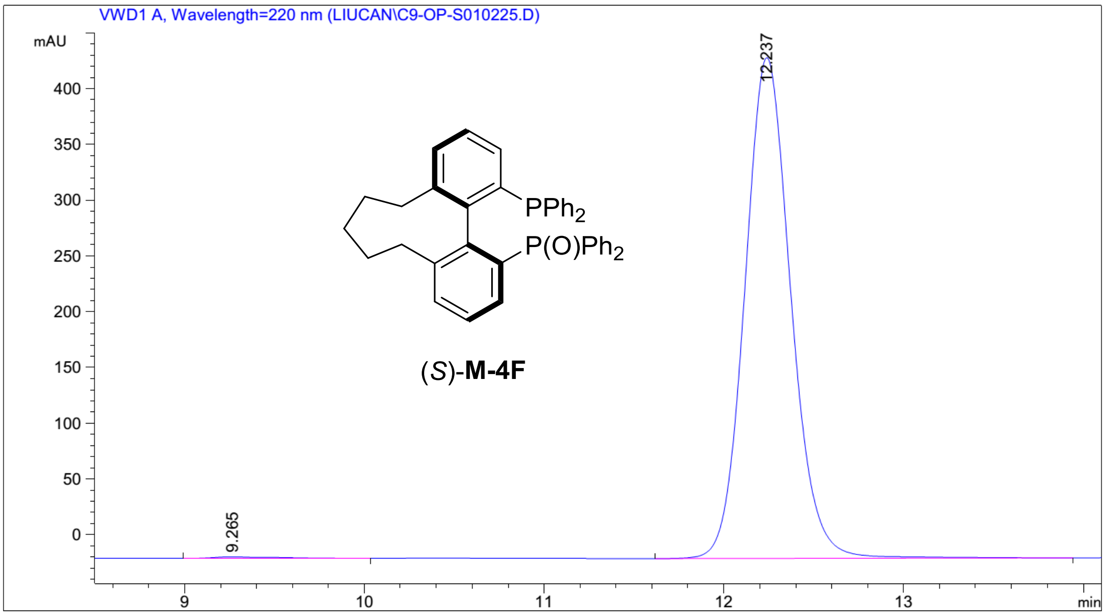

Peak #	RetTime [min]	Type	Width [min]	Area mAU *s	Height [mAU]	Area %
1	9.265	BB	0.1464	33.42749	1.46109	0.4179
2	12.237	BB	0.2739	7964.59814	448.94794	99.5821

Figure S10. HPLC of (*S*)-M-4F, related to Figure 2.

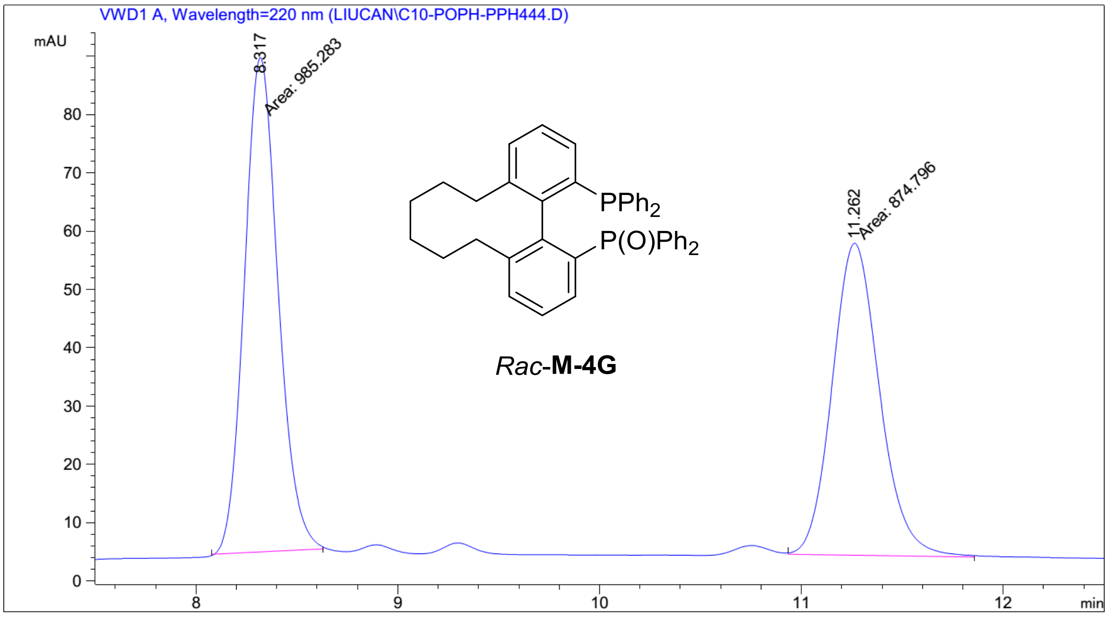

Peak #	RetTime [min]	Type	Width [min]	Area mAU *s	Height [mAU]	Area %
1	8.317	MM	0.1935	985.28302	84.85686	52.9700
2	11.262	MM	0.2722	874.79578	53.55995	47.0300

Figure S11. HPLC of *Rac*-M-4G, related to Figure 2.

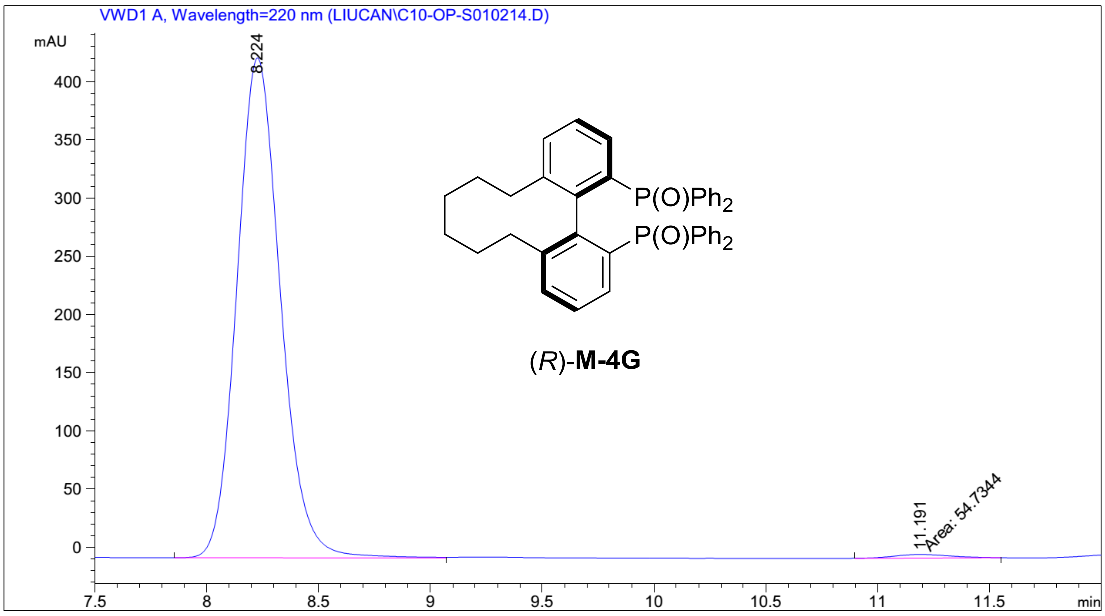

Peak #	RetTime [min]	Type	Width [min]	Area mAU *s	Height [mAU]	Area %
1	8.224	BV	0.2104	5812.08350	429.28046	99.0671
2	11.191	MM	0.2940	54.73437	3.10315	0.9329

Figure S12. HPLC of (*R*)-M-4G, related to Figure 2.

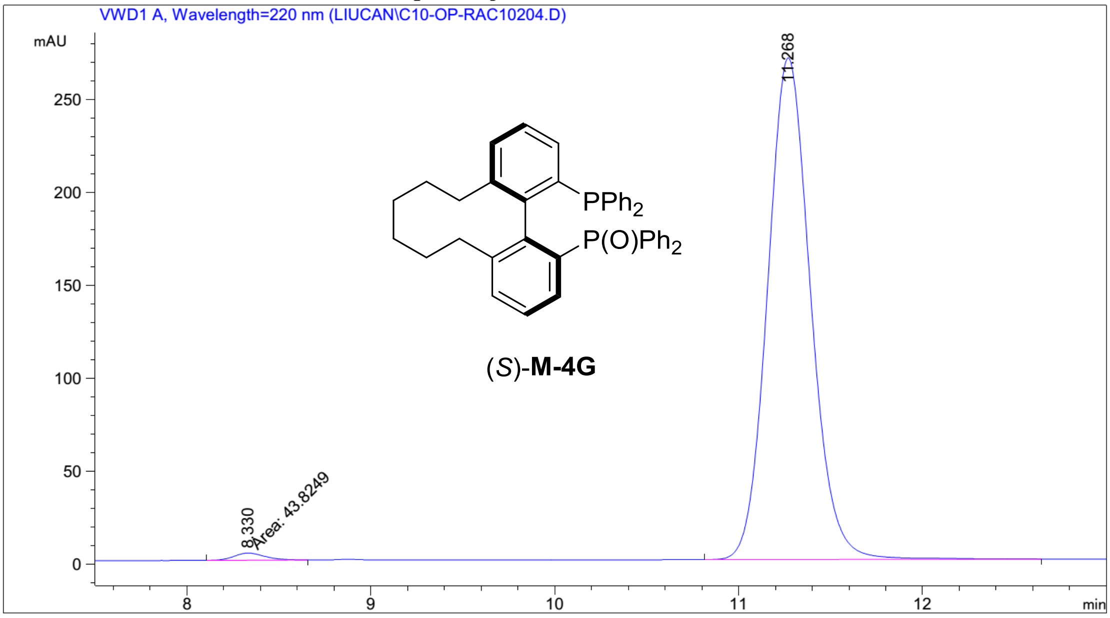

Peak #	RetTime [min]	Type	Width [min]	Area mAU *s	Height [mAU]	Area %
1	8.330	MM	0.1924	43.82491	3.79612	0.9911
2	11.268	BB	0.2503	4378.00195	270.00085	99.0089

Figure S13. HPLC of (*S*)-M-4G, related to Figure 2.

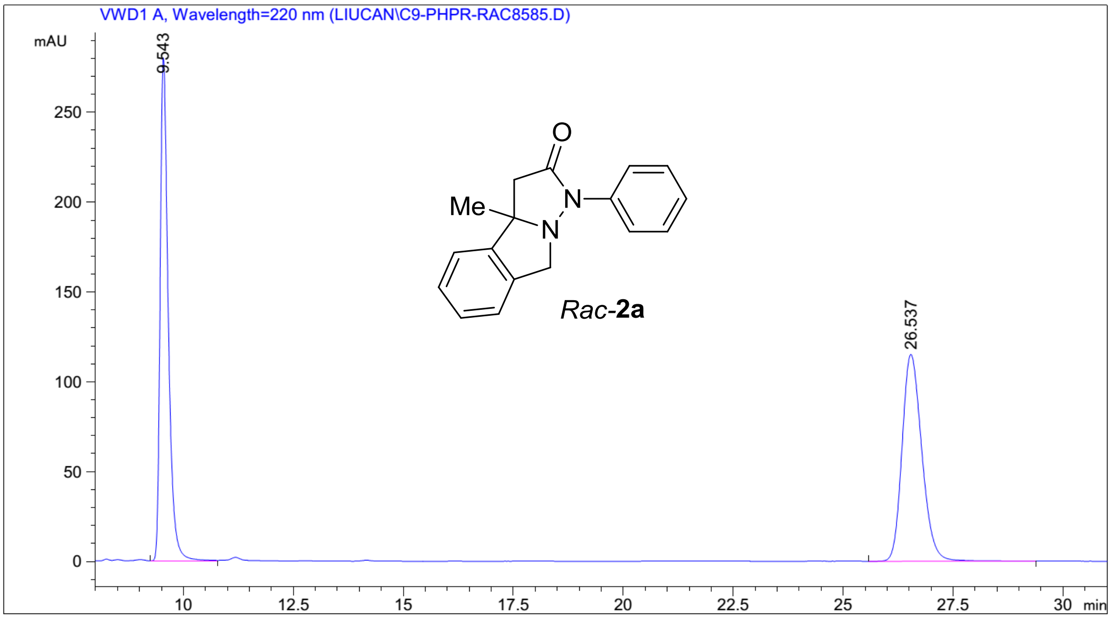

Peak #	RetTime [min]	Type	Width [min]	Area mAU *s	Height [mAU]	Area %
1	9.543	BV	0.1933	3589.92920	280.00446	49.9524
2	26.537	BB	0.4839	3596.77075	115.11349	50.0476

Figure S14. HPLC of *Rac*-2a, related to Table 1.

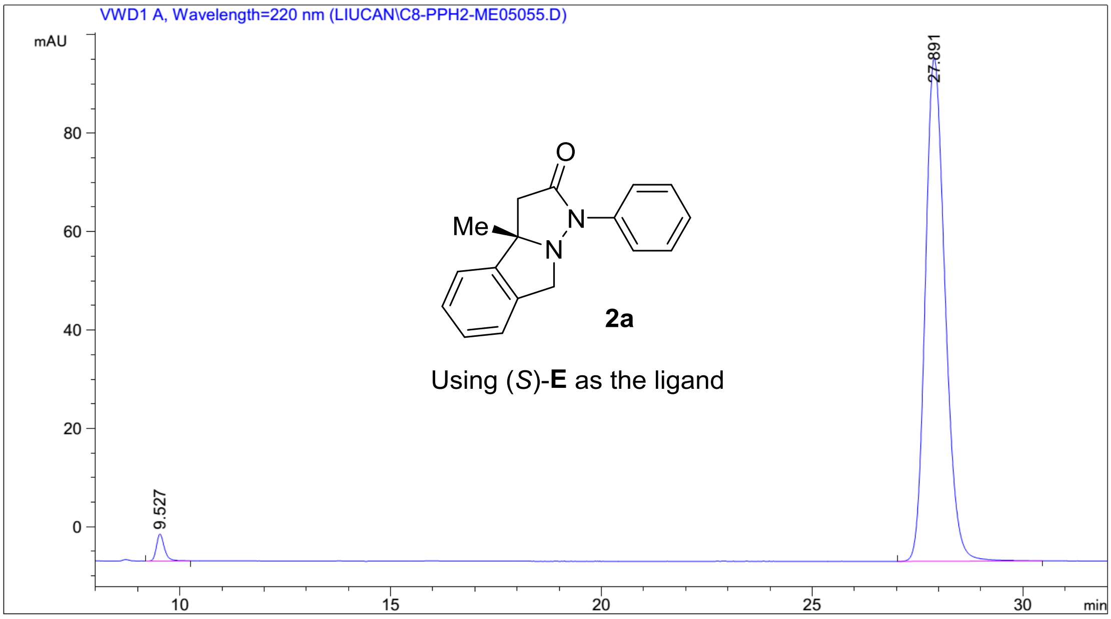

Peak #	RetTime [min]	Type	Width [min]	Area mAU *s	Height [mAU]	Area %
1	9.527	BB	0.1988	71.49776	5.48058	2.1022
2	27.891	BB	0.5049	3329.64673	102.31725	97.8978

Figure S15. HPLC of (*S*)-2a, related to Table 1.

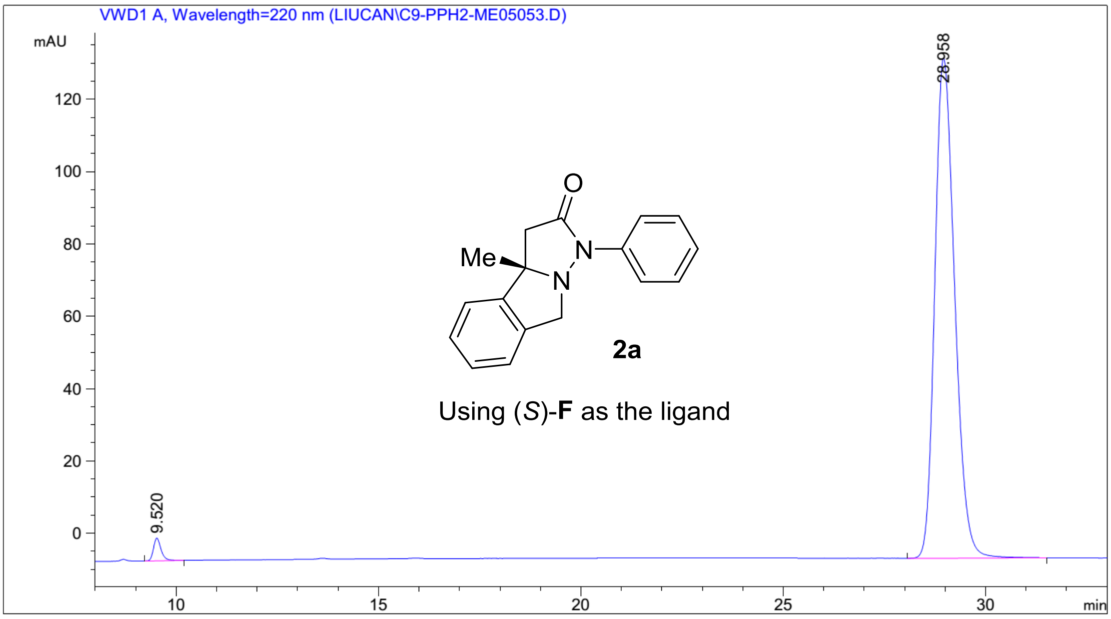

Peak #	RetTime [min]	Type	Width [min]	Area mAU *s	Height [mAU]	Area %
1	9.520	BB	0.1997	82.50786	6.28716	1.7191
2	28.958	BB	0.5304	4716.94922	138.28914	98.2809

Figure S16. HPLC of (*S*)-2a, related to Table 1.

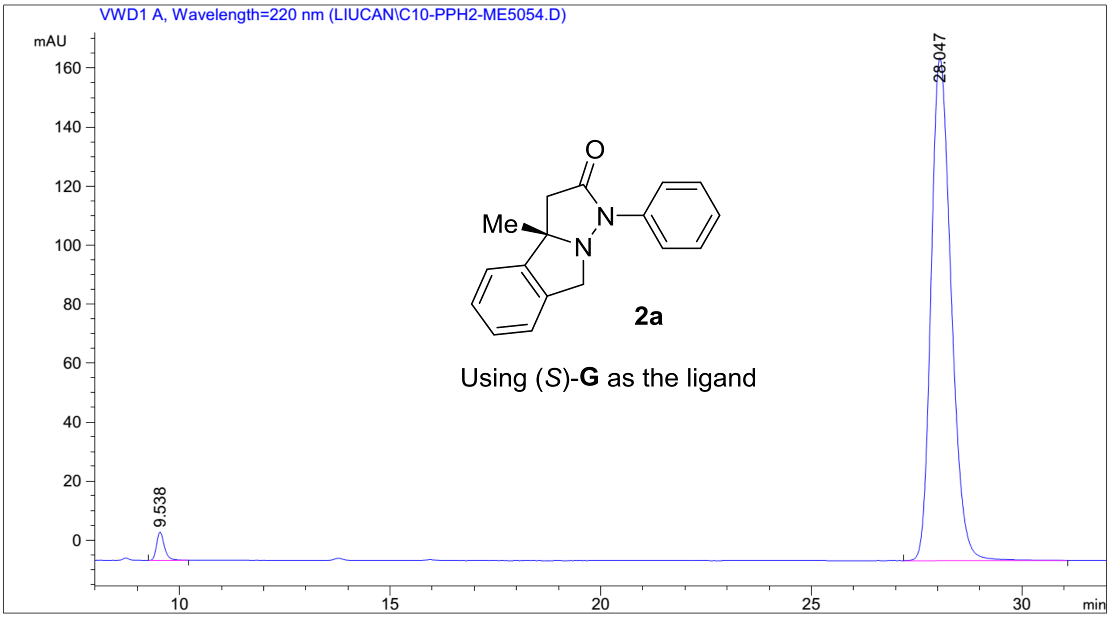

Peak #	RetTime [min]	Type	Width [min]	Area mAU *s	Height [mAU]	Area %
1	9.538	BB	0.1961	123.97203	9.58267	2.1489
2	28.047	BB	0.5174	5645.06152	170.44727	97.8511

Figure S17. HPLC of (*S*)-2a, related to Table 1.

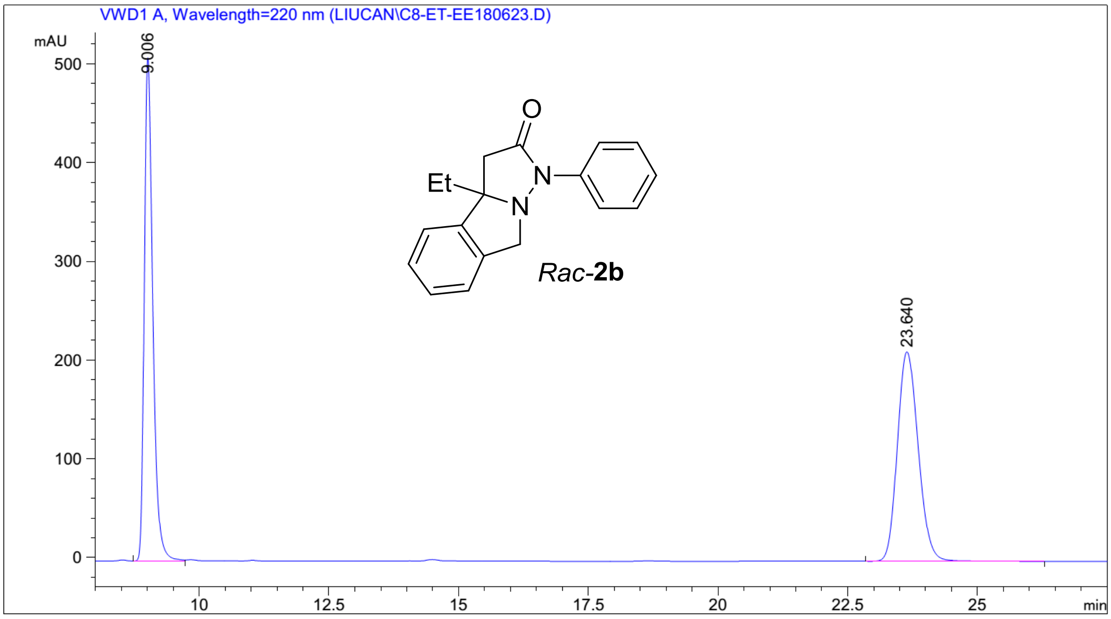

Peak #	RetTime [min]	Type	Width [min]	Area mAU *s	Height [mAU]	Area %
1	9.006	VV	0.1761	5851.36523	509.76456	49.6755
2	23.640	BB	0.4368	5927.81445	212.07364	50.3245

Figure S18. HPLC of *Rac*-2b, related to Table 2.

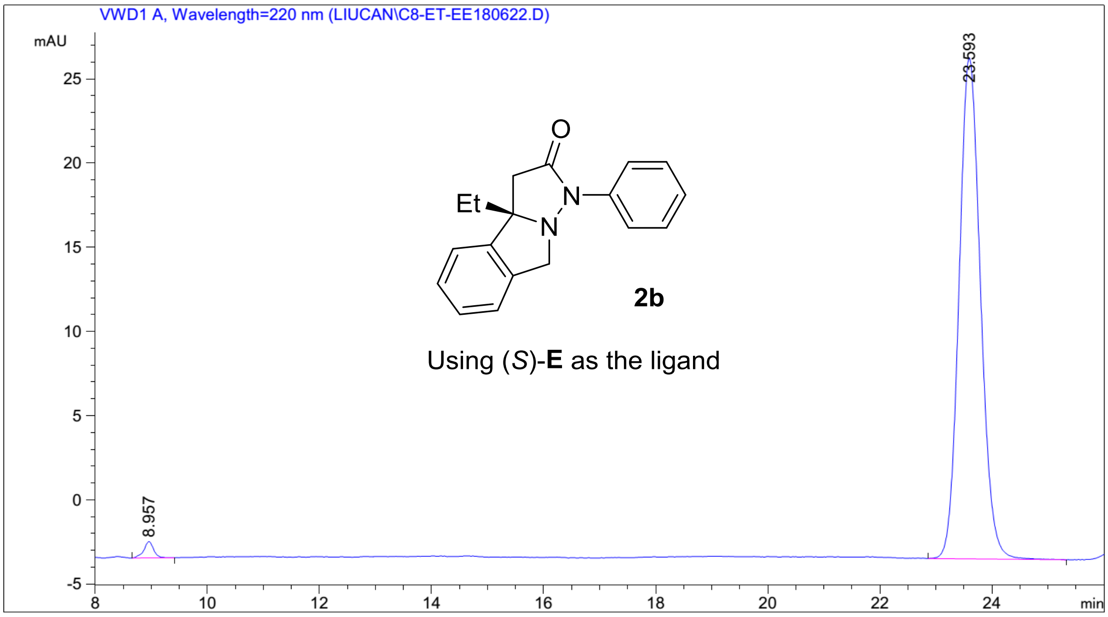

Peak #	RetTime [min]	Type	Width [min]	Area mAU *s	Height [mAU]	Area %
1	8.957	BB	0.1860	11.93875	9.68928e-1	1.4865
2	23.593	BB	0.4105	791.17944	29.77001	98.5135

Figure S19. HPLC of (*S*)-2b, related to Table 2.

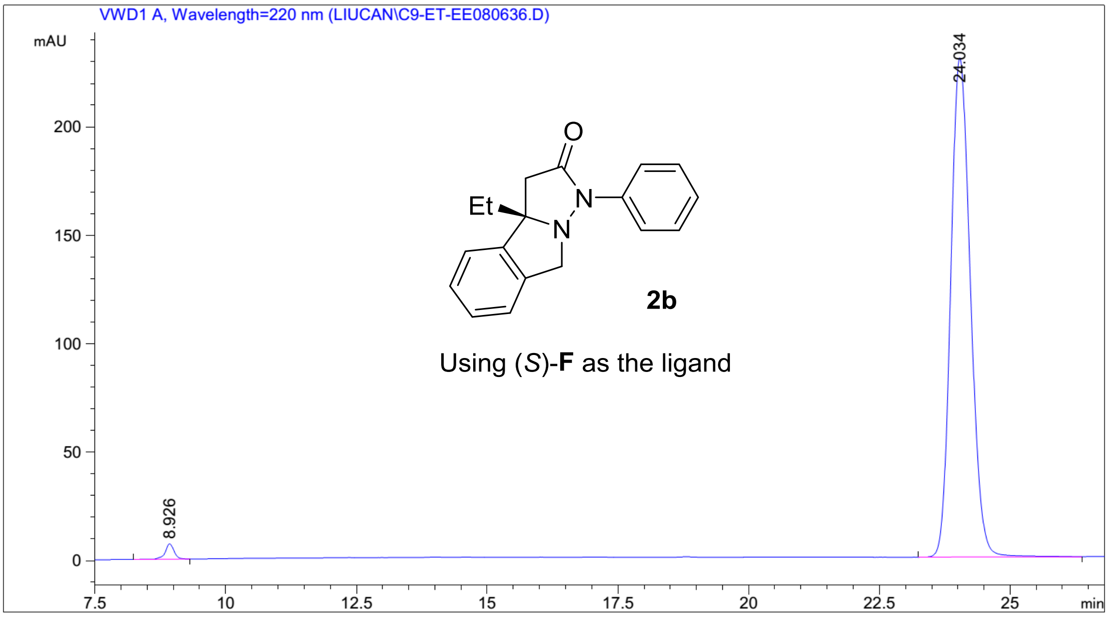

Peak #	RetTime [min]	Type	Width [min]	Area mAU *s	Height [mAU]	Area %
1	8.926	BB	0.1798	84.76732	7.11257	1.3884
2	24.034	BB	0.4070	6020.58252	230.23846	98.6116

Figure S20. HPLC of (*S*)-2b, related to Table 2.

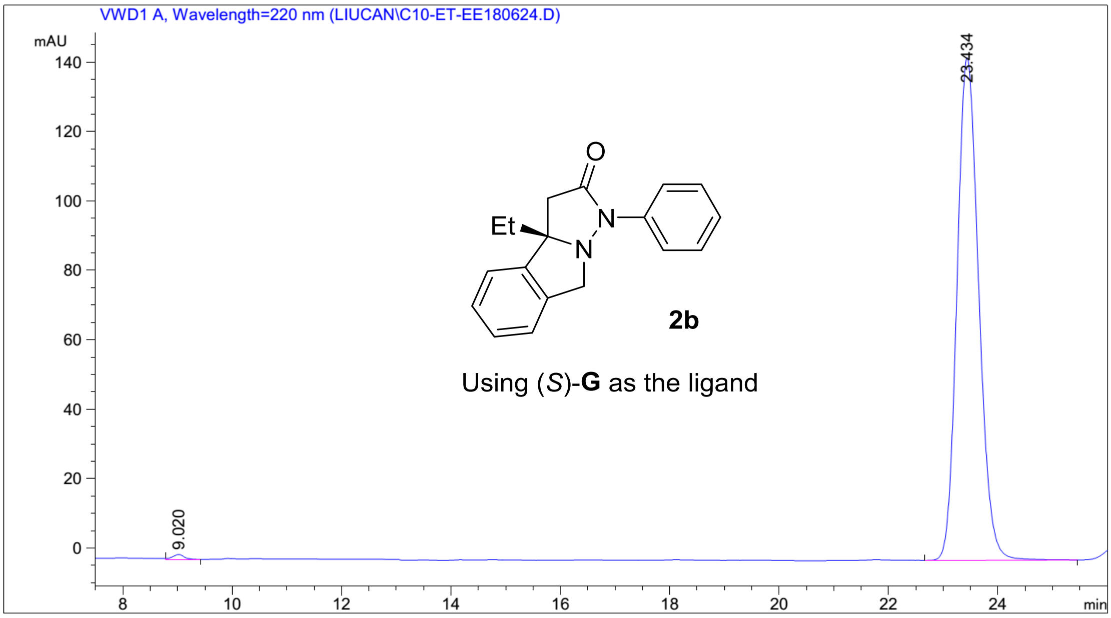

Peak #	RetTime [min]	Type	Width [min]	Area mAU *s	Height [mAU]	Area %
1	9.020	VB	0.2267	21.98778	1.44772	0.5486
2	23.434	BB	0.4289	3986.19263	144.78496	99.4514

Figure S21. HPLC of (*S*)-2b, related to Table 2.

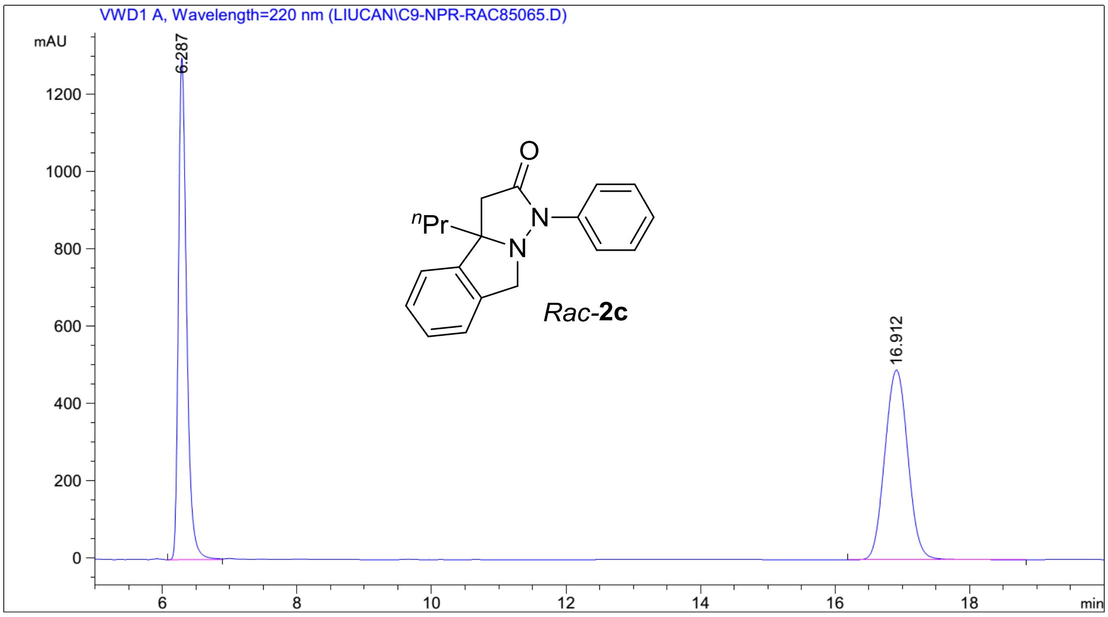

Peak #	RetTime [min]	Type	Width [min]	Area mAU *s	Height [mAU]	Area %
1	6.287	BV	0.1307	1.09849e4	1299.38330	48.4757
2	16.912	BB	0.3779	1.16757e4	491.01810	51.5243

Figure S22. HPLC of *Rac*-2c, related to Table 2.

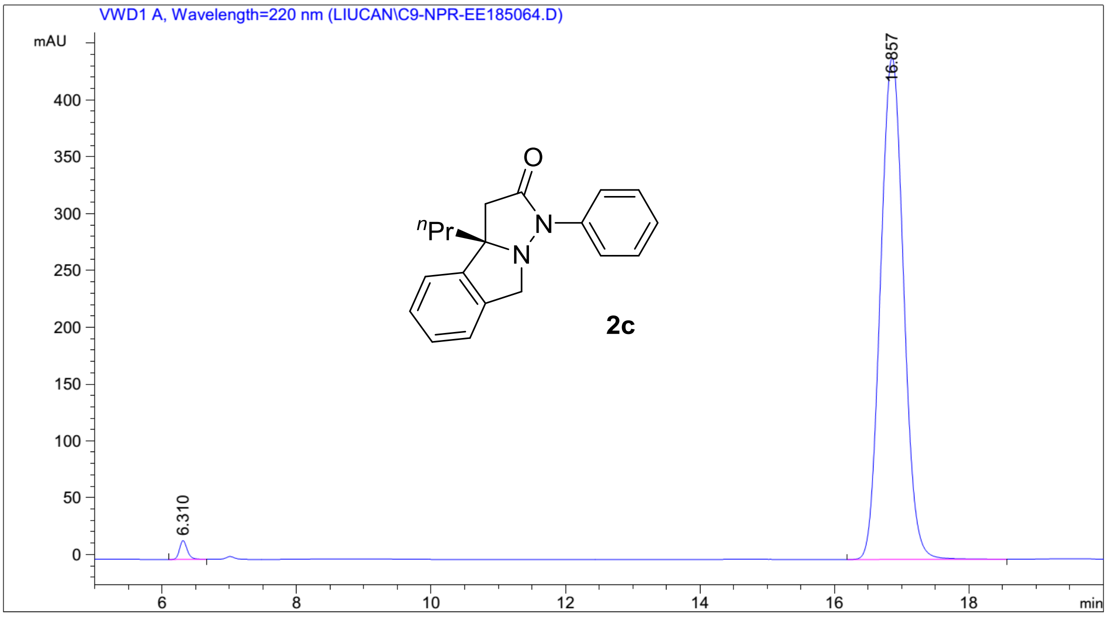

Peak #	RetTime [min]	Type	Width [min]	Area mAU *s	Height [mAU]	Area %
1	6.310	BB	0.1242	132.83827	16.55860	1.2472
2	16.857	BB	0.3770	1.05177e4	441.34576	98.7528

Figure S23. HPLC of (*S*)-2c, related to Table 2.

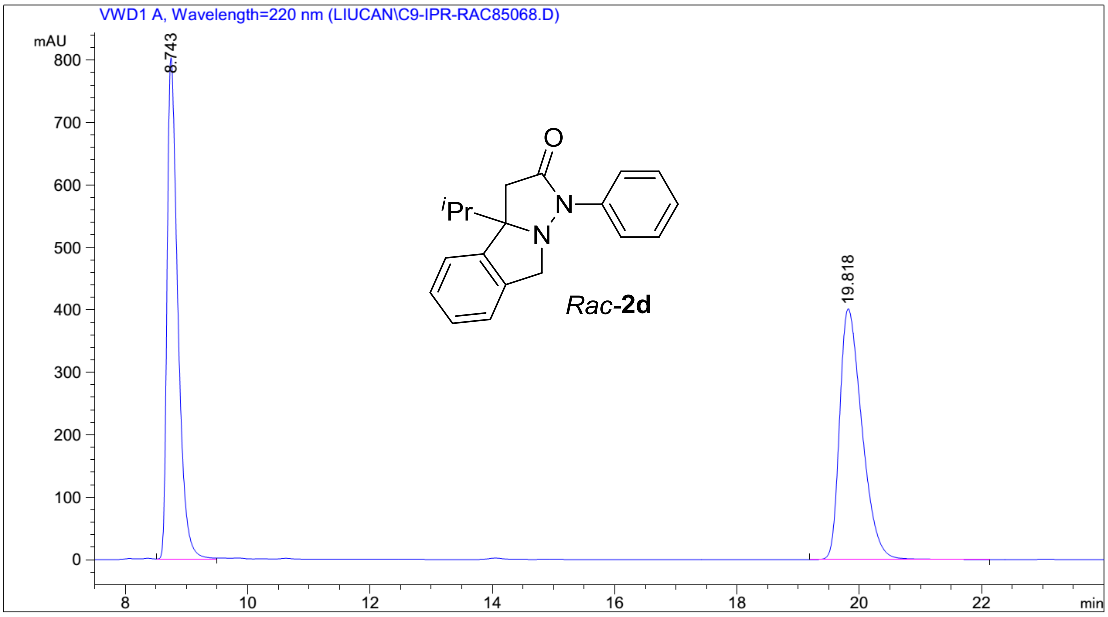

Peak #	RetTime [min]	Type	Width [min]	Area mAU *s	Height [mAU]	Area %
1	8.743	BV	0.1847	9805.22168	802.93268	49.1233
2	19.818	BB	0.3899	1.01552e4	401.31204	50.8767

Figure S24. HPLC of *Rac*-2d, related to Table 2.

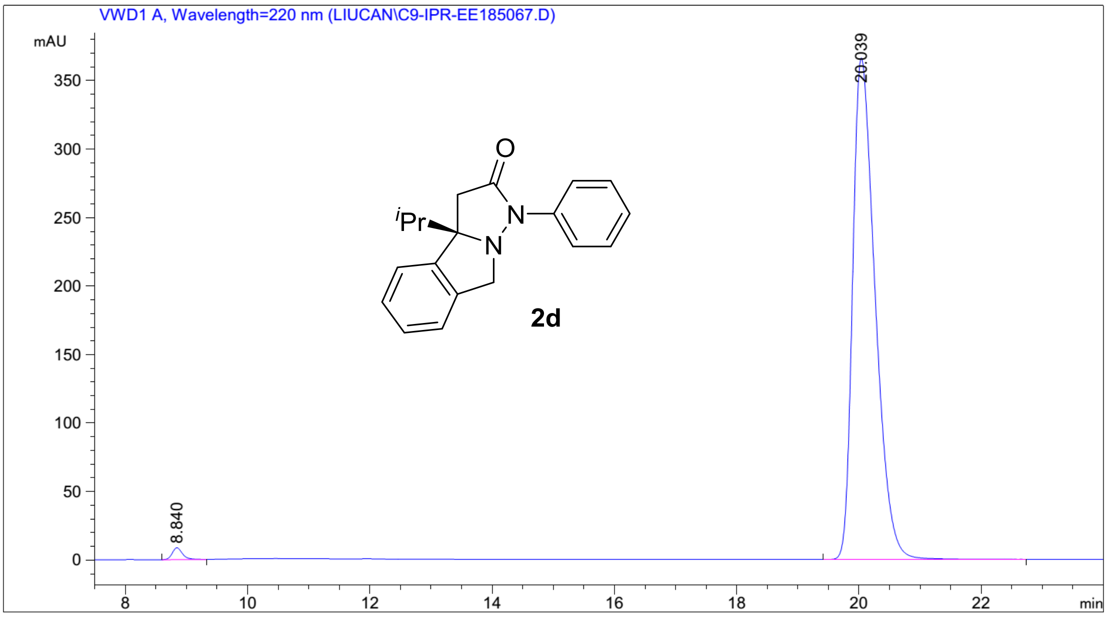

Peak #	RetTime [min]	Type	Width [min]	Area mAU *s	Height [mAU]	Area %
1	8.840	BV	0.1784	100.66415	8.62471	1.0669
2	20.039	BB	0.3934	9334.23828	366.26318	98.9331

Figure S25. HPLC of (*S*)-2d, related to Table 2.

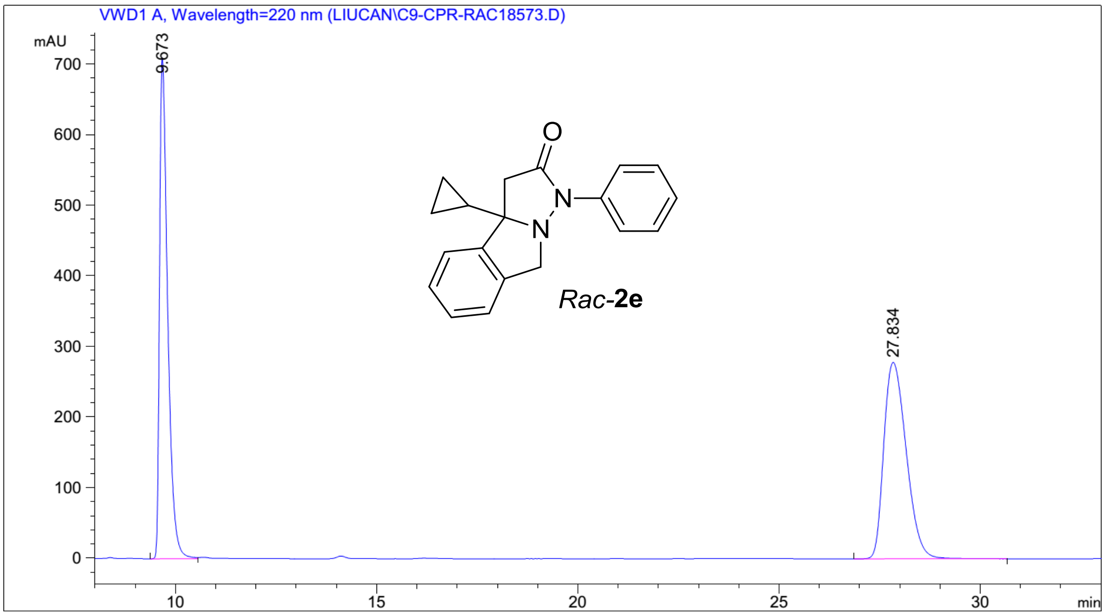

Peak #	RetTime [min]	Type	Width [min]	Area mAU *s	Height [mAU]	Area %
1	9.673	BV	0.2119	9961.88477	709.32764	48.0556
2	27.834	BB	0.6147	1.07680e4	278.16705	51.9444

Figure S26. HPLC of *Rac*-2e, related to Table 2.

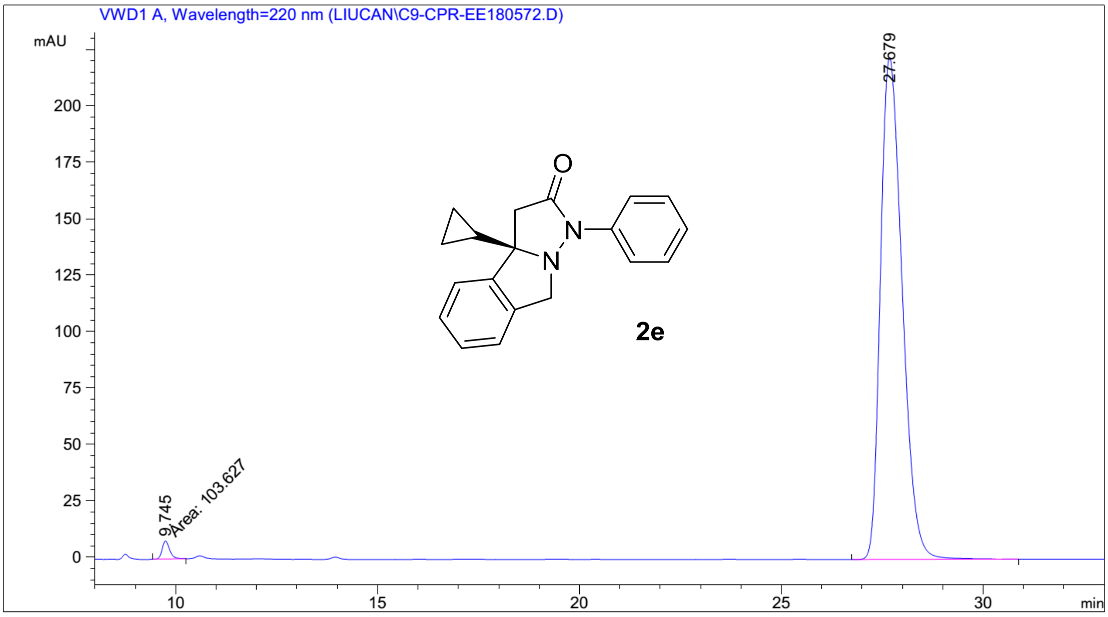

Peak #	RetTime [min]	Type	Width [min]	Area mAU *s	Height [mAU]	Area %
1	9.745	MM	0.2150	103.62657	8.03235	1.2309
2	27.679	BB	0.5878	8315.21777	222.32358	98.7691

Figure S27. HPLC of (*S*)-2e, related to Table 2.

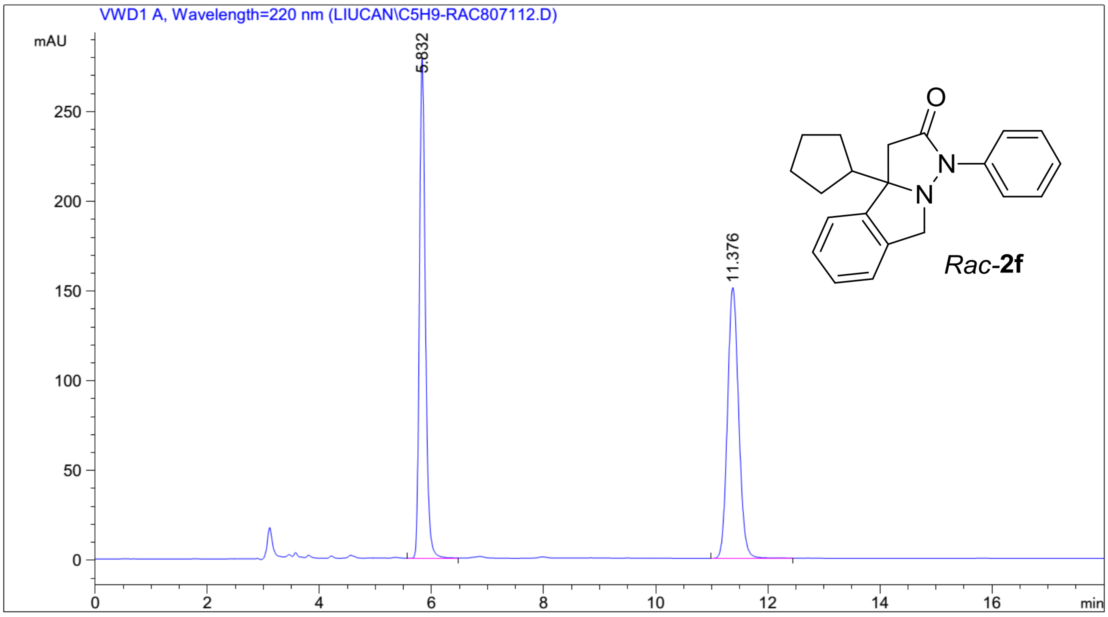

Peak #	RetTime [min]	Type	Width [min]	Area mAU *s	Height [mAU]	Area %
1	5.832	BV	0.1122	2057.15210	279.11142	49.6313
2	11.376	BB	0.2152	2087.71753	150.97113	50.3687

Figure S28. HPLC of *Rac*-2b, related to Table 2.

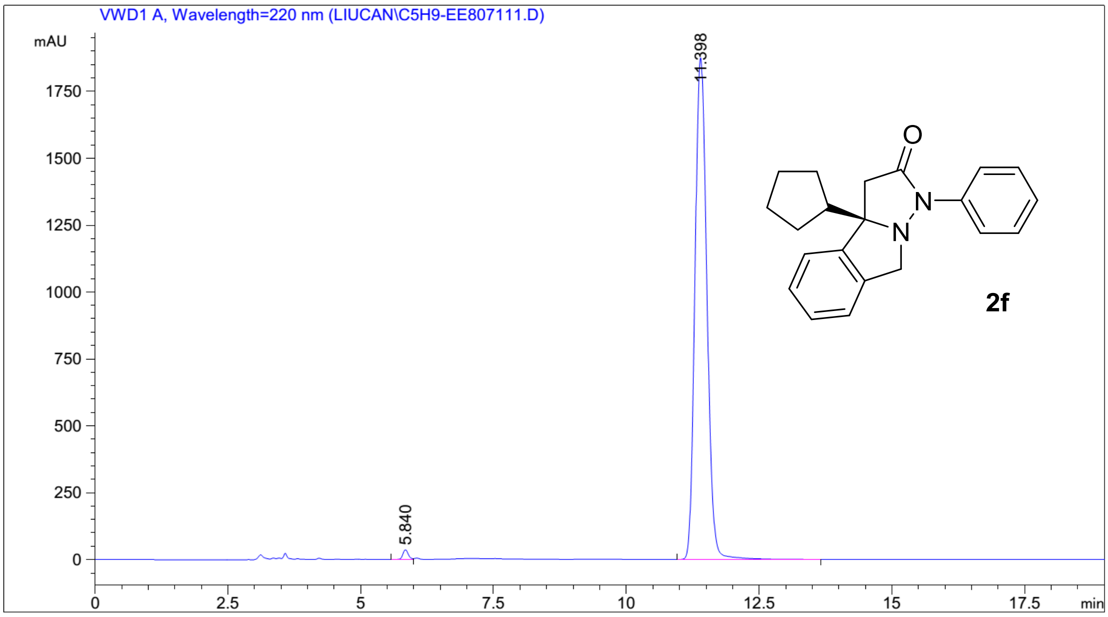

Peak #	RetTime [min]	Type	Width [min]	Area mAU *s	Height [mAU]	Area %
1	5.840	BV	0.1112	264.65854	36.34045	0.8958
2	11.398	BB	0.2463	2.92813e4	1874.67334	99.1042

Figure S29. HPLC of (*S*)-2f, related to Table 2.

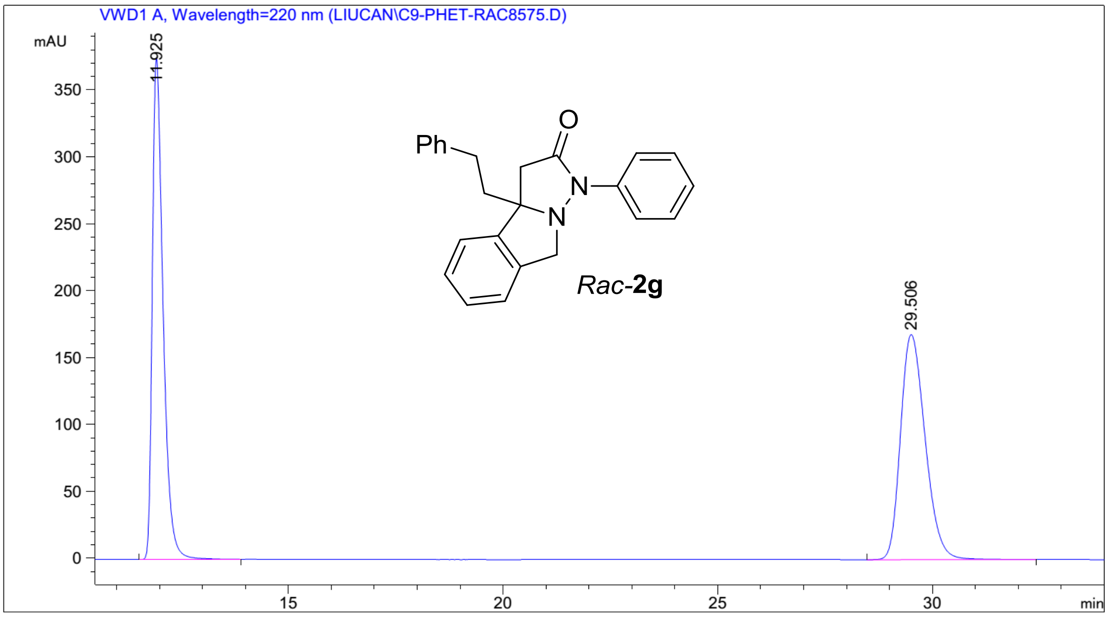

Peak #	RetTime [min]	Type	Width [min]	Area mAU *s	Height [mAU]	Area %
1	11.925	BB	0.2647	6541.70215	374.91364	49.3920
2	29.506	BB	0.6237	6702.75586	168.16367	50.6080

Figure S30. HPLC of *Rac*-2g, related to Table 2.

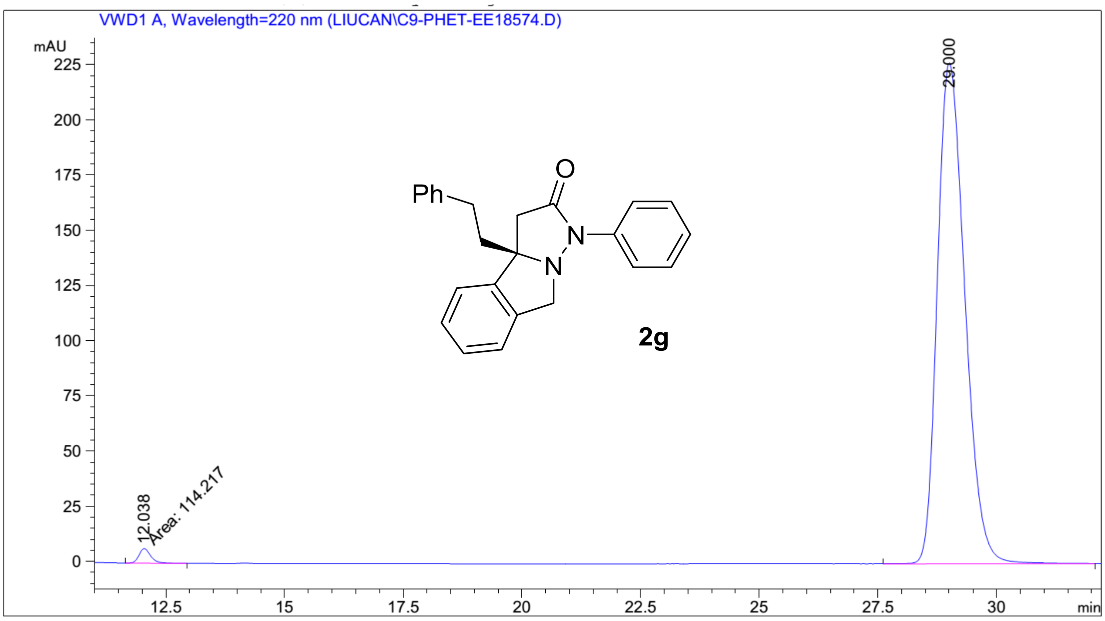

Peak #	RetTime [min]	Type	Width [min]	Area mAU *s	Height [mAU]	Area %
1	12.038	MM	0.2874	114.21745	6.62457	1.2368
2	29.000	BB	0.6294	9120.94824	226.74910	98.7632

Figure S31. HPLC of (*S*)-2g, related to Table 2.

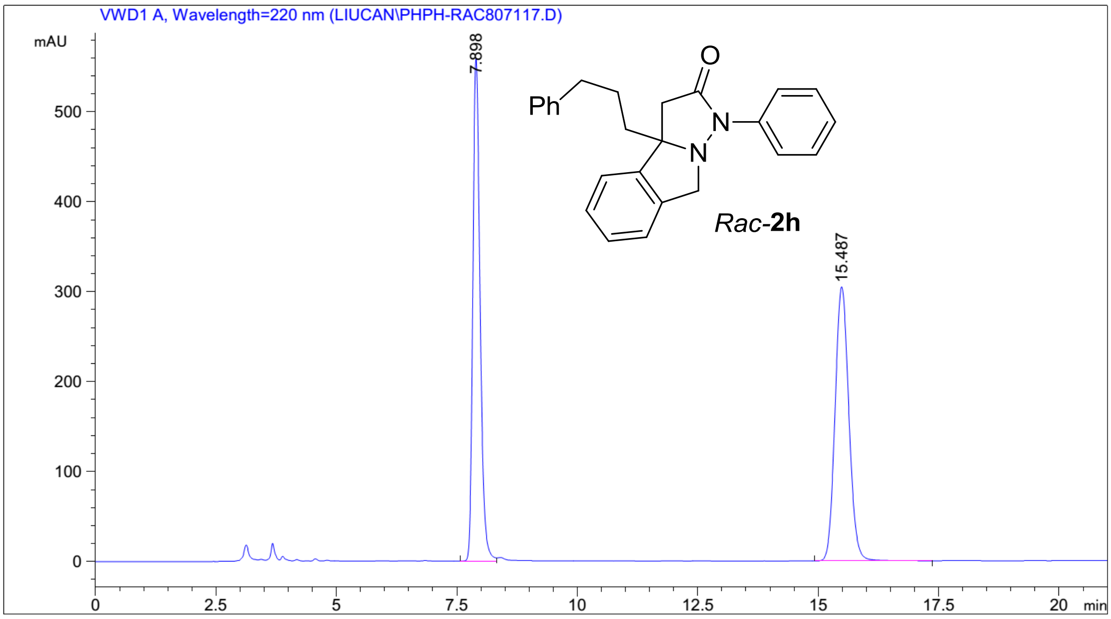

Peak #	RetTime [min]	Type	Width [min]	Area mAU *s	Height [mAU]	Area %
1	7.898	BV	0.1627	5846.34277	559.28894	49.0175
2	15.487	BB	0.3115	6080.70947	304.67856	50.9825

Figure S32. HPLC of *Rac*-2h, related to Table 2.

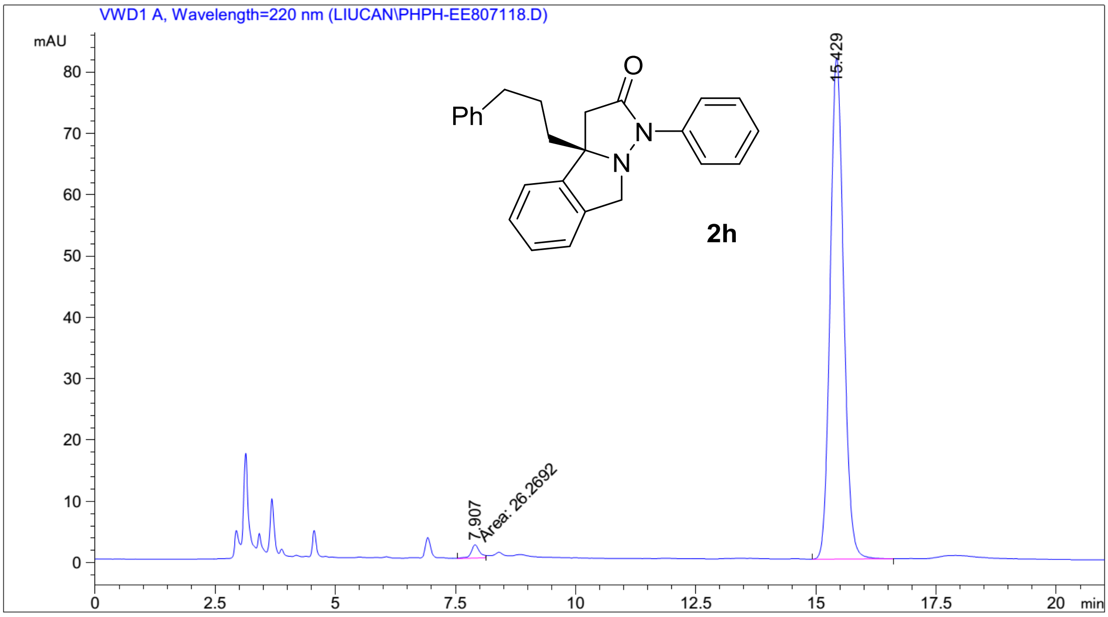

Peak #	RetTime [min]	Type	Width [min]	Area mAU *s	Height [mAU]	Area %
1	7.907	MM	0.2030	26.26925	2.15662	1.6224
2	15.429	BB	0.3016	1592.84412	81.74945	98.3776

Figure S33. HPLC of (*S*)-2h, related to Table 2.

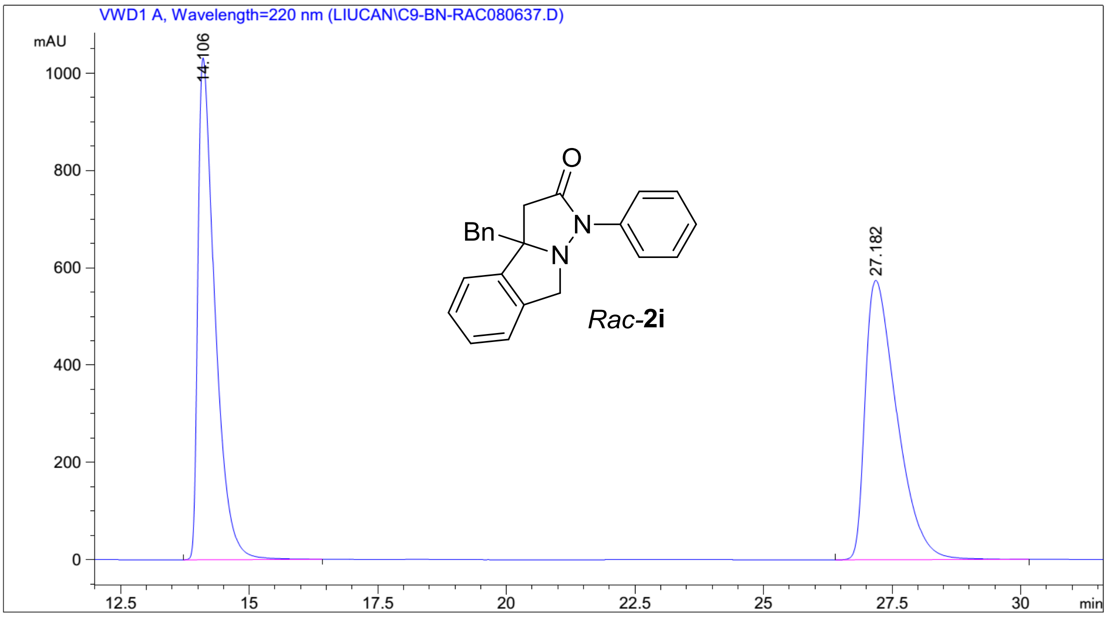

Peak #	RetTime [min]	Type	Width [min]	Area mAU *s	Height [mAU]	Area %
1	14.106	BB	0.3429	2.37481e4	1031.33533	49.1815
2	27.182	BB	0.6500	2.45386e4	574.24286	50.8185

Figure S34. HPLC of *Rac*-2i, related to Table 2.

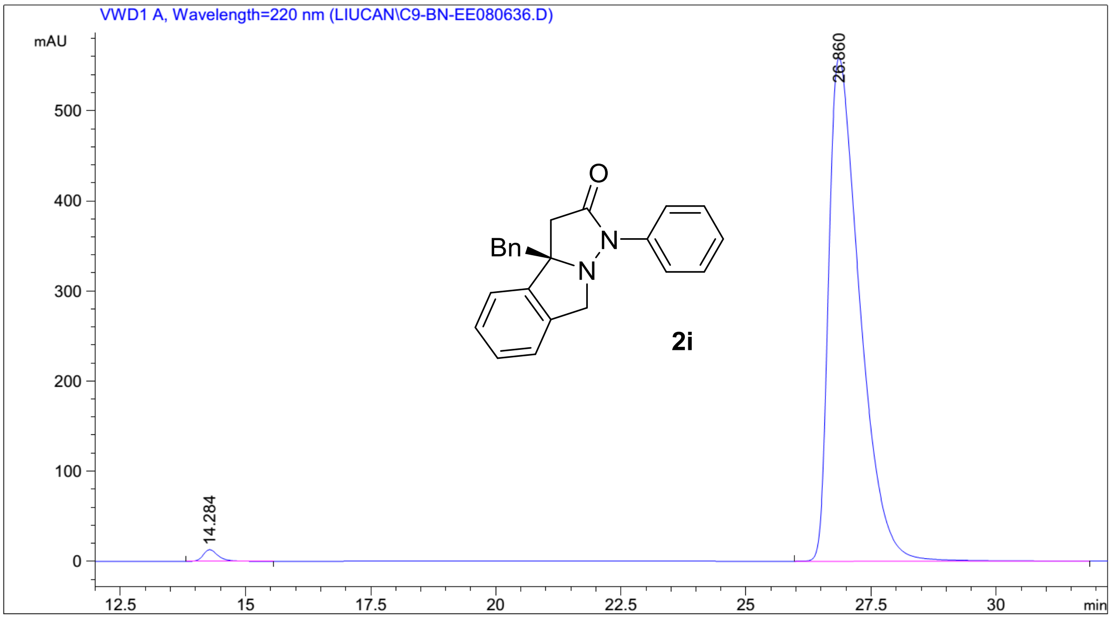

Peak #	RetTime [min]	Type	Width [min]	Area mAU *s	Height [mAU]	Area %
1	14.284	BB	0.3188	269.52255	12.85985	1.1089
2	26.860	BB	0.6550	2.40369e4	558.52997	98.8911

Figure S35. HPLC of (*S*)-2i, related to Table 2.

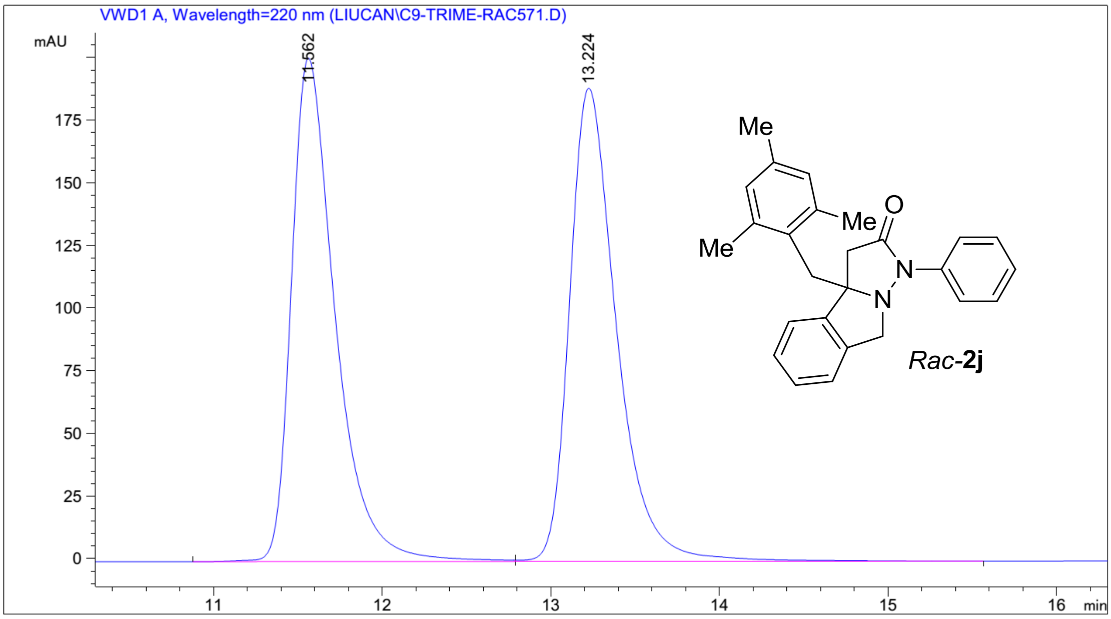

Peak #	RetTime [min]	Type	Width [min]	Area mAU *s	Height [mAU]	Area %
1	11.562	BV	0.2716	3625.66699	200.95407	49.7994
2	13.224	VB	0.2942	3654.87915	188.88303	50.2006

Figure S36. HPLC of *Rac*-2j, related to Table 2.

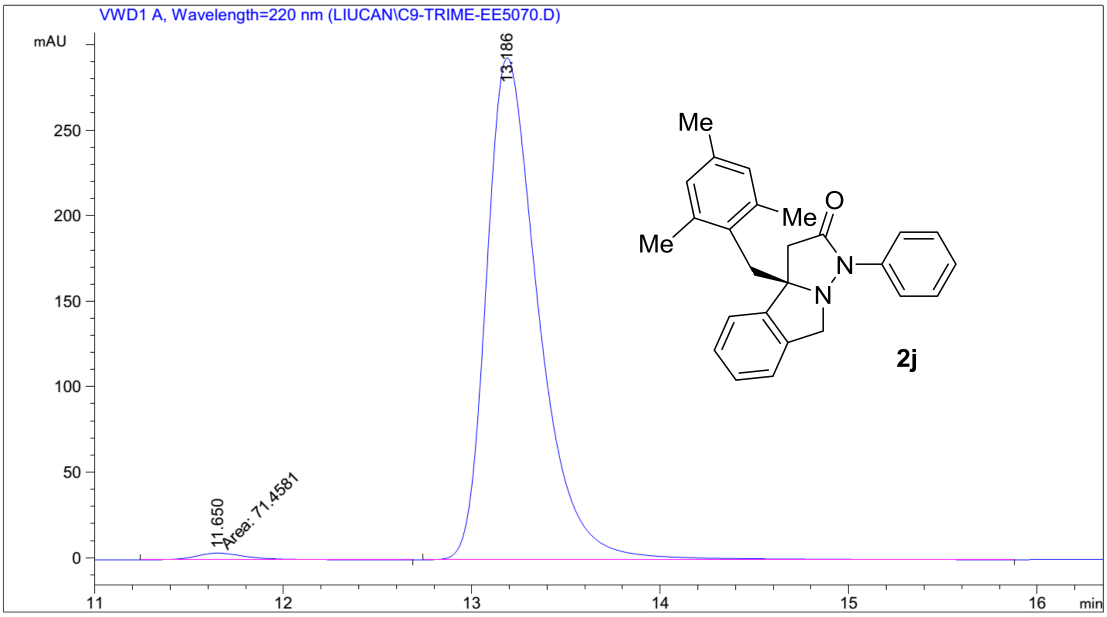

Peak #	RetTime [min]	Type	Width [min]	Area mAU *s	Height [mAU]	Area %
1	11.650	MM	0.3094	71.45806	3.84900	1.2477
2	13.186	BB	0.2918	5655.90186	293.49121	98.7523

Figure S37. HPLC of (*S*)-2j, related to Table 2.

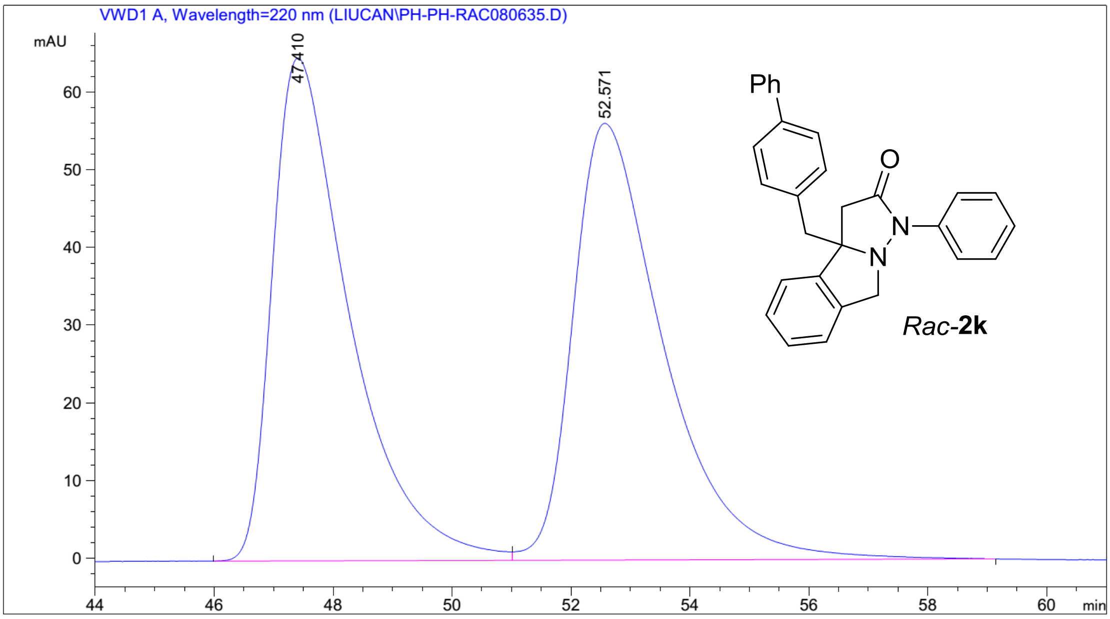

Peak #	RetTime [min]	Type	Width [min]	Area mAU *s	Height [mAU]	Area %
1	47.410	BV	1.3291	5851.86084	64.68917	49.5328
2	52.571	VB	1.5352	5962.24805	56.18066	50.4672

Figure S38. HPLC of *Rac*-2k, related to Table 2.

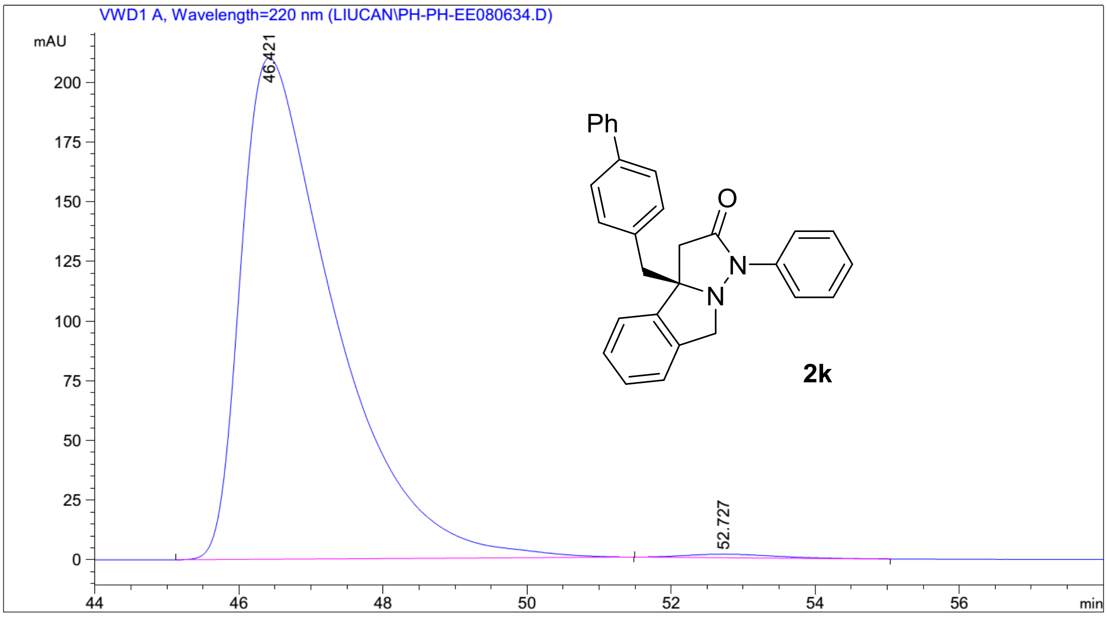

Peak #	RetTime [min]	Type	Width [min]	Area mAU *s	Height [mAU]	Area %
1	46.421	BB	1.3191	1.90242e4	209.98152	99.3489
2	52.727	BB	1.0040	124.67218	1.46790	0.6511

Figure S39. HPLC of (*S*)-2k, related to Table 2.

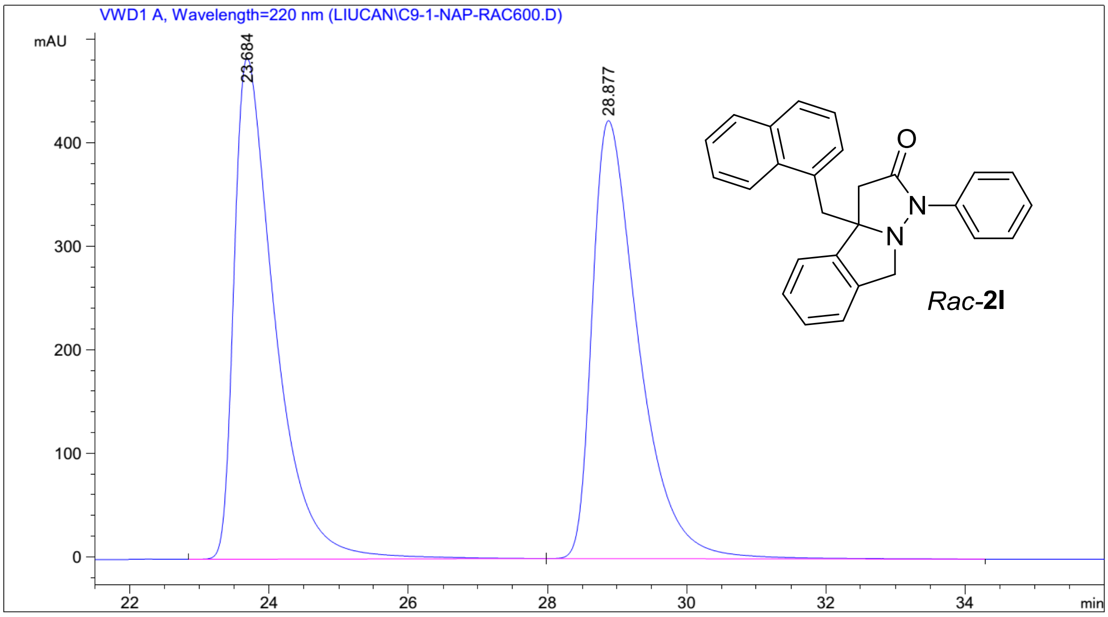

Peak #	RetTime [min]	Type	Width [min]	Area mAU *s	Height [mAU]	Area %
1	23.684	BB	0.6053	1.96760e4	484.05600	50.1408
2	28.877	BB	0.6941	1.95654e4	422.89490	49.8592

Figure S40. HPLC of *Rac*-2l, related to Table 2.

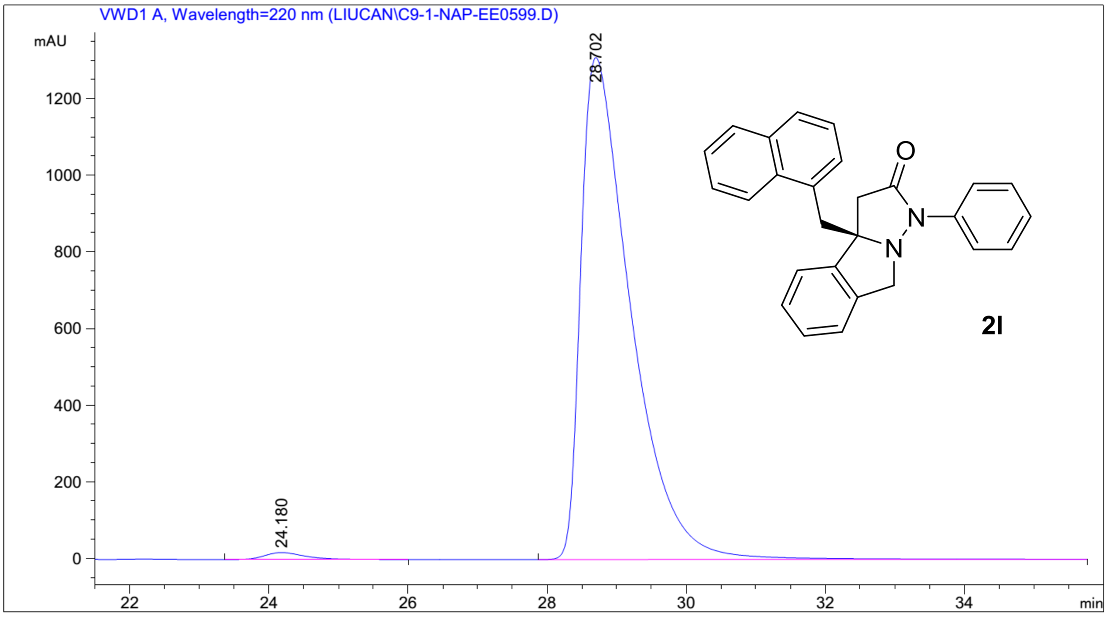

Peak #	RetTime [min]	Type	Width [min]	Area mAU *s	Height [mAU]	Area %
1	24.180	BB	0.6072	728.69769	18.13118	1.1029
2	28.702	BBA	0.7408	6.53412e4	1309.57288	98.8971

Figure S41. HPLC of (*S*)-2l, related to Table 2.

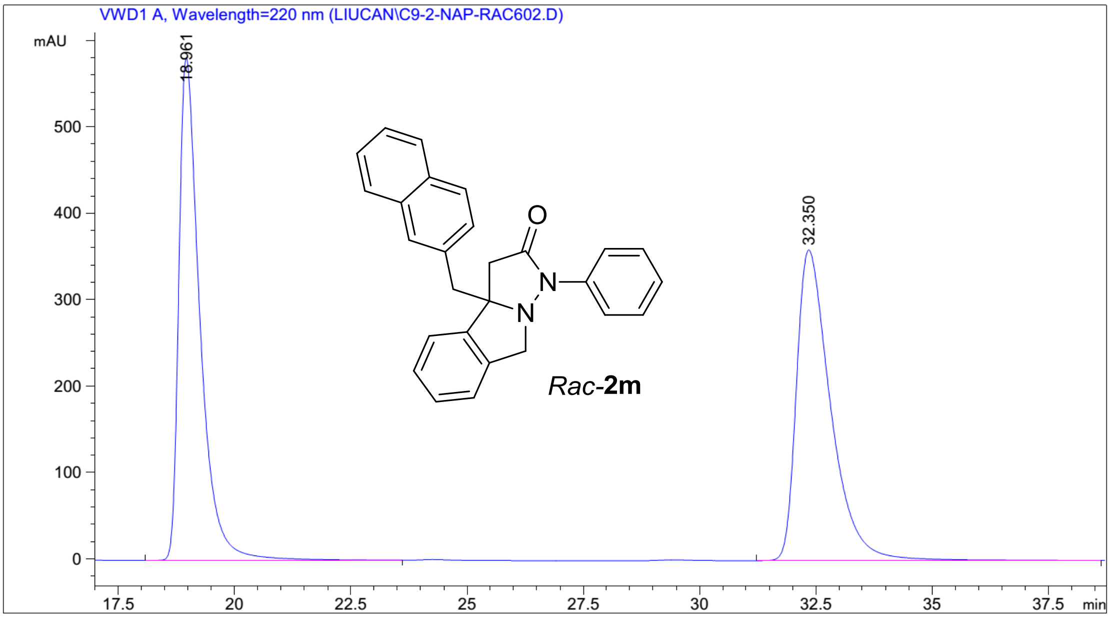

Peak #	RetTime [min]	Type	Width [min]	Area mAU *s	Height [mAU]	Area %
1	18.961	BB	0.4718	1.83670e4	581.62030	49.9865
2	32.350	BBA	0.7698	1.83769e4	359.38956	50.0135

Figure S42. HPLC of *Rac*-2m, related to Table 2.

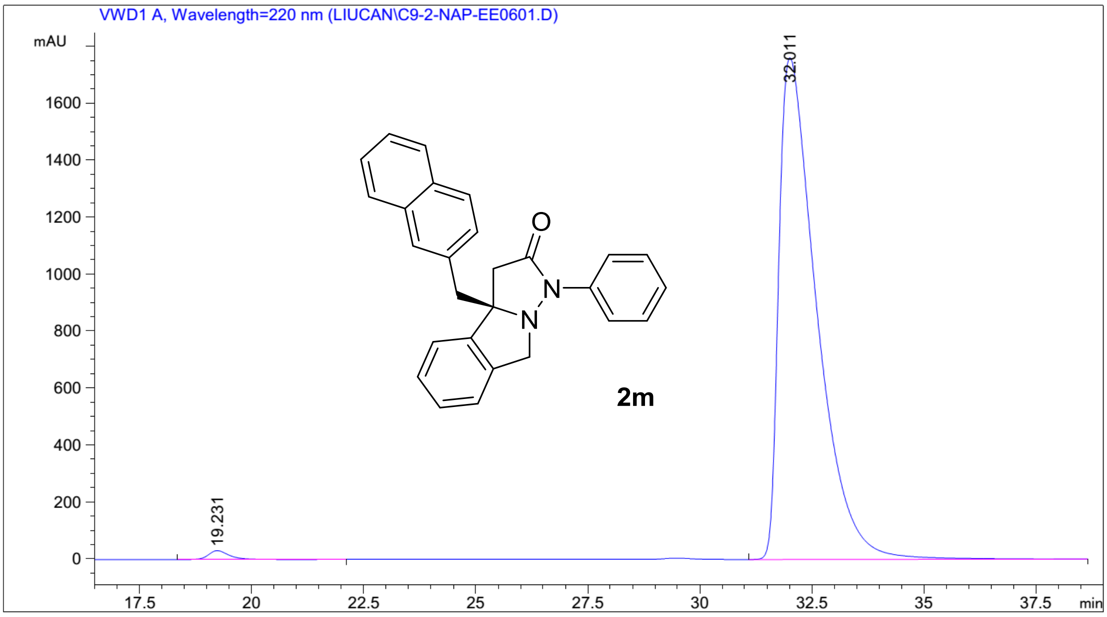

Peak #	RetTime [min]	Type	Width [min]	Area mAU *s	Height [mAU]	Area %
1	19.231	BB	0.4943	1033.42554	31.18626	0.9931
2	32.011	BBA	0.8819	1.03028e5	1761.29675	99.0069

Figure S43. HPLC of (*S*)-2m, related to Table 2.

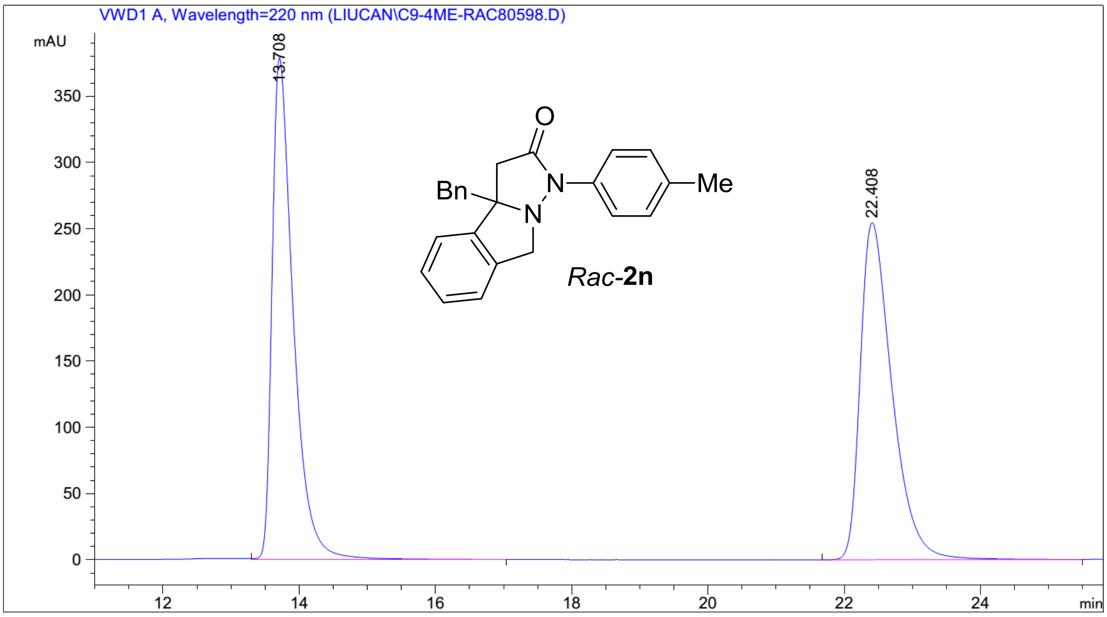

Peak #	RetTime [min]	Type	Width [min]	Area mAU *s	Height [mAU]	Area %
1	13.708	VB	0.3234	8185.55273	378.81033	49.8499
2	22.408	BB	0.4924	8234.83594	254.54823	50.1501

Figure S44. HPLC of *Rac*-2n, related to Table 2.

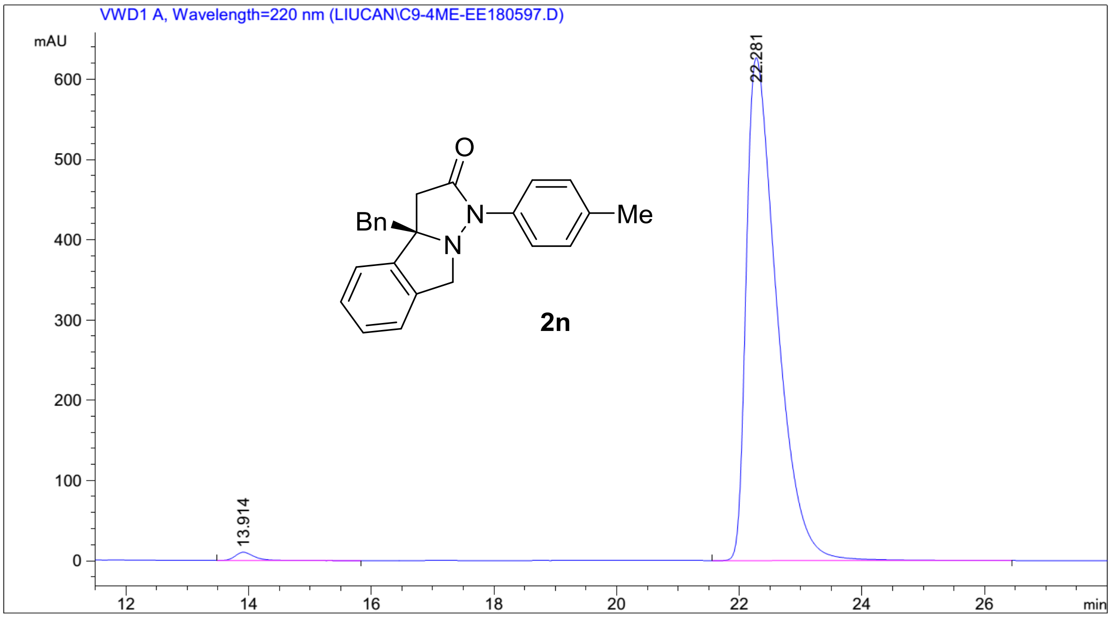

Peak #	RetTime [min]	Type	Width [min]	Area mAU *s	Height [mAU]	Area %
1	13.914	BB	0.3304	224.58611	10.28644	1.0282
2	22.281	BB	0.5220	2.16174e4	626.47821	98.9718

Figure S45. HPLC of (*S*)-2n, related to Table 2.

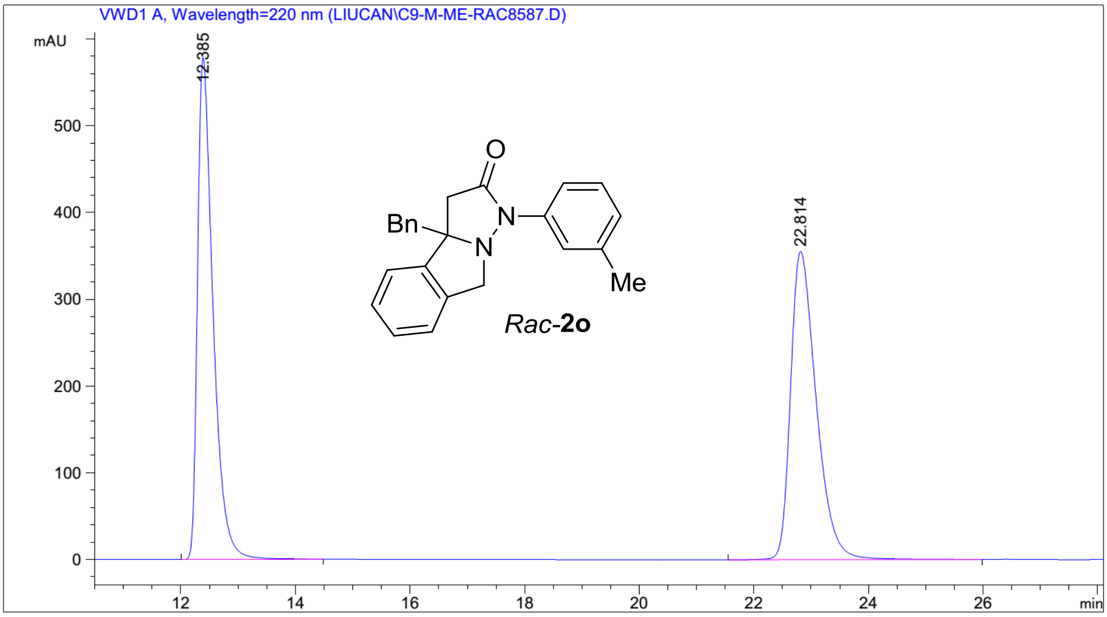

Peak #	RetTime [min]	Type	Width [min]	Area mAU *s	Height [mAU]	Area %
1	12.385	BB	0.2836	1.08896e4	578.61523	49.6448
2	22.814	BB	0.4747	1.10455e4	355.35995	50.3552

Figure S46. HPLC of *Rac*-2o, related to Table 2.

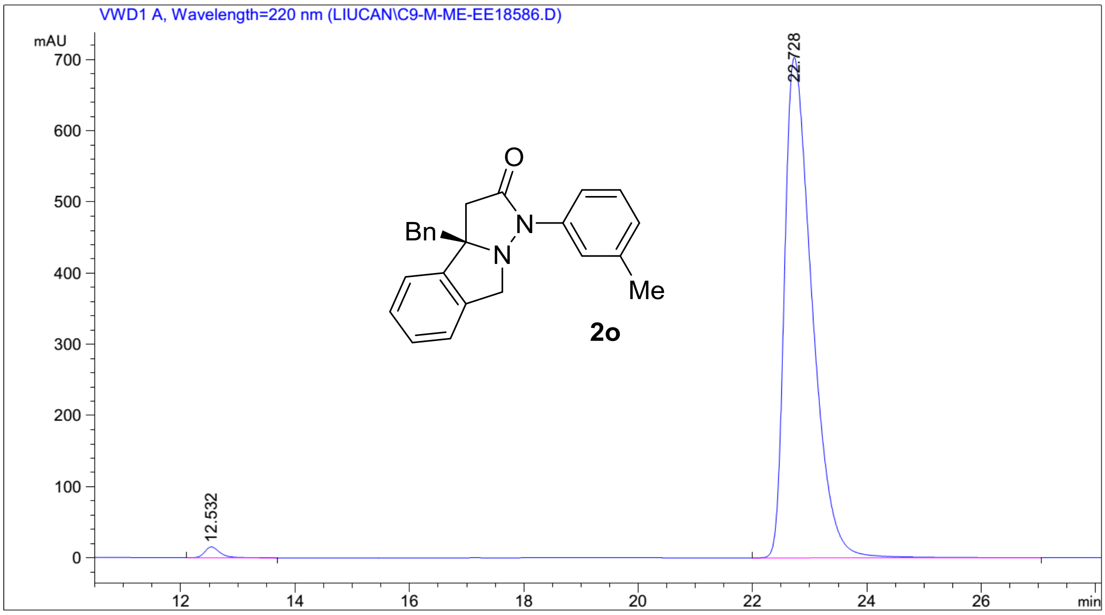

Peak #	RetTime [min]	Type	Width [min]	Area mAU *s	Height [mAU]	Area %
1	12.532	BB	0.2826	281.82867	15.14880	1.1925
2	22.728	BB	0.5083	2.33514e4	703.06427	98.8075

Figure S47. HPLC of (*S*)-2o, related to Table 2.

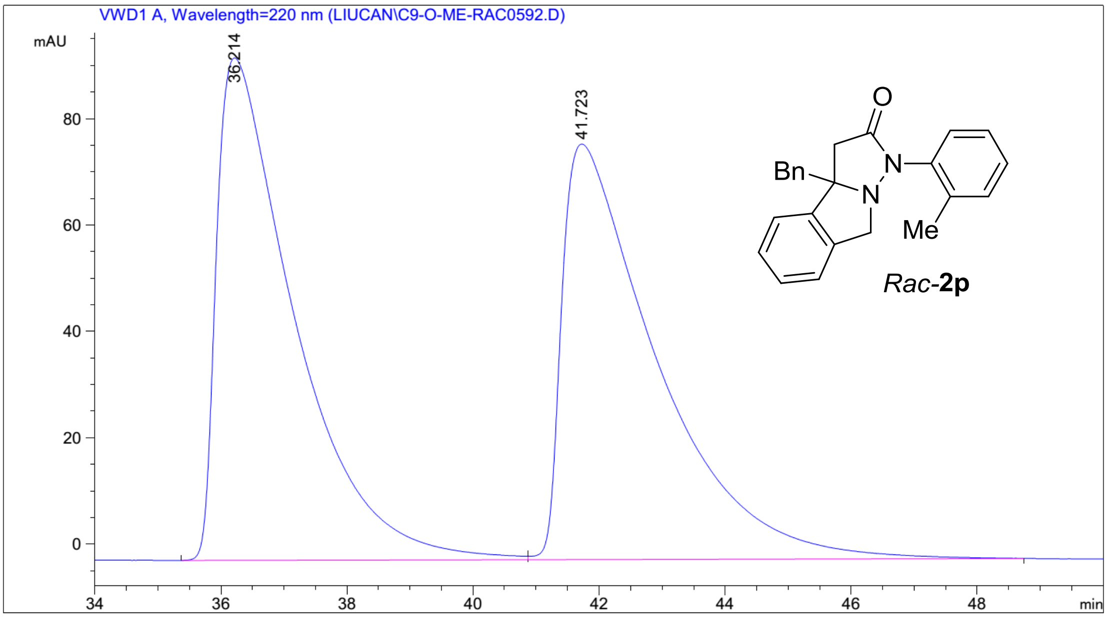

Peak #	RetTime [min]	Type	Width [min]	Area mAU *s	Height [mAU]	Area %
1	36.214	BV	1.2130	8110.57861	94.44979	49.8283
2	41.723	VB	1.4354	8166.46191	78.10155	50.1717

Figure S48. HPLC of *Rac*-2p, related to Table 2.

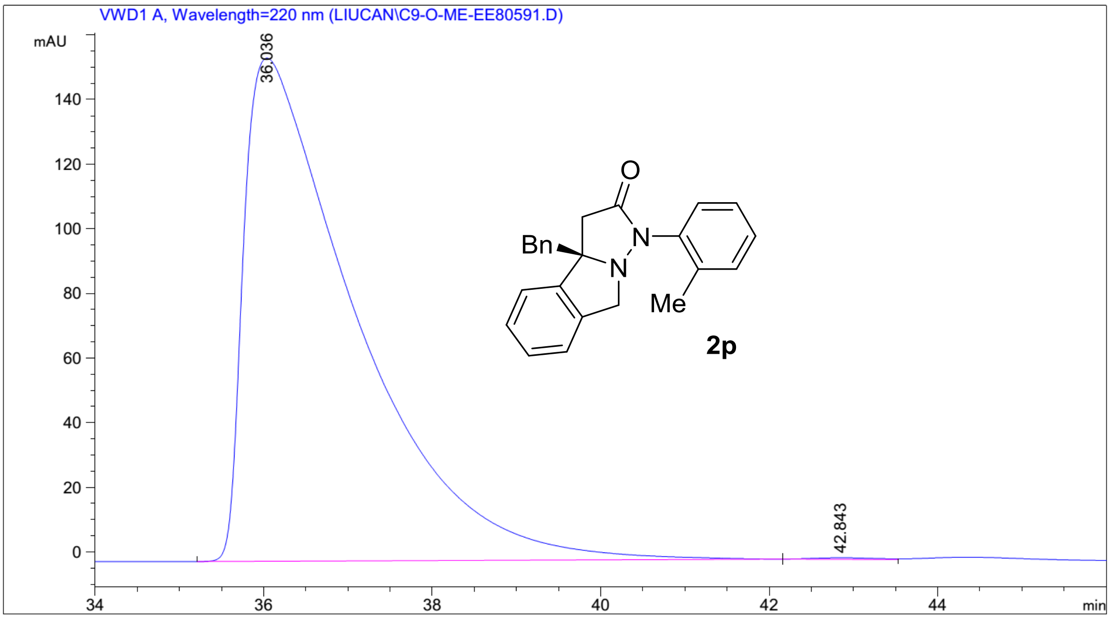

Peak #	RetTime [min]	Type	Width [min]	Area mAU *s	Height [mAU]	Area %
1	36.036	BB	1.3335	1.44537e4	155.68726	99.8319
2	42.843	BV	0.6311	24.34075	4.57325e-1	0.1681

Figure S49. HPLC of (*S*)-2p, related to Table 2.

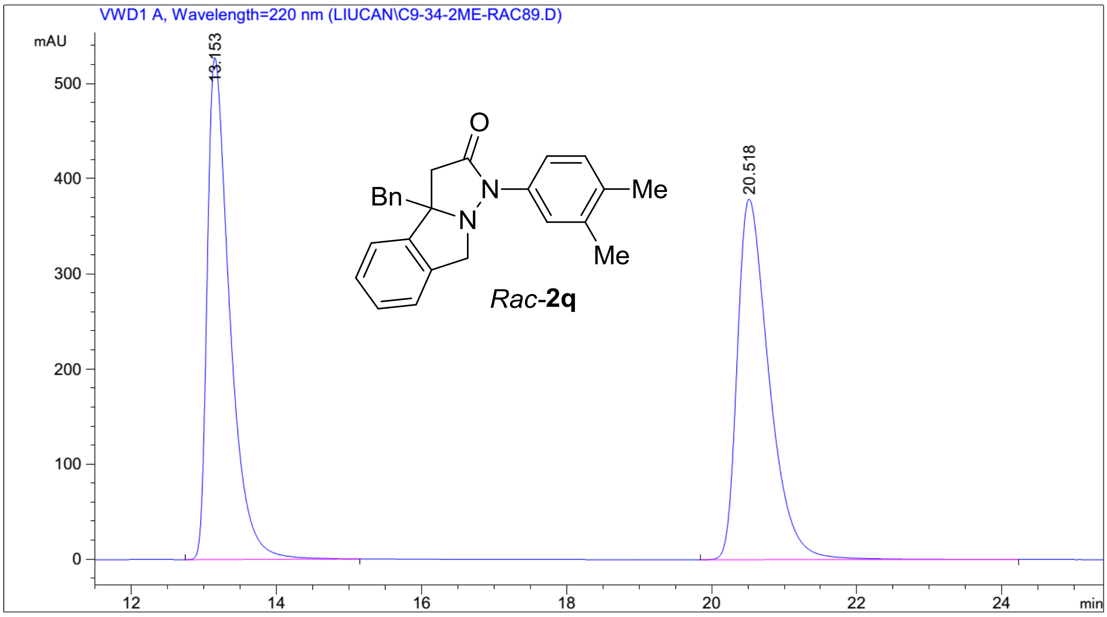

Peak #	RetTime [min]	Type	Width [min]	Area mAU *s	Height [mAU]	Area %
1	13.153	BB	0.3230	1.13799e4	527.50494	49.6336
2	20.518	BB	0.4618	1.15479e4	378.99115	50.3664

Figure S50. HPLC of *Rac*-2q, related to Table 2.

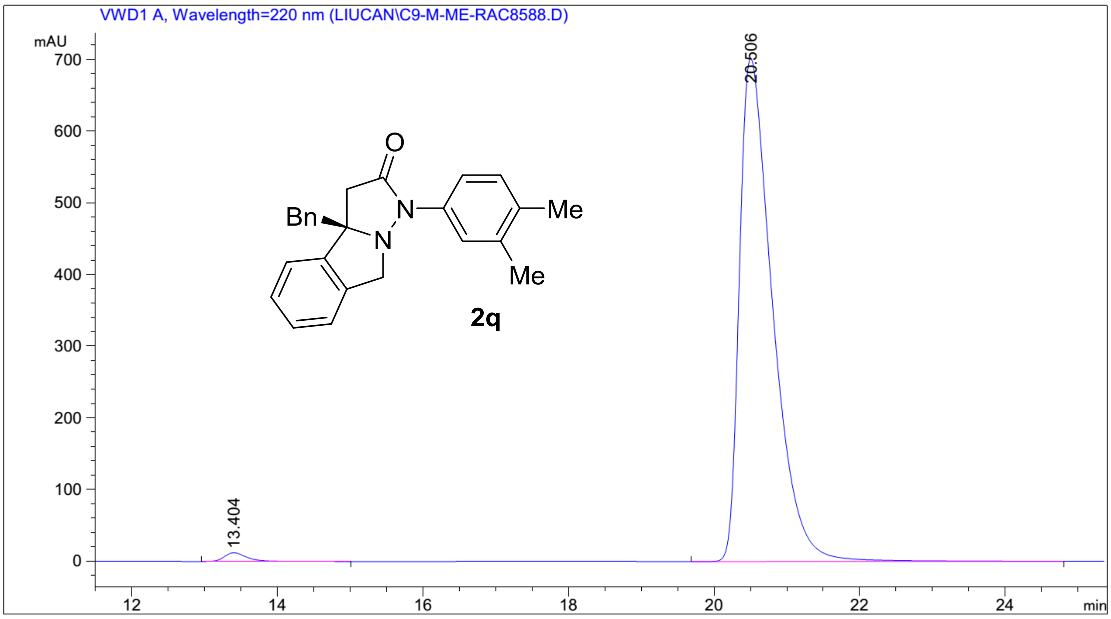

Peak #	RetTime [min]	Type	Width [min]	Area mAU *s	Height [mAU]	Area %
1	13.404	BB	0.3276	265.64832	12.16198	1.1817
2	20.506	BB	0.4796	2.22140e4	702.40350	98.8183

Figure S51. HPLC of (*S*)-2q, related to Table 2.

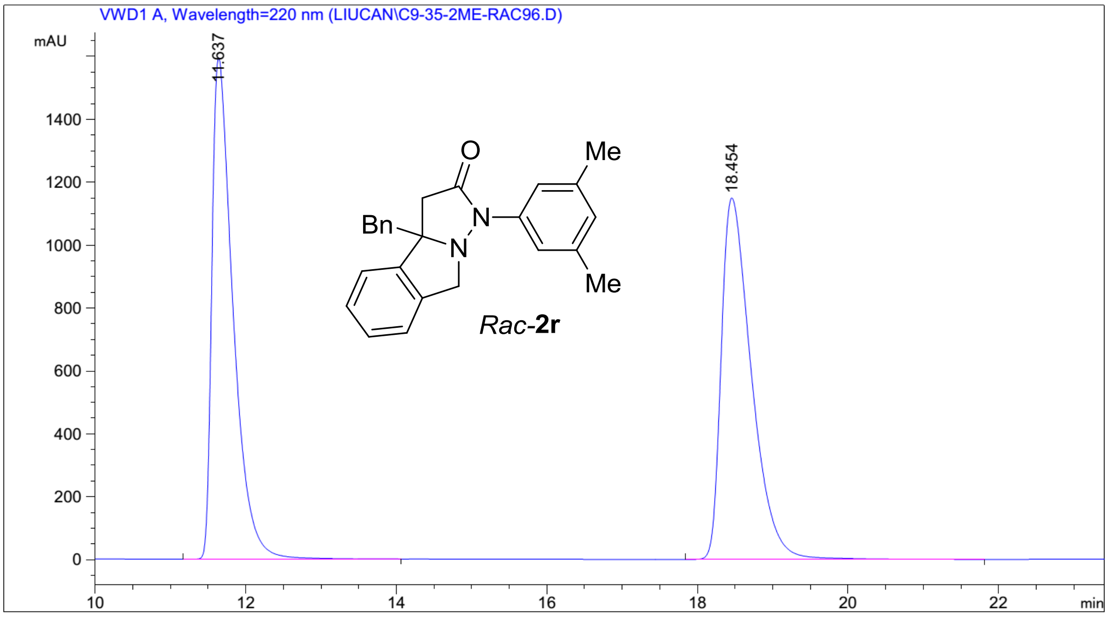

Peak #	RetTime [min]	Type	Width [min]	Area mAU *s	Height [mAU]	Area %
1	11.637	BB	0.2928	3.10593e4	1594.40588	49.3227
2	18.454	BB	0.4230	3.19124e4	1149.20239	50.6773

Figure S52. HPLC of *Rac*-2r, related to Table 2.

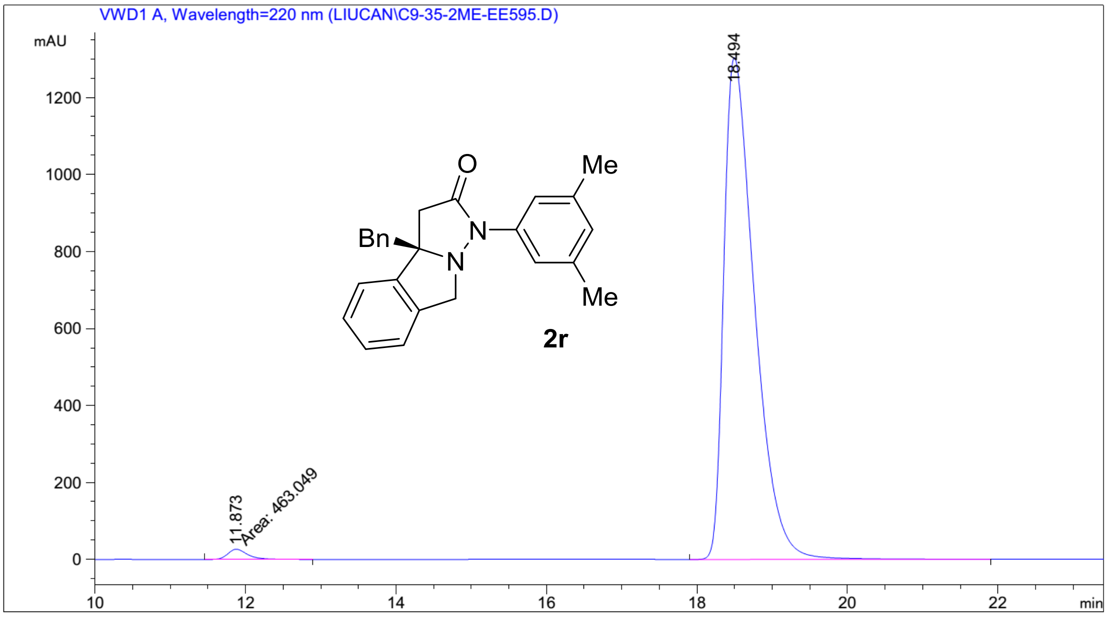

Peak #	RetTime [min]	Type	Width [min]	Area mAU *s	Height [mAU]	Area %
1	11.873	MM	0.2958	463.04886	26.09230	1.2381
2	18.494	BB	0.4325	3.69373e4	1303.53076	98.7619

Figure S53. HPLC of (*S*)-2r, related to Table 2.

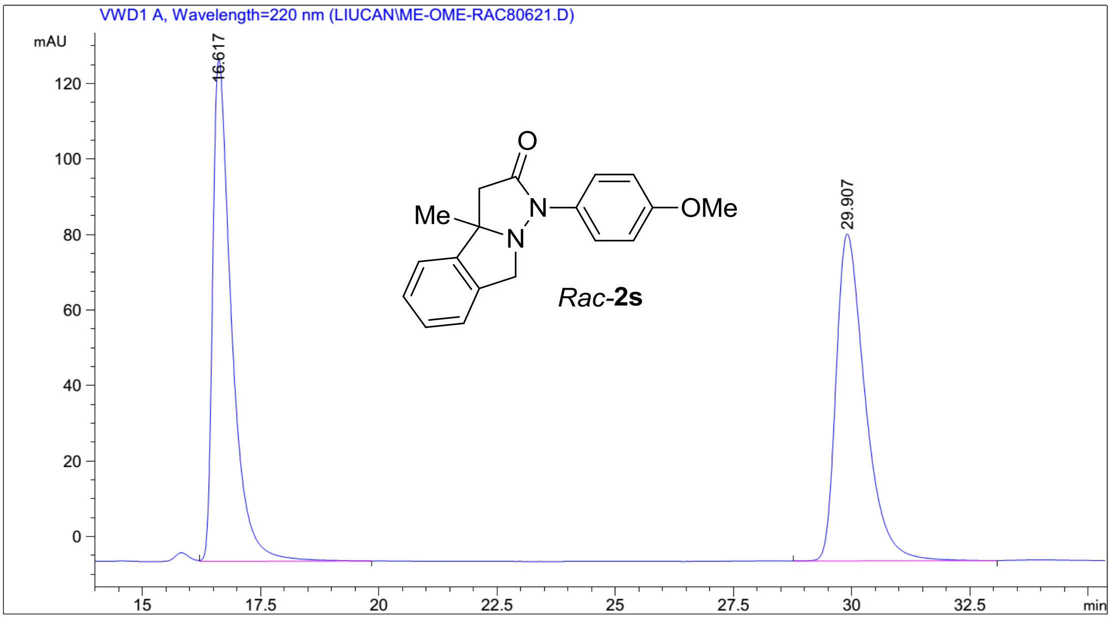

Peak #	RetTime [min]	Type	Width [min]	Area mAU *s	Height [mAU]	Area %
1	16.617	VB	0.4104	3691.99683	133.33612	49.9703
2	29.907	BB	0.6465	3696.38452	86.59996	50.0297

Figure S54. HPLC of *Rac*-2s, related to Table 2.

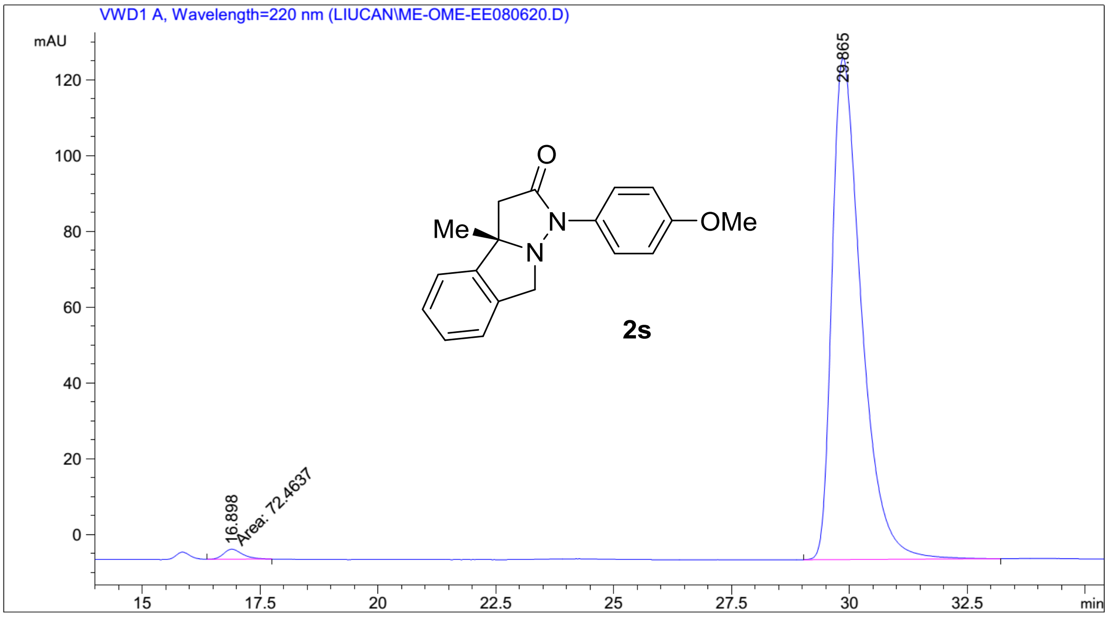

Peak #	RetTime [min]	Type	Width [min]	Area mAU *s	Height [mAU]	Area %
1	16.898	MM	0.4593	72.46369	2.62971	1.2481
2	29.865	BB	0.6595	5733.41797	132.40321	98.7519

Figure S55. HPLC of (*S*)-2s, related to Table 2.

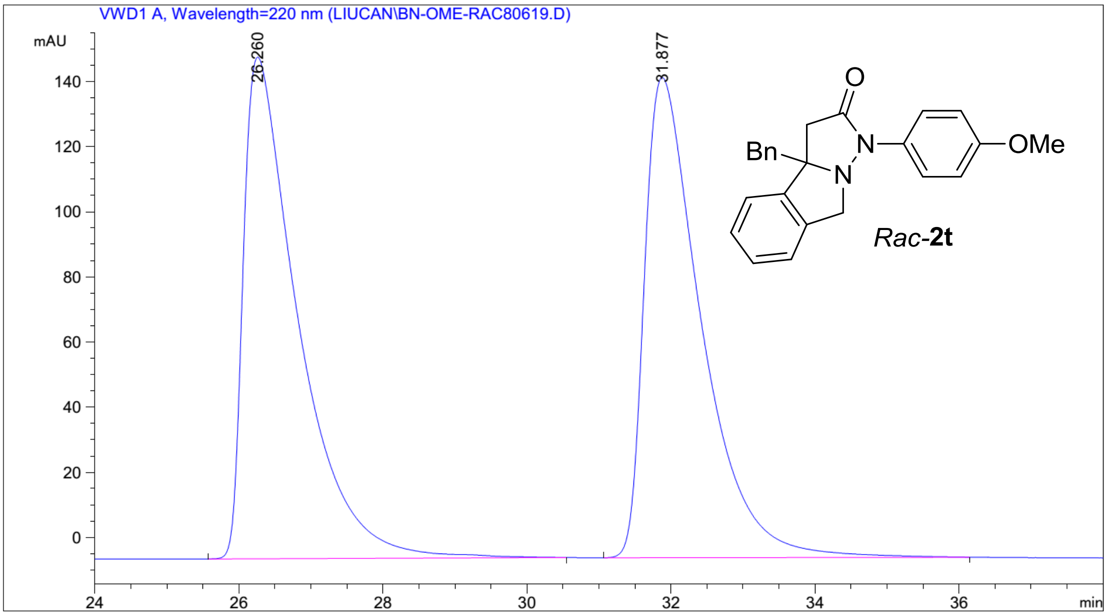

Peak #	RetTime [min]	Type	Width [min]	Area mAU *s	Height [mAU]	Area %
1	26.260	BB	0.7740	8104.12500	154.03474	49.9894
2	31.877	BB	0.8117	8107.55762	147.49445	50.0106

Figure S56. HPLC of *Rac*-2t, related to Table 2.

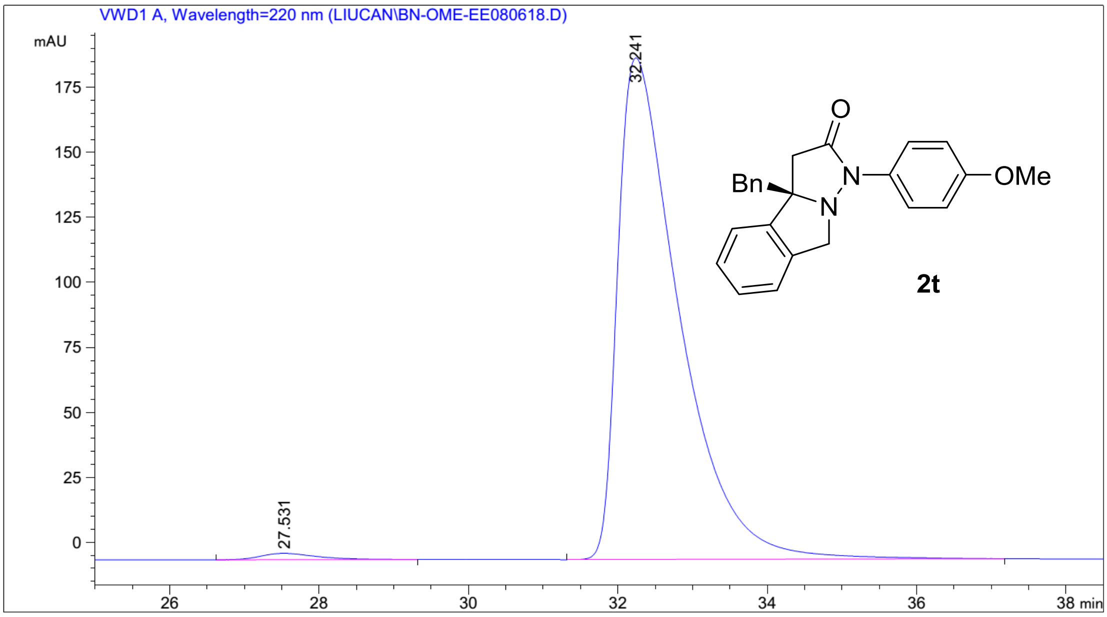

Peak #	RetTime [min]	Type	Width [min]	Area mAU *s	Height [mAU]	Area %
1	27.531	BB	0.6606	127.94970	2.43108	1.1436
2	32.241	BB	0.8437	1.10603e4	192.90025	98.8564

Figure S57. HPLC of (*S*)-2t, related to Table 2.

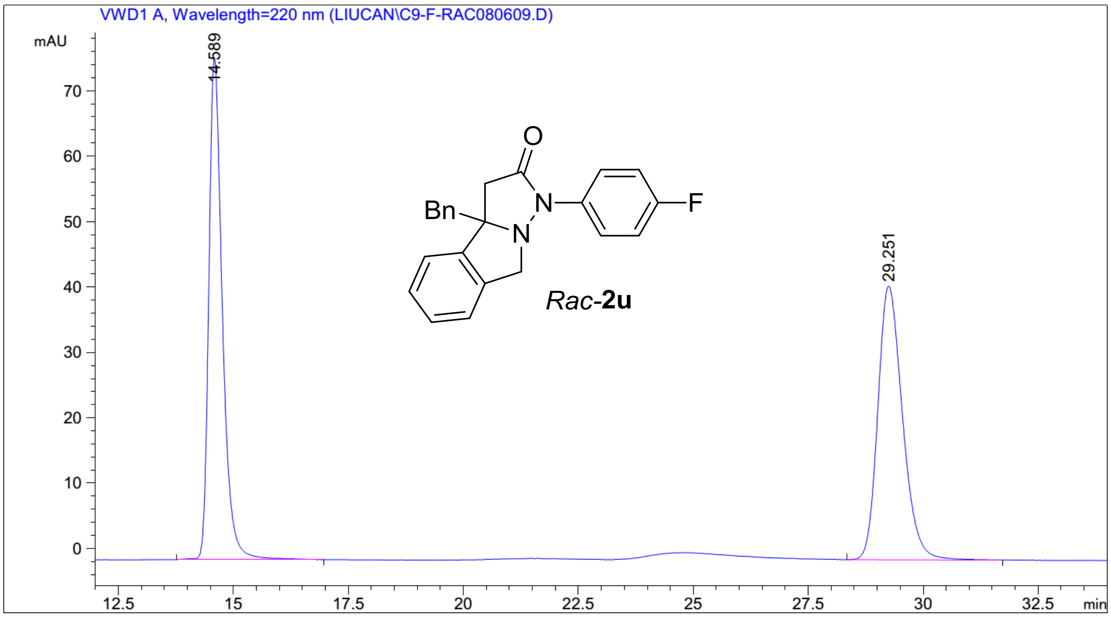

Peak #	RetTime [min]	Type	Width [min]	Area mAU *s	Height [mAU]	Area %
1	14.589	BB	0.3118	1572.08752	76.73154	50.2386
2	29.251	BB	0.5738	1557.15442	41.85207	49.7614

Figure S58. HPLC of *Rac*-2u, related to Table 2.

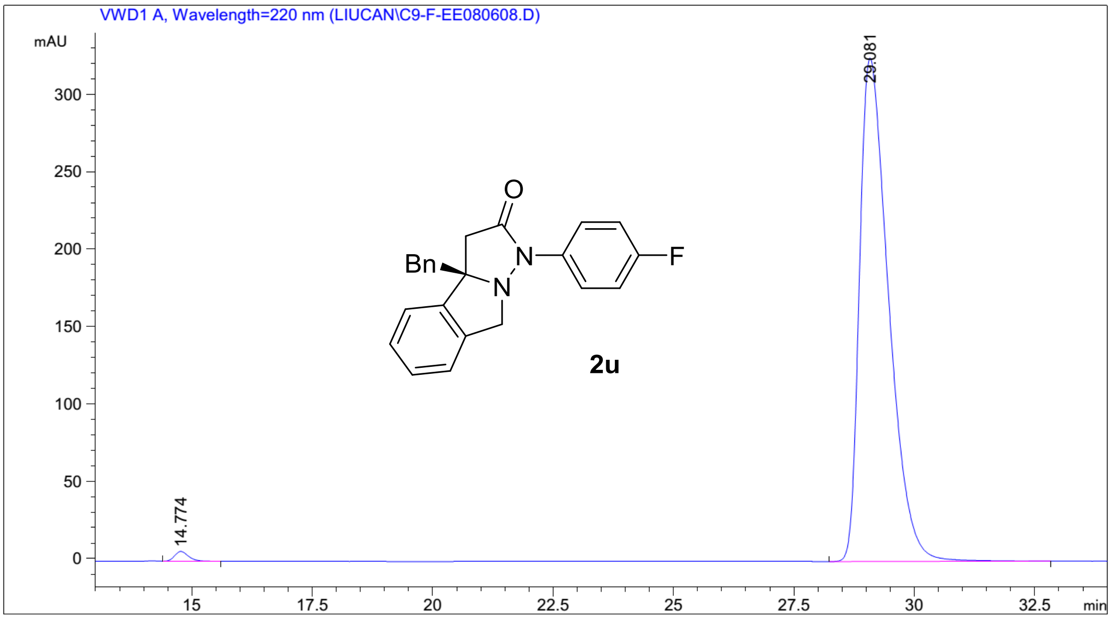

Peak #	RetTime [min]	Type	Width [min]	Area mAU *s	Height [mAU]	Area %
1	14.774	VB	0.3108	130.24582	6.38403	0.9477
2	29.081	BB	0.6456	1.36130e4	325.19955	99.0523

Figure S59. HPLC of (*S*)-2u, related to Table 2.

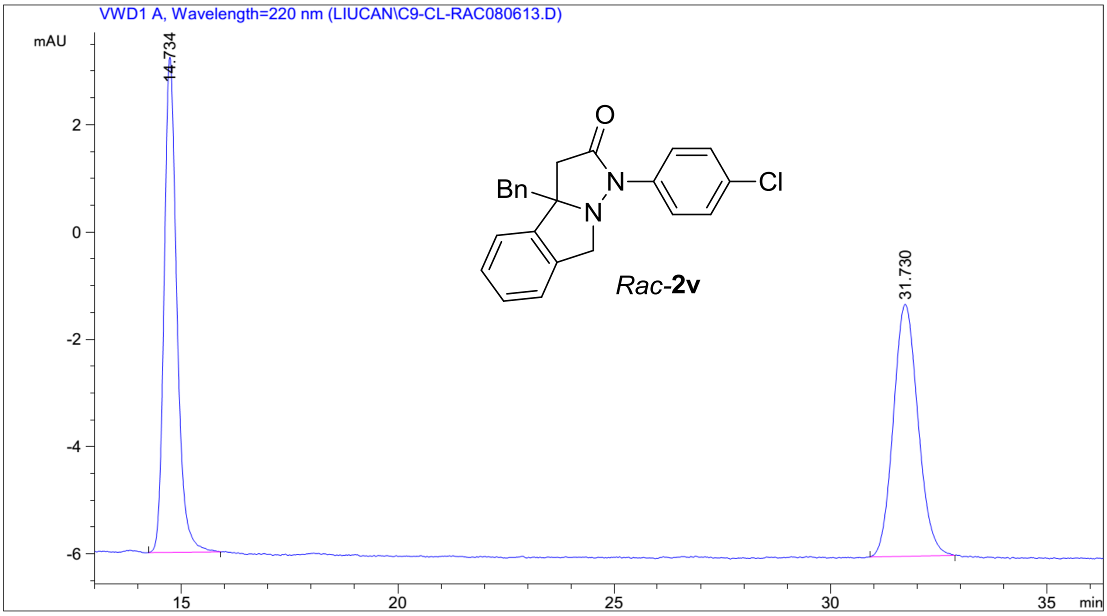

Peak #	RetTime [min]	Type	Width [min]	Area mAU *s	Height [mAU]	Area %
1	14.734	BB	0.3118	189.12756	9.23044	50.4611
2	31.730	BB	0.6038	185.67105	4.69788	49.5389

Figure S60. HPLC of *Rac*-2v, related to Table 2.

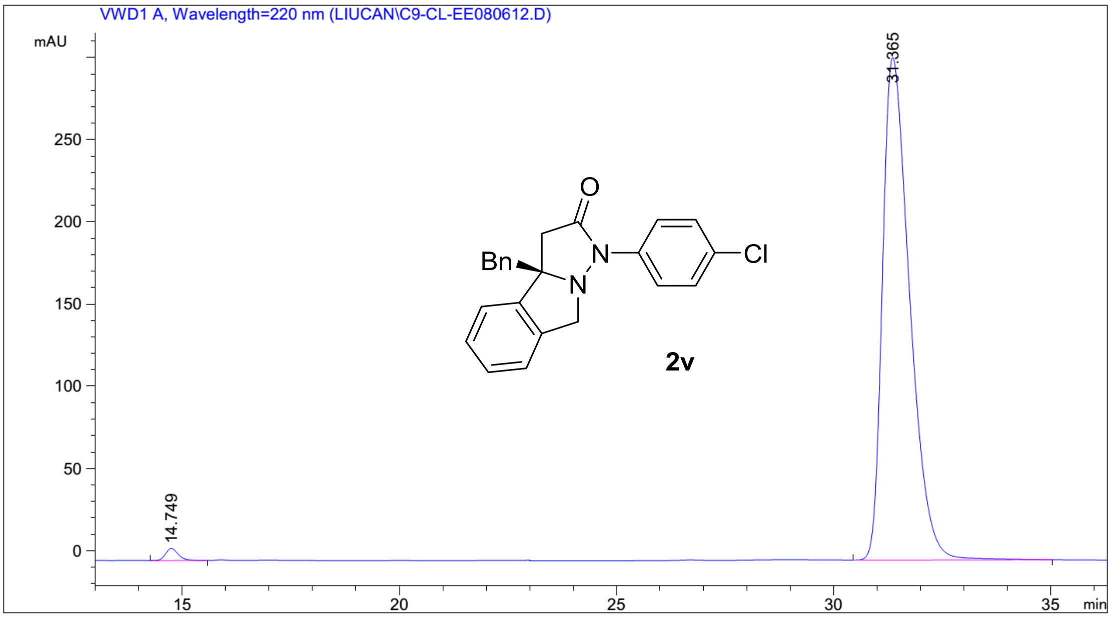

Peak #	RetTime [min]	Type	Width [min]	Area mAU *s	Height [mAU]	Area %
1	14.749	BB	0.3081	149.78223	7.38171	1.1152
2	31.365	BB	0.6672	1.32815e4	305.58240	98.8848

Figure S61. HPLC of (*S*)-2v, related to Table 2.

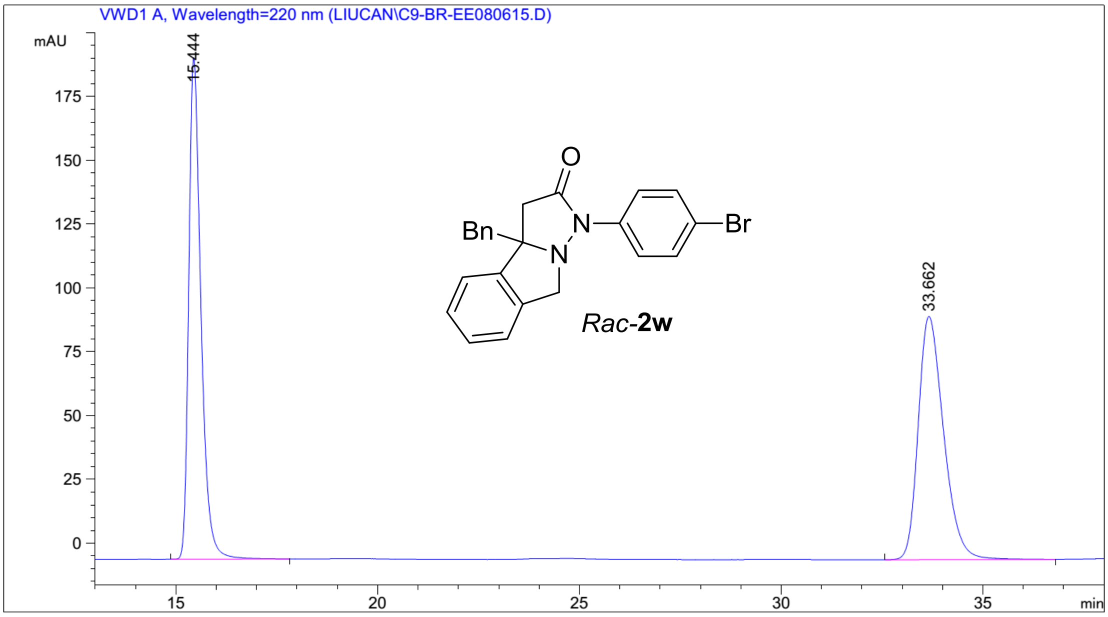

Peak #	RetTime [min]	Type	Width [min]	Area mAU *s	Height [mAU]	Area %
1	15.444	BB	0.3190	4121.93701	196.51964	49.7478
2	33.662	BB	0.6729	4163.72314	95.27980	50.2522

Figure S62. HPLC of *Rac*-2w, related to Table 2.

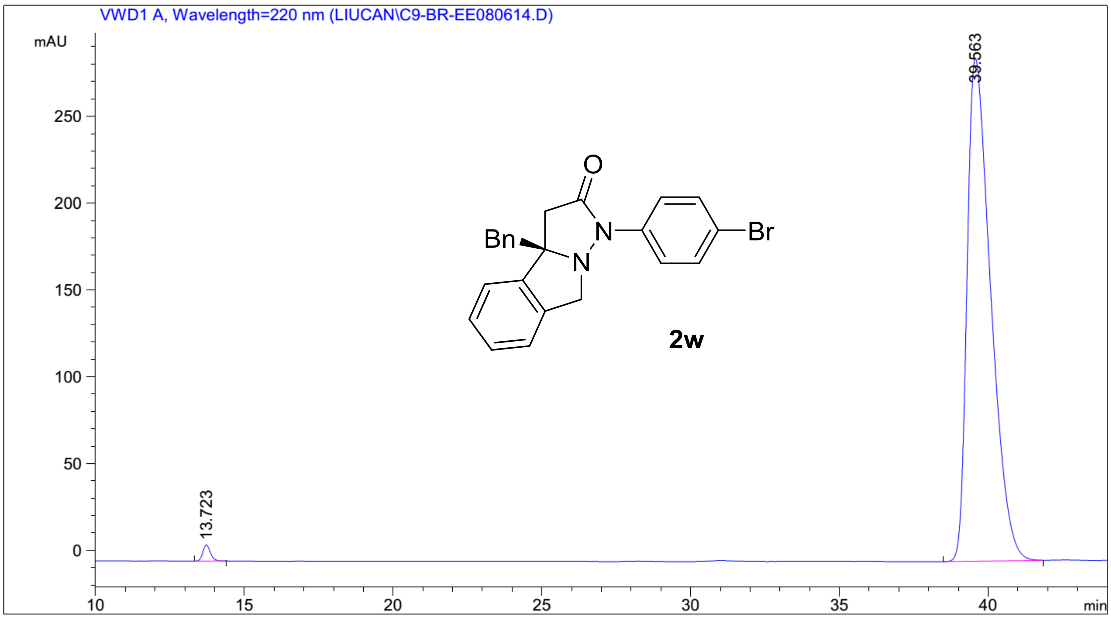

Peak #	RetTime [min]	Type	Width [min]	Area mAU *s	Height [mAU]	Area %
1	13.723	BB	0.2722	165.47081	9.34255	1.0268
2	39.563	BB	0.8434	1.59492e4	289.62326	98.9732

Figure S63. HPLC of (*S*)-2w, related to Table 2.

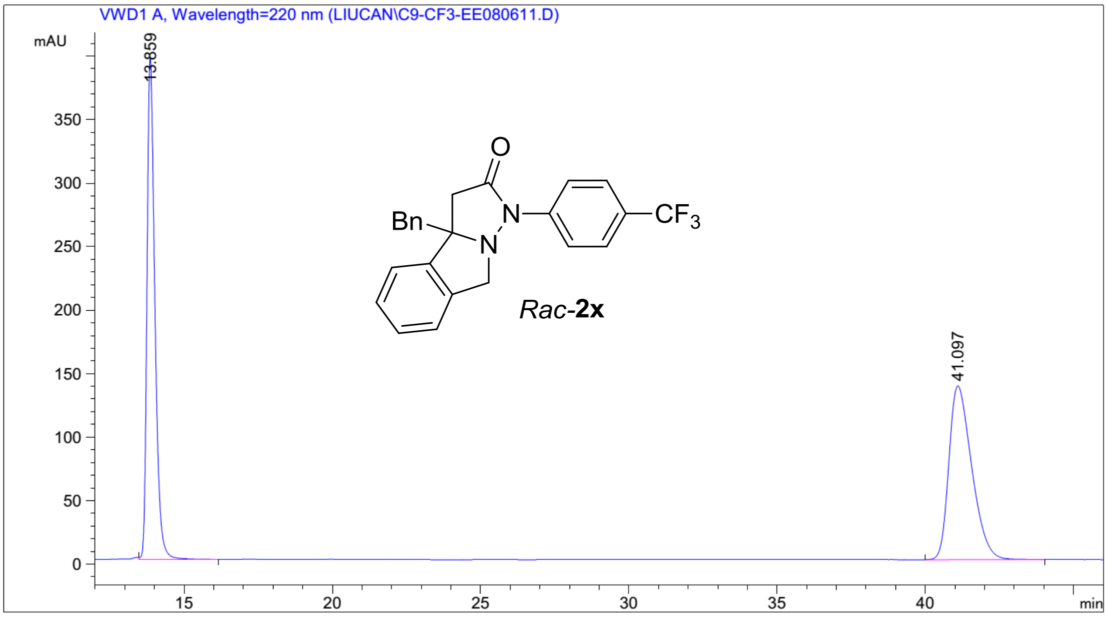

Peak #	RetTime [min]	Type	Width [min]	Area mAU *s	Height [mAU]	Area %
1	13.859	VB	0.2824	7295.52002	395.18219	49.9558
2	41.097	BB	0.8244	7308.41602	136.76088	50.0442

Figure S64. HPLC of *Rac*-2x, related to Table 2.

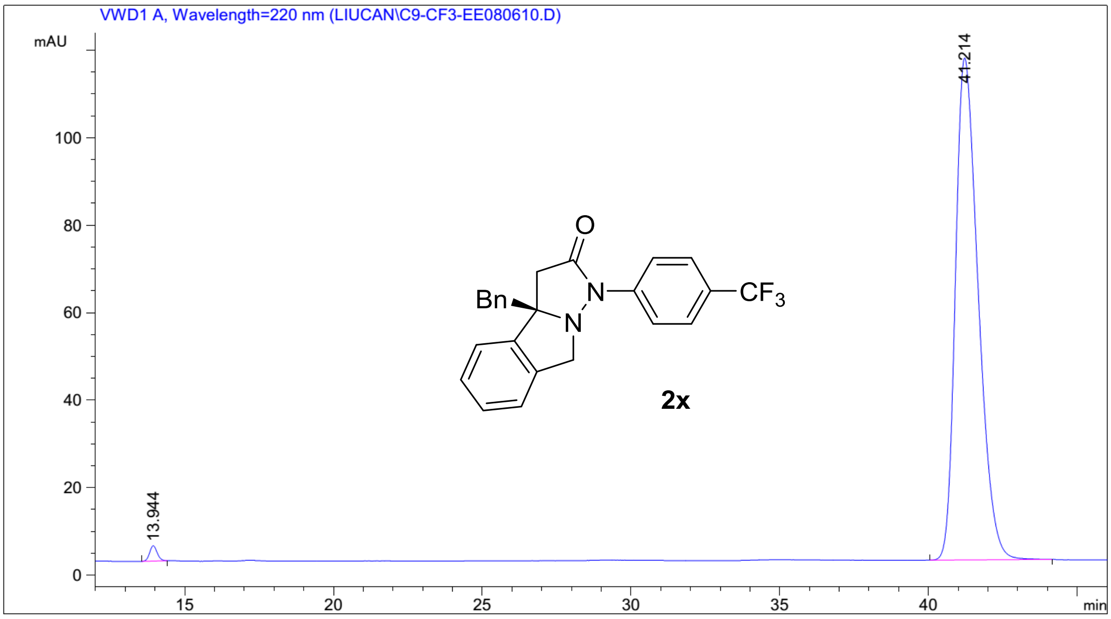

Peak #	RetTime [min]	Type	Width [min]	Area mAU *s	Height [mAU]	Area %
1	13.944	BB	0.2735	61.97538	3.50176	1.0055
2	41.214	BB	0.8196	6101.80078	114.79584	98.9945

Figure S65. HPLC of (*S*)-2x, related to Table 2.

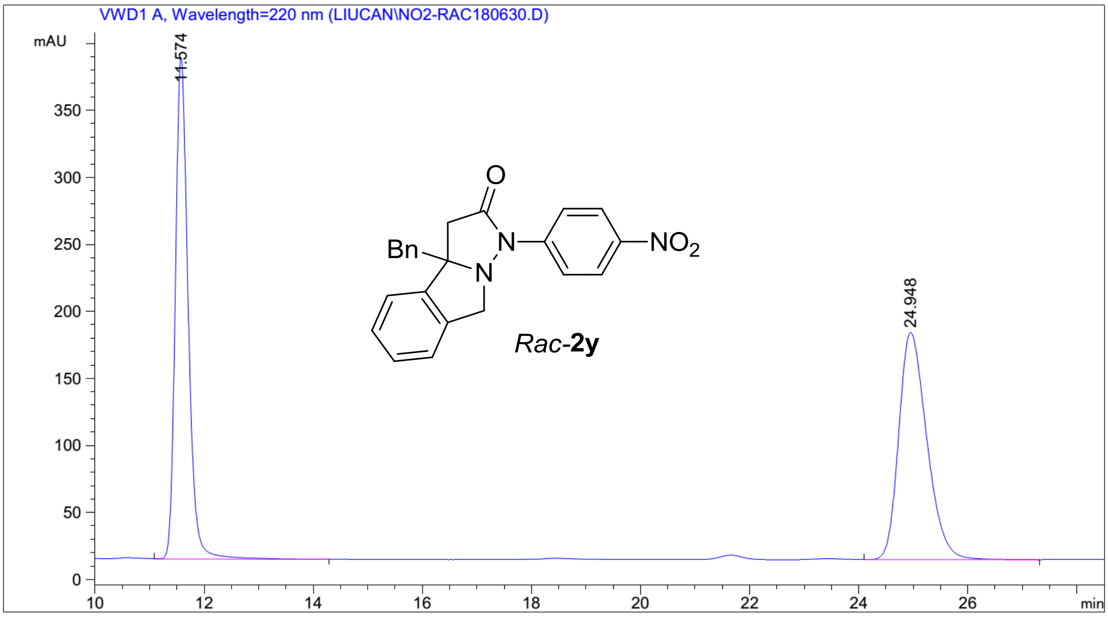

Peak #	RetTime [min]	Type	Width [min]	Area mAU *s	Height [mAU]	Area %
1	11.574	VB	0.2515	6100.75830	373.82654	50.1937
2	24.948	BB	0.5507	6053.67432	169.47473	49.8063

Figure S66. HPLC of *Rac*-2y, related to Table 2.

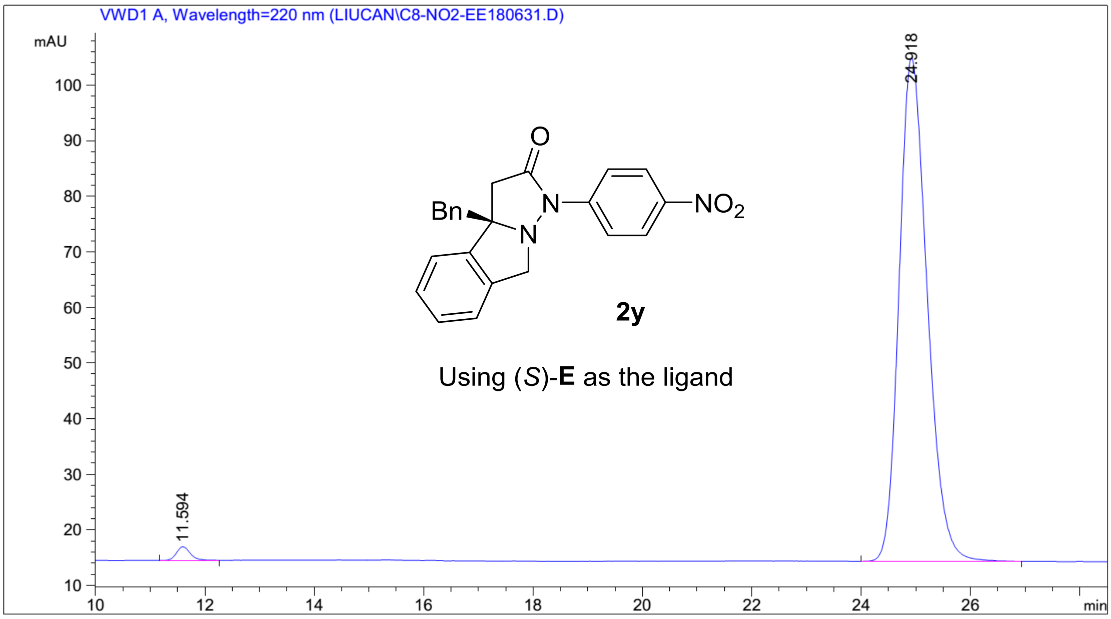

Peak #	RetTime [min]	Type	Width [min]	Area mAU *s	Height [mAU]	Area %
1	11.594	BB	0.2713	43.76435	2.46427	1.3357
2	24.918	BB	0.5505	3232.77905	90.53340	98.6643

Figure S67. HPLC of (*S*)-2y, related to Table 2.

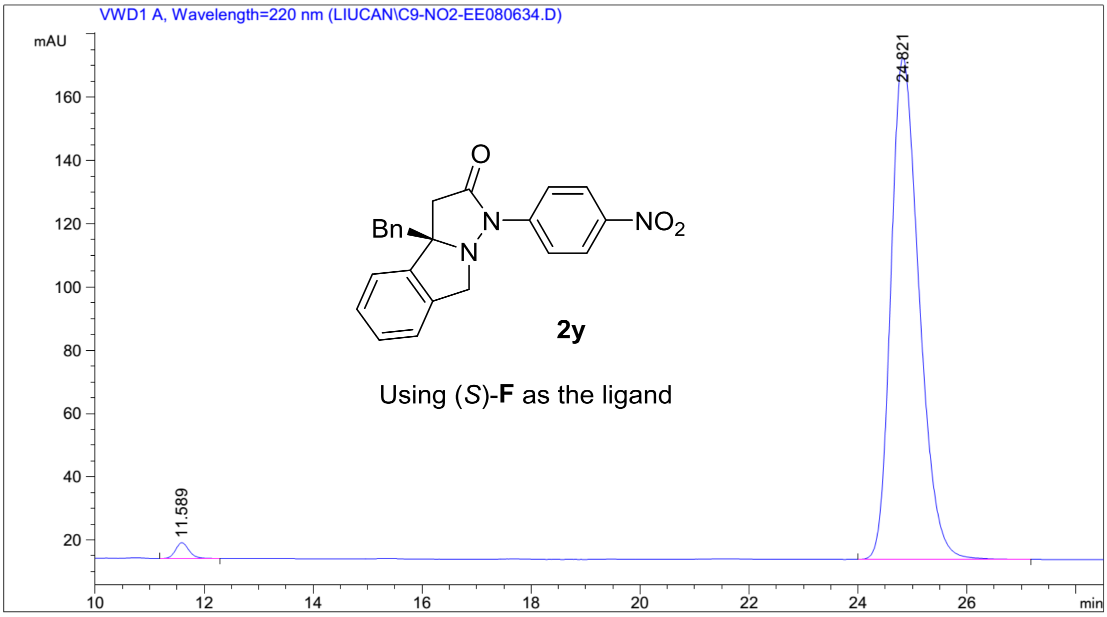

Peak #	RetTime [min]	Type	Width [min]	Area mAU *s	Height [mAU]	Area %
1	11.589	BB	0.2691	86.81152	5.01185	1.4995
2	24.821	BB	0.5564	5702.36768	158.56264	98.5005

Figure S68. HPLC of (*S*)-2y, related to Table 2.

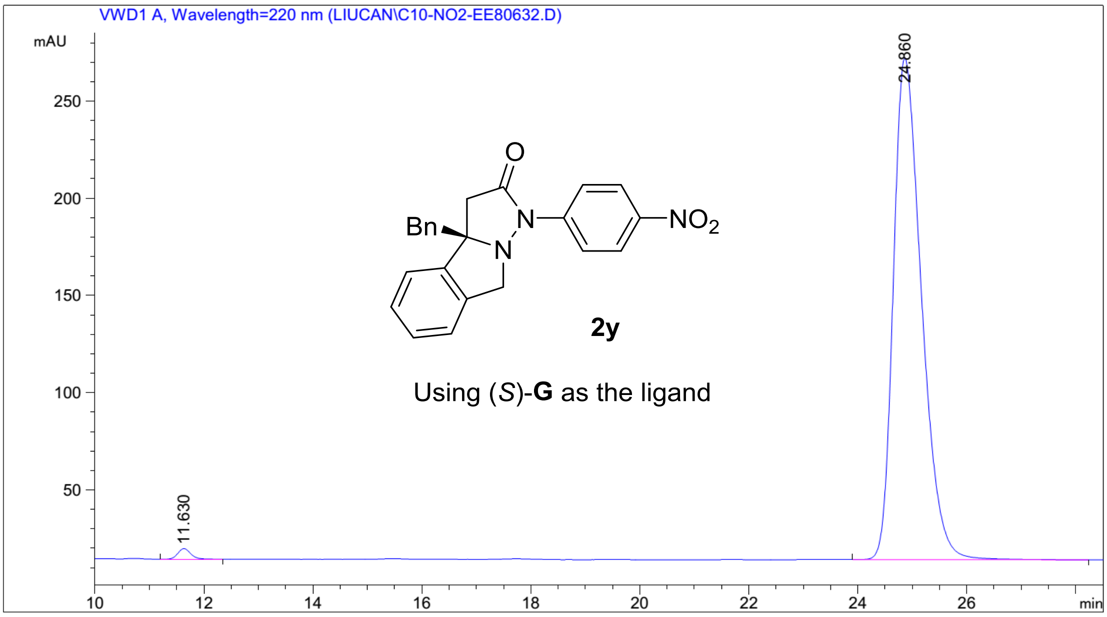

Peak #	RetTime [min]	Type	Width [min]	Area mAU *s	Height [mAU]	Area %
1	11.630	BB	0.2570	92.18953	5.49088	0.9880
2	24.860	BB	0.5487	9238.61914	258.08063	99.0120

Figure S69. HPLC of (*S*)-2y, related to Table 2.

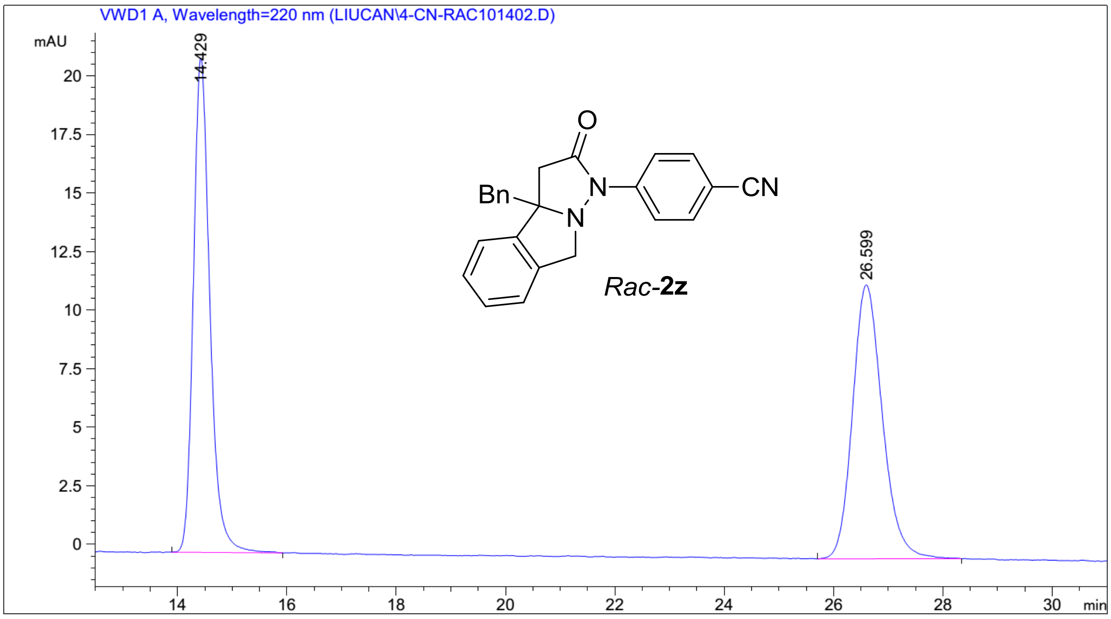

Peak #	RetTime [min]	Type	Width [min]	Area mAU *s	Height [mAU]	Area %
1	14.429	BB	0.3207	443.24789	21.10505	50.0349
2	26.599	BB	0.5786	442.62976	11.69038	49.9651

Figure S70. HPLC of *Rac*-2z, related to Table 2.

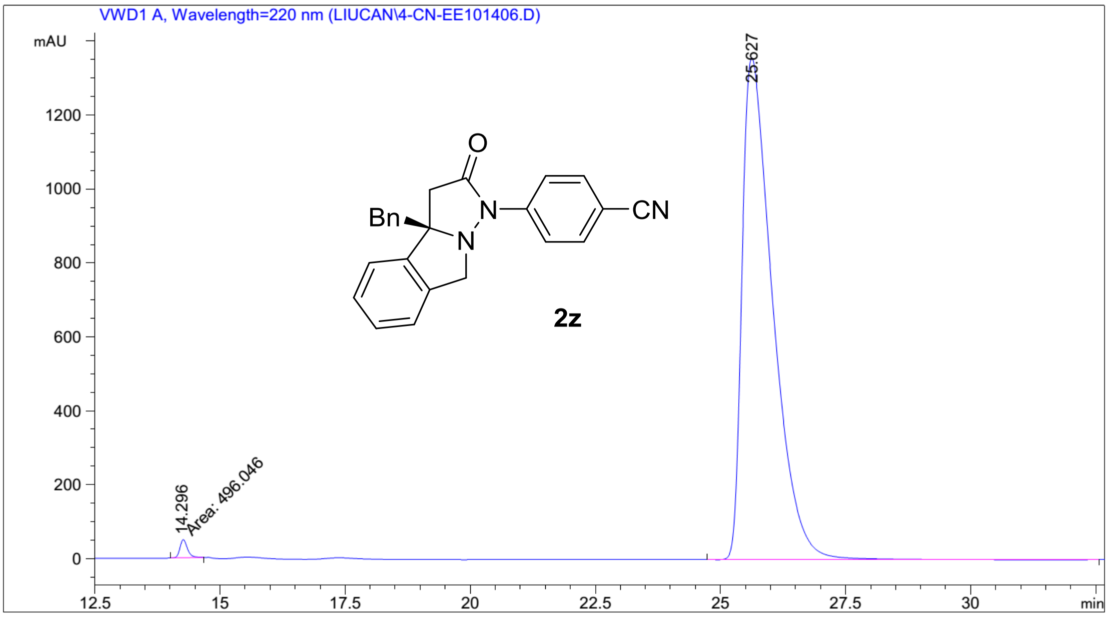

Peak #	RetTime [min]	Type	Width [min]	Area mAU *s	Height [mAU]	Area %
1	14.296	MM	0.1764	496.04568	46.85562	0.8510
2	25.627	BB	0.6423	5.77949e4	1357.44946	99.1490

Figure S71. HPLC of (*S*)-2z, related to Table 2.

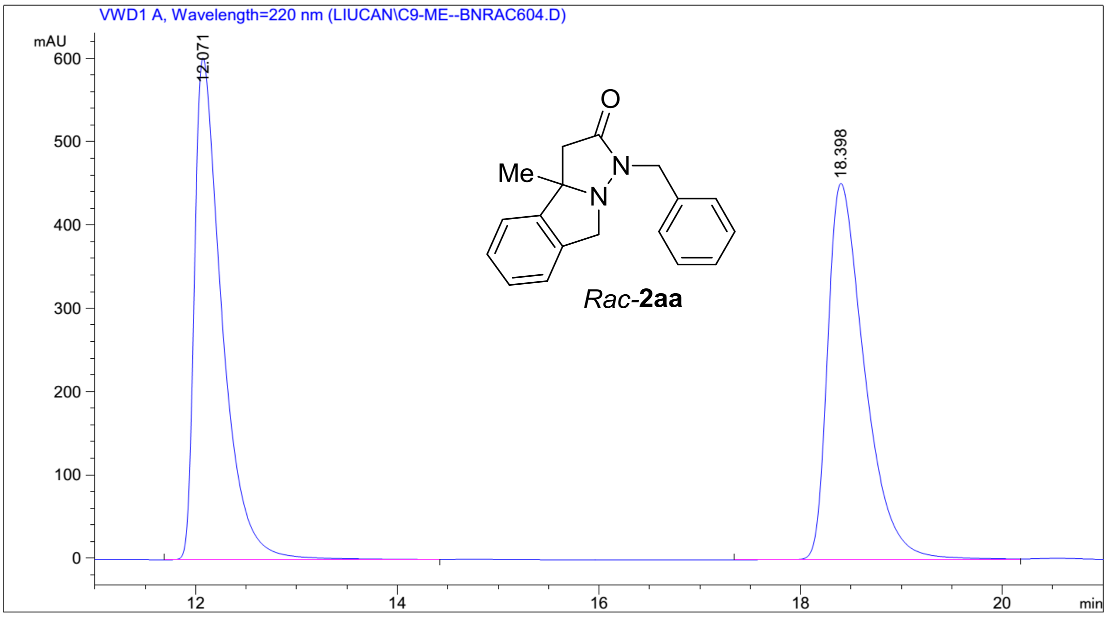

Peak #	RetTime [min]	Type	Width [min]	Area mAU *s	Height [mAU]	Area %
1	12.071	BV	0.2808	1.14189e4	602.43781	49.7547
2	18.398	BV	0.3866	1.15315e4	451.83405	50.2453

Figure S72. HPLC of *Rac*-2aa, related to Table 2.

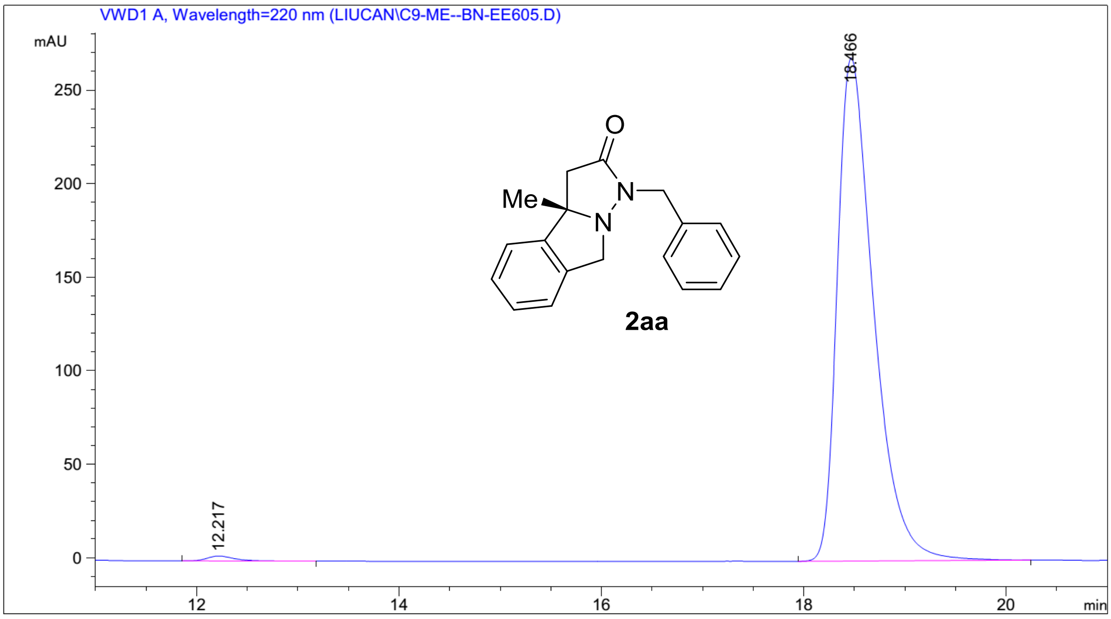

Peak #	RetTime [min]	Type	Width [min]	Area mAU *s	Height [mAU]	Area %
1	12.217	BB	0.2822	50.22261	2.66774	0.7430
2	18.466	BB	0.3784	6709.56104	268.92368	99.2570

Figure S73. HPLC of (*S*)-2aa, related to Table 2.

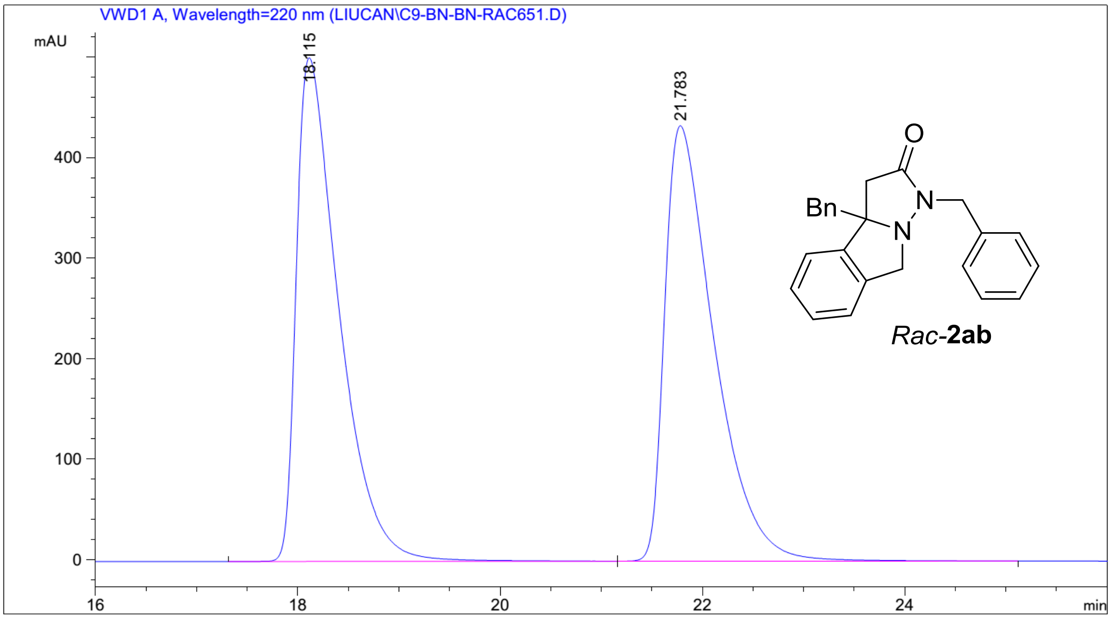

Peak #	RetTime [min]	Type	Width [min]	Area mAU *s	Height [mAU]	Area %
1	18.115	BB	0.4351	1.46261e4	501.21518	49.9847
2	21.783	BB	0.5075	1.46351e4	433.38348	50.0153

Figure S74. HPLC of *Rac*-2ab, related to Table 2.

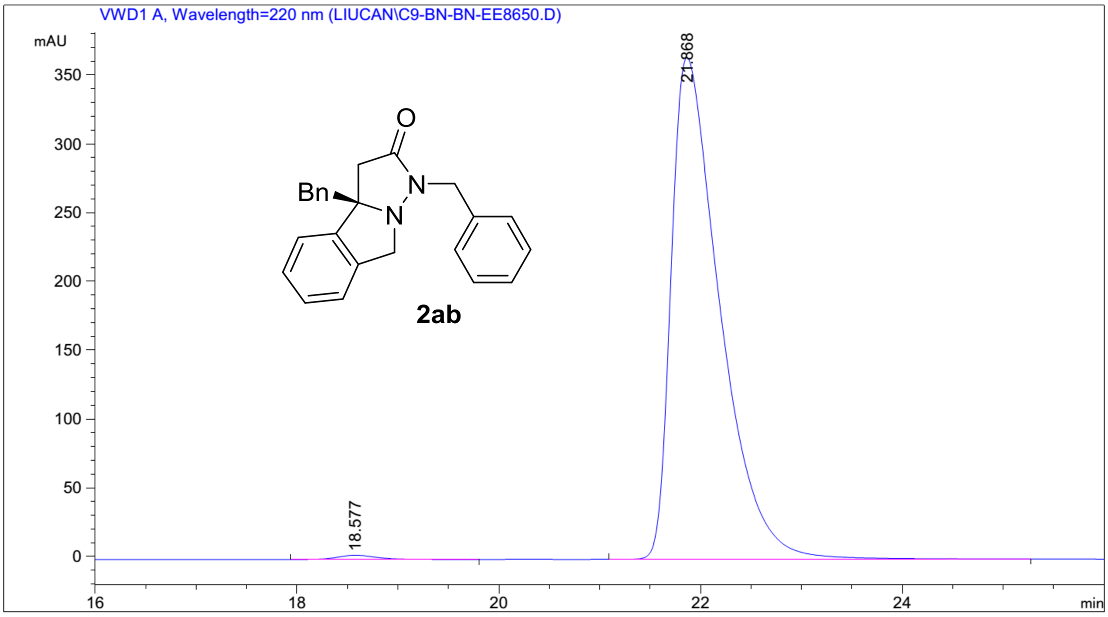

Peak #	RetTime [min]	Type	Width [min]	Area mAU *s	Height [mAU]	Area %
1	18.577	BB	0.4162	83.20202	3.04637	0.6770
2	21.868	BB	0.5042	1.22065e4	364.56906	99.3230

Figure S75. HPLC of (*S*)-2ab, related to Table 2.

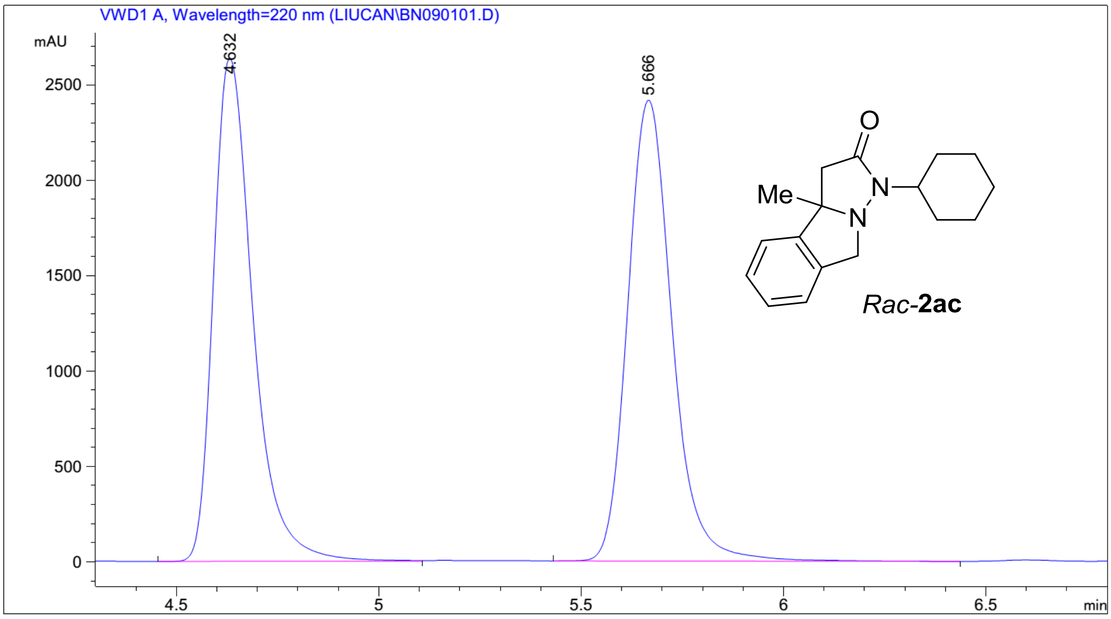

Peak #	RetTime [min]	Type	Width [min]	Area mAU *s	Height [mAU]	Area %
1	4.632	BV	0.1050	1.78426e4	2641.08325	49.1888
2	5.666	BV	0.1181	1.84312e4	2416.49292	50.8112

Figure S76. HPLC of *Rac*-2ac, related to Table 2.

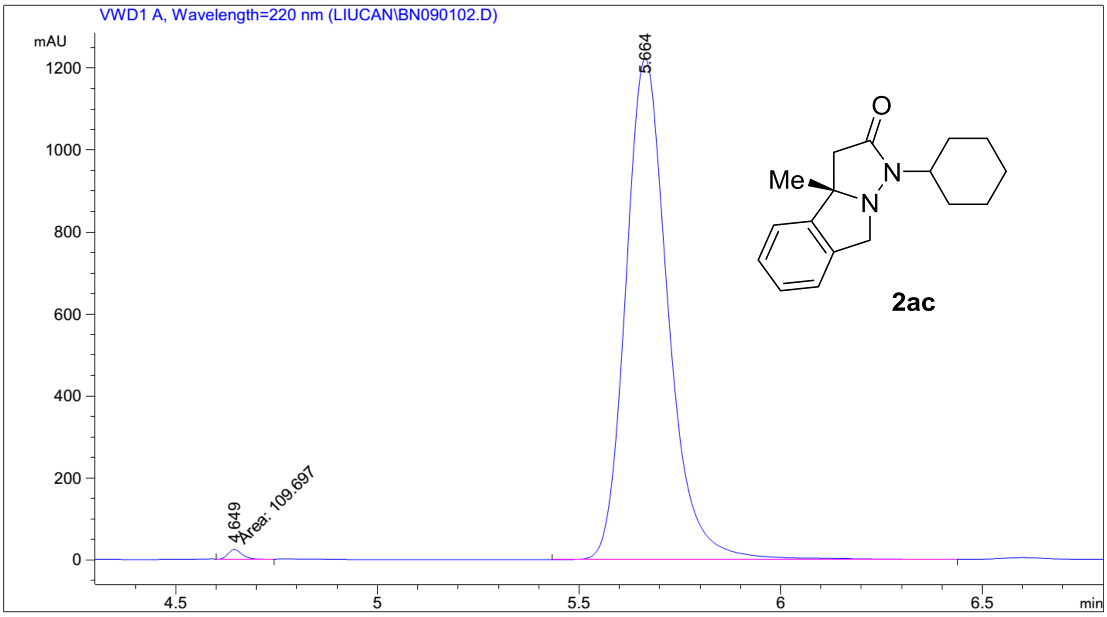

Peak #	RetTime [min]	Type	Width [min]	Area mAU *s	Height [mAU]	Area %
1	4.649	MM	0.0570	109.69749	32.06429	1.2113
2	5.664	BV	0.1129	8946.20898	1224.31409	98.7887

Figure S77. HPLC of (*S*)-2ac, related to Table 2.

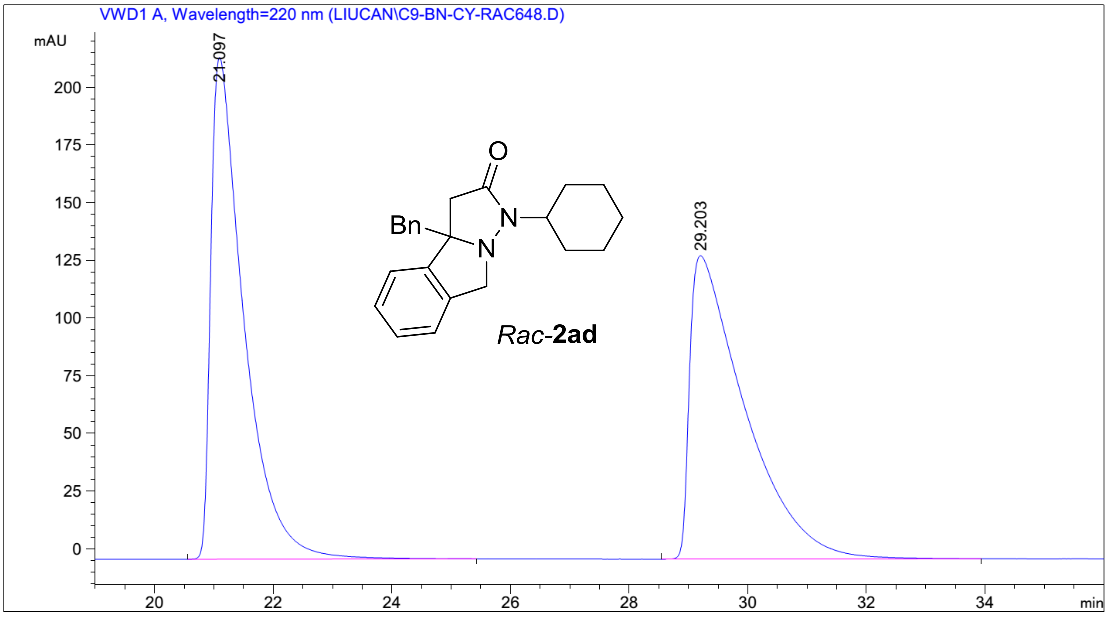

Peak #	RetTime [min]	Type	Width [min]	Area mAU *s	Height [mAU]	Area %
1	21.097	BB	0.5671	8425.39941	217.42725	49.7880
2	29.203	BB	0.9086	8497.14355	131.43498	50.2120

Figure S78. HPLC of *Rac*-2ad, related to Table 2.

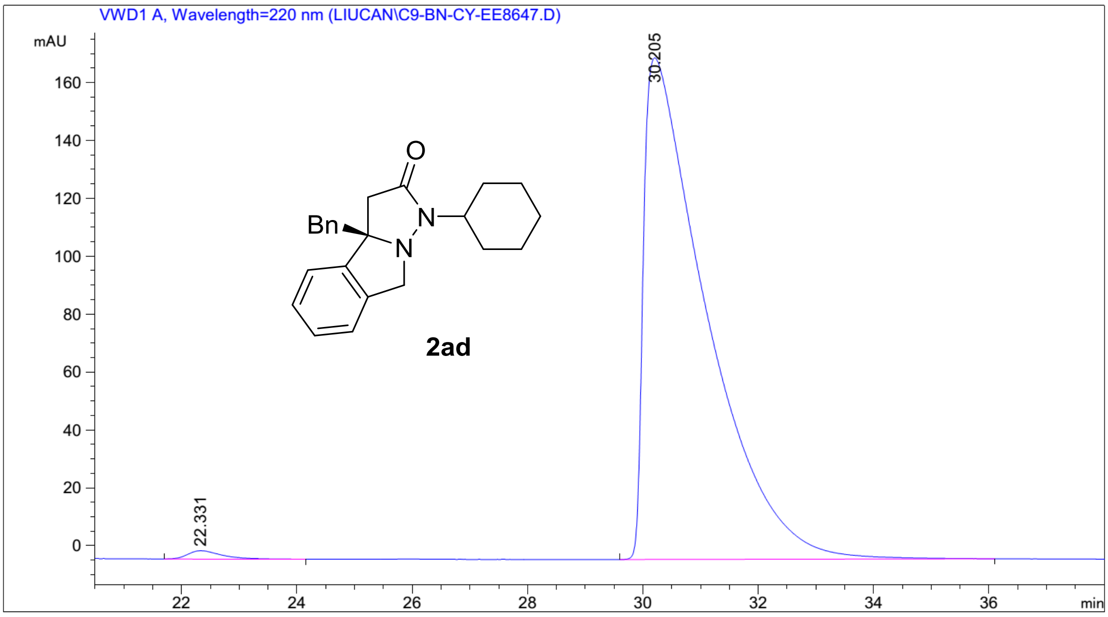

Peak #	RetTime [min]	Type	Width [min]	Area mAU *s	Height [mAU]	Area %
1	22.331	BB	0.5742	117.32352	2.86322	0.8907
2	30.205	BB	1.0625	1.30553e4	173.27299	99.1093

Figure S79. HPLC of (*S*)-2ad, related to Table 2.

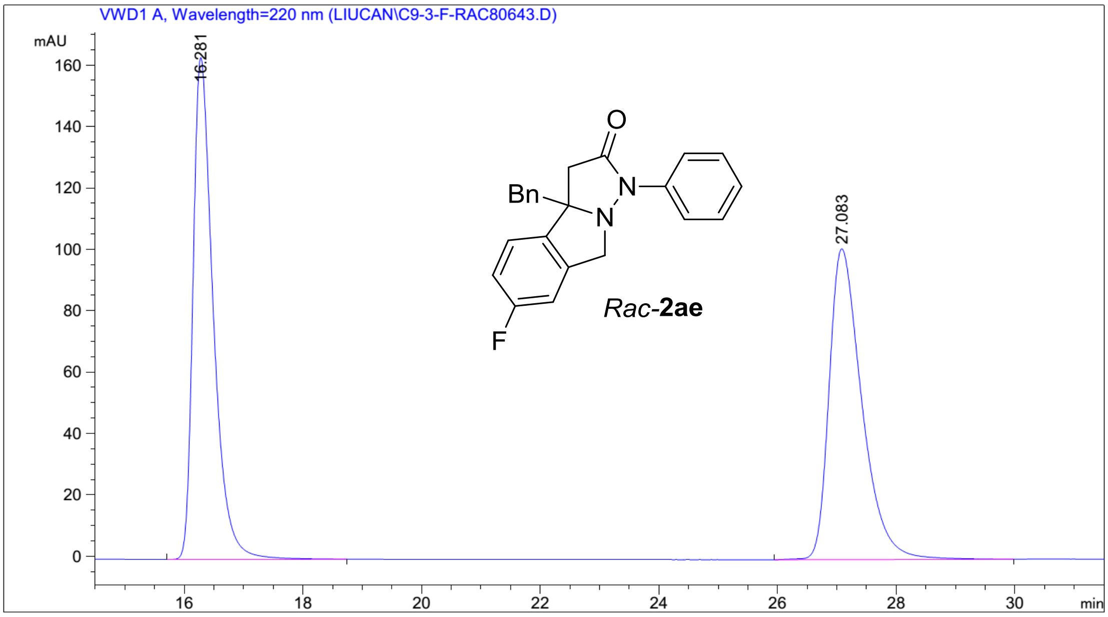

Peak #	RetTime [min]	Type	Width [min]	Area mAU *s	Height [mAU]	Area %
1	16.281	BB	0.3567	3856.23291	163.47878	49.8987
2	27.083	BB	0.5830	3871.89014	101.25486	50.1013

Figure S80. HPLC of *Rac*-2ae, related to Table 2.

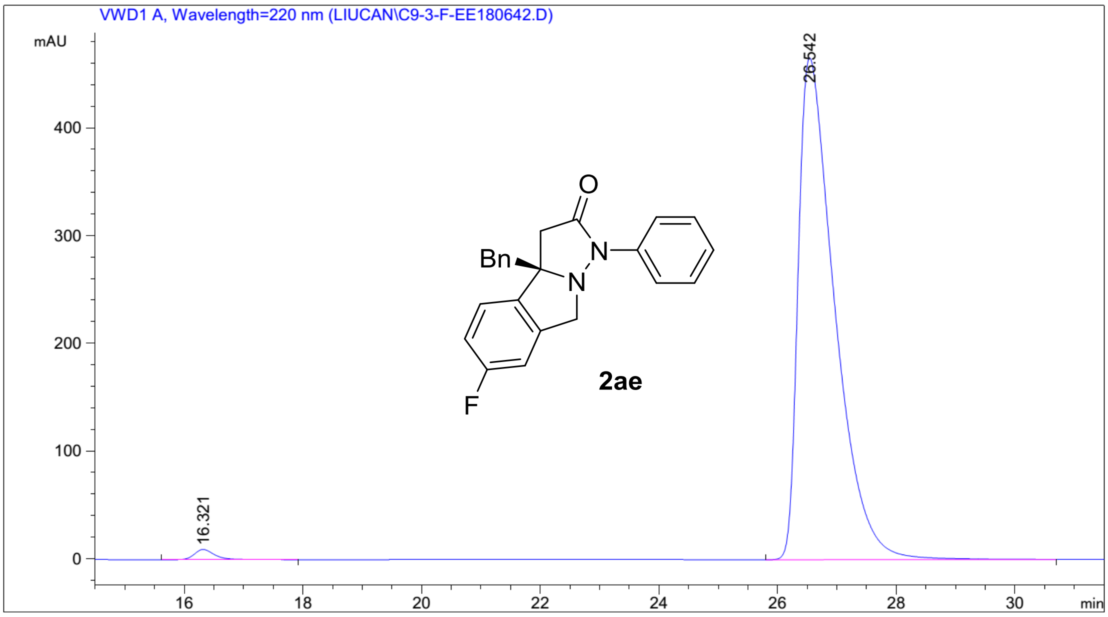

Peak #	RetTime [min]	Type	Width [min]	Area mAU *s	Height [mAU]	Area %
1	16.321	BB	0.3653	227.38745	9.44409	1.1354
2	26.542	BB	0.6357	1.98004e4	465.82135	98.8646

Figure S81. HPLC of (*S*)-2ae, related to Table 2.

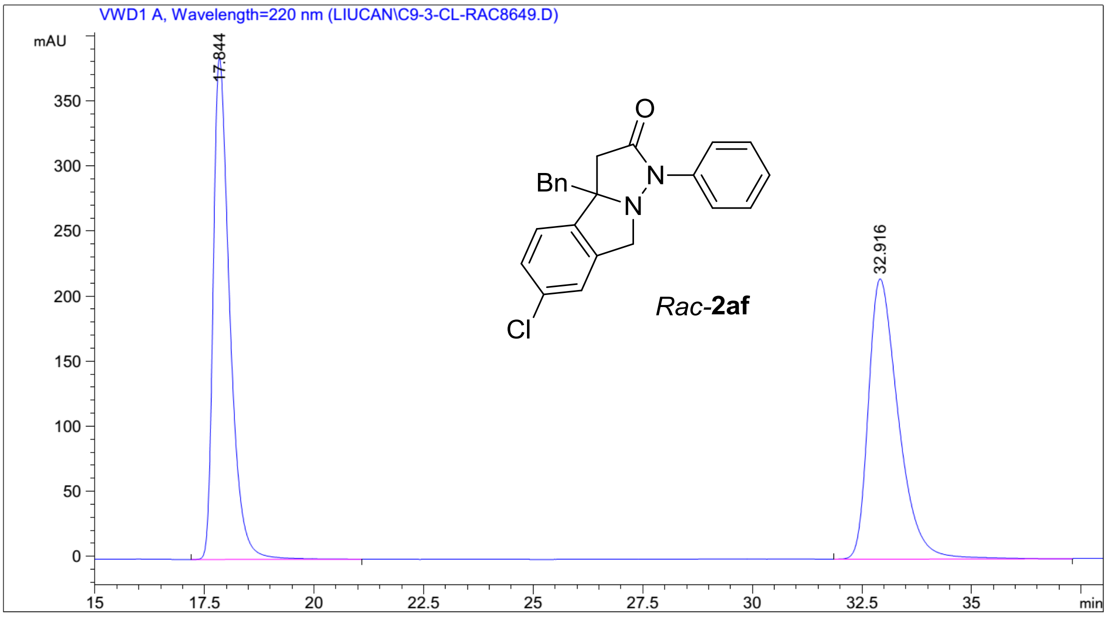

Peak #	RetTime [min]	Type	Width [min]	Area mAU *s	Height [mAU]	Area %
1	17.844	BB	0.3968	1.00765e4	385.40063	49.5704
2	32.916	BB	0.7245	1.02511e4	215.25081	50.4296

Figure S82. HPLC of *Rac*-2af, related to Table 2.

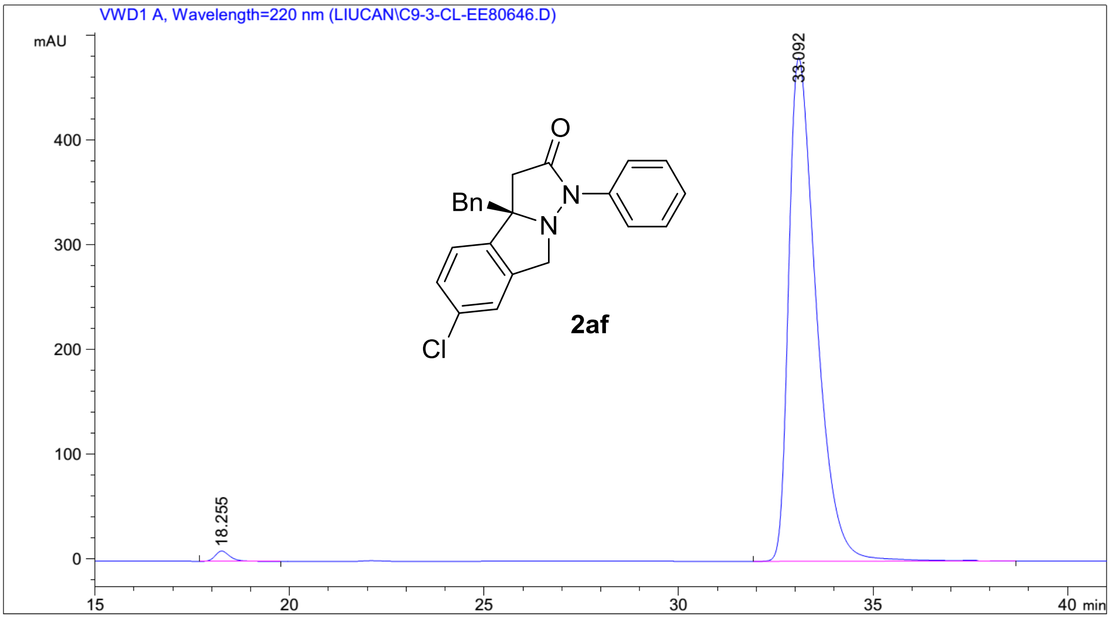

Peak #	RetTime [min]	Type	Width [min]	Area mAU *s	Height [mAU]	Area %
1	18.255	BB	0.4037	260.69507	9.84072	1.0888
2	33.092	BB	0.7447	2.36834e4	481.06613	98.9112

Figure S83. HPLC of (*S*)-2af, related to Table 2.

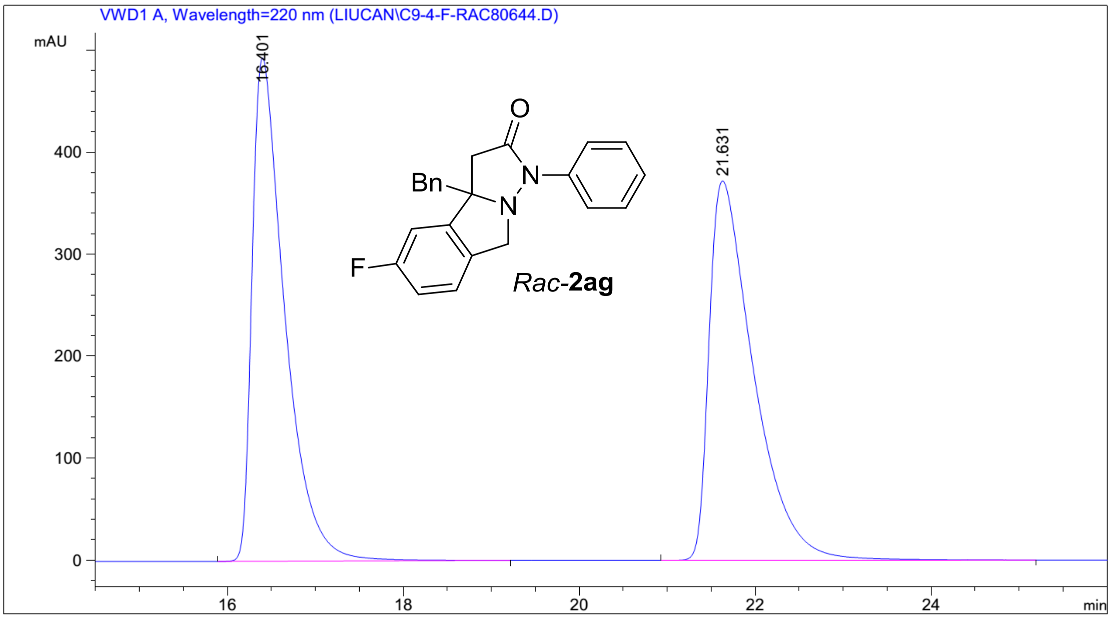

Peak #	RetTime [min]	Type	Width [min]	Area mAU *s	Height [mAU]	Area %
1	16.401	BB	0.3863	1.27614e4	493.21194	49.4415
2	21.631	BB	0.5200	1.30497e4	371.90881	50.5585

Figure S84. HPLC of *Rac*-2ag, related to Table 2.

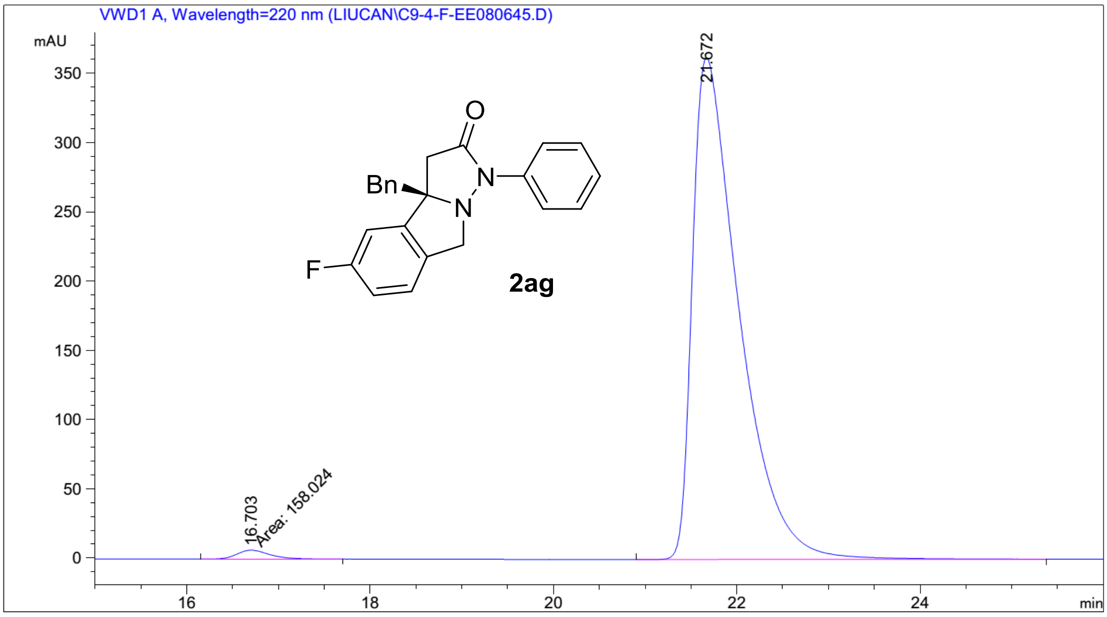

Peak #	RetTime [min]	Type	Width [min]	Area mAU *s	Height [mAU]	Area %
1	16.703	MM	0.4121	158.02449	6.39035	1.2218
2	21.672	BB	0.5221	1.27757e4	362.30176	98.7782

Figure S85. HPLC of (*S*)-2ag, related to Table 2.

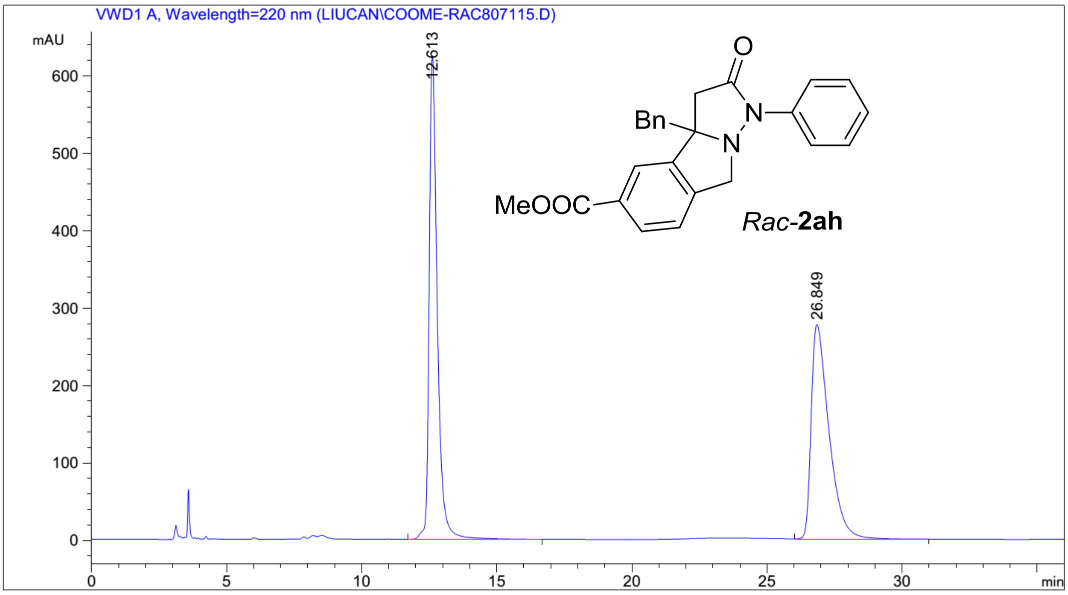

Peak #	RetTime [min]	Type	Width [min]	Area mAU *s	Height [mAU]	Area %
1	12.613	BB	0.3145	1.30880e4	624.26660	50.6649
2	26.849	VB	0.6875	1.27445e4	277.36618	49.3351

Figure S86. HPLC of *Rac*-2ah, related to Table 2.

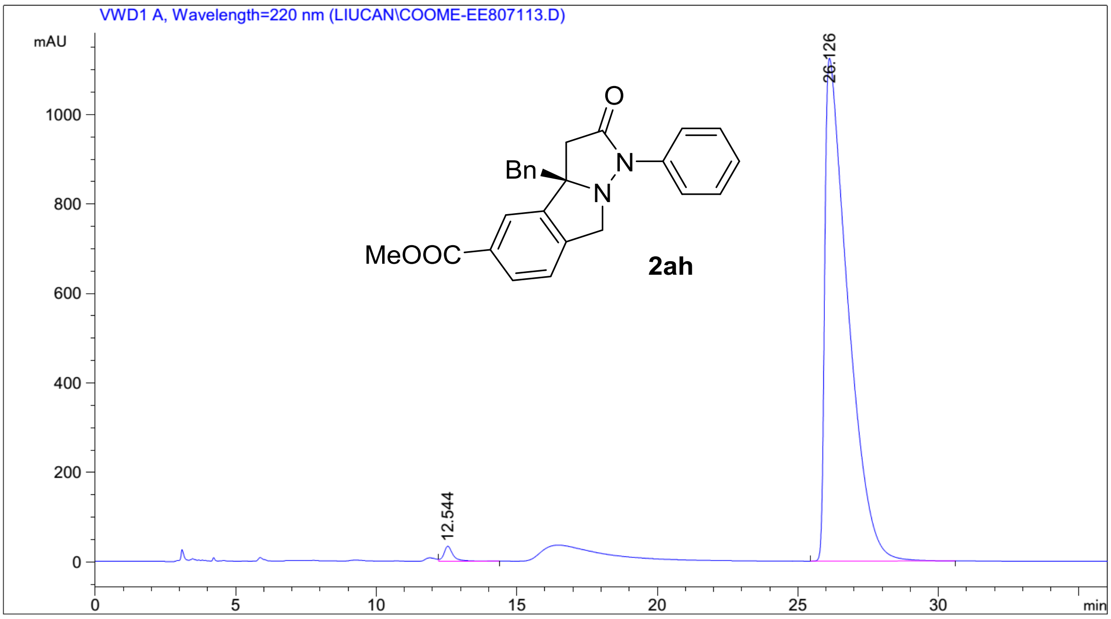

Peak #	RetTime [min]	Type	Width [min]	Area mAU *s	Height [mAU]	Area %
1	12.544	VB	0.3350	761.85925	33.53305	1.1770
2	26.126	BB	0.8167	6.39651e4	1124.12646	98.8230

Figure S87. HPLC of (*S*)-2ah, related to Table 2.

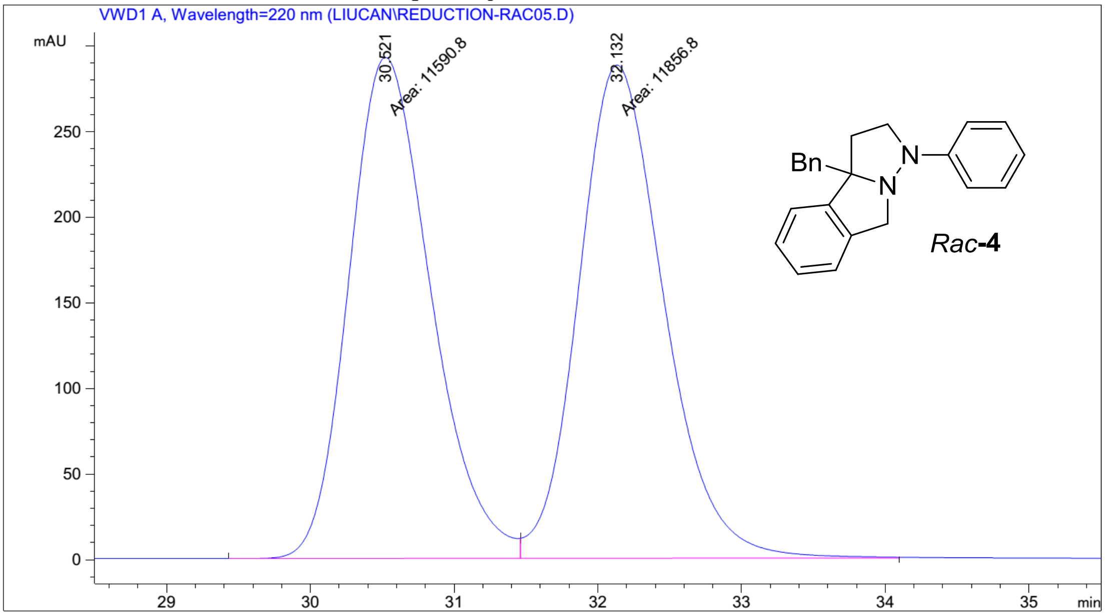

Peak #	RetTime [min]	Type	Width [min]	Area mAU *s	Height [mAU]	Area %
1	30.521	MF	0.6602	1.15908e4	292.61334	49.4326
2	32.132	FM	0.6862	1.18568e4	287.97058	50.5674

Figure S88. HPLC of *Rac*-4, related to Figure 4.

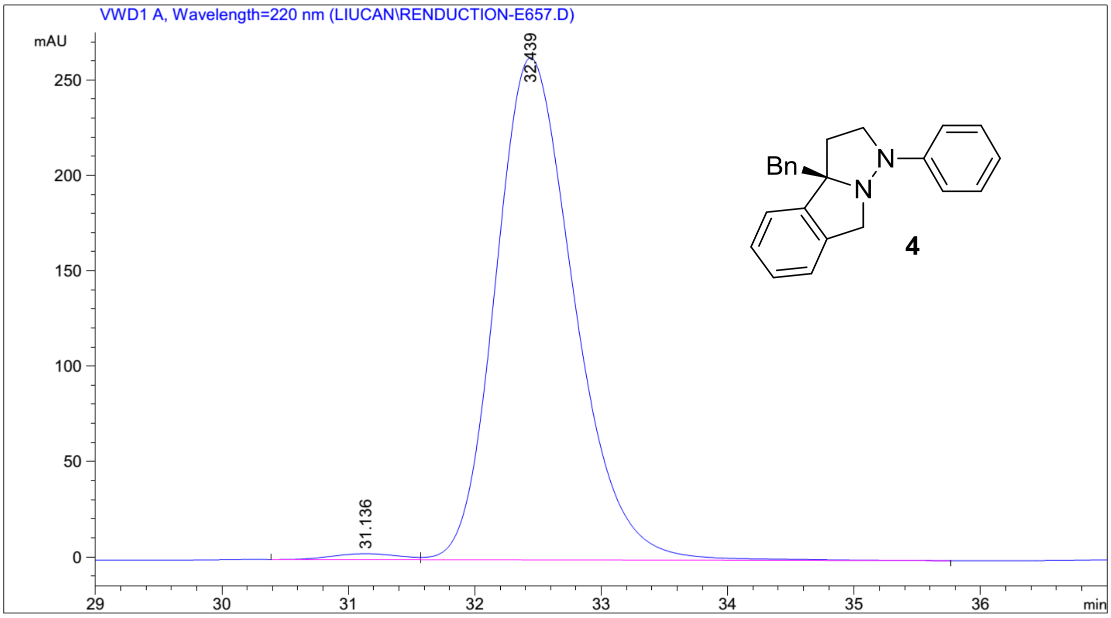

Peak #	RetTime [min]	Type	Width [min]	Area mAU *s	Height [mAU]	Area %
1	31.136	BV	0.4945	115.28838	3.12490	1.0054
2	32.439	VB	0.6620	1.13517e4	263.14926	98.9946

Figure S89. HPLC of (*S*)-4, related to Figure 4.

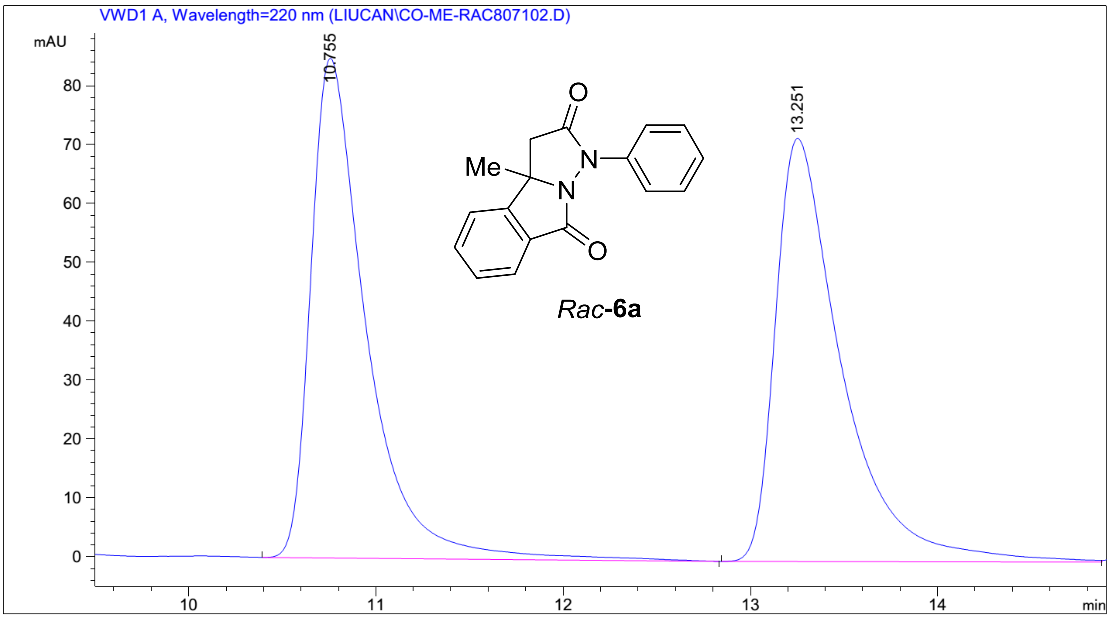

Peak #	RetTime [min]	Type	Width [min]	Area mAU *s	Height [mAU]	Area %
1	10.755	BB	0.3062	1761.38025	84.87390	49.7892
2	13.251	BV	0.3626	1776.29614	71.86400	50.2108

Figure S90. HPLC of *Rac*-6a, related to Figure 6.

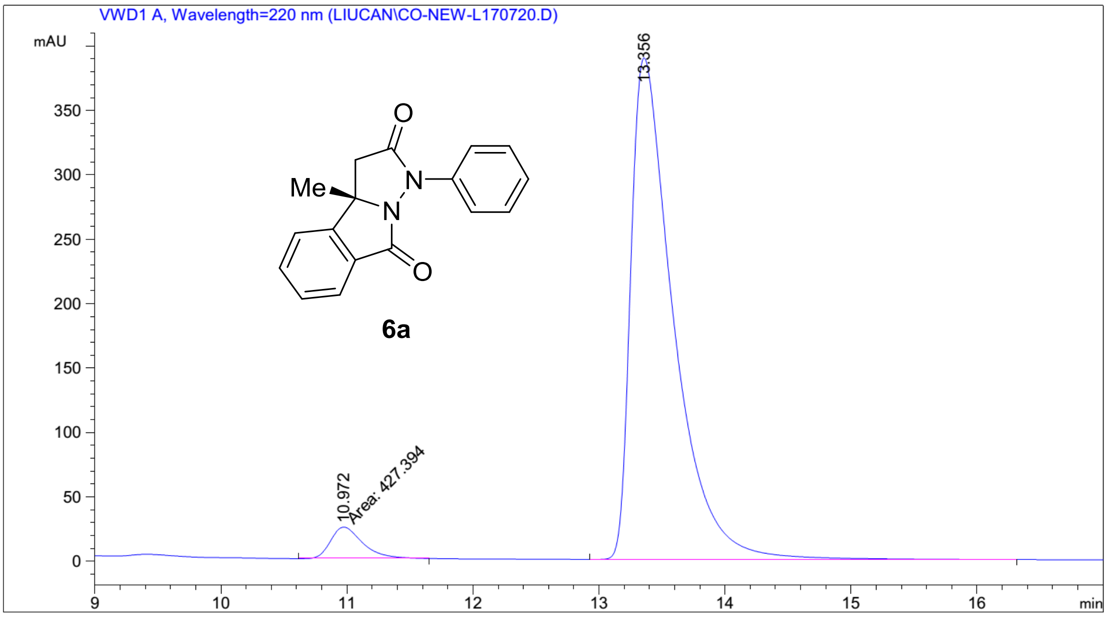

Peak #	RetTime [min]	Type	Width [min]	Area mAU *s	Height [mAU]	Area %
1	10.972	MM	0.2926	427.39365	24.34558	4.4640
2	13.356	BB	0.3483	9146.90527	389.43353	95.5360

Figure S91. HPLC of (*S*)-6a, related to Figure 6.

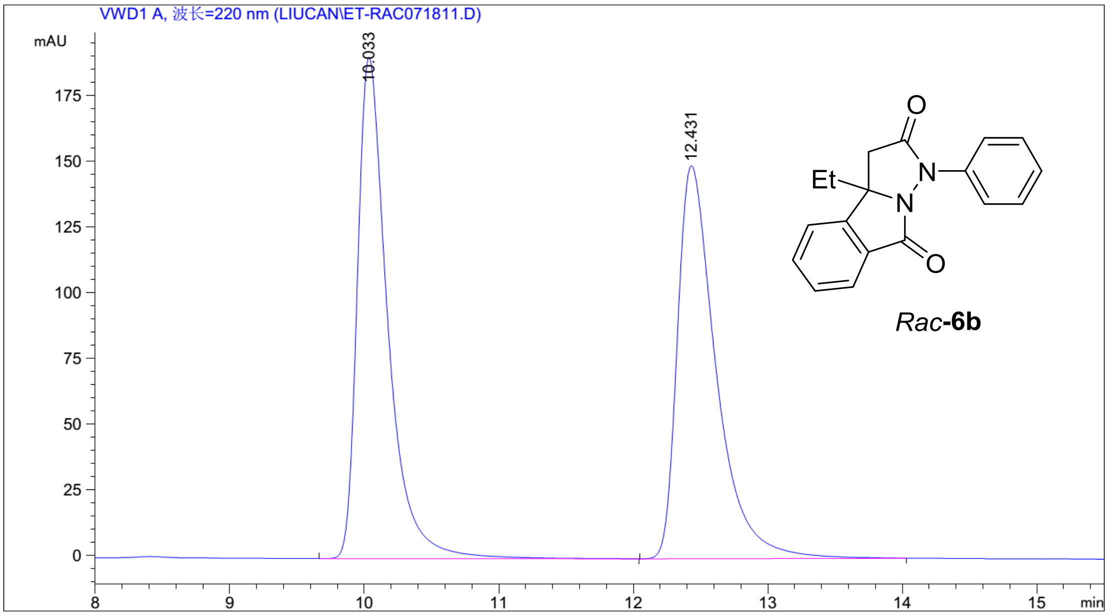

Peak #	RetTime [min]	Type	Width [min]	Area mAU *s	Height [mAU]	Area %
1	10.033	BB	0.2354	2993.44751	190.81116	50.0245
2	12.431	BB	0.3018	2990.51367	149.51964	49.9755

Figure S92. HPLC of *Rac*-6b, related to Figure 6.

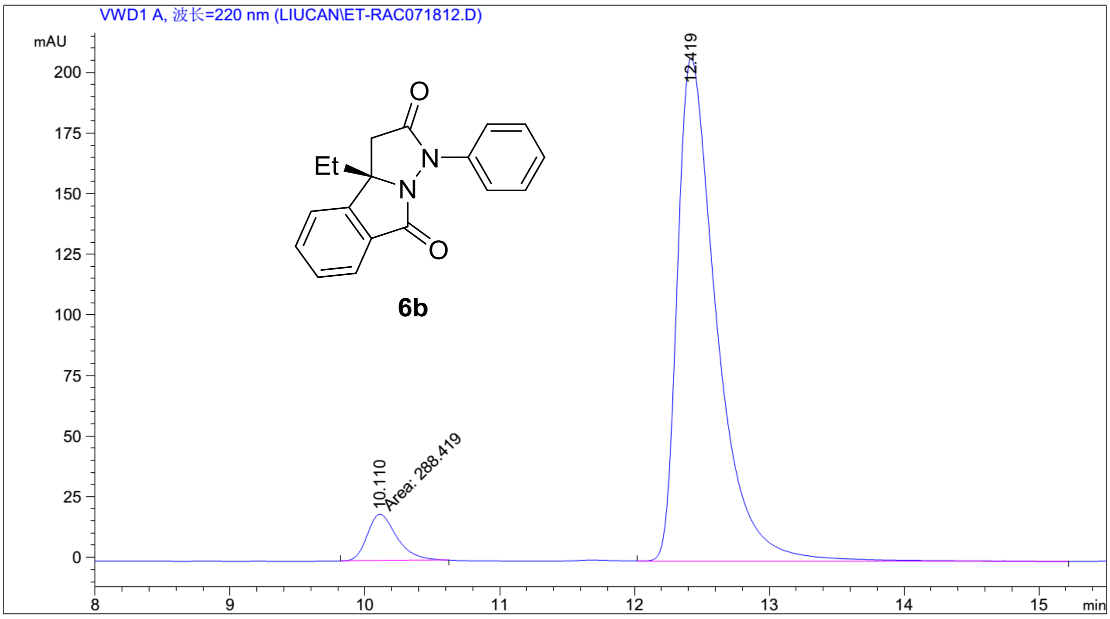

Peak #	RetTime [min]	Type	Width [min]	Area mAU *s	Height [mAU]	Area %
1	10.110	MM	0.2519	288.41867	19.08532	6.4235
2	12.419	VB	0.3032	4201.60693	207.49075	93.5765

Figure S93. HPLC of (*S*)-6b, related to Figure 6.

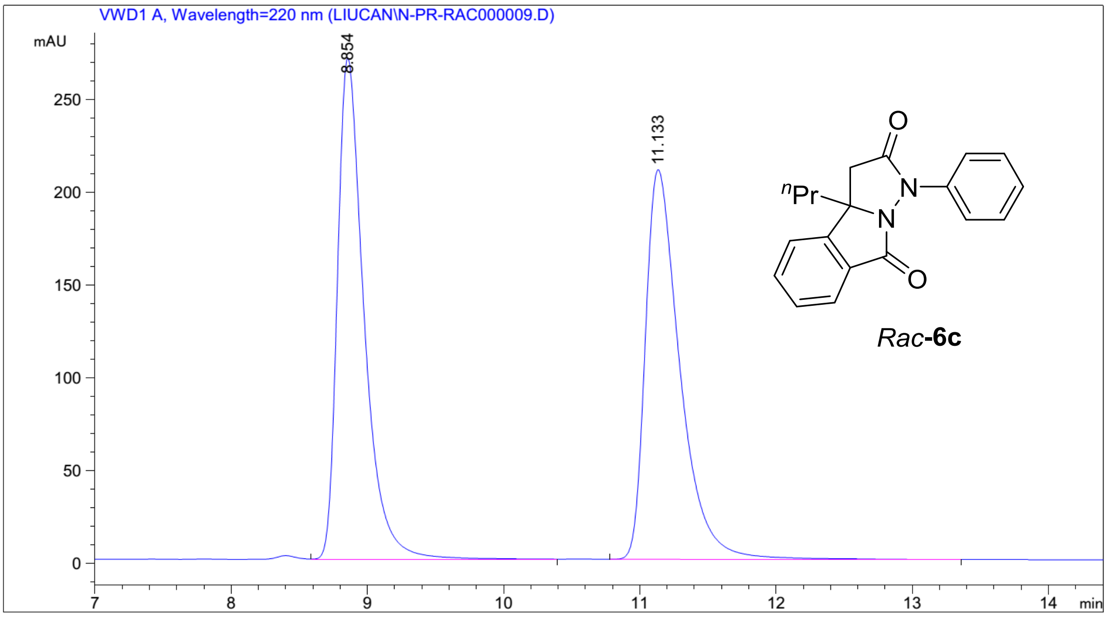

Peak #	RetTime [min]	Type	Width [min]	Area mAU *s	Height [mAU]	Area %
1	8.854	VB	0.2049	3669.81494	270.41547	49.7017
2	11.133	BB	0.2674	3713.86768	210.10957	50.2983

Figure S94. HPLC of *Rac*-6c, related to Figure 6.

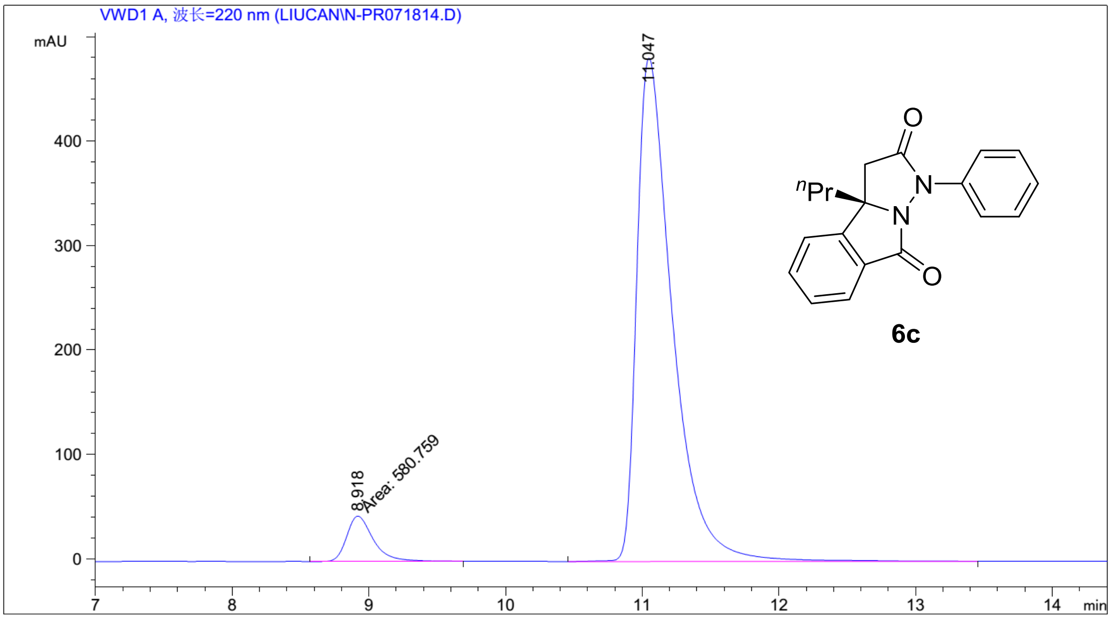

Peak #	RetTime [min]	Type	Width [min]	Area mAU *s	Height [mAU]	Area %
1	8.918	MM	0.2234	580.75854	43.33228	6.2395
2	11.047	BB	0.2724	8726.95410	481.95743	93.7605

Figure S95. HPLC of (*S*)-6c, related to Figure 6.

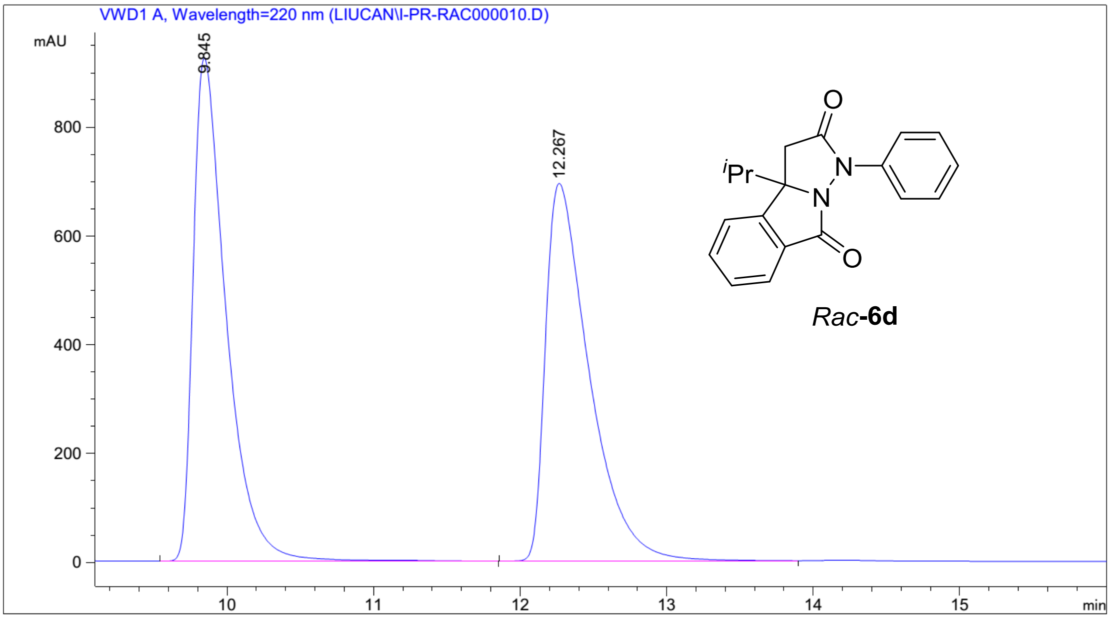

Peak #	RetTime [min]	Type	Width [min]	Area mAU *s	Height [mAU]	Area %
1	9.845	BB	0.2351	1.44948e4	925.55493	50.0591
2	12.267	BV	0.3114	1.44606e4	694.28271	49.9409

Figure S96. HPLC of *Rac*-6d, related to Figure 6.

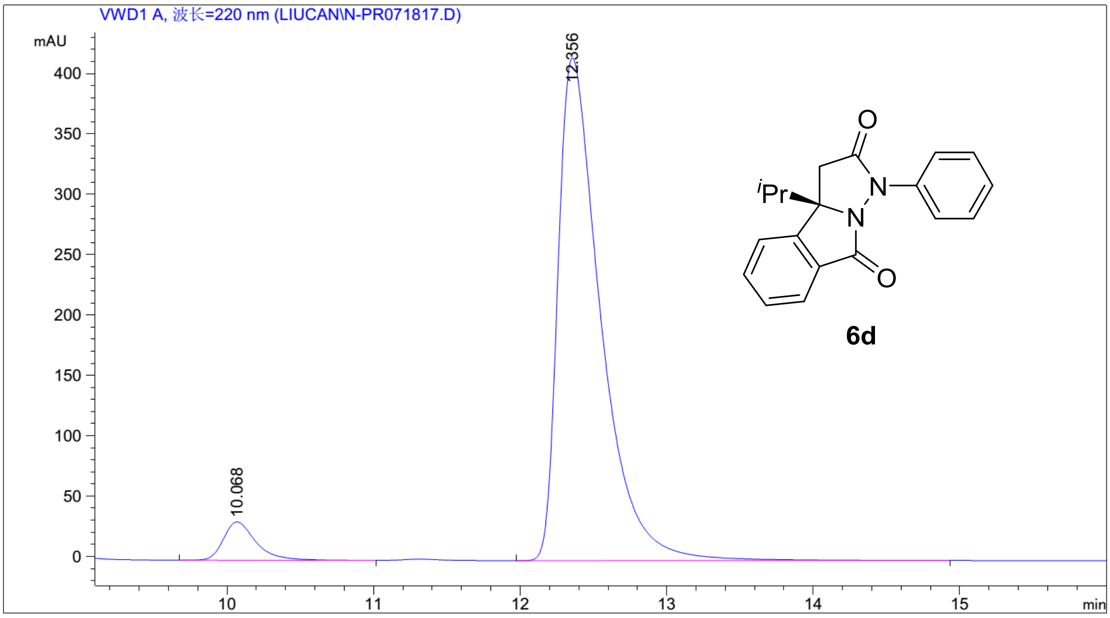

Peak #	RetTime [min]	Type	Width [min]	Area mAU *s	Height [mAU]	Area %
1	10.068	BB	0.2325	491.12711	31.81108	5.5219
2	12.356	BB	0.3024	8403.10742	416.37469	94.4781

Figure S97. HPLC of (*S*)-6d, related to Figure 6.

12. NMR Spectra of M-1, M-2, M-3, M-4, (*S*)-E, (*S*)-F, (*S*)-G, 1a-ah, 2a-ah, 3, 4, 5 and 6

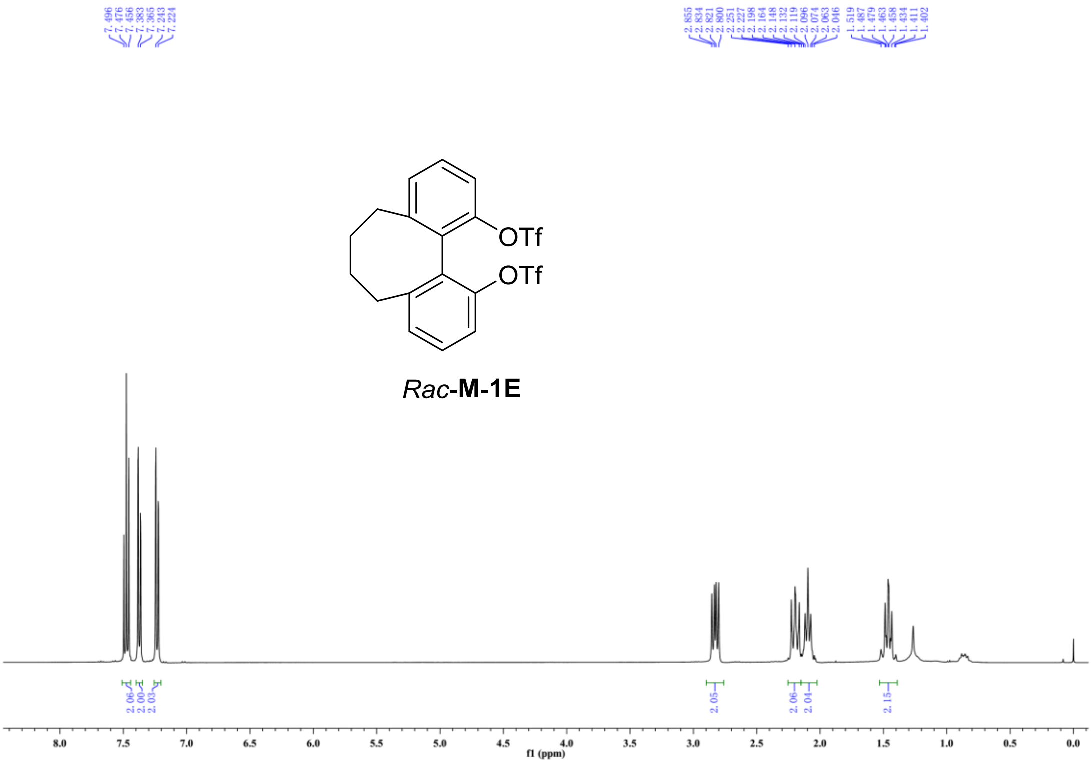

Figure S98. ^{1}H NMR of *Rac*-M-1E, related to Figure 2.

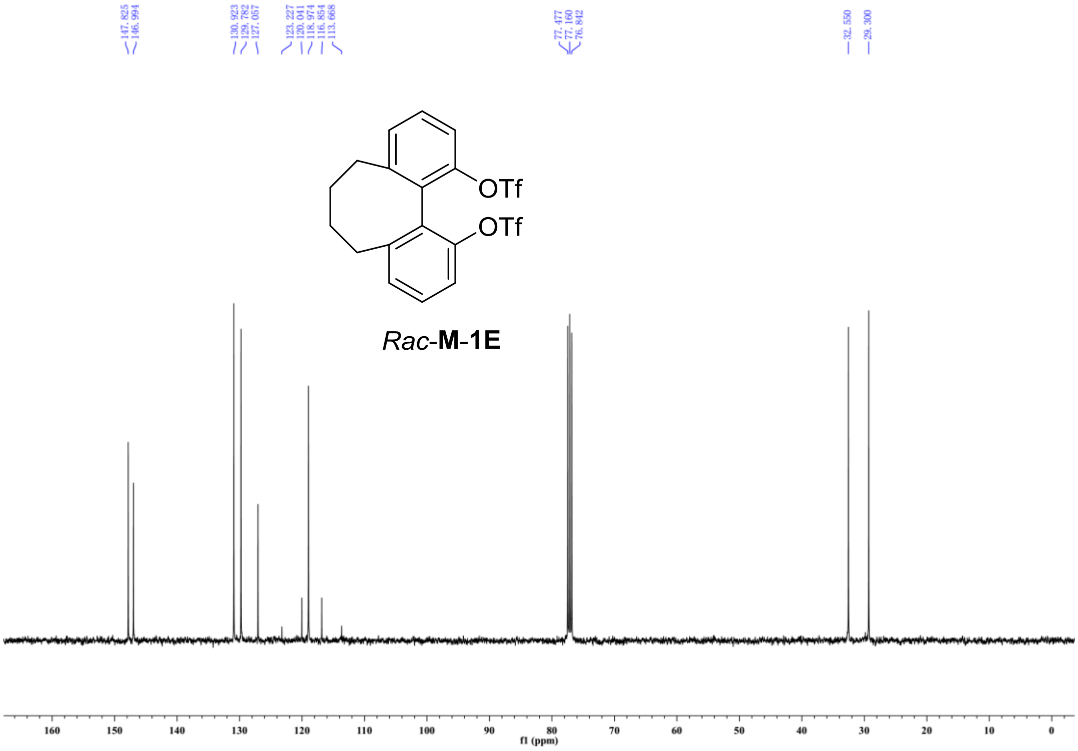

Figure S99. ^{13}C NMR of *Rac*-M-1E, related to Figure 2.

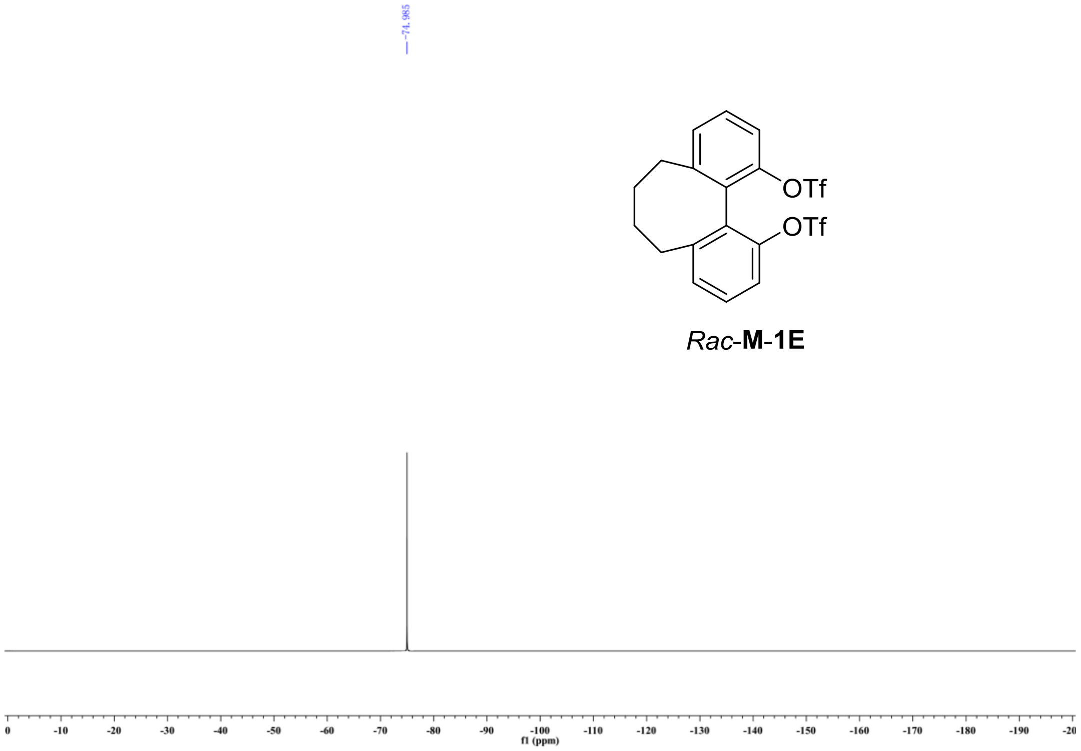

Figure S100. ^{19}F NMR of *Rac*-M-1E, related to Figure 2.

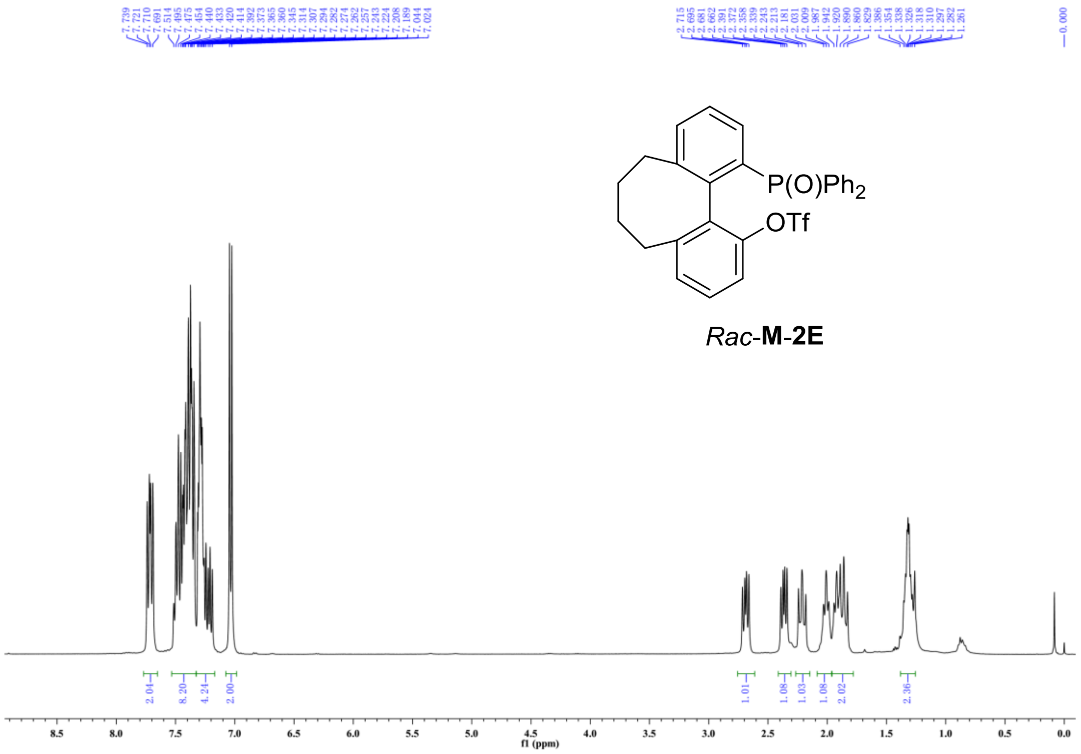

Figure S101. ^{1}H NMR of *Rac*-M-2E, related to Figure 2.

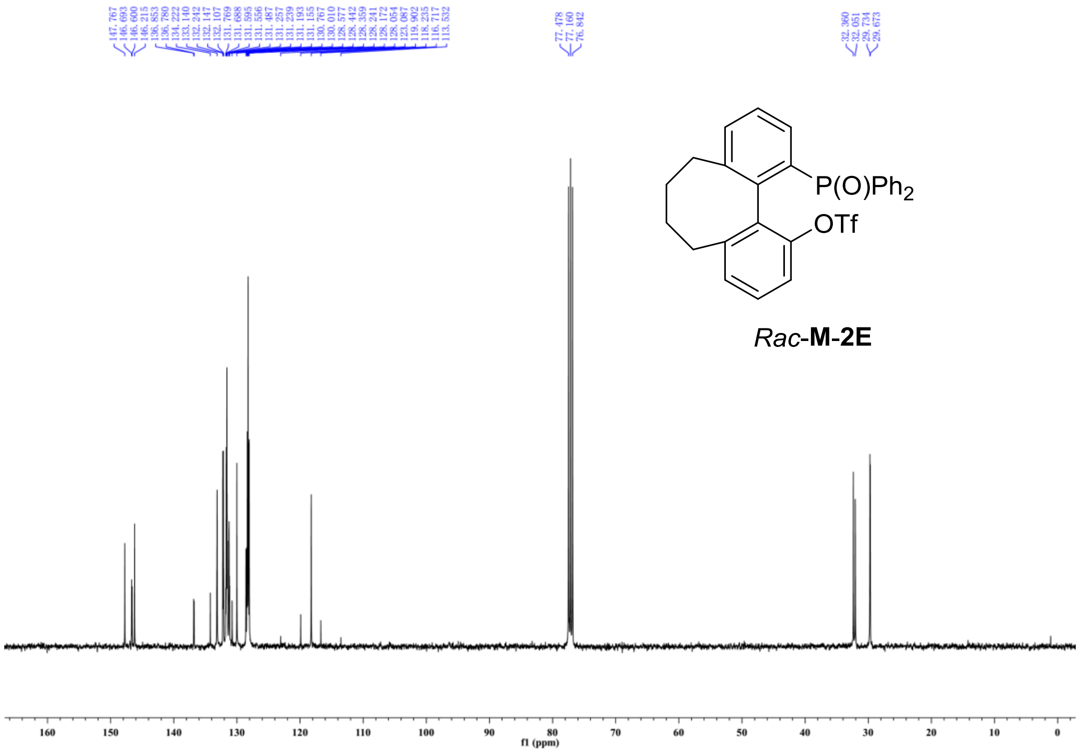

Figure S102. ^{13}**C NMR of** ***Rac*****-M-2E, related to Figure 2.**

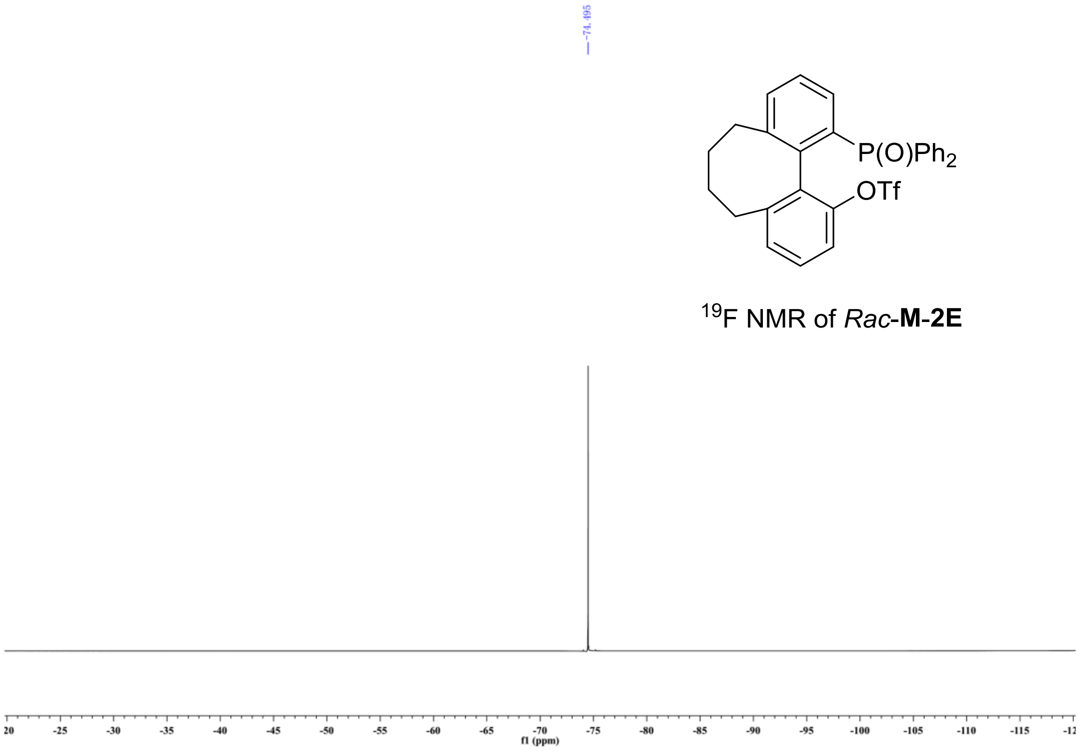

Figure S103. ^{19}**F NMR of** ***Rac*****-M-2E, related to Figure 2.**

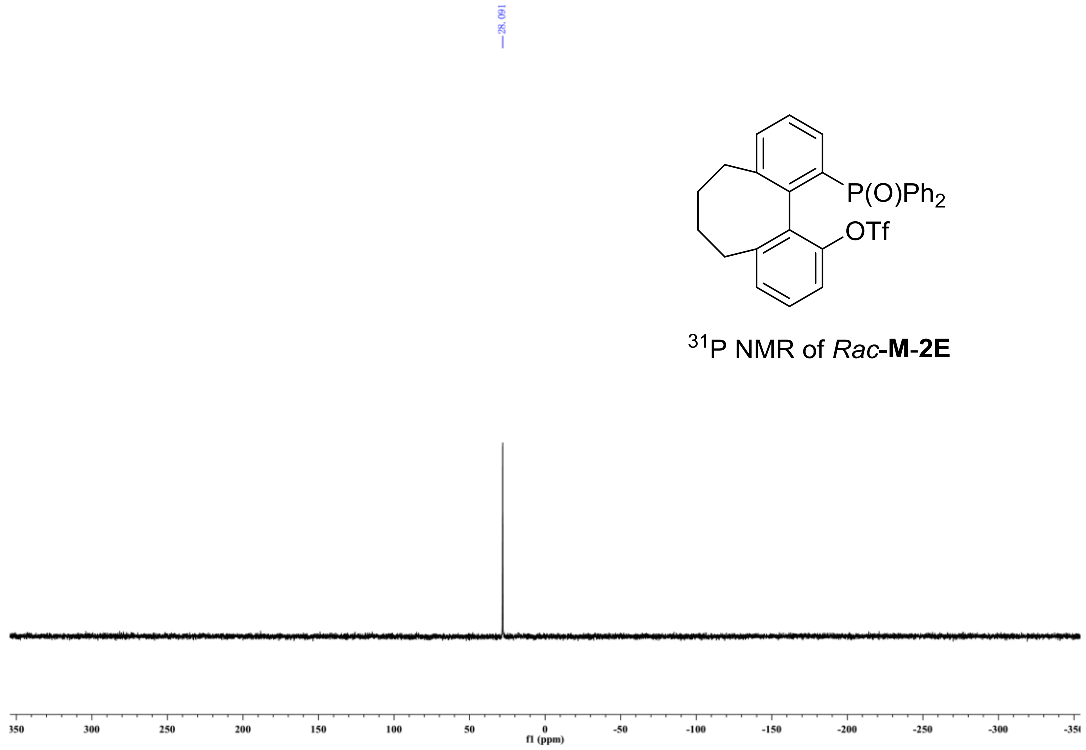

Figure S104. ^{31}P NMR of *Rac*-M-2E, related to Figure 2.

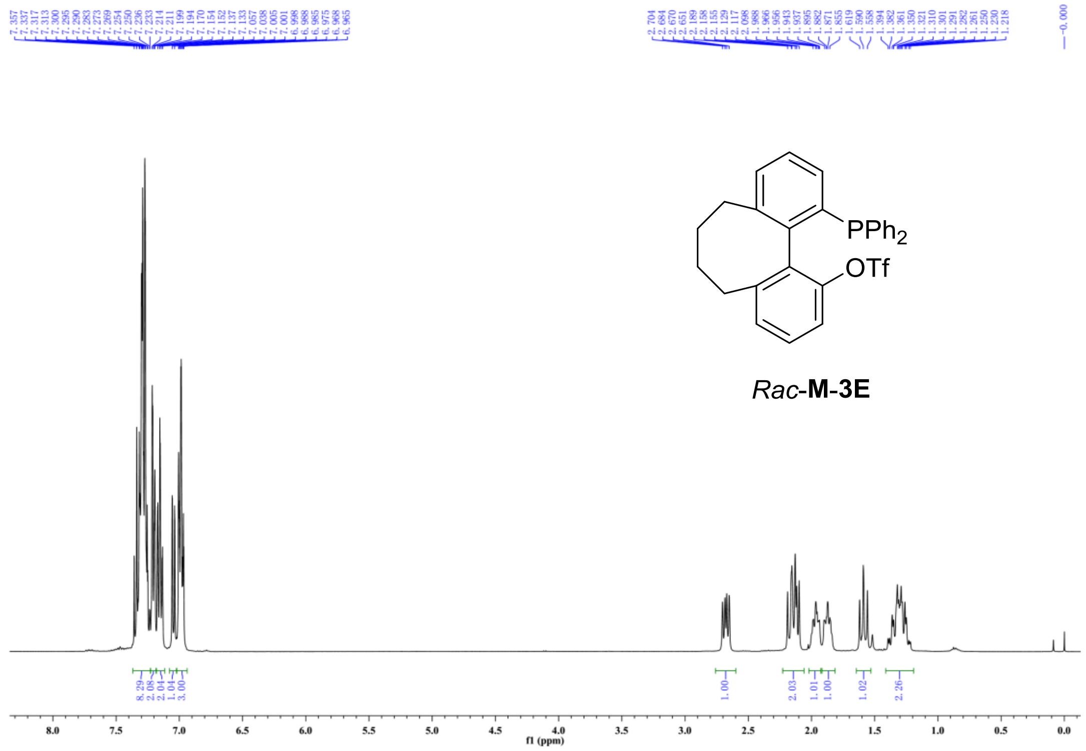

Figure S105. ^{1}H NMR of *Rac*-M-3E, related to Figure 2.

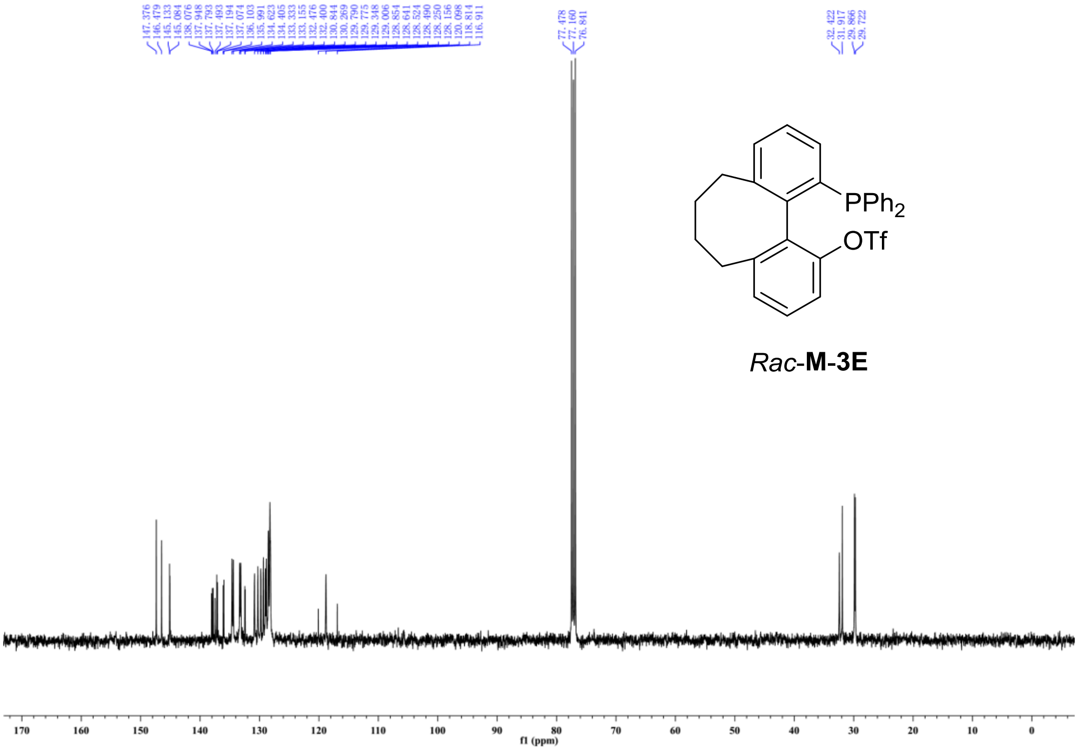

Figure S106. ^{13}C NMR of *Rac*-M-3E, related to Figure 2.

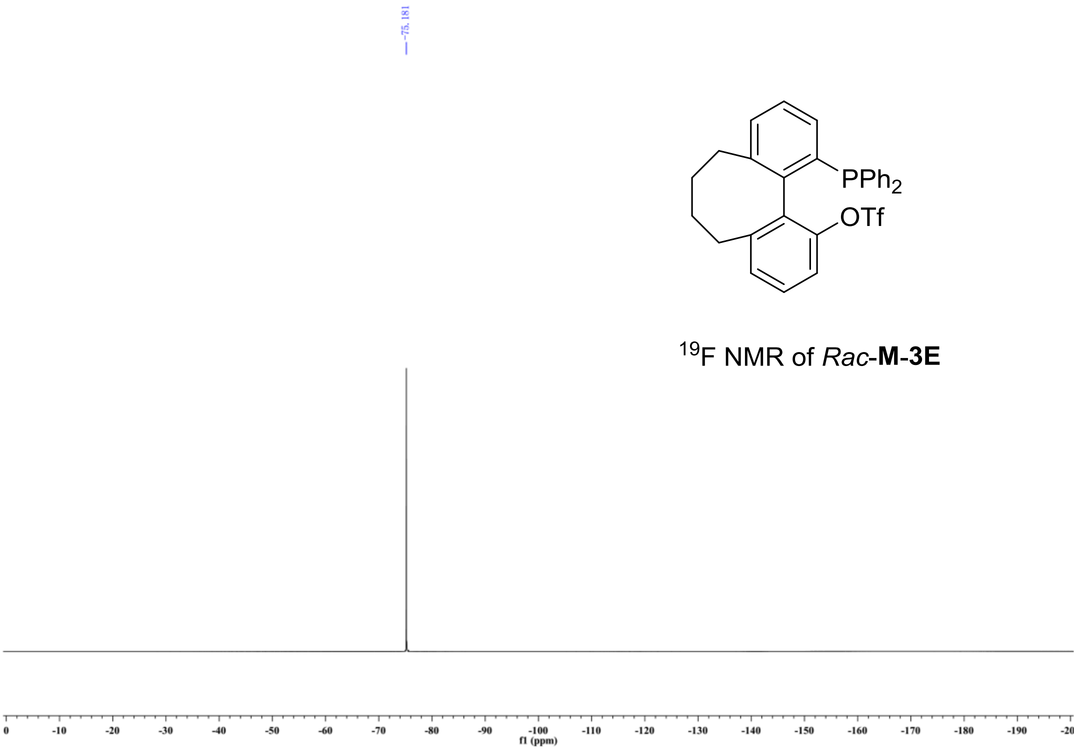

Figure S107. ^{19}F NMR of *Rac*-M-3E, related to Figure 2.

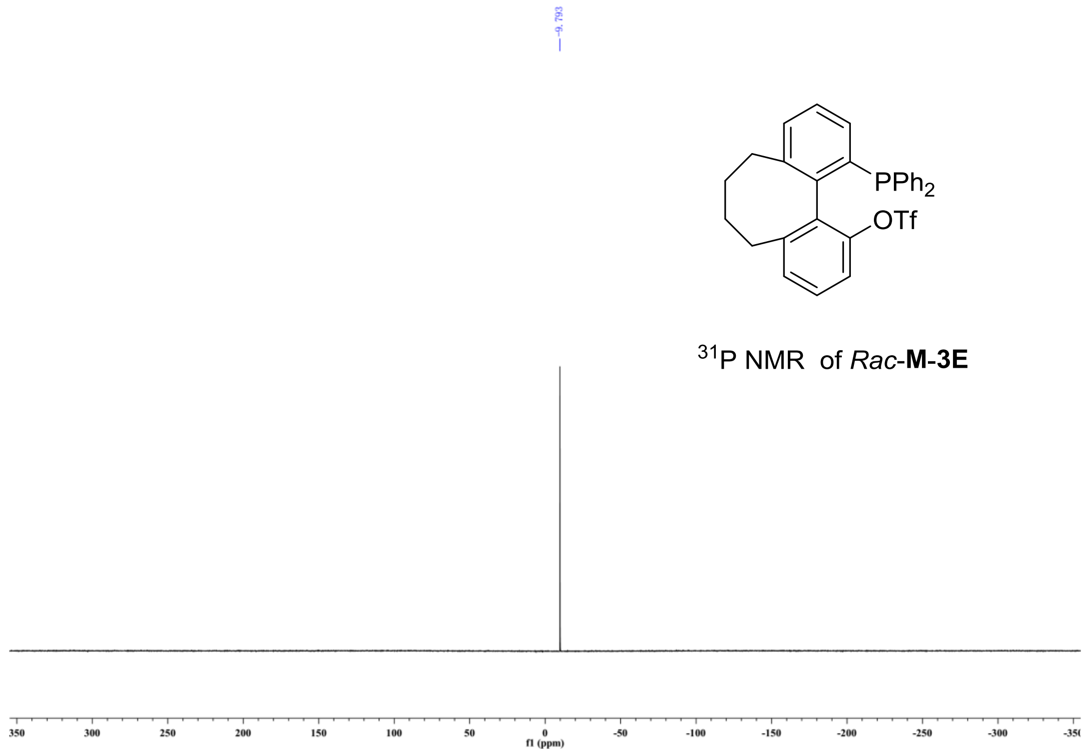

Figure S108. ^{31}P NMR of *Rac*-M-3E, related to Figure 2.

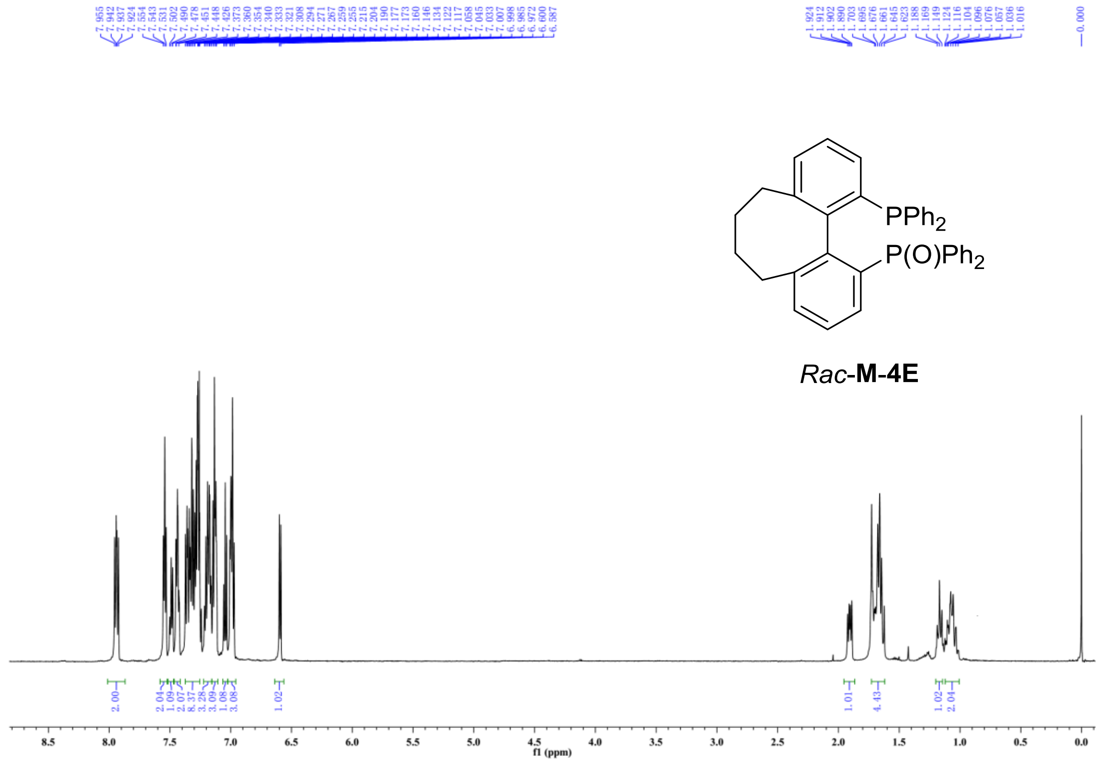

Figure S109. ^{1}H NMR of *Rac*-M-4E, related to Figure 2.

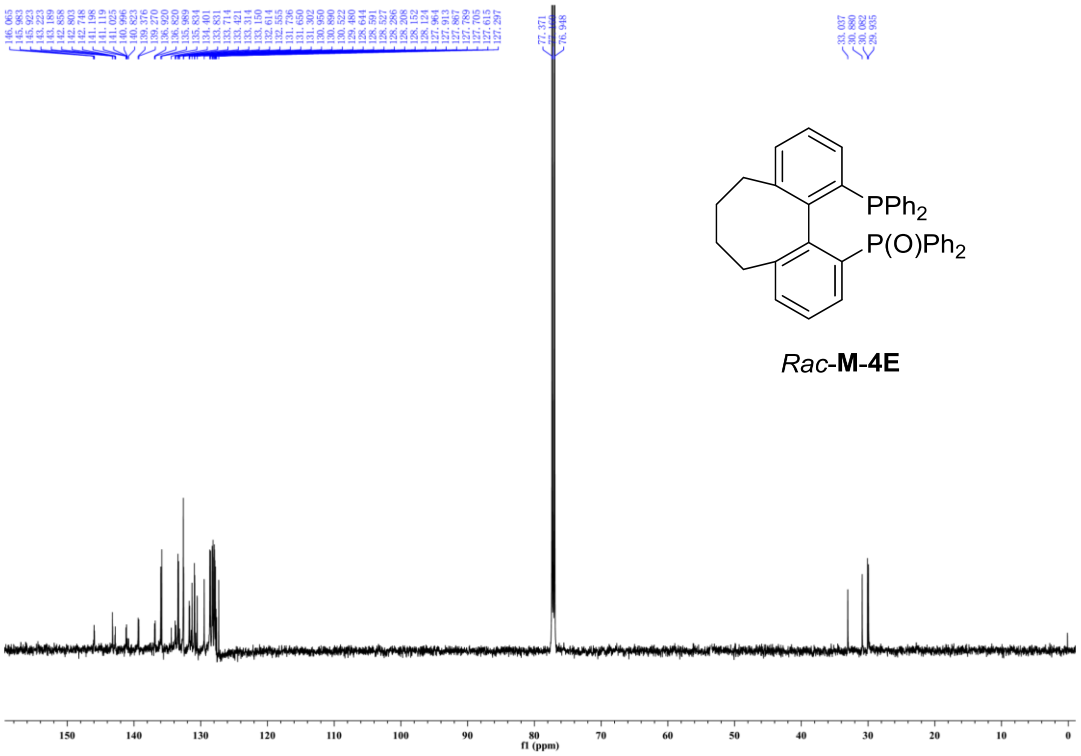

Figure S110. ^{13}C **NMR of *Rac*-M-4E, related to Figure 2.**

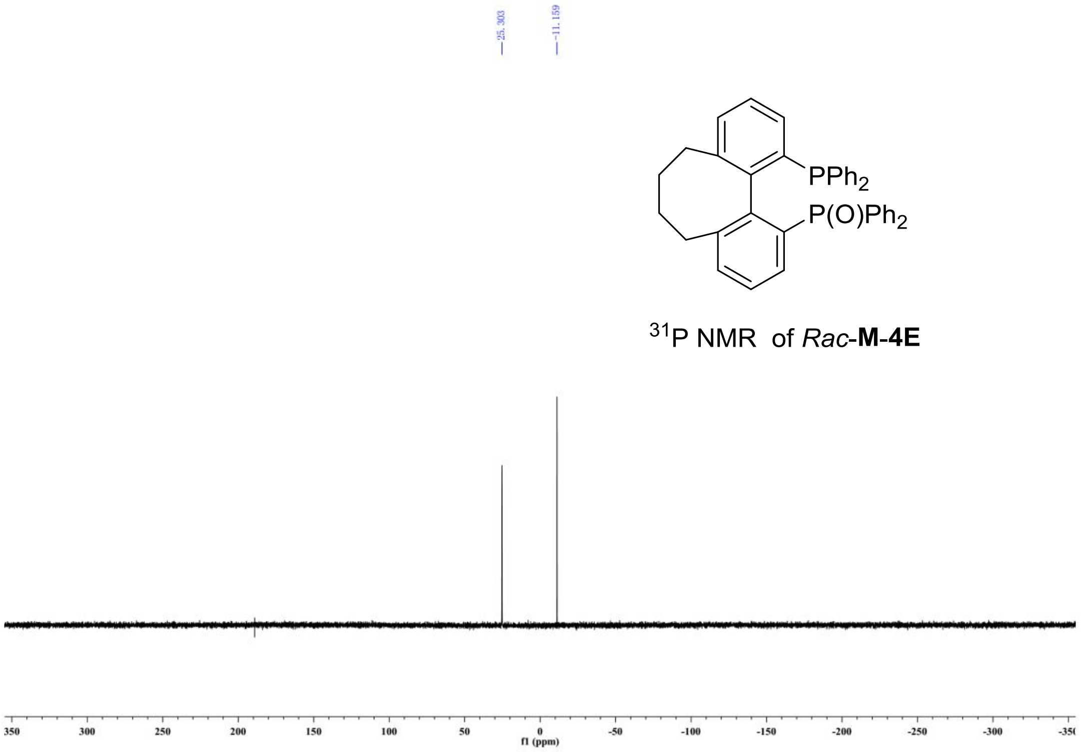

Figure S111. ^{31}P **NMR of *Rac*-M-4E, related to Figure 2.**

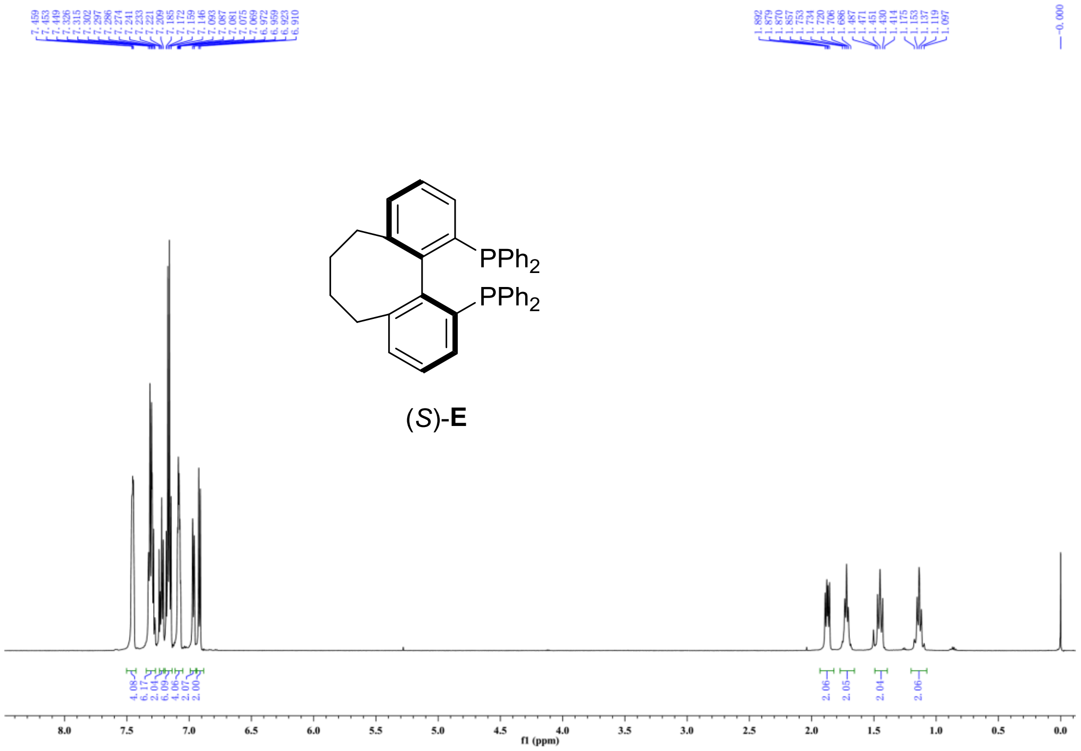

Figure S112. ^{1}**H NMR of (*S*)-E, related to Figure 2.**

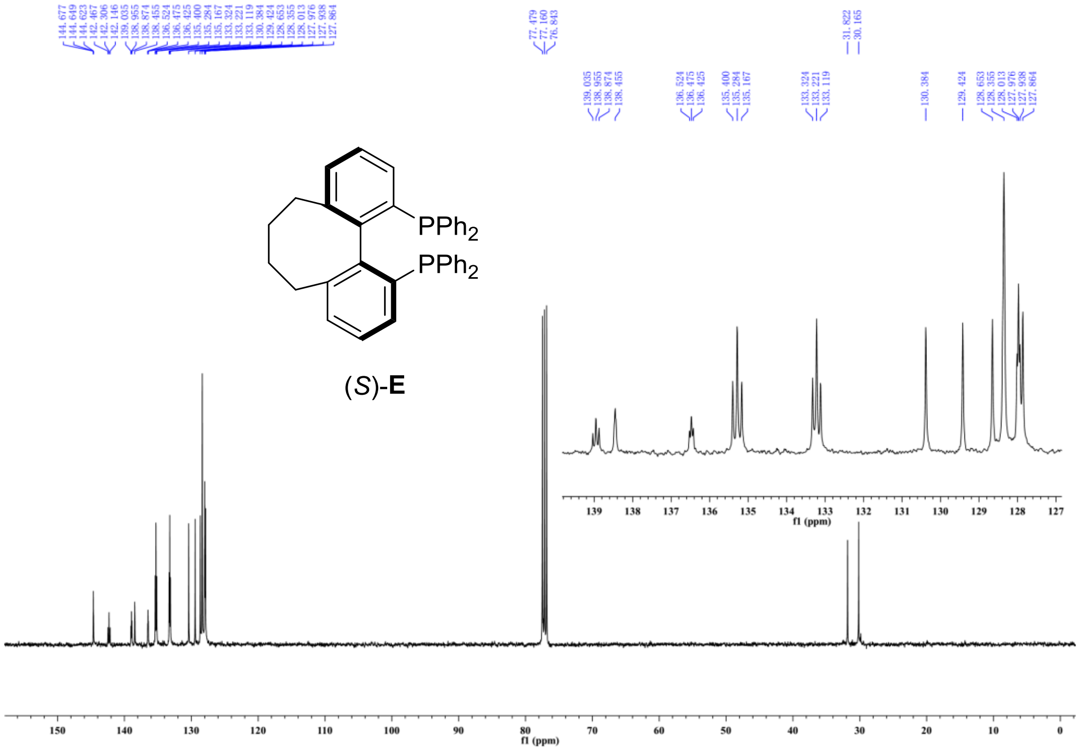

Figure S113. ^{13}**C NMR of (*S*)-E, related to Figure 2.**

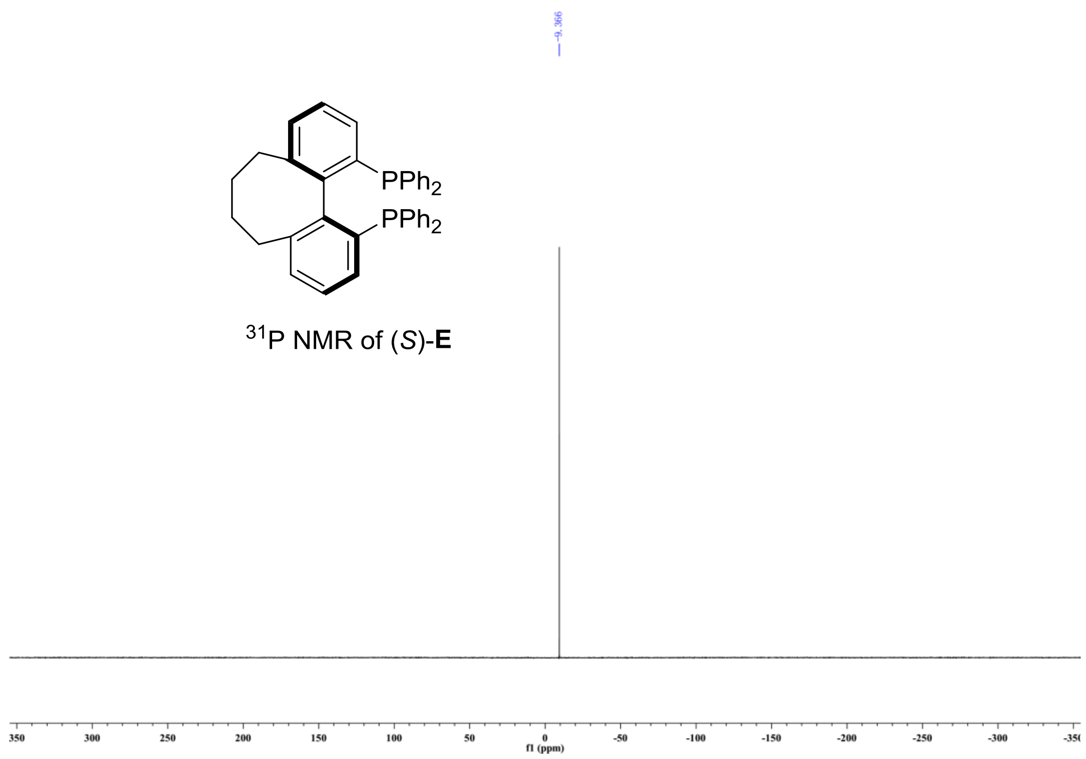

Figure S114. **^{31}P NMR of (*S*)-E, related to Figure 2.**

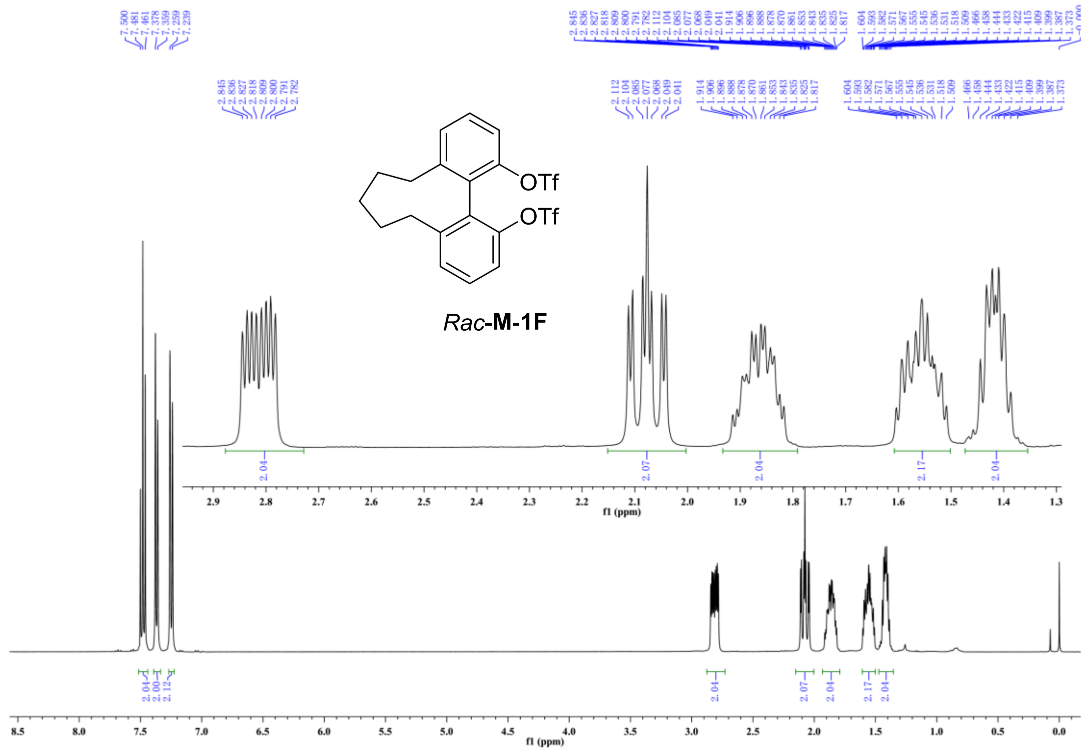

Figure S115. **^{1}H NMR of *Rac*-M-1F, related to Figure 2.**

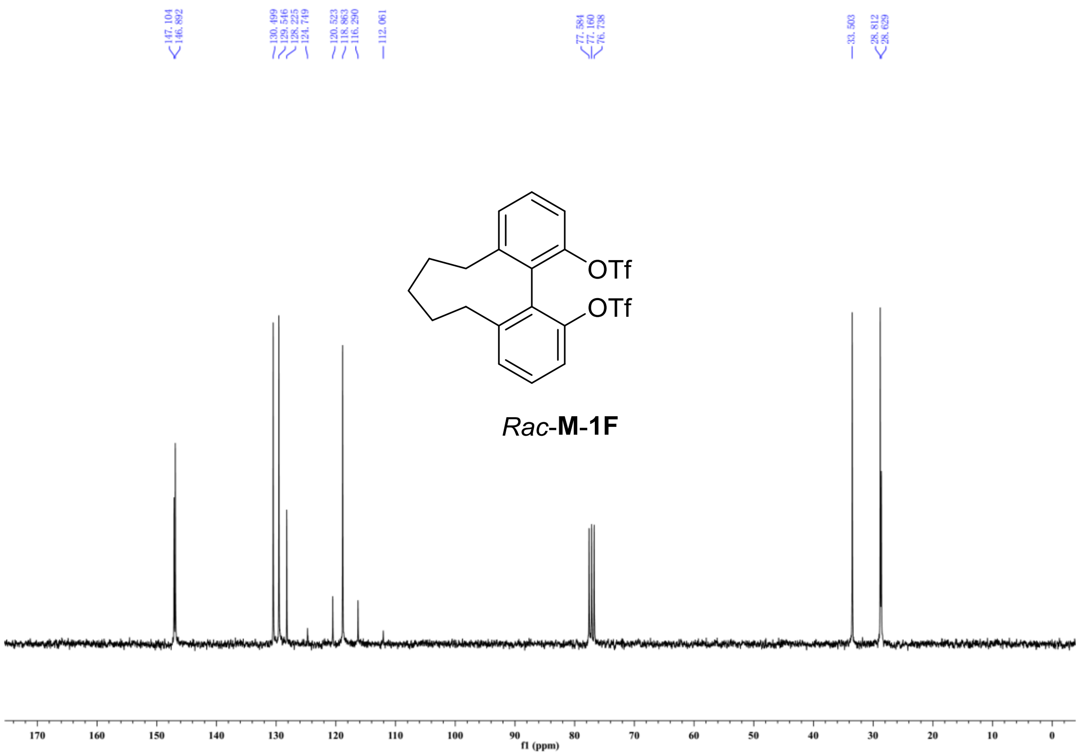

Figure S116. ^{13}C **NMR of** ***Rac*-M-1F, related to Figure 2.**

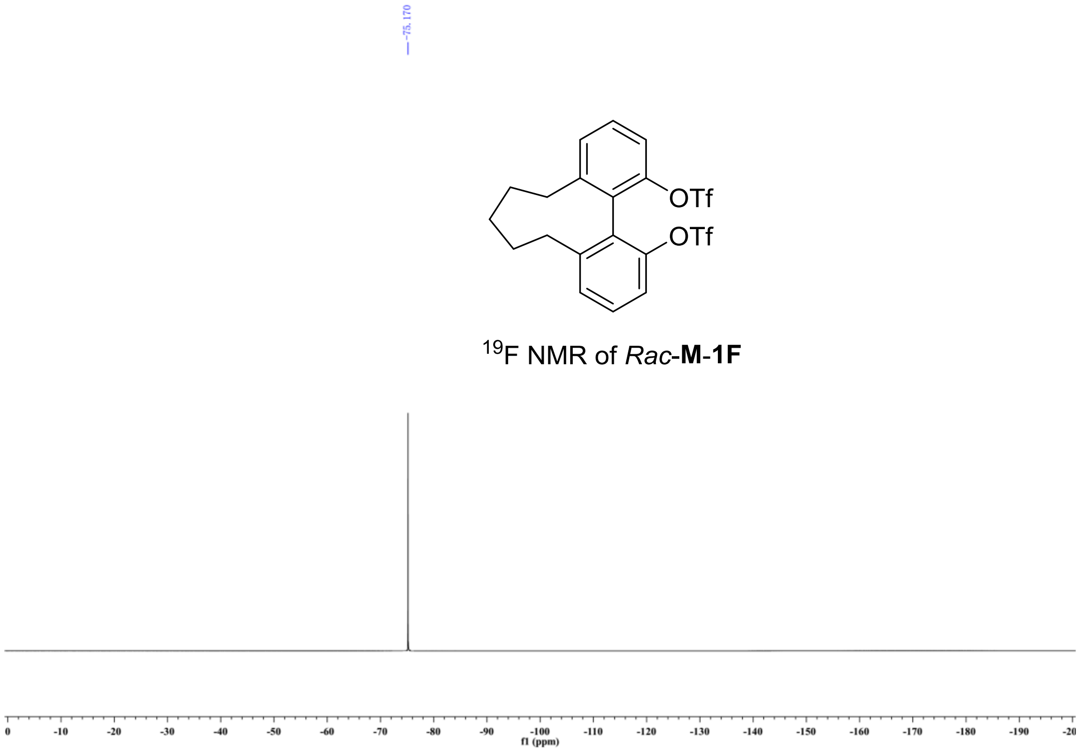

Figure S117. ^{19}F **NMR of** ***Rac*-M-1F, related to Figure 2.**

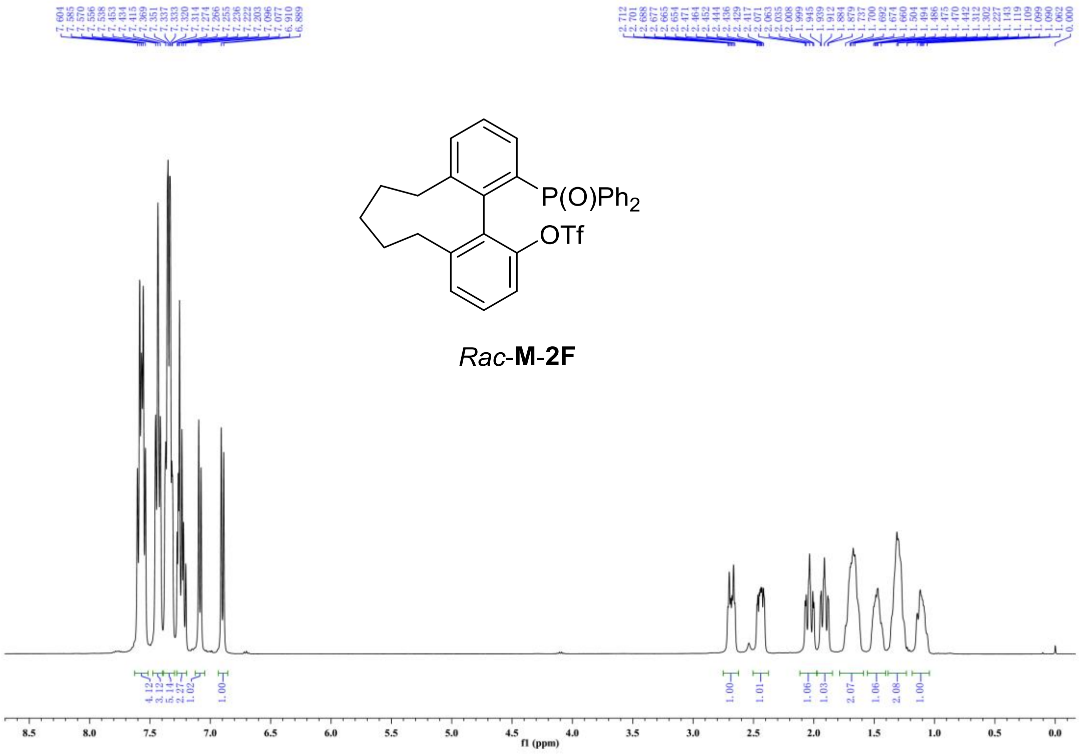

Figure S118. ¹H NMR of *Rac*-M-2F, related to Figure 2.

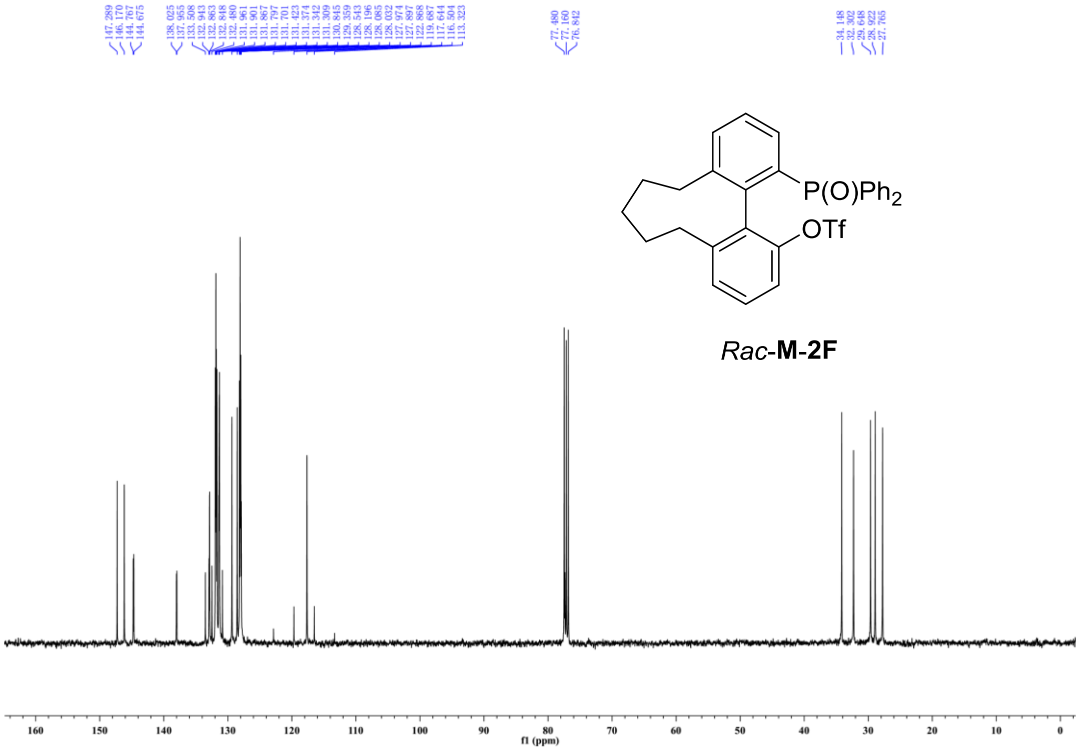

Figure S119. ¹³C NMR of *Rac*-M-2F, related to Figure 2.

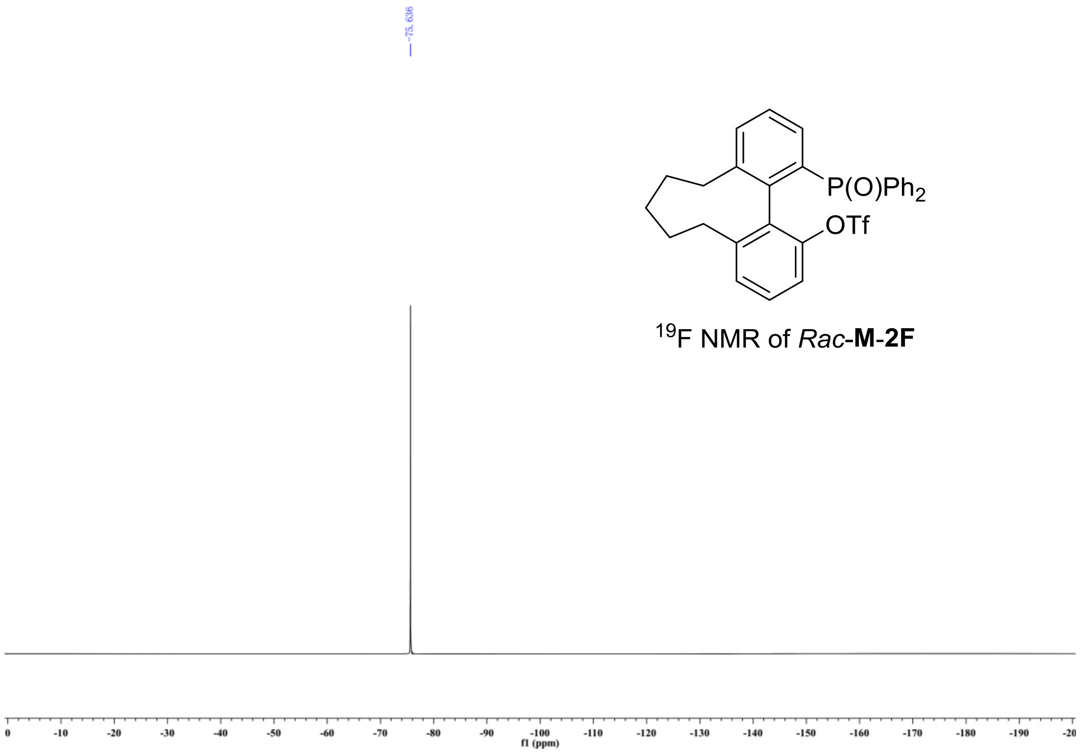

Figure S120. ^{19}**F NMR of** ***Rac*****-M-2F, related to Figure 2.**

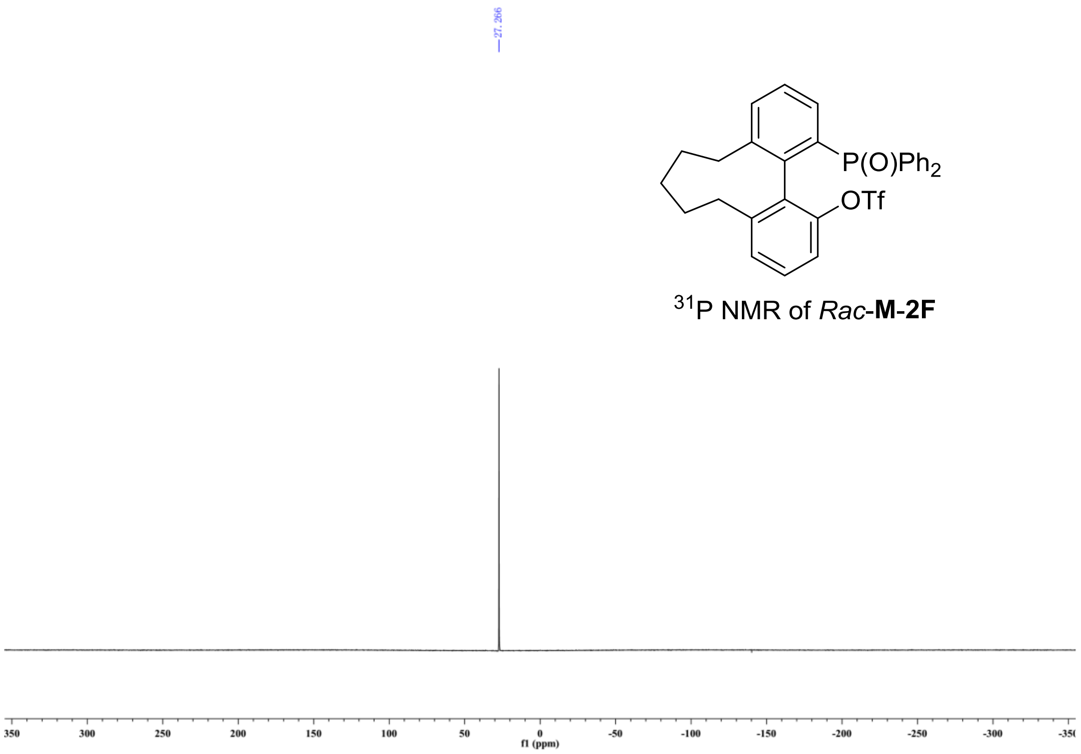

Figure S121. ^{31}**P NMR of** ***Rac*****-M-2F, related to Figure 2.**

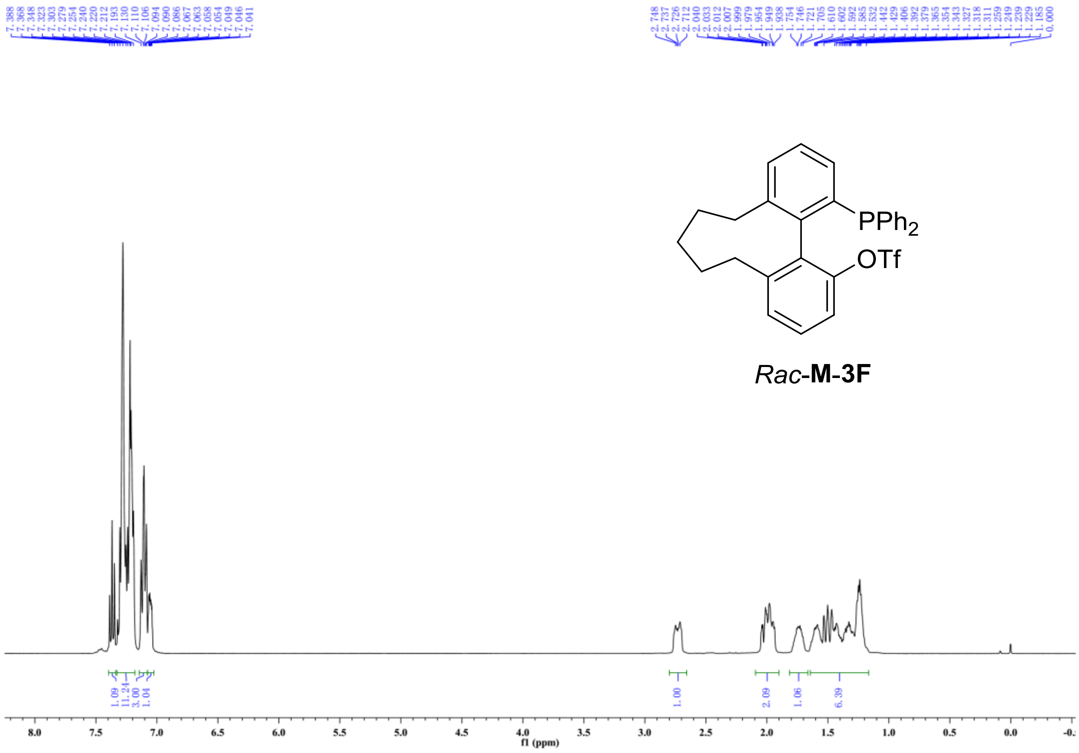

Figure S122. ^{1}H NMR of *Rac*-M-3F, related to Figure 2.

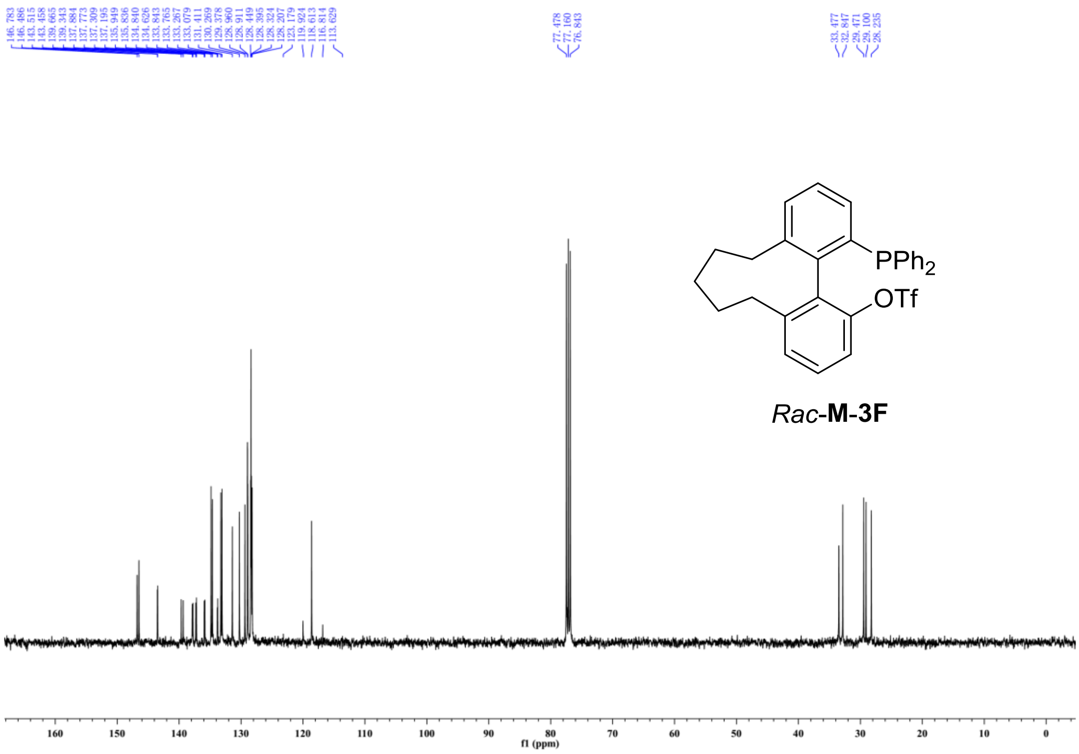

Figure S123. ^{13}C NMR of *Rac*-M-3F, related to Figure 2.

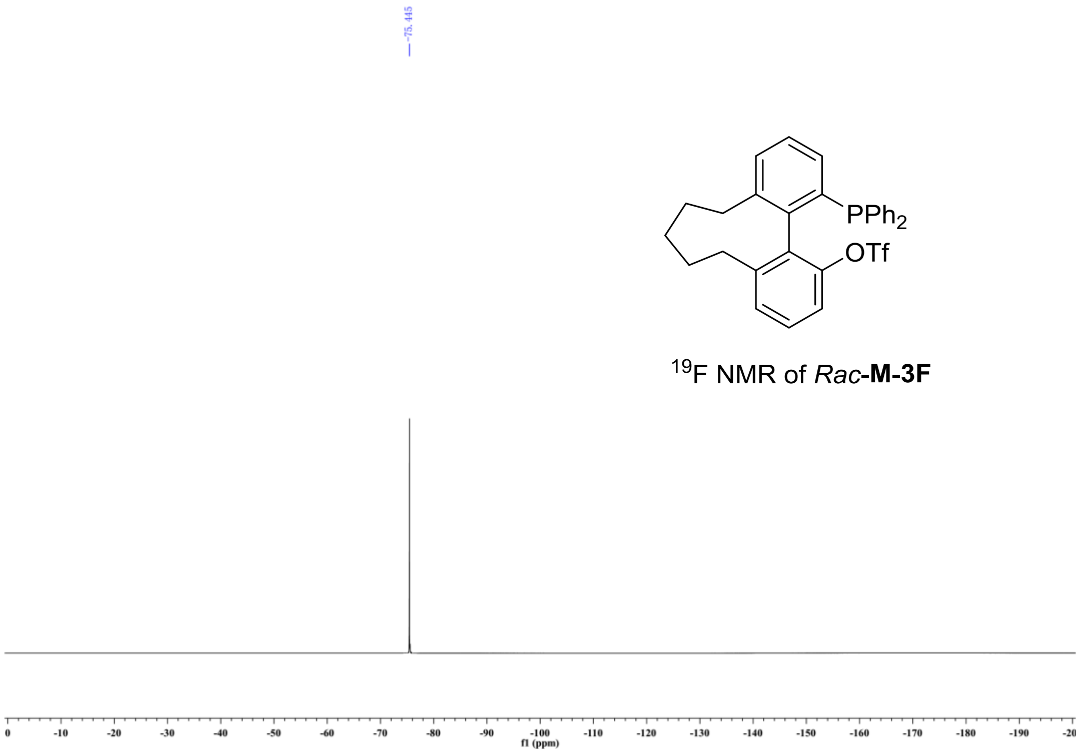

Figure S124. ^{19}F NMR of *Rac*-M-3F, related to Figure 2.

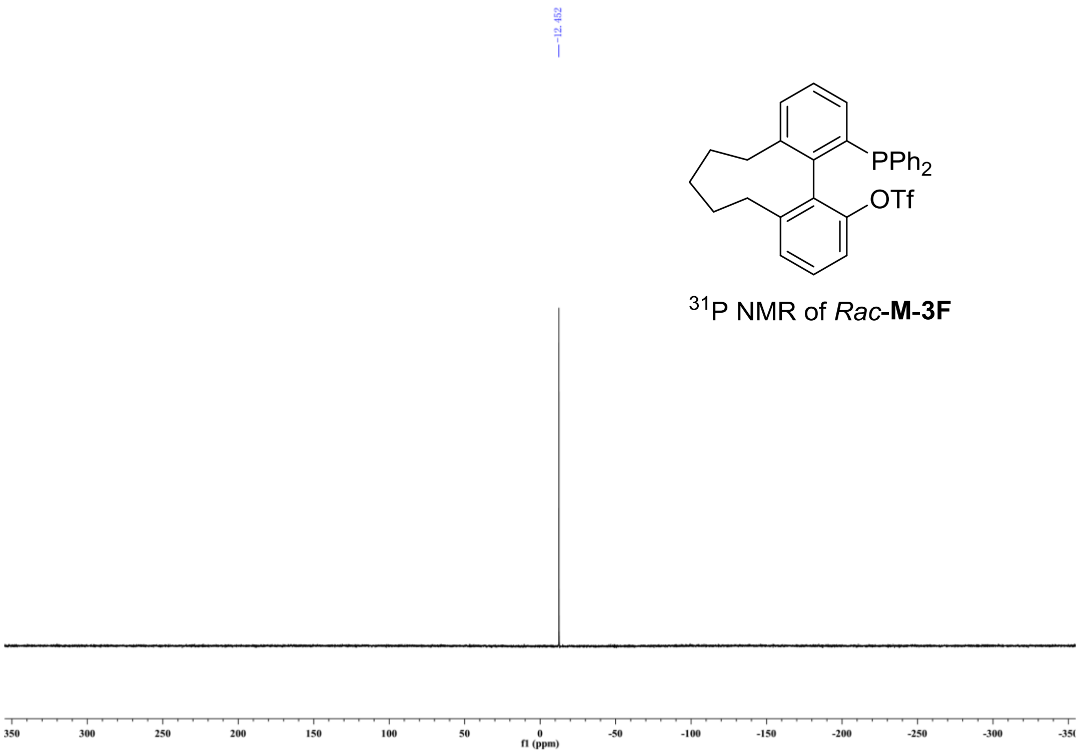

Figure S125. ^{31}P NMR of *Rac*-M-3F, related to Figure 2.

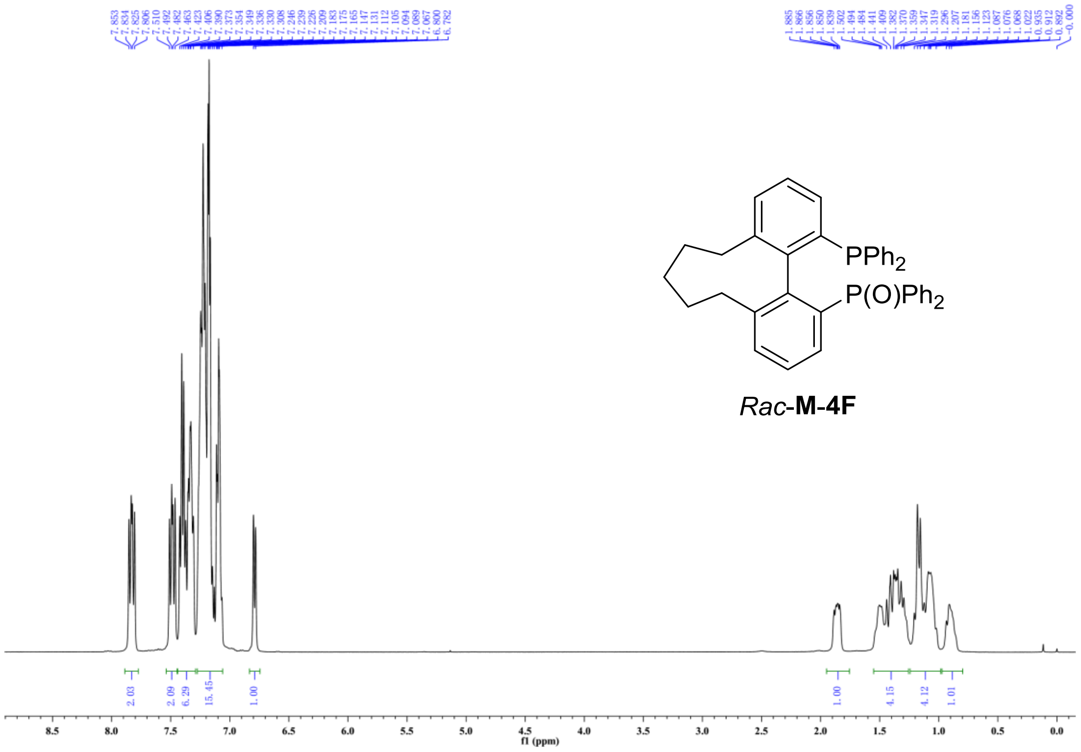

Figure S126. ^{1}H NMR of *Rac*-M-4F, related to Figure 2.

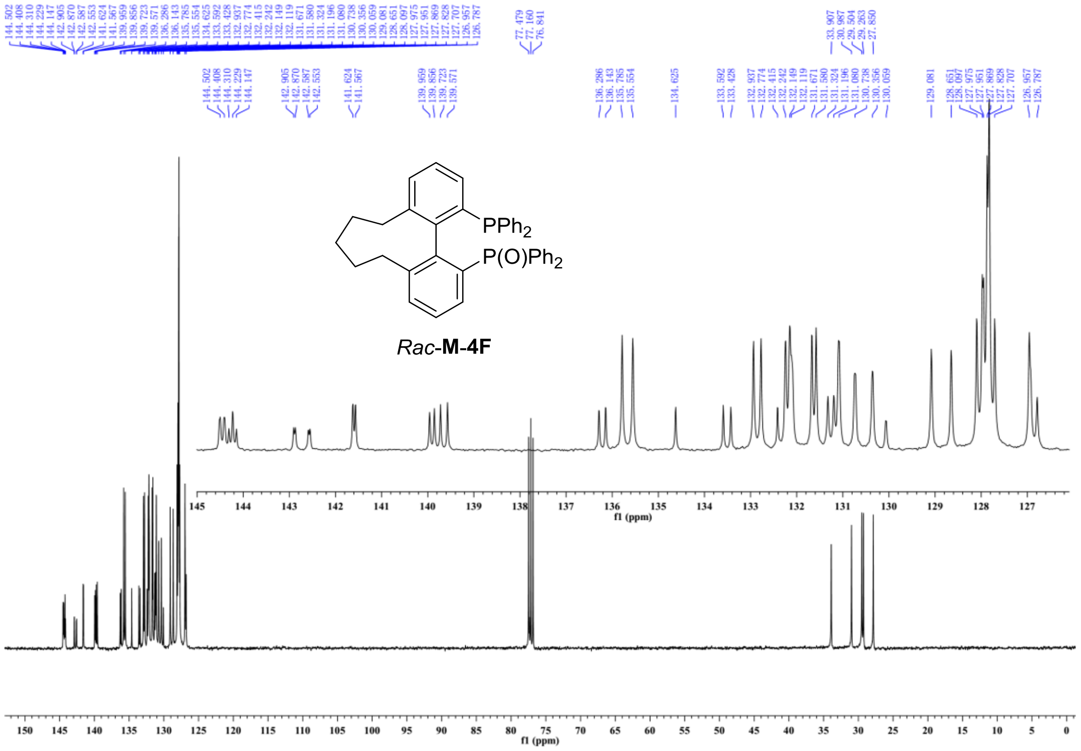

Figure S127. ^{13}C NMR of *Rac*-M-4F, related to Figure 2.

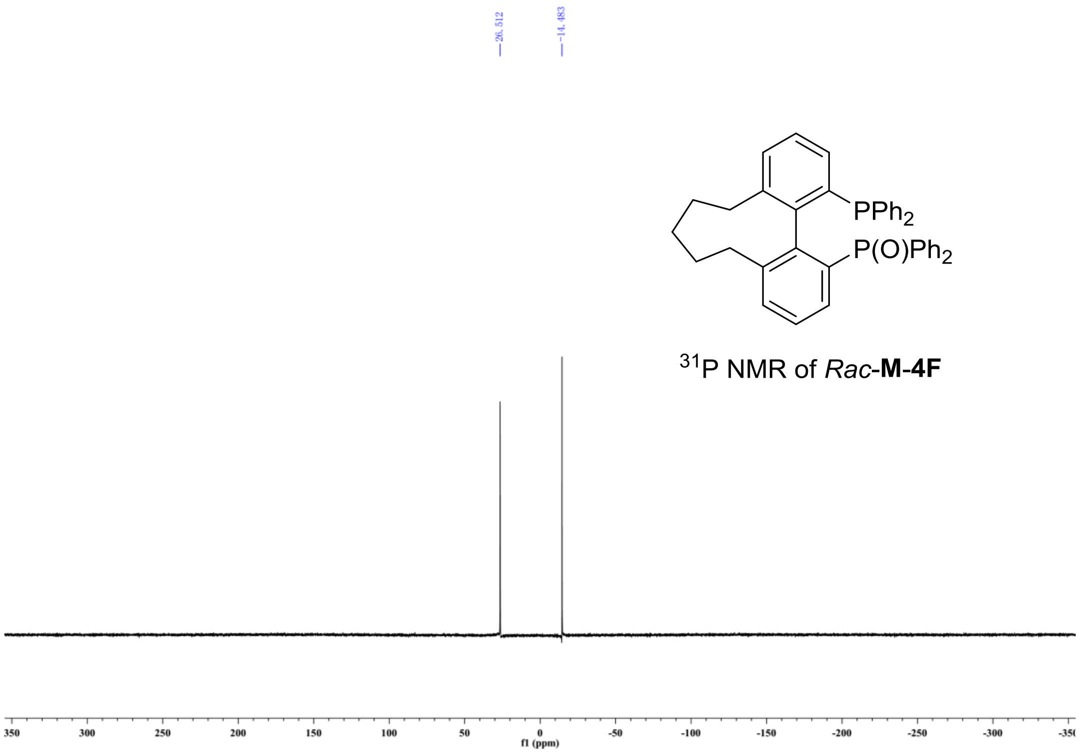

Figure S128. ^{31}P NMR of *Rac*-M-4F, related to Figure 2.

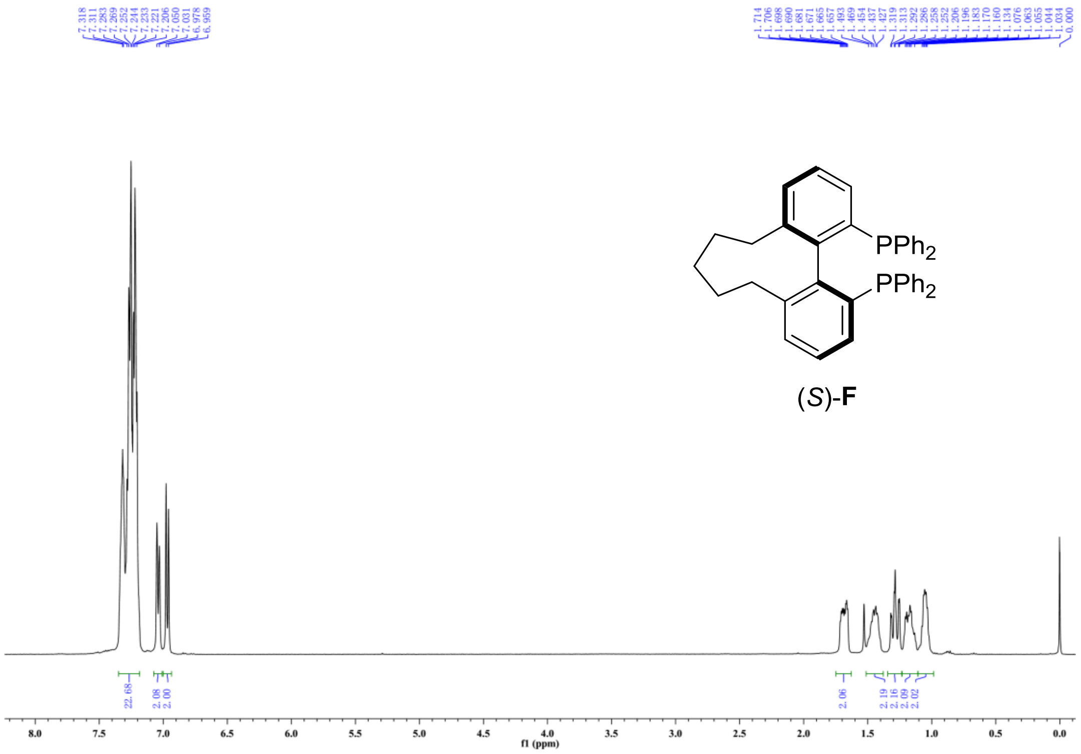

Figure S129. ^{1}H NMR of (*S*)-F, related to Figure 2.

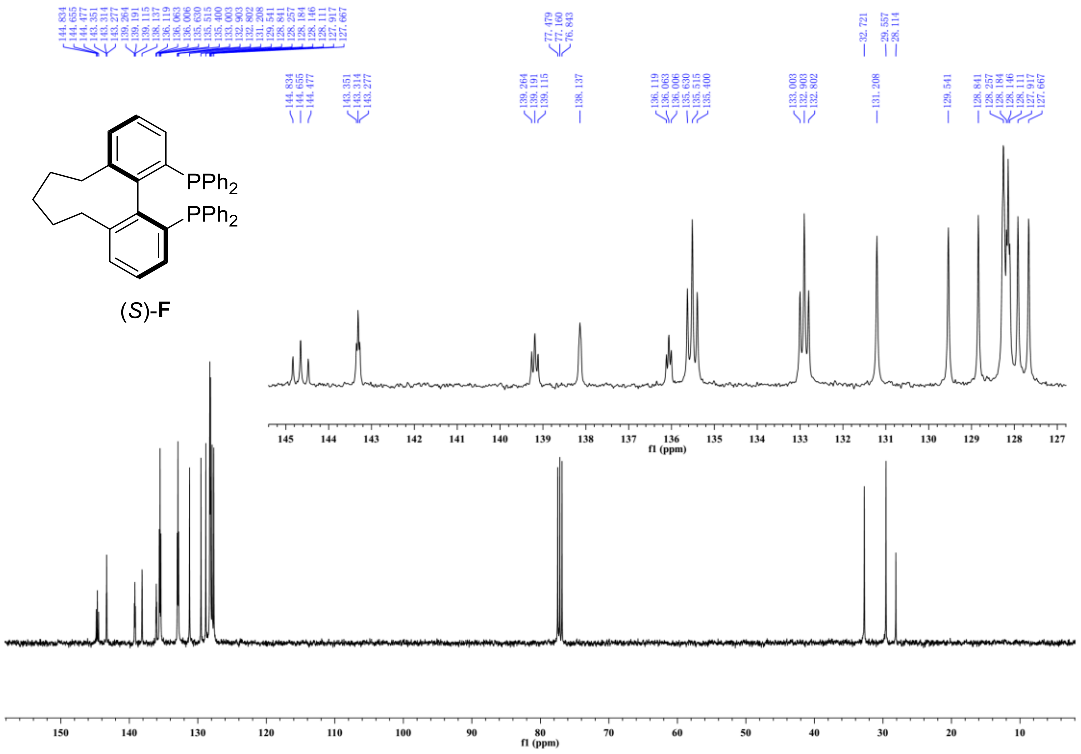

Figure S130. ^{13}**C NMR of (*S*)-F, related to Figure 2.**

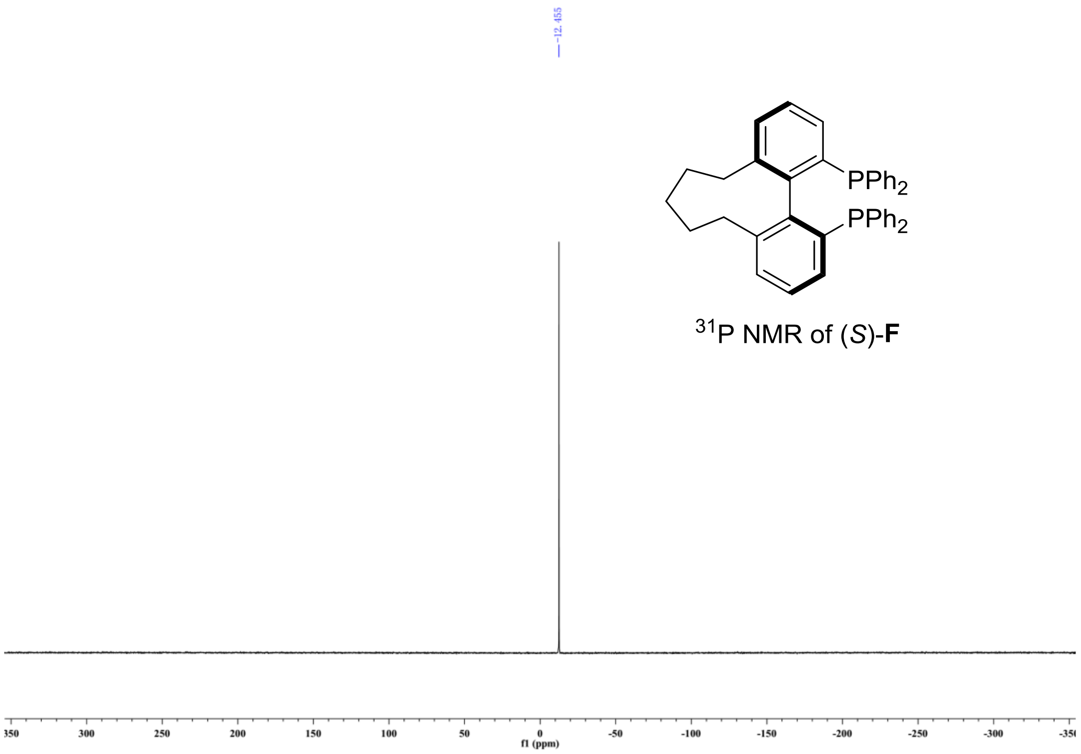

Figure S131. ^{31}**P NMR of (*S*)-F, related to Figure 2.**

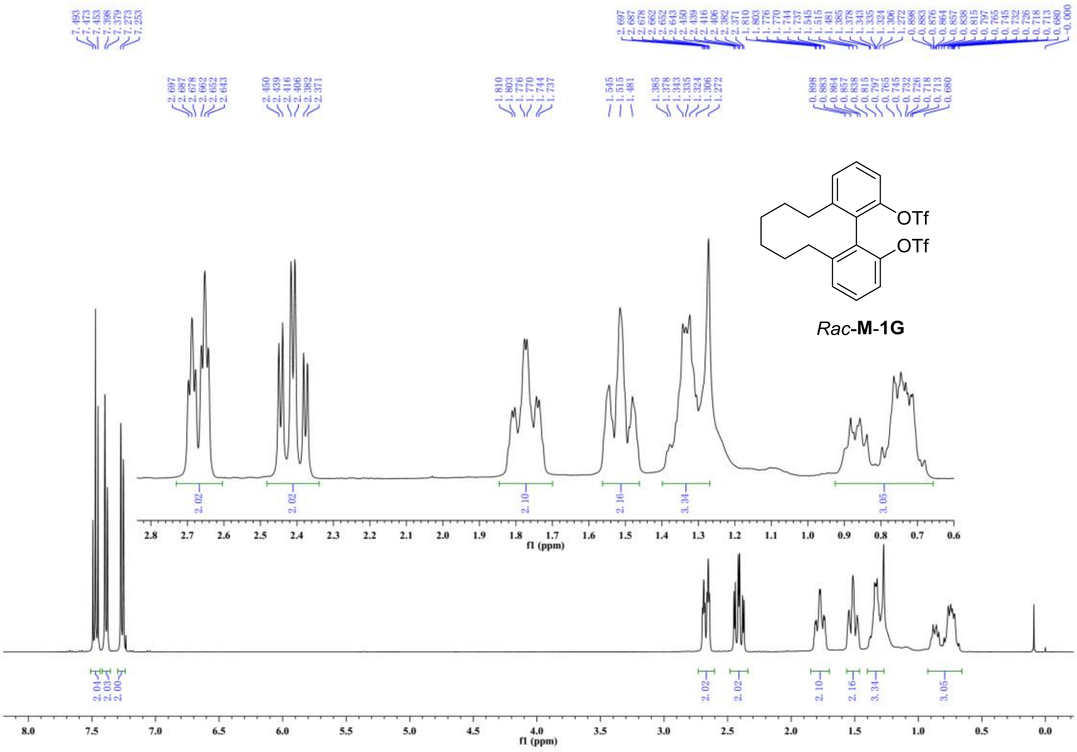

Figure S132. **^{1}H NMR of *Rac*-M-1G, related to Figure 2.**

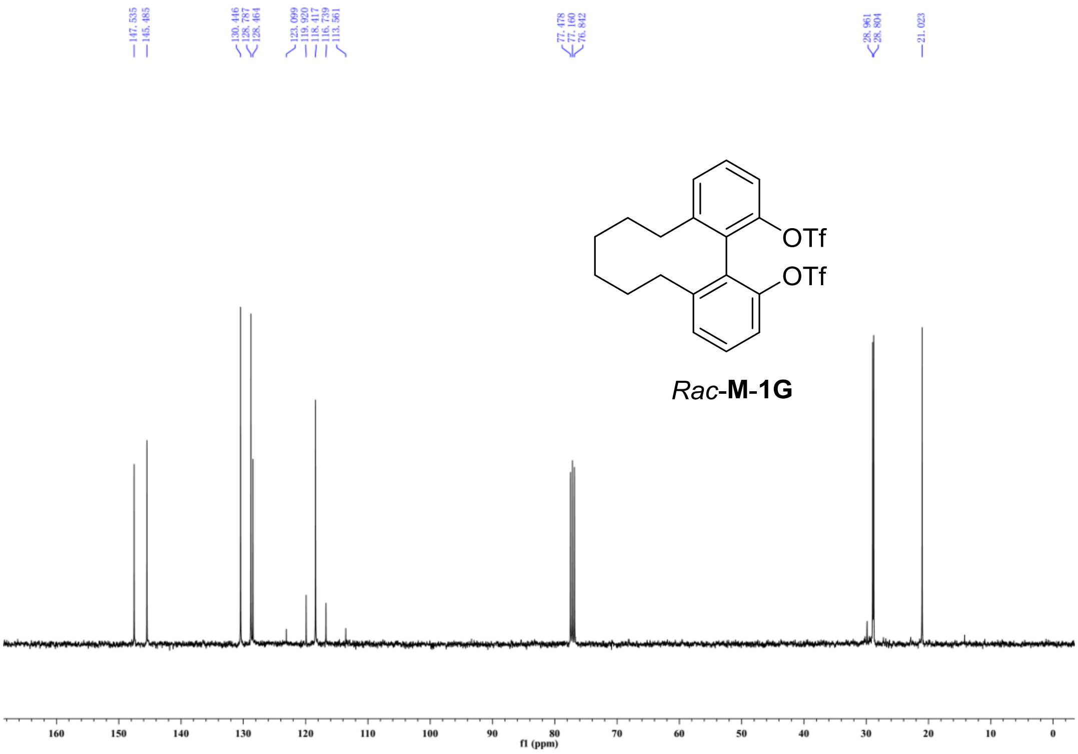

Figure S133. **^{13}C NMR of *Rac*-M-1G, related to Figure 2.**

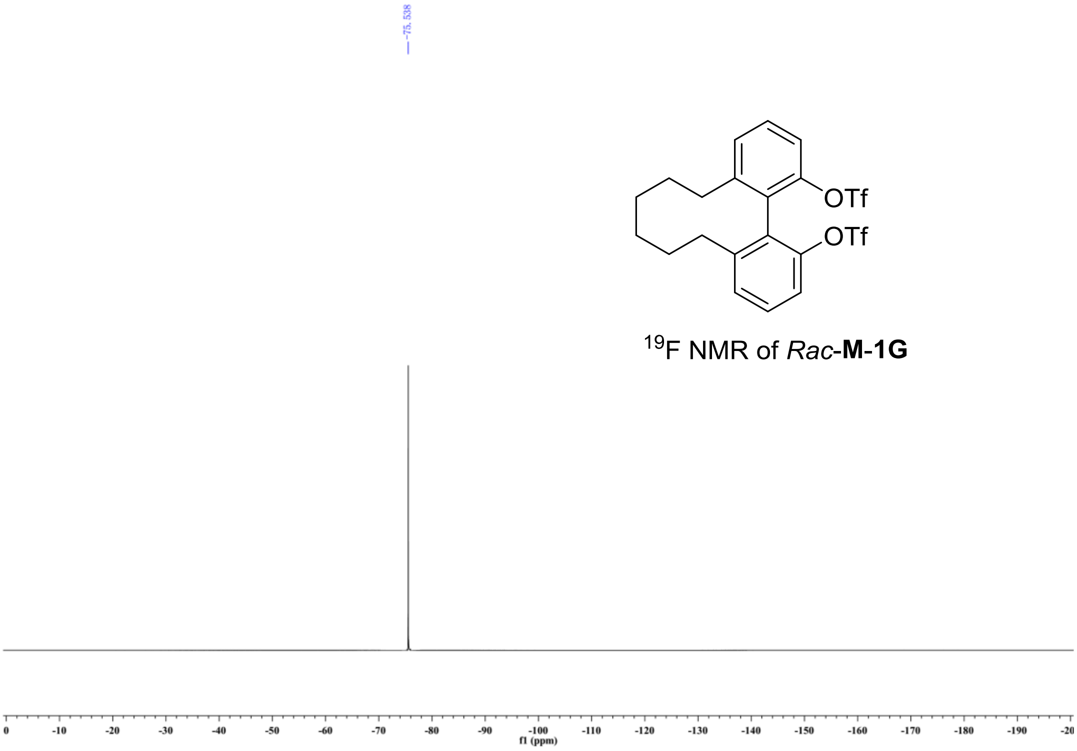

Figure S134. **^{19}F NMR of *Rac*-M-1G, related to Figure 2.**

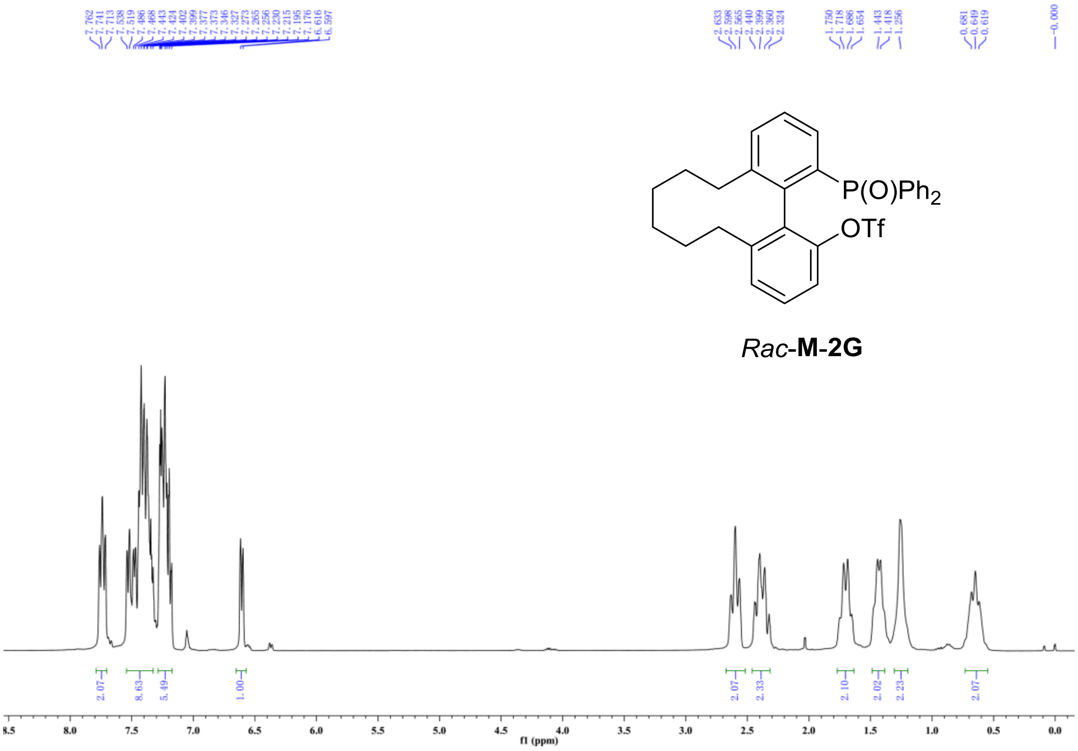

Figure S135. **^{1}H NMR of *Rac*-M-2G, related to Figure 2.**

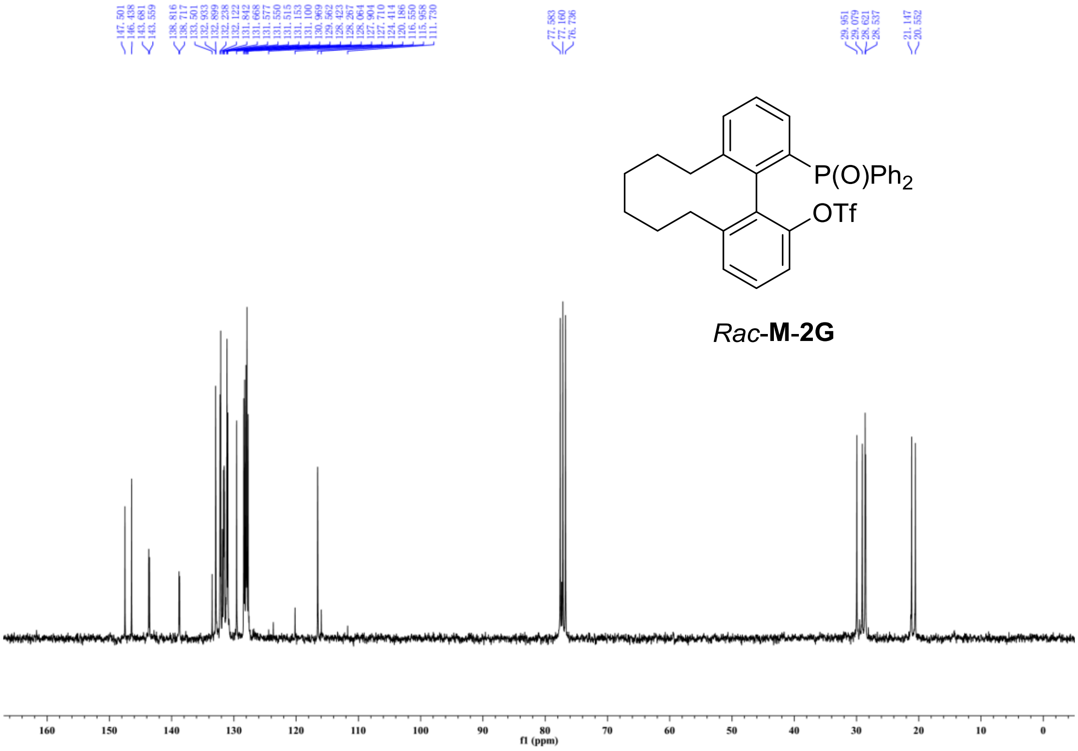

Figure S136. **^{13}C NMR of *Rac*-M-2G, related to Figure 2.**

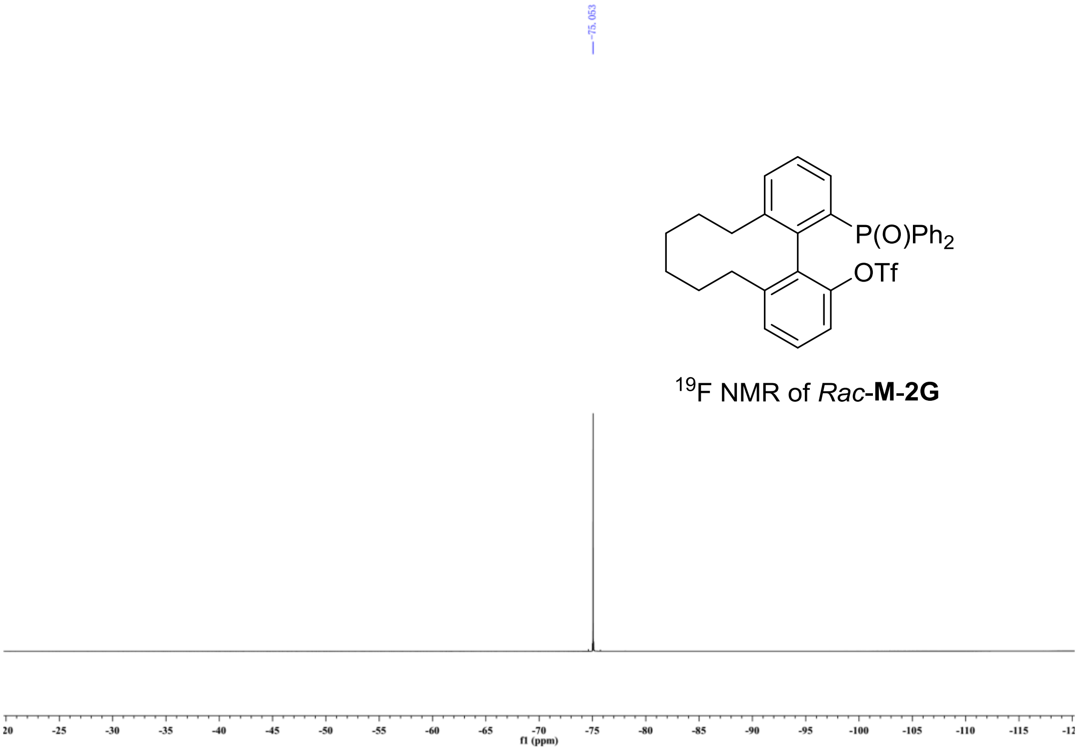

Figure S137. **^{19}F NMR of *Rac*-M-2G, related to Figure 2.**

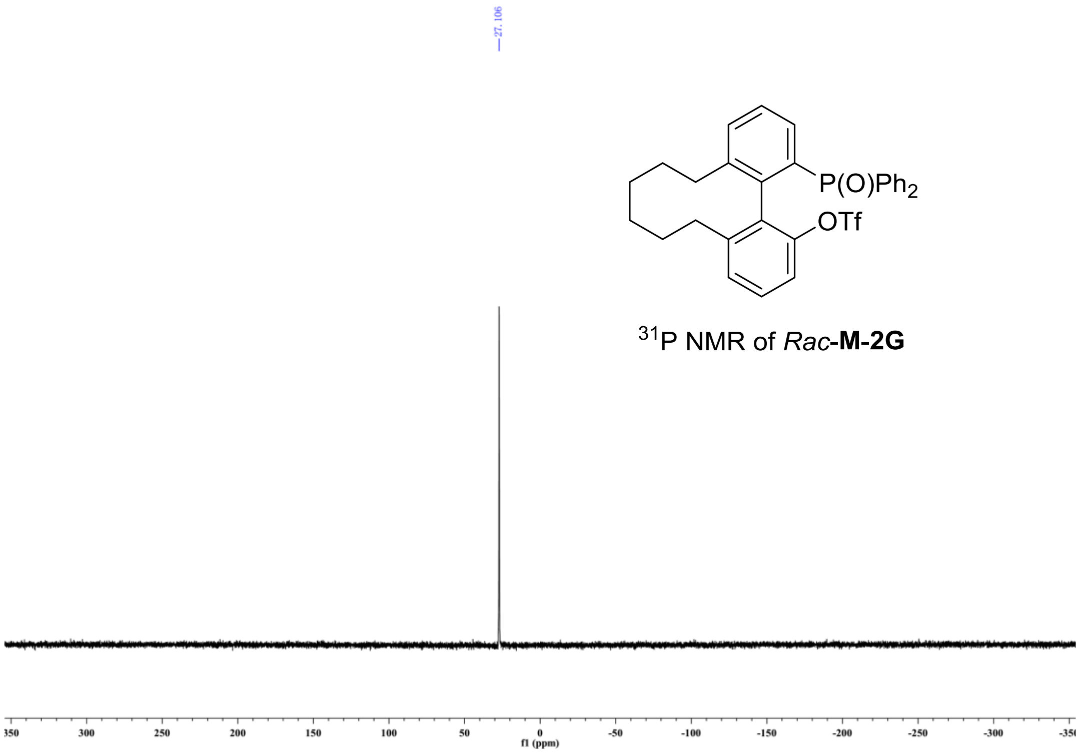

Figure S138. ^{31}P NMR of *Rac*-M-2G, related to Figure 2.

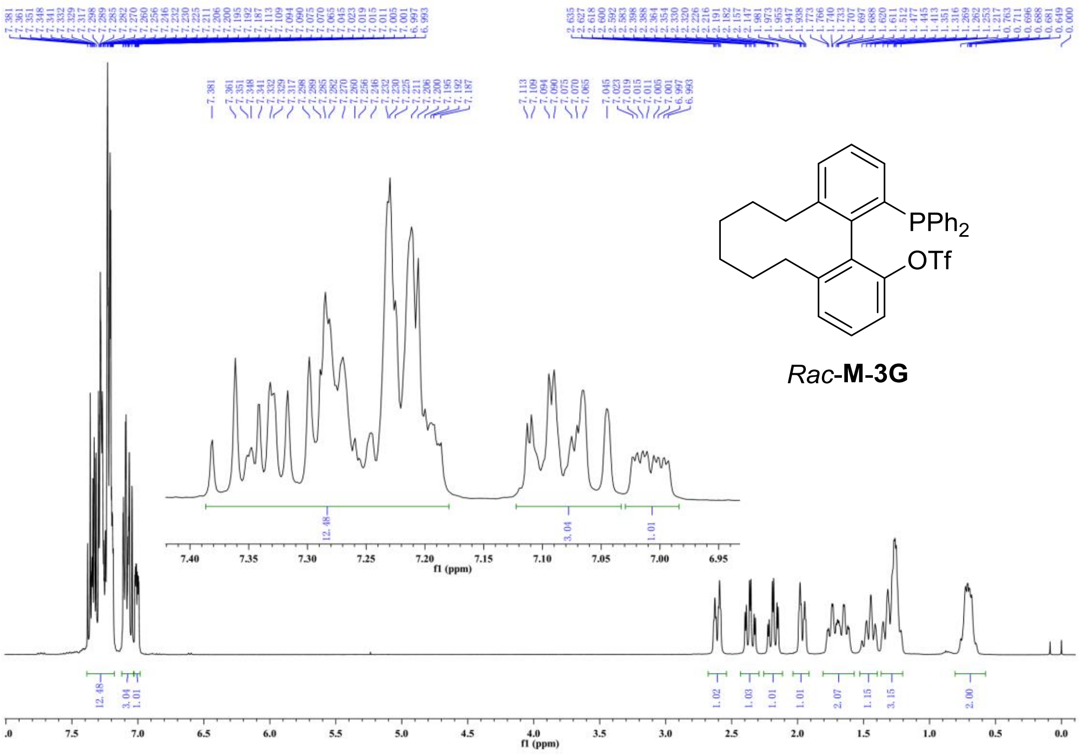

Figure S139. ^{1}H NMR of *Rac*-M-3G, related to Figure 2.

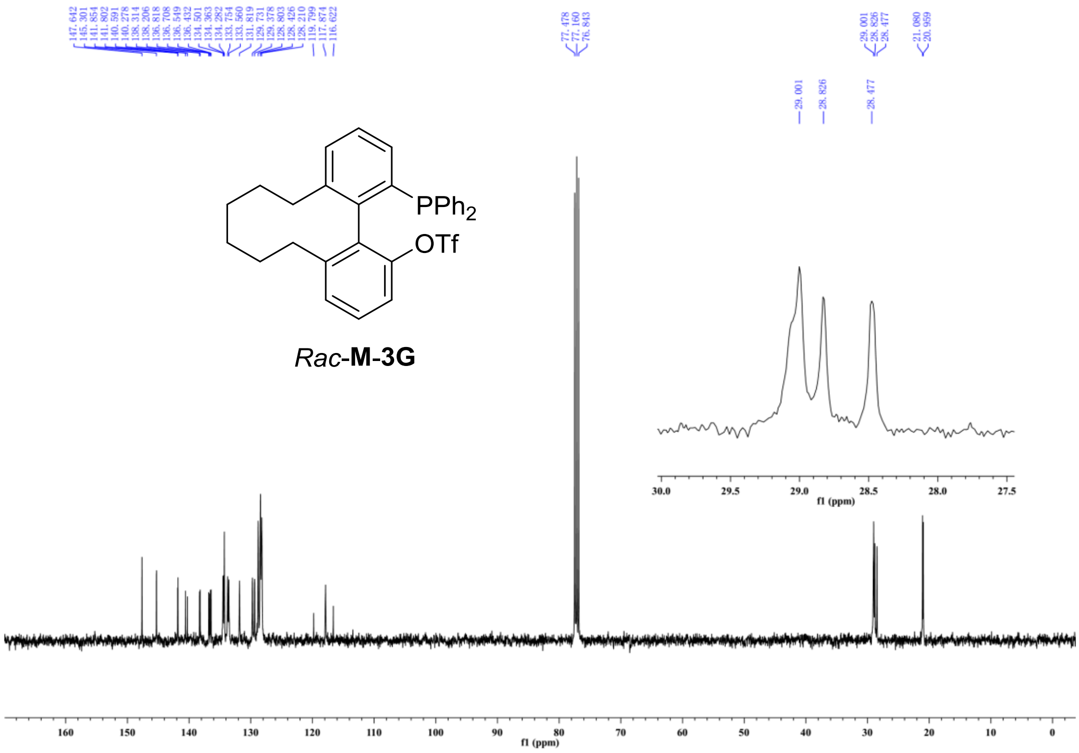

Figure S140. ^{13}C NMR of *Rac*-M-3G, related to Figure 2.

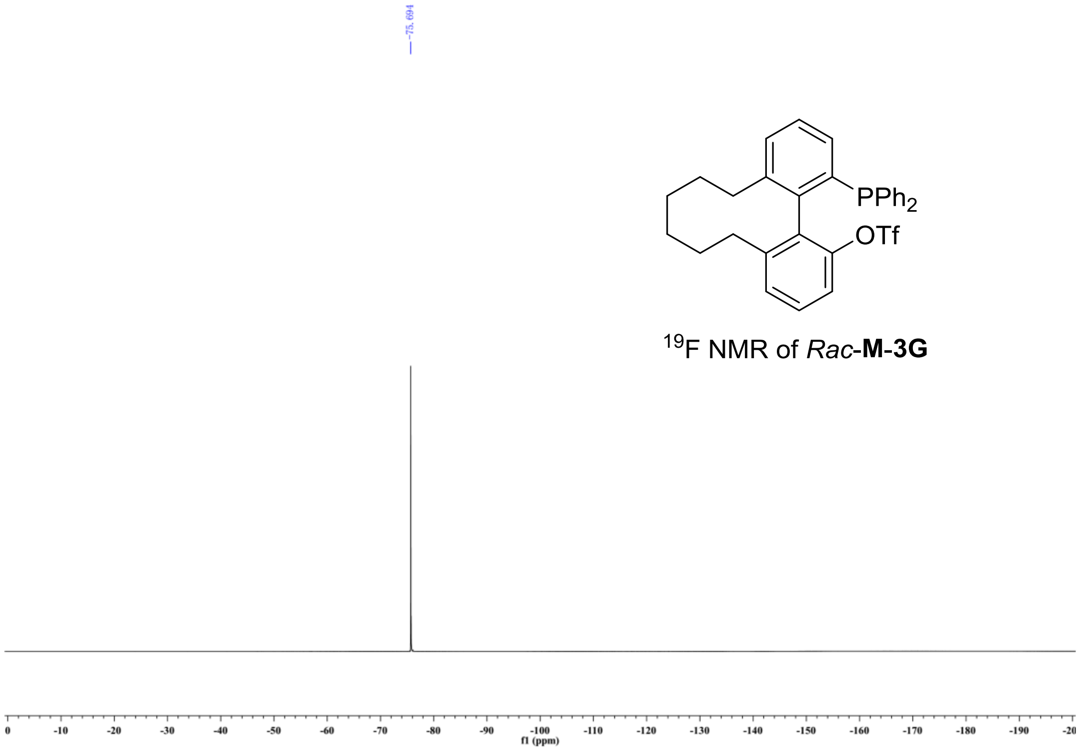

Figure S141. ^{19}F NMR of *Rac*-M-3G, related to Figure 2.

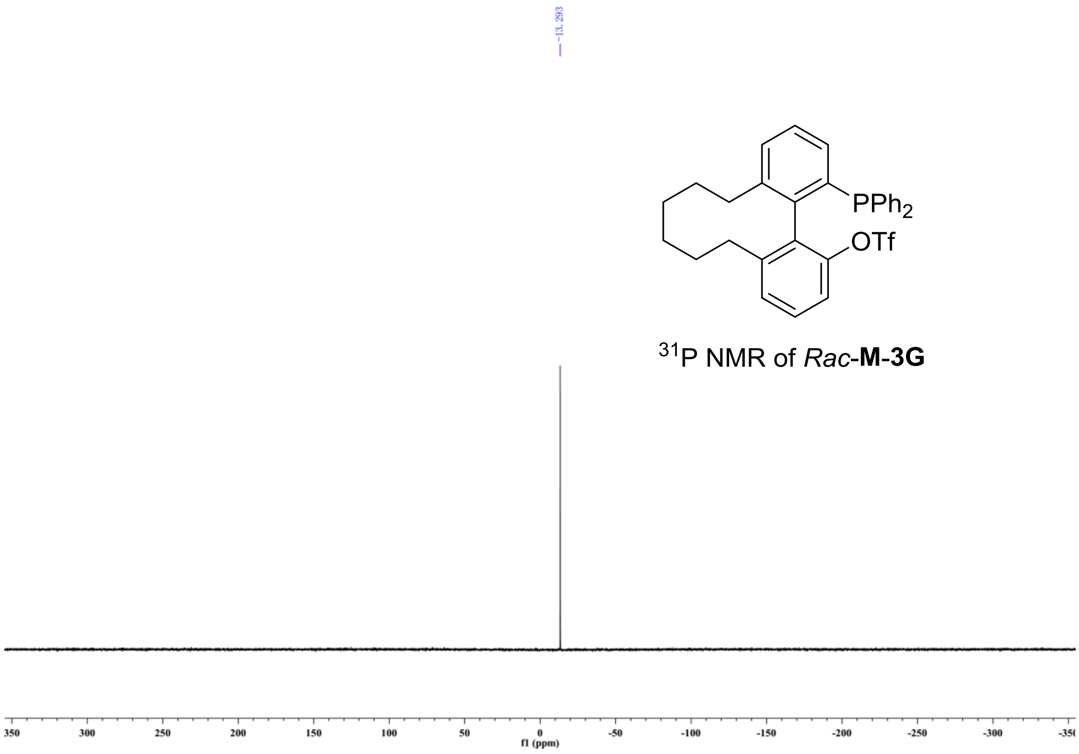

Figure S142. [31]H NMR of *Rac*-M-3G, related to Figure 2.

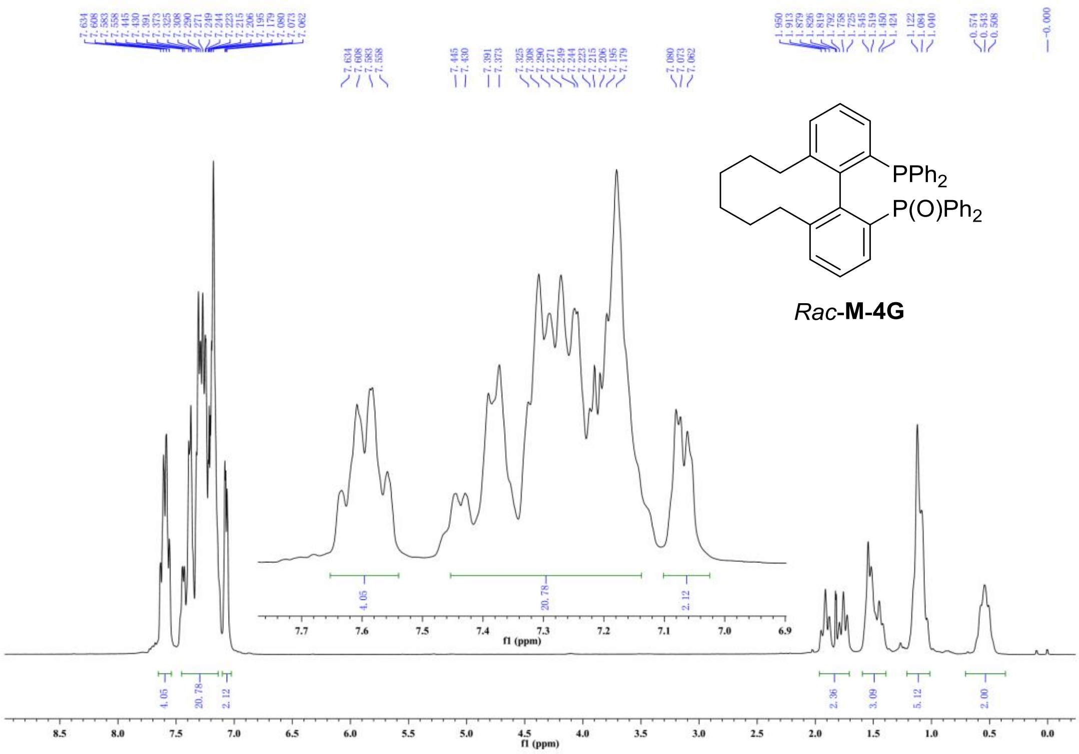

Figure S143. ^{1}H NMR of *Rac*-M-4G, related to Figure 2.

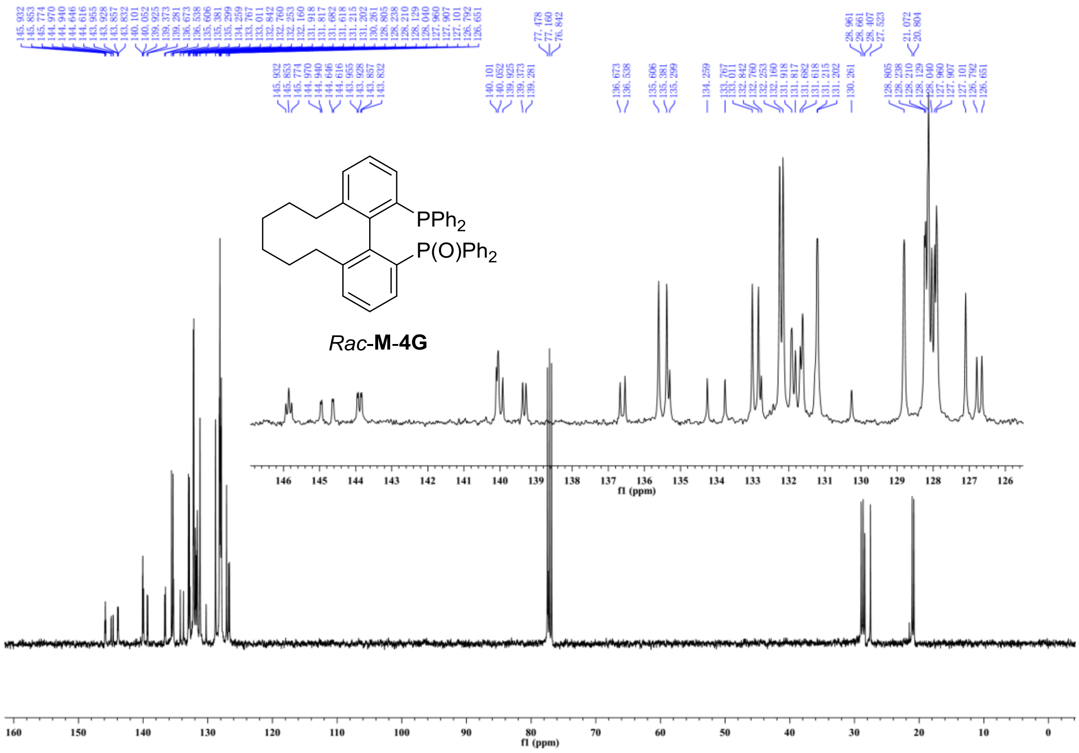

Figure S144. ^{13}**C NMR of** ***Rac*****-M-4G, related to Figure 2.**

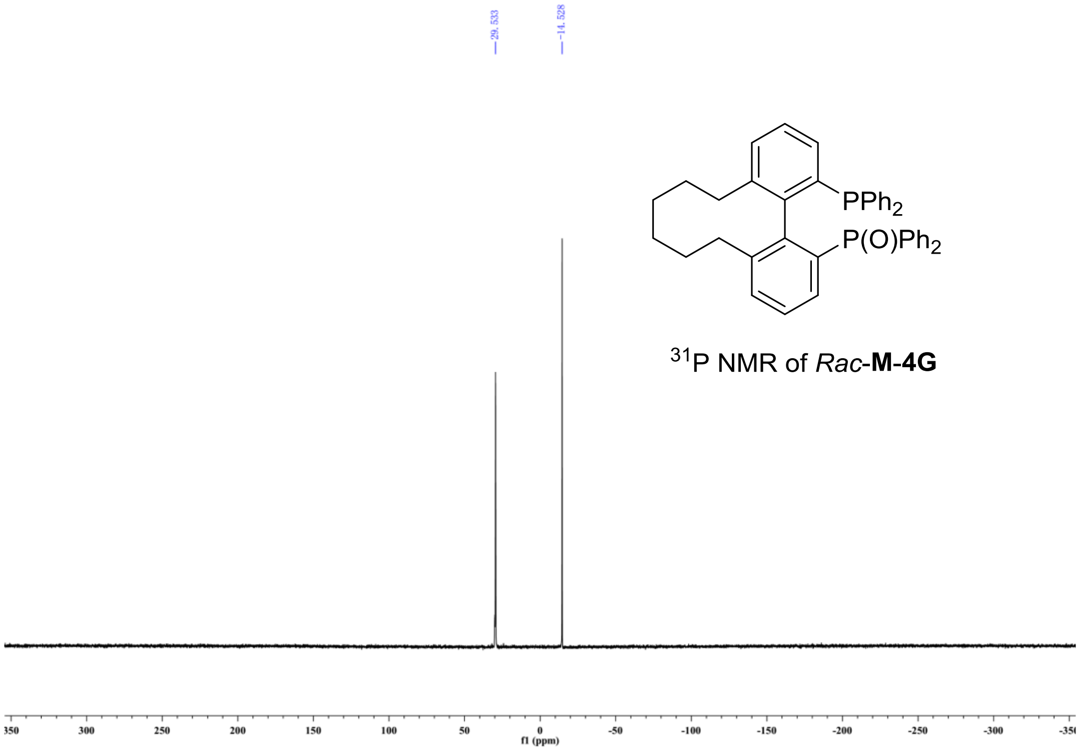

Figure S145. ^{31}**P NMR of** ***Rac*****-M-4G, related to Figure 2.**

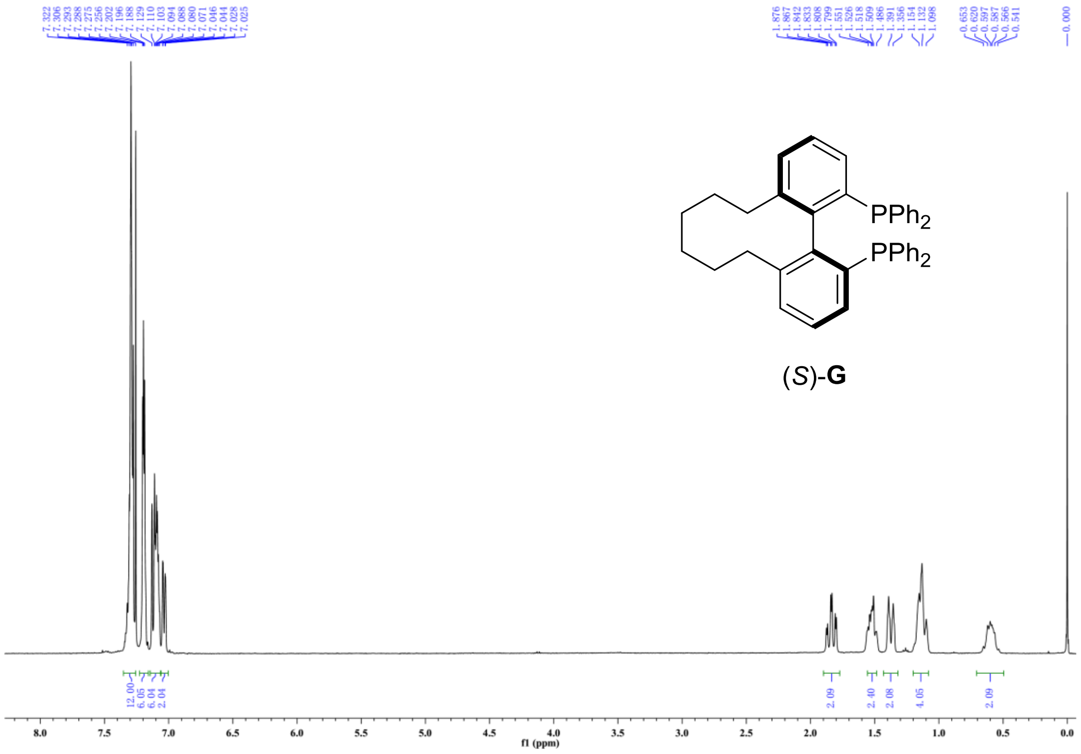

Figure S146. ^{1}H NMR of (*S*)-G, related to Figure 2.

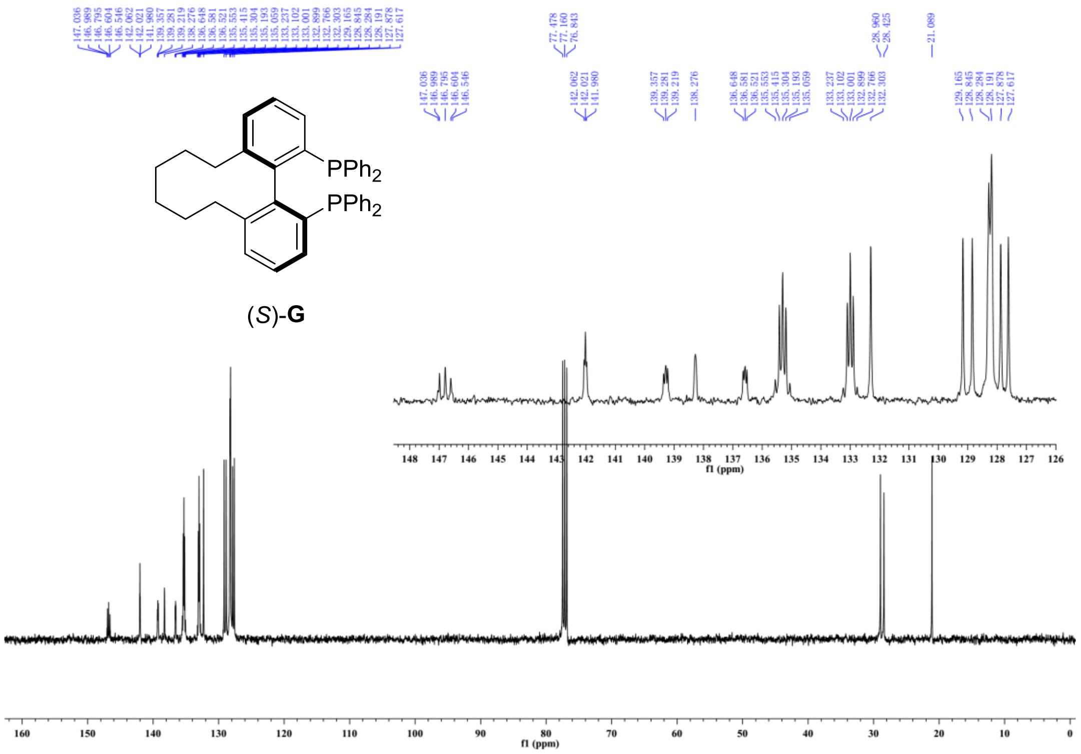

Figure S147. ^{13}C NMR of (*S*)-G, related to Figure 2.

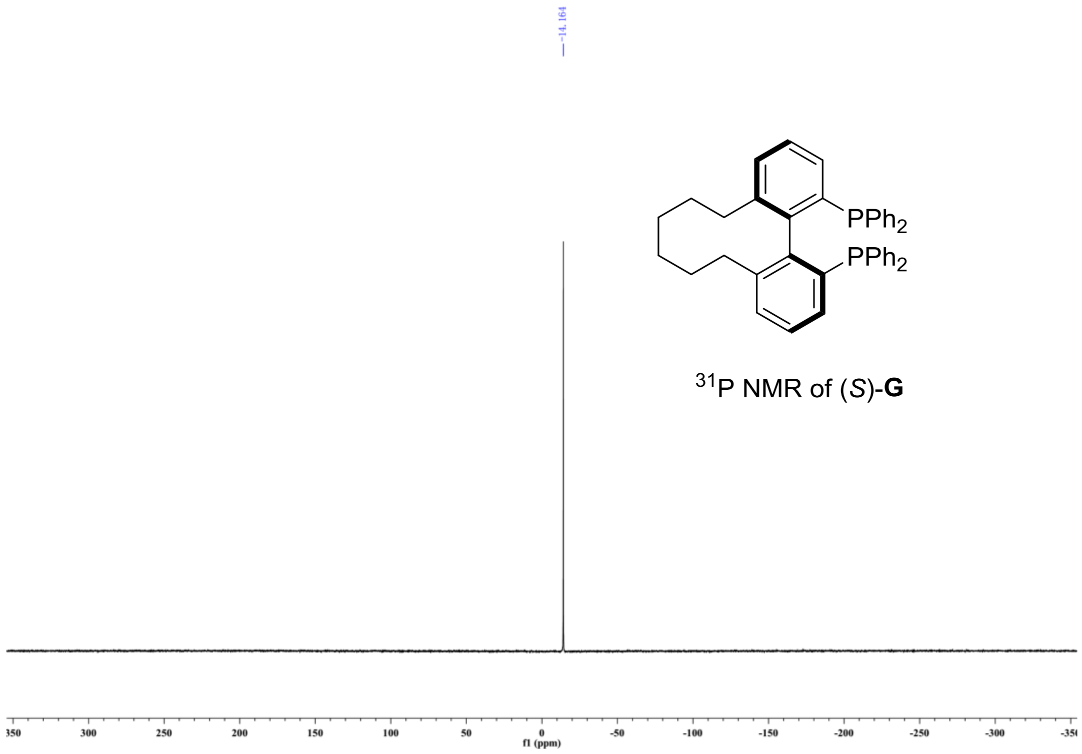

Figure S148. ^{31}P NMR of (*S*)-G, related to Figure 2.

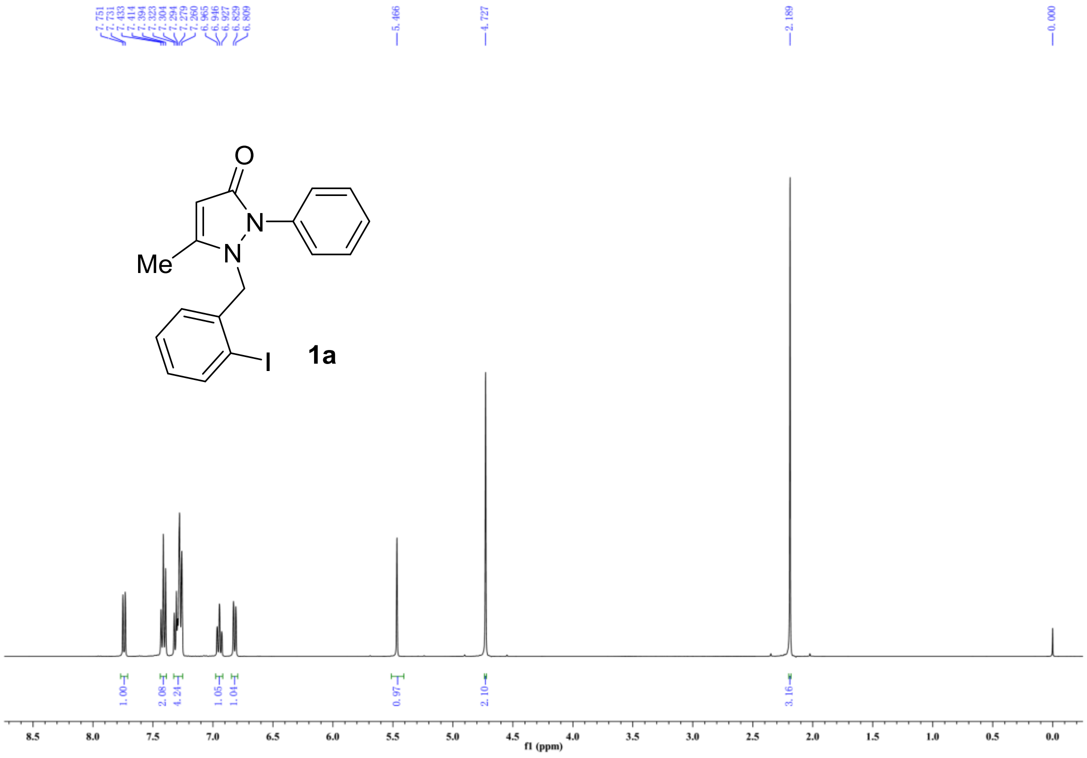

Figure S149. ^{1}H NMR of 1a, related to Table 2.

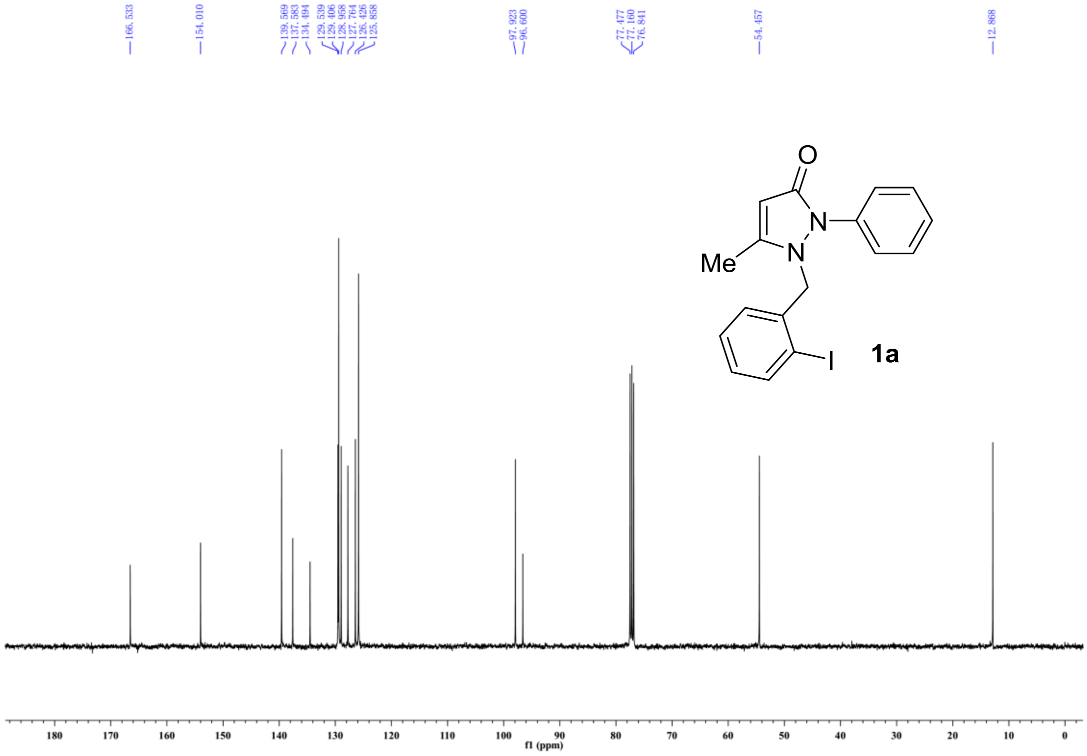

Figure S150. **^{13}C NMR of 1a, related to Table 2.**

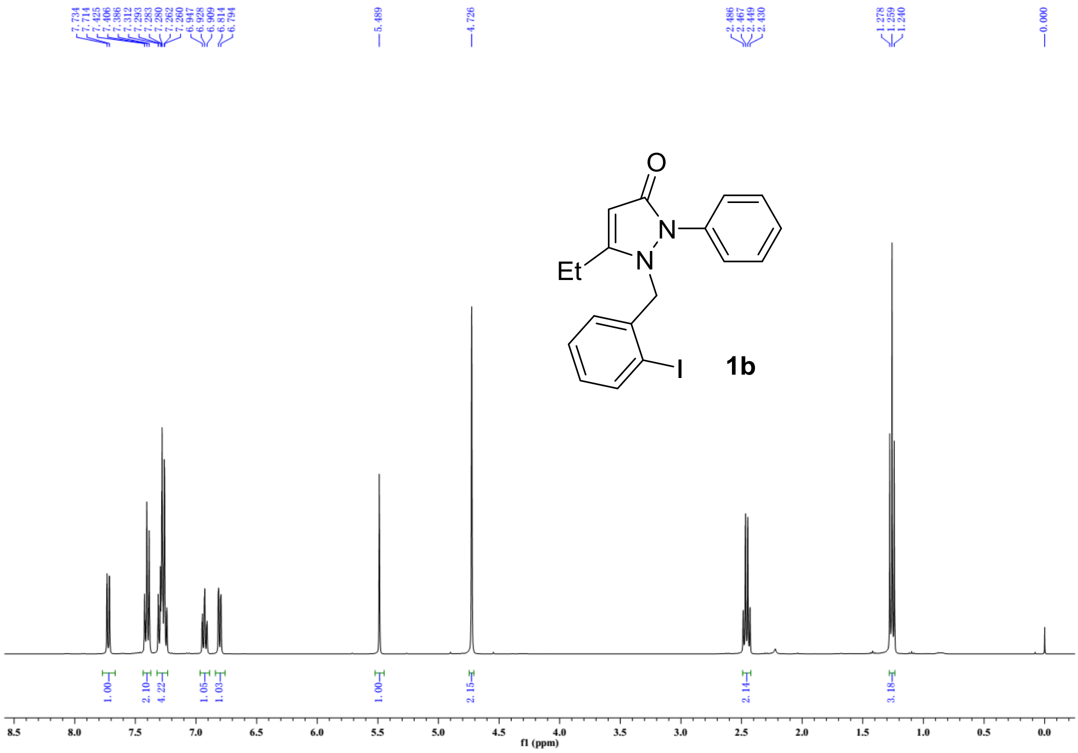

Figure S151. **^{13}C NMR of 1b, related to Table 2.**

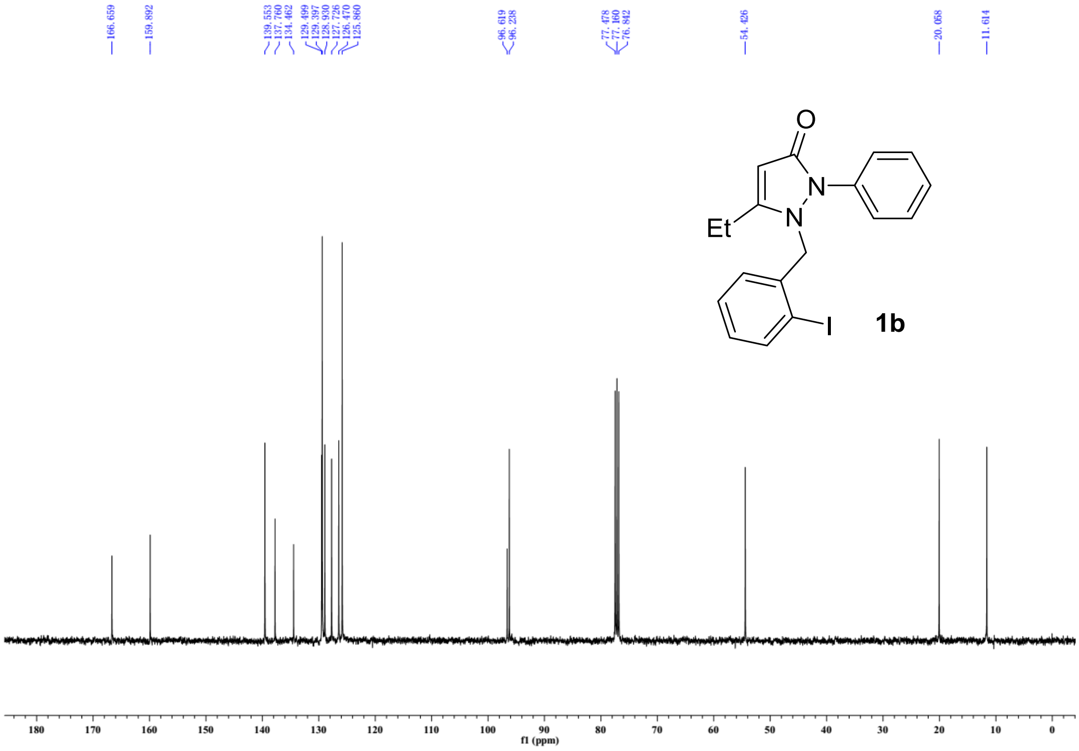

Figure S152. ^{13}C NMR of 1b, related to Table 2.

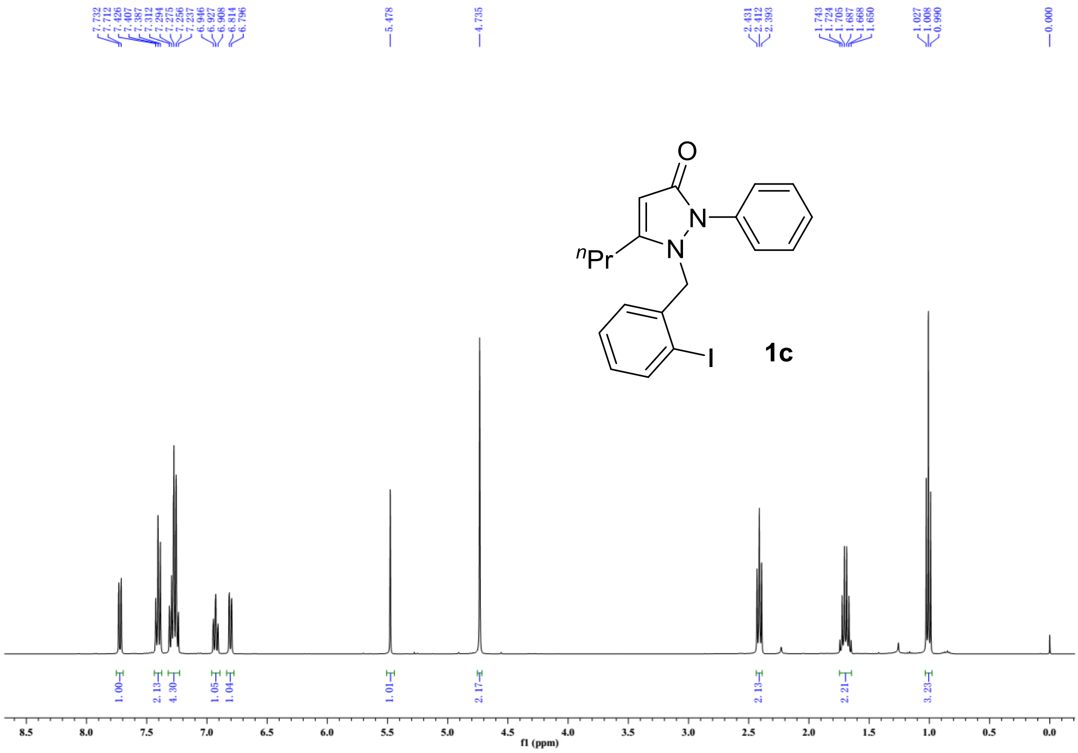

Figure S153. ^{1}H NMR of 1c, related to Table 2.

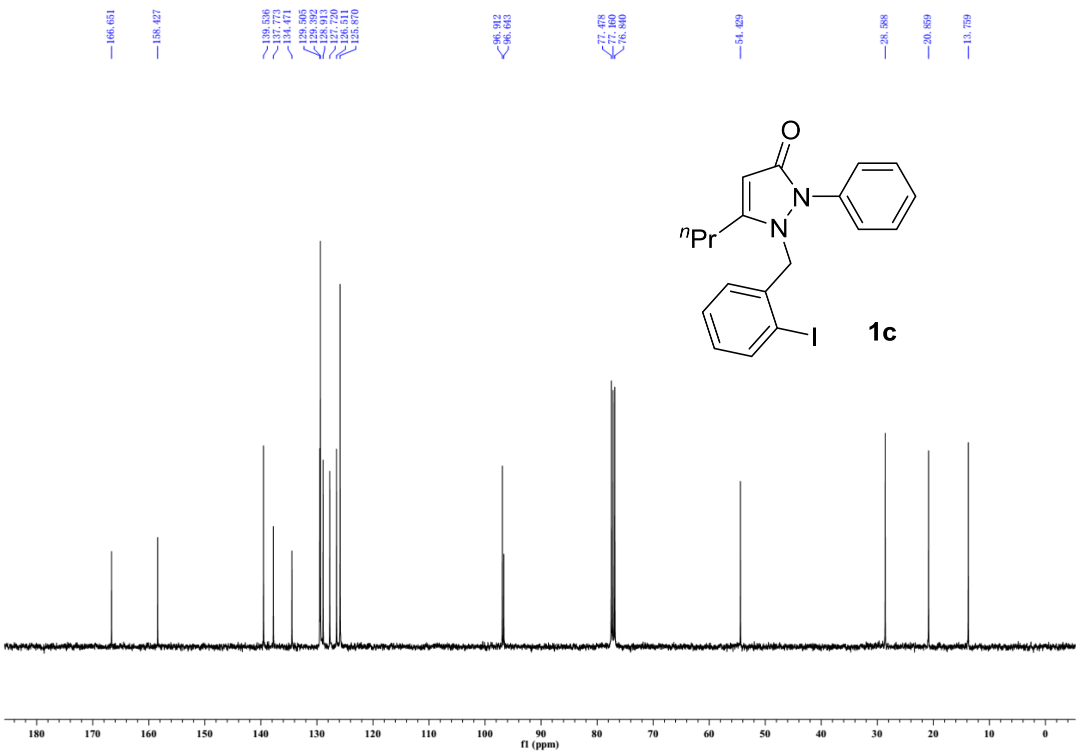

Figure S154. ^{13}**C NMR of 1c, related to Table 2.**

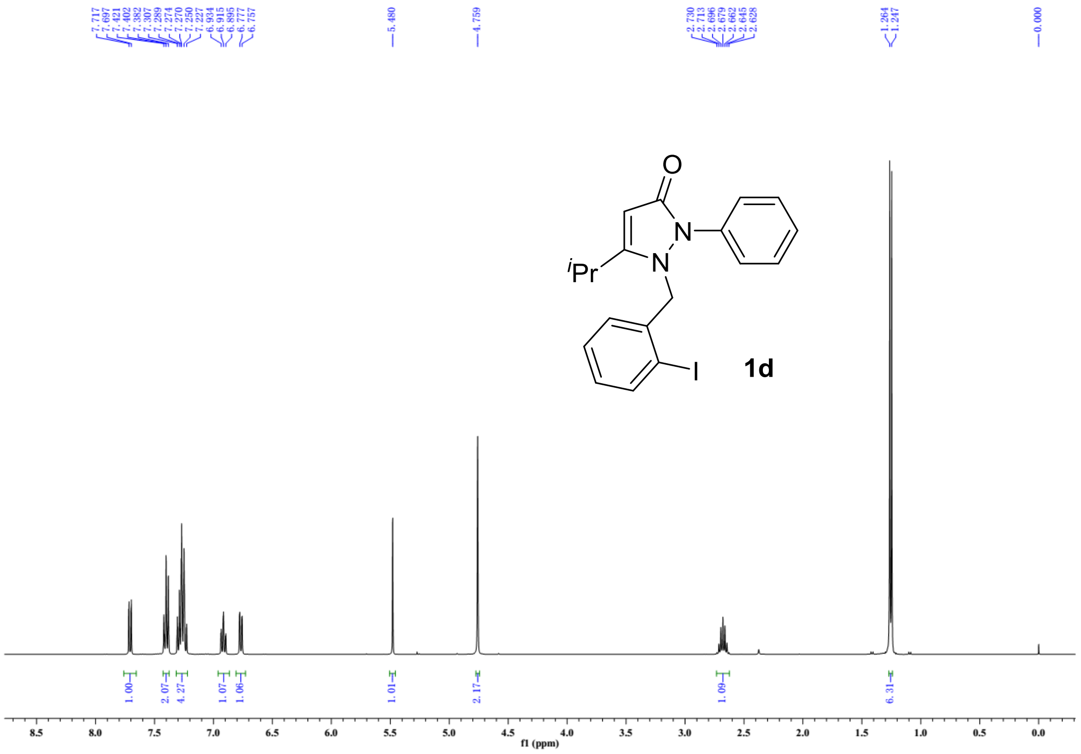

Figure S155. ^{1}**H NMR of 1d, related to Table 2.**

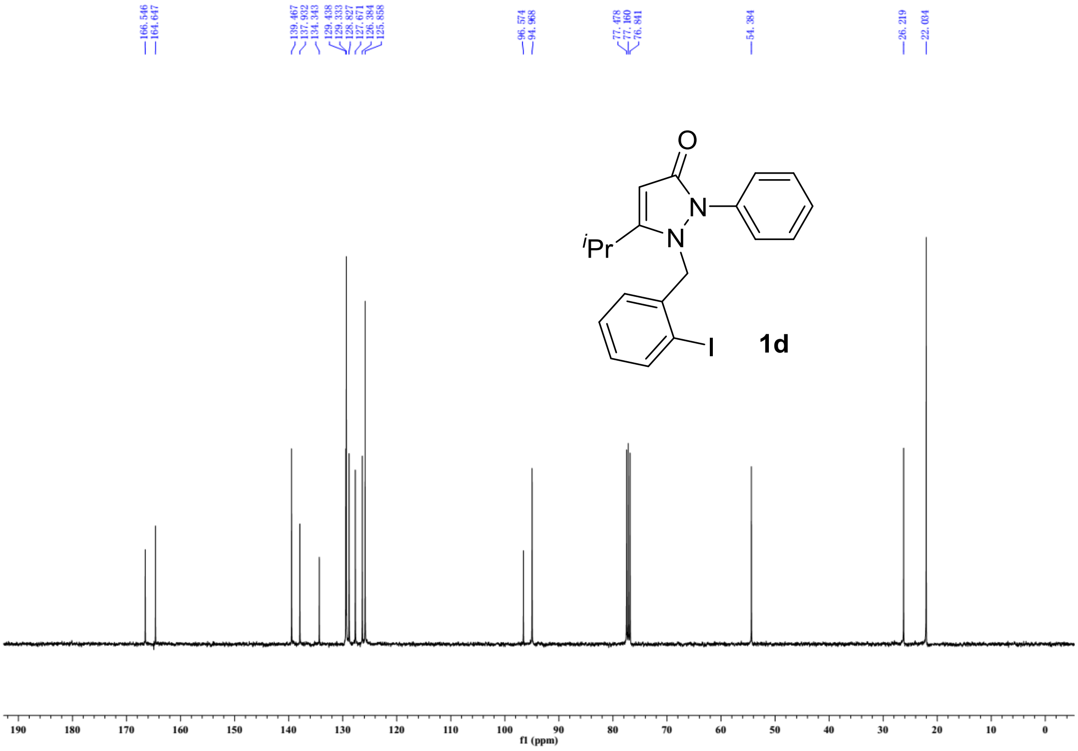

Figure S156. ^{13}C **NMR of 1d, related to Table 2.**

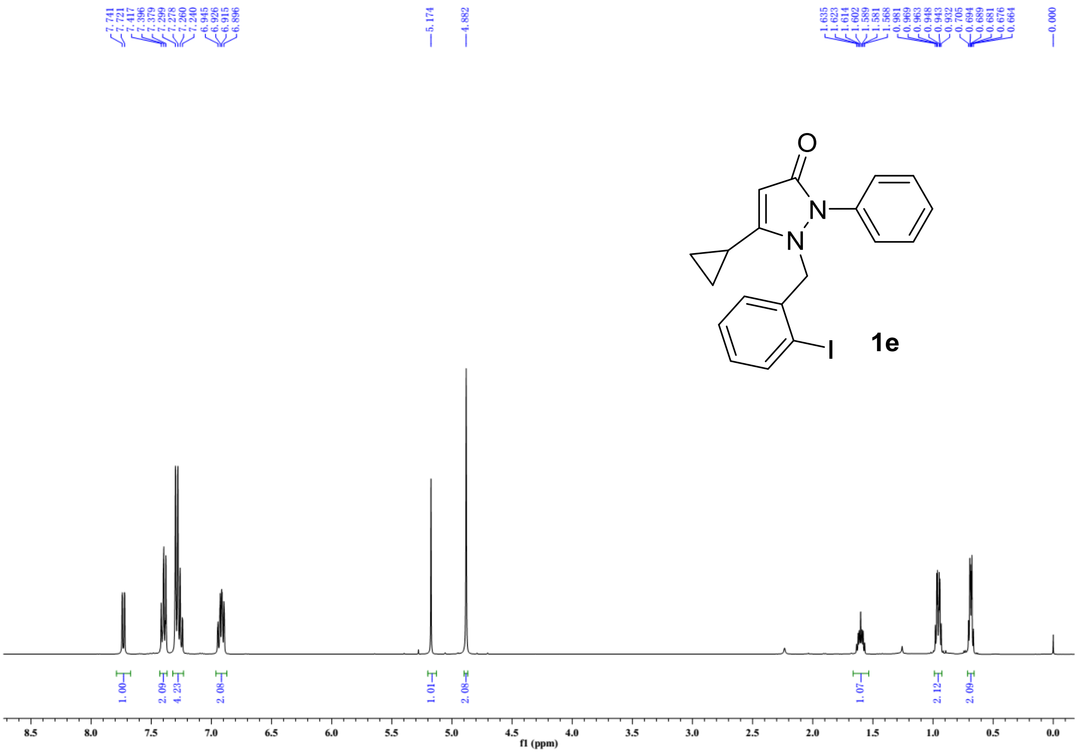

Figure S157. ^{1}H **NMR of 1e, related to Table 2.**

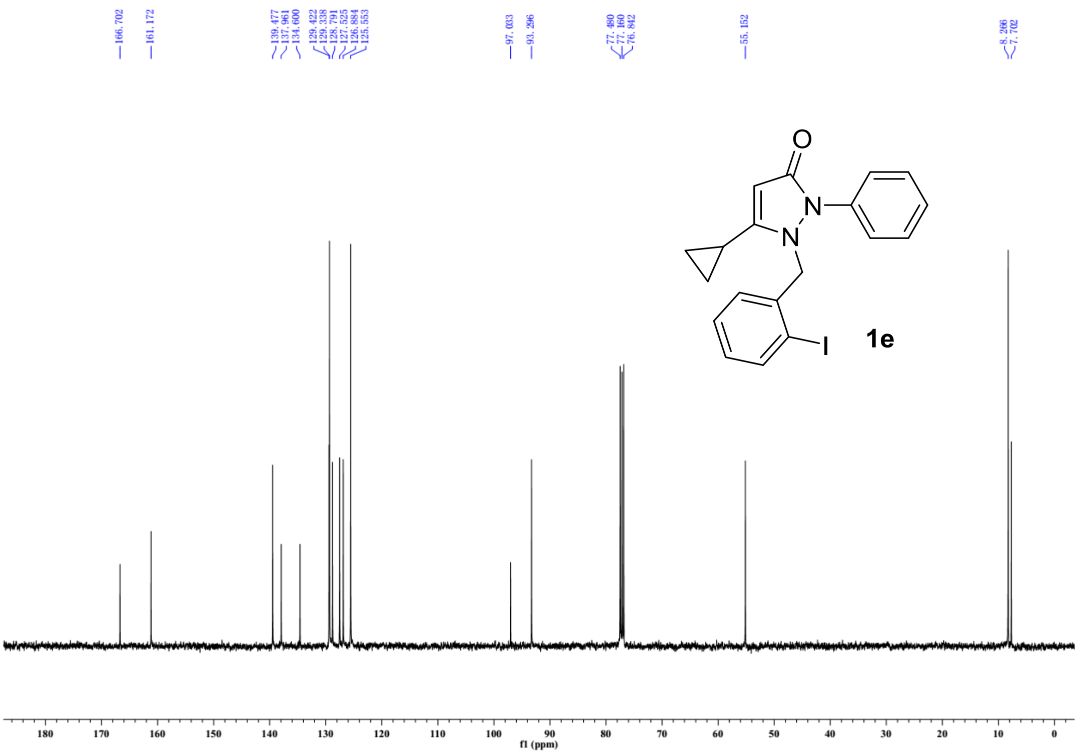

Figure S158. ^{13}**C NMR of 1e, related to Table 2.**

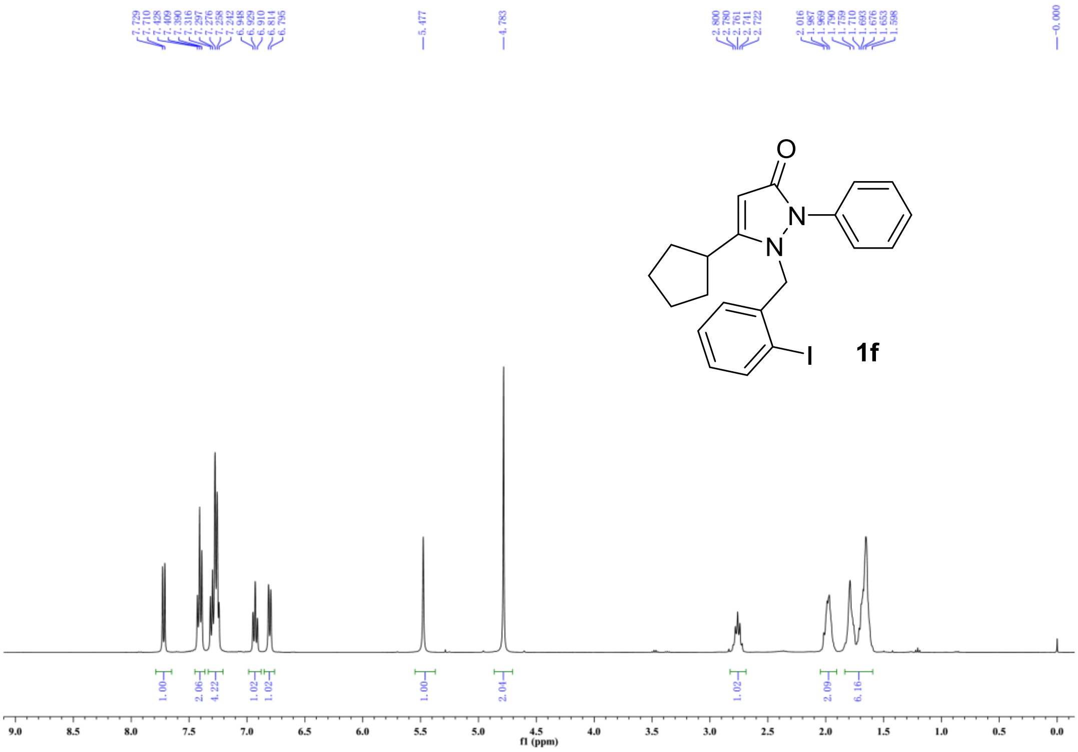

Figure S159. ^{1}**H NMR of 1f, related to Table 2.**

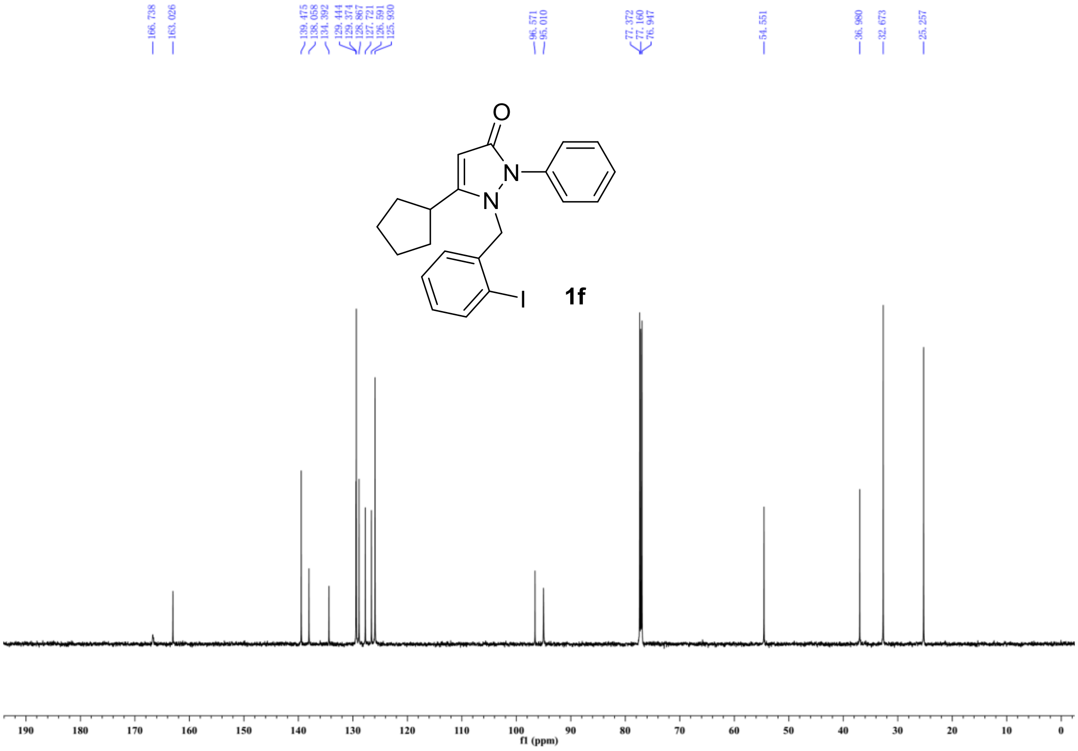

Figure S160. ^{13}C NMR of 1f, related to Table 2.

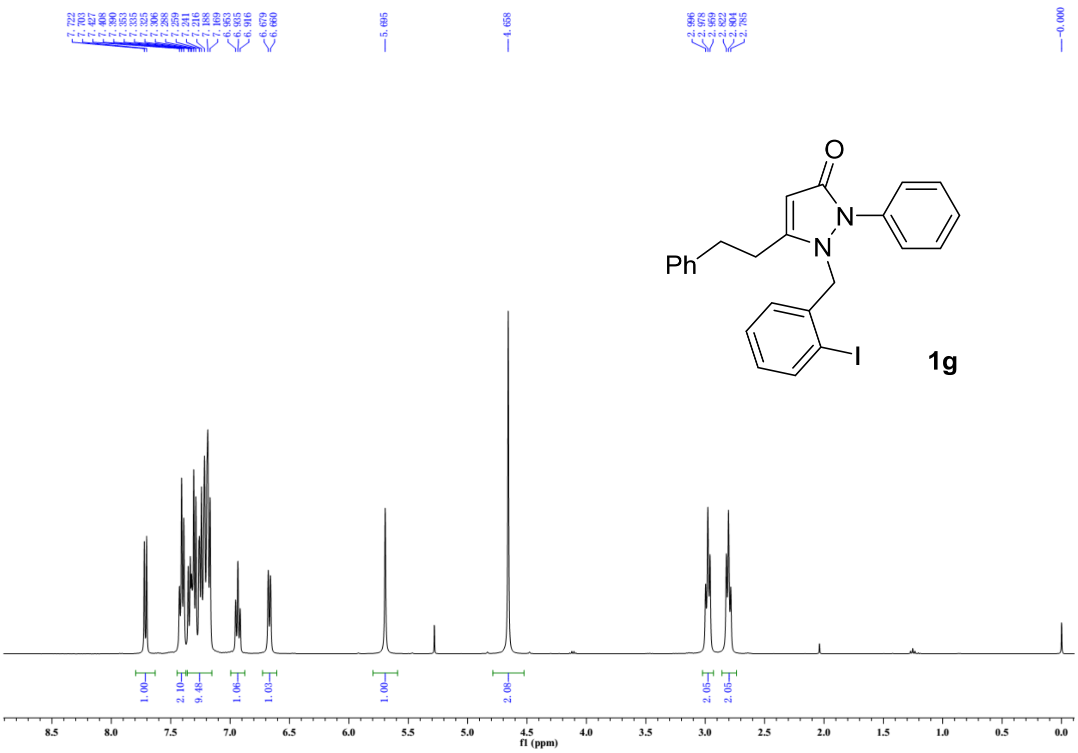

Figure S161. ^{1}H NMR of 1g, related to Table 2.

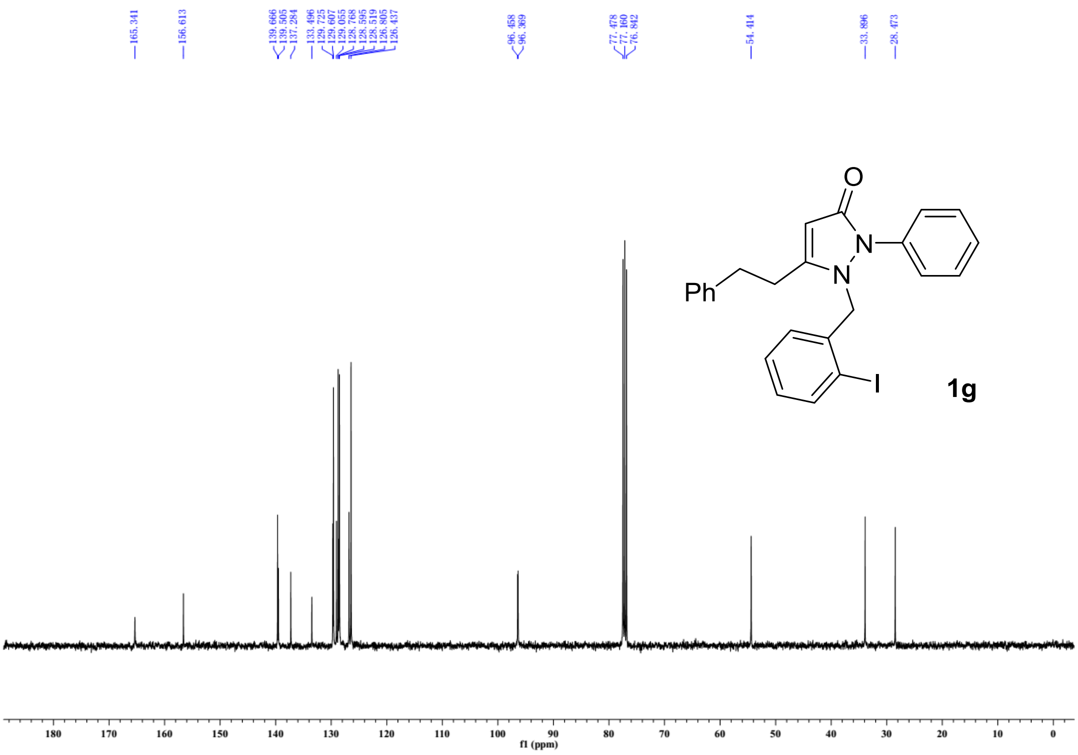

Figure S162. ^{13}C NMR of 1g, related to Table 2.

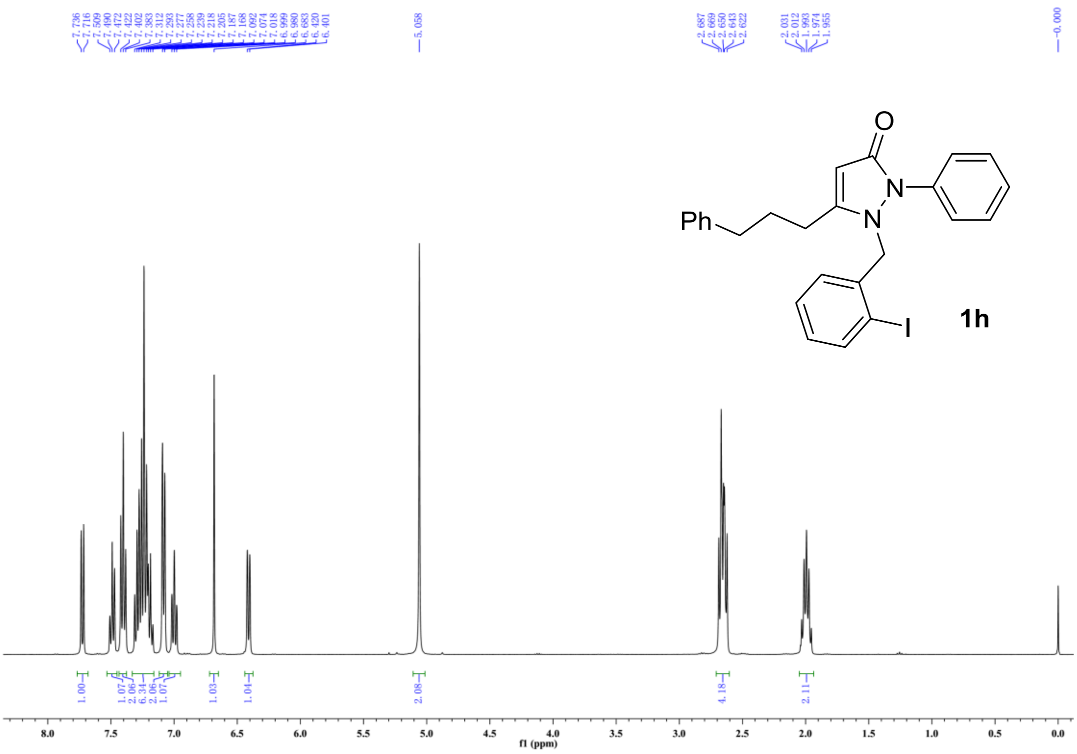

Figure S163. ^{1}H NMR of 1h, related to Table 2.

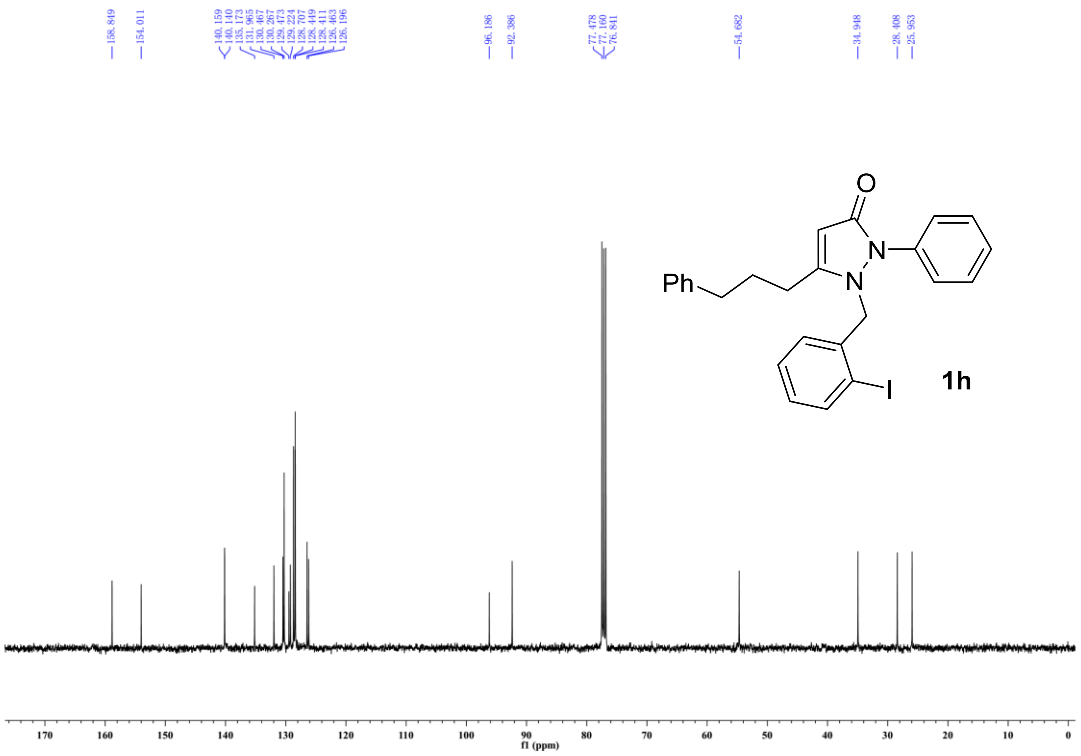

Figure S164. ^{13}C NMR of 1h, related to Table 2.

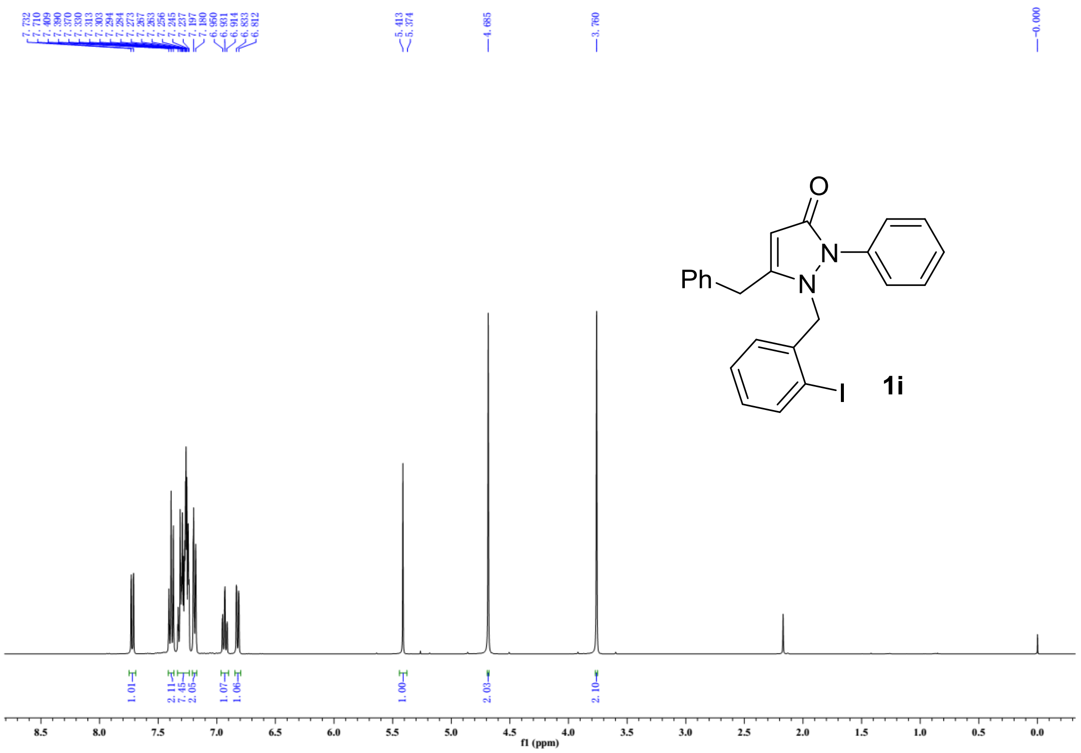

Figure S165. ^{1}H NMR of 1i, related to Table 2.

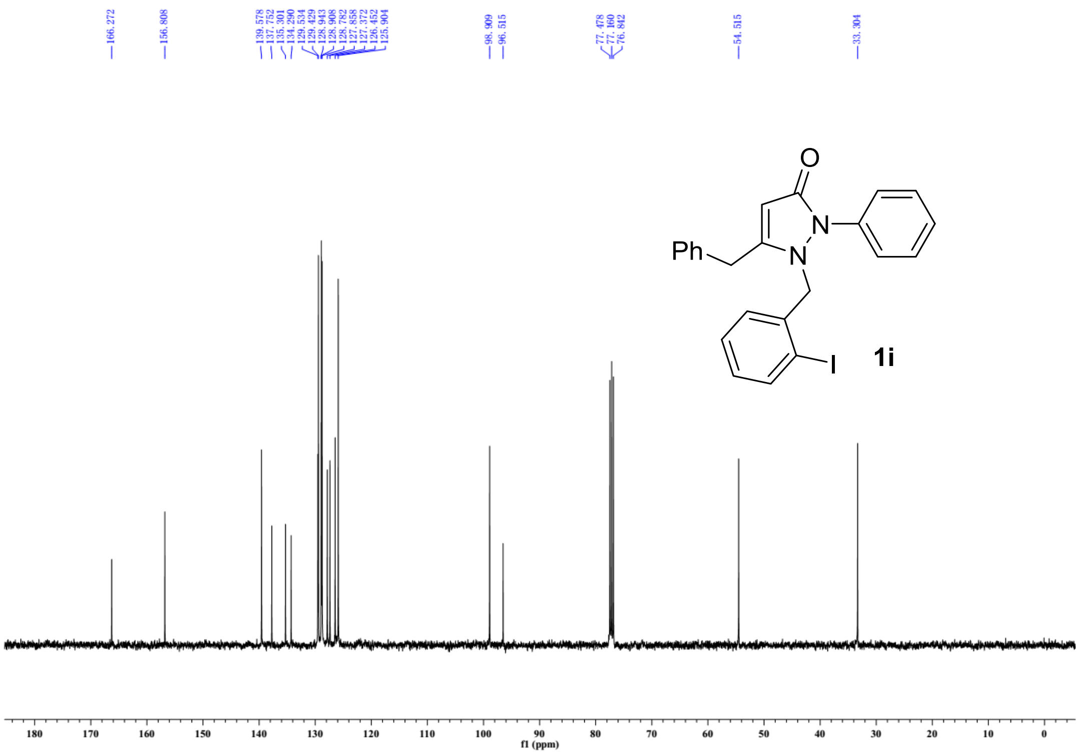

Figure S166. ^{13}C NMR of 1i, related to Table 2.

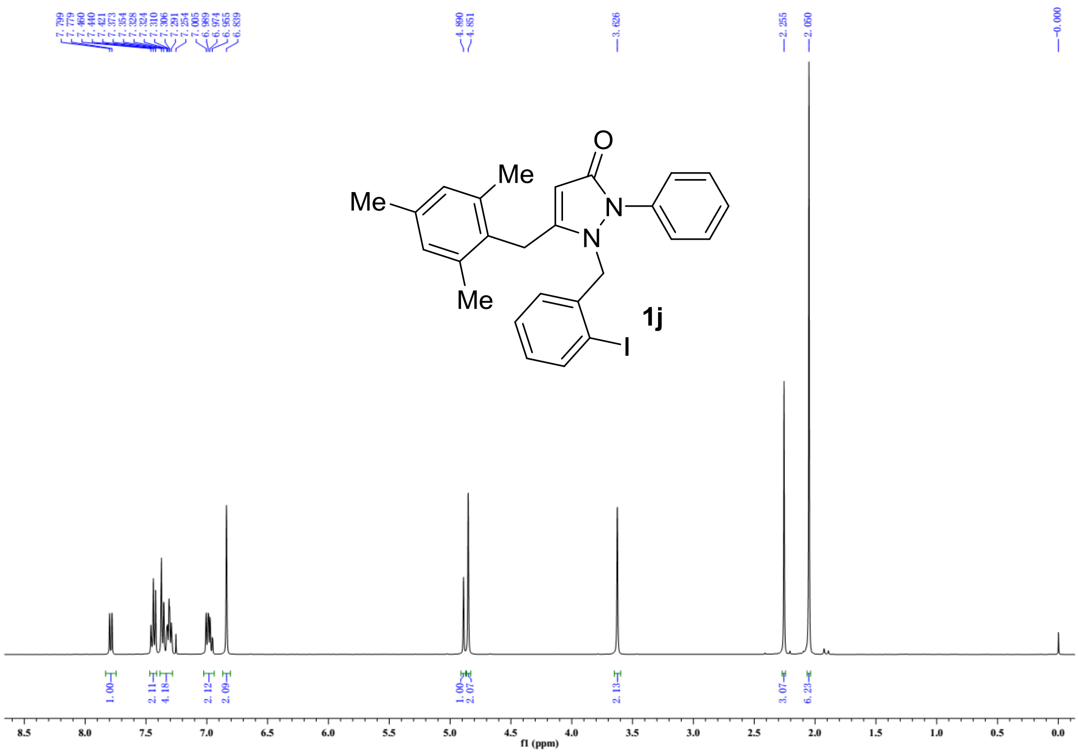

Figure S167. ^{1}H NMR of 1j, related to Table 2.

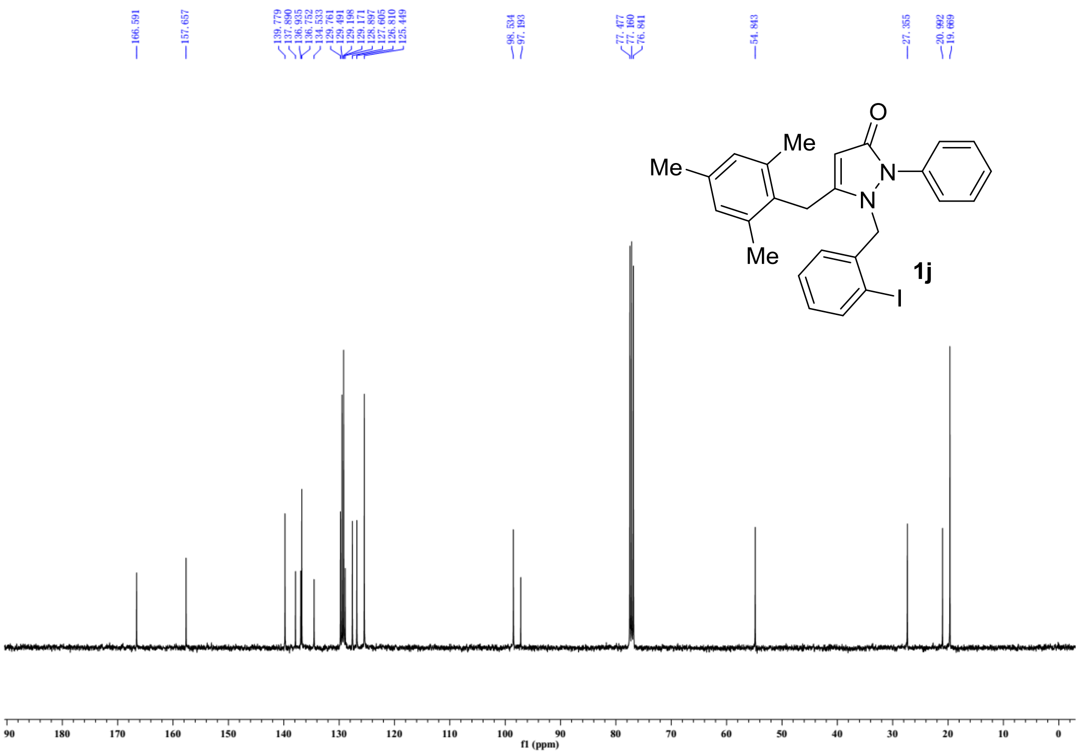

Figure S168. ^{13}C NMR of 1j, related to Table 2.

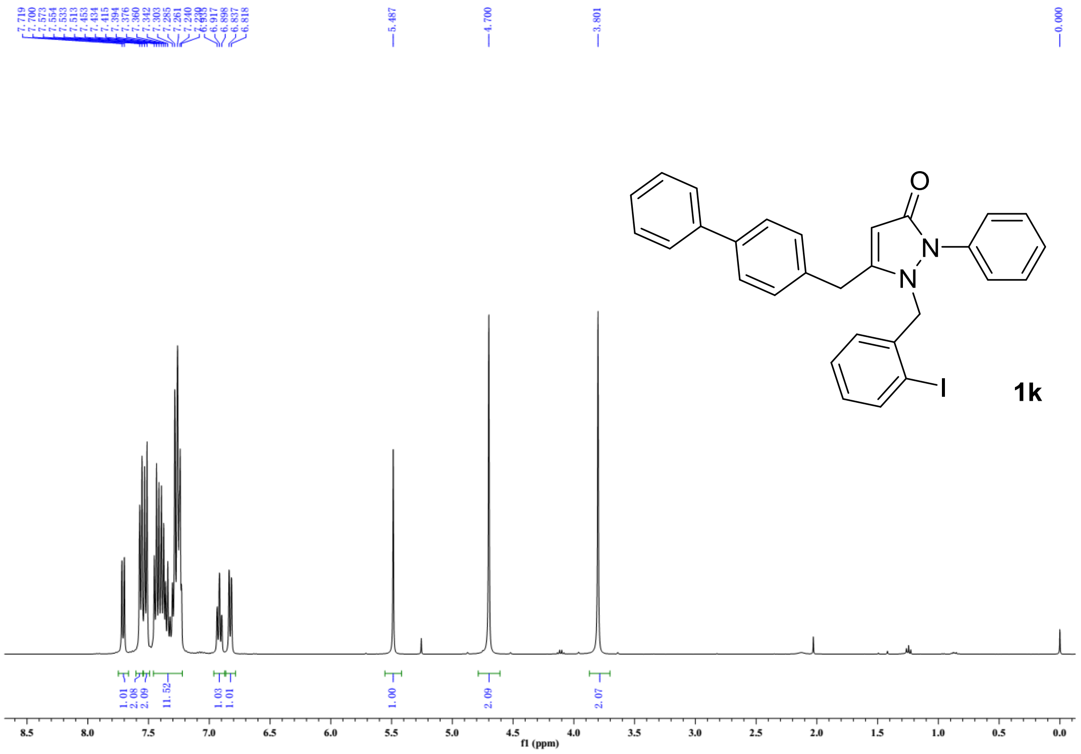

Figure S169. ^{1}H NMR of 1k, related to Table 2.

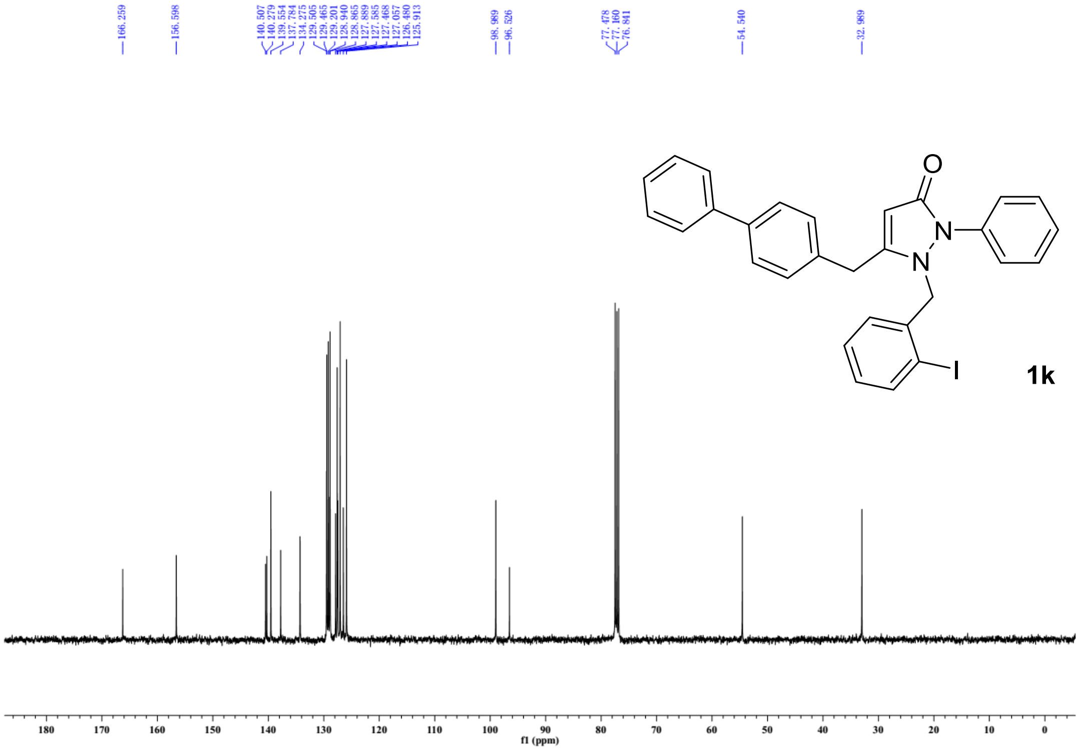

Figure S170. ^{13}C **NMR of 1k, related to Table 2.**

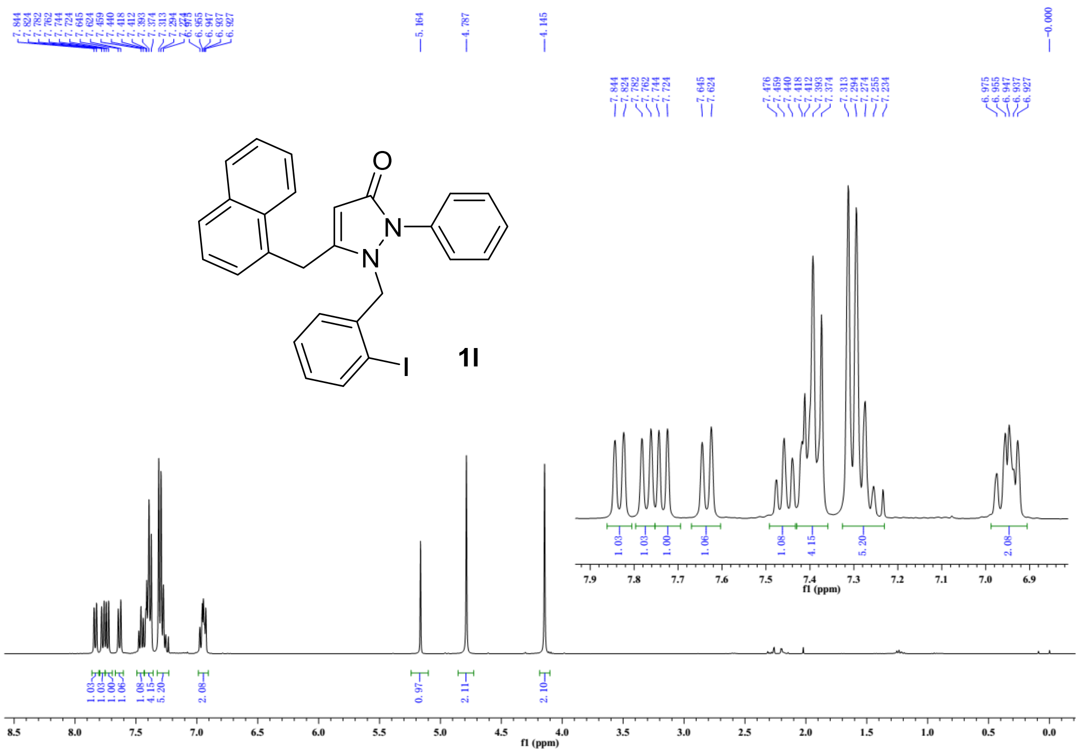

Figure S171. ^{1}H **NMR of 1l, related to Table 2.**

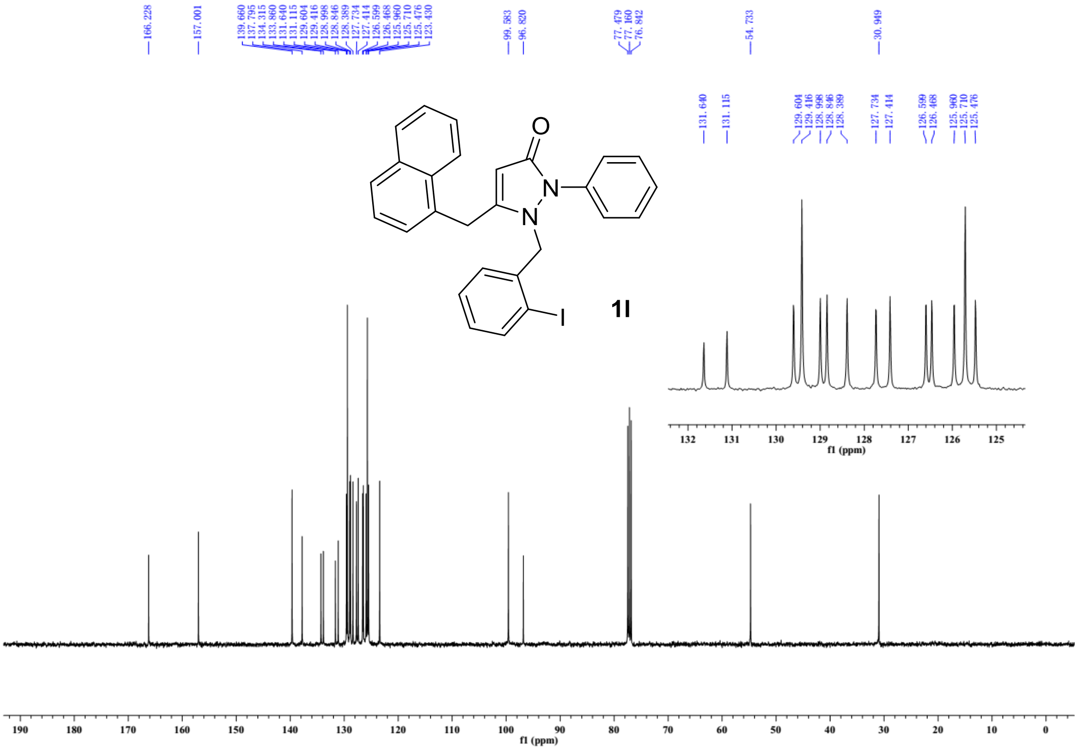

Figure S172. ^{13}C NMR of 1l, related to Table 2.

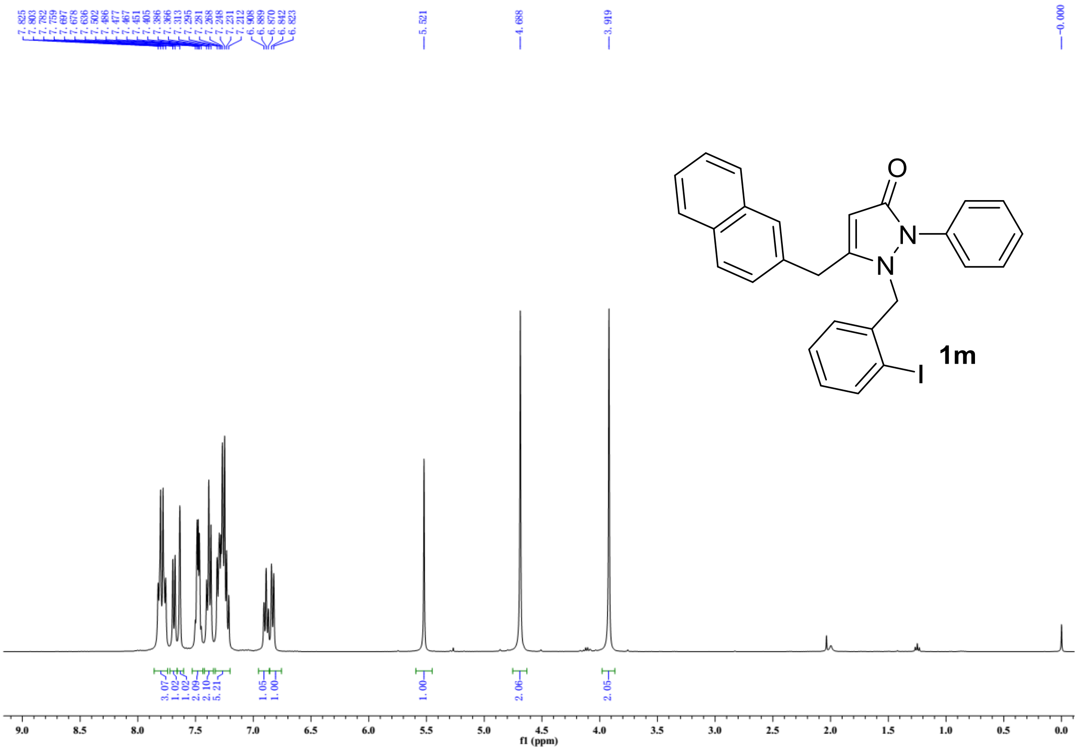

Figure S173. ^{1}H NMR of 1m, related to Table 2.

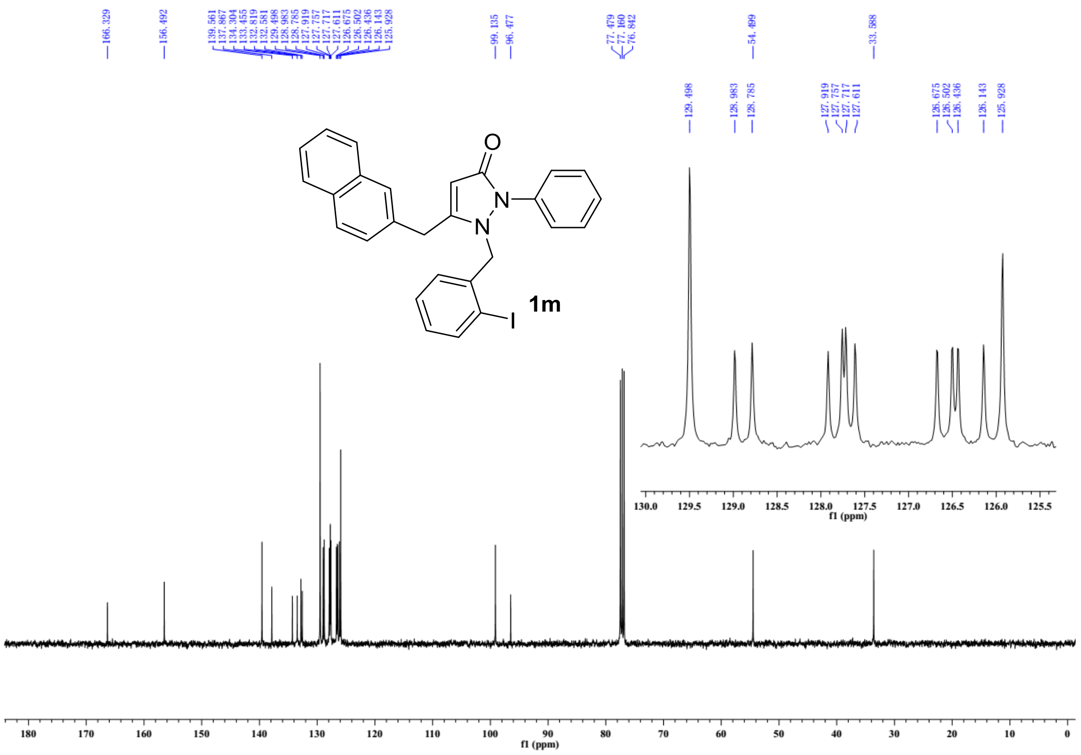

Figure S174. ^{13}C NMR of 1m, related to Table 2.

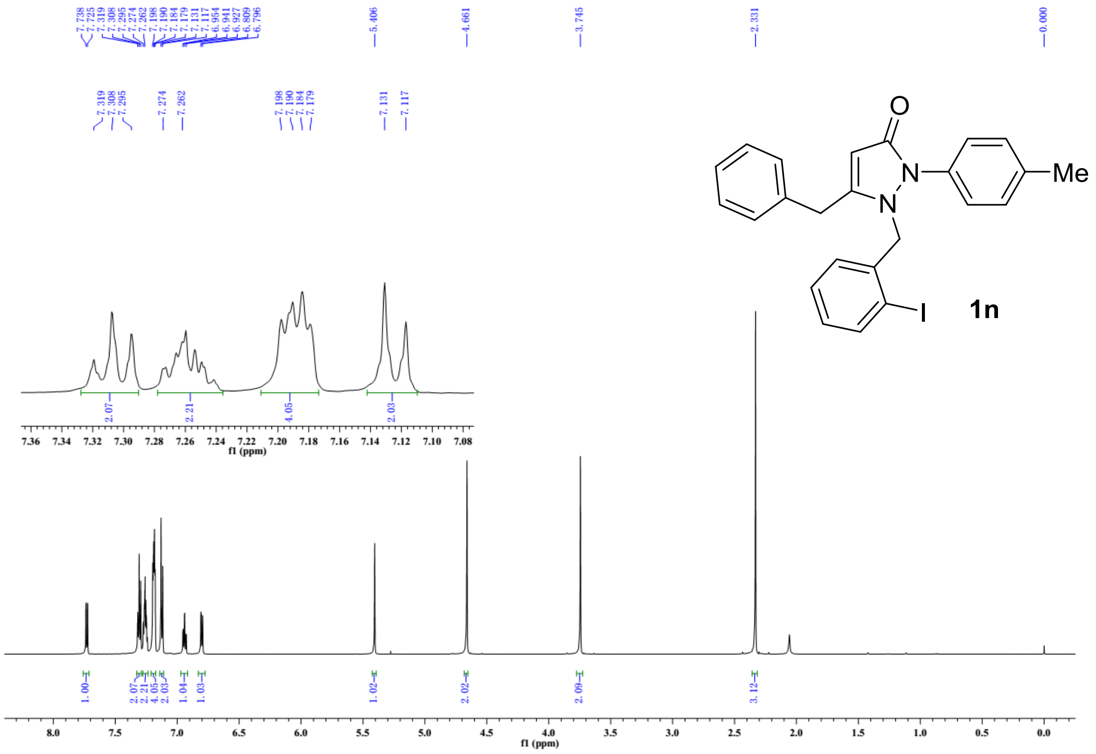

Figure S175. ^{1}H NMR of 1n, related to Table 2.

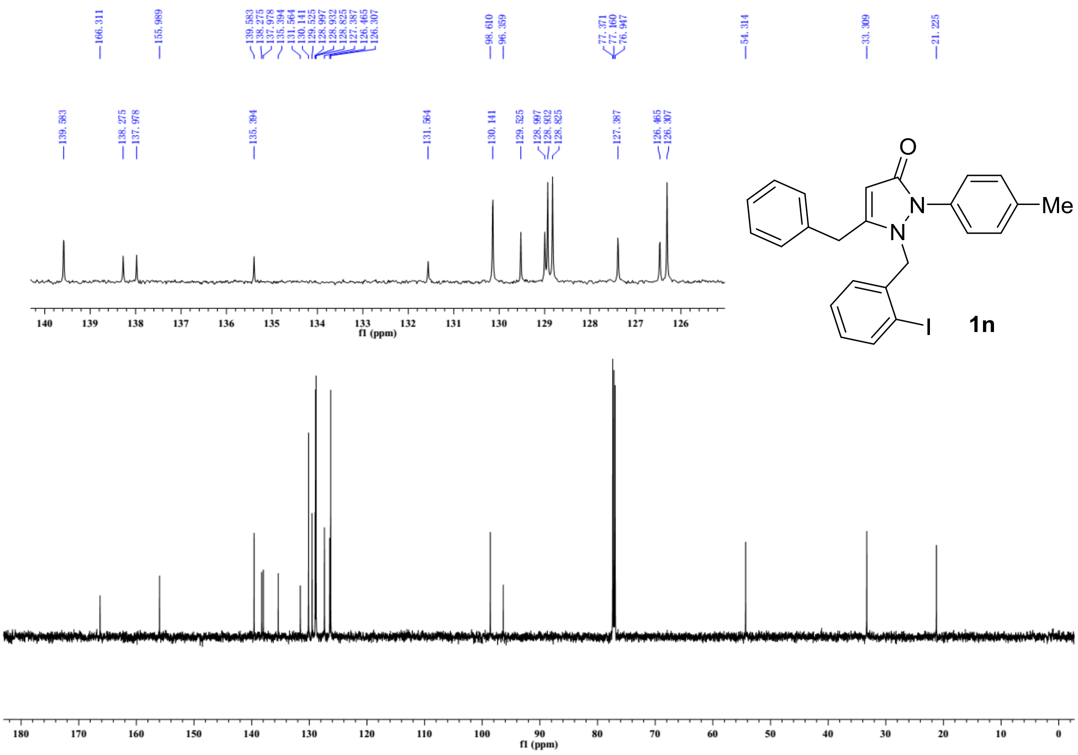

Figure S176. ^{13}C NMR of 1n, related to Table 2.

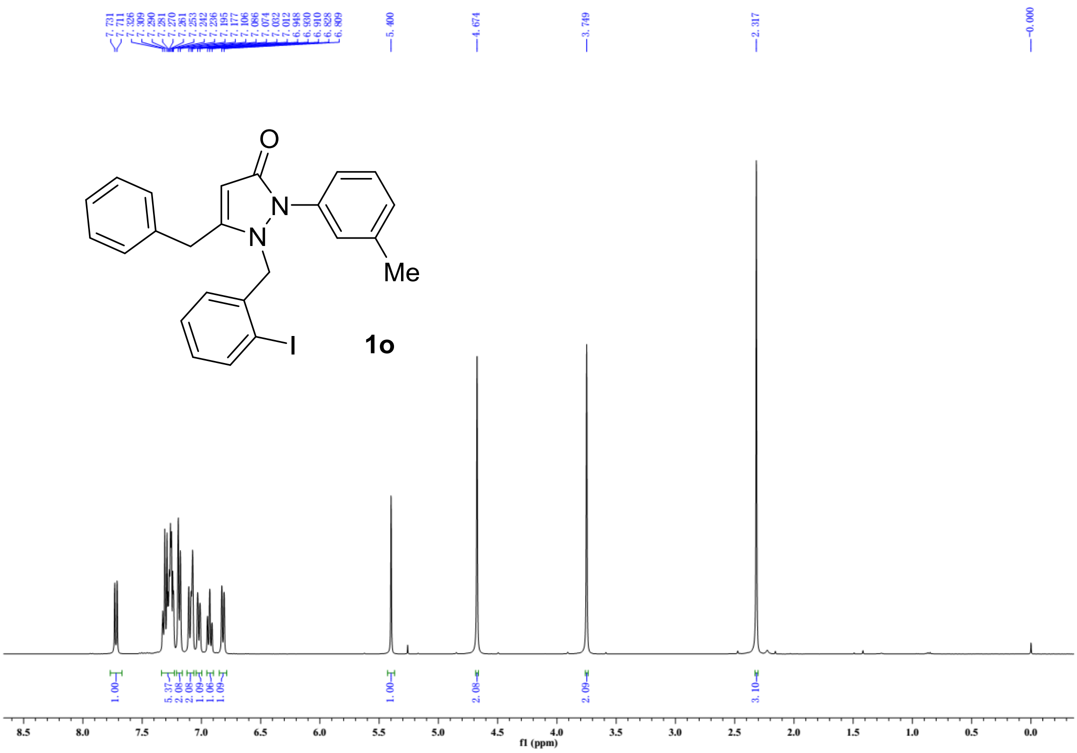

Figure S177. ^{1}H NMR of 1o, related to Table 2.

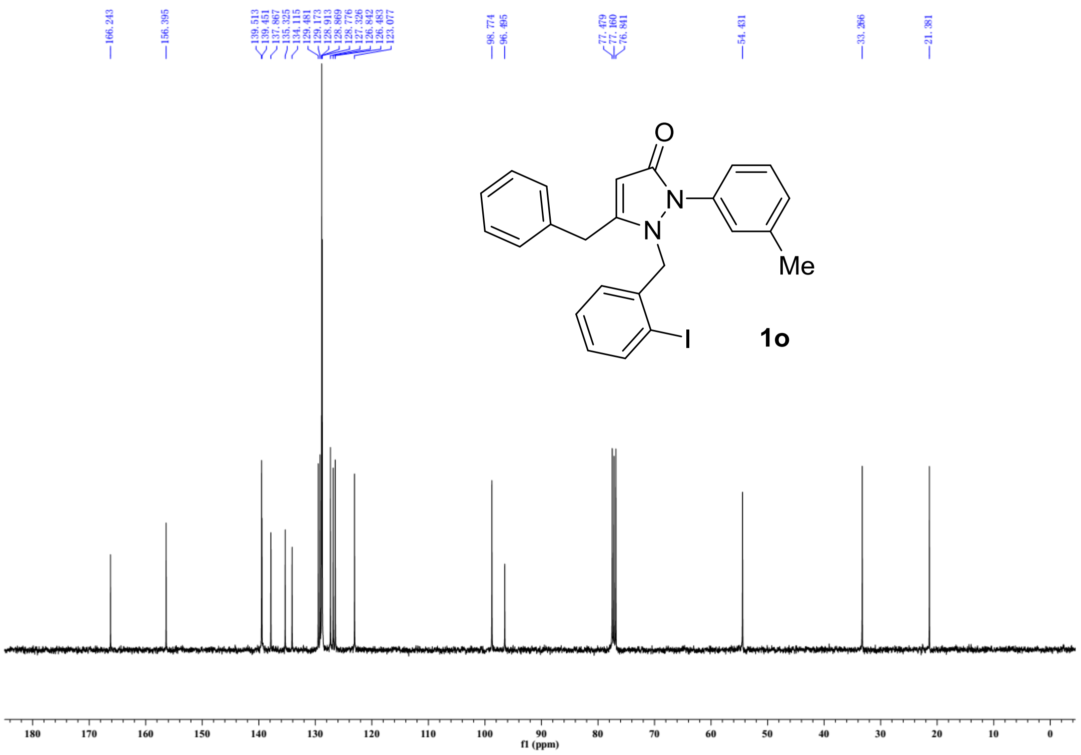

Figure S178. **^{13}C NMR of 1o, related to Table 2.**

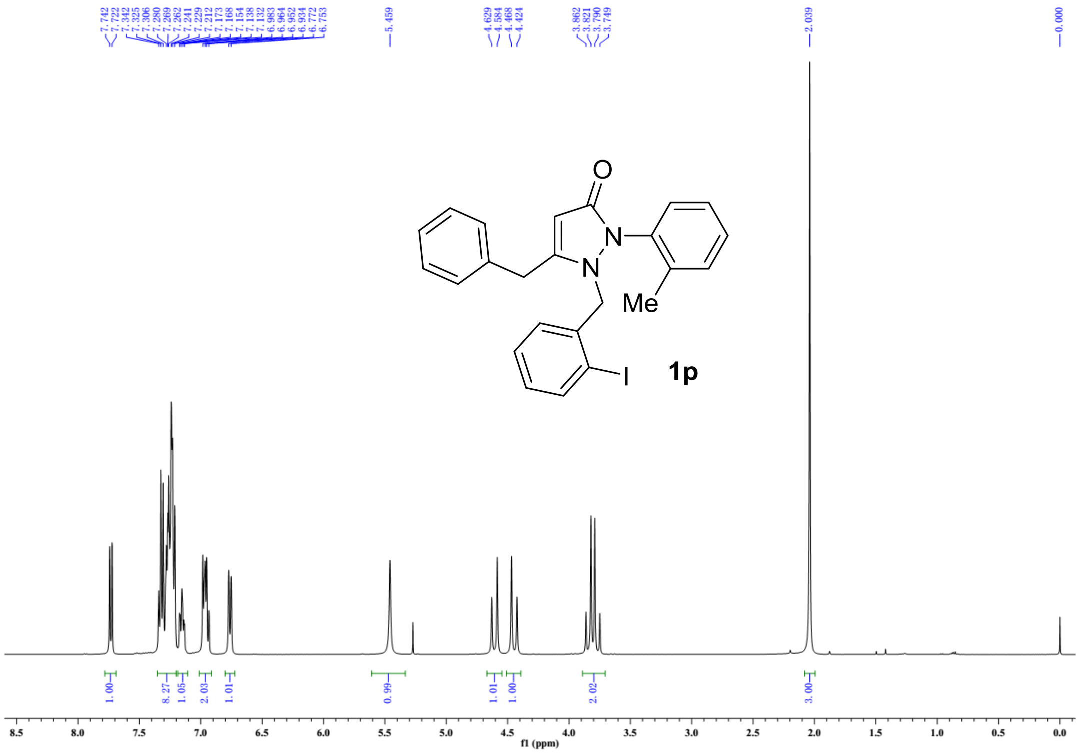

Figure S179. **^{1}H NMR of 1p, related to Table 2.**

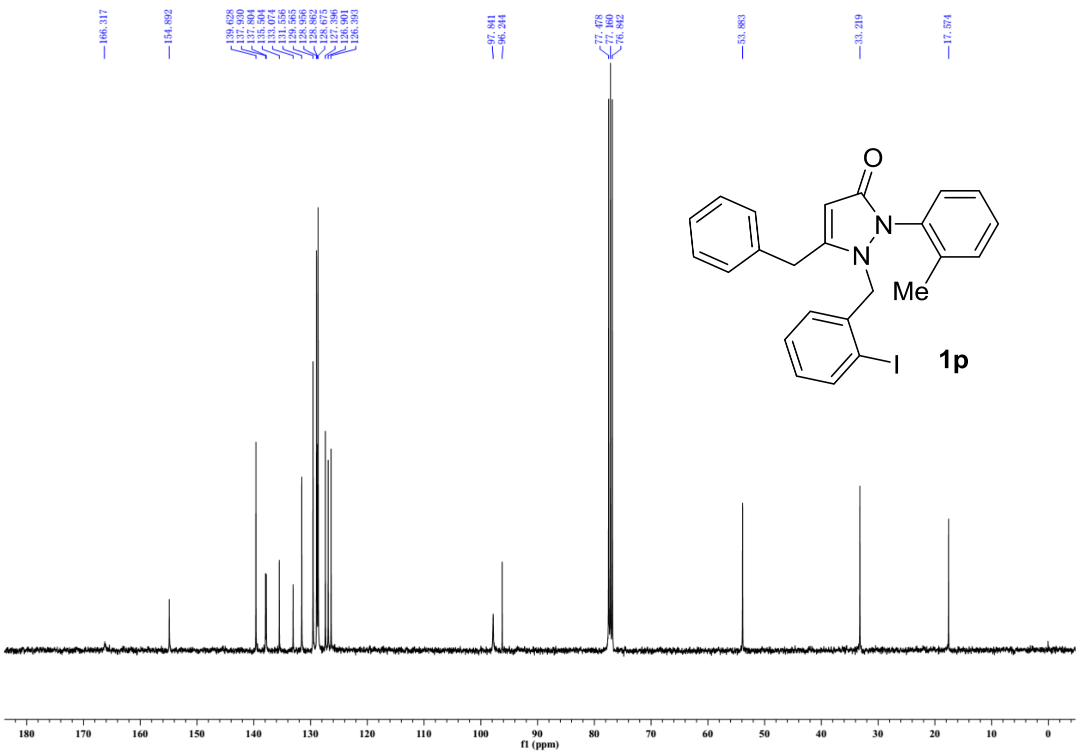

Figure S180. **^{13}C NMR of 1p, related to Table 2.**

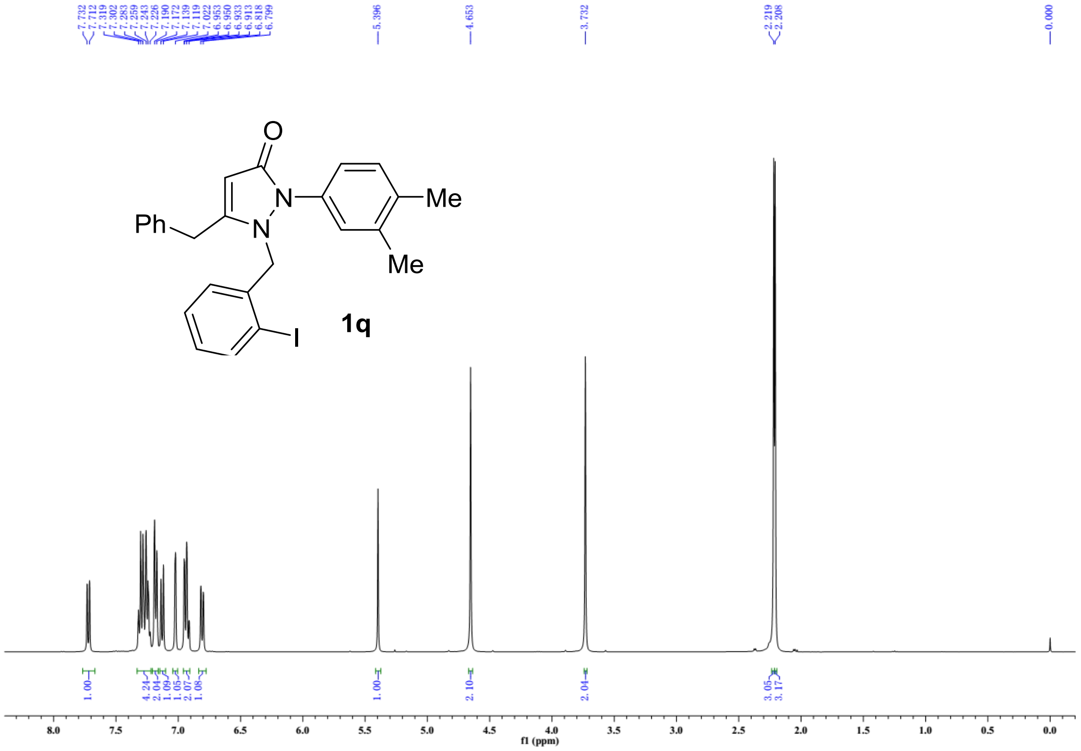

Figure S181. **^{1}H NMR of 1q, related to Table 2.**

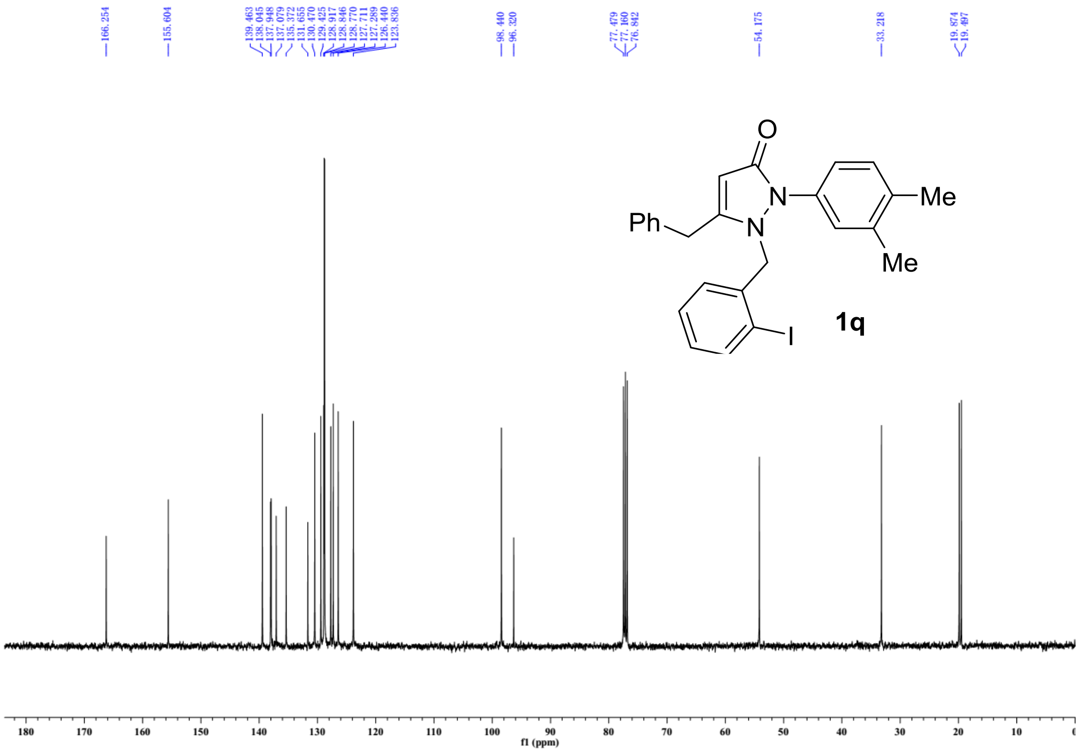

Figure S182. **^{13}C NMR of 1q, related to Table 2.**

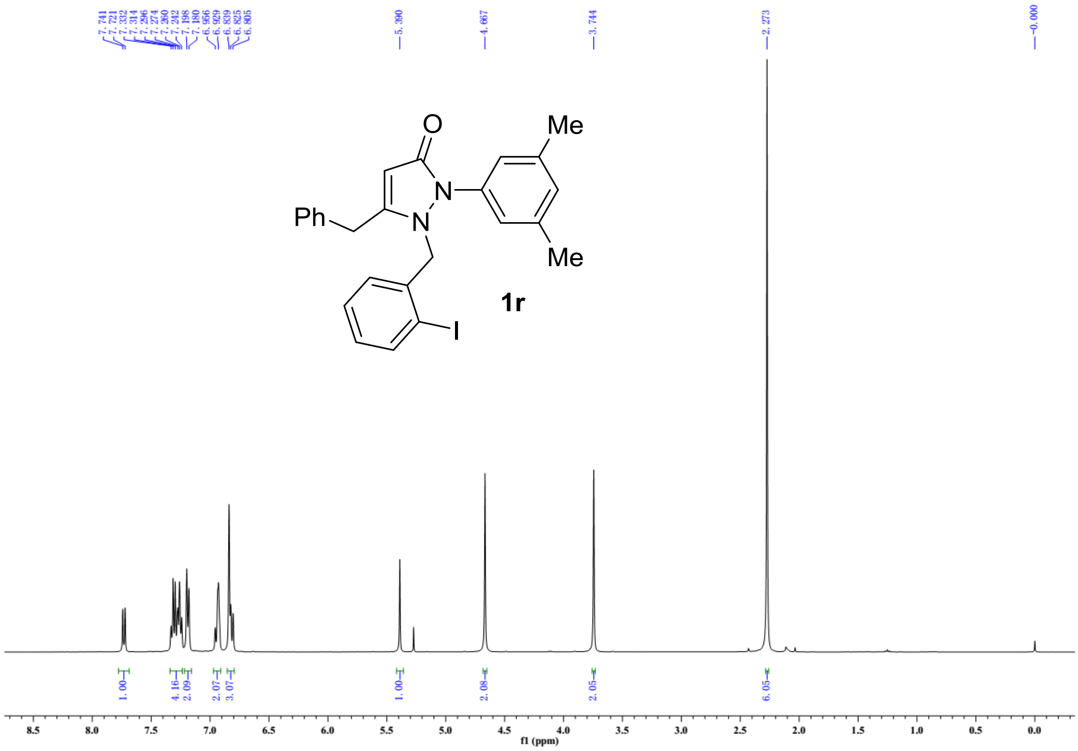

Figure S183. **^{1}H NMR of 1r, related to Table 2.**

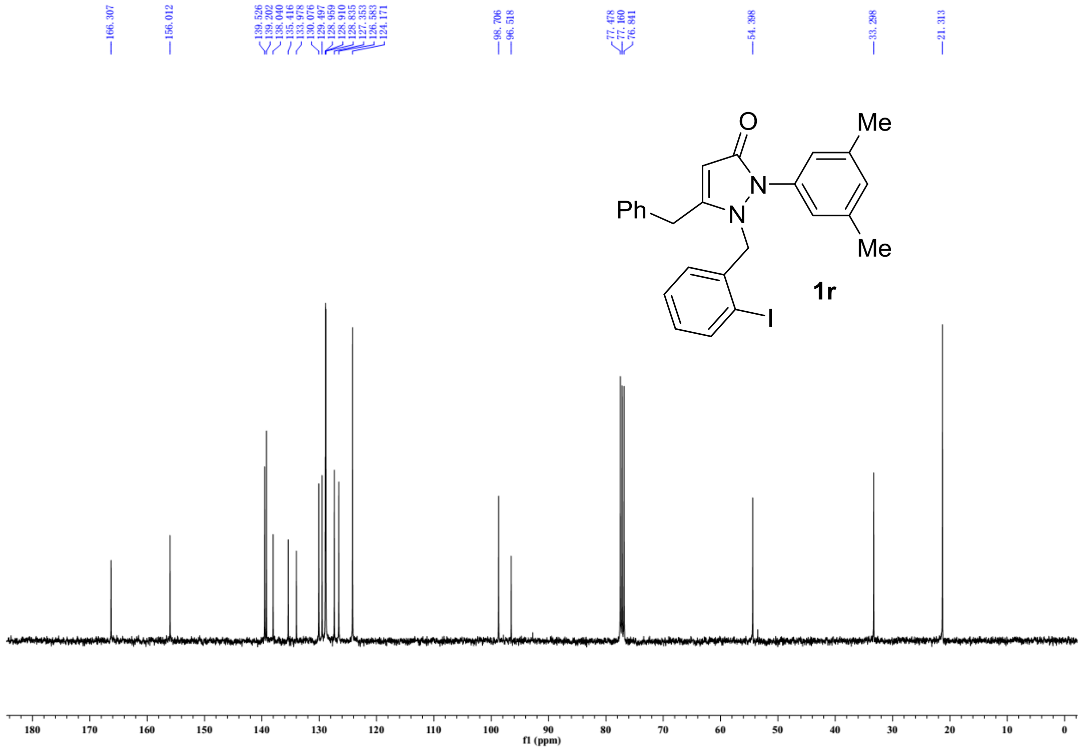

Figure S184. ^{13}C NMR of 1r, related to Table 2.

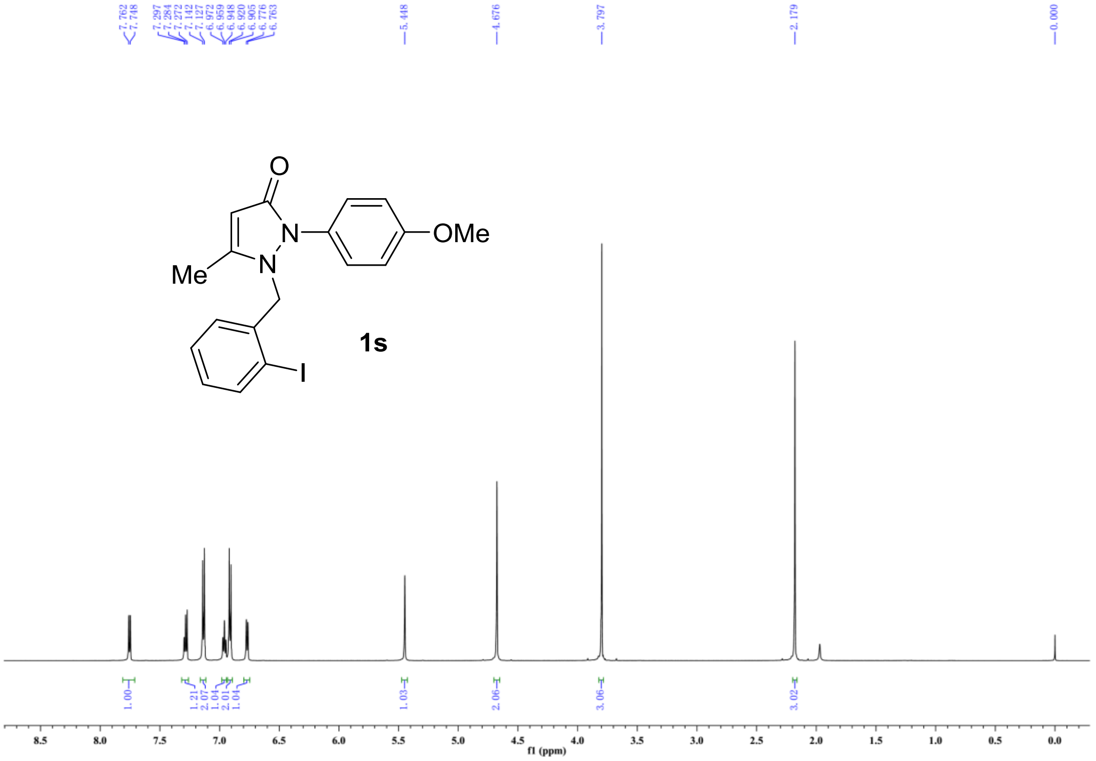

Figure S185. ^{1}H NMR of 1s, related to Table 2.

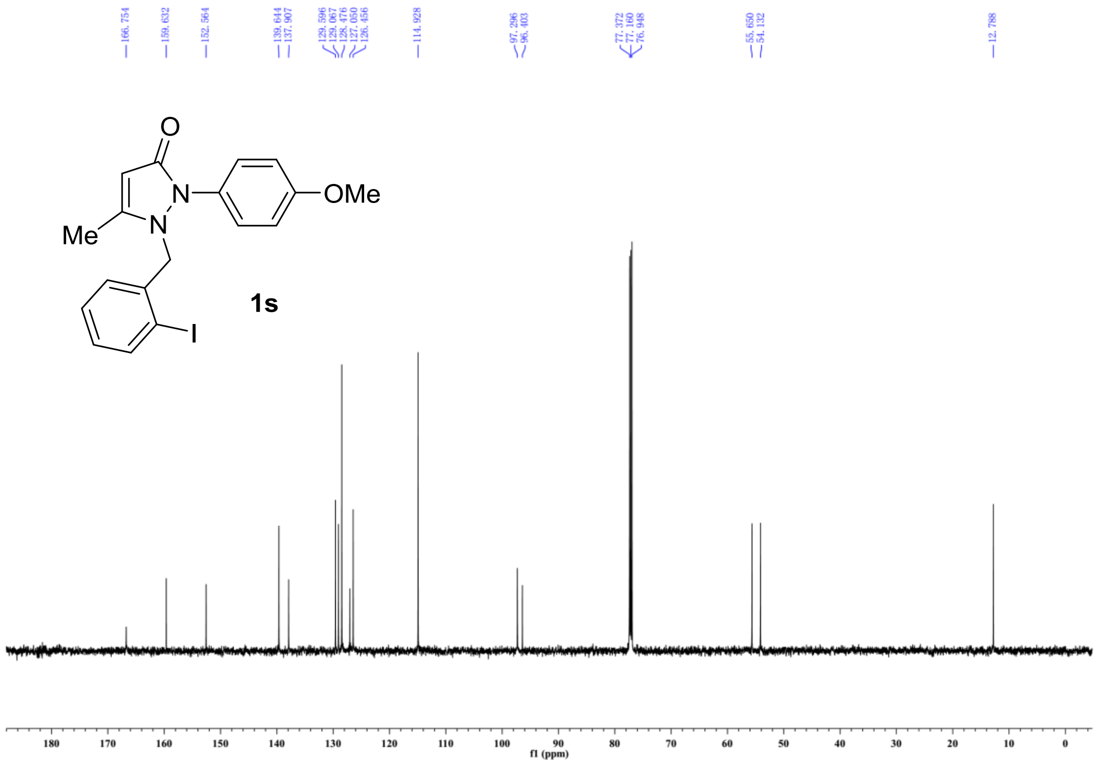

Figure S186. ^{13}C NMR of 1s, related to Table 2.

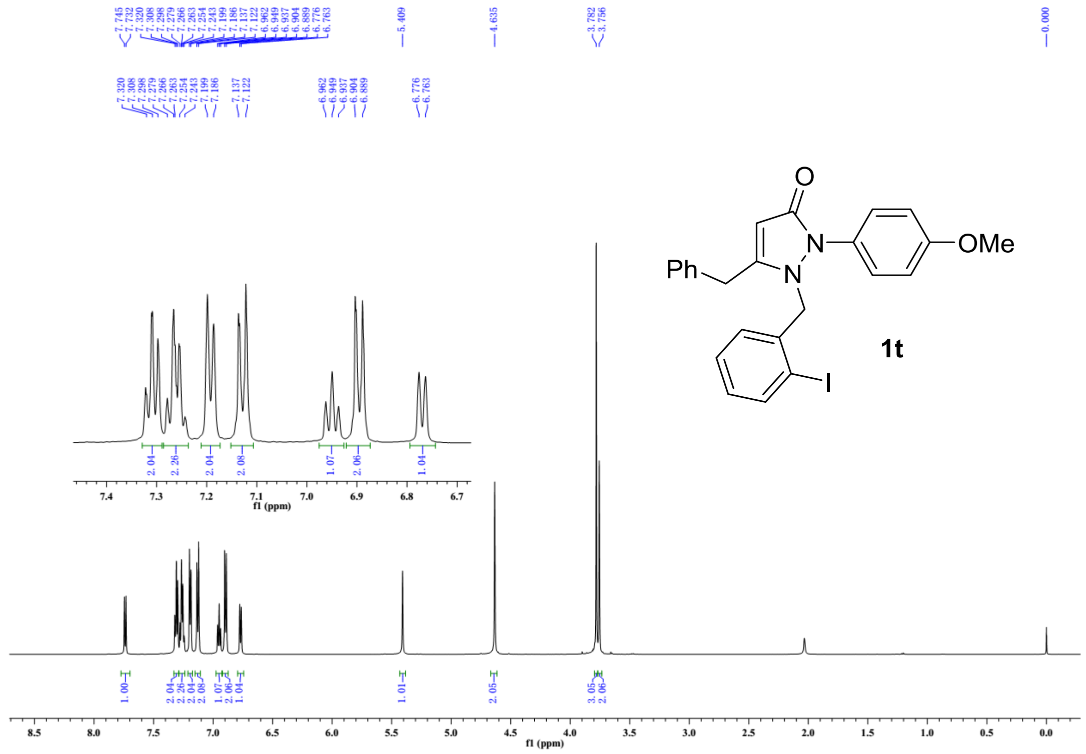

Figure S187. ^{1}H NMR of 1t, related to Table 2.

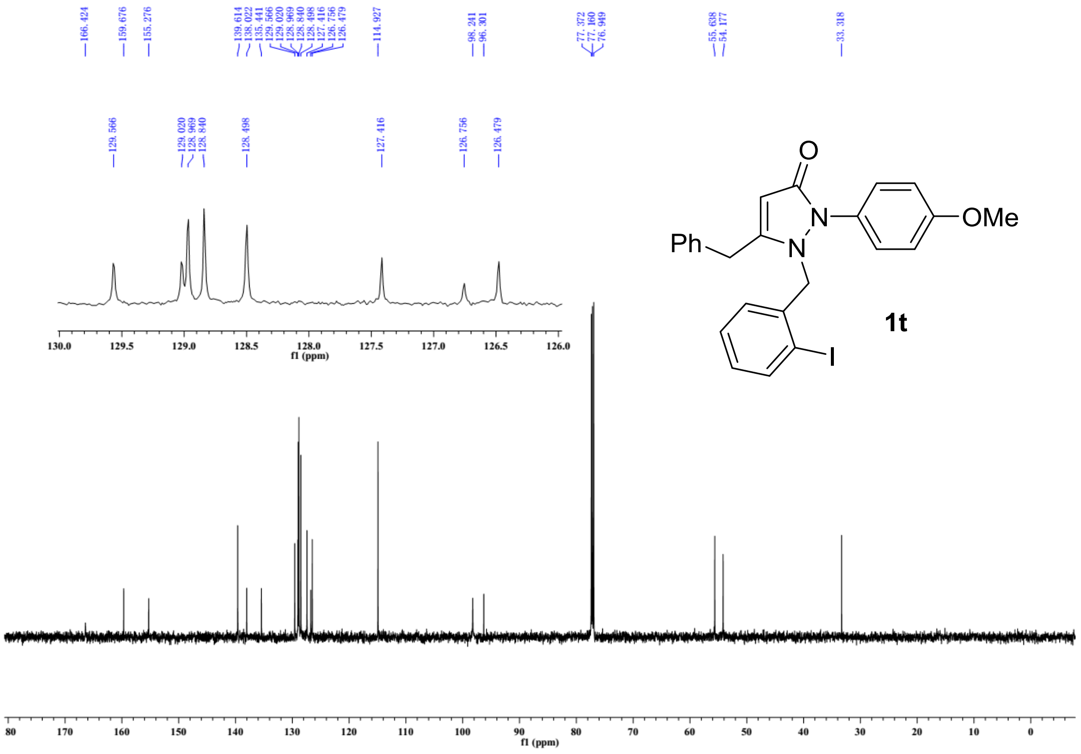

Figure S188. ^{13}C NMR of 1t, related to Table 2.

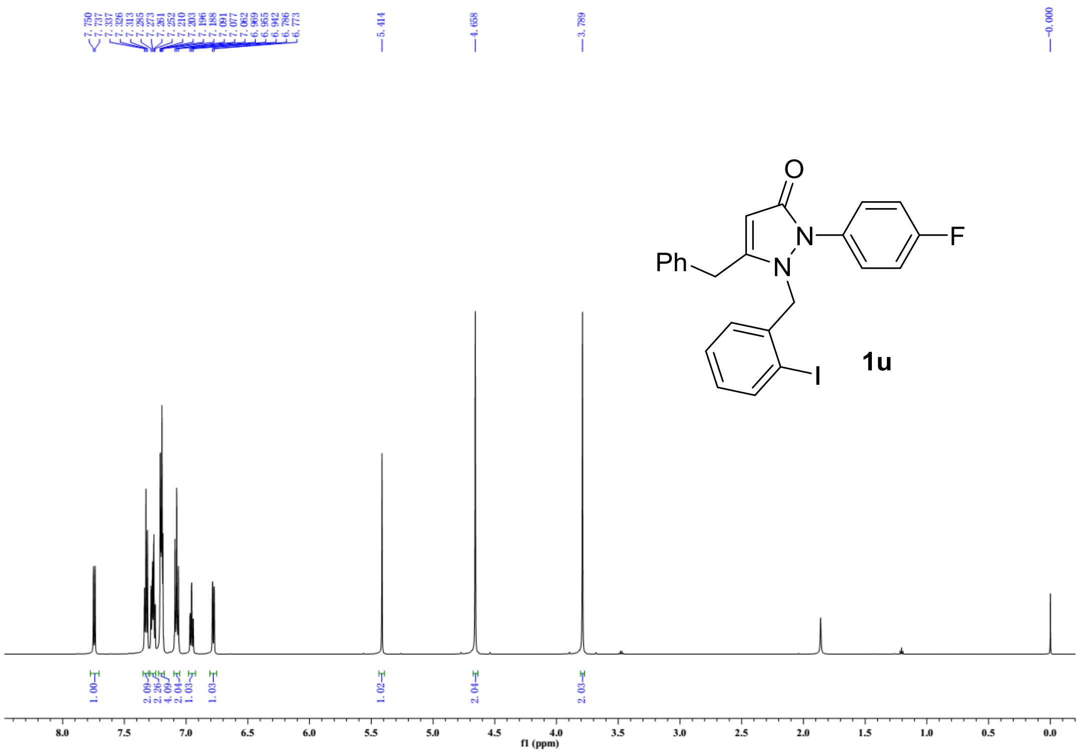

Figure S189. ^{1}H NMR of 1u, related to Table 2.

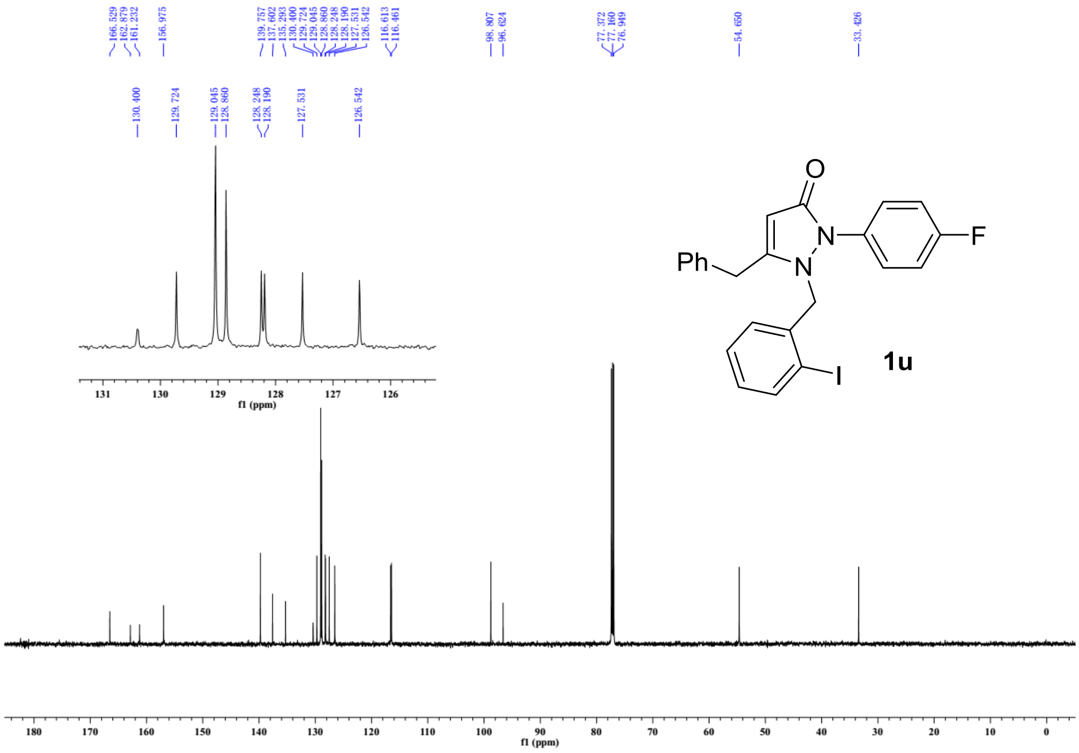

Figure S190. ^{13}C NMR of 1u, related to Table 2.

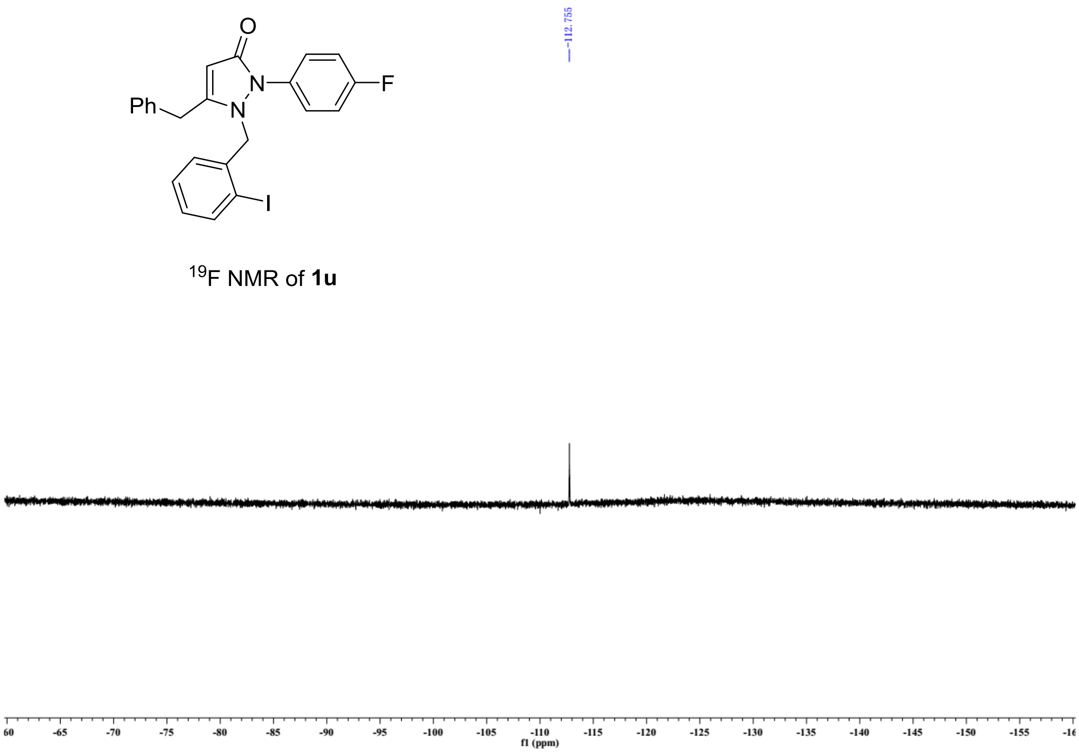

Figure S191. ^{19}F NMR of 1u, related to Table 2.

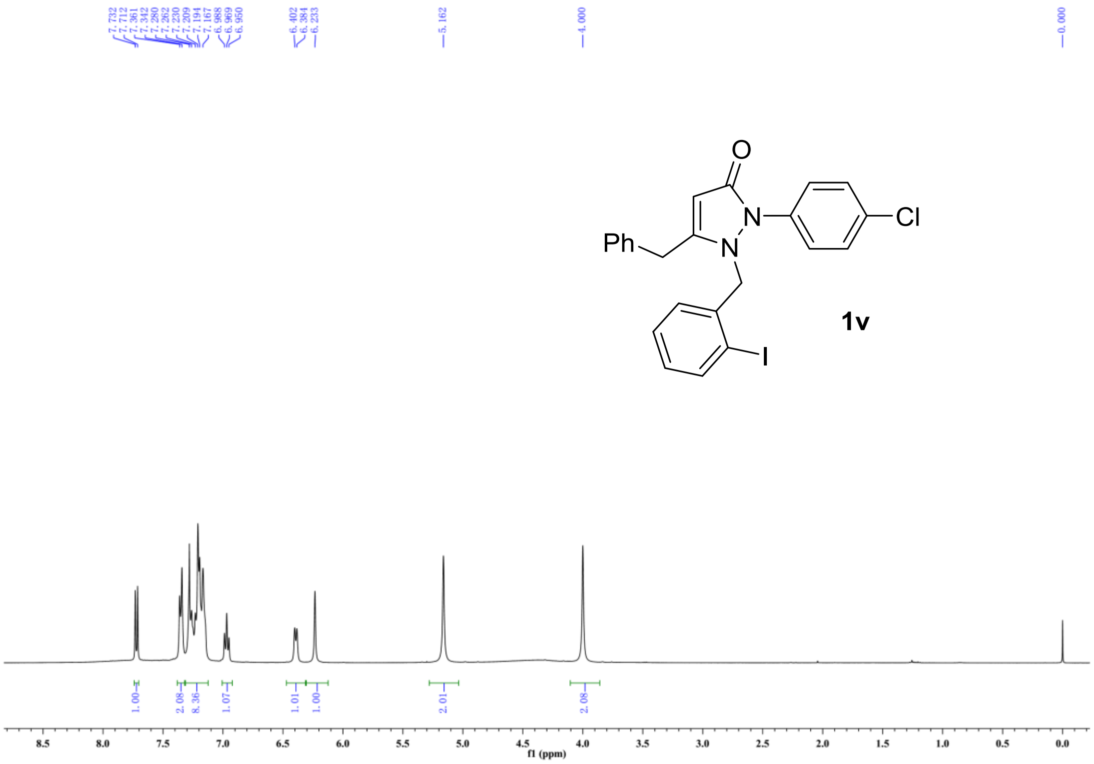

Figure S192. ^{1}H NMR of 1v, related to Table 2.

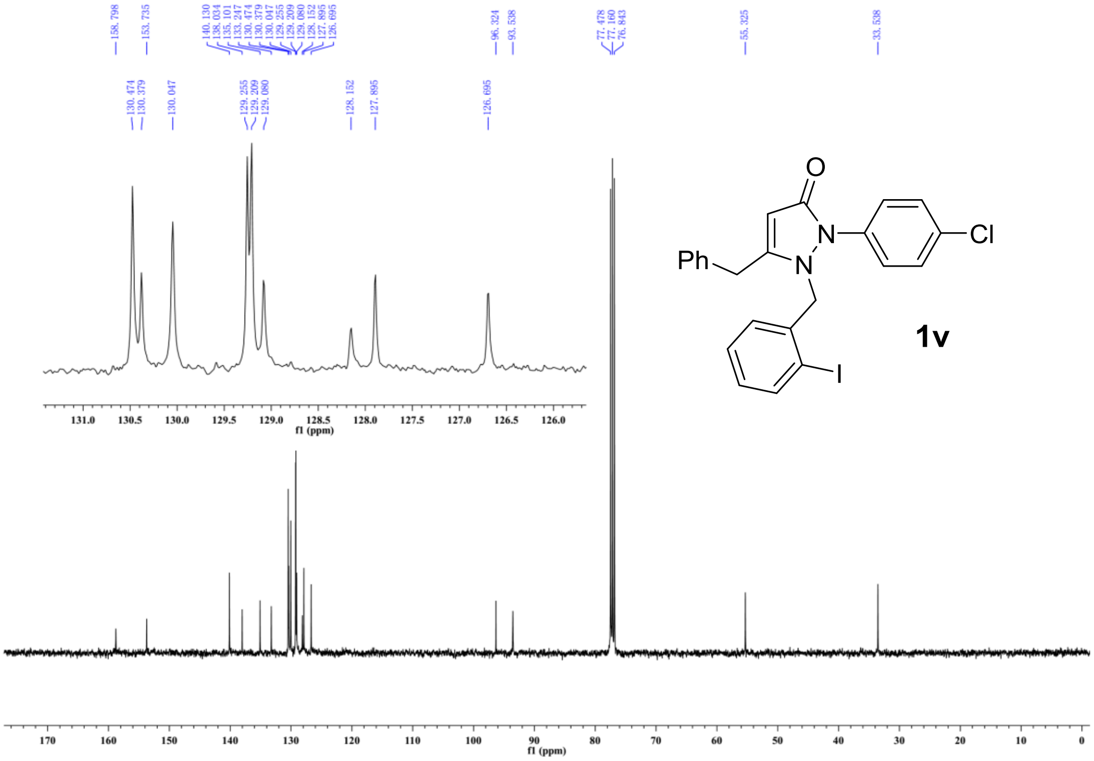

Figure S193. ^{13}C NMR of 1v, related to Table 2.

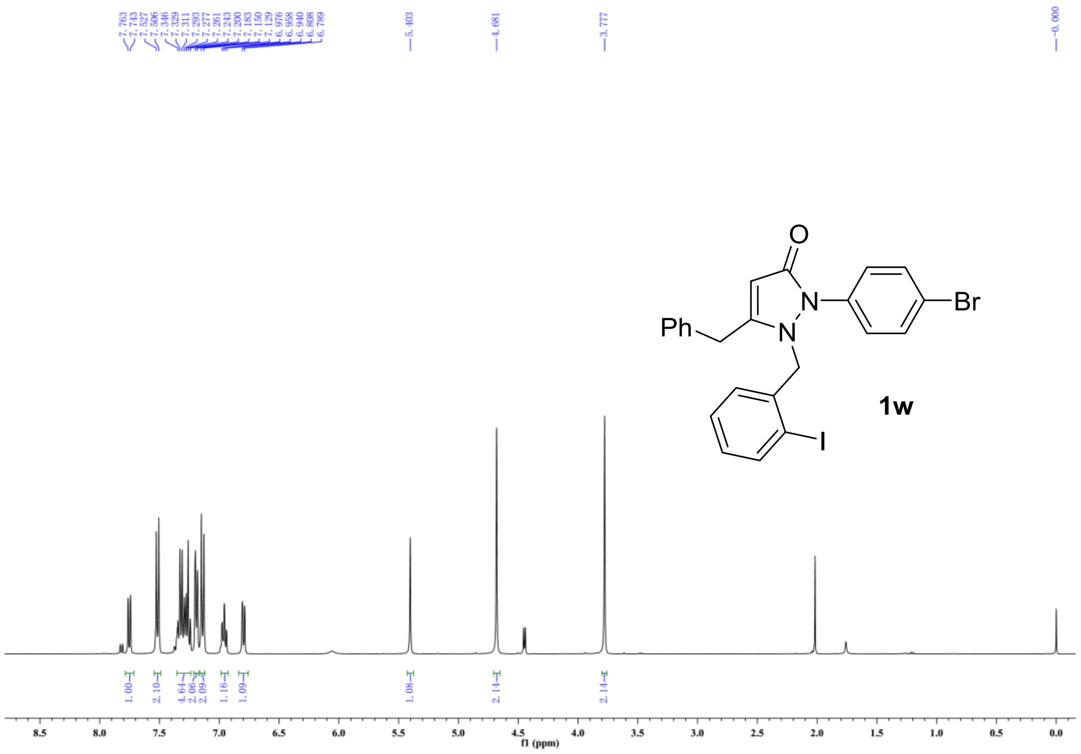

Figure S194. ^{1}H NMR of 1w, related to Table 2.

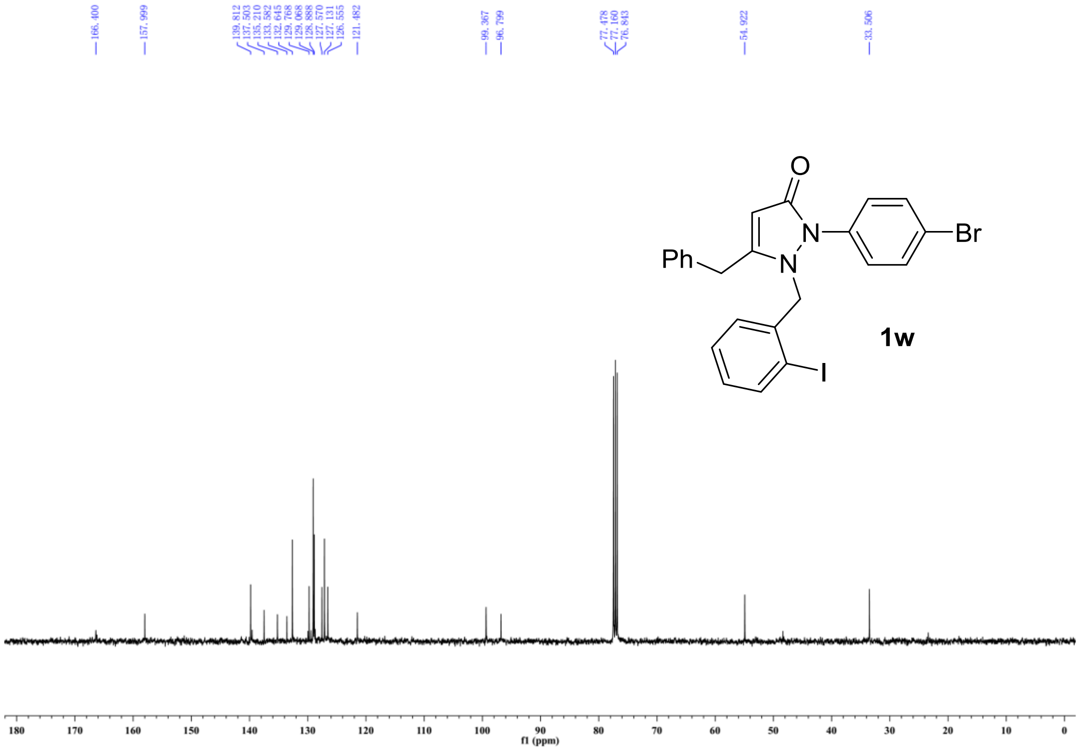

Figure S195. ^{13}C NMR of 1w, related to Table 2.

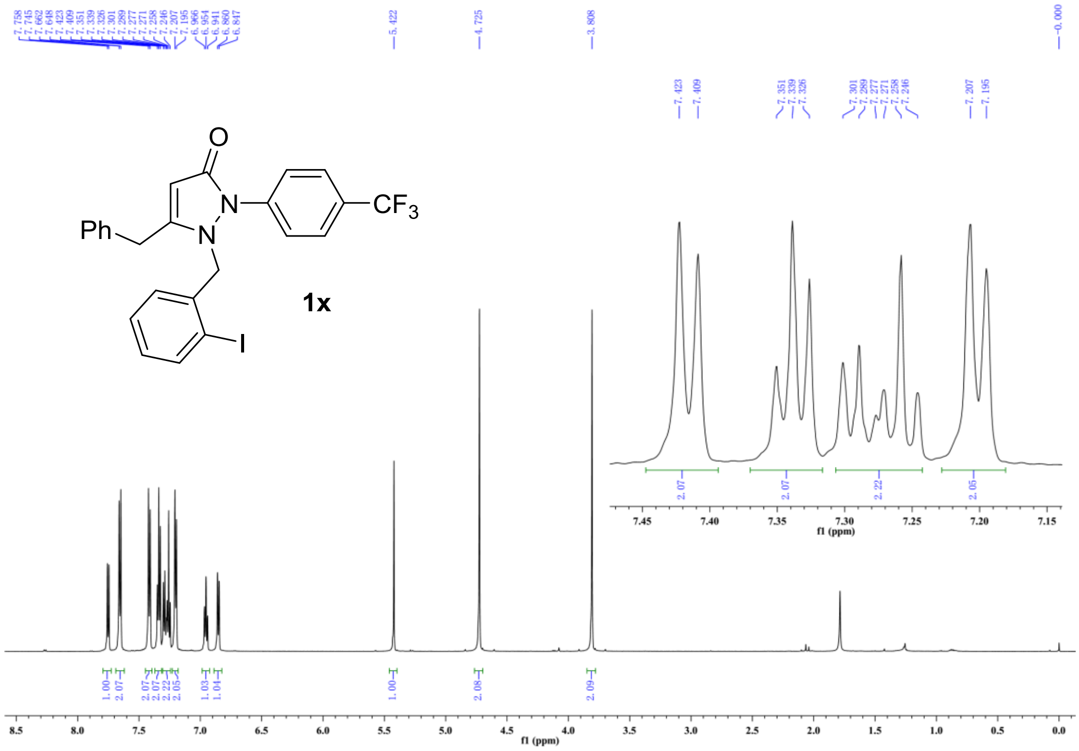

Figure S196. ^{1}H NMR of 1x, related to Table 2.

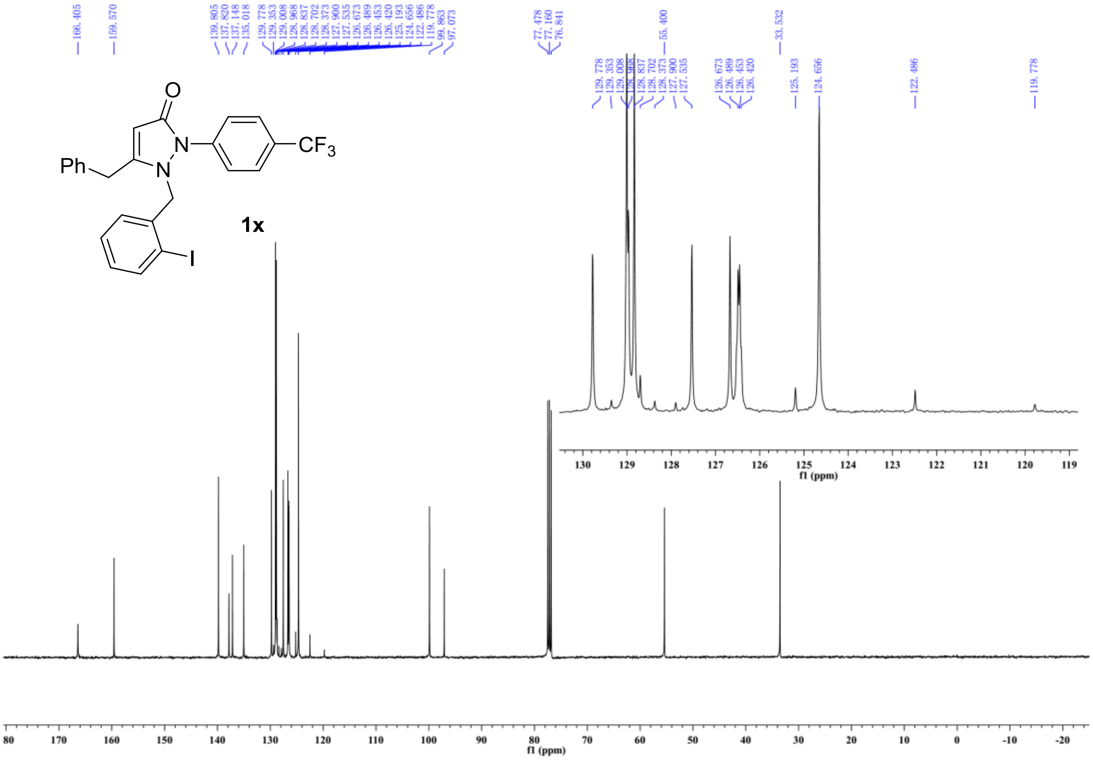

Figure S197. ^{13}C NMR of 1x, related to Table 2.

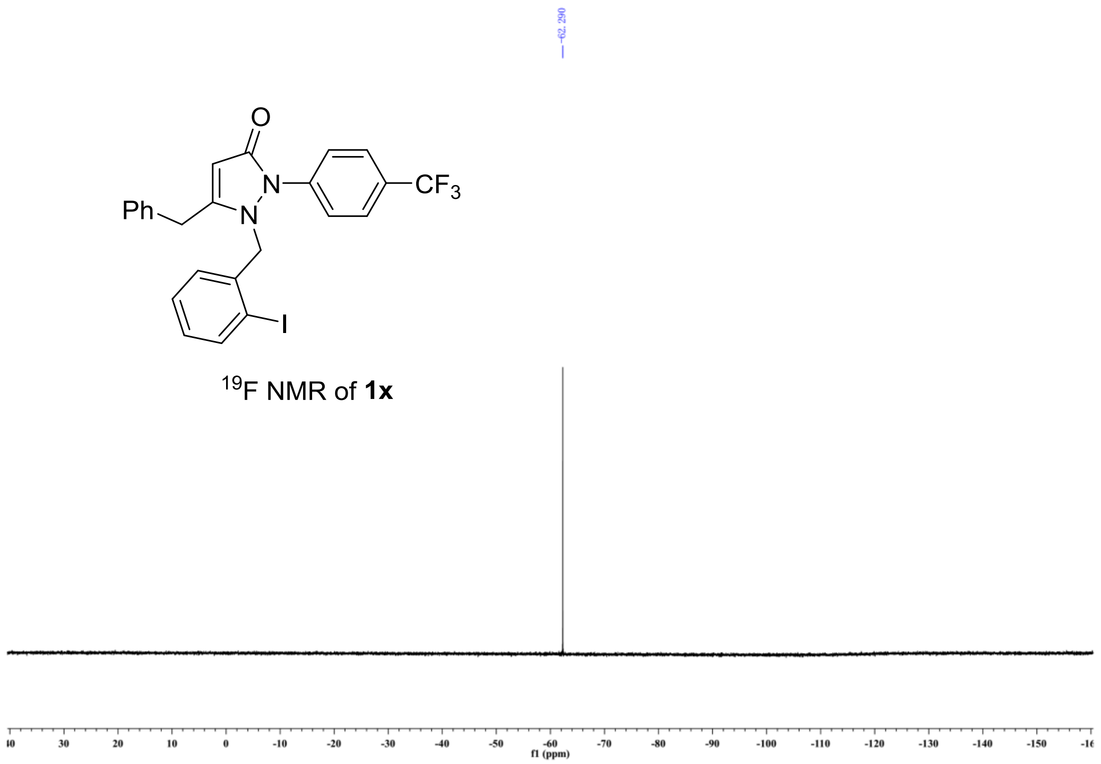

Figure S198. ^{19}F NMR of 1x, related to Table 2.

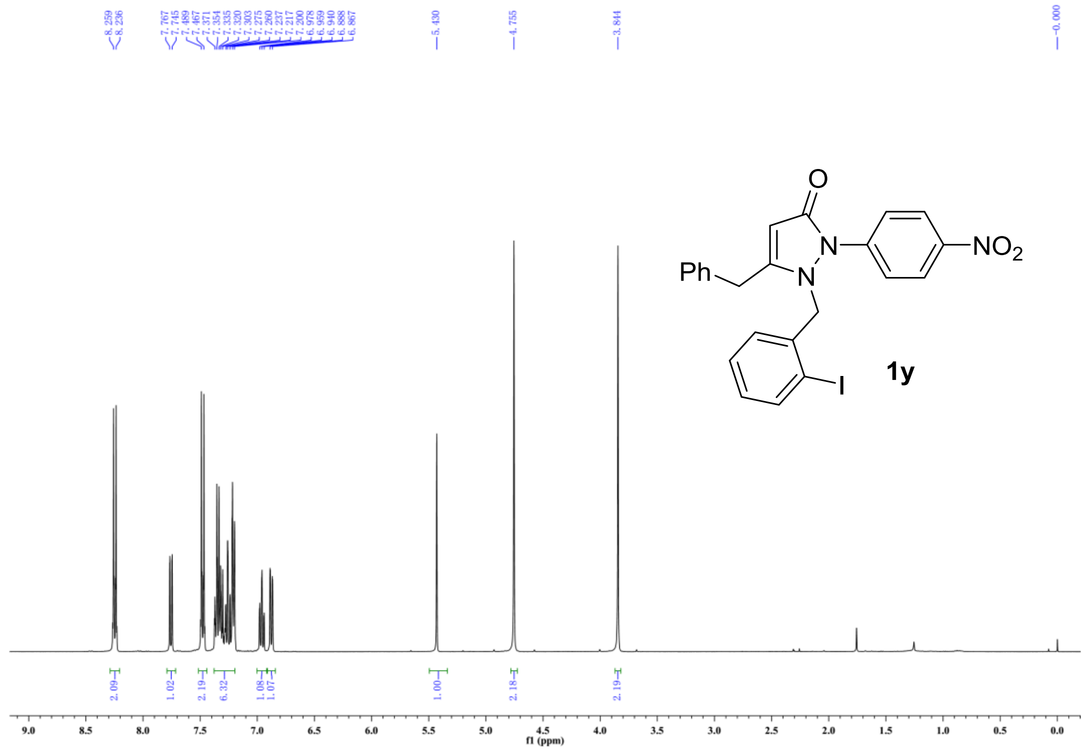

Figure S199. ^{1}H NMR of 1y, related to Table 2.

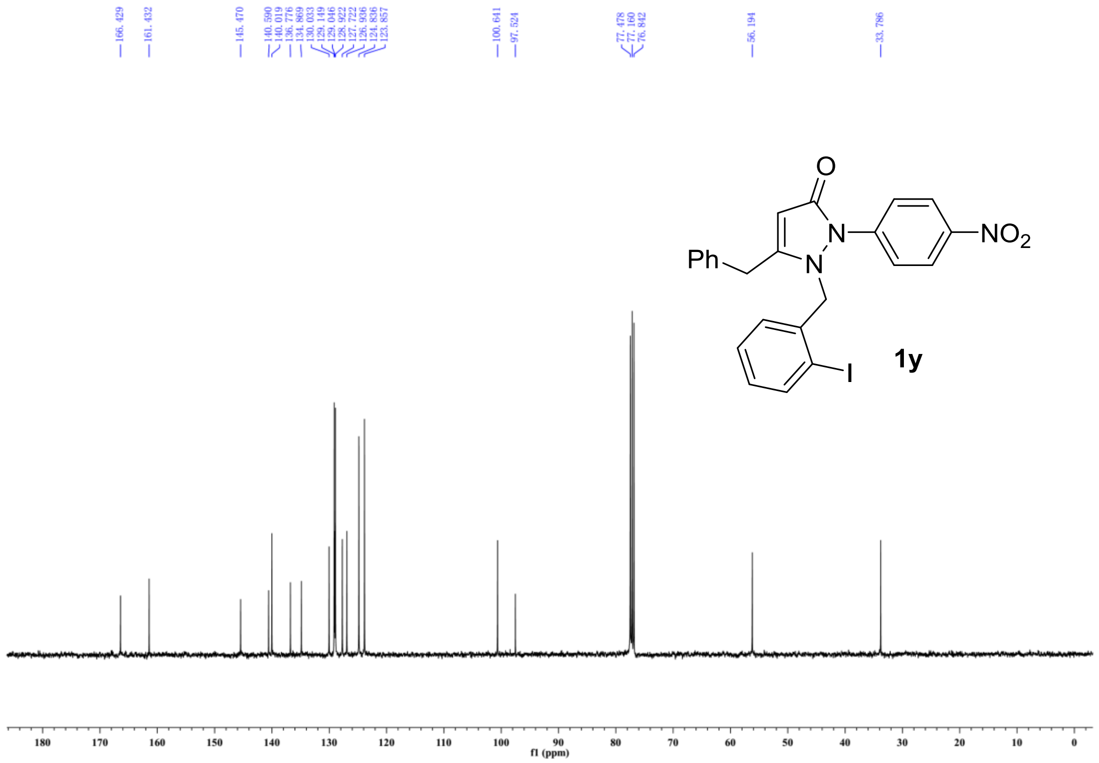

Figure S200. ^{13}C NMR of 1y, related to Table 2.

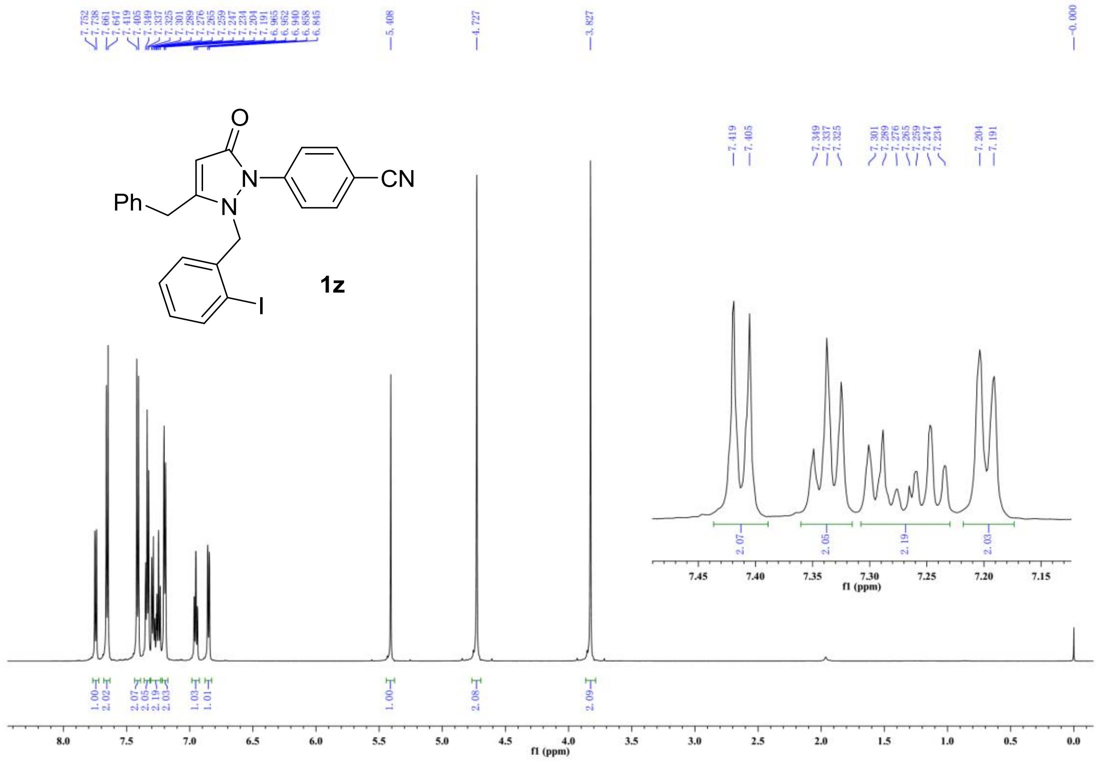

Figure S201. ^{1}H NMR of 1z, related to Table 2.

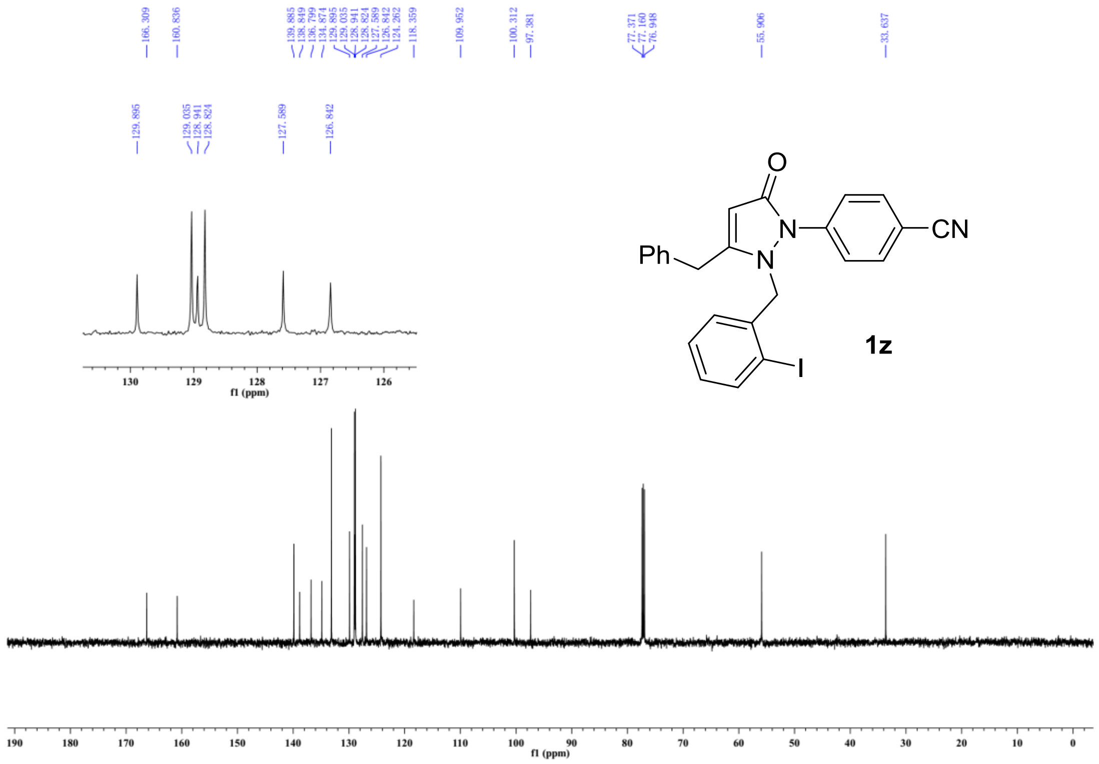

Figure S202. ^{13}C NMR of 1z, related to Table 2.

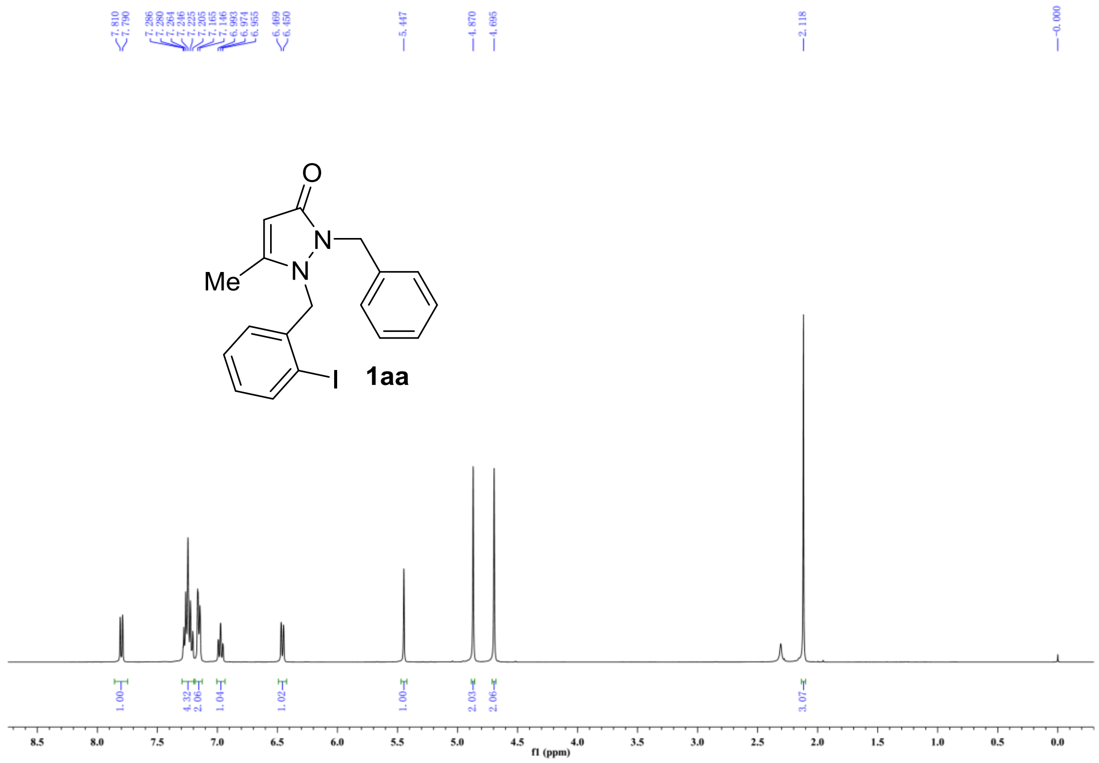

Figure S203. ^{1}H NMR of 1aa, related to Table 2.

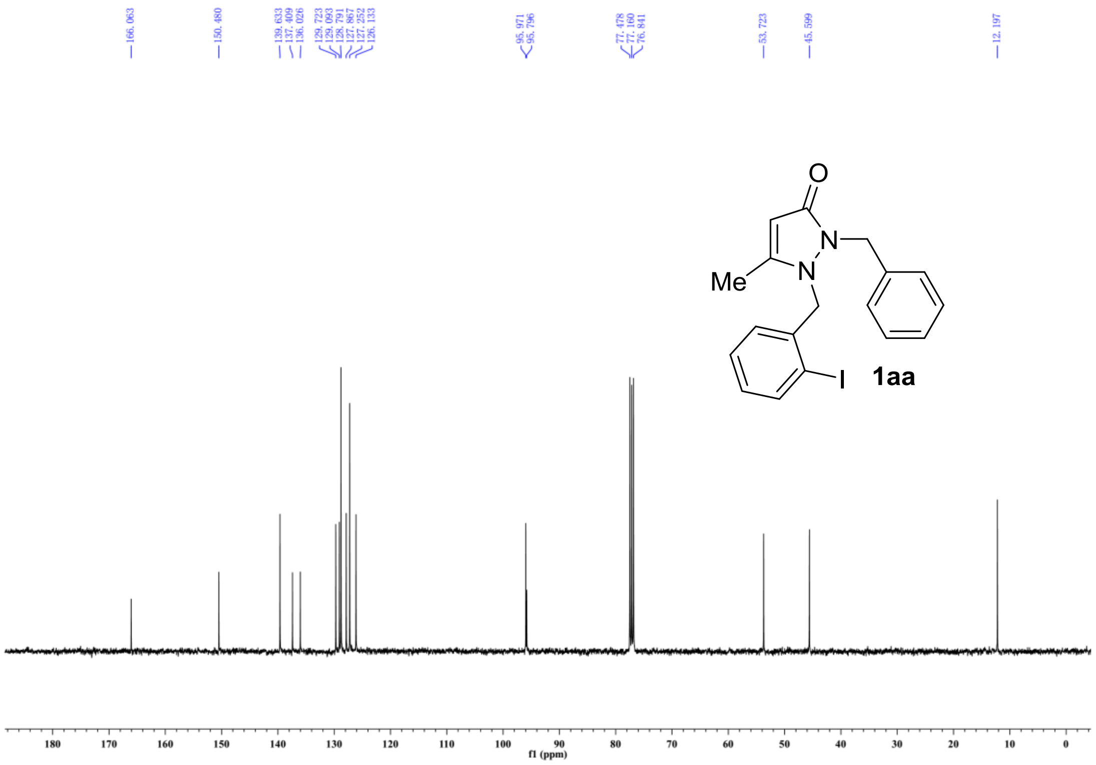

Figure S204. ^{13}C NMR of 1aa, related to Table 2.

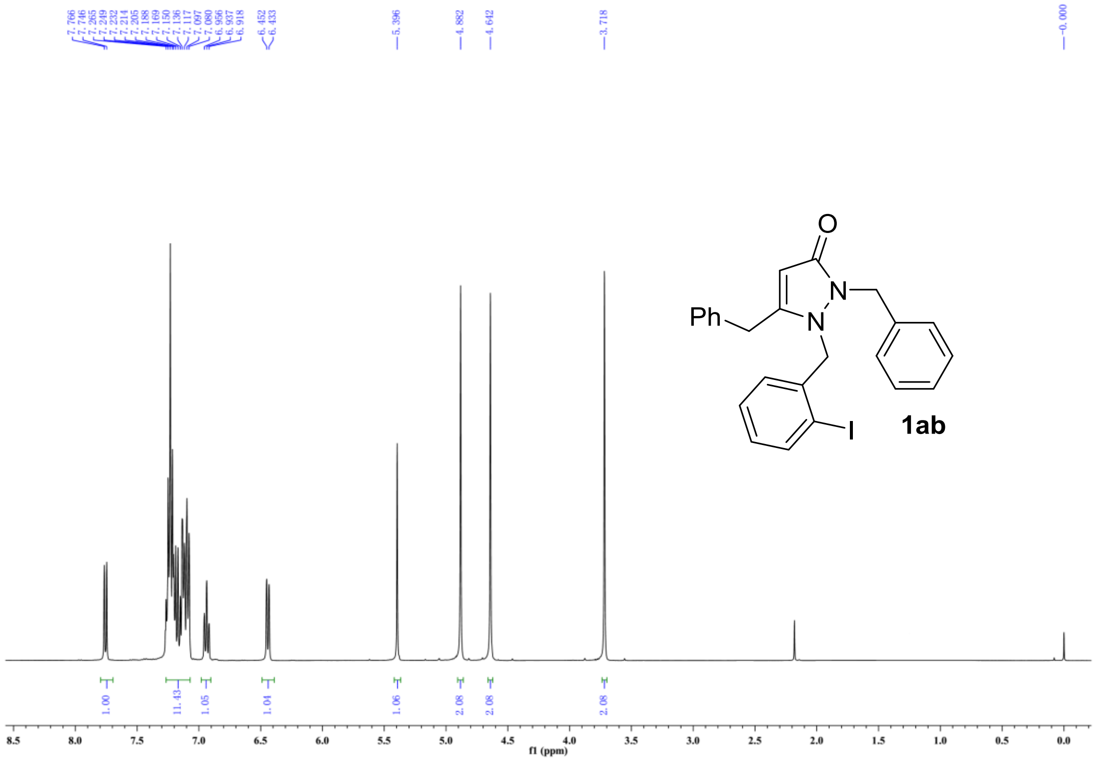

Figure S205. ^{1}H NMR of 1ab, related to Table 2.

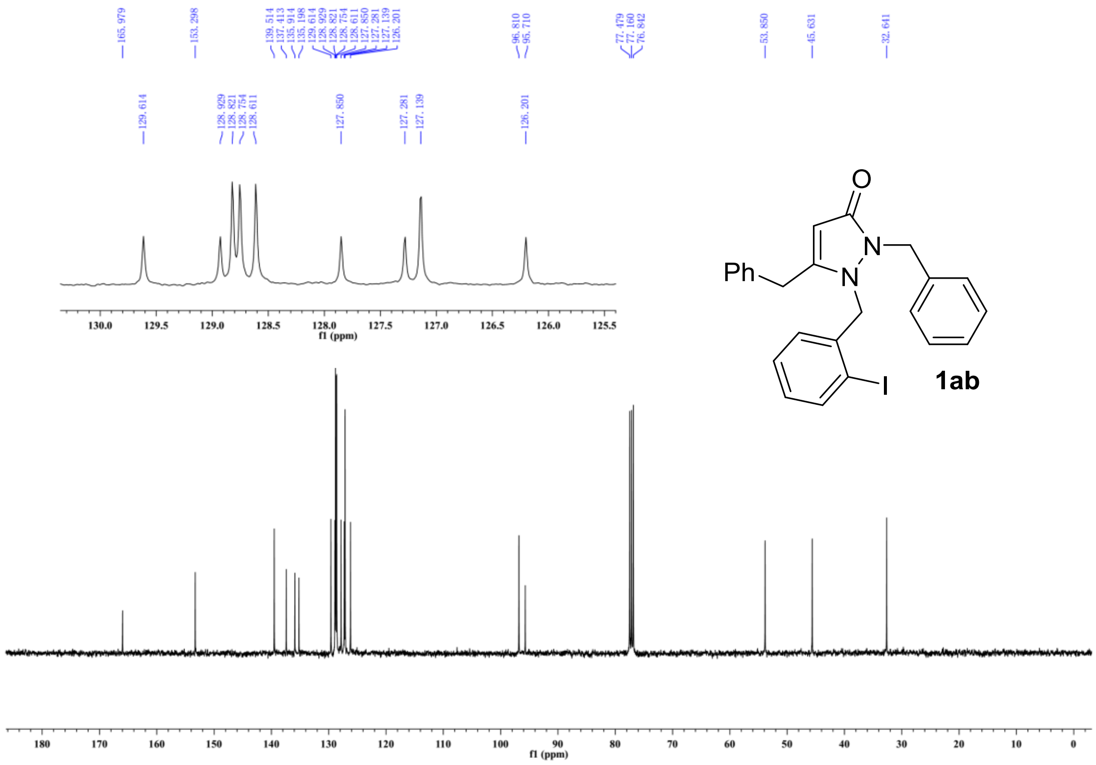

Figure S206. **^{13}C NMR of 1ab, related to Table 2.**

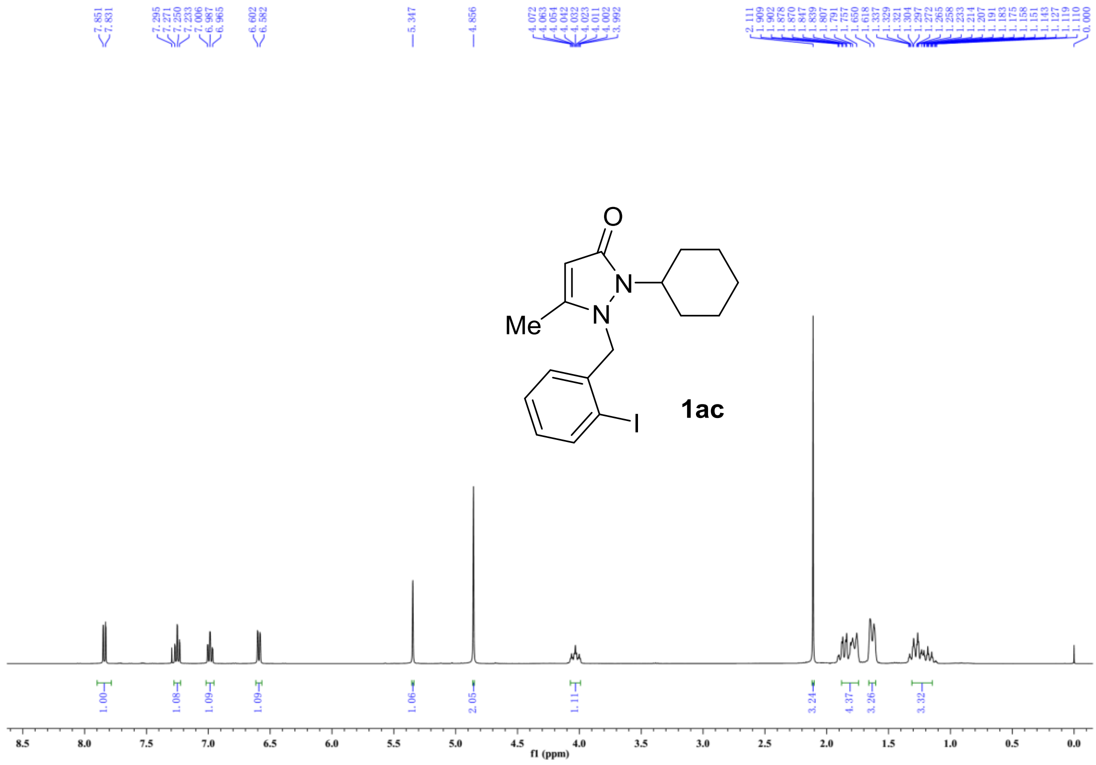

Figure S207. **^{1}H NMR of 1ac, related to Table 2.**

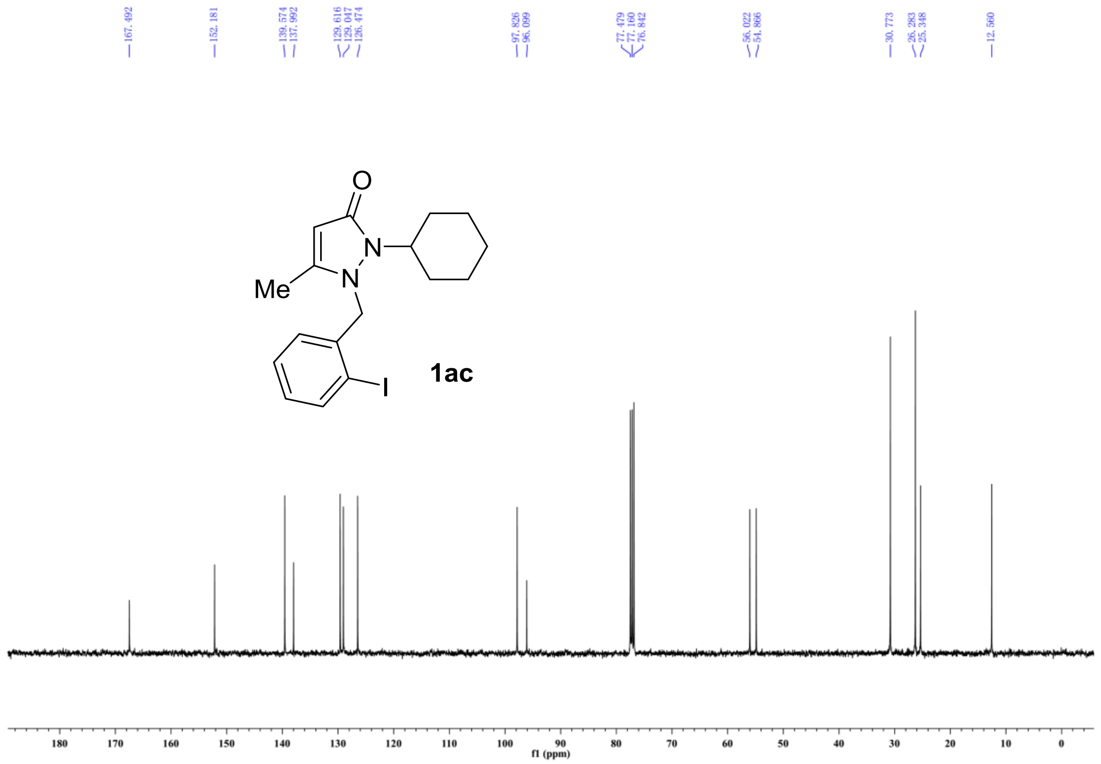

Figure S208. ^{13}C NMR of 1ac, related to Table 2.

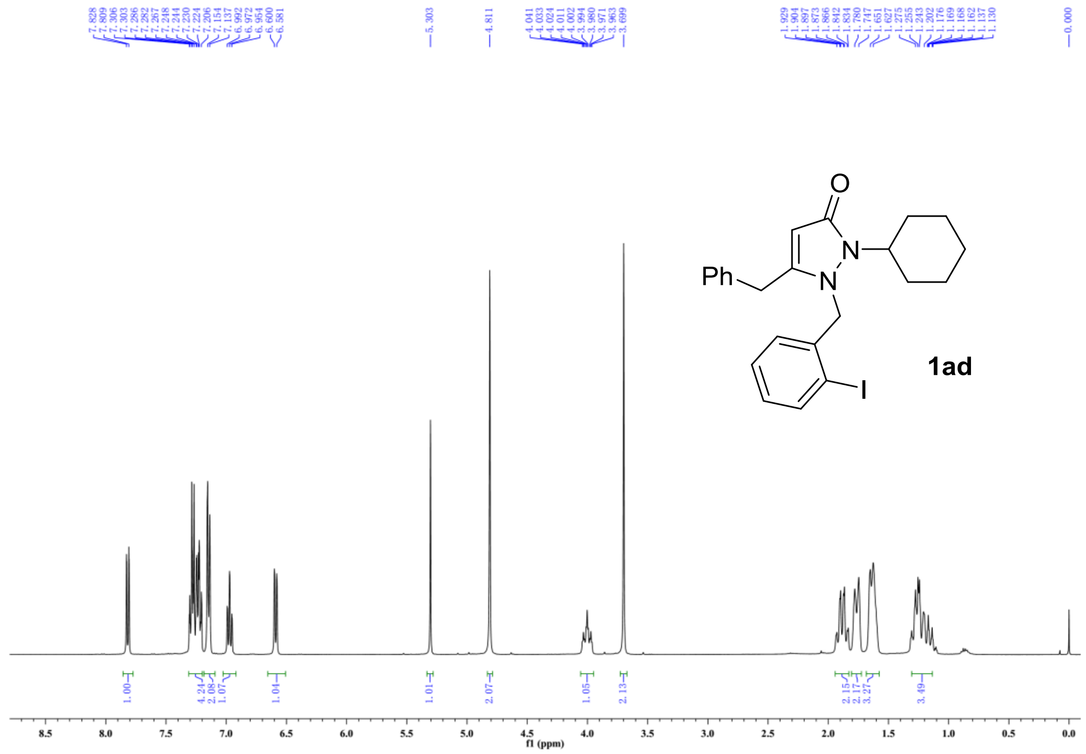

Figure S209. ^{1}H NMR of 1ad, related to Table 2.

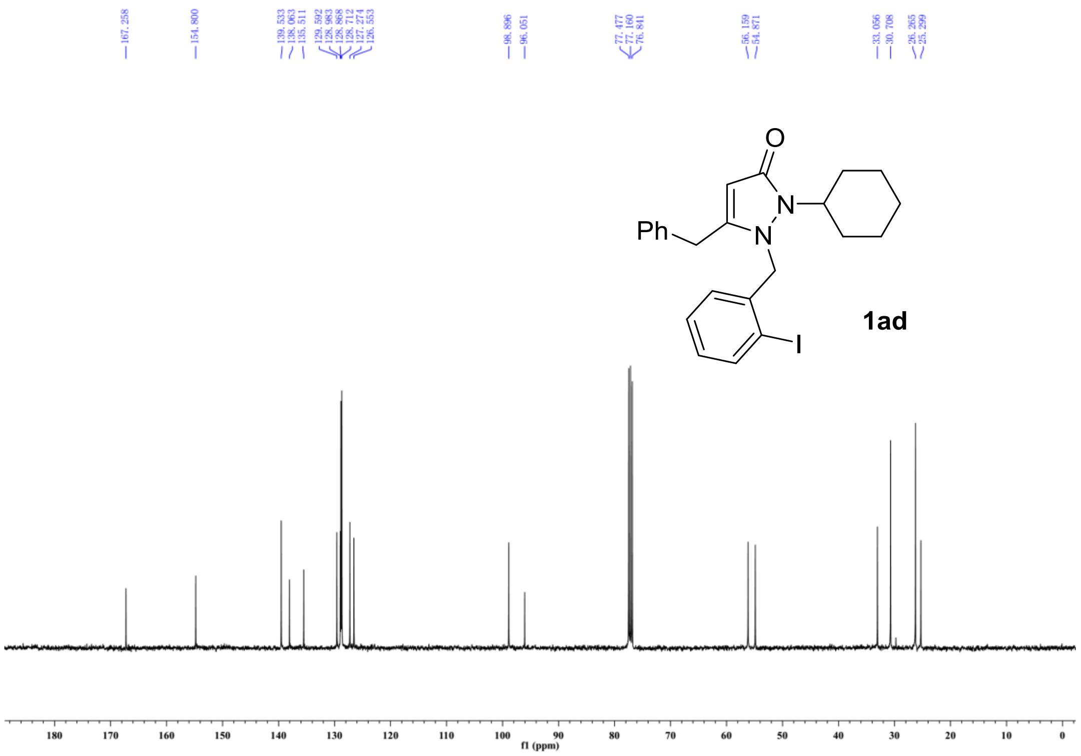

Figure S210. ^{13}C NMR of 1ad, related to Table 2.

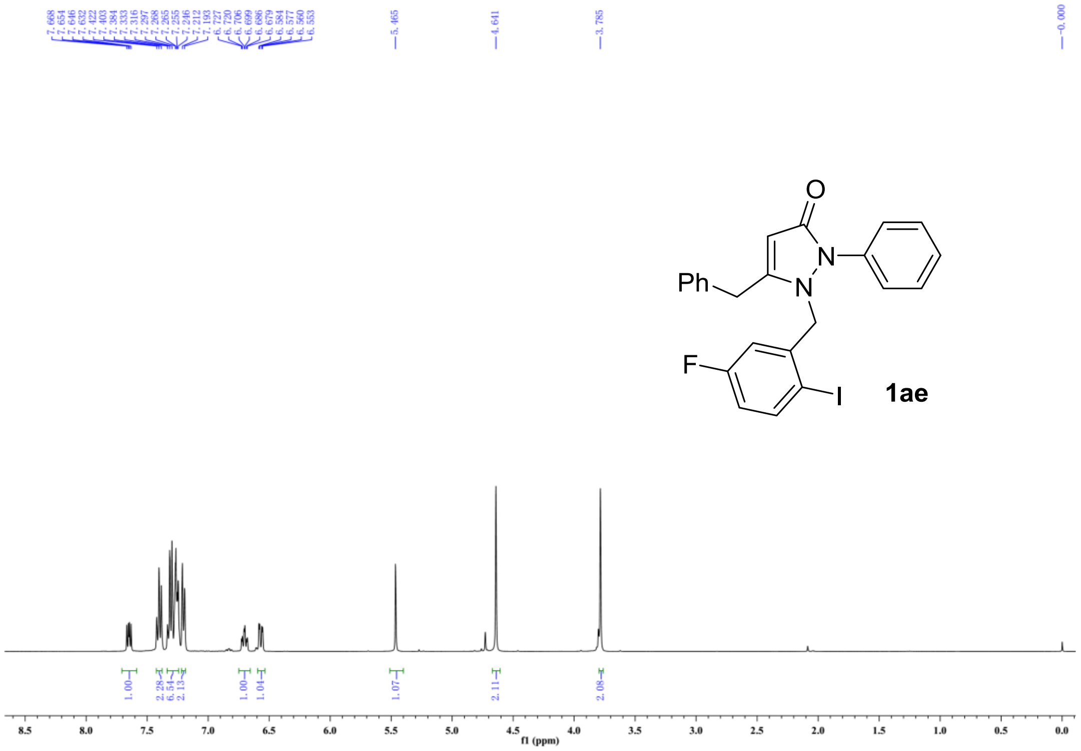

Figure S211. ^{1}H NMR of 1ae, related to Table 2.

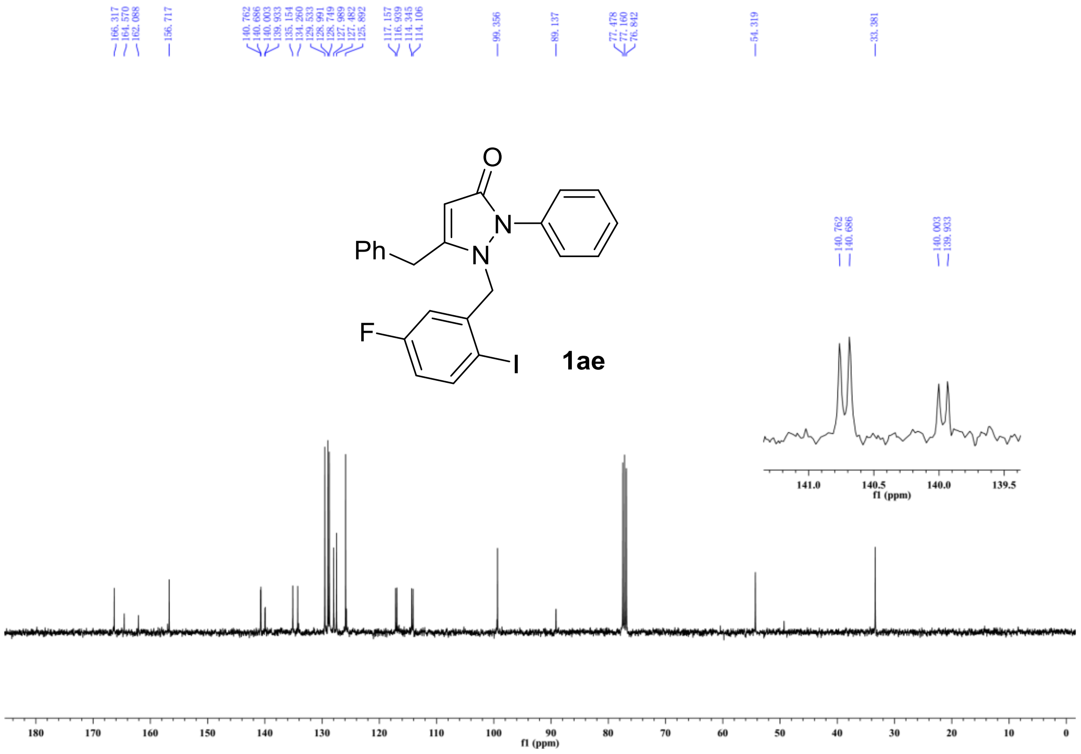

Figure S212. **^{13}C NMR of 1ae, related to Table 2.**

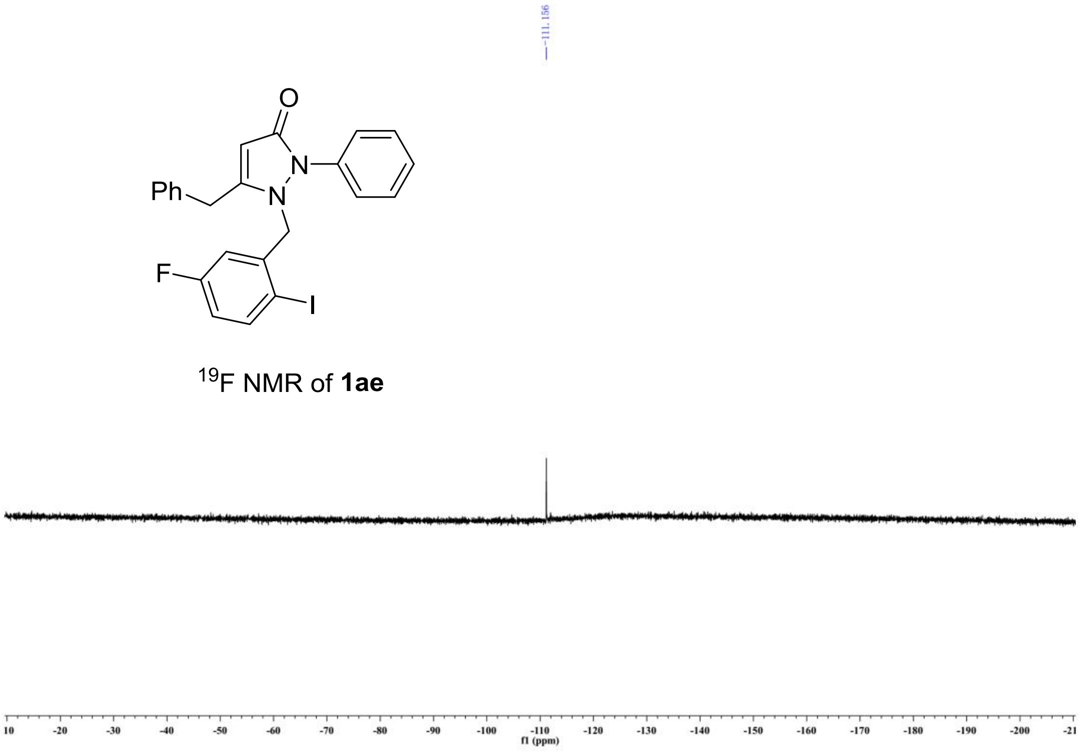

Figure S213. **^{19}F NMR of 1ae, related to Table 2.**

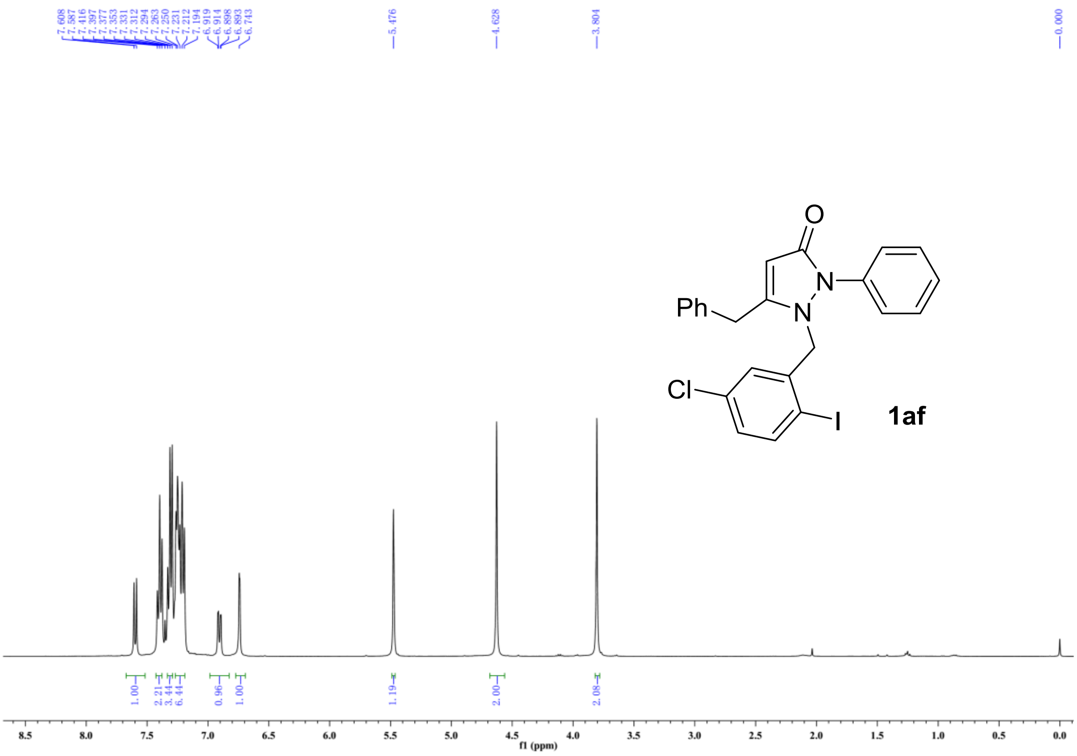

Figure S214. ^{1}H NMR of 1af, related to Table 2.

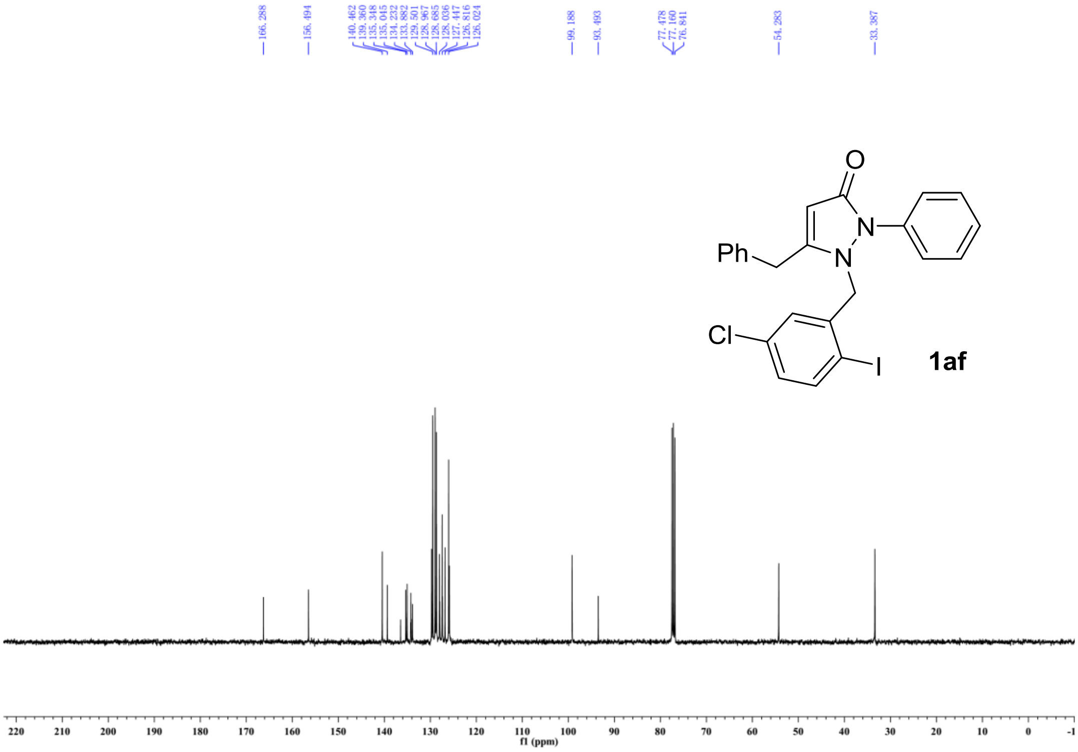

Figure S215. ^{13}C NMR of 1af, related to Table 2.

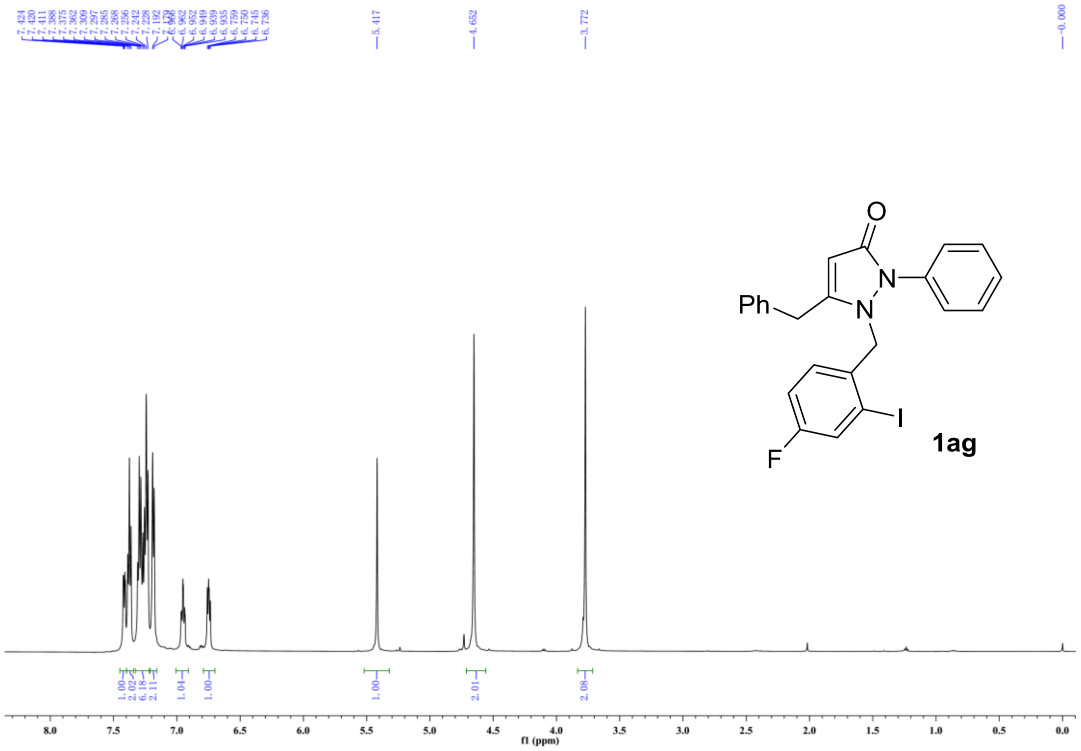

Figure S216. ^{1}H NMR of 1ag, related to Table 2.

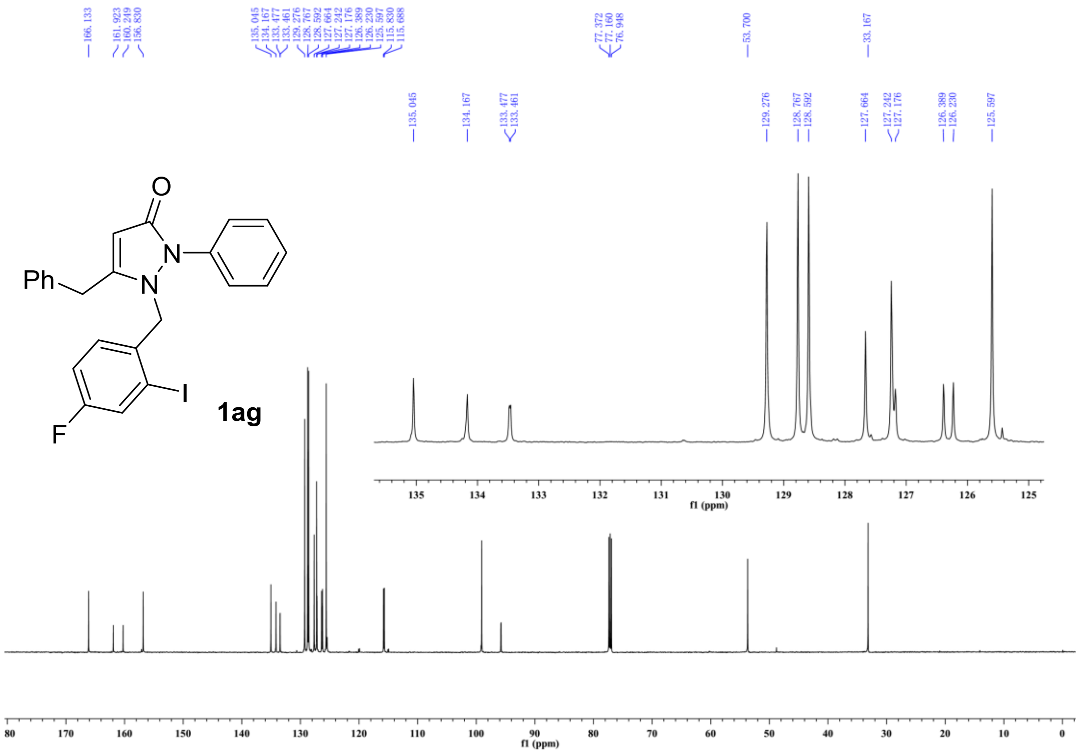

Figure S217. ^{13}C NMR of 1ag, related to Table 2.

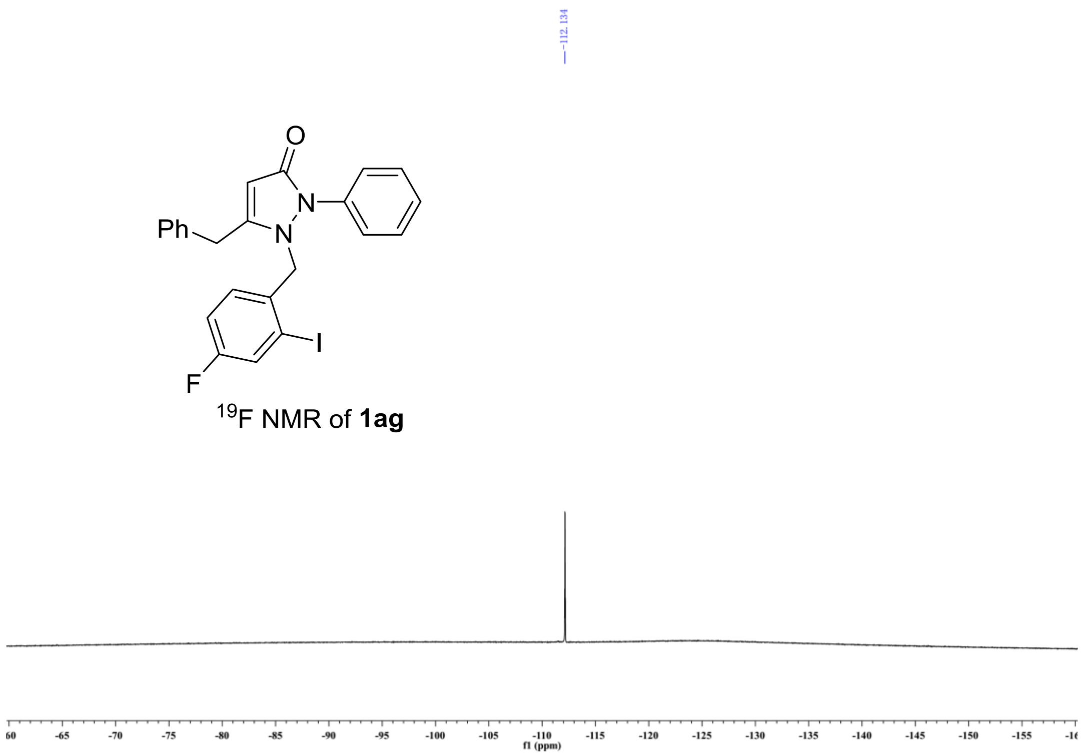

Figure S218. ^{19}F NMR of 1ag, related to Table 2.

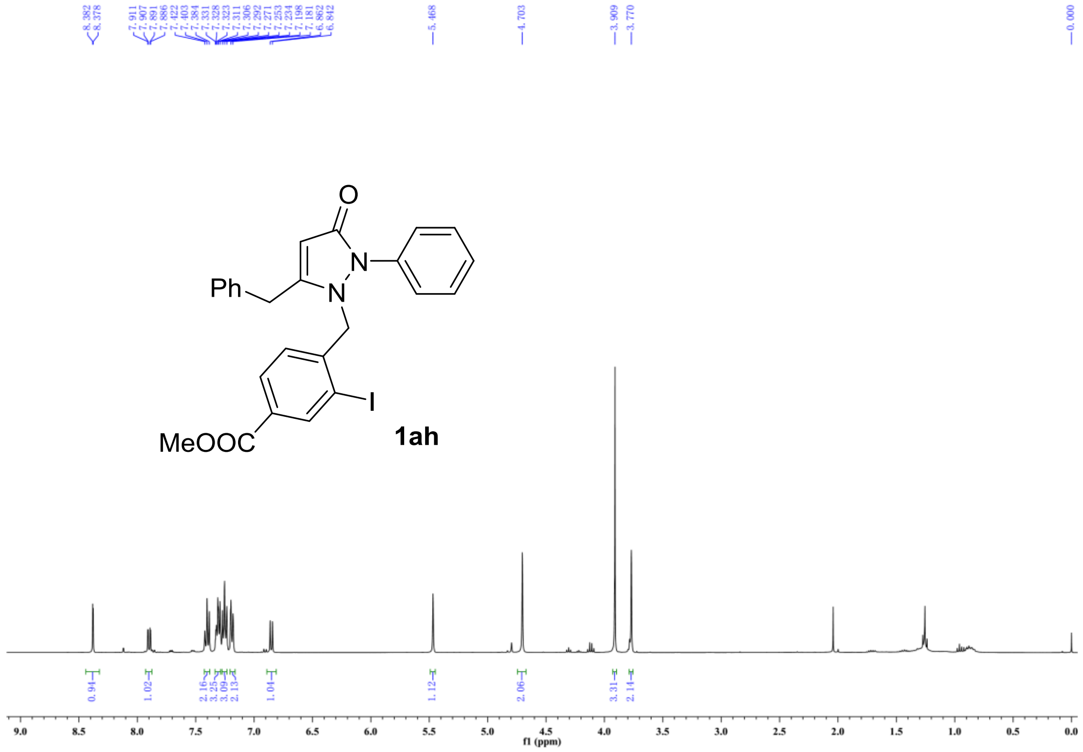

Figure S219. ^{1}H NMR of 1ah, related to Table 2.

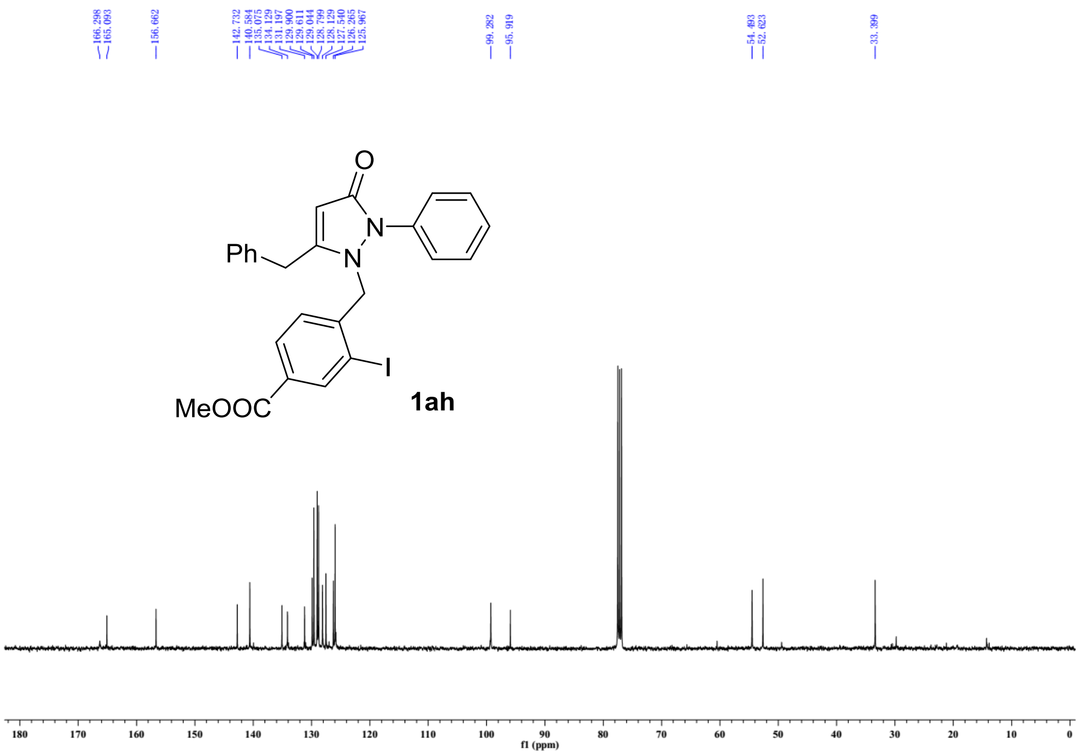

Figure S220. ^{13}C NMR of 1ah, related to Table 2.

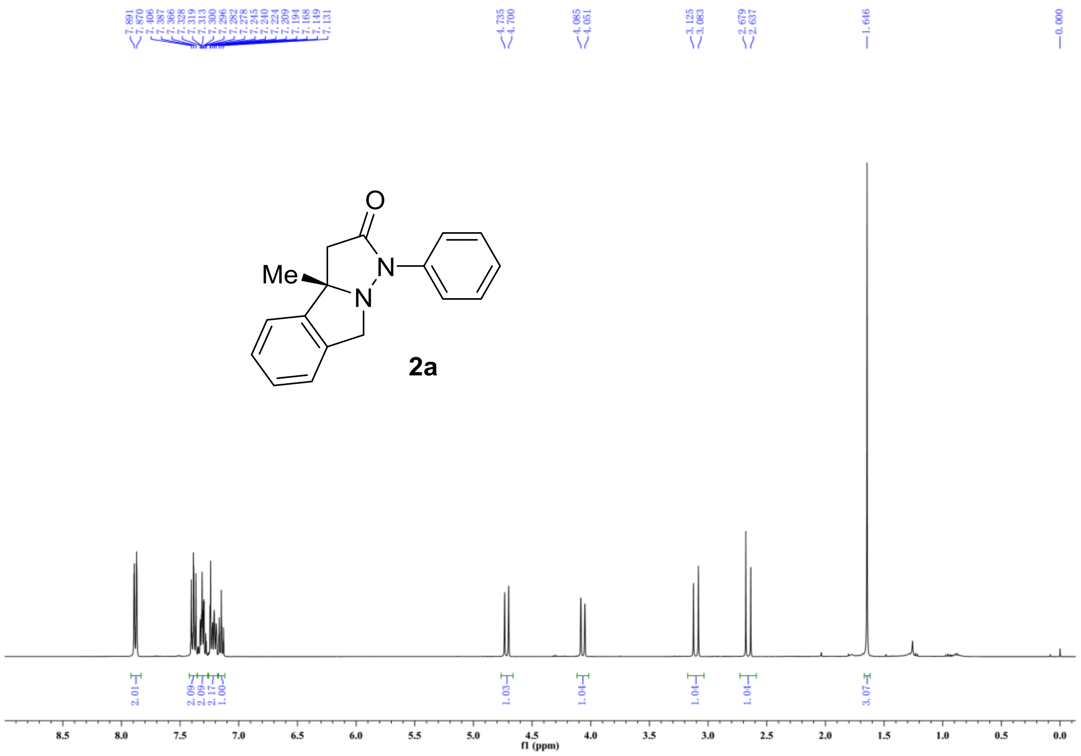

Figure S221. [1]**H NMR of 2a, related to Table 2.**

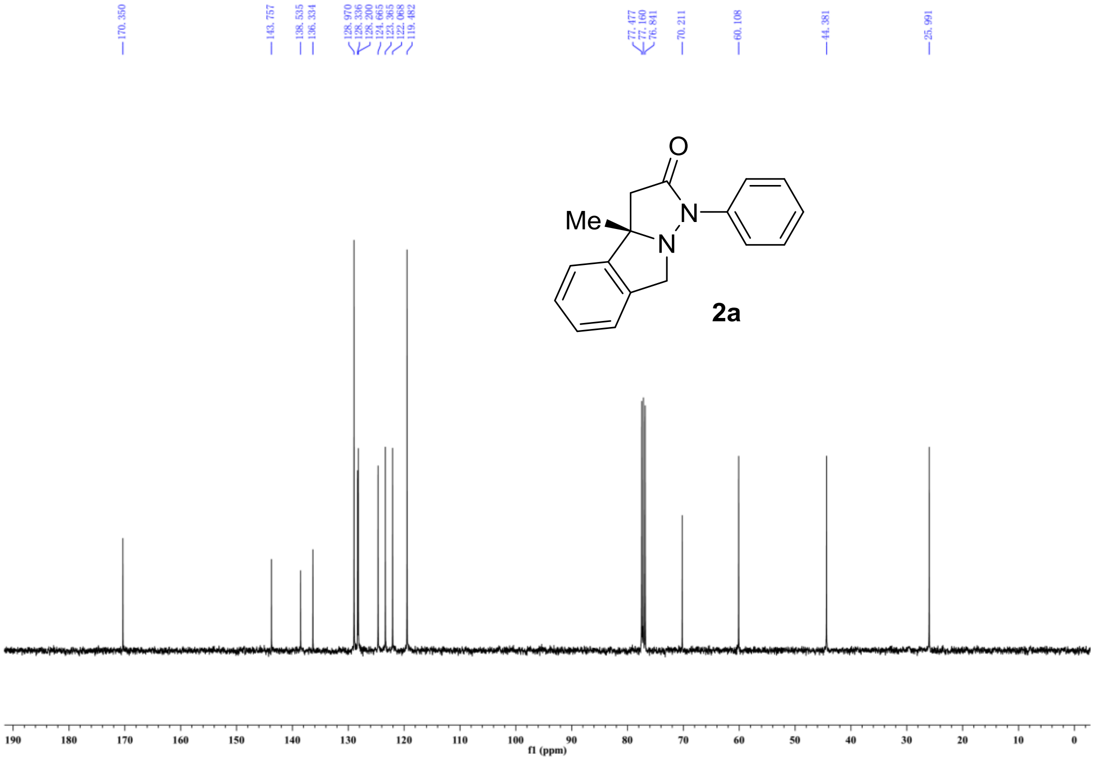

Figure S222. [13]**C NMR of 2a, related to Table 2.**

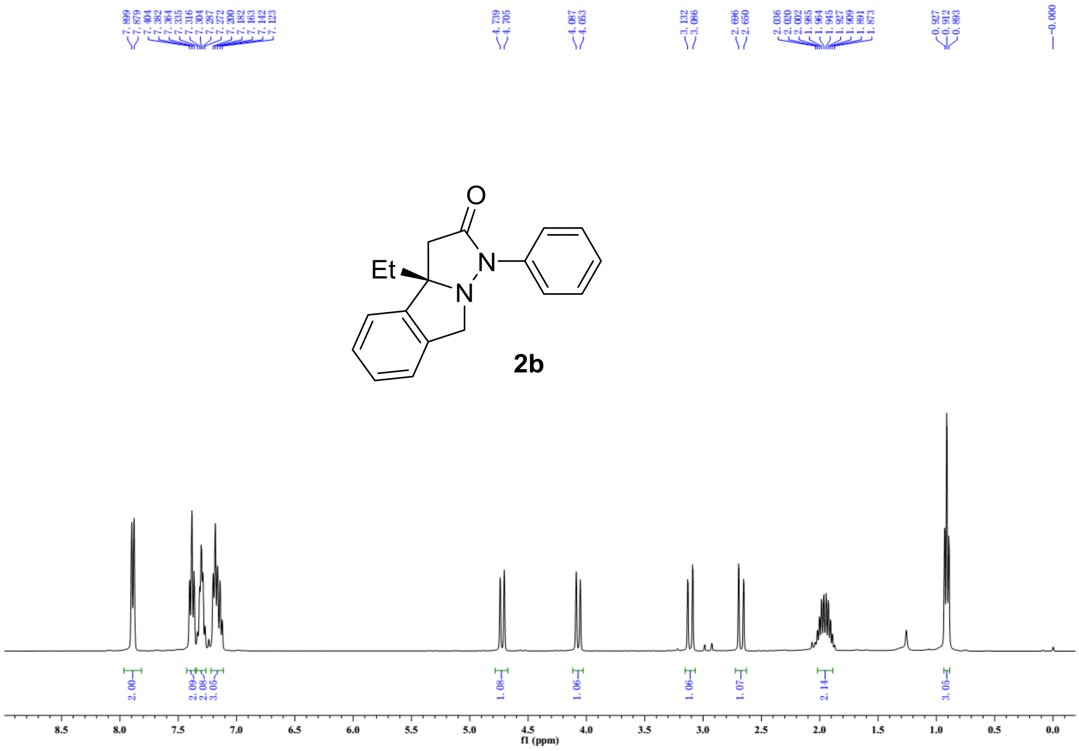

Figure S223. ^{1}H **NMR of 2b, related to Table 2.**

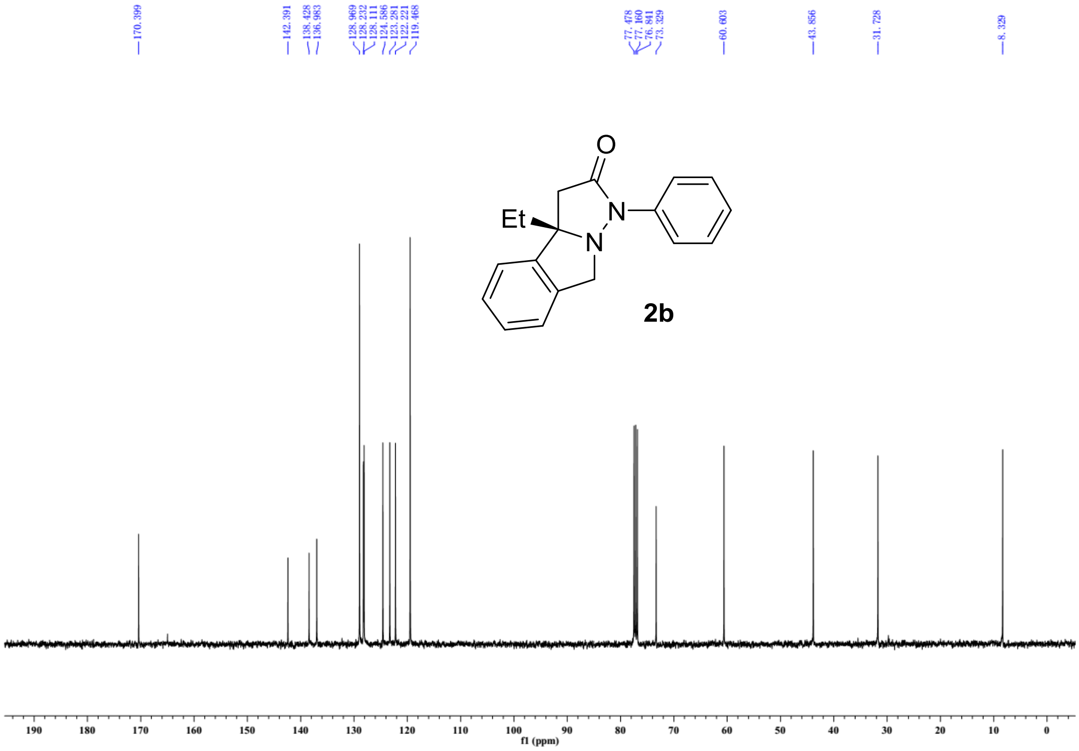

Figure S224. ^{13}C **NMR of 2b, related to Table 2.**

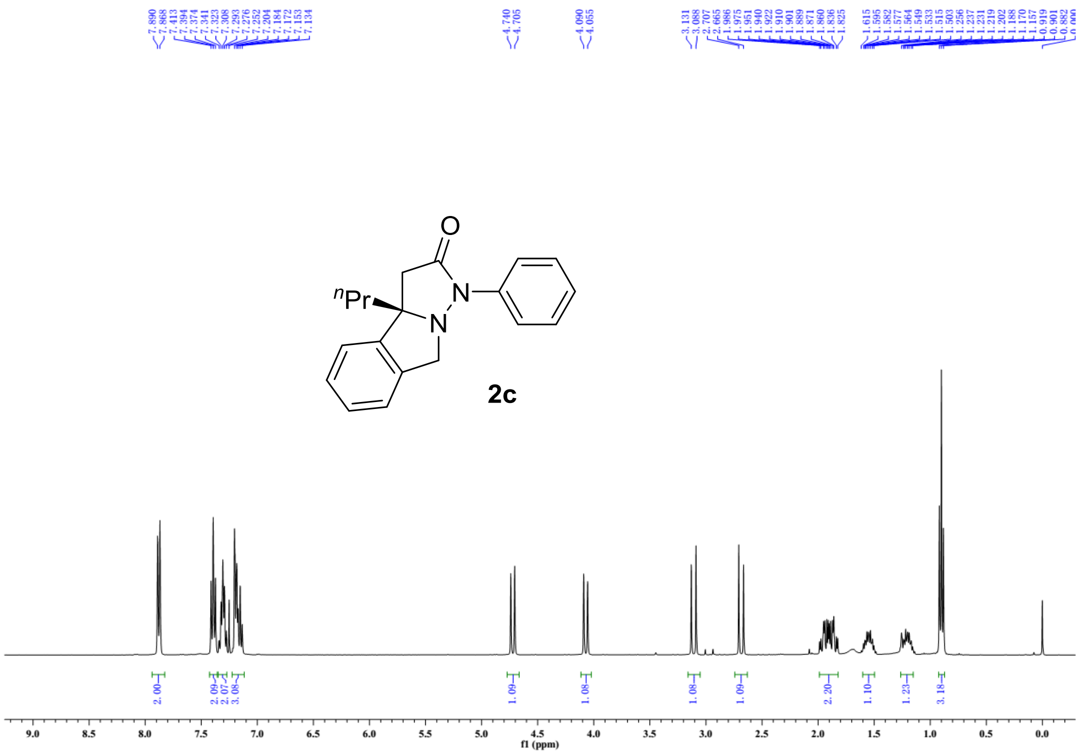

Figure S225. ^{1}H NMR of 2c, related to Table 2.

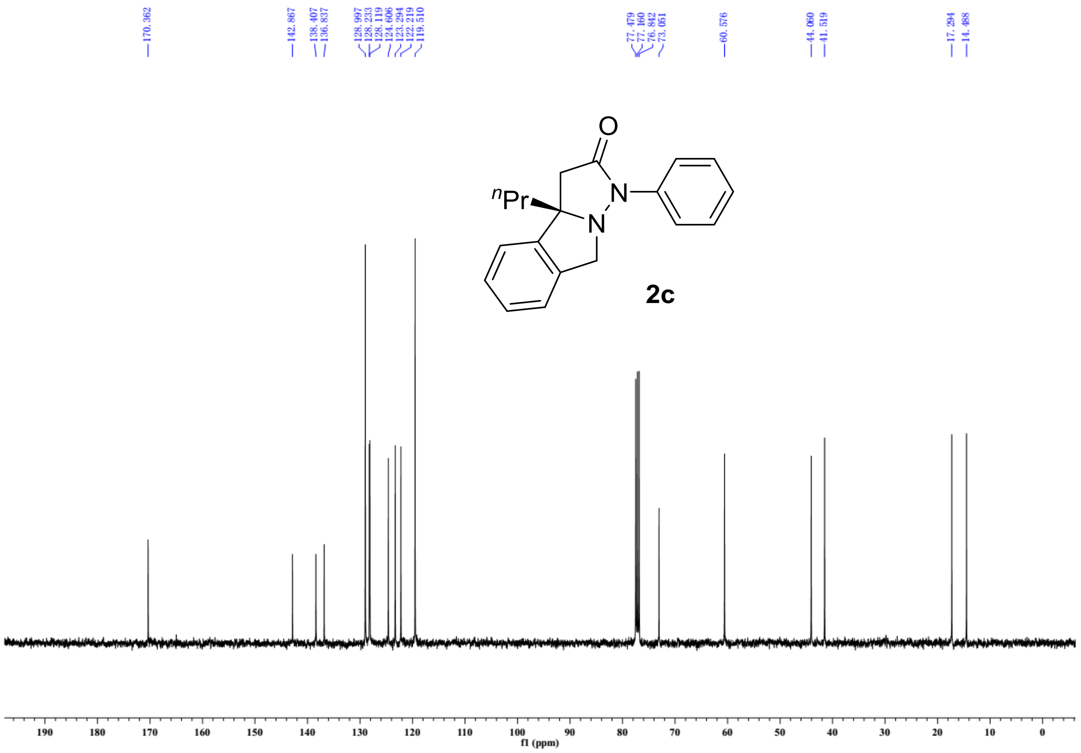

Figure S226. ^{13}C NMR of 2c, related to Table 2.

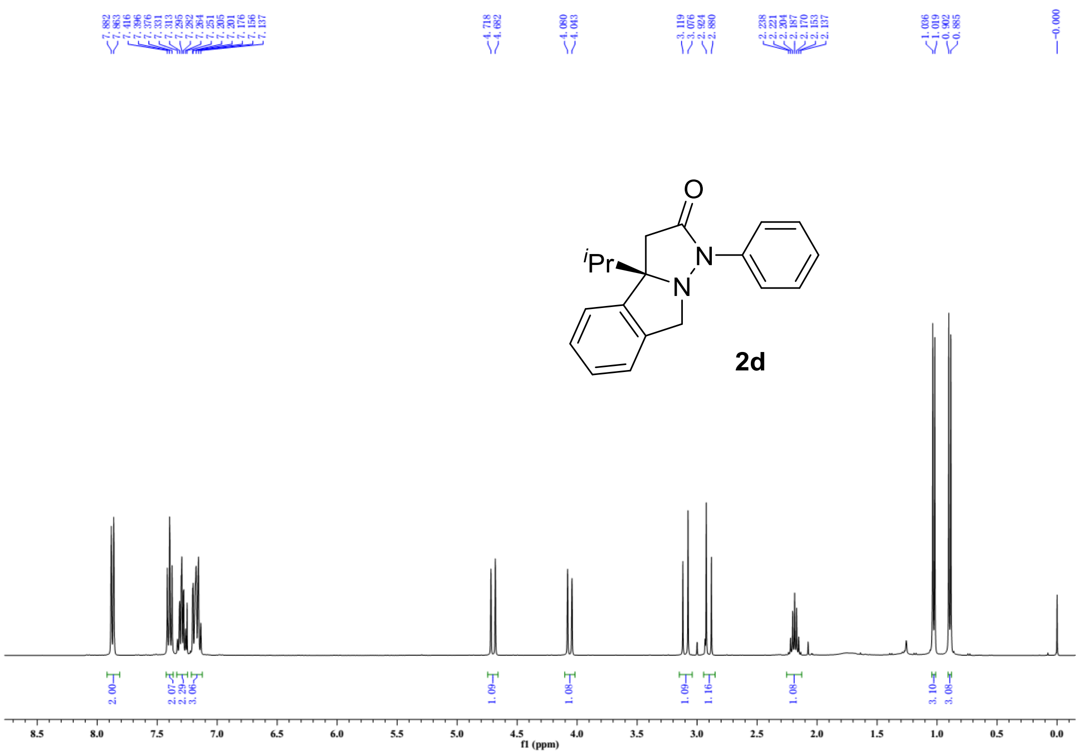

Figure S227. ^{1}**H NMR of 2d, related to Table 2.**

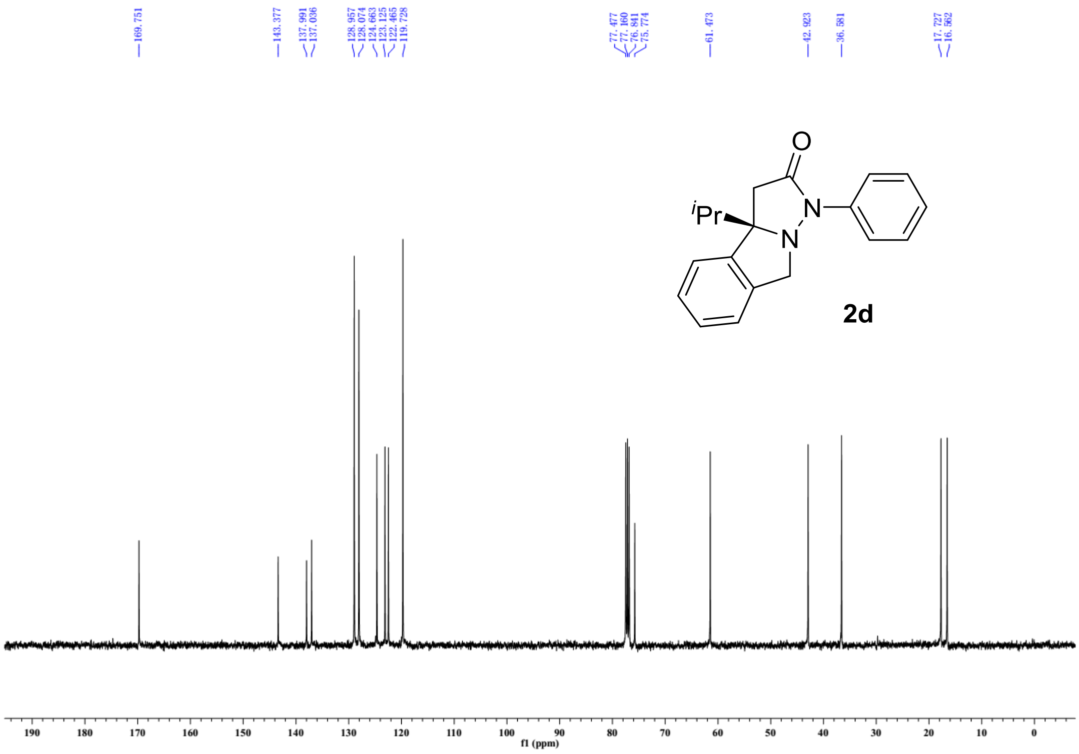

Figure S228. ^{13}**C NMR of 2d, related to Table 2.**

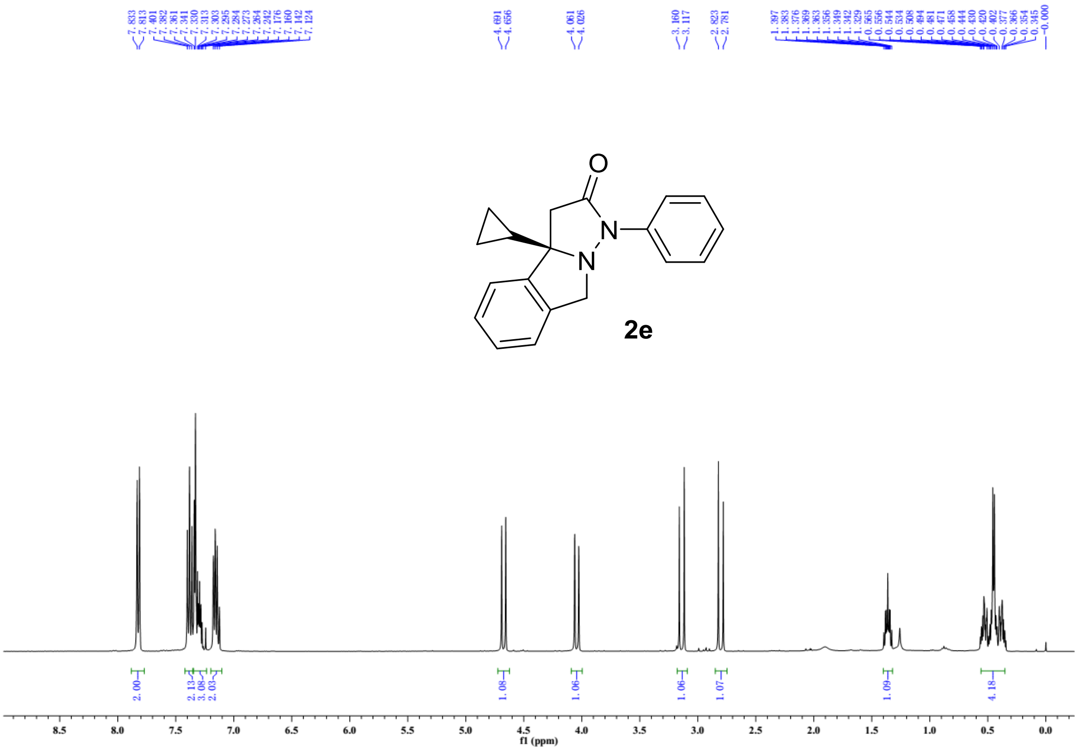

Figure S229. ^{1}H NMR of 2e, related to Table 2.

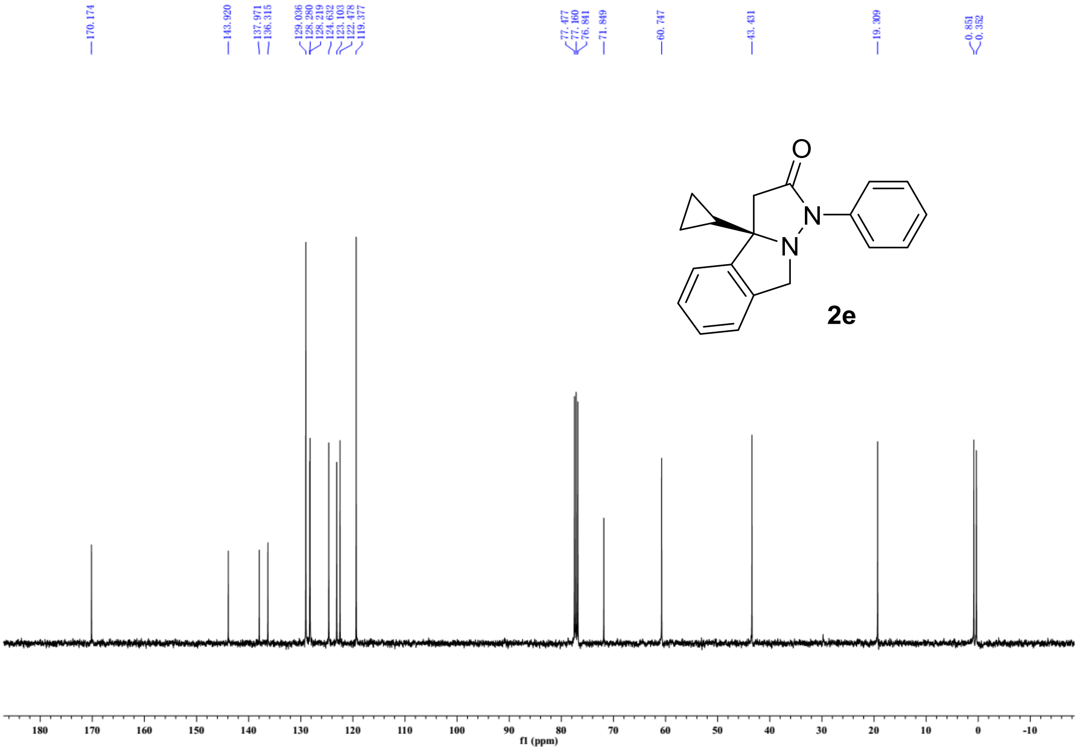

Figure S230. ^{13}C NMR of 2e, related to Table 2.

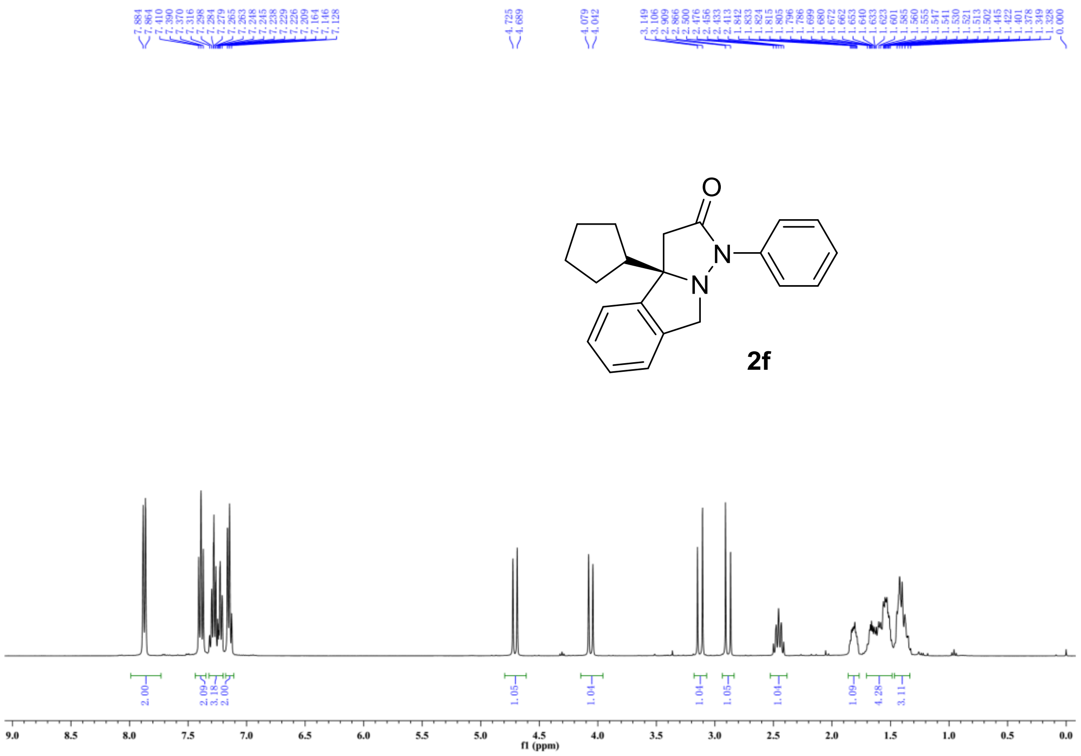

Figure S231. **^{1}H NMR of 2f, related to Table 2.**

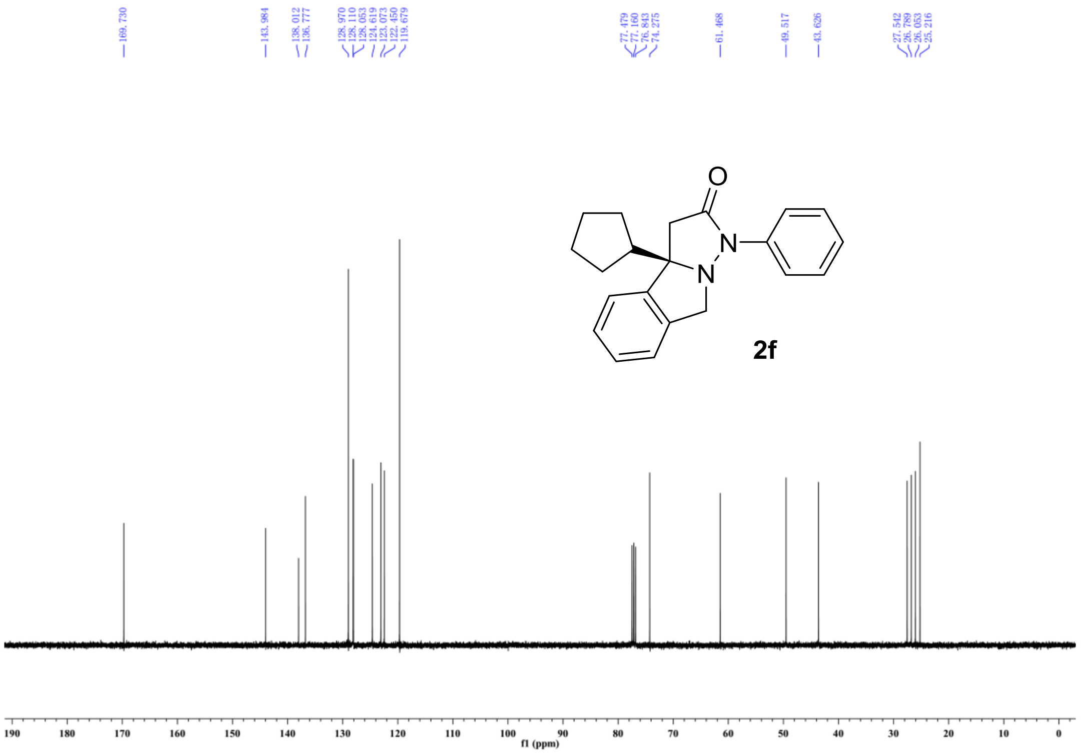

Figure S232. **^{13}C NMR of 2f, related to Table 2.**

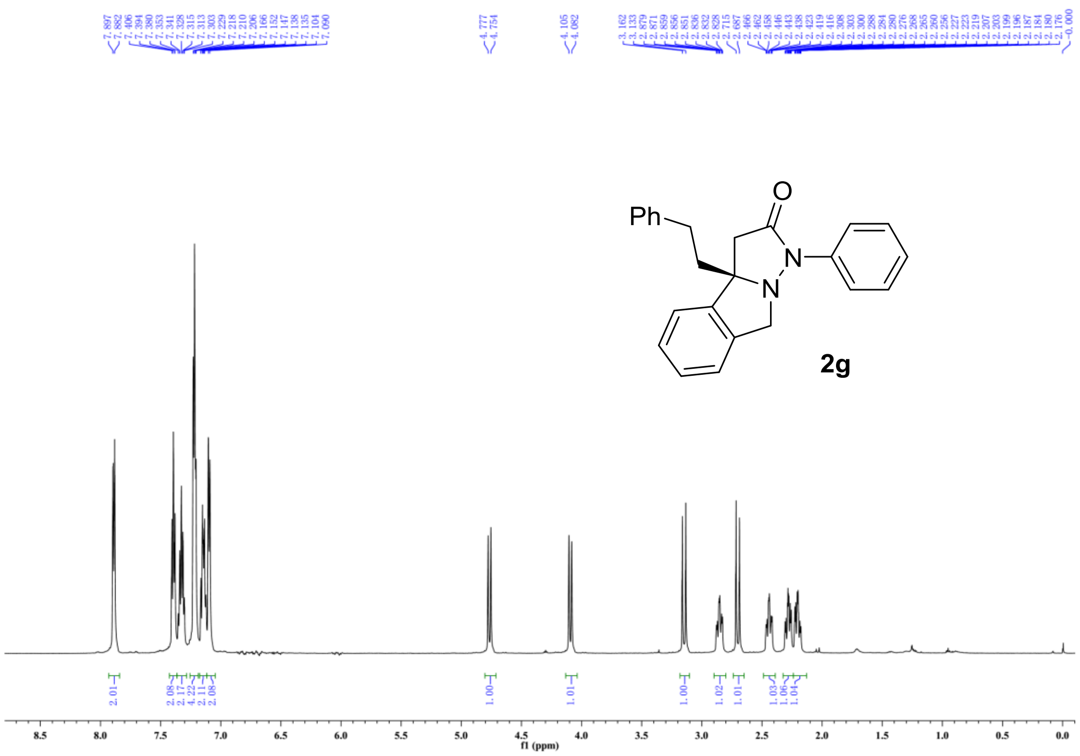

Figure S233. ¹H NMR of 2g, related to Table 2.

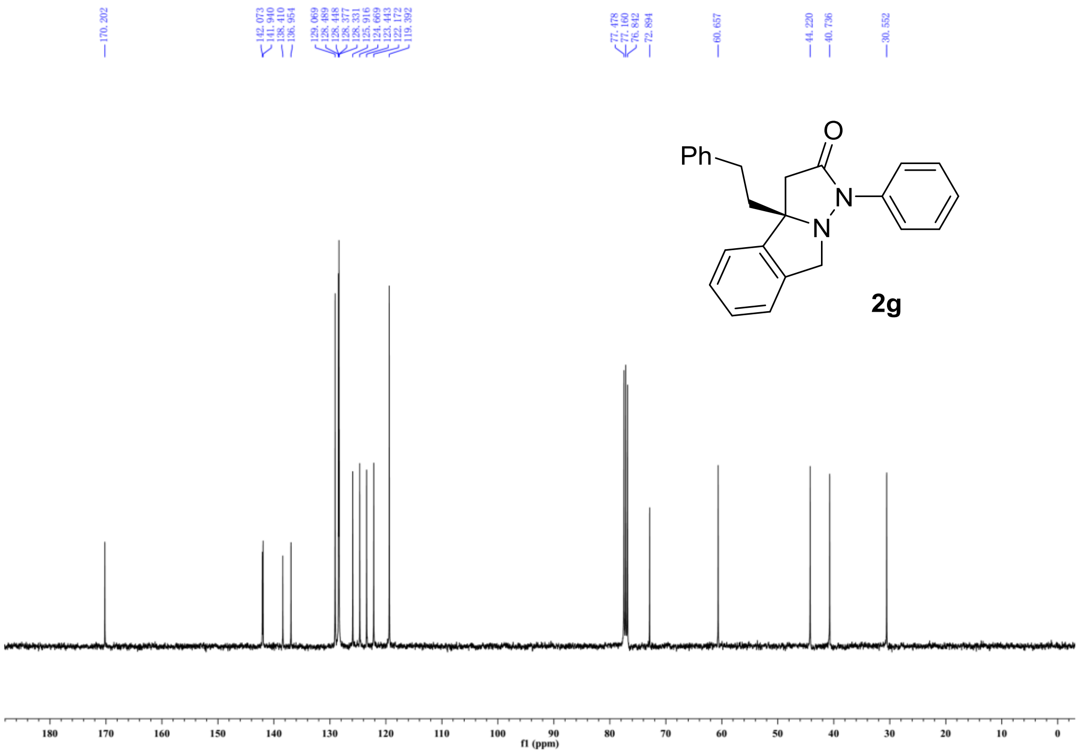

Figure S234. ¹³C NMR of 2g, related to Table 2.

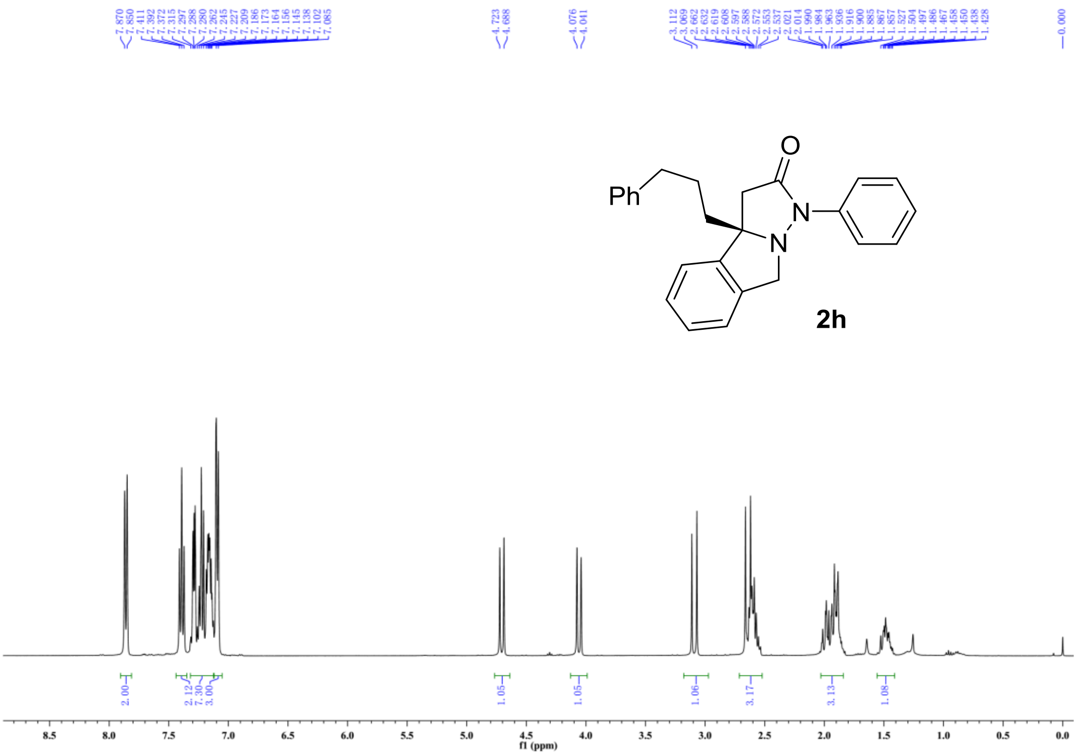

Figure S235. ^{1}H NMR of 2h, related to Table 2.

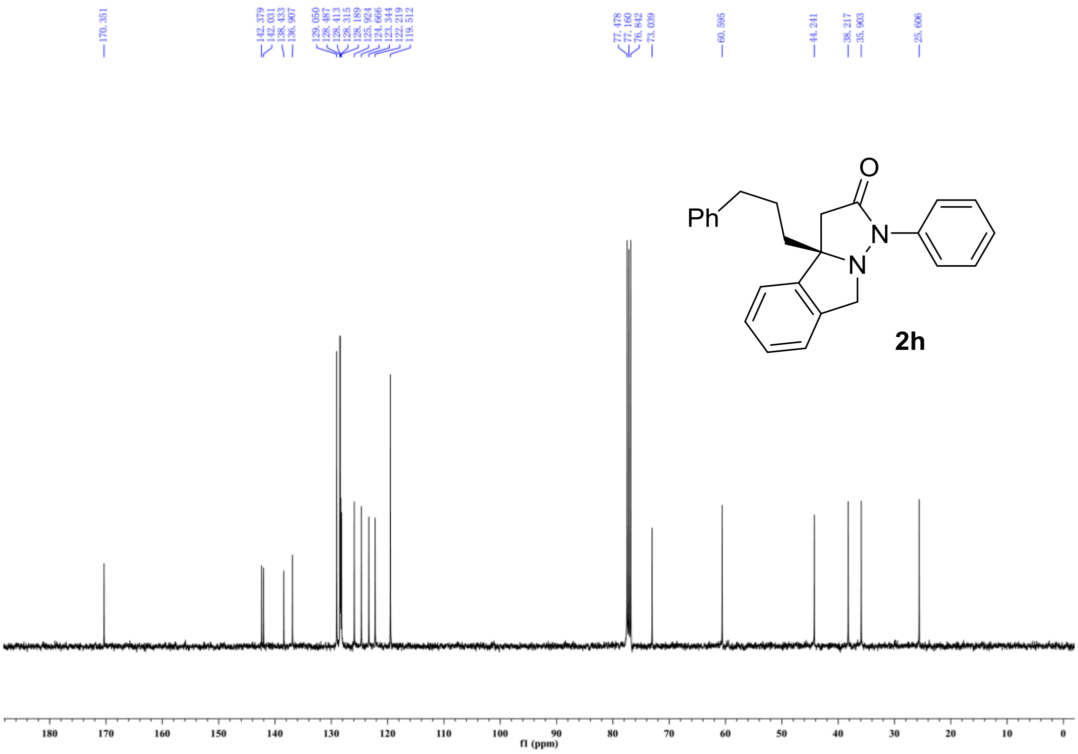

Figure S236. ^{13}C NMR of 2h, related to Table 2.

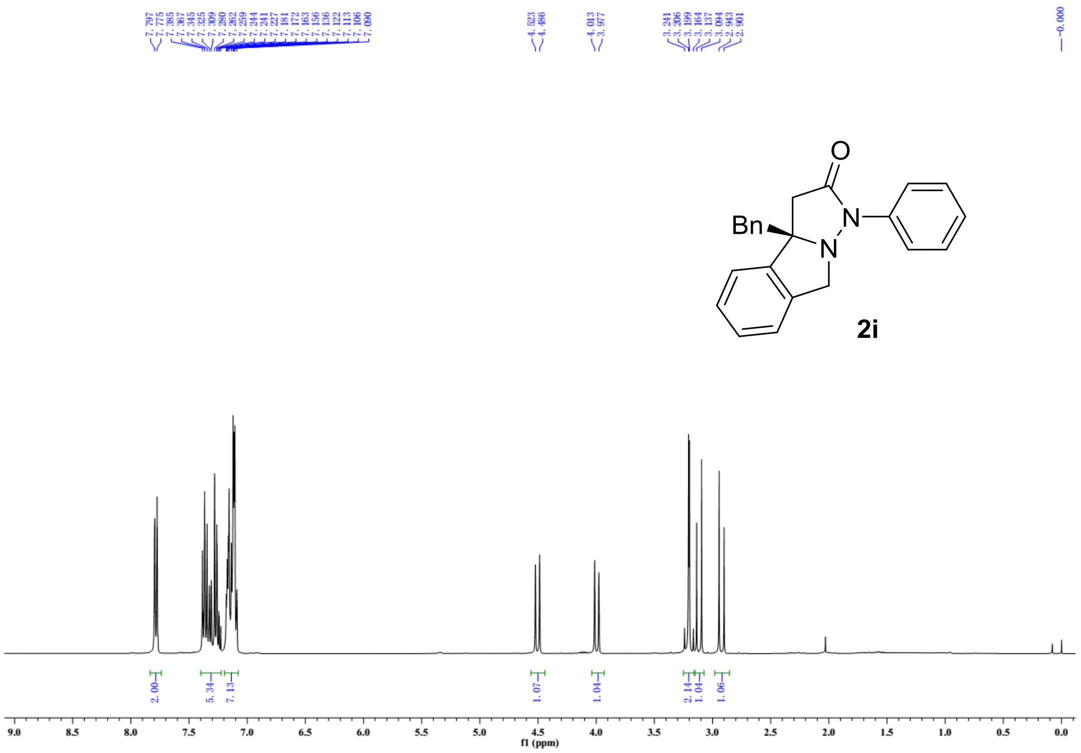

Figure S237. ^{1}H NMR of 2i, related to Table 2.

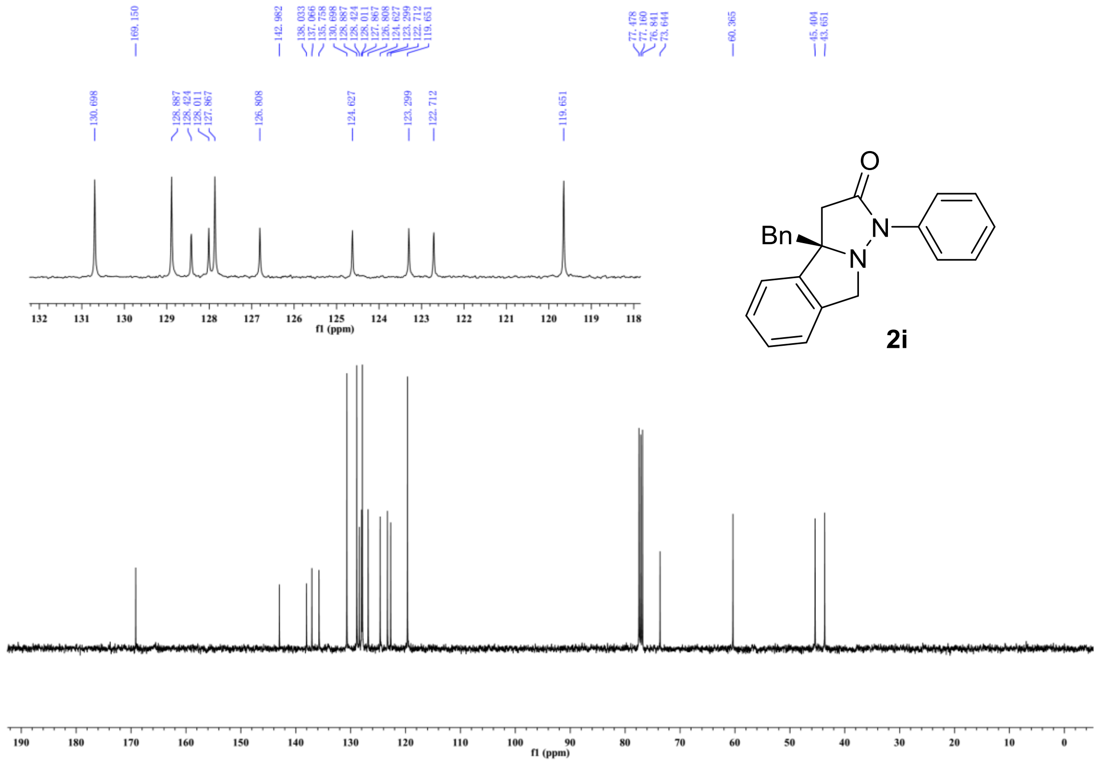

Figure S238. ^{13}C NMR of 2i, related to Table 2.

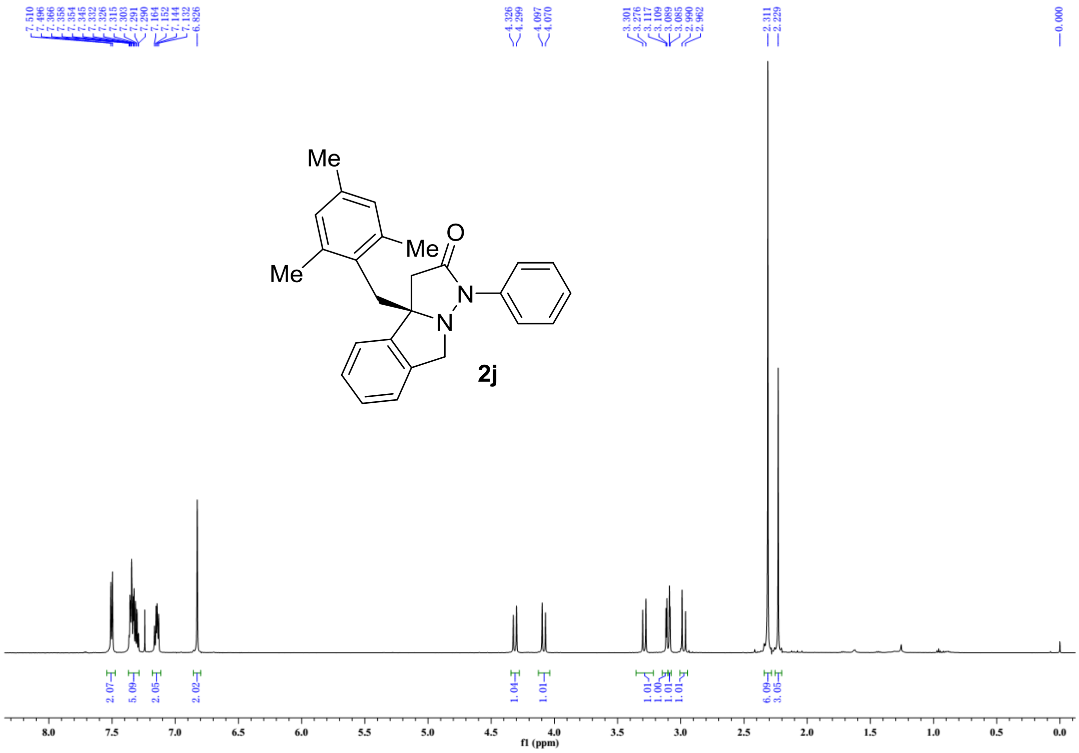

Figure S239. **^{1}H NMR of 2j, related to Table 2.**

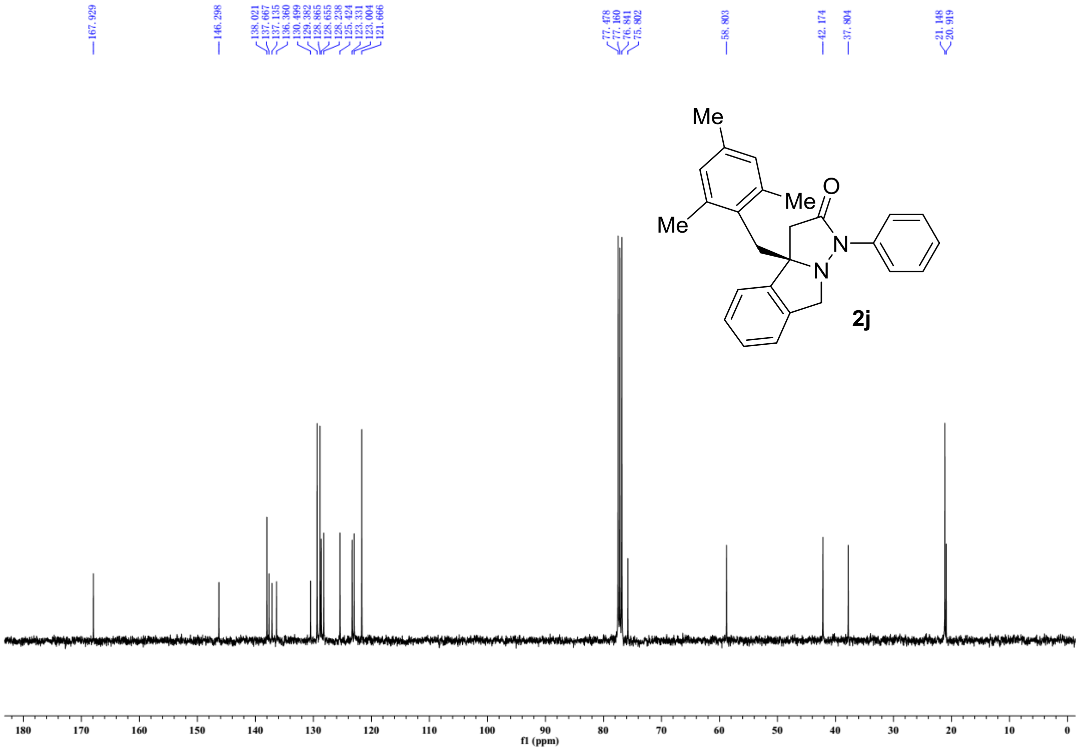

Figure S240. **^{13}C NMR of 2j, related to Table 2.**

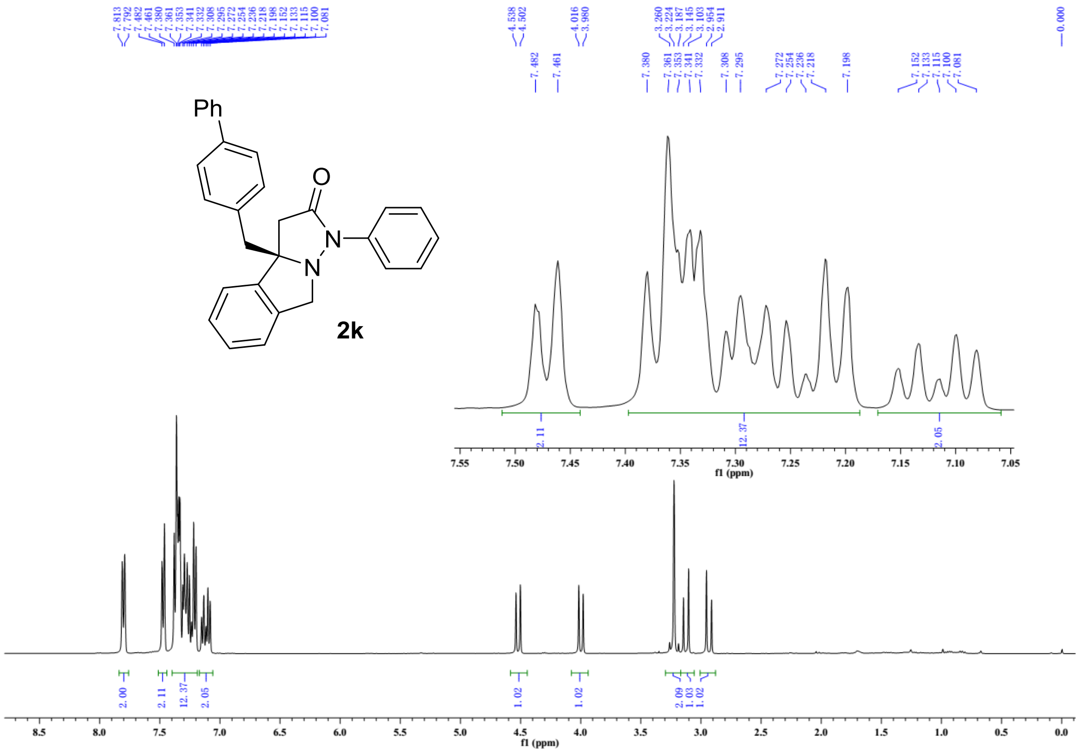

Figure S241. ^{1}H NMR of 2k, related to Table 2.

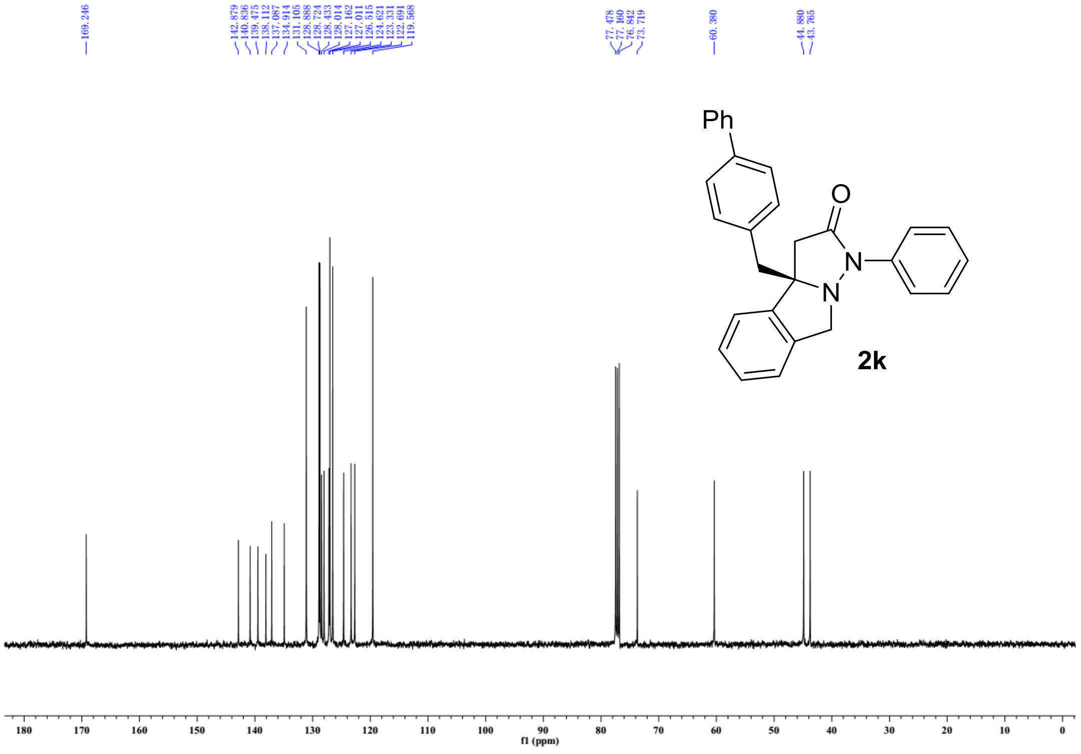

Figure S242. ^{13}C NMR of 2k, related to Table 2.

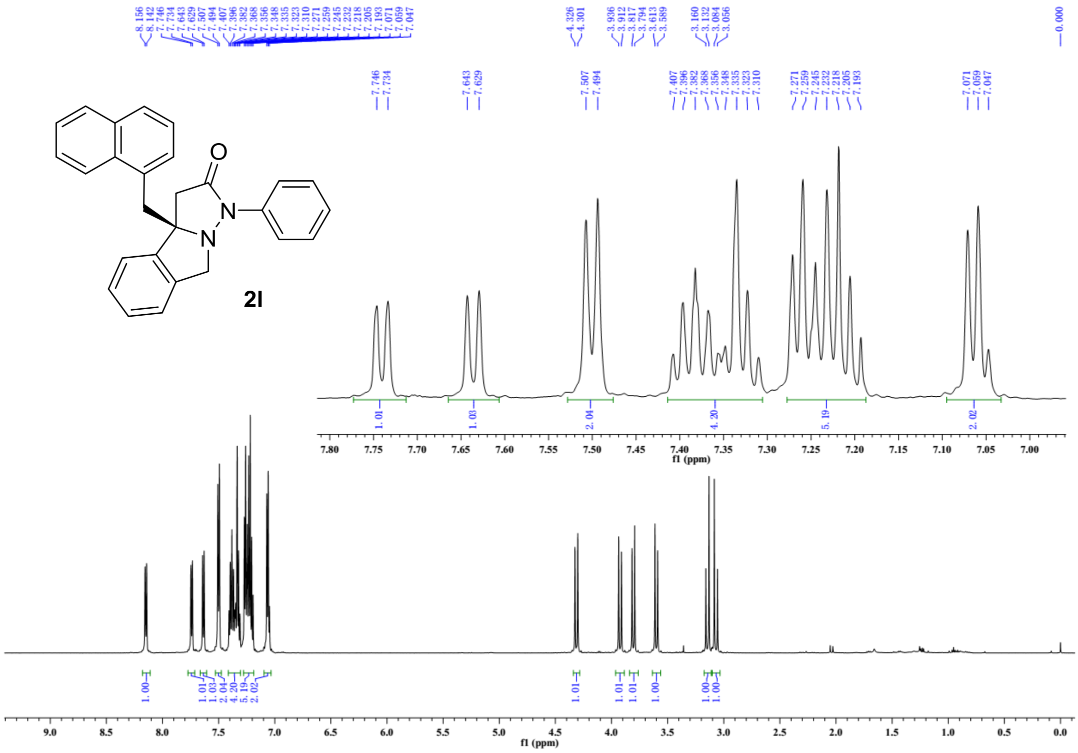

Figure S243. ^{1}H NMR of 2l, related to Table 2.

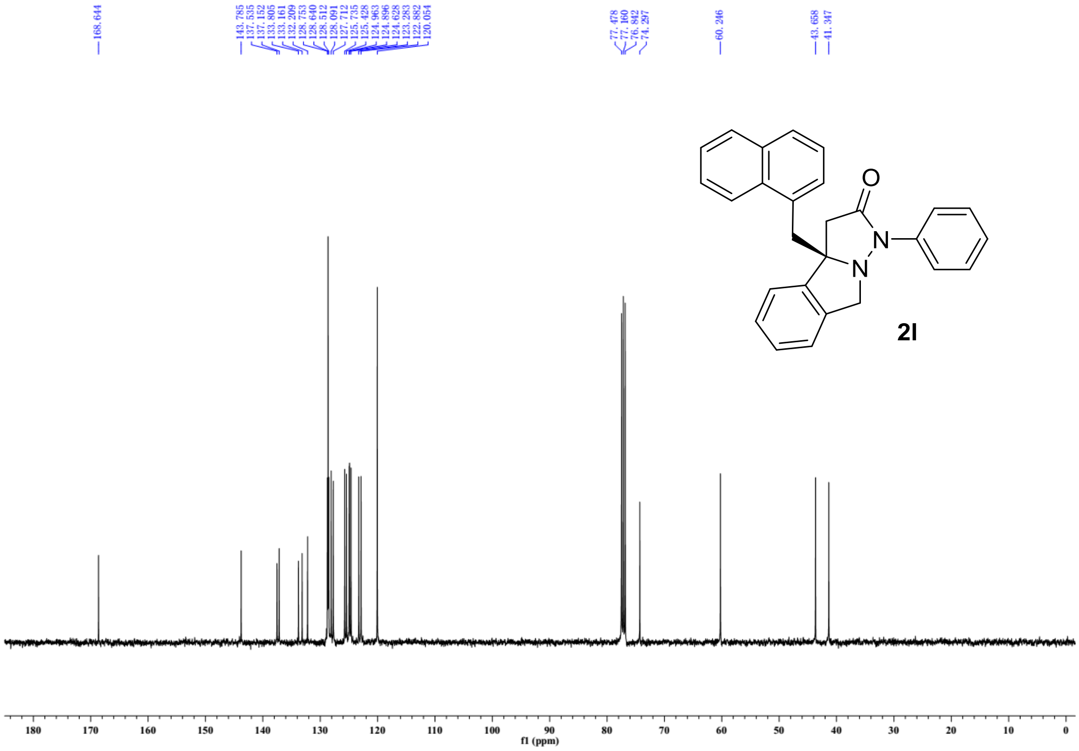

Figure S244. ^{13}C NMR of 2l, related to Table 2.

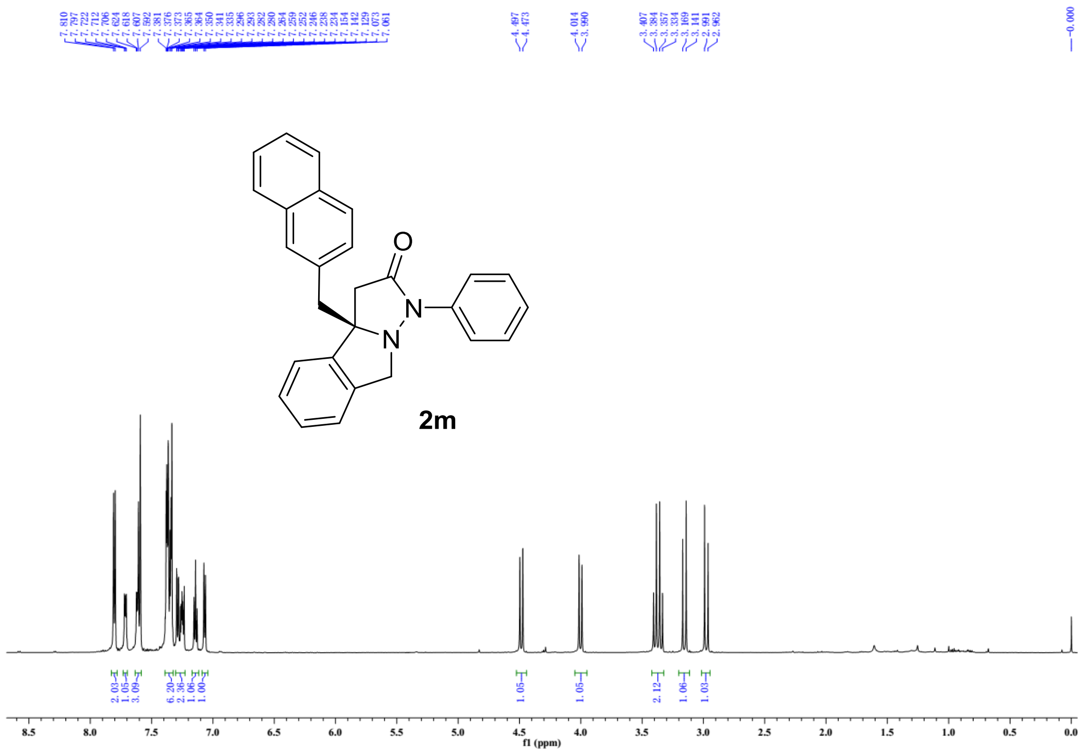

Figure S245. ^{1}H NMR of 2m, related to Table 2.

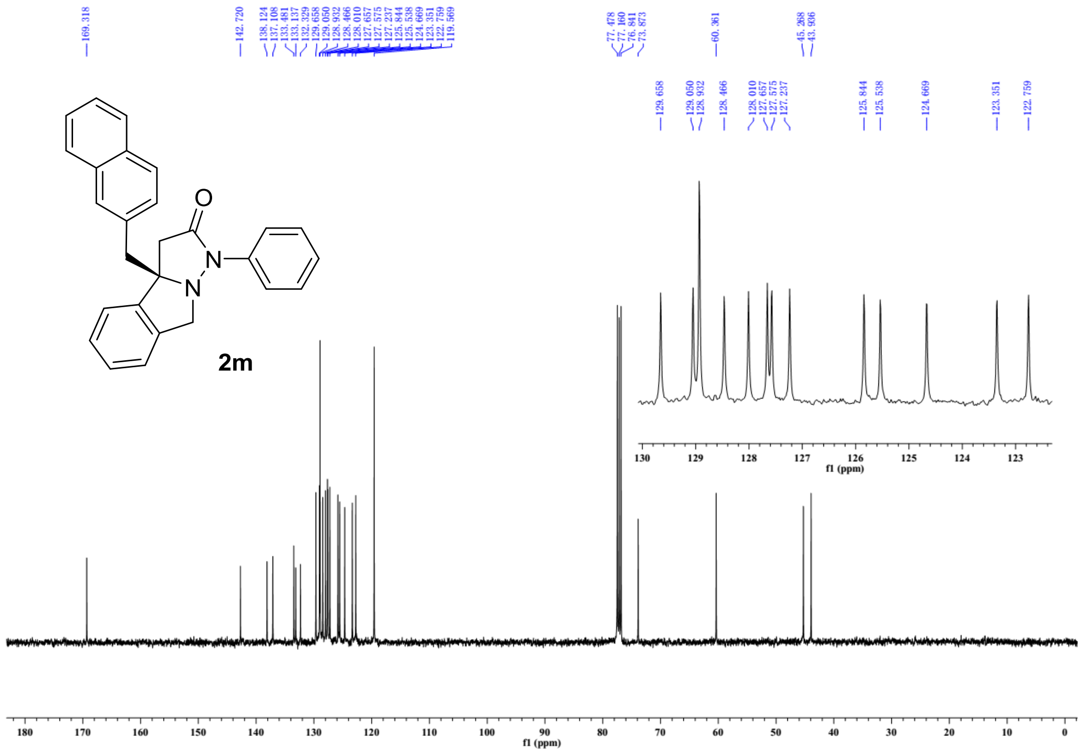

Figure S246. ^{13}C NMR of 2m, related to Table 2.

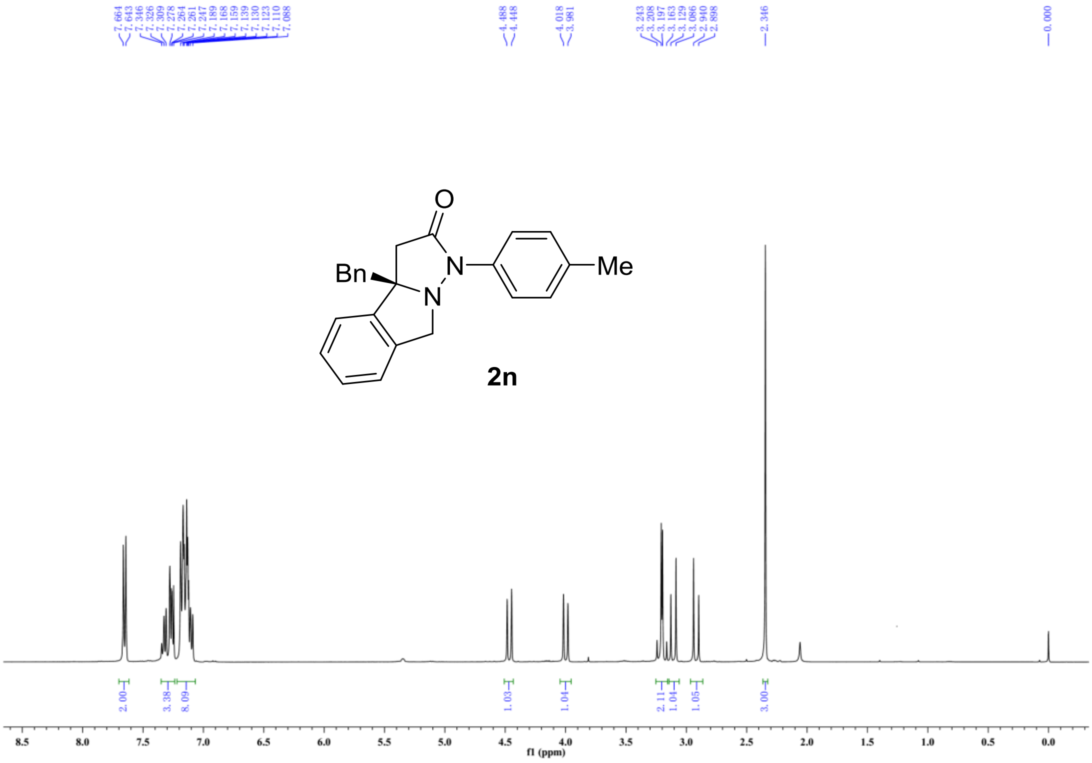

Figure S247. ^{1}H NMR of 2n, related to Table 2.

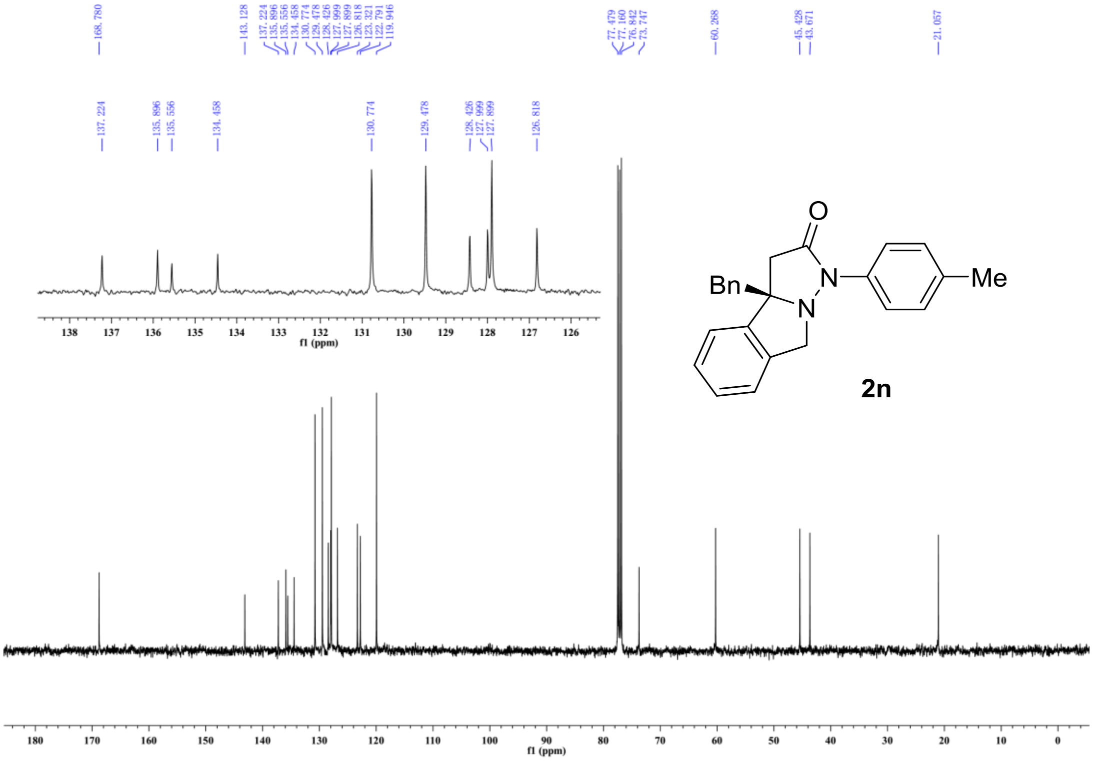

Figure S248. ^{13}C NMR of 2n, related to Table 2.

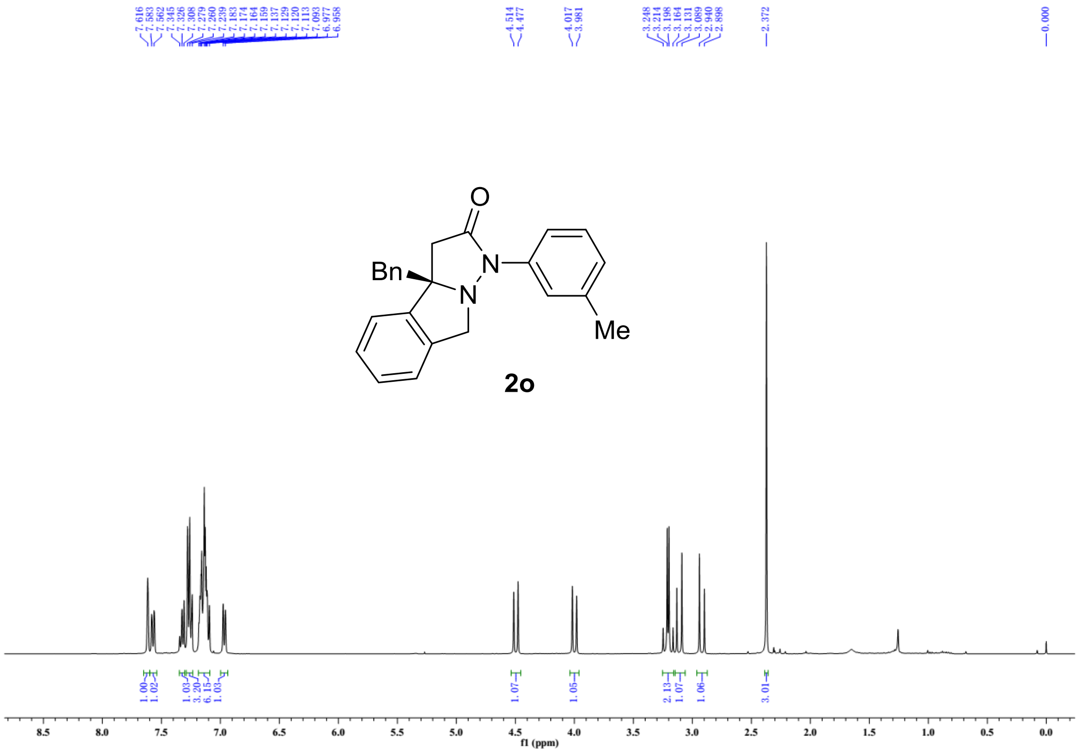

Figure S249. ^{1}H NMR of 2o, related to Table 2.

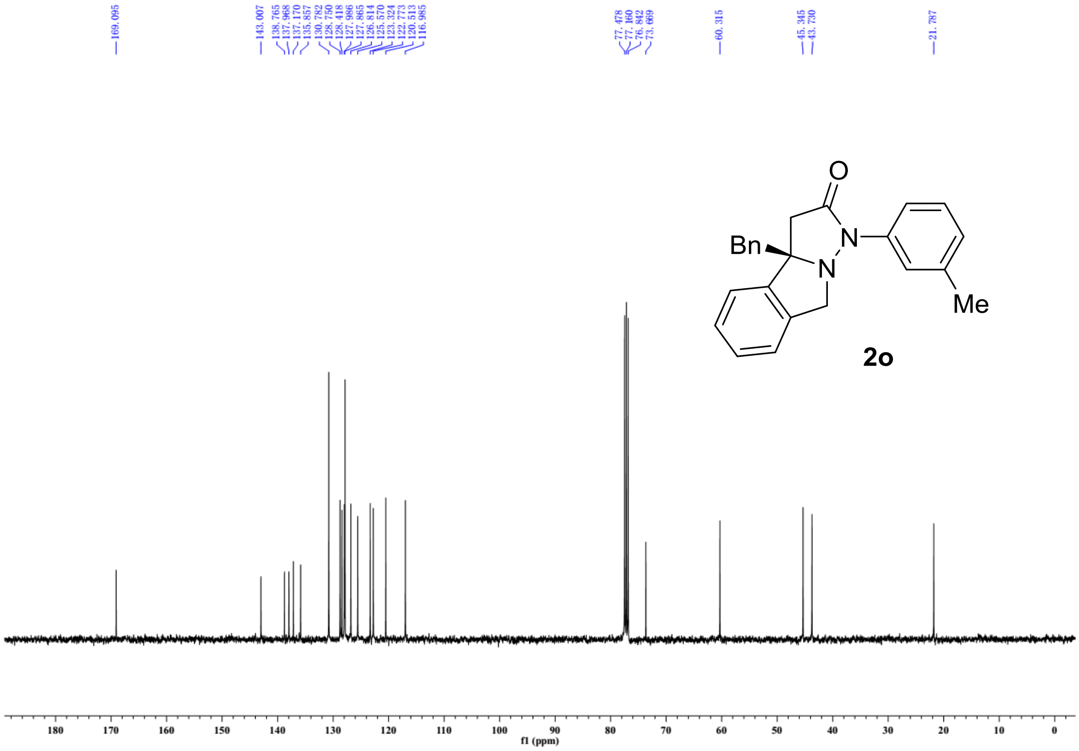

Figure S250. ^{13}C NMR of 2o, related to Table 2.

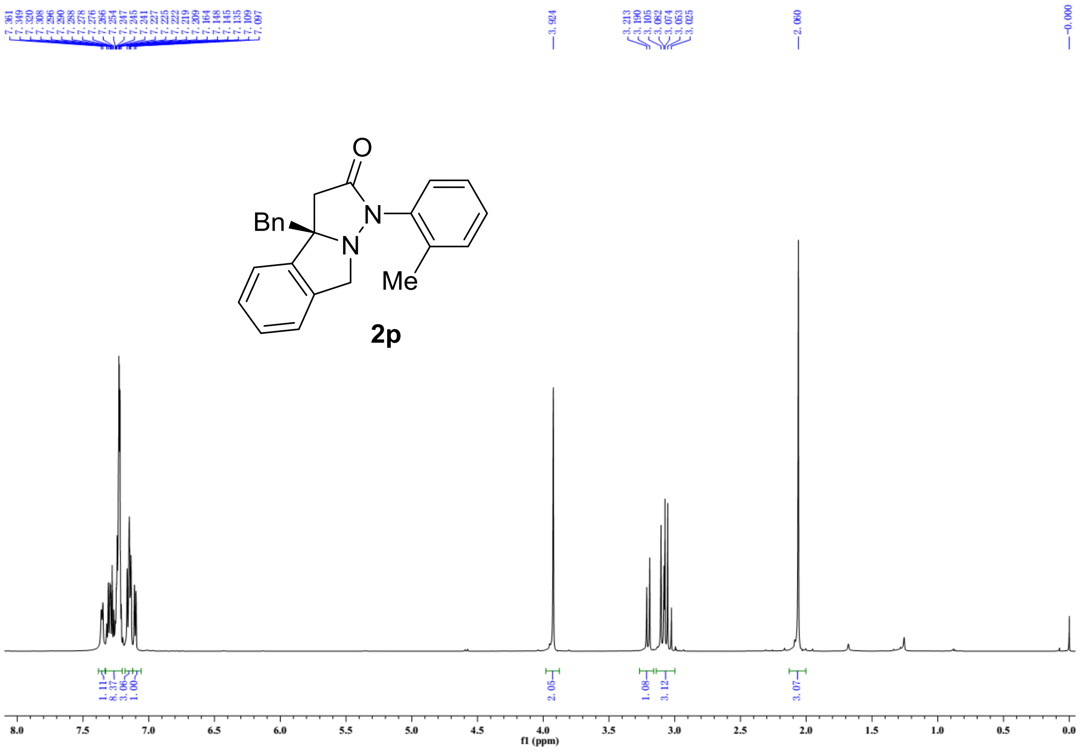

Figure S251. ^{1}**H NMR of 2p, related to Table 2.**

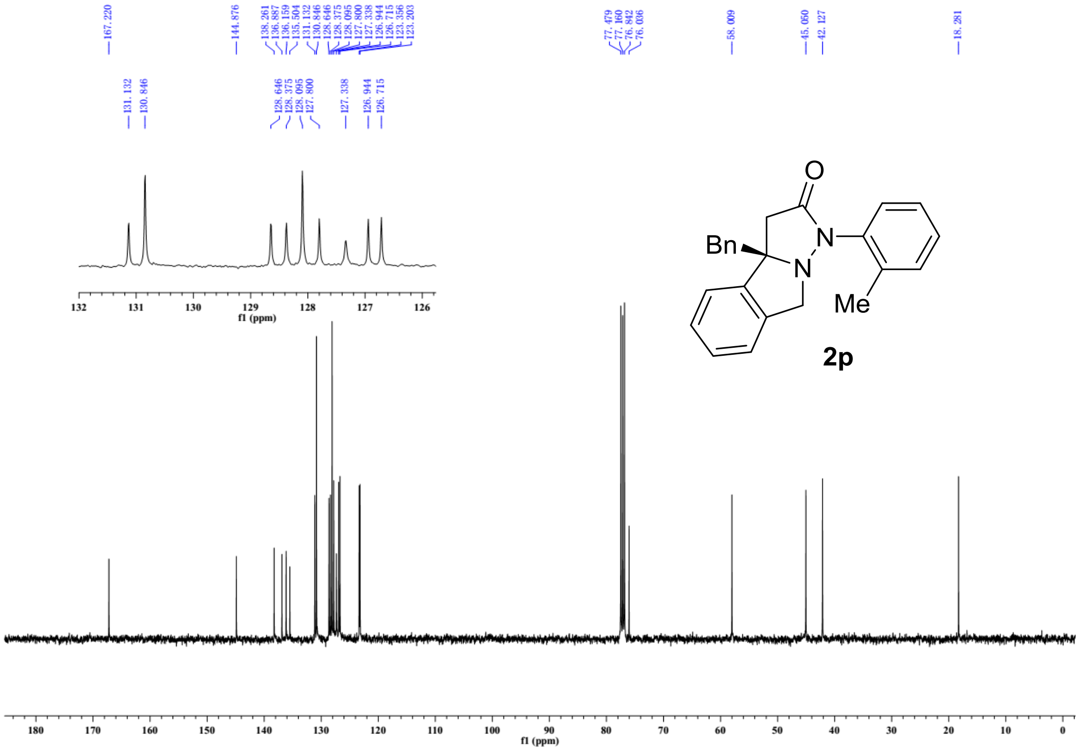

Figure S252. ^{13}**C NMR of 2p, related to Table 2.**

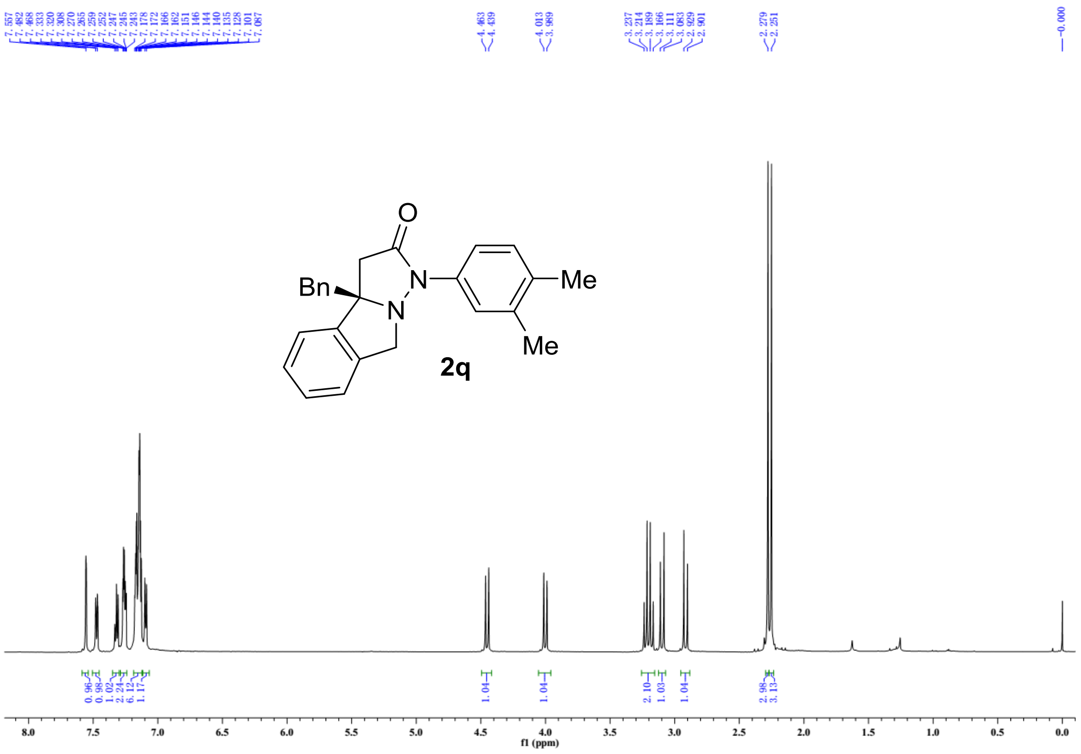

Figure S253. ¹H NMR of 2q, related to Table 2.

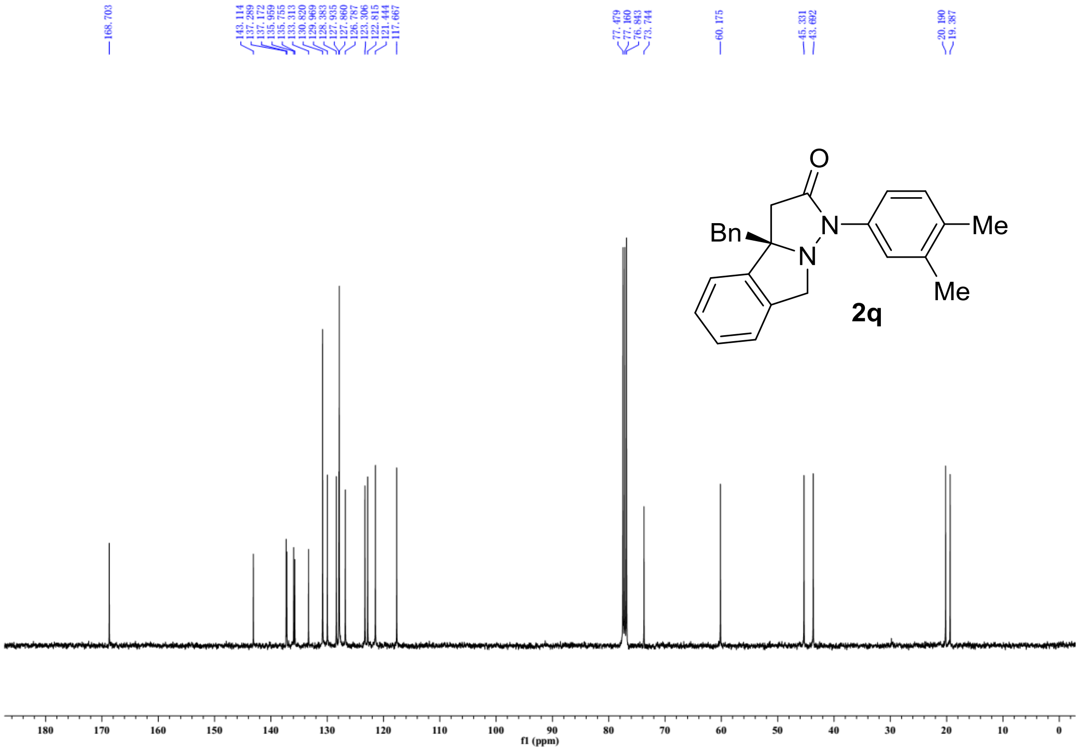

Figure S254. ¹³C NMR of 2q, related to Table 2.

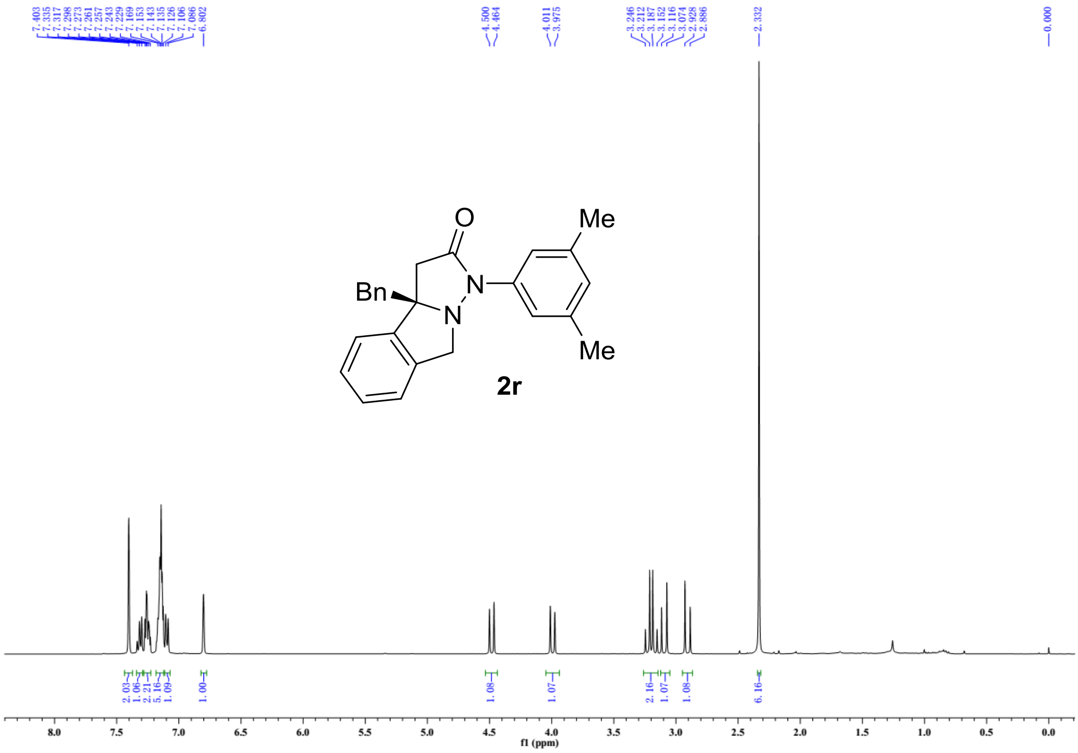

Figure S255. **^{1}H NMR of 2r, related to Table 2.**

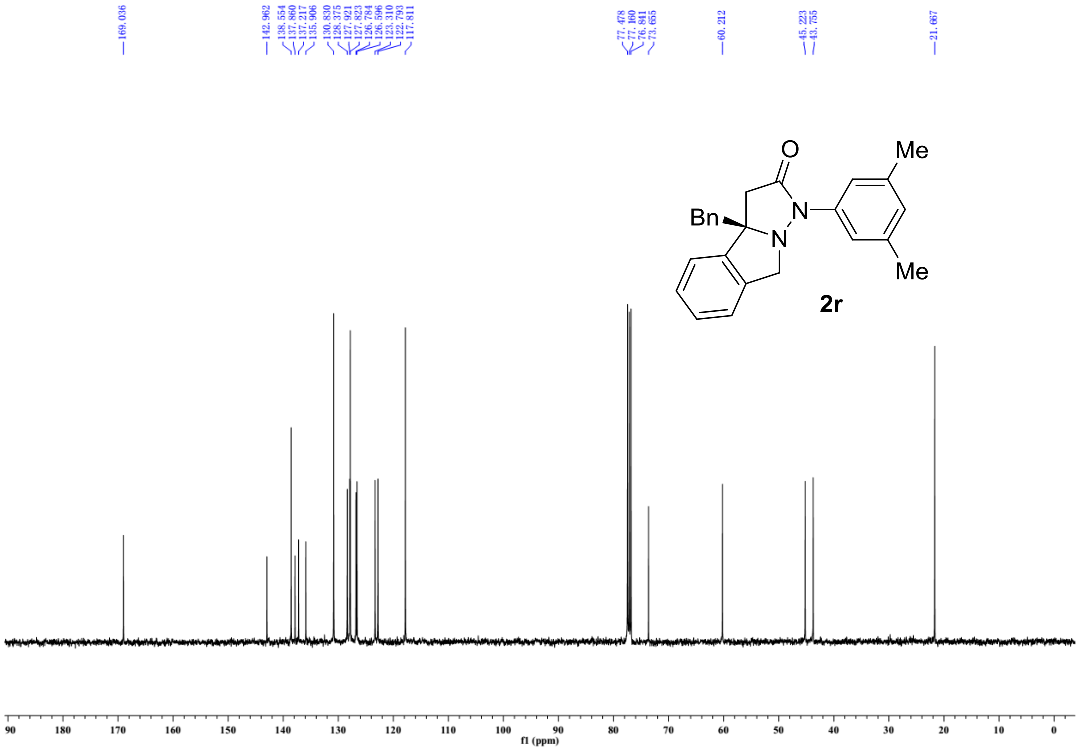

Figure S256. **^{13}C NMR of 2r, related to Table 2.**

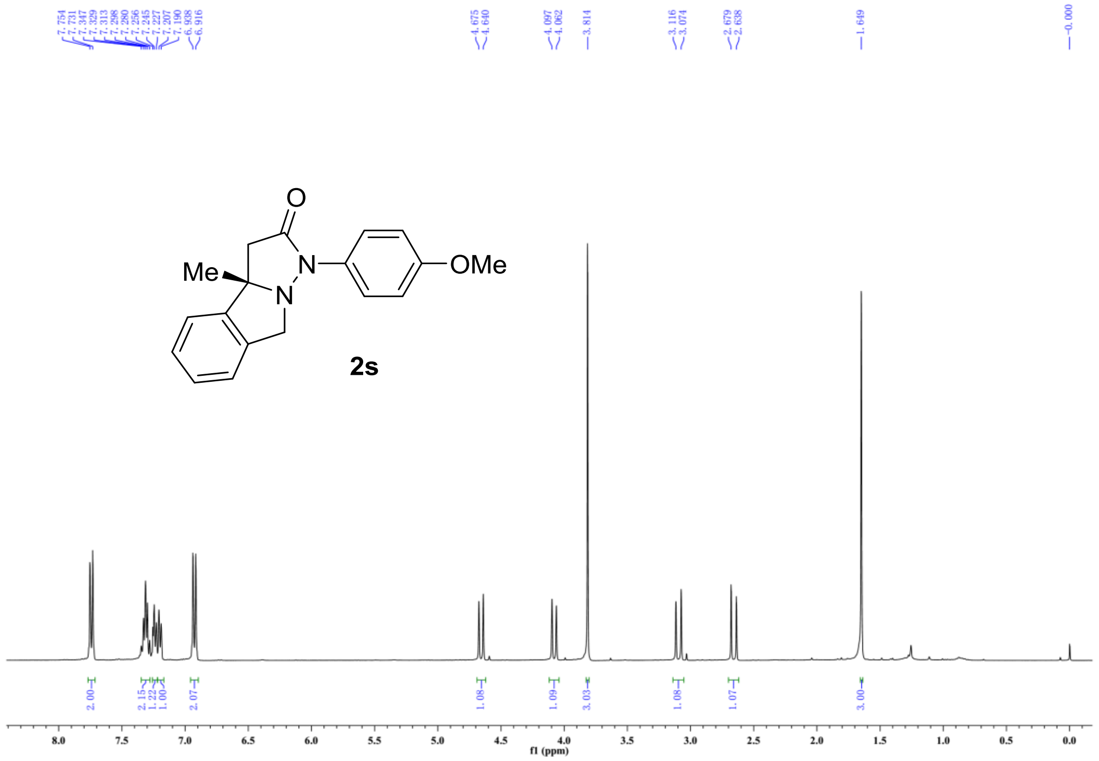

Figure S257. ^{1}H NMR of 2s, related to Table 2.

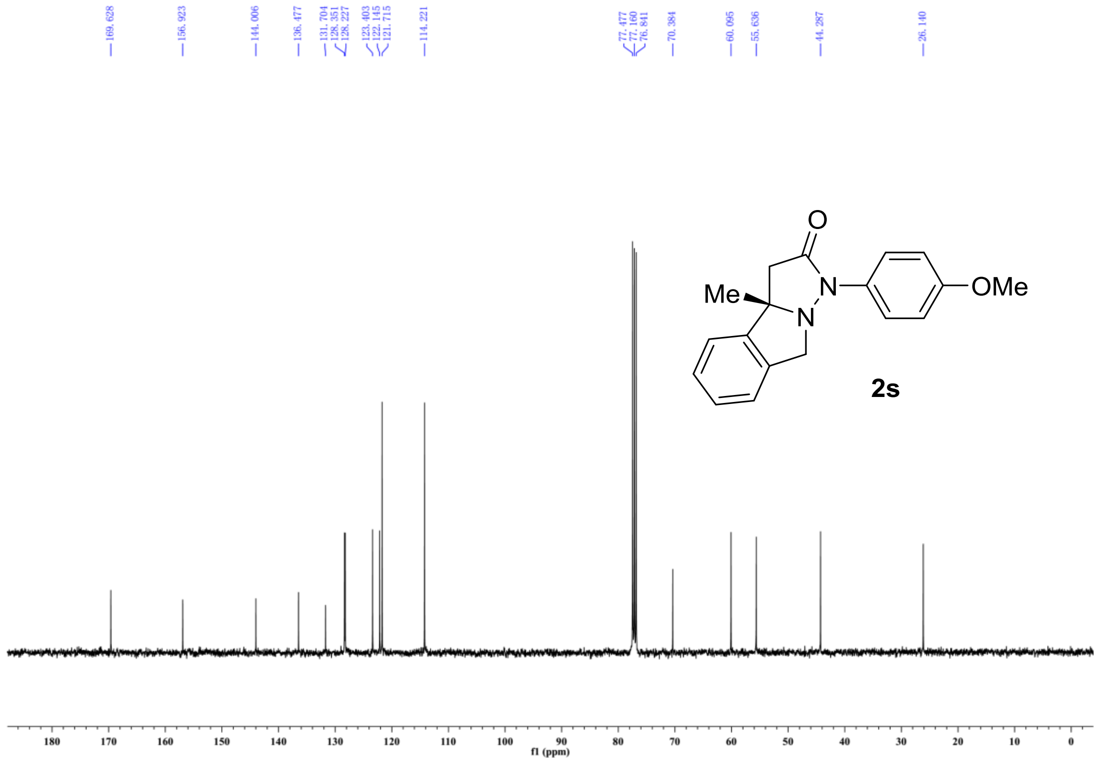

Figure S258. ^{13}C NMR of 2s, related to Table 2.

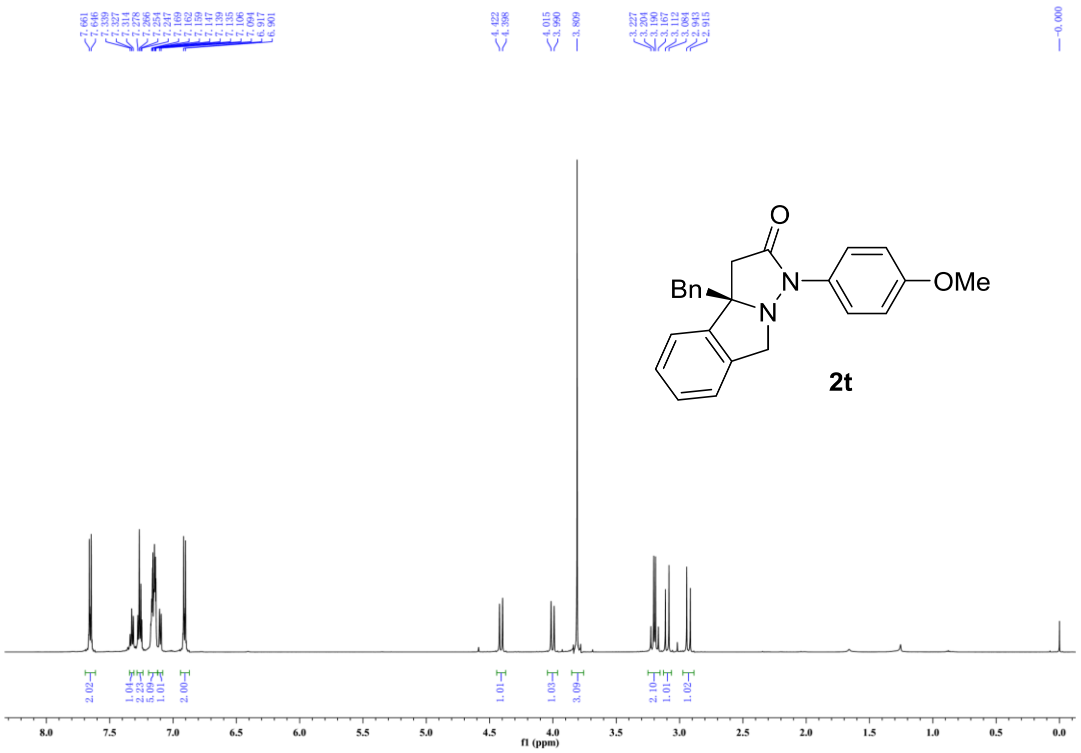

Figure S259. ^{1}H NMR of 2t, related to Table 2.

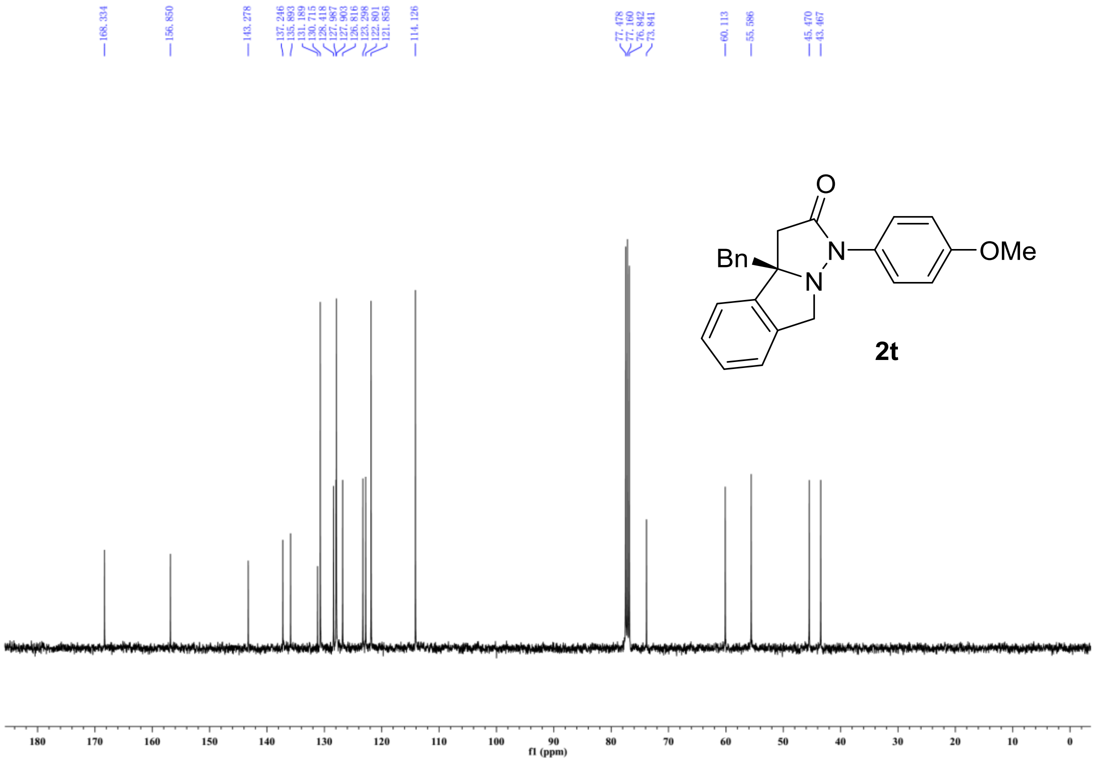

Figure S260. ^{13}C NMR of 2t, related to Table 2.

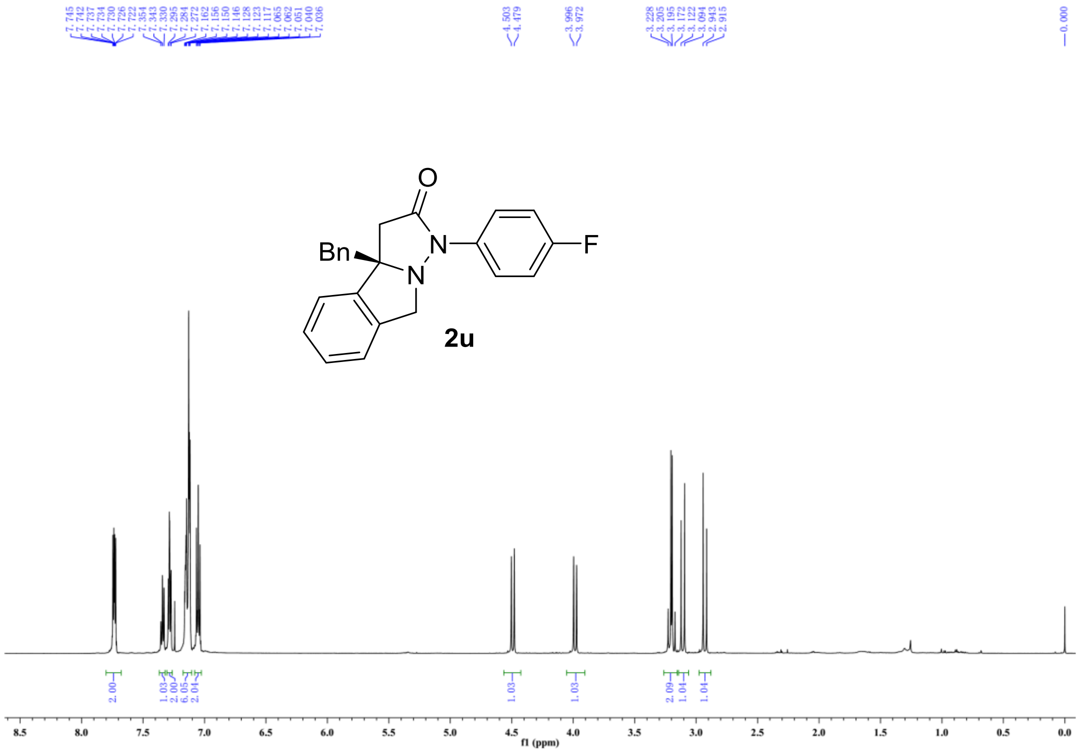

Figure S261. ^{1}H NMR of 2u, related to Table 2.

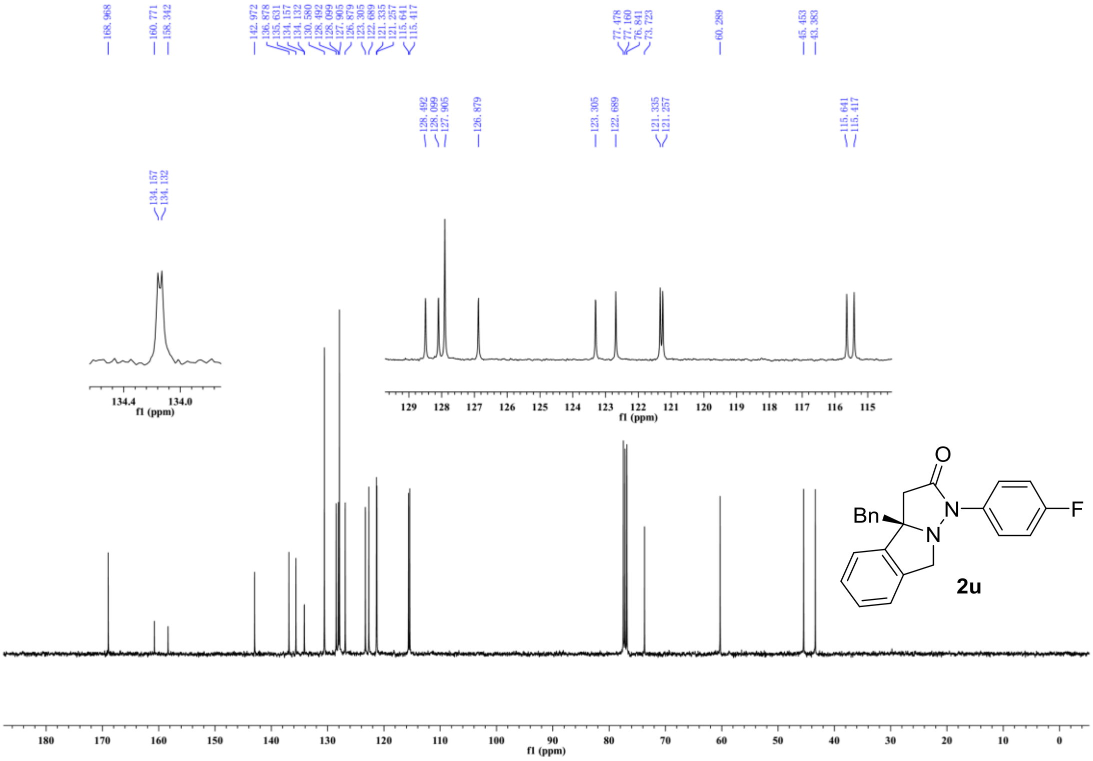

Figure S262. ^{13}C NMR of 2u, related to Table 2.

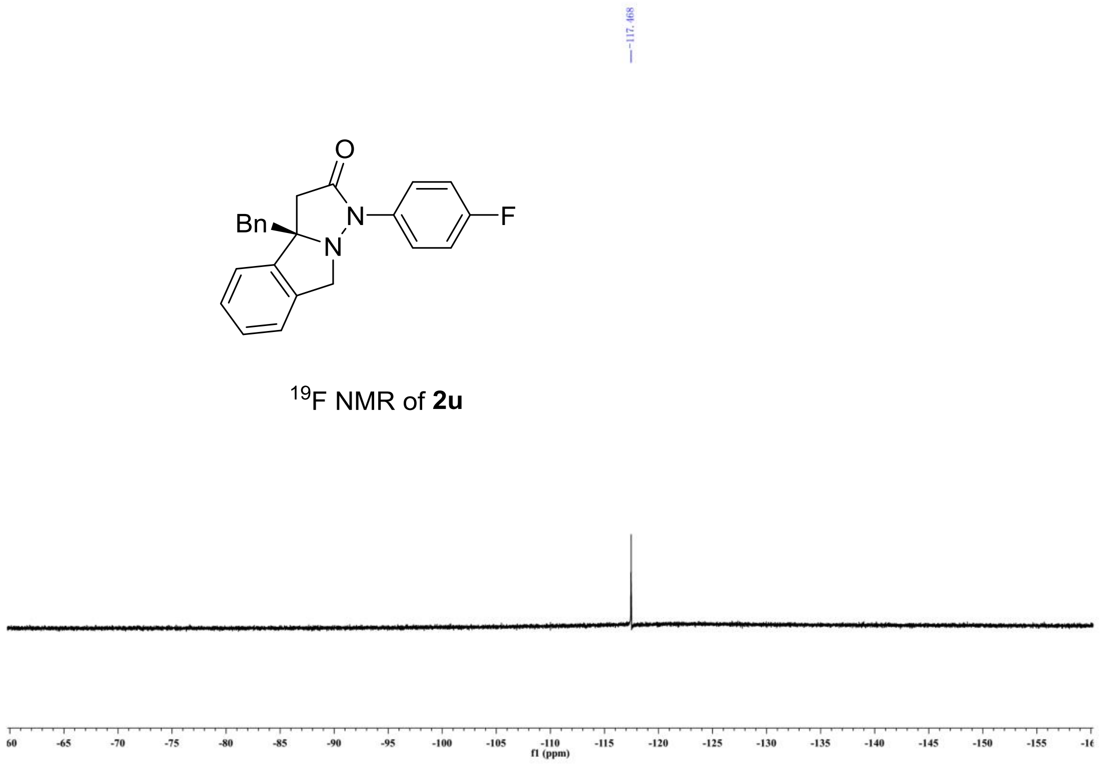

Figure S263. ^{19}F NMR of 2u, related to Table 2.

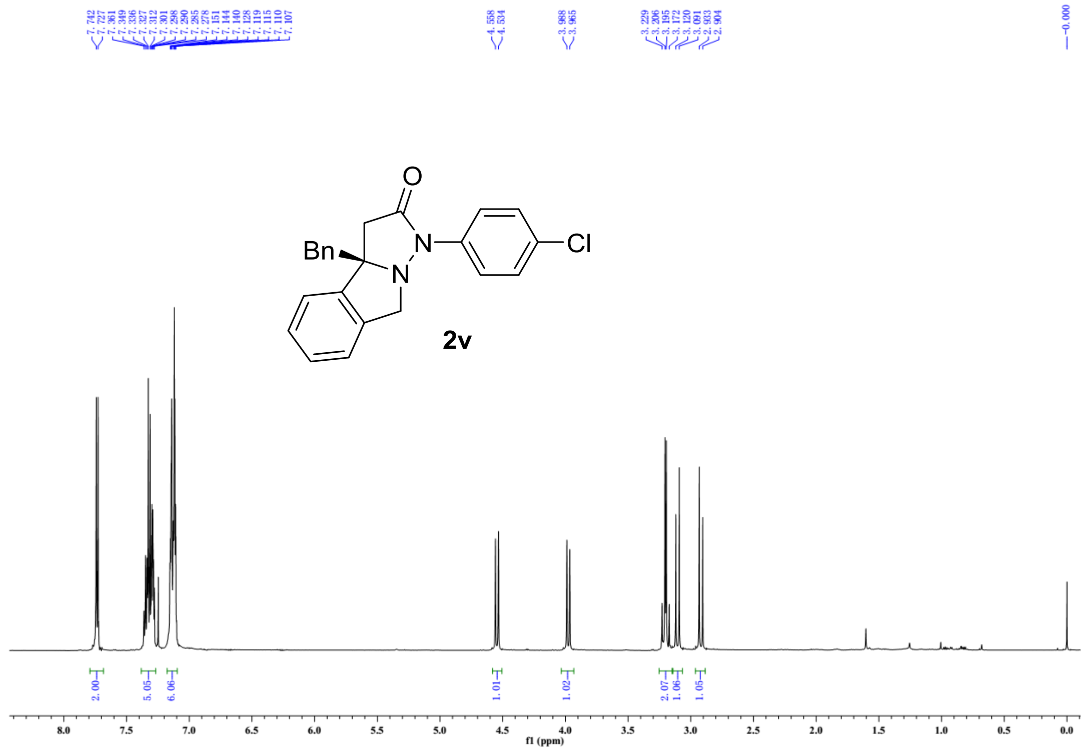

Figure S264. ^{1}H NMR of 2v, related to Table 2.

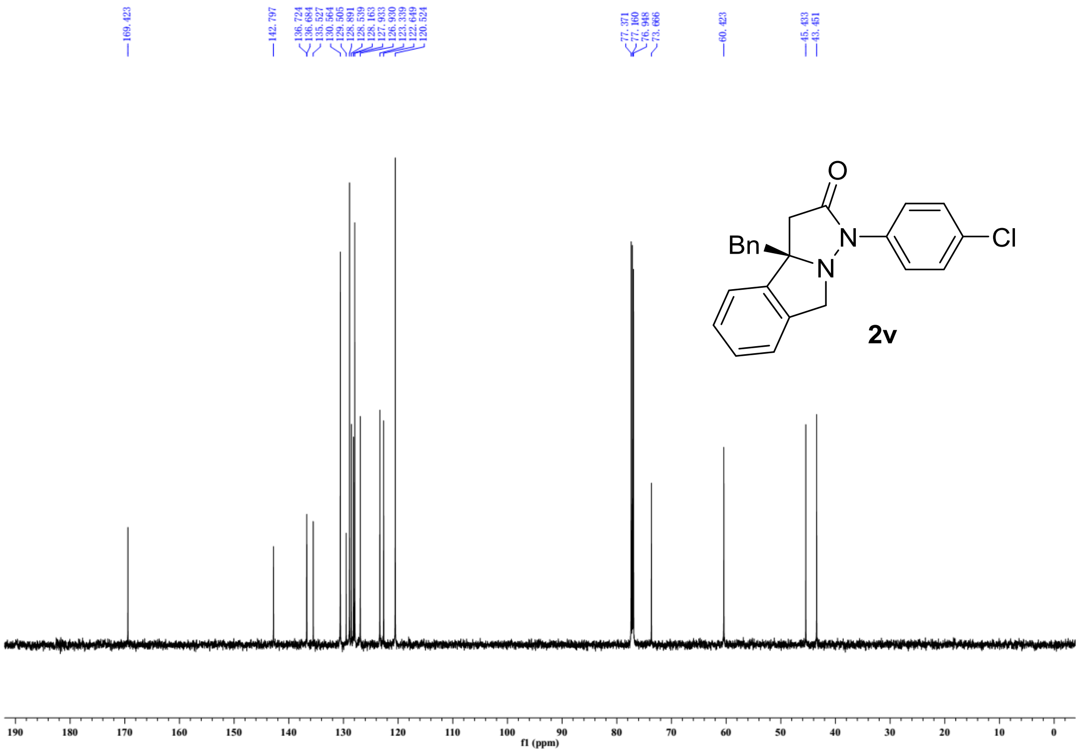

Figure S265. **^{13}C NMR of 2v, related to Table 2.**

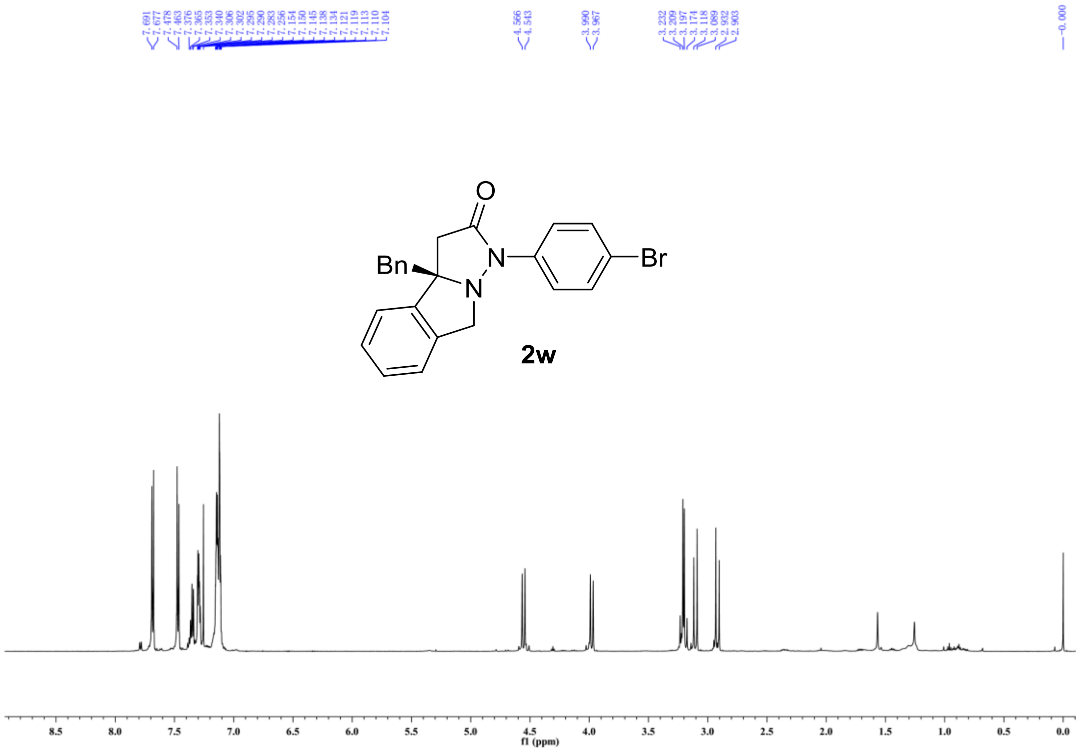

Figure S266. **^{1}H NMR of 2w, related to Table 2.**

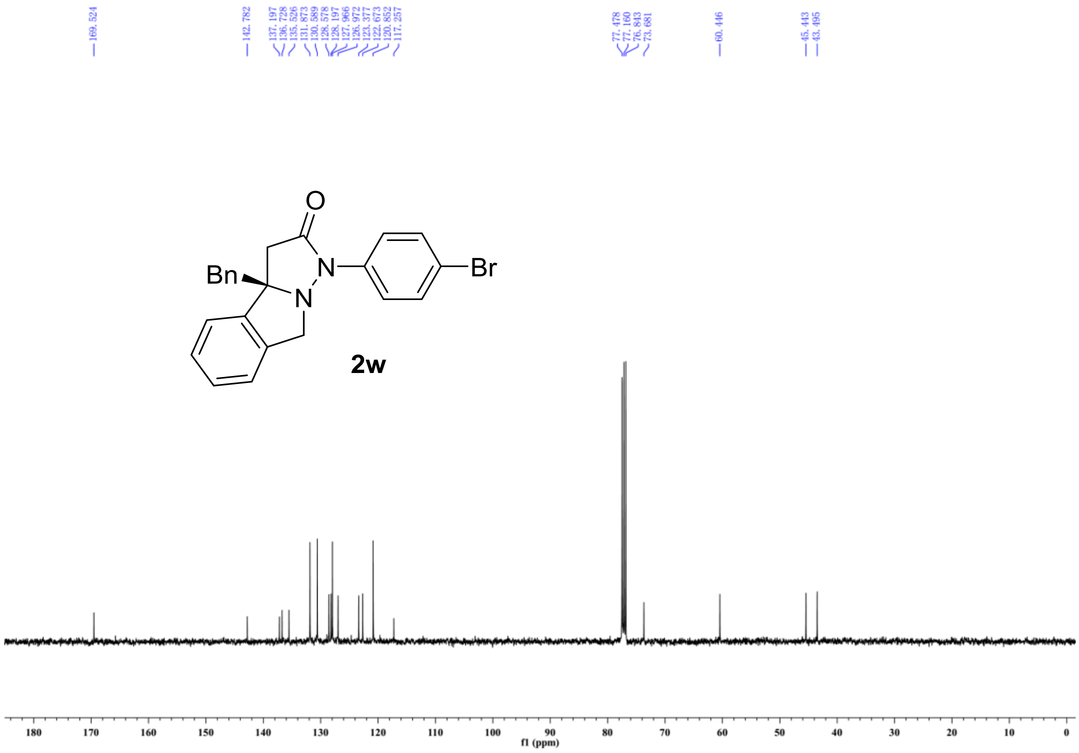

Figure S267. ^{13}C NMR of 2w, related to Table 2.

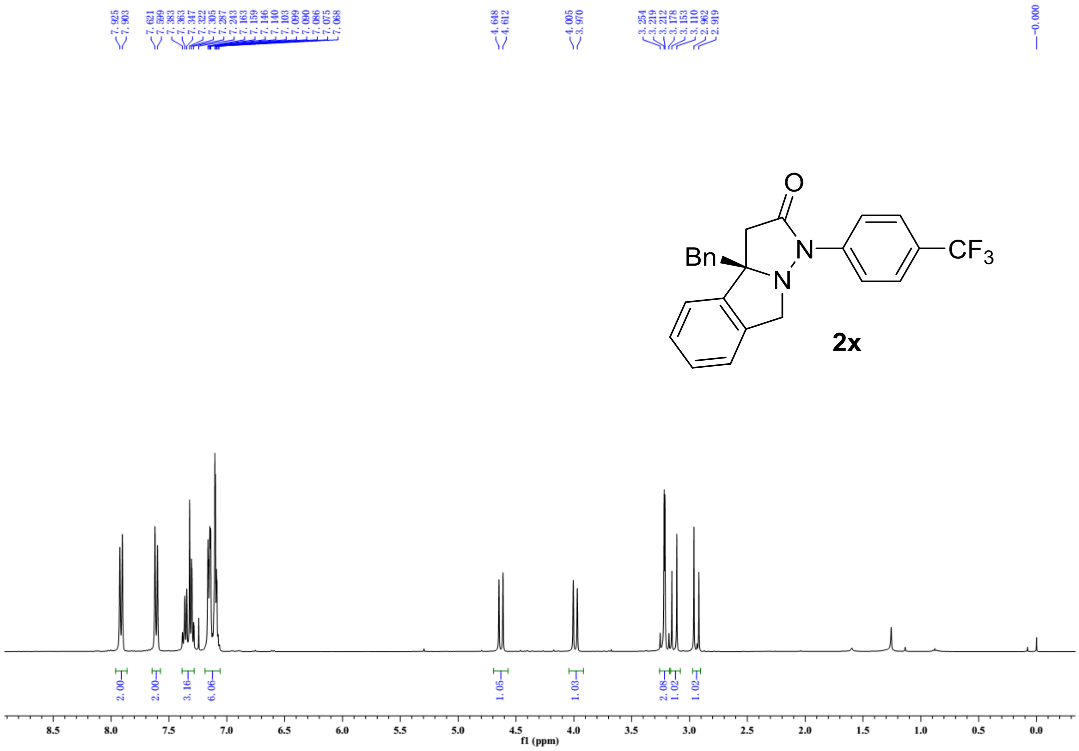

Figure S268. ^{1}H NMR of 2x, related to Table 2.

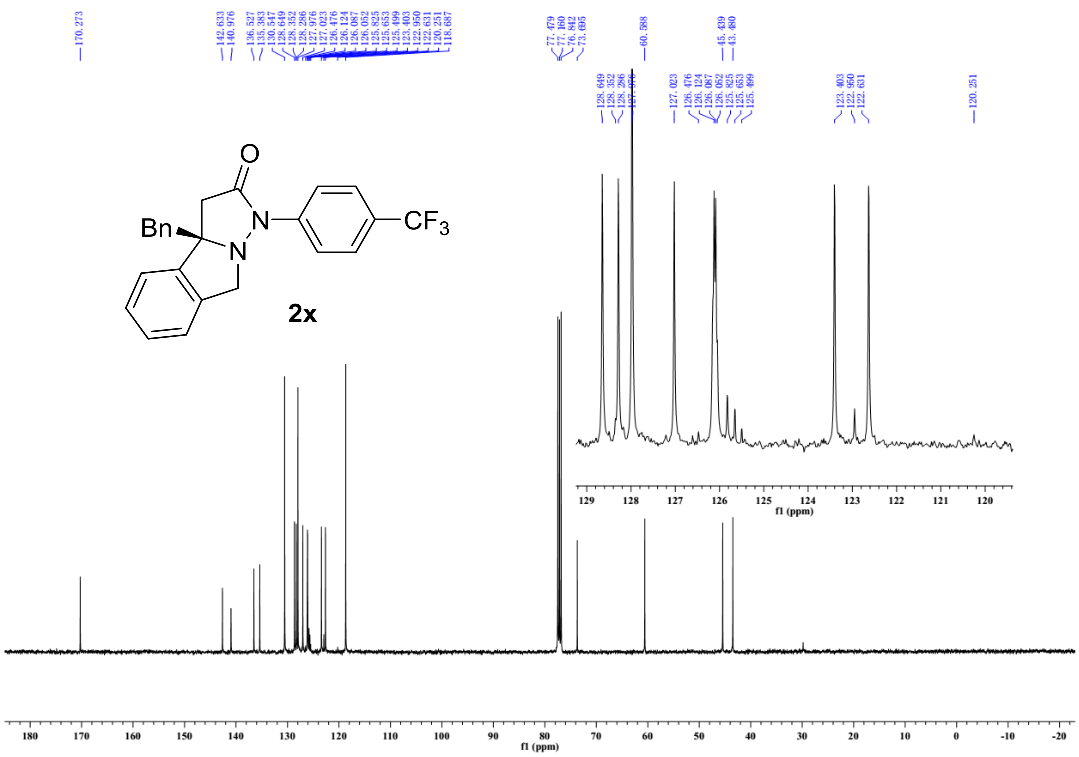

Figure S269. **^{13}C NMR of 2x, related to Table 2.**

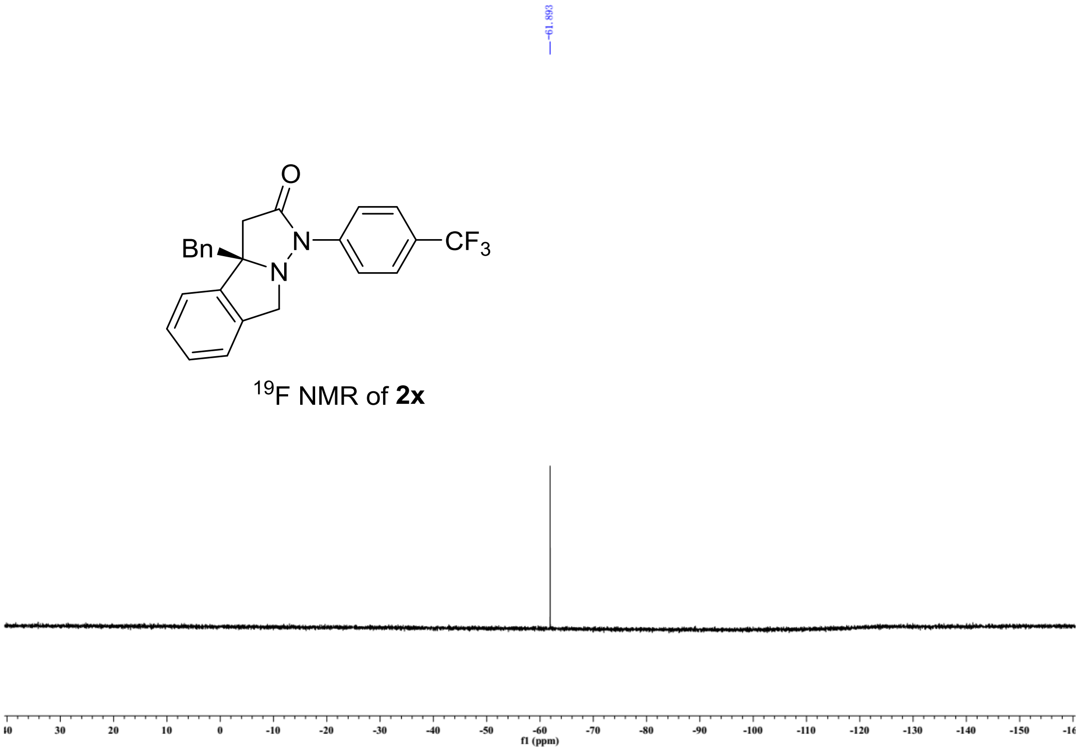

Figure S270. **^{19}F NMR of 2x, related to Table 2.**

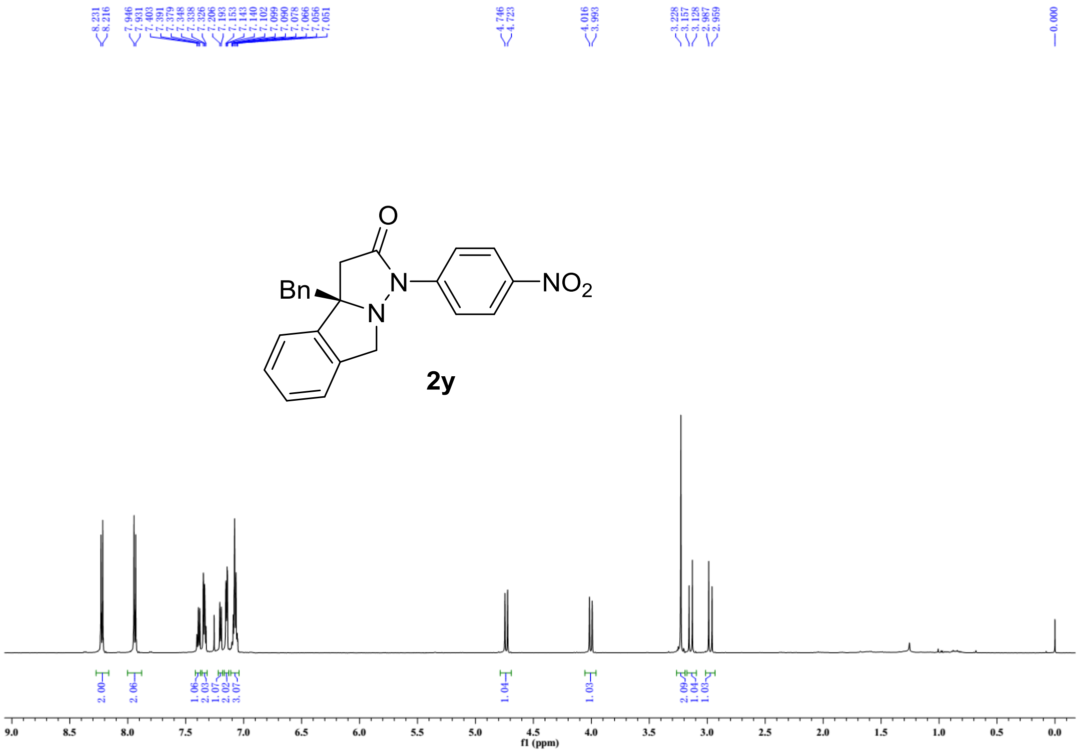

Figure S271. ^{1}H NMR of 2y, related to Table 2.

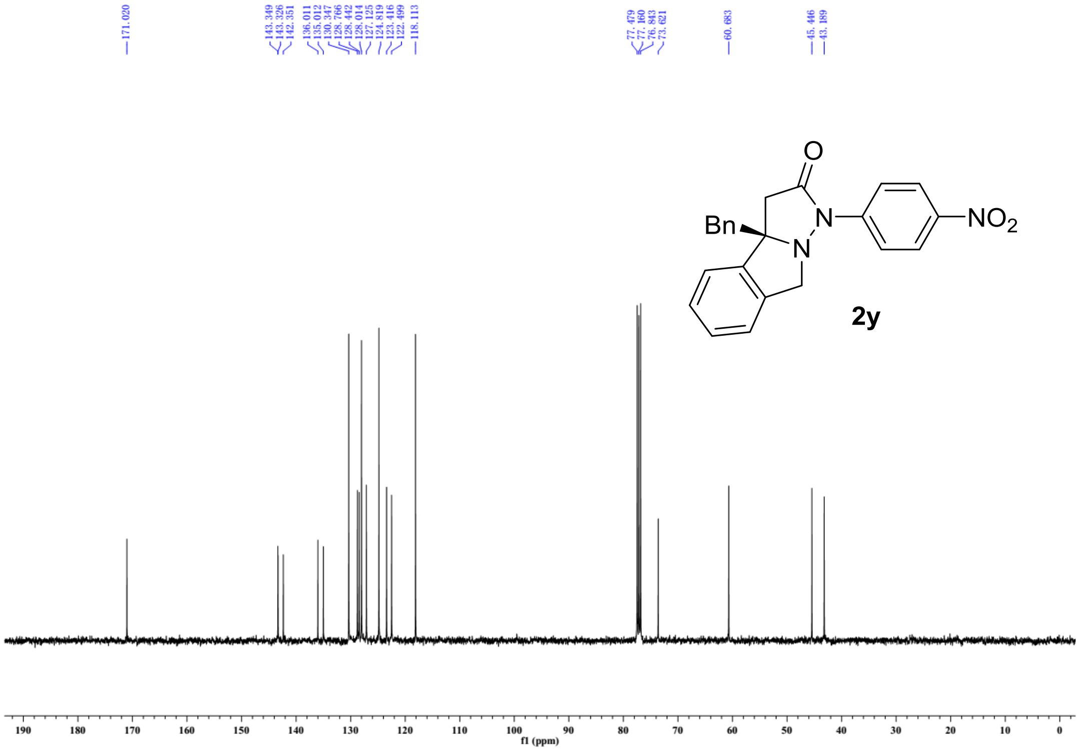

Figure S272. ^{13}C NMR of 2y, related to Table 2.

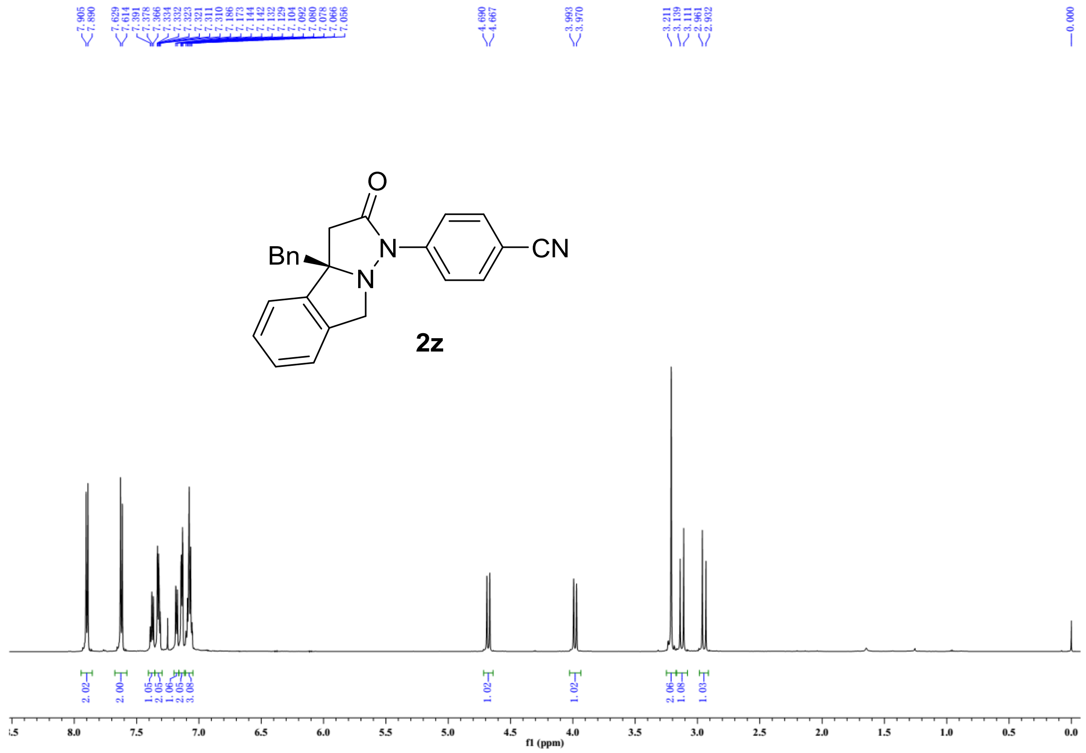

Figure S273. ^{1}H NMR of 2z, related to Table 2.

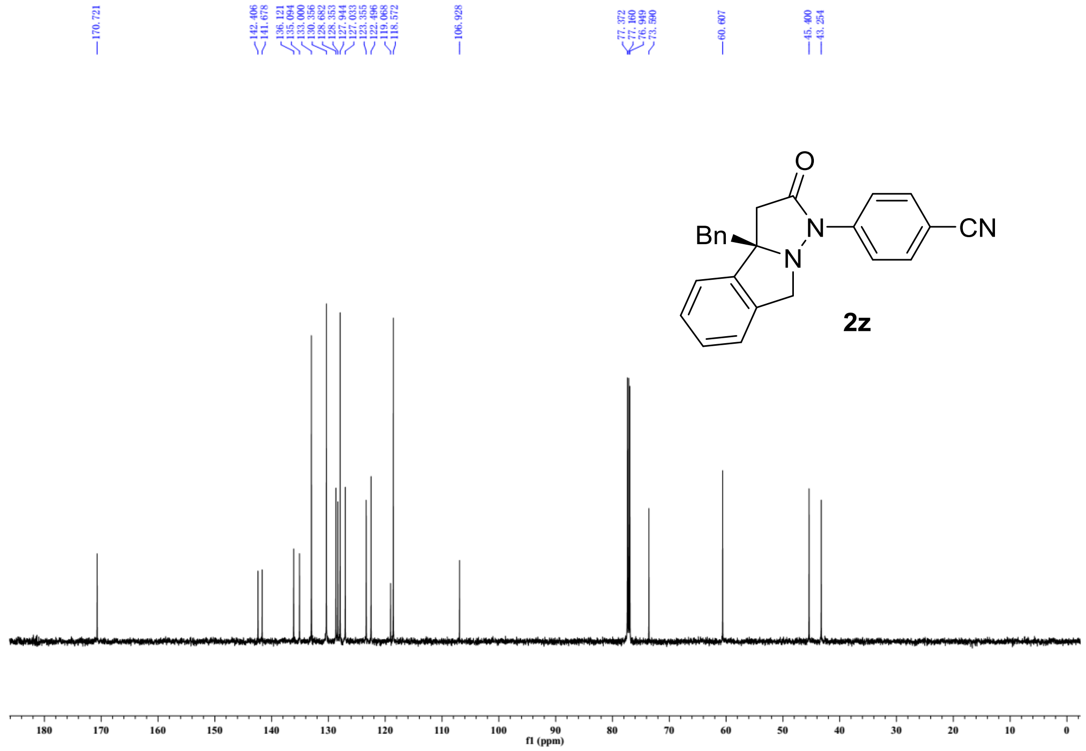

Figure S274. ^{13}C NMR of 2z, related to Table 2.

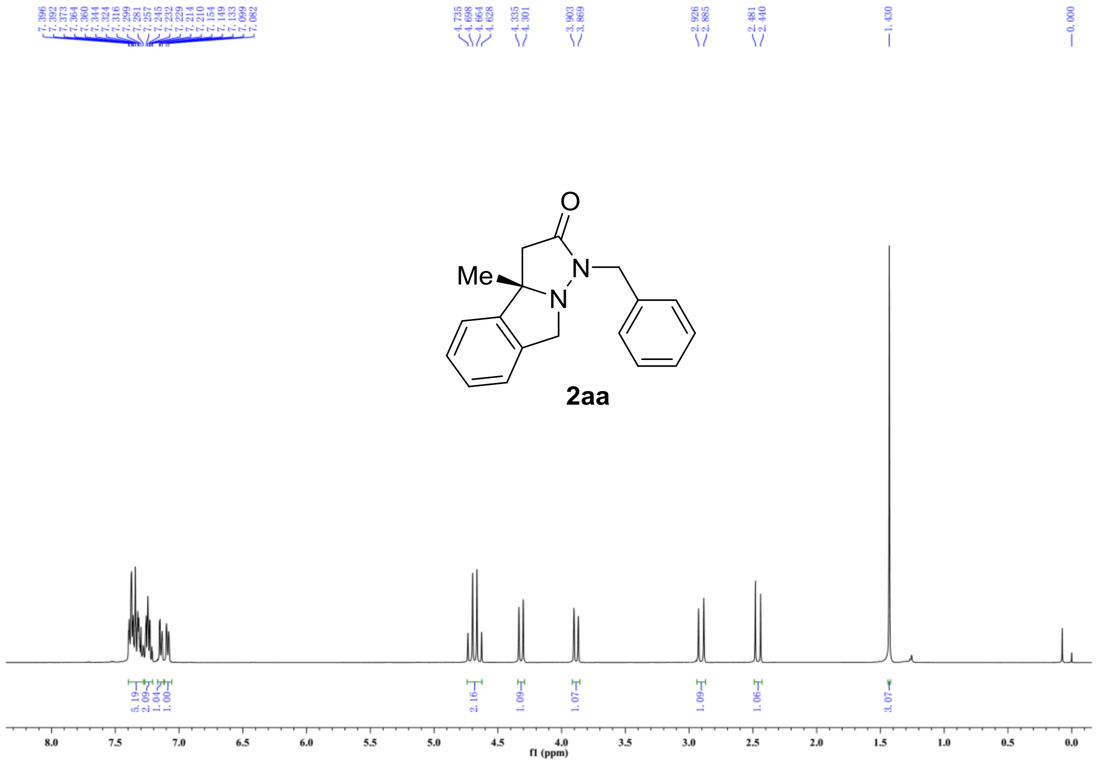

Figure S275. ^{1}H NMR of 2aa, related to Table 2.

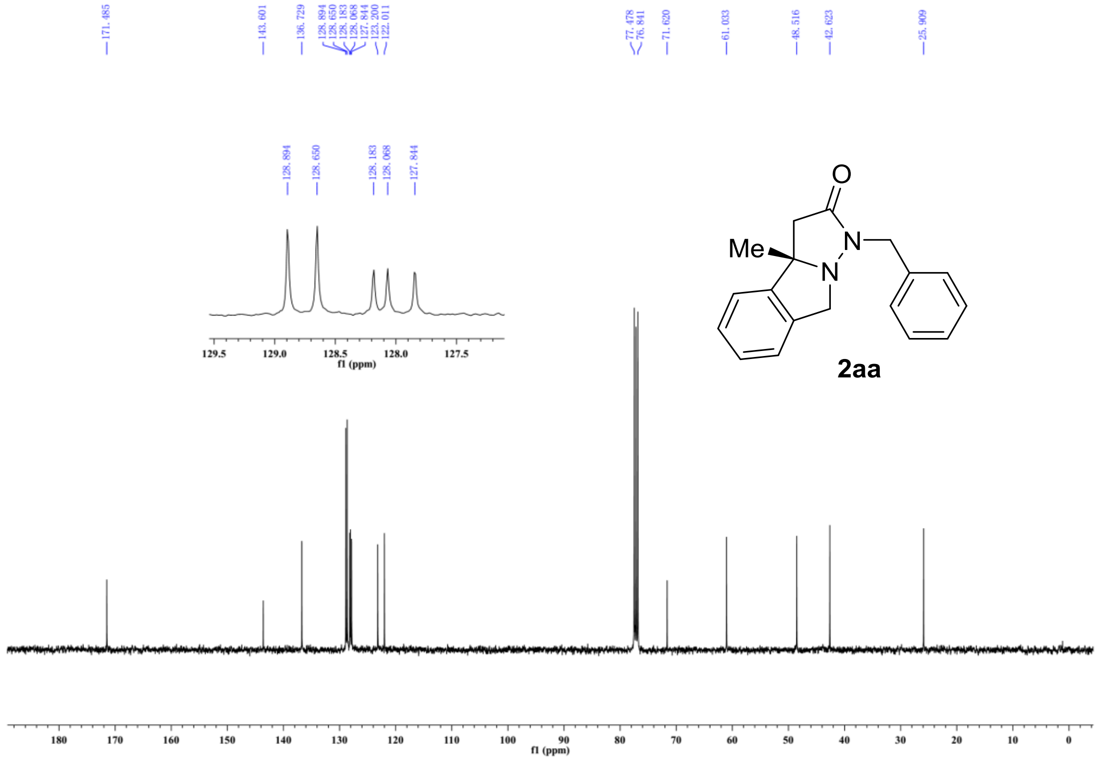

Figure S276. ^{13}C NMR of 2aa, related to Table 2.

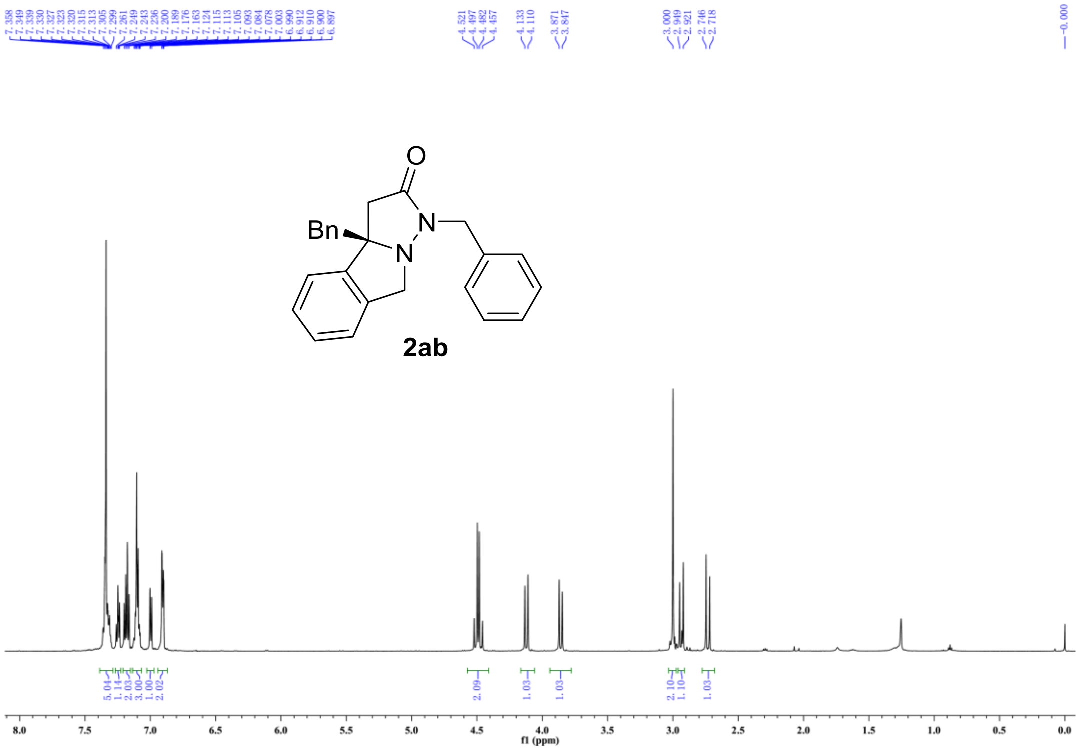

Figure S277. ^{1}H NMR of 2ab, related to Table 2.

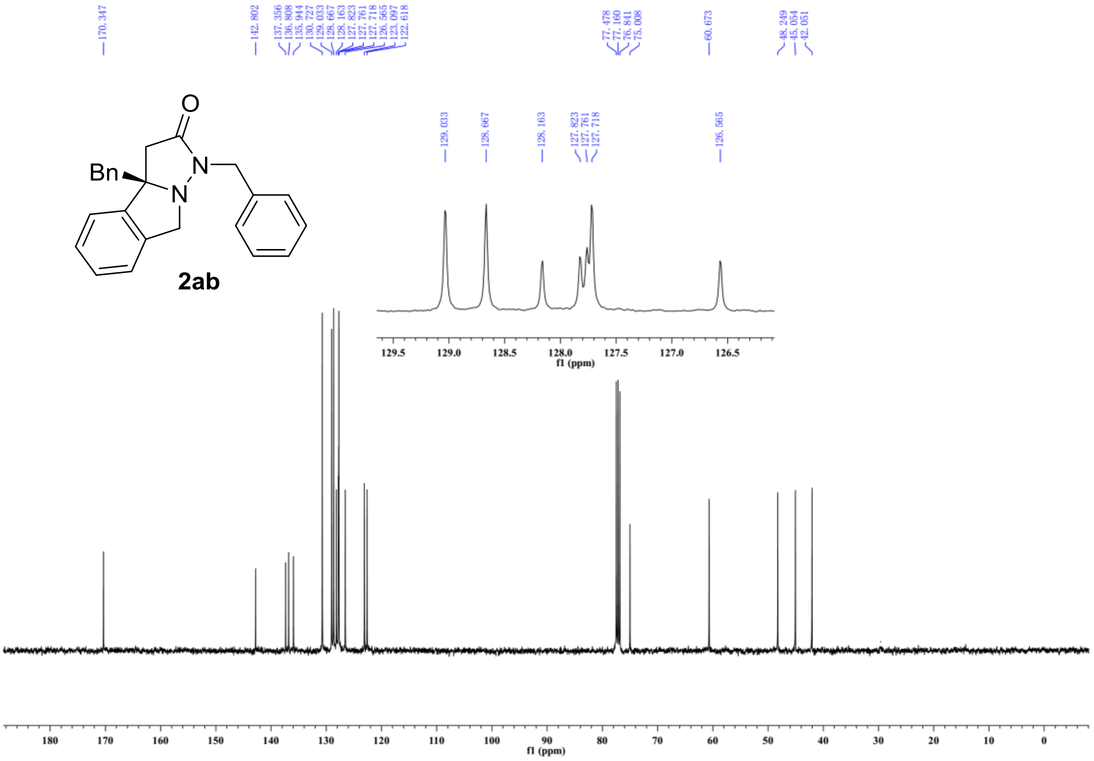

Figure S278. ^{13}C NMR of 2ab, related to Table 2.

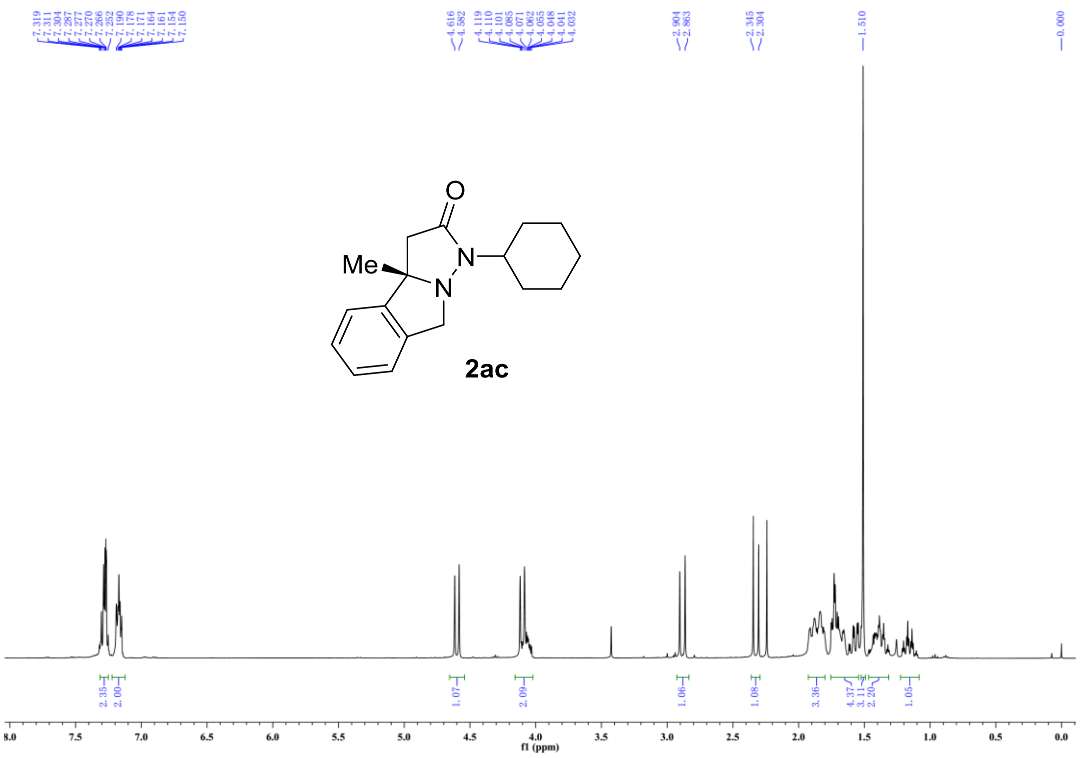

Figure S279. [1]**H NMR of 2ac, related to Table 2.**

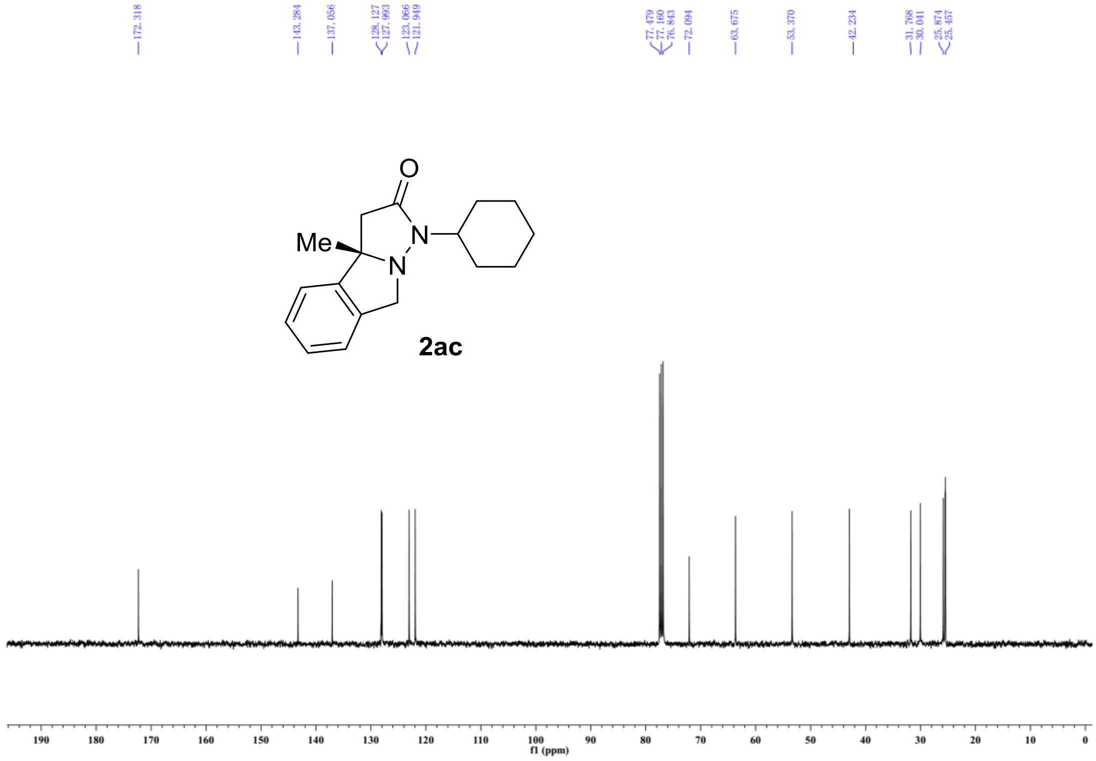

Figure S280. [13]**C NMR of 2ac, related to Table 2.**

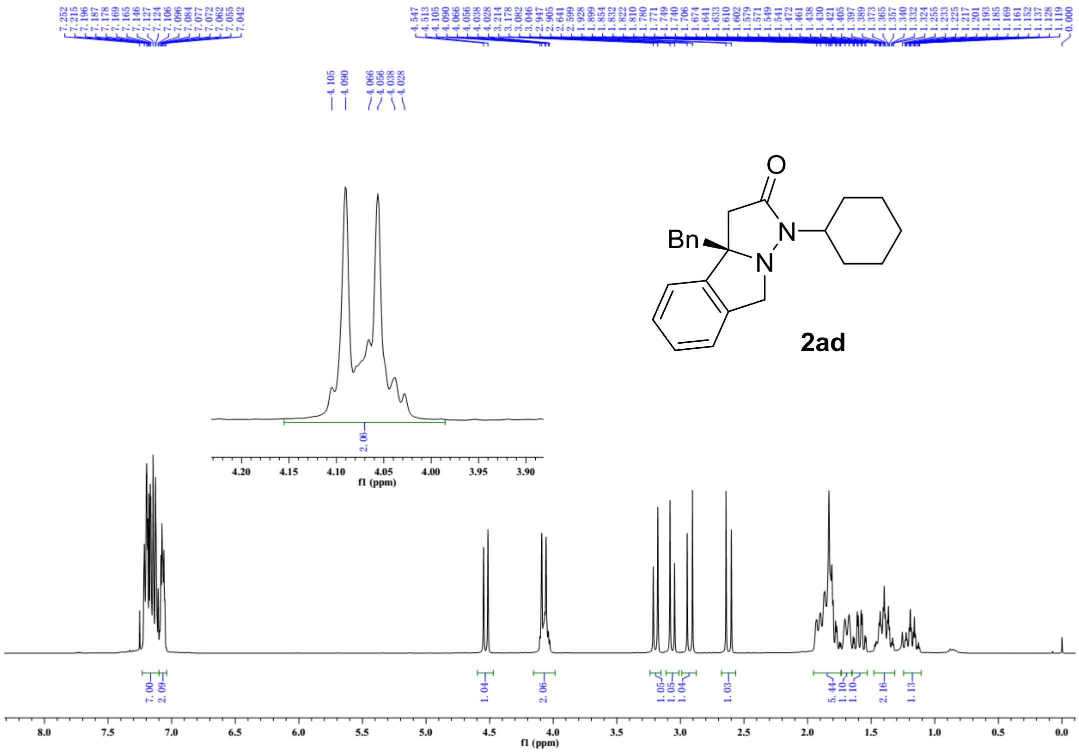

Figure S281. ^{1}H NMR of 2ad, related to Table 2.

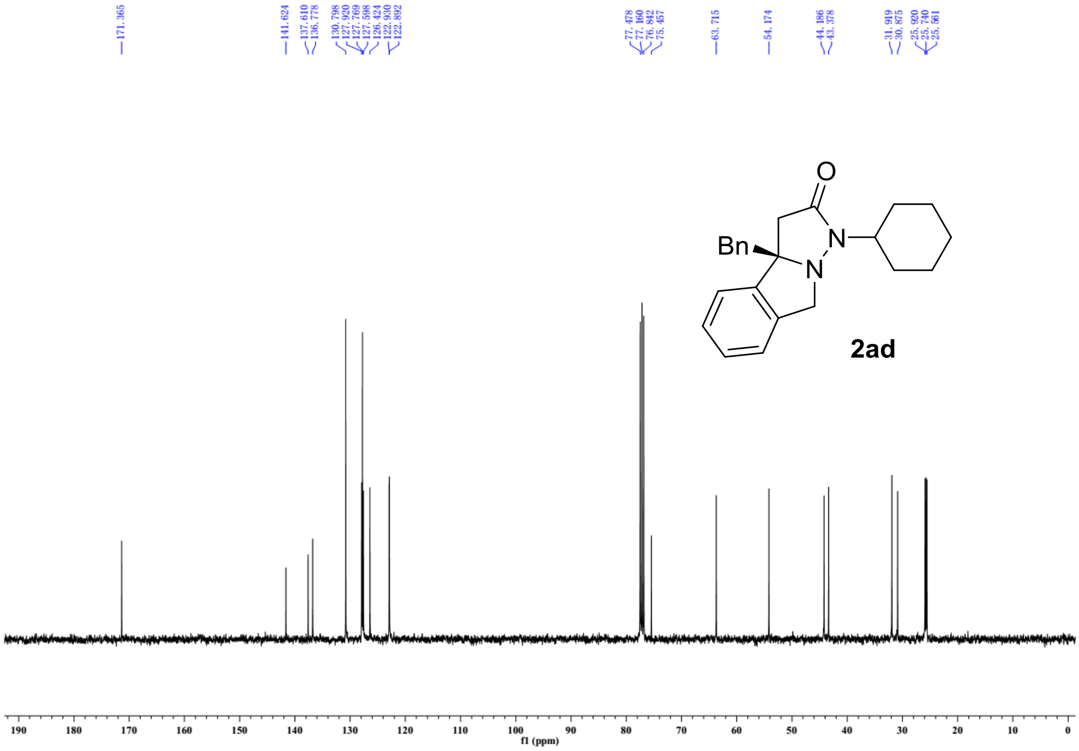

Figure S282. ^{13}C NMR of 2ad, related to Table 2.

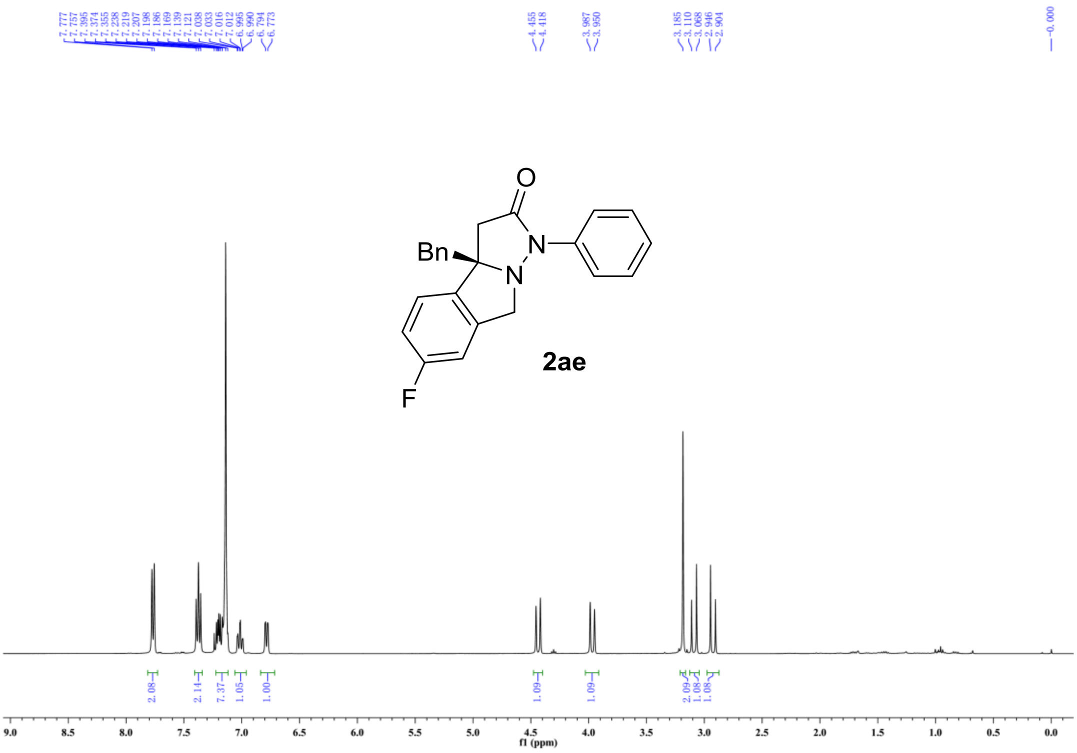

Figure S283. ¹H NMR of 2ae, related to Table 2.

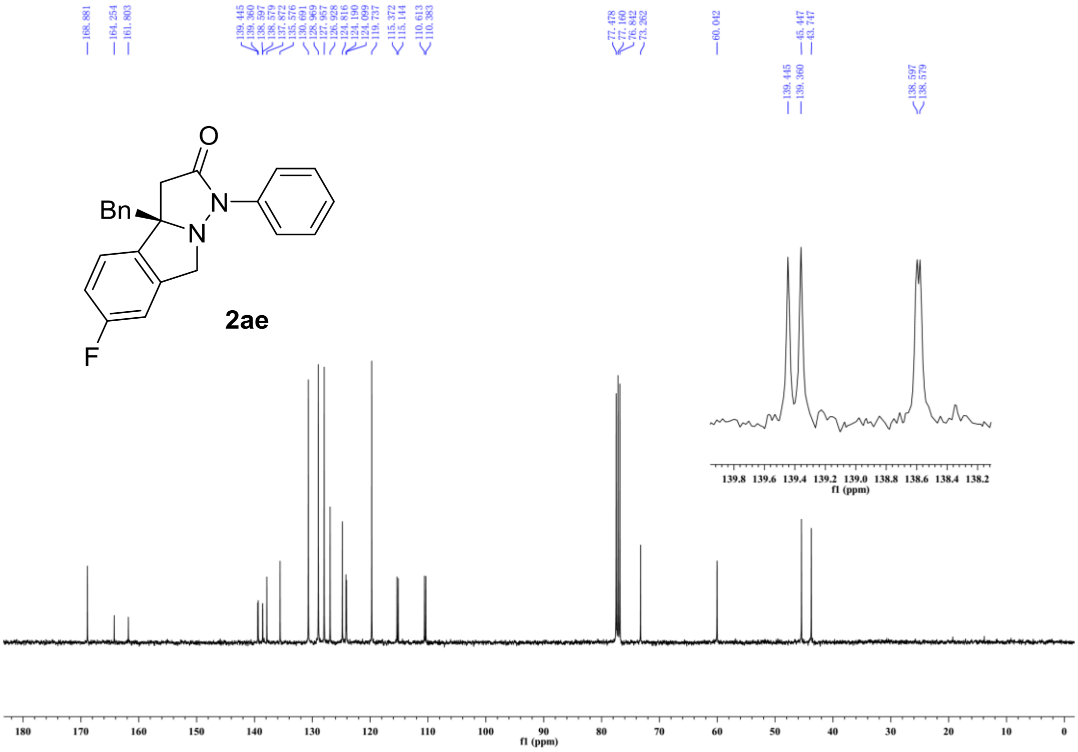

Figure S284. ¹³C NMR of 2ae, related to Table 2.

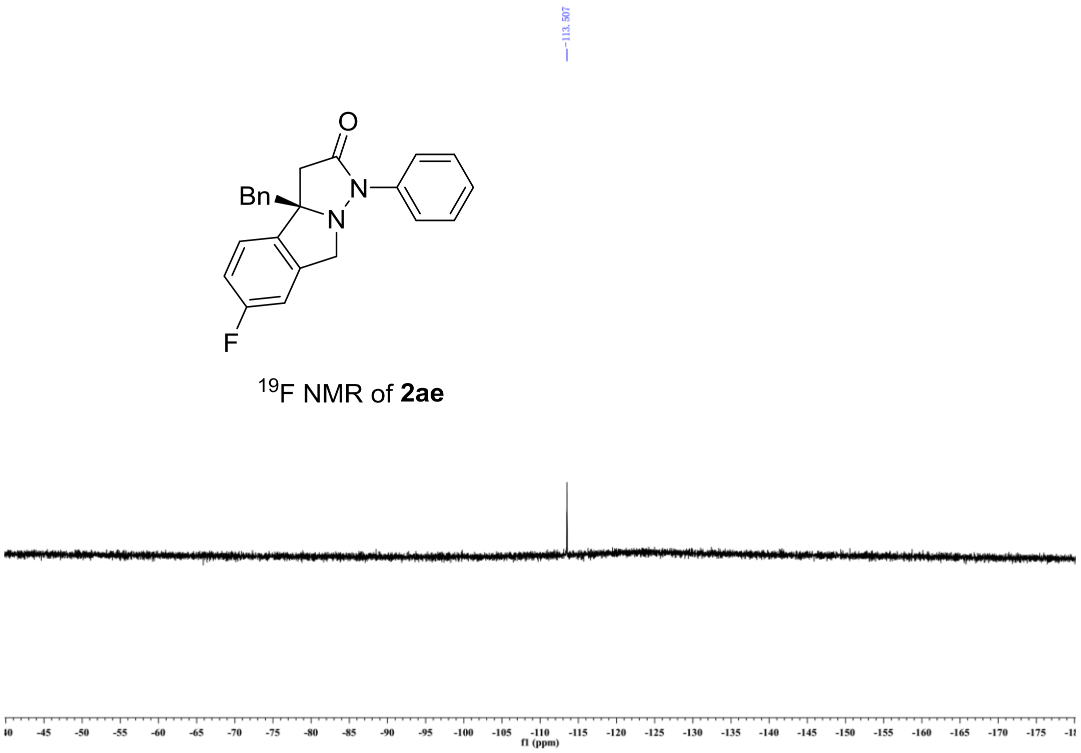

Figure S285. ^{19}F NMR of 2ae, related to Table 2.

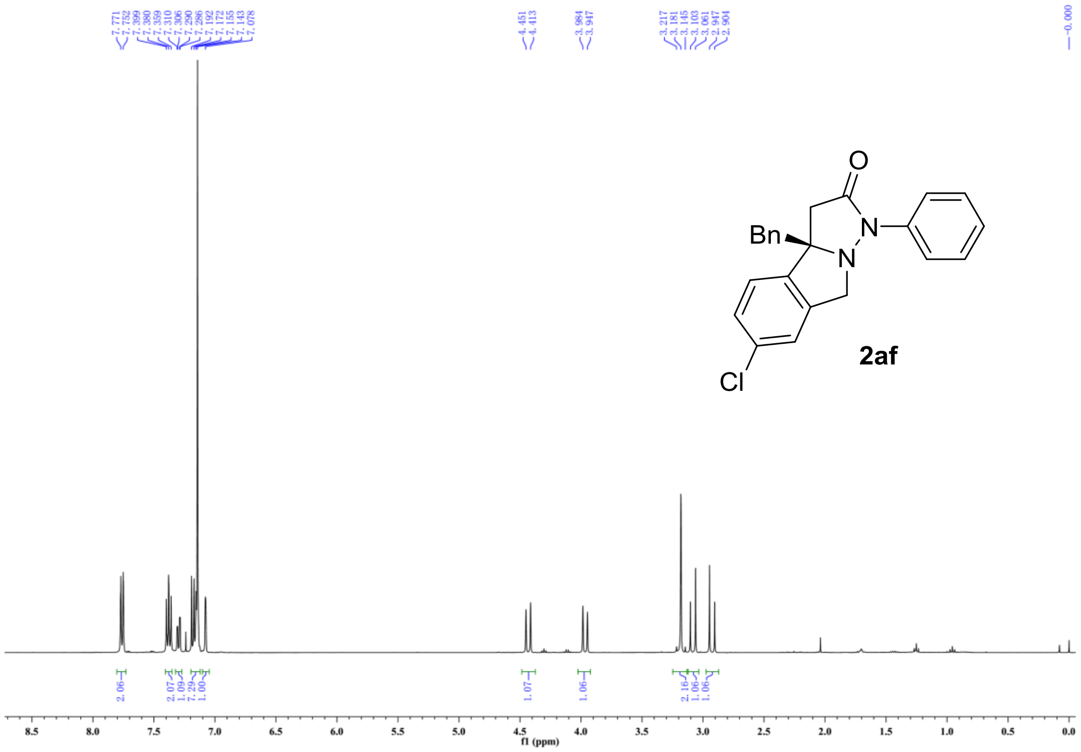

Figure S286. ^{1}H NMR of 2af, related to Table 2.

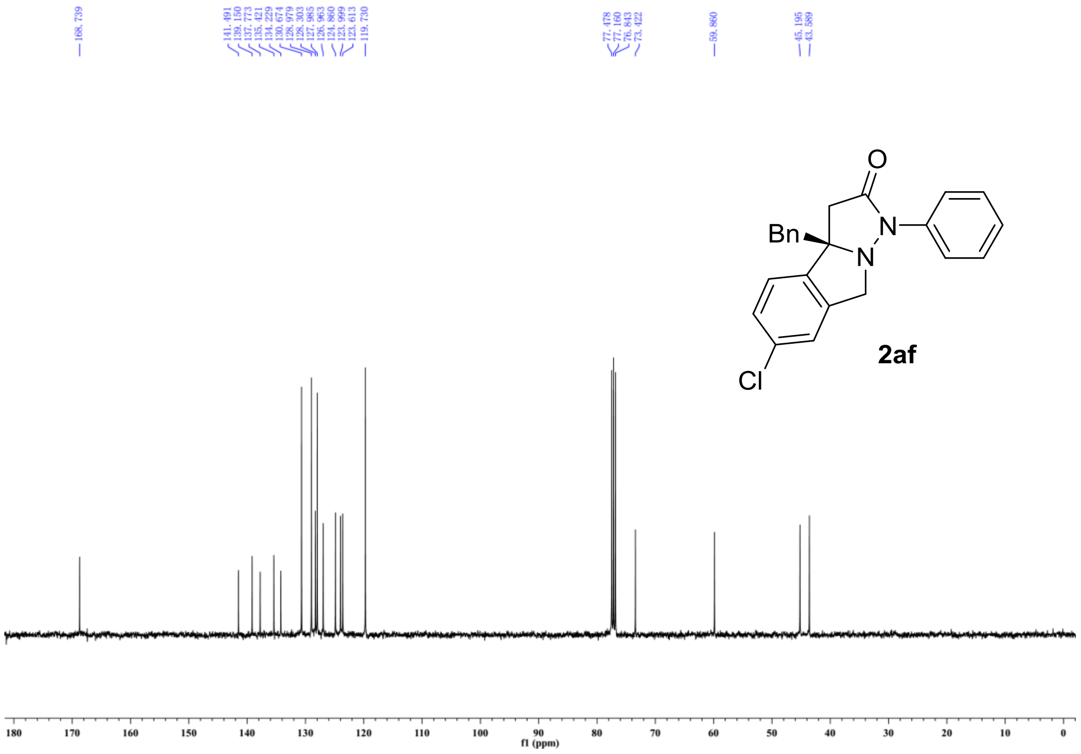

Figure S287. ^{13}C NMR of 2af, related to Table 2.

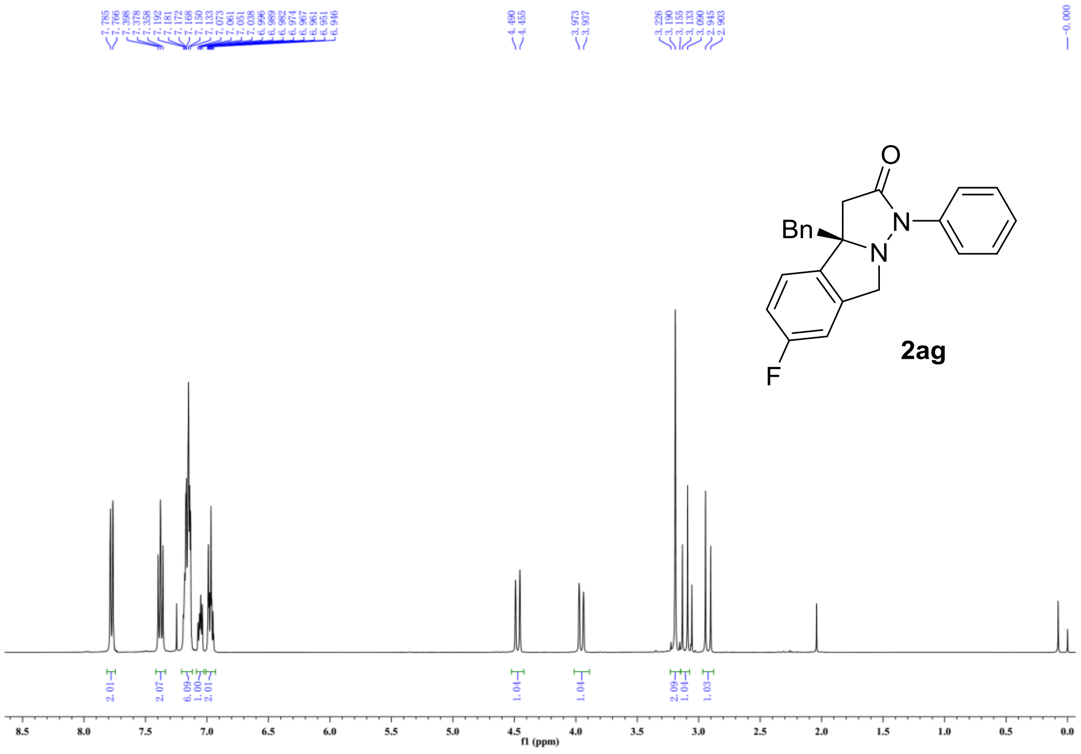

Figure S288. ^{1}H NMR of 2ag, related to Table 2.

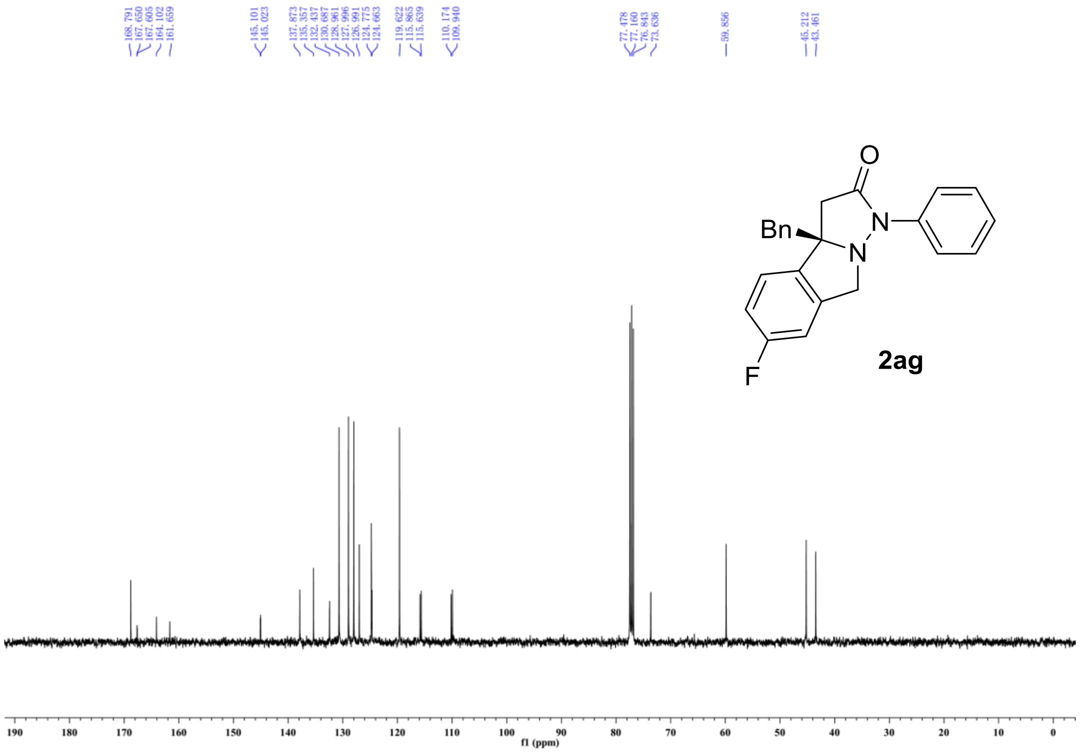

Figure S289. ^{13}C NMR of 2ag, related to Table 2.

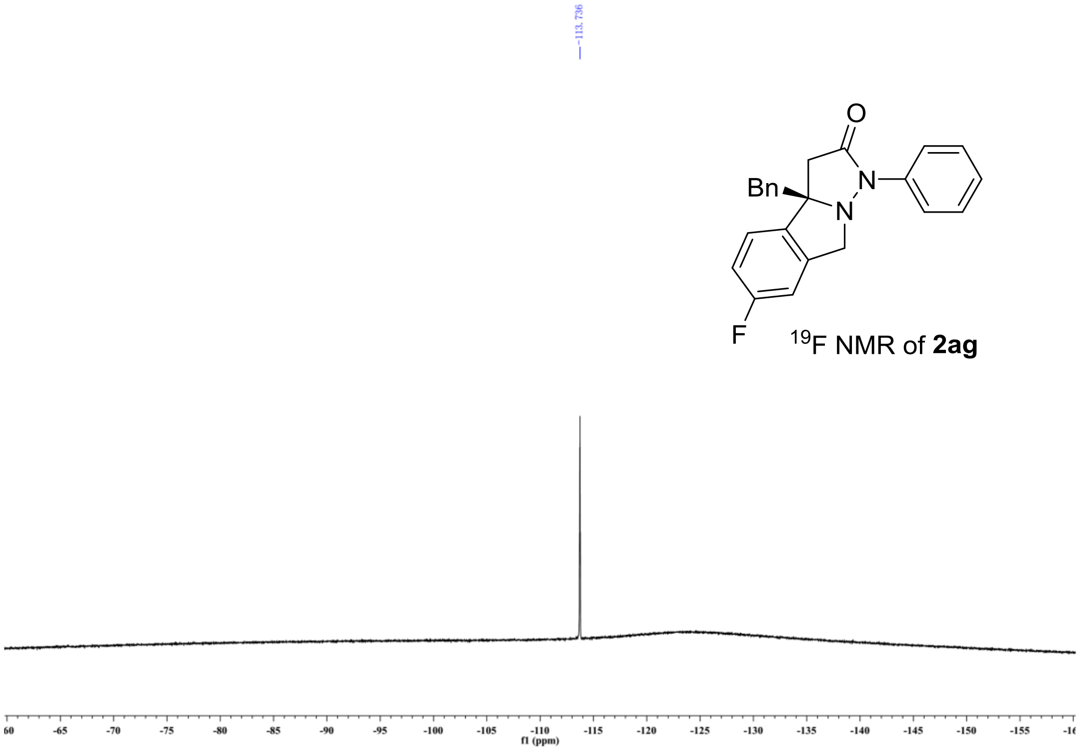

Figure S290. ^{19}F NMR of 2ag, related to Table 2.

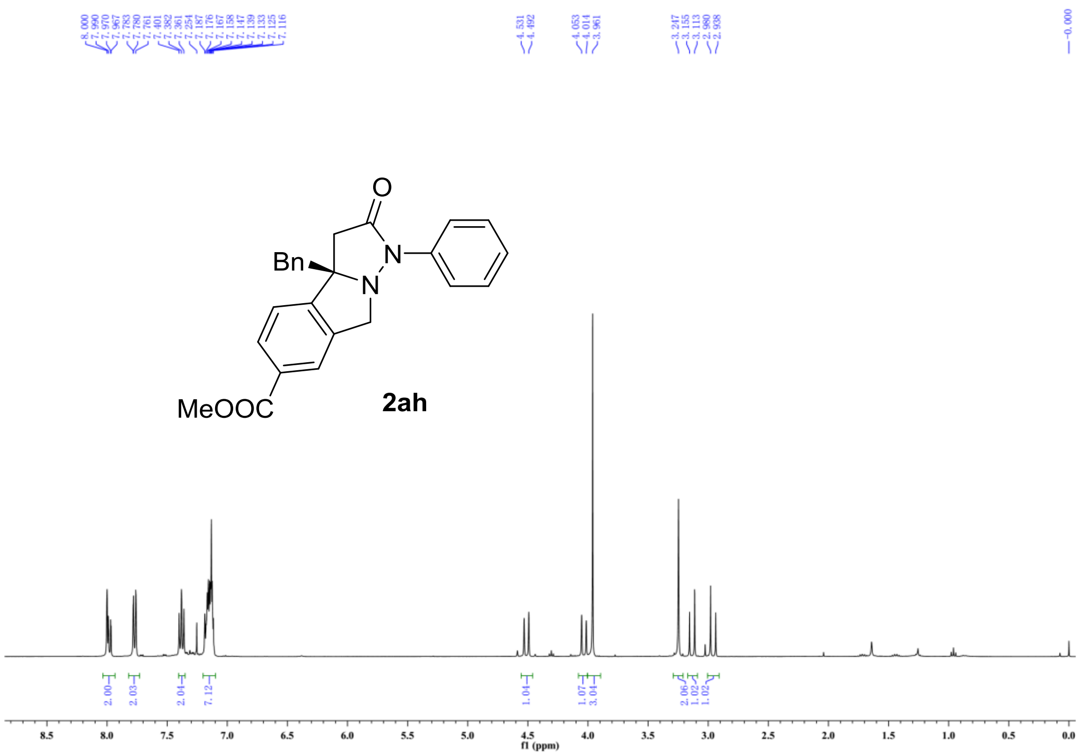

Figure S291. ^{1}H NMR of 2ah, related to Table 2.

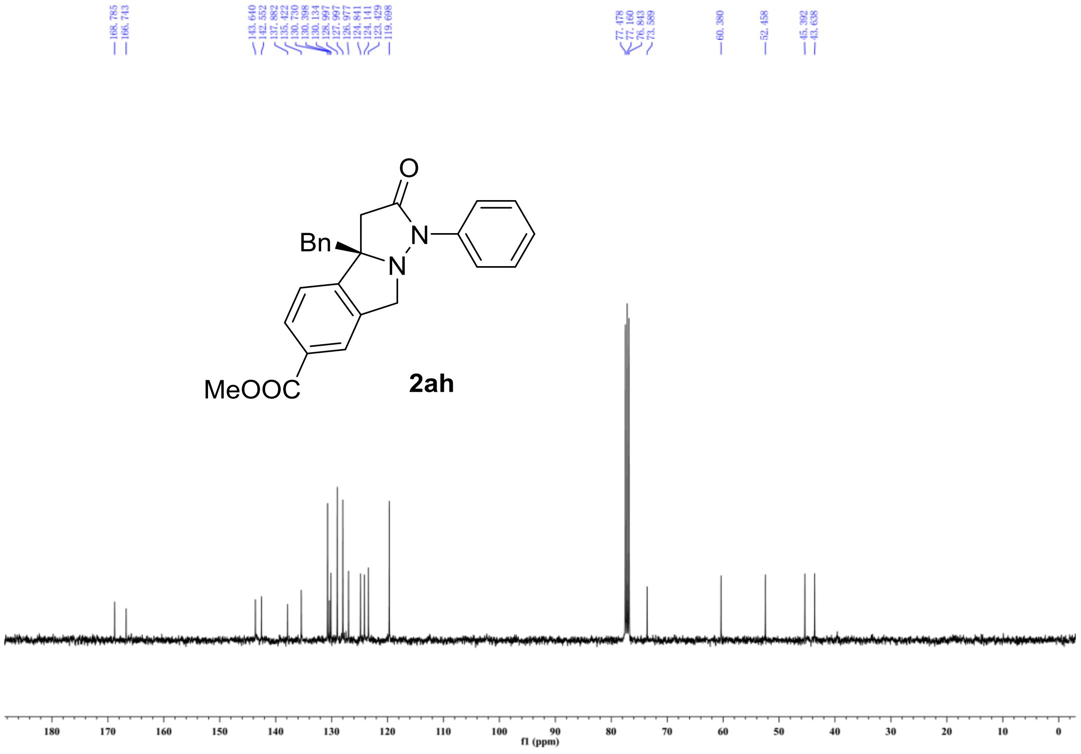

Figure S292. ^{13}C NMR of 2ah, related to Table 2.

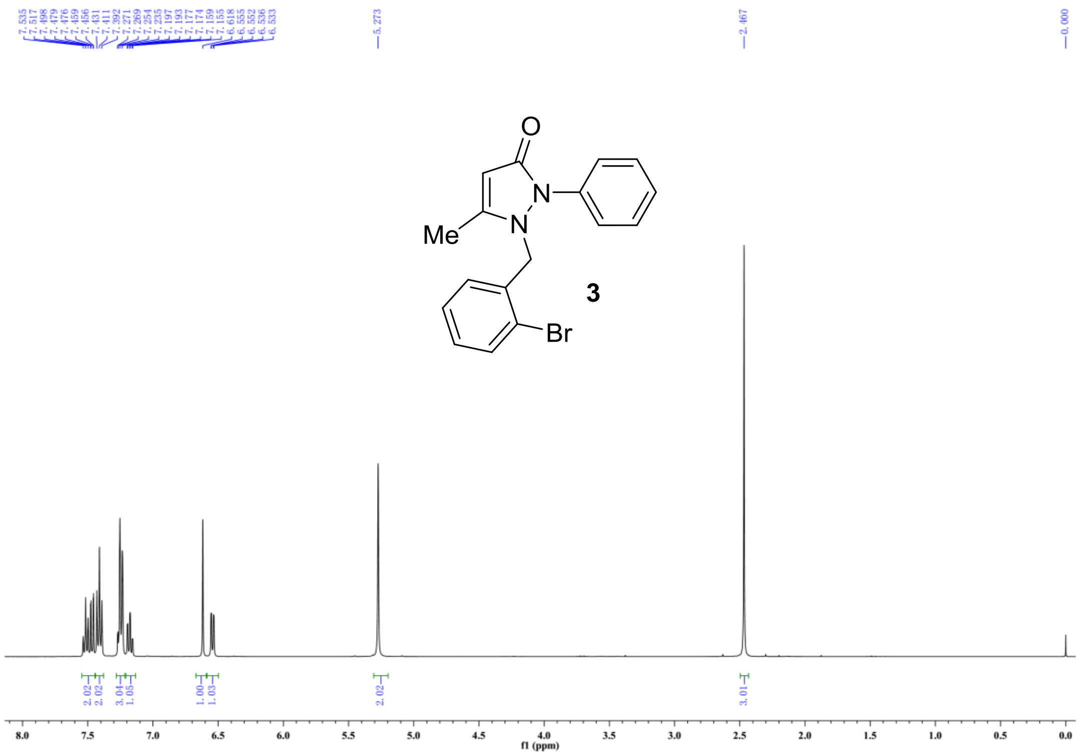

Figure S293. ¹H NMR of 3, related to Figure 4.

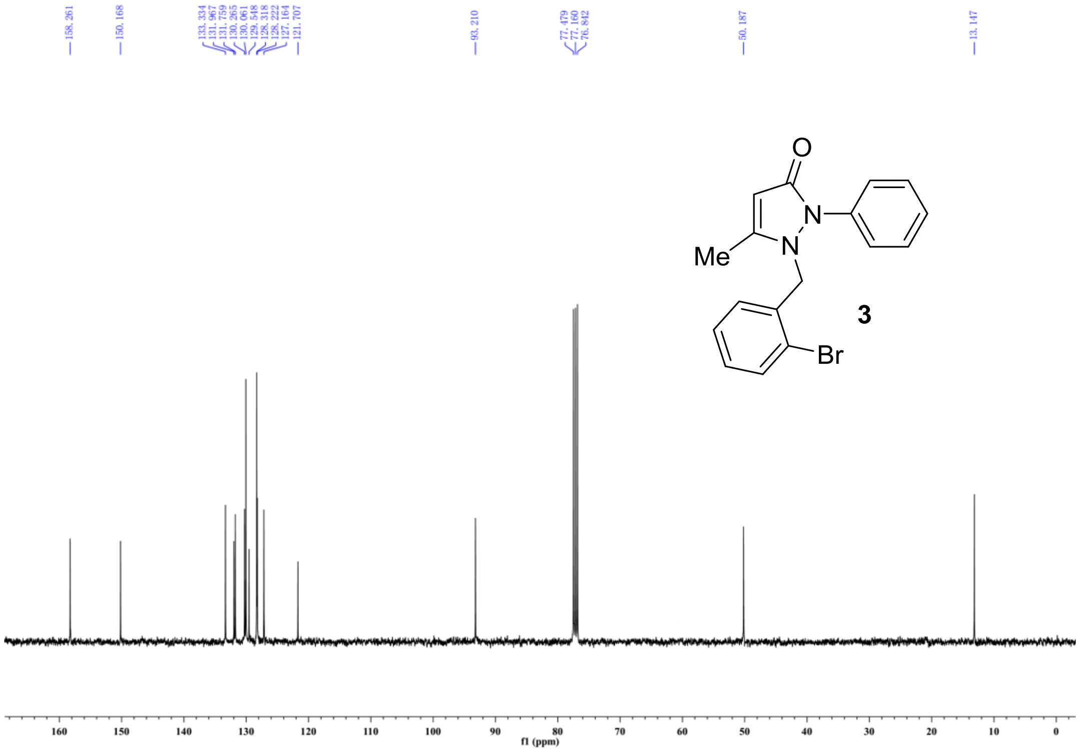

Figure S294. ¹³C NMR of 3, related to Figure 4.

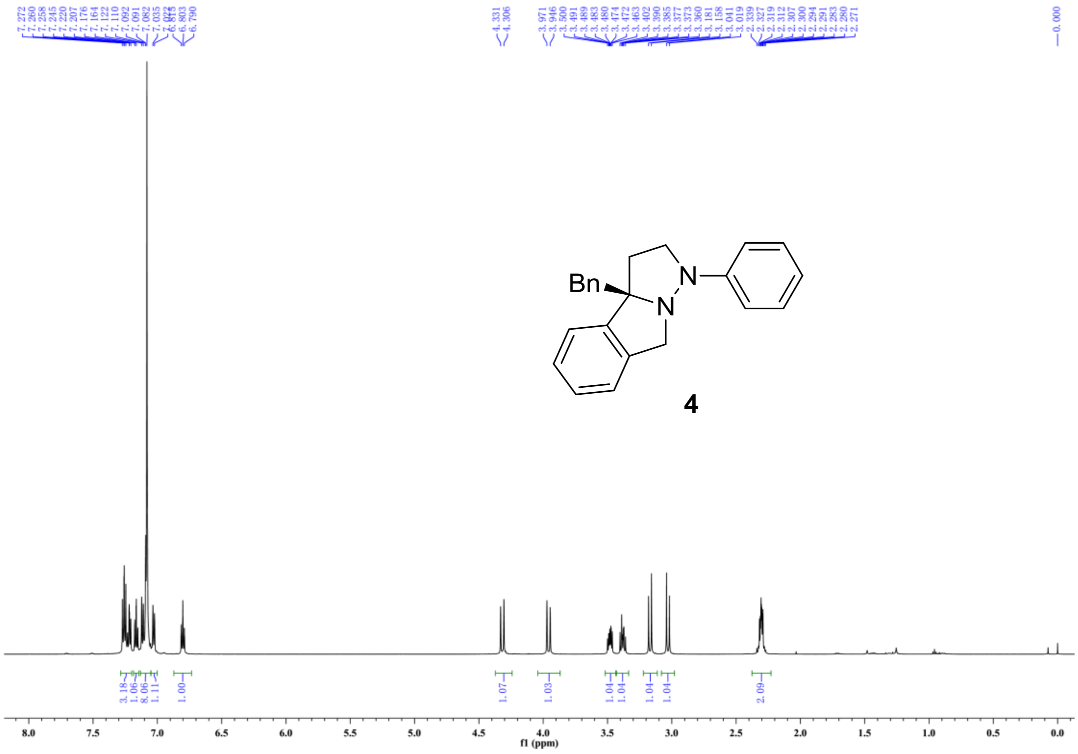

Figure S295. ^{1}H NMR of 4, related to Figure 4.

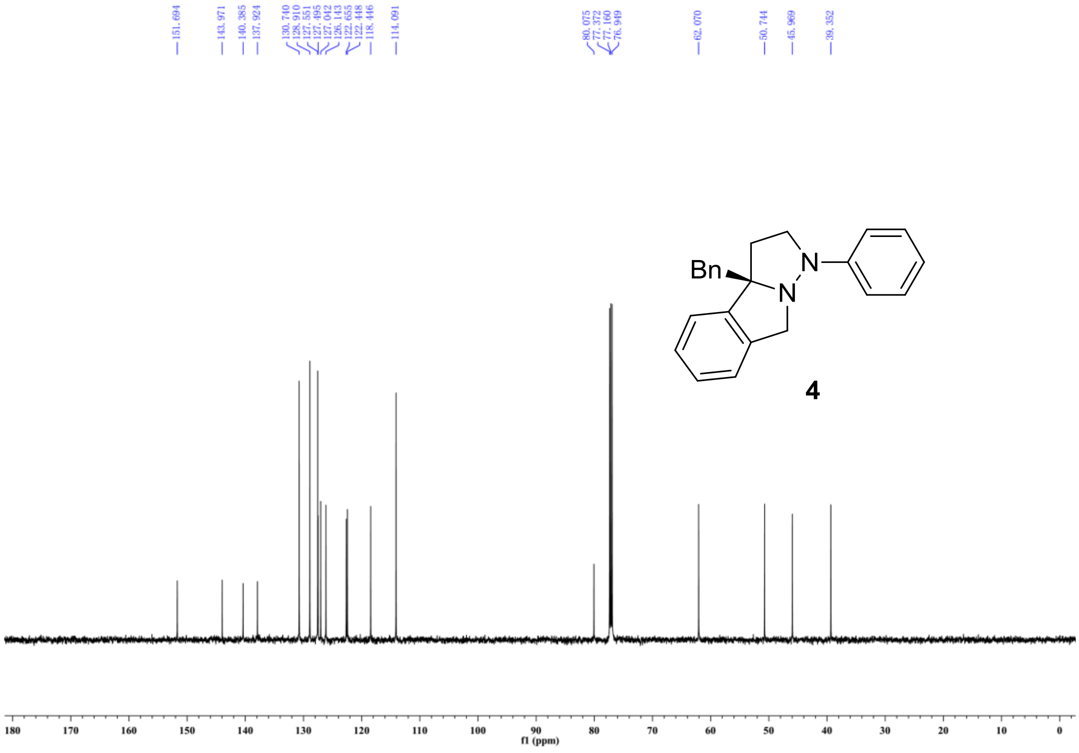

Figure S296. ^{13}C NMR of 4, related to Figure 4.

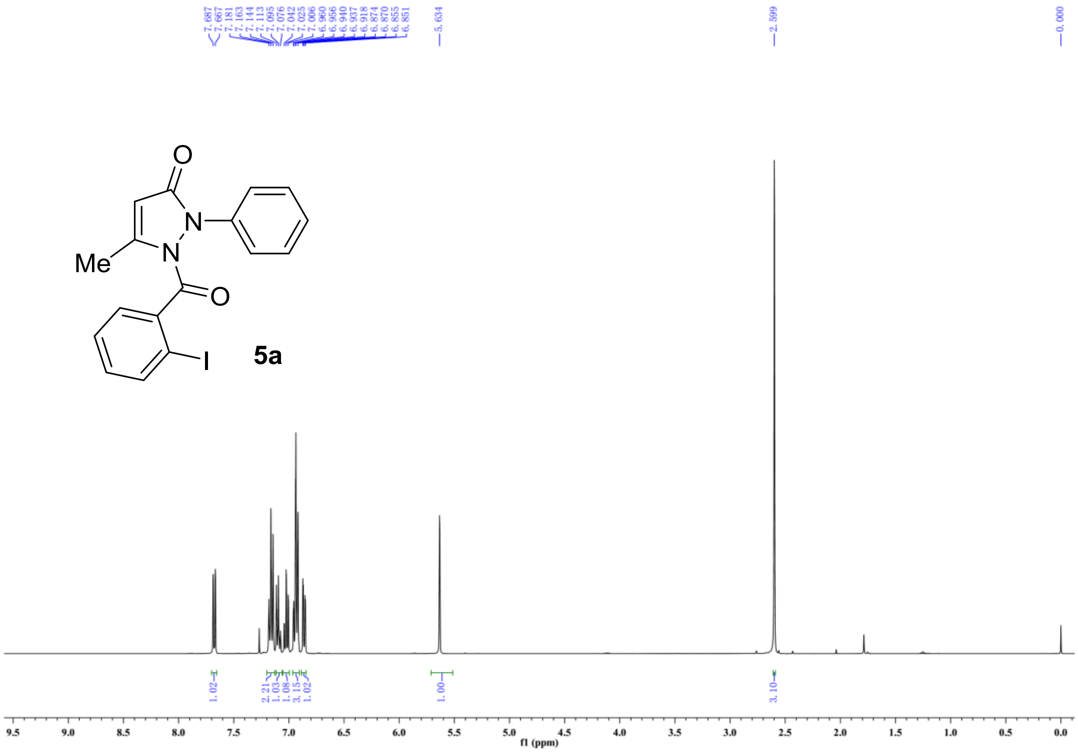

Figure S297. ^{1}H NMR of 5a, related to Figure 6.

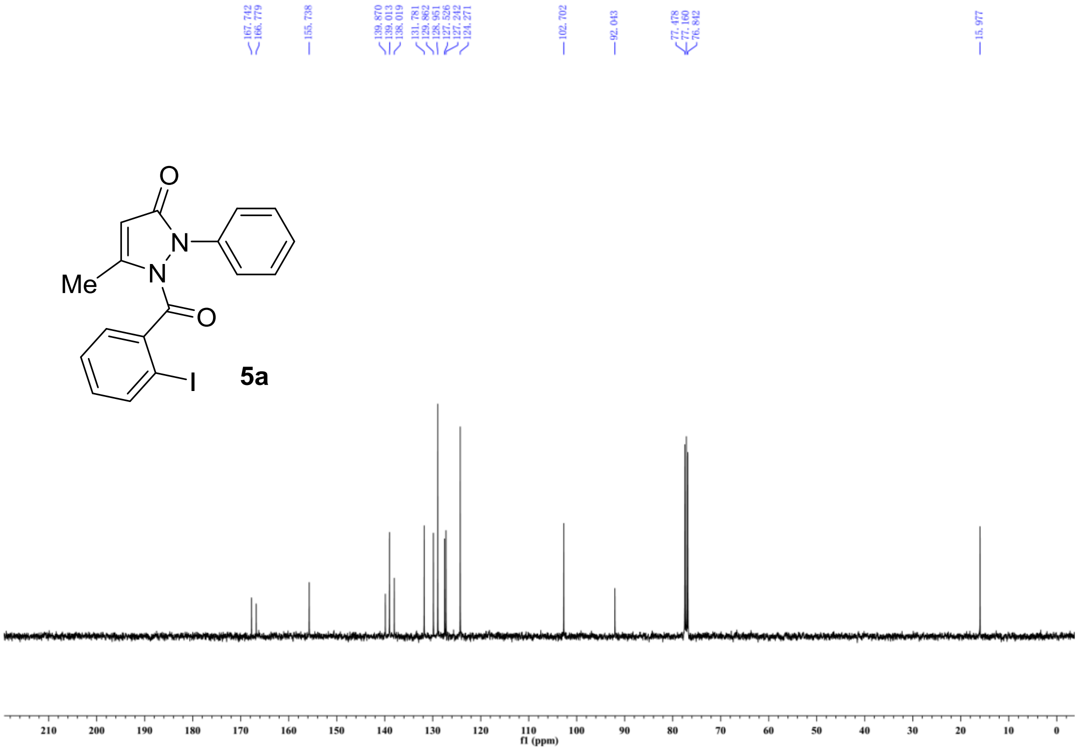

Figure S298. ^{13}C NMR of 5a, related to Figure 6.

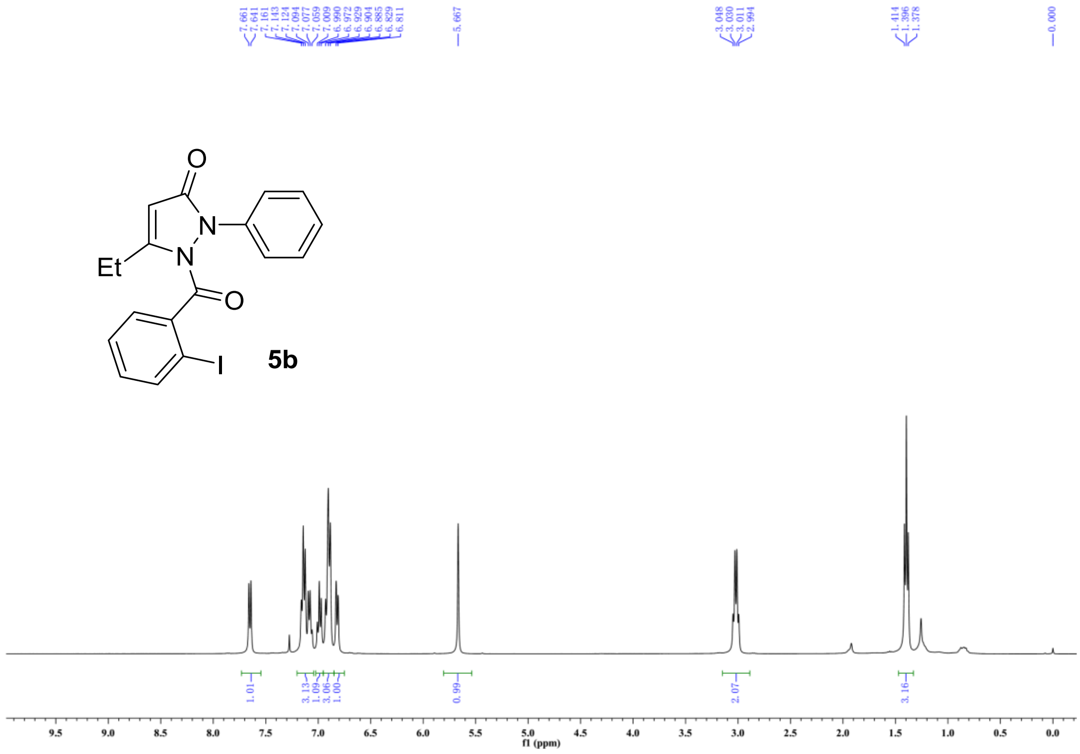

Figure S299. ^{1}H NMR of 5b, related to Figure 6.

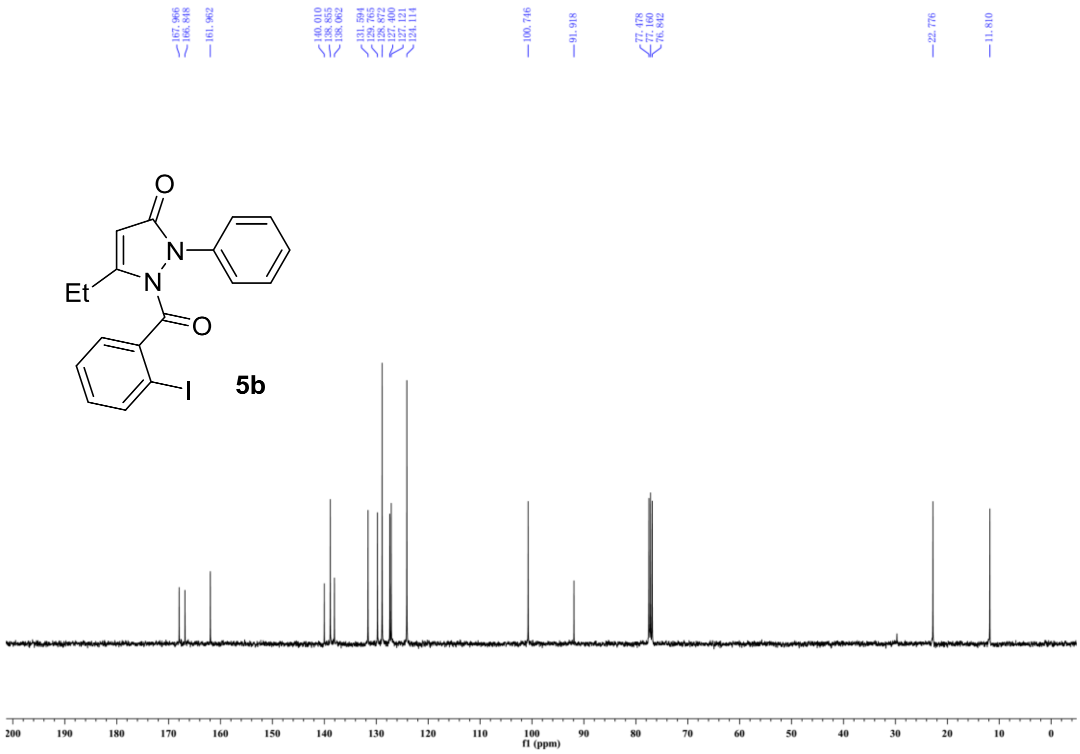

Figure S300. ^{13}C NMR of 5b, related to Figure 6.

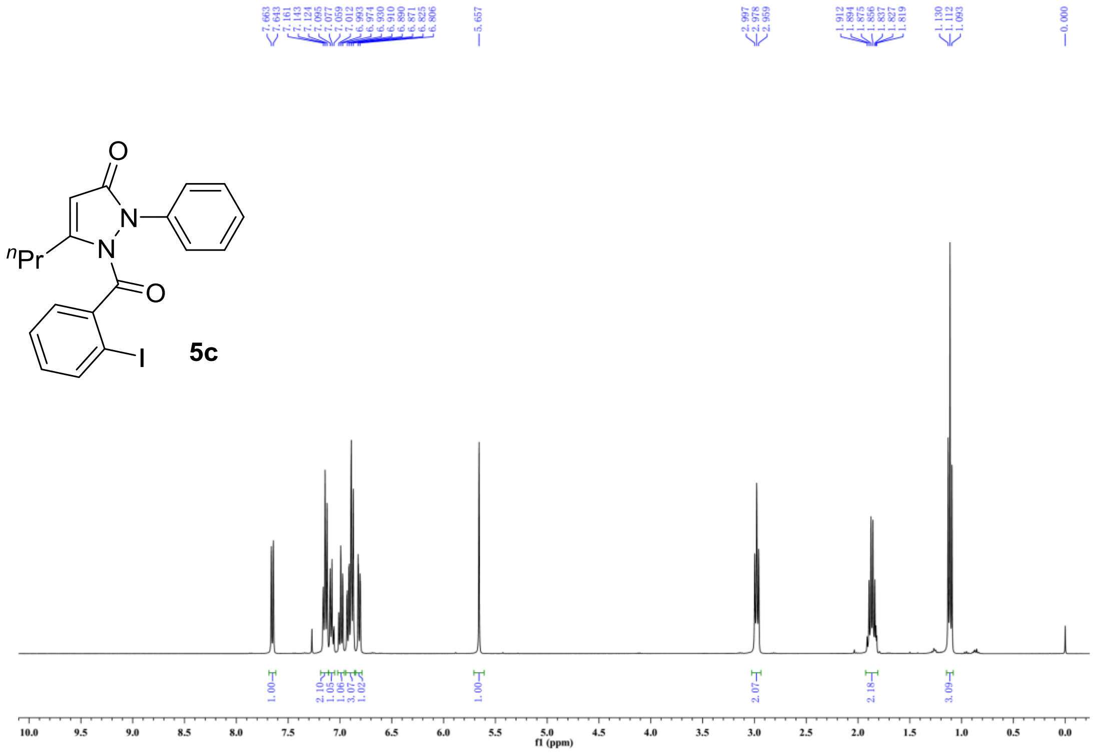

Figure S301. ^{1}H NMR of 5c, related to Figure 6.

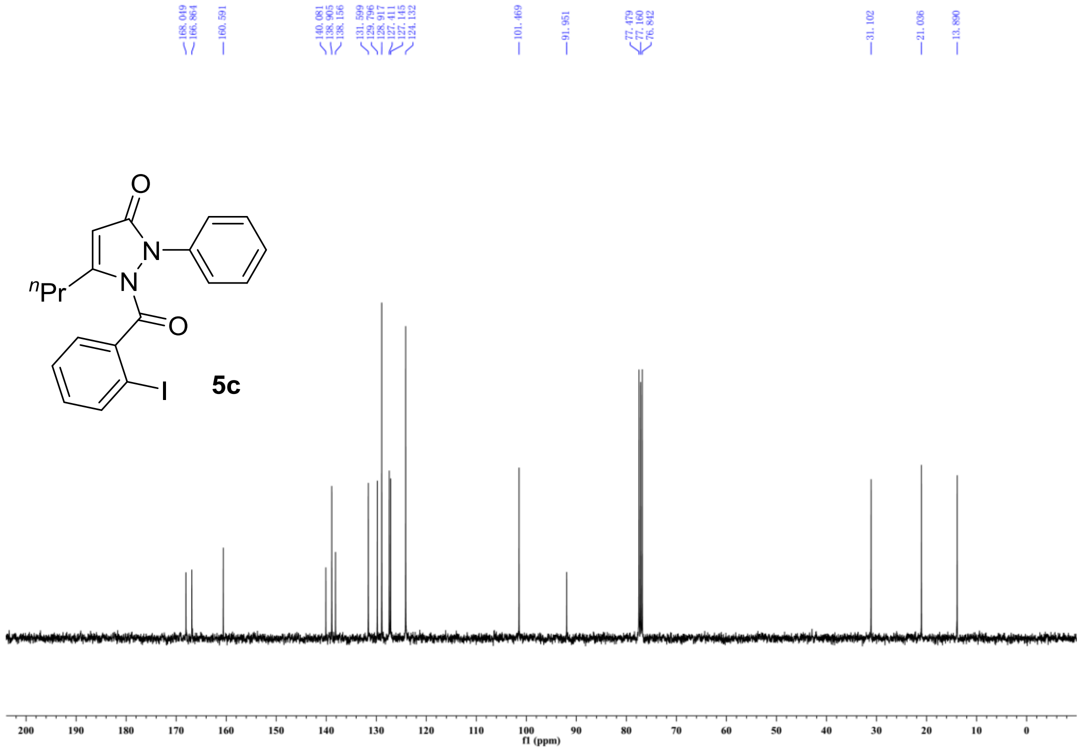

Figure S302. ^{13}C NMR of 5c, related to Figure 6.

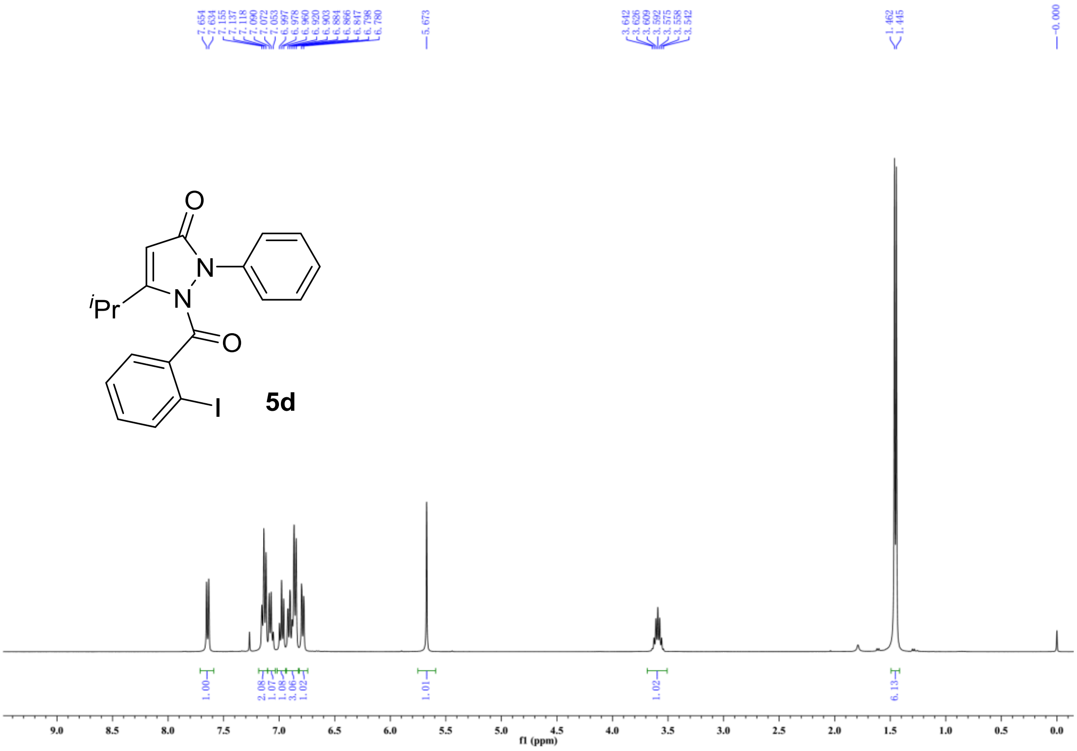

Figure S303. ^{1}**H NMR of 5d, related to Figure 6.**

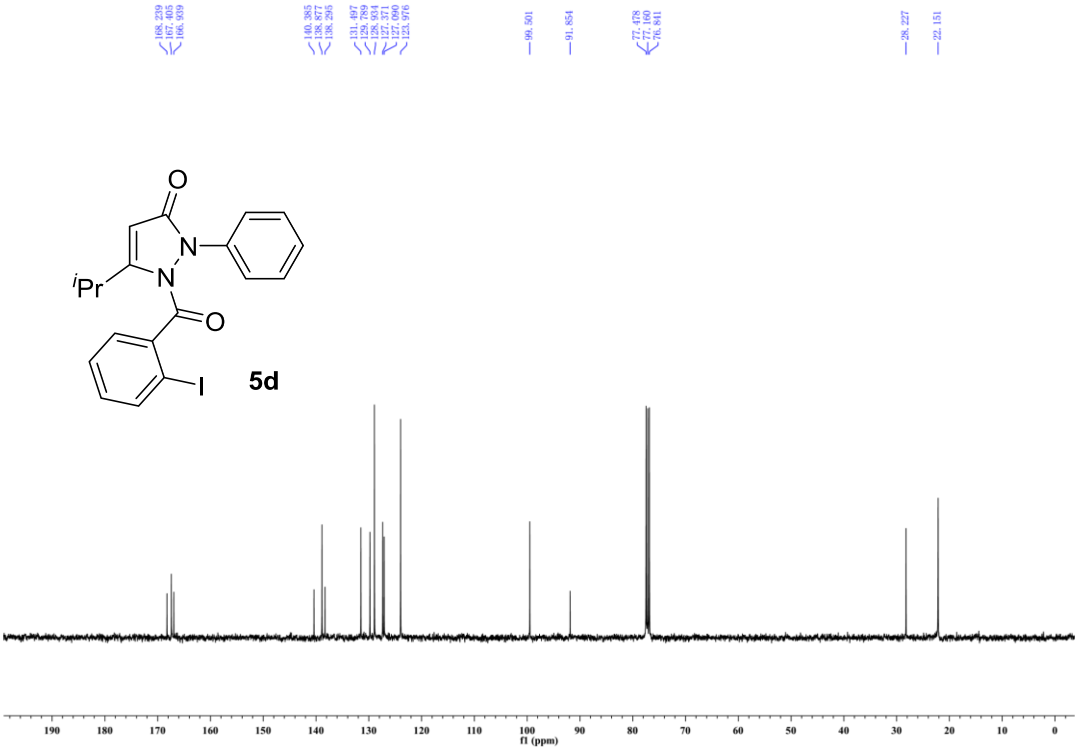

Figure S304. ^{13}**C NMR of 5d, related to Figure 6.**

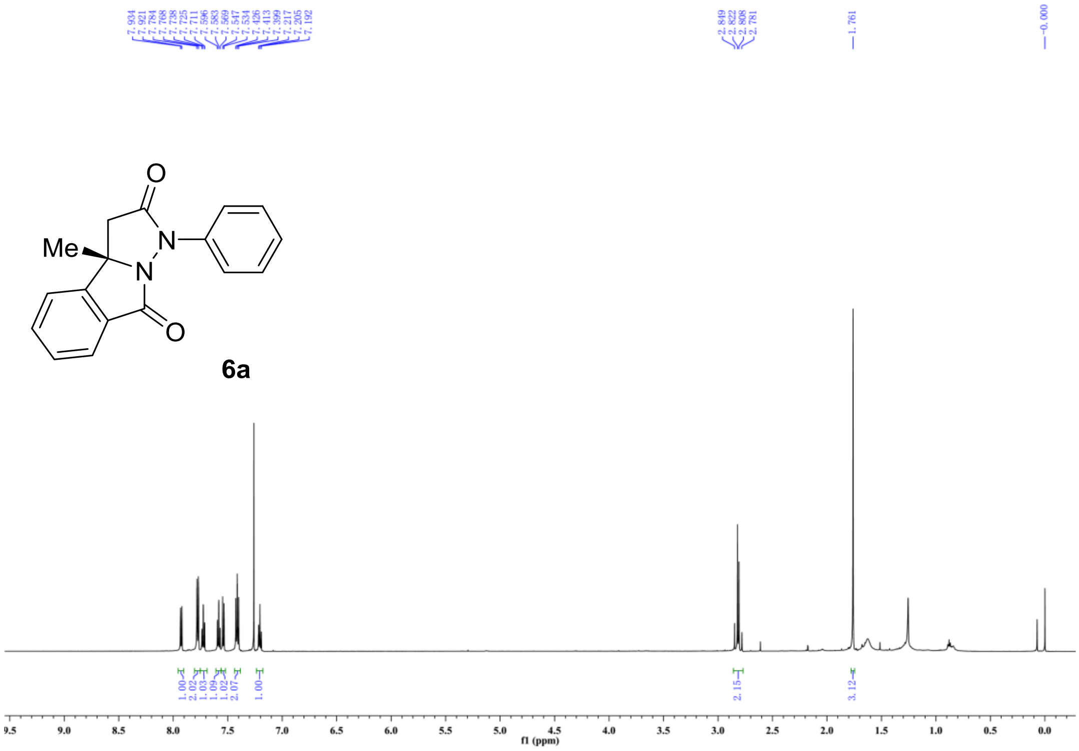

Figure S305. ^{1}H **NMR of 6a, related to Figure 6.**

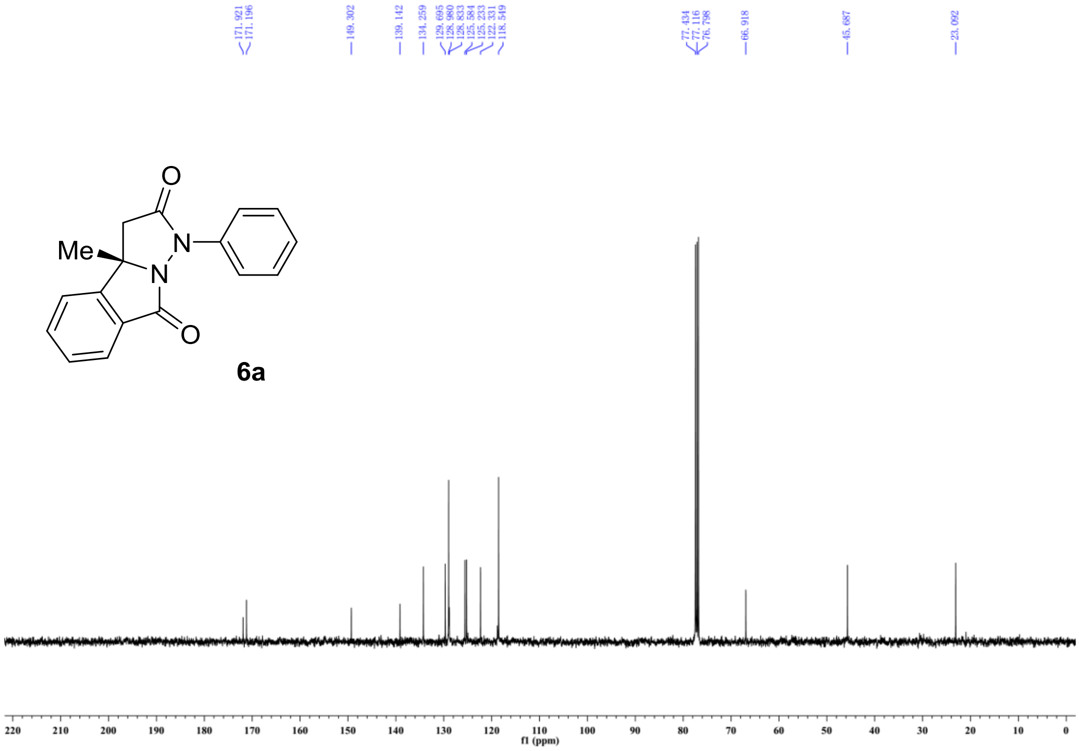

Figure S306. ^{13}C **NMR of 6a, related to Figure 6.**

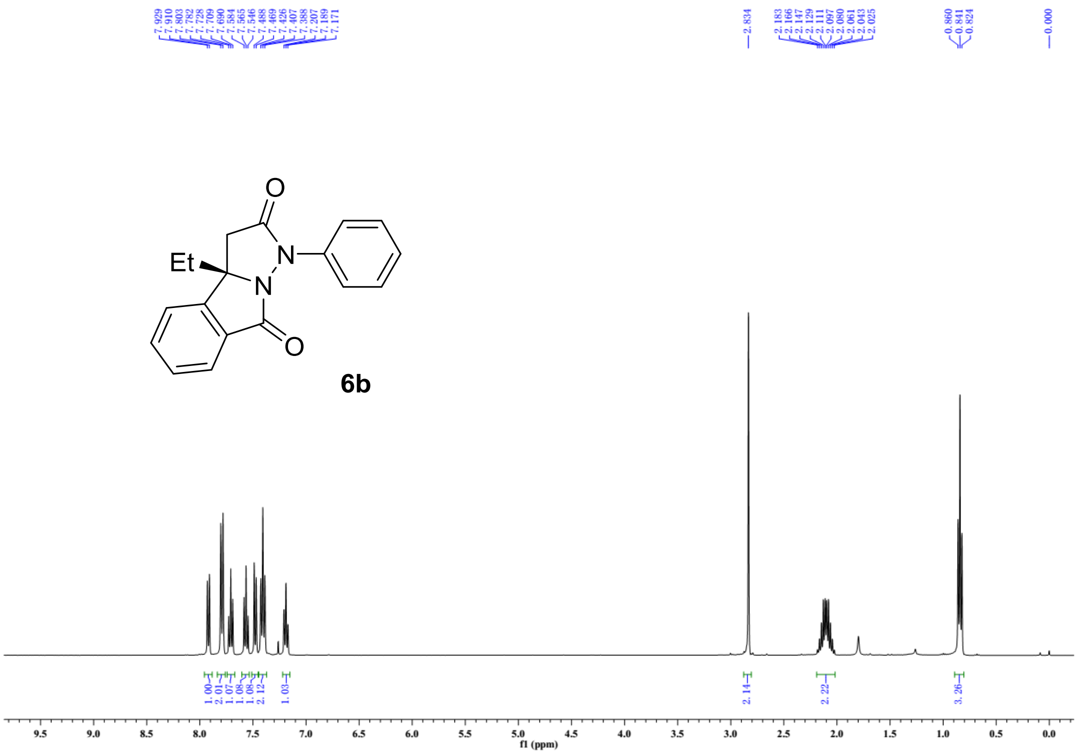

Figure S307. ^{1}H NMR of 6b, related to Figure 6.

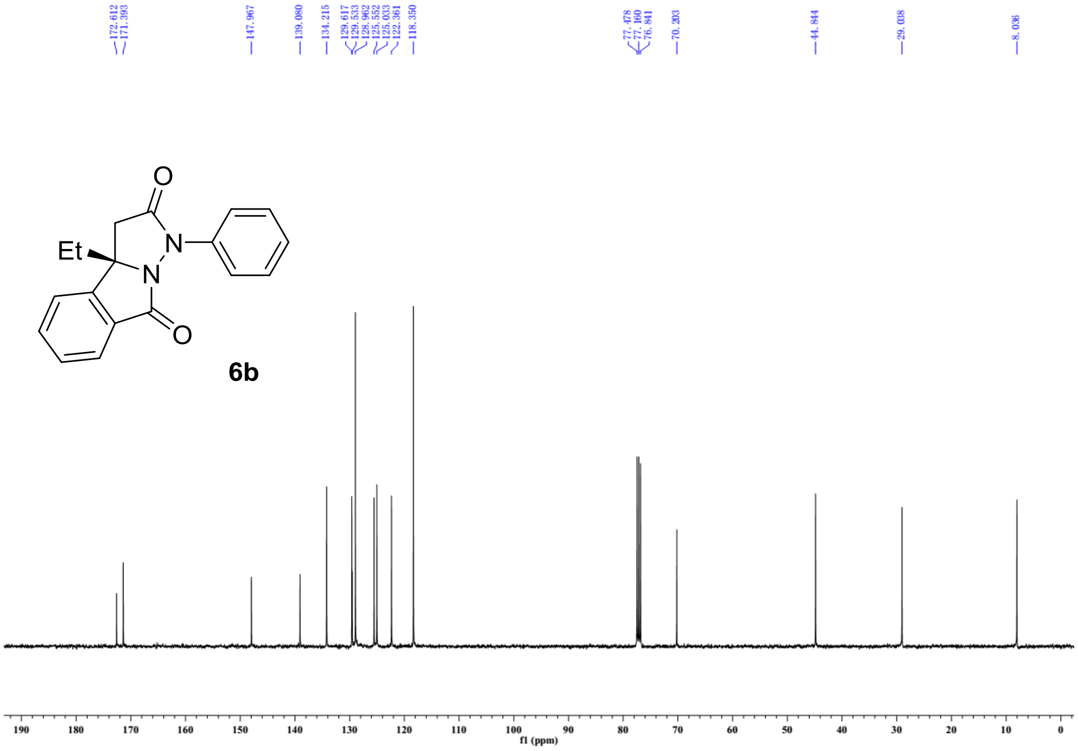

Figure S308. ^{13}C NMR of 6b, related to Figure 6.

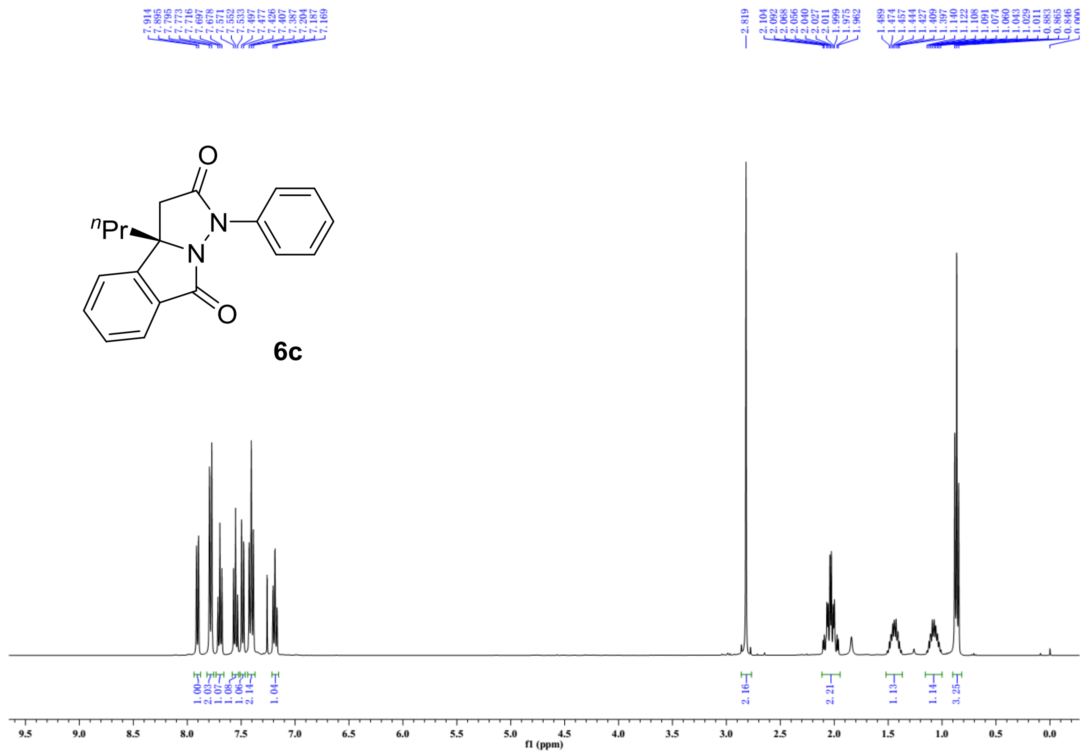

Figure S309. **^{1}H NMR of 6c, related to Figure 6.**

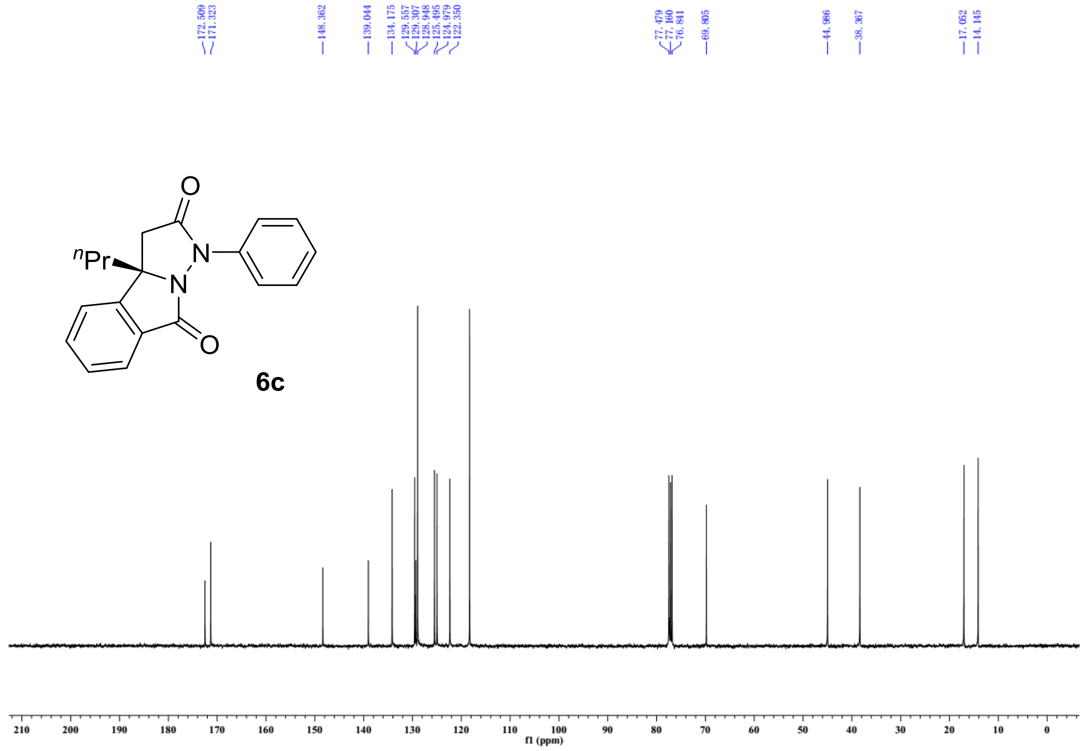

Figure S310. **^{13}C NMR of 6c, related to Figure 6.**

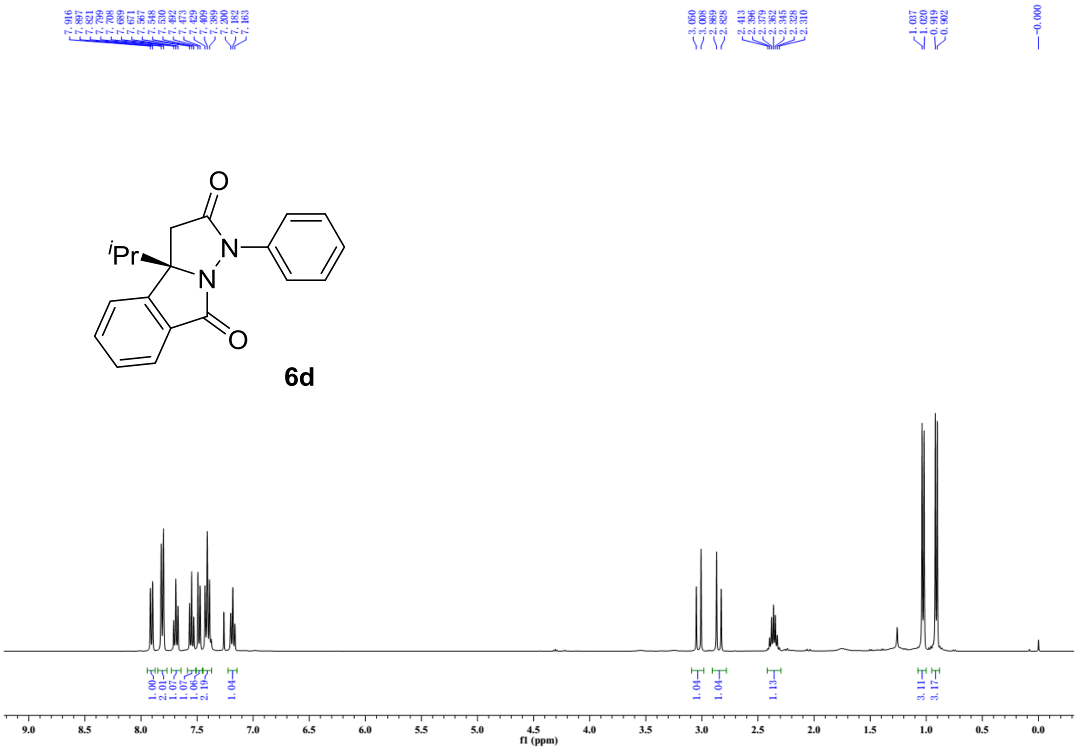

Figure S311. ^{1}H NMR of 6d, related to Figure 6.

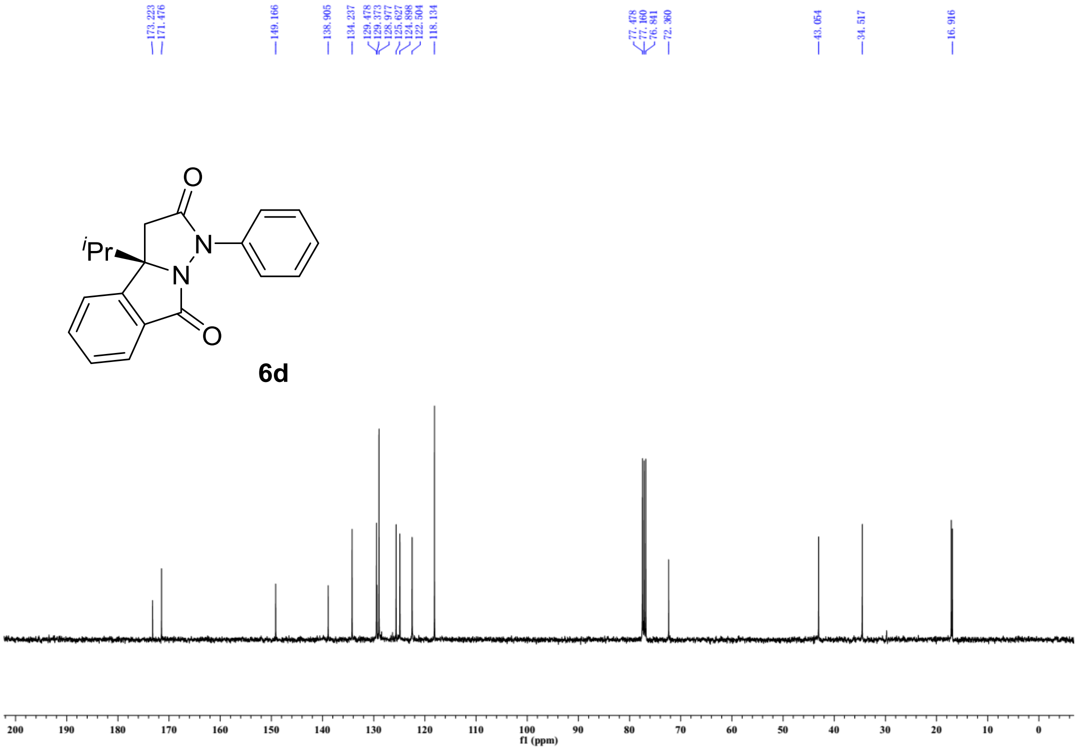

Figure S312. ^{13}C NMR of 6d, related to Figure 6.
